# An Assessment of Computational Methods for Calculating Accurate Structures and Energies of Bio-Relevant Polysulfur/Selenium-Containing Compounds

**DOI:** 10.3390/molecules23123323

**Published:** 2018-12-14

**Authors:** Sahar Nikoo, Paul J. Meister, John J. Hayward, James W. Gauld

**Affiliations:** Department of Chemistry and Biochemistry, University of Windsor, Windsor, ON N9B 3P4, Canada; nikoo@uwindsor.ca (S.N.); meisterp@uwindsor.ca (P.J.M.); jhayward@uwindsor.ca (J.J.H.)

**Keywords:** DFT, cysteine polysulfide, reactive sulfur species, gas phase basicity, proton affinity, bond dissociation enthalpy, hydrogen affinity, thermochemistry

## Abstract

The heavier chalcogens sulfur and selenium are important in organic and inorganic chemistry, and the role of such chalcogens in biological systems has recently gained more attention. Sulfur and, to a lesser extent selenium, are involved in diverse reactions from redox signaling to antioxidant activity and are considered essential nutrients. We investigated the ability of the DFT functionals (B3LYP, B3PW91, ωB97XD, M06-2X, and M08-HX) relative to electron correlation methods MP2 and QCISD to produce reliable and accurate structures as well as thermochemical data for sulfur/selenium-containing systems. Bond lengths, proton affinities (PA), gas phase basicities (GPB), chalcogen–chalcogen bond dissociation enthalpies (BDE), and the hydrogen affinities (HA) of thiyl/selenyl radicals were evaluated for a range of small polysulfur/selenium compounds and cysteine per/polysulfide. The S–S bond length was found to be the most sensitive to basis set choice, while the geometry of selenium-containing compounds was less sensitive to basis set. In mixed chalcogens species of sulfur and selenium, the location of the sulfur atom affects the S–Se bond length as it can hold more negative charge. PA, GPB, BDE, and HA of selenium systems were all lower, indicating more acidity and more stability of radicals. Extending the sulfur chain in cysteine results in a decrease of BDE and HA, but these plateau at a certain point (199 kJ mol^−1^ and 295 kJ mol^−1^), and PA and GPB are also decreased relative to the thiol, indicating that the polysulfur species exist as thiolates in a biological system. In general, it was found that ωB97XD/6-311G(2d,p) gave the most reasonable structures and thermochemistry relative to benchmark calculations. However, nuances in performance are observed and discussed.

## 1. Introduction

The chalcogens sulfur and selenium have long been known to play key roles in a diverse array of important physiological and biological processes including enzymatic mechanisms [1,2], signaling [3,4,5], and mediation and repair of oxidatively damaged biomolecules [6,7,8]. Indeed, in addition to being found within three of the 22 proteinogenic amino acids—cysteine (Cys), methionine (Met), and selenocysteine (Sec)—they are also found in many essential metabolites (e.g., thiazole). This is due to their ability to possess a broad range of oxidation states as well as bonding environments, and they can often undergo reversible redox [9]. For instance, the most abundant antioxidant in animal cells is glutathione (GSH), which can mediate the cellular redox environment through interconversion with its oxidized disulfide form, GSSG [10].

**Scheme 1:**
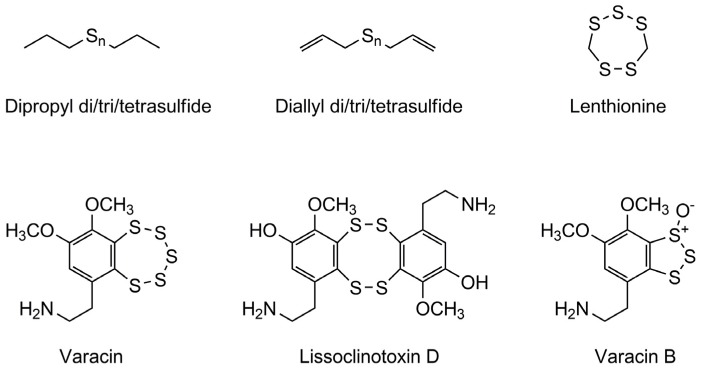
Several examples of naturally occurring polysulfur species [17,28].

Recently, it has increasingly been recognized that reactive sulfur species (RSS) comprise a rich and diverse range of physiologically important species (Scheme 1) [11]. Indeed, hydrogen sulfide is now known to be a ubiquitous essential signaling molecule that plays key roles in many physiological and inflammatory processes including blood pressure regulation, cell proliferation and apoptosis, insulin signaling, and neurotransmission [7,12,13,14,15,16]. However, sulfur can also form strong homonuclear single bonds and, as a result, can react with other sulfur species (for example, in proteins) to form a variety of polysulfur-containing RSS [17].

Previously, the presence of per- (HSSH) and polysulfides (e.g., H_2_S*_n_ n* = 3–7) in biological systems was thought to be an experimental artifacts or store for H_2_S [18]. Now, however, they are increasingly proposed or recognized as being biochemically important [17,19,20,21,22]; for instance, some polysulfides have been shown to possess antibiotic or anticancer properties [23,24]. More recently, Cysteinyl-tRNA synthetase (CysRS), an ancient enzyme with a critical role in gene-encoded protein synthesis, has also been shown to catalyze the formation of Cys-derived polysulfides [21]. This further underscores the potential biologically important activity of peptide-derived hydropersulfides [25]. Meanwhile, Se has been shown to form an Se–S intermediate in the selenoproteins thioredoxin reductase and formate dehydrogenase and plays a central role in the enzyme’s activation [26,27]. Unfortunately, due to the high reactivity of RSS, specifically per/polysulfides within biological environments, many of their properties and much of their chemistry remain unclear or even unknown.

Computational chemistry has established itself as a key tool for the study of the properties and chemistry of biomolecular systems. Several such studies have been performed on S- and, to a notably lesser extent, Se- or mixed S/Se-containing per- and poly-seleno/sulfides. For example, Brzostowska et al. used the B3LYP method to examine the intramolecular reactions of the naturally occurring polysulfur-containing pentathiepins (such as varacin, Scheme 1) that generate S_3_ and S_2_ transfer units via a tetra- or trisulfide anion, respectively [29]. Recently, the high reactivity of several smaller hydropersulfides toward alkyl, alkoxy, peroxyl, and thiyl radicals was investigated using both experimental and computational (CBS-QB3) methods [30]. It was concluded that such reactions are exothermic by 15–34 kcal mol^−1^ due to the low RSS–H bond dissociation enthalpy and high stability of perthiyl radicals [30]. Meanwhile, a computational study has used dispersion-corrected B3LYP (B3LYP-D3) to examine the role of the chalcogen atoms in the mechanism of glutathione peroxidase 4, which involves formation of a -Se–S- species [31]. Bachrach et al. used several computational methods including MP2 and B3LYP to examine possible mechanisms for nucleophilic attack at the Se in diselenides and selenosulfides and concluded that attack at Se is kinetically and thermodynamically preferred [32]. Using an ONIOM QM/MM approach, wherein the DFT method M06-2X was used for the QM region, Huang et al. examined *S*-sulfhydration via a persulfide (RSS^−^) intermediate as catalyzed by mercaptopyruvate sulfurtransferase (MST) and obtained reasonable agreement with the experiment [33]. In all of these studies, smaller basis sets (e.g., 6-31G(d)) were used to obtain structures upon which they then based their calculations of thermochemical properties. The ability of a computational study to reliably and accurately provide insights into the structures and properties of any biomolecular system usually critically depends on the choice of QM method and basis set. Thus, an essential step towards computationally studying RSS is determining appropriate methodologies.

**Scheme 2:**
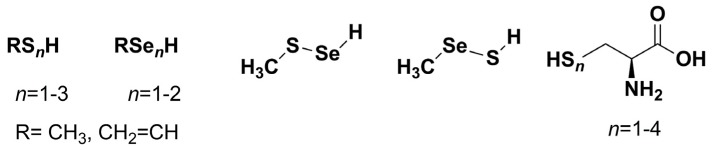
Schematic illustration of the species considered in this study.

In this present study, the ability of a range of computational, in particular density functional theory (DFT) methods [34] to provide reliable and accurate structures and thermochemical properties of biologically relevant poly-sulfur/selenium containing compounds has been assessed. More specifically, the DFT methods B3LYP, B3PW91, ωB97XD, M06-2X, and M08-HX were applied to a systematic series of biologically relevant RX*_n_*(H) (X = S/Se, *n* = 1–3, R = CH_3_, CH_2_ =CH and cysteine, Scheme 2) species. As well as their structures, a variety of key thermochemical properties including their X–X (chalcogen–chalcogen) bond dissociation enthalpies, hydrogen affinities, and gas phase basicities were examined and benchmarked.

## 2. Results and Discussion

**Structural Assessment of CH_3_XXH and CH_3_XX^−^ (X = S, Se).** We began by using the broadest variety of DFT methods (B3LYP, B3PW91, ωB97XD, M06-2X, M08-HX) and range of basis sets (6-31G(d) to 6-311++G(3df,3pd)) used in this study to obtain optimized structures for CH_3_XXH and CH_3_XX^−^ (X = S, Se). These are the smallest homoatomic persulf/selenides considered in this present study. Due to a paucity of corresponding experimental data, benchmark optimized structures were obtained at the QCISD/6-311+G(2df,p) level of theory. For simplicity, only the key C–X, X–X, and X–H (X = S, Se) distances are discussed herein and summarized in Table 1; MP2 data are shown in Table S1.

*Method Sensitivity to Basis Set Changes*: All five DFT methods showed similar overall sensitivities to changes in the basis set from 6-31G(d) to 6-311++G(3df,3pd). For example, the smallest variations were observed for the C–X and X–H (X = S, Se) bond distances; for any given method and chemical system (be it neutral or anionic) they varied by ≤ 0.020 Å. The only exceptions occurred for the C–Se bond in CH_3_SeSe^−^ when using M06-2X method, which varied overall by 0.027 Å, and the Se–H bond using MP2, which varied by 0.025 Å (Table S1). Notably, for any DFT method and chemical system the C–S bond distances showed greater variation than that of C–Se bonds. The only exceptions to this trend occurred for the C–X bonds in the CH_3_XX^−^ anions when using the B3LYP or M06-2X methods. However, the reverse trend was observed for the X–H bonds; optimized Se–H bond lengths are more sensitive to basis set choice than S–H bonds (Table 1 and Table S1).

For all chemical systems (both neutral and anionic), regardless of the choice of DFT method, the largest variations upon changing basis set were observed in their X–X bonds. For CH_3_SSH and CH_3_SS^−^ it was found that the S–S bond distances varied by 0.032–0.043 and 0.044–0.051 Å, respectively. Meanwhile, in the corresponding CH_3_SeSeH and CH_3_SeSe^−^ species the Se–Se bond distances varied by 0.024–0.029 and 0.023–0.029 Å, respectively, except for using MP2 where the variation exceeded the trend for DFT data (Table S1). That is, except for Se–H bonds, those bonds involving sulfur (i.e., C–X and X–X) in CH_3_XXH and CH_3_XX^−^ (X = S, Se) are most sensitive to the choice of basis set.

*Effects of Increasing Basis Set Size*: As can be seen in Table 1, for all DFT methods considered and for both neutral CH_3_XXH and anionic CH_3_XX^−^ (X = S, Se), similar trends were generally observed upon increasing the basis set from double- to triple-zeta, and then subsequently by inclusion of diffuse and polarization functions.

For instance, increasing the basis set from 6-31G(d) to 6-311++G(3df,3pd) generally caused a systematic shortening in the X–H and C–X bonds. Notably, one does not need to increase the basis set significantly in order to get reasonable agreement with the corresponding values obtained at the QCISD/6-311+G(2df,p) level of theory. In fact, upon changing the basis set from 6-31G(d) to 6-311G(d) (i.e., double- to triple-zeta) resulted in their lengths differing from the benchmark values by ≤ 0.015 Å. Further increases in basis set size by inclusion of diffuse (i.e., 6-311G(d,p) to 6-311+G(d,p)) or *f*- and/or *d*-polarization functions on heavy atoms or hydrogen resulted in only minor individual decreases.

In contrast, modifying the basis set showed quite different trends for the X–X (X = S, Se) bonds. For instance, for all methods assessed, improving the basis set from 6-31G(d) to 6-311G(d) resulted in an increase in their optimized length for all chemical systems of up to 0.025 Å. The only exception occurred for the M06-2X method applied to CH_3_SeSe^−^ for which the Se–Se bond shortened slightly by 0.008 Å. Notably, in CH_3_SSH and CH_3_SS^−^ the observed ranges of bond lengthening were 0.009–0.015 and 0.014–0.027 Å, respectively, with the largest increases observed when using the B3LYP method. That is to say, the anionic persulfide is more sensitive to basis set changes than the neutral hydropersulfide. In contrast, for CH_3_SeSeH and CH_3_SeSe^−^ the observed increases were quite similar lying in the range of 0.017–0.025 Å (except where noted above). The further inclusion of *p*-functions on hydrogen (i.e., 6-311G(d) to 6-311G(d,p)) or diffuse functions on heavy atoms (i.e., 6-311G(d,p) to 6-311+G(d,p)) had negligible effect on the X–X bond lengths in both the neutral CH_3_XXH and anionic CH_3_XX^−^ (X = S, Se) systems.

In general, more significant changes in the X–X lengths were observed upon inclusion of either a second set of *d*- or a set of *f*-functions on heavy atoms (i.e., 6-311G(d,p) to 6-311G(2d,p) or 6-311G(df,p)). Specifically, for both these basis set changes the S–S bonds in CH_3_SSH and CH_3_SS^−^ shortened by 0.010 to 0.017 Å, except for MP2 where the distance increases for the 6-311G(2d,p) basis set. In contrast, for the corresponding selenium containing systems, the inclusion of a second set of *d*-functions on heavy atoms marginally lengthened Se–Se bonds by ≤ 0.004 Å, while the inclusion of a set of *f*-functions on heavy atoms shortened the Se–Se bonds by 0.010–0.017 Å. Combining or adding further diffuse and polarization functions by use of the 6-311+G(2df,p) or 6-311++G(3df,3pd) basis sets respectively, resulted in all DFT methods except B3LYP giving X–X (X = S, Se) bond lengths that were markedly shorter than their corresponding benchmark values. In the case of B3LYP (which overestimates the length of these bonds) increasing the basis set to 6-311+G(2df,p) or 6-311++G(3df,3pd) was in fact required in order to get good agreement with the benchmark values.

Importantly, for all DFT methods that were considered (with the exception of B3LYP), the 6-311G(2d,p) and 6-311G(df,p) basis sets gave optimized C–X, X–X, and X–H distances for CH_3_XXH and CH_3_XX^−^ (X = S) that were in closest general agreement with their corresponding benchmark values. Meanwhile, for the corresponding Se analogues, the best performing basis sets were generally 6-311G(d), 6-311G(d,p), 6-311+G(d,p), and 6-311G(2d,p). It is also noted that the M08-HX method does not offer much if any improvement over M06-2X. In fact, when Se is in the system, it slightly underestimates the bond lengths. As such, subsequent tables showing optimized parameters will only include results obtained using the B3PW91, ωB97XD and M06-2X methods in combination with the identified preferred basis sets. It should be noted that, for completeness, the corresponding values for the other methods are included in the Supplementary Materials. Since MP2 trends were mostly similar to DFT, we do not include results obtained with this method.

**Effect of Conjugation: CH_2_CHXXH and CH_2_CHXX^−^ (X = S, Se).** These model systems were examined to gain insights into the influence of conjugation on the neutral and anionic persulfide and perselenide groups. Based on the results obtained for the CH_3_XX^−^/H systems, optimized structures were obtained using only the B3PW91, ωB97XD, and M06-2X methods in combination with the 6-311+G(d,p), 6-311G(2d,p), and 6-311G(df,p) basis sets. The most significant changes were observed in their C–X and X–X bonds, hence only these optimized values are shown in Table 2. Table S2 includes the full set of optimized parameters with all DFT functionals and the X–H bonds.

In general, upon changing basis sets and methods similar trends were observed as for the CH_3_XX^−^/H systems, though with some key differences. For instance, for X = S, the closest agreement of the optimized bond lengths with the benchmark values was obtained for the 6-311G(df,p) and 6-311G(2d,p) basis sets. In contrast, for X = Se the X–X bond is more sensitive to the choice of basis set. In particular, for CH_2_CHSeSe^−^ the best agreement with the benchmark values are obtained using the 6-311+G(d,p) or 6-311G(2d,p) basis sets; inclusion of *f*-functions results in too short an Se–Se bond. Meanwhile, for neutral CH_2_CHSeSeH it is essential to include *f*-functions in order to obtain good agreement with the corresponding benchmark values.

Comparison of the optimized bond lengths in CH_3_XX^−^/H and CH_2_CHXX^−^/H (X = S, Se) shows that for all species, at all levels of theory, the optimized C–X distance in CH_2_CHXX^−^/H is 0.04–0.05 Å shorter than in the corresponding CH_3_XX^−^/H analogue (cf. Table 1). This is also observed when structures are optimized at the M06-2X/aug-cc-pVTZ level of theory (data not shown). In contrast, the X–X bond lengths in CH_2_CHXX^−^/H (X = S, Se) are all within ±0.01 Å of their optimized values in the corresponding CH_3_XX^−^/H analogue. When X = S the X–X bond in the anion is 0.017 Å longer in the conjugated species compared to 0.023 Å in the alkyl terminated species. It is noted that this again indicates that deprotonation of RXXH (X = S, Se) causes the X–X bond to lengthen, although conjugation lessens the bond lengthening upon going to the anion.

**Effects of Mixed Sulfur/Selenium: CH_3_XYH (X = S, Se; Y = Se, S).** We then considered mixed sulfur/selenide species for which selected optimized parameters are given in Table 3. Full optimized parameters for all DFT methods and basis sets are shown in Table S3.

For all four mixed species the optimized lengths of the C–X bond (X = S, Se) are all within 0.01 Å of those obtained for their analogous persulfide or perselenide (i.e., CH_3_XXH (X = S, Se)) at the same level of theory (cf. Table 1). Thus, a similar method and basis set trends were also observed and do not require further detailed discussion. However, it should be noted that, while the X–Y bond lengths in the neutral species CH_3_SSeH and CH_3_SeSH are close to each other, the bond is consistently predicted to be marginally shorter (<0.01 Å) in CH_3_SSeH. The changes observed in the CH_3_X–Y bond length upon deprotonation (*i.e.,* CH_3_XYH to CH_3_XY^−^), depends on whether S or Se is the terminal atom. When S is the terminal atom, upon deprotonation of the thiol group the Se–S bond lengthens marginally by <0.01 Å. In contrast, when Se is the terminal atom deprotonation causes the S–Se bond to lengthen by ≥0.04 Å. The larger impact of deprotonating a selenol versus thiol group is also seen in the species shown in Table 1 and Table 2, though to a smaller extent. We found that when the sulfur atom is in the middle of the chain, it has a larger Mulliken charge compared to selenium (−0.23 vs. −0.13) in the anionic species. By comparison, the charge of the terminal chalcogen is the same in both species. Thus, repulsive interactions cause the bond length to increase when sulfur is in the center of the chain.

**Extending a Sulfide: CH_3_SSSH and CH_2_CHSSSH.** Polysulfides, but to-date not polyselenides, have been suggested to be potentially biochemically important. Thus, for completeness, we considered the effect of extending the persulfide chain. Specifically, the simplest alkyl- and conjugate-containing trisulfides, CH_3_SSSH and CH_2_CHSSSH, were examined. The C–S_1_ bond in all species had very similar optimized lengths (see Table S4 in the Supplementary Materials), for all methods and basis sets used, to those obtained for the analogous alkyl persulfides (cf. Table 1); the observed shortened C–X bond in the conjugated neutral and anionic persulfides (Table 2) was not observed in CH_2_CHSSSH, further evidence that it is due to delocalization across the persulf/selenide and conjugated R-group (see Supplementary Materials). Thus, in Table 4 only the optimized lengths of the S–S bonds are given.

As can be seen in Table 4, the B3PW91 method gives the worst agreement with the benchmark values, especially for the deprotonated (anionic) species with errors of up to 0.07 Å. Furthermore, for any DFT method, the largest errors in the optimized S_i_–S_j_ bond lengths are observed upon use of the 6-311+G(d,p) basis set. The best agreement is instead obtained using the M06-2X and ωB97XD methods, the former performing slightly better in conjunction with the 6-311G(2d,p) basis set. Indeed, their errors lie in the ranges of 0.000–0.011 Å and −0.003–0.009 Å, respectively.

Notably, in CH_3_SSSH and CH_2_CHSSSH, the CS_1_–S_2_ bond is predicted to be shorter than the S_2_–S_3_H bond by >0.02 and <0.01 Å, respectively. However, upon deprotonation of the terminal thiol group in each, i.e., formation of CH_3_SSS^−^ and CH_2_CHSSS^−^, the CS_1_–S_2_ bond lengthens significantly by ~0.05 Å, from ~2.06 and 2.07 Å in CH_3_SSSH and CH_2_CHSSSH, respectively, to approximately 2.1 and 2.12 Å. In contrast, the S_2_–S_3_ bond in CH_3_SSS^−^ and CH_2_CHSSS^−^ has shortened by 0.01–0.02 Å. Mulliken charges on S_1_ were found to decrease more than they do on S_2_ upon deprotonation in the benchmark calculation, for both alkyl and conjugated polysulfur species. However, the difference between the two was small compared to the large charge on S_3_; it becomes much more negative upon deprotonation. This indicates that there is some degree of charge delocalization along the sulfur chain (Table S5).

**Obtaining Reliable and Accurate Thermochemistry for CH_3_SSH and CH_2_CHSSH.** Two of the most common goals when applying computational methods to the study of chemical problems are obtaining reliable and accurate optimized structures and thermochemical data. For biochemical or related problems, common reactions often require knowledge of proton affinities (PAs), gas phase basicities (GPBs), hydrogen affinities (HAs) and bond dissociation enthalpies (BDEs). Furthermore, given the size of the systems often encountered there is simultaneously considerable interest in identifying a DFT-based approach for calculating such properties. Thus, having assessed the use of DFT methods for the accurate optimization of structures, we also assessed the ability of the DFT methods B3LYP, B3PW91, ωB97XD, M06-2X, M08-HX, in combination with a range of basis sets to provide reliable and accurate biochemically-relevant thermochemical data.

Given the poor performance of B3LYP and minimal improvement of M08-HX over M06-2X in obtaining reliable structures, vide supra, here we only report the performance of B3PW91, ωB97XD, and M06-2X, unless otherwise noted. Furthermore, we have focused on reporting basis sets that for such systems have been previously used (e.g., 6-31G(d)), shown herein to be most consistently reliable (i.e., 6-311G(2d,p)), or often used for calculating accurate thermochemistry (i.e., 6-311+G(2df,p) and 6-311++G(3df,3pd)). The results obtained are summarized in Table 5, although the data for all functionals and basis sets that were studied are shown in Tables S6–S11.

The benchmark values were again obtained using the QCISD/6-311+G(2df,p) level of theory. The thermochemical values obtained for the conjugated persulfides compared to the alkyl persulfides reflects the trends observed structurally. For instance, the BDE(S–S) for CH_2_CHSSH is slightly higher by 5.7 kJ mol^−1^ than that of CH_3_SSH, while the PA and GPB of CH_2_CHSS^−^ are 23.6 and 24.4 kJ mol^−1^ lower, respectively, than those of CH_3_SS^−^. This is due to delocalization across the persulfide and CH_2_CH group in CH_2_CHSSH/^−^. Meanwhile, the hydrogen affinity of CH_2_CHSS^•^ is predicted to be only marginally higher than that of CH_3_SS^•^ by 0.8 kJ mol^−1^. Notably, we did see spin contamination in the QCISD calculations with a spin of 0.88. This may be the reason for lower energies calculated by QCISD compared to DFT methods where spin contamination was much lower (0.77).

A DFT-based model was determined to be accurate if it gave values within 10 kJ mol^−1^, generally held to be experimental accuracy, of the benchmark values. From Table 5 it can be seen that not all methods or basis set choices were reliable, nor did all thermochemical properties exhibit the same method/basis set requirement. For instance, for accurate determination of the BDE(S–S) of both CH_3_SSH and CH_2_CHSSH, the best performance was observed for B3PW91 and ωB97XD in combination with the 6-31G(d) basis set. The former method slightly underestimating compared to the benchmark value while the latter overestimated slightly. The M06-2X method only gave an accurate BDE(S–S) for CH_2_CHSSH and again when using the 6-31G(d) basis set. In general, the use of basis sets larger than 6-31G(d) gave BDE(S–S) values that are markedly higher (14–36 kJ mol^−1^) than those obtained at the QCISD/6-311+G(2df,p) level of theory.

For the PA and GPB of the CH_3_SS^−^ and CH_2_CHSS^−^ anions the M06-2X method again only gives accurate values when used in combination with the 6-31G(d) basis set. In contrast, the B3PW91 and ωB97XD give accurate values for all the basis sets considered. However, the triple-zeta basis sets (6-311G(2d,p), 6-311+G(2df,p), and 6-311++G(3df,3pd)) gave best agreement with calculated values within 5 kJ mol^−1^ of their corresponding benchmark value (see Table 5).

The calculated values of the hydrogen affinity of CH_3_SS^•^ and CH_2_CHSS^•^ follow almost the same method and basis set trends and accuracy as that observed for the PA and GPB of CH_3_SS^−^ and CH_2_CHSS^−^. Namely, the B3PW91 and ωB97XD methods in conjunction with any of the basis sets considered give calculated values within ±10 kJ mol^−1^. The only exception occurs when at the B3PW91/6-31G(d) level of theory for CH_2_CHSS^•^, which gives a HA value 10.7 kJ mol^−1^ lower than the corresponding QCISD/6-311+G(2df,p) benchmark value (see Table 5). Meanwhile, the M06-2X method is inconsistent; for CH_3_SS^•^ only the 6-31G(d) and 6-311+G(2df,p) basis sets give values within 10 kJ mol^−1^ of the benchmark values while for CH_2_CHSS^•^ it gives good agreement for all basis sets considered herein.

Several overall trends are suggested in this examination of the performance of the DFT methods B3PW91, ωB97XD, and M06-2X, in conjunction with a range of basis sets, for the noted important thermochemical properties. In particular, the M06-2X functional is the least consistent and usually gives values that differ from the benchmark values by more than 10 kJ mol^−1^. Furthermore, for all DFT functionals the values obtained using the 6-311+G(2df,p) basis set are within 4 kJ mol^−1^ of the corresponding values obtained using the much larger and more expensive 6-311++G(3df,3pd) basis set. Hence, for the remainder of this report, for simplicity, only thermochemical values obtained using the B3PW91 and ωB97XD functionals in combination with basis sets no larger than 6-311+G(2df,p) are discussed, unless otherwise noted (see Tables S6–S11 for the complete datasets).

**Thermochemistry of Selenium-Containing Species.** As noted above in the structural assessment, for selenium containing species considered herein; that is CH_3_SeSe^•^/^−^/H, CH_2_CHSeSe^•^/^−^/H, CH_3_SSe^•^/^−^/H, and CH_3_SeS^•^/^−^/H, the smallest consistently reliable basis set was 6-311+G(d,p), though with exceptions as noted. Hence, for these species we have limited our discussion herein to results obtained using B3PW91 and ωB97XD in conjunction with the 6-311+G(d,p) and 6-311+G(2df,p) basis sets. The results are shown in Table 6.

As for the analogous purely sulfur-containing species (cf. Table 5), the calculated PAs and GPBs of all species obtained using B3PW91 or ωB97XD with either basis set choice gives values within 10 kJ mol^−1^ of the corresponding benchmark values. A similar consistency is observed for the calculated HAs, though with some exceptions. In particular, at the ωB97XD/6-311+G(d,p) level of theory the calculated HAs of CH_2_CHSeSe^•^ and CH_3_SSe^•^ are 11.2 and 12.4 kJ mol^−1^, respectively, higher than their corresponding QCISD/6-311+G(2df,p) values. Again, as seen in Table 5, the calculated RX—YH BDEs of all species are generally markedly overestimated by 12.7–26.3 kJ mol^−1^ using either DFT method and basis set. Only three values fall within the desired 10 kJ mol^−1^ error margin and all occur for the mixed chalcogen species; all using the 6-311+G(d,p) basis set.

The thermochemical values provided in Table 5 and Table 6 show several key differences between persulfides and perselenides and the mixed chalcogens. These are most clearly and simply illustrated by examination of the calculated benchmark values. In particular, increasing the number of Se atoms in an RX–YH bond reduces its BDE as shown by comparing those of CH_3_SSH (236.3 kJ mol^−1^), CH_3_SSeH (218.7 kJ mol^−1^), CH_3_SeSH (223.2 kJ mol^−1^), and CH_3_SeSeH (202.7 kJ mol^−1^). In addition, a conjugated group adjacent to the RX–XH group increases its BDE slightly by 3–6 kJ mol^−1^. Meanwhile, the HAs are reasonably consistent and depend primarily on whether the formal radical terminal is a sulfur or selenium. For the former, all values lie within the range 282.7 kJ mol^−1^ (CH_3_SS^•^) to 286.1 kJ mol^−1^ (CH_3_SeS^•^), while the latter are notably lower, between 265.9 kJ mol^−1^ (CH_2_CHSeSe^•^) and 267.3 kJ mol^−1^ (CH_3_SeSe^•^ and CH_3_SSe^•^).

Comparison of the PAs and GPBs of these perselenides and mixed per-sulf/selenides with their analogous persulfides (cf. Table 5), shows several interesting trends. It is noted that, for simplicity, as the observed trends were the same for all the DFT methods, the values discussed here refer to those obtained at the benchmark level of theory. Firstly, systematically increasing the occurrence of Se in a per-chalcogenide group decreases their PA and GPB values. For instance, the PAs of CH_3_SS^−^, CH_3_SeS^−^, CH_3_SSe^−^, and CH_3_SeSe^−^ are 1445.4 kJ mol^−1^, 1438.9 kJ mol^−1^, 1411.3 kJ mol^−1^, and 1404.3 kJ mol^−1^. Simultaneously, their GPB values decrease from 1415.1 kJ mol^−1^, to 1408.6 kJ mol^−1^ and 1381.1 kJ mol^−1^, to 1374.0 kJ mol^−1^. In addition, replacing CH_3_- with CH_2_CH- decreases the PA and GPB values of the pure persulfides and perselenides by 24–25 and 17–18 kJ mol^−1^, respectively. This perhaps reflects a larger delocalization when a conjugated group is adjacent *and* that the effect is less for the selenides.

*Extending the persulfides to trisulfides.* We also examined the effect of extending the persulfide group by an additional sulfur to a trisulfide; specifically, we considered key bio-relevant thermochemistry of CH_3_SSS^•^/^−^/H and CH_2_CHSSS^•^/^−^/H. Based on the trends observed for persulfide systems, thermochemical values were obtained using the B3PW91 and ωB97XD DFT methods in combination with the 6-311+G(2df,p) basis set, and again at the QCISD/6-311+G(2df,p) benchmark level of theory (all other data shown in Table S9). As can be seen in Table 7, both DFT methods give thermochemical results in generally good agreement with the benchmark values with B3PW91 slightly preferred, although the differences in average errors are relatively small.

Comparison of the calculated benchmark values of the trisulfides with those of the corresponding persulfides (cf. Table 6) shows that the BDE of RS_1_S_2_—S_3_H (i.e., the BDE of the terminal S_2_–S_3_ bond), where R = CH_3_- and CH_2_CH-, decreases significantly by 53.3 and 56.3 kJ mol^−1^, respectively. Similarly, their calculated PAs and GPBs decrease markedly by 31–33 and 18–19 kJ mol^−1^ for R = CH_3_- and CH_2_CH-, respectively. As a result of these changes the RSS–SH BDE, and PAs and GPBs of RSSS^−^ all lie within a narrower range (≤13 kJ mol^−1^) of each other. This perhaps reflects in part a decrease in the influence of the R group on the increasingly removed S–SH bond. In contrast, the hydrogen affinities of CH_3_SSS^•^ and CH_2_CHSSS^•^ are 6.7 and 11.5 kJ mol^−1^ higher than that of their corresponding persulfide analogues with values of 289.4 and 295.0 kJ mol^−1^, respectively.

**Figure 1:**
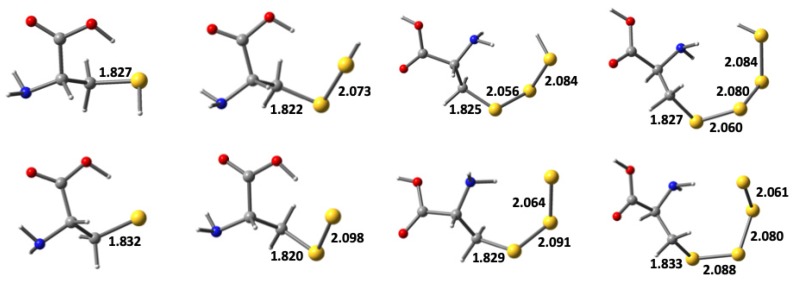
Optimized structures (with C–S and S–S bond lengths given in ångstroms (Å)) obtained at the ωB97XD/6-311G(2d,p) level of theory for neutral cysteine and its polysulfide (CysS–S*_n_*H *n* = 1–3) derivatives (top row), and their corresponding anionic deprotonated derivatives (CysS–S*_n_*^−^ *n* = 0–3; bottom row).

**Cysteine-derived polysulfides: _Cys_SS*_n_*H (*n* = 1–3).** Within biological systems, as noted above, per- and polysulfide derivatives of cysteine play important roles. Hence, we also considered the structures and thermochemistry of such species using the ωB97XD/6-311G(2d,p) level of theory. This level was selected as it emerged in our earlier study as the most able to provide reliable structures and thermochemistry for related model systems (see above). Selected parameters of optimized structures obtained for _Cys_SS*_n_*H (*n* = 0–3) and _Cys_SS*_n_*^−^ (*n* = 0–3) are provided in Table 8 and representative optimized structures are shown in Figure 1, at this level of theory.

As can be seen, for both the neutral and deprotonated derivatives, the *r*(C−S) bond length decreases slightly upon forming a perthiol/sulfide group, but then gradually lengthens, essentially returning to its length in cysteine, as one goes to the corresponding trithiol/sulfide and tetrathiol/sulfide derivatives. It is also noted that for both _Cys_SSH and _Cys_SS^−^, the C–S and S–S bond lengths are in close agreement with the corresponding values obtained at the same level of theory for CH_3_SSH (1.816 and 2.067 Å) and CH_3_SS^−^ (1.816 and 2.092 Å), respectively (cf. Table 1). In addition, for the neutral polysulfides the S–S bond in the chain that is farthest removed from the alkyl group has the longest length, with the S–S bonds being sequentially shorter the closer they are to the alkyl group. In contrast, for the corresponding anionic deprotonated series (_Cys_SS*_n_*^−^, *n* = 0–3), the opposite trend is observed; the S–S bond in the chain farthest from the alkyl group is shortest, and they get longer the closer they are to the alkyl group. In the latter species this trend may reflect a diminishing effect of the negative charge on the terminal sulfur atom the further removed the bond.

**Figure 2:**
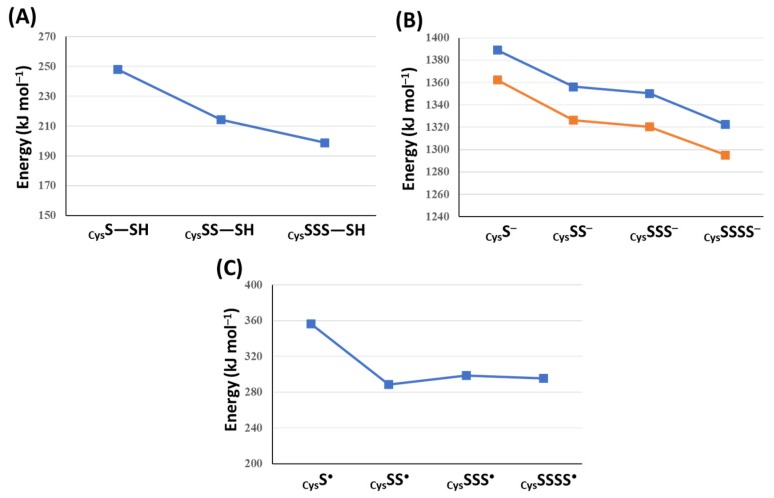
Calculated (at the ωB97XD/6-311G(2d,p) level of theory) values of (**A**) RS*_n_*—SH BDEs for _Cys_SS*_n_*H (*n* = 1–3), (**B**) PAs (blue) and GPBs (orange) of _Cys_SS*_n_*^−^ (*n* = 0–3), and (**C**) HAs of _Cys_SS*_n_*^•^ (*n* = 0–3). All energies are in kJ mol^−1^.

*Thermochemistry of cysteine-derived polysulfides.* As for the other species considered above, we calculated the RS*_n_*–SH homolytic BDEs for _Cys_SS*_n_*H (*n* = 1–3), the PAs and GPBs of the _Cys_SS*_n_*^−^ (*n* = 0–3) series of derivatives, and the hydrogen affinities (HAs) of the _Cys_SS*_n_*^•^ (*n* = 0–3) series of species. However, all values were again only obtained using the chosen ωB97XD/6-311G(2d,p) level of theory. The results obtained are shown in Figure 2A–C.

The calculated RS*_n_*–SH (*n* = 1–3) homolytic bond dissociation enthalpies, that is, the BDE of the terminal S–SH bond in the perthiol chain decreases by 33.7 kJ mol^−1^ from 248.1 to 214.4, upon going from the perthiol (*n* = 1) to trithiol (*n* = 2). It then decreases a further 15.4 kJ mol^−1^ to 199 kJ mol^−1^ upon increasing to the chain further to the tetrathiol (*n* = 3); Figure 2A. This trend suggests that while the strength of the terminal S–SH bond does weaken as the chain is lengthened, it approaches a limiting value below 199.0 kJ mol^−1^, although possibly not too much lower than that. In contrast, as seen in Figure 2C, the hydrogen affinities of the _Cys_SS*_n_*^•^ (*n* = 0–3) species decrease significantly by 67.8 kJ mol^−1^ upon going from a cysteinyl thiyl radical (*n* = 0; 356.5 kJ mol^−1^) to the perthiyl radical (*n* = 1; 288.7 kJ mol^−1^). However, extending the chain further to *n* = 2 or *n* = 3 results in a slight increase in the HAs to 298.7 kJ mol^−1^ and 295.6 kJ mol^−1^, respectively. This suggests that at least for HAs of the polysulfur radicals, they are reasonably constant for the perthiyl and beyond.

Figure 2B shows that as the polysulfide chain is extended from the cysteine thiolate to _Cys_SSSS^−^ both the calculated PAs and GPBs decrease. However, while there are significant decreases of 33.0 (PA) and 35.9 (GPB) kJ mol^−1^ going from the thiolate (_Cys_S^−^) to perthiolate (_Cys_SS^−^), this does not appear to continue upon extending the chain further. Indeed, extending the chain to _Cys_SSS^−^ results in only comparatively small further decreases of 5.8 and 5.9 kJ mol^−1^ to 1350.5 and 1320.6 kJ mol^−1^. However, upon extending the chain to _Cys_SSSS^−^, larger decreases in both the PA and GPB are again observed; they decrease by 27.9 and 25.4 kJ mol^−1^ to 1322.6 and 1295.2 kJ mol^−1^. This may in part reflect that in the cysteinyl polysulfide derivatives, weak intramolecular hydrogen bonding was observed in some optimized structures between the terminal S^−^ center and the cysteine’s amino group. This interaction would also help decrease PA and GPB values. It should also be noted that comparison of the calculated PAs and GPBs of the cysteine-derived sulfides _Cys_SS^−^ and _Cys_SSS^−^ with that of their smaller corresponding analogs CH_3_SS^−^ (cf. Table 5) and CH_3_SSS^−^ (cf. Table 7) shows that that those of the former two species are markedly lower by 60-90 kJ mol^−1^. Thus, overall, these results suggest that in biological systems, extending the polysulfide chain will increase its likelihood of being deprotonated and that hydrogen bonding may help to stabilize such anions.

## 3. Computational Methods

All calculations were performed using the Gaussian 09 [35] and Gaussian 16 [36] suites of programs. Optimized geometries for a systematic series of biologically relevant chemical models, shown in Scheme 2, were obtained using a variety of conventional wavefunction and density functional theory methods applied in conjunction with a range of Pople basis sets from 6-31G(d) to 6-311++G(3df,3pd).

Specifically, the hybrid DFT methods B3LYP and B3PW91, comprised of Becke’s three parameter exchange functional [37] (B3) in combination with the Lee, Yang, and Parr correlation functional [38] (LYP) or the Perdew and Wang functional [39] (PW91), were assessed. B3LYP is commonly applied in the treatment of biomolecular systems while B3PW91 has been used previously for systems containing sulfur and selenium [40,41]. In addition, two *meta*-GGA functionals were assessed; M06-2X [42], a commonly employed functional in the study of enzymatic catalysis, and the more recently developed related functional M08-HX [43]. For this functional, optimized structures were obtained using Gaussian 16 whereas all other structures were obtained with Gaussian 09. Furthermore, the double-hybrid range-corrected functional ωB97XD [44] was also evaluated to further examine any effects of dispersion correction on geometric or energetic parameters. Geometries optimized at these levels of theory were compared to those obtained using the ab initio MP2 (see Supplementary Materials) and QCISD methods. As DFT methods are the preferred choice for biochemical systems due to their computational cost and reliability, the MP2 results are only given in the Table S1 for our baseline geometry assessment of CH_3_SSH/^−^ and CH_3_SeSeH/^−^. The Minnesota family of functionals (M06-2X and M08-HX) are more empirical and explicitly contain dispersion correction while B3LYP only contains three empirical paraments and has no explicit correction for dispersion.

All optimized structures were confirmed to be minima by harmonic vibrational frequency calculations performed at the same level of theory. These were also used to determine zero-point vibrational energy (ZPVE) and enthalpy corrections for subsequent calculations of a range of their thermochemical properties including proton affinities (^298.15K^PA_A–_ = –H = –(H_AH_ – H_A–_ – H_H+_)), gas-phase basicities (^298.15K^GPB_A–_ = –G = –(–PA – T(S_AH_ – S_A–_ – S_H+_)), and hydrogen affinities (^298.15K^HA_A_ = –H = –(H_AH_ – H_A_ – H_H_)) [45,46]. In addition, homolytic bond dissociation enthalpies were calculated for production of the **^·^**SH or **^·^**SeH radicals (^298.15K^BDE_AX–XH_ = H = H_AX•_ + H_XH•_ – H_AXXH_).

## 4. Conclusions

The reliability and accuracy of several commonly used DFT functionals (e.g., B3LYP, B3PW91, ωB97XD, M06-2X, M08-HX) as well as MP2 was assessed for a systematic series of bio-relevant polysulfur/selenium-containing systems. In particular, optimized structures and thermodynamic properties of a range of RX*_n_*(H) (X = S, Se, R = CH_3_, CH_2_CH, and cysteine, *n* = 1–4) were examined with a variety of Pople basis sets of increasing size. We offer the following conclusions from this detailed study:

1. Evaluation of the bond lengths in CH_3_XXH/^−^ (X = S, Se, cf. Table 1) showed that the S–S bond is the most sensitive to changes in basis set. The smallest basis set used (6-31G(d)) frequently resulted in optimized geometries that were very similar to the benchmark given by QCISD/6-311+G(2df,p), although the preferred basis sets were 6-311G(2d,p) and 6-311G(df,p) due to the higher sensitivity of sulfur to basis set choice.
2. We also saw that M08-HX did not offer much improvement over M06-2X and in some cases was detrimental. The best functionals for geometry optimization were found to be B3PW91, ωB97XD and M06-2X.
3. In the conjugated system CH_2_CHXXH, the C–X bond length decreases due to more delocalized electron density.
4. In mixed chalcogen species, the location of the chalcogen atom has a significant effect on the X–Y bond length. The RSe–S^−^ bond is slightly longer than the RS–Se^−^ bond since sulfur takes on more negative charge. Deprotonation of polysulfide species increases the C–S bond as well, for the same reason: charge delocalization along a sulfur chain.
5. In the polysulfide species PA, GPB, BDE, and HA all decrease with the increasing sulfur chain. The lower PA and GPB values indicate that in a biological system, polysulfide chains are more likely to exist as deprotonated rather than neutral species compared to the parent thiol or selenol.
6. To perform reliable thermochemical calculations, the ωB97XD/6-311G(2d,p) level of theory was found to give the most accurate results relative to the benchmark and this was then used to evaluate the geometry and thermochemistry of the more complex cysteine and cysteine per/polysulfide species.

## Figures and Tables

**Table 1:** Selected optimized bond lengths (in ångstroms; Å) for CH_3_XXH and CH_3_XX^−^ (X = S, Se).

		CH_3_SSH			CH_3_SS^−^		CH_3_SeSeH			CH_3_SeSe^−^	
Method	Basis Set	C–S	S–S	S–H	C–S	S–S	C–Se	Se-Se	*S*e–H	C–Se	Se-Se
**B3LYP**	6-31G(d)	1.834	2.091	1.357	1.837	2.117	1.977	2.337	1.495	1.985	2.366
	6-311G(d)	1.830	2.103	1.357	1.831	2.134	1.979	2.362	1.488	1.987	2.391
	6-311G(d,p)	1.831	2.105	1.354	1.833	2.134	1.978	2.364	1.478	1.987	2.391
	6-311+G(d,p)	1.830	2.103	1.354	1.832	2.128	1.978	2.364	1.478	1.987	2.388
	6-311G(2d,p)	1.830	2.088	1.349	1.831	2.115	1.975	2.366	1.476	1.984	2.395
	6-311G(df,p)	1.829	2.093	1.354	1.831	2.120	1.973	2.351	1.479	1.981	2.376
	6-311+G(2df,p)	1.825	2.072	1.351	1.827	2.092	1.971	2.350	1.477	1.979	2.373
	6-311++G(3df,3pd)	1.820	2.064	1.349	1.822	2.083	1.971	2.351	1.476	1.978	2.373
**B3PW91**	6-31G(d)	1.821	2.072	1.354	1.824	2.096	1.961	2.315	1.491	1.960	2.336
	6-311G(d)	1.817	2.081	1.356	1.819	2.110	1.963	2.338	1.486	1.963	2.361
	6-311G(d,p)	1.818	2.083	1.353	1.820	2.110	1.962	2.340	1.476	1.962	2.360
	6-311+G(d,p)	1.818	2.083	1.353	1.821	2.105	1.962	2.339	1.476	1.962	2.358
	6-311G(2d,p)	1.817	2.067	1.349	1.819	2.091	1.959	2.342	1.474	1.959	2.363
	6-311G(df,p)	1.816	2.072	1.353	1.818	2.097	1.957	2.327	1.477	1.958	2.345
	6-311+G(2df,p)	1.812	2.053	1.351	1.814	2.071	1.955	2.327	1.475	1.955	2.342
	6-311++G(3df,3pd)	1.808	2.046	1.349	1.810	2.063	1.955	2.327	1.475	1.955	2.342
**ωB97XD**	6-31G(d)	1.819	2.070	1.349	1.821	2.095	1.954	2.309	1.486	1.968	2.342
	6-311G(d)	1.816	2.079	1.350	1.816	2.110	1.957	2.331	1.480	1.971	2.365
	6-311G(d,p)	1.816	2.081	1.348	1.817	2.110	1.956	2.333	1.470	1.970	2.365
	6-311+G(d,p)	1.816	2.080	1.348	1.817	2.105	1.956	2.333	1.470	1.970	2.362
	6-311G(2d,p)	1.816	2.067	1.344	1.816	2.092	1.953	2.334	1.469	1.967	2.369
	6-311G(df,p)	1.815	2.070	1.348	1.815	2.099	1.951	2.320	1.472	1.965	2.351
	6-311+G(2df,p)	1.810	2.053	1.346	1.811	2.074	1.950	2.319	1.470	1.963	2.348
	6-311++G(3df,3pd)	1.806	2.046	1.344	1.807	2.066	1.949	2.320	1.469	1.962	2.347
**M06-2X**	6-31G(d)	1.819	2.069	1.348	1.819	2.096	1.954	2.306	1.486	1.985	2.366
	6-311G(d)	1.816	2.078	1.348	1.816	2.110	1.960	2.330	1.480	1.964	2.358
	6-311G(d,p)	1.817	2.079	1.346	1.817	2.110	1.959	2.331	1.472	1.964	2.358
	6-311+G(d,p)	1.816	2.089	1.346	1.816	2.105	1.959	2.331	1.472	1.964	2.355
	6-311G(2d,p)	1.817	2.066	1.342	1.816	2.093	1.956	2.334	1.471	1.961	2.362
	6-311G(df,p)	1.814	2.068	1.347	1.816	2.097	1.954	2.320	1.473	1.960	2.345
	6-311+G(2df,p)	1.811	2.052	1.345	1.812	2.073	1.953	2.319	1.472	1.958	2.340
	6-311++G(3df,3pd)	1.808	2.046	1.342	1.808	2.065	1.953	2.319	1.472	1.958	2.341
**M08-HX**	6-31G(d)	1.817	2.068	1.350	1.817	2.094	1.955	2.305	1.488	1.958	2.330
	6-311G(d)	1.816	2.078	1.345	1.815	2.108	1.961	2.327	1.482	1.964	2.352
	6-311G(d,p)	1.816	2.079	1.348	1.815	2.108	1.960	2.328	1.474	1.963	2.352
	6-311+G(d,p)	1.816	2.079	1.349	1.815	2.103	1.960	2.328	1.474	1.964	2.348
	6-311G(2d,p)	1.814	2.067	1.344	1.814	2.093	1.957	2.331	1.473	1.960	2.357
	6-311G(df,p)	1.814	2.069	1.349	1.814	2.095	1.954	2.318	1.475	1.959	2.341
	6-311+G(2df,p)	1.809	2.053	1.347	1.810	2.072	1.953	2.317	1.474	1.957	2.335
	6-311++G(3df,3pd)	1.807	2.047	1.344	1.808	2.063	1.953	2.318	1.472	1.957	2.336
**QCISD**	6-311+G(2df,p)	1.815	2.065	1.347	1.816	2.088	1.965	2.333	1.474	1.961	2.359

**Table 2:** Selected optimized bond lengths (in ångstroms; Å) for CH_2_CHXXH and CH_2_CHXX^−^ (X = S, Se).

		CH_2_CHSSH		CH_2_CHSS^−^		CH_2_CHSeSeH		CH_2_CHSeSe^−^	
Method	Basis Set	C–S	S–S	C–S	S–S	C–Se	Se–Se	C–Se	Se–Se
**B3PW91**	6-311+G(d,p)	1.768	2.088	1.745	2.098	1.913	2.347	1.899	2.359
	6-311G(2d,p)	1.767	2.074	1.747	2.084	1.910	2.350	1.898	2.364
	6-311G(df,p)	1.768	2.077	1.748	2.090	1.908	2.334	1.894	2.346
**ωB97XD**	6-311+G(d,p)	1.771	2.084	1.748	2.101	1.913	2.338	1.898	2.357
	6-311G(2d,p)	1.770	2.072	1.750 ^a^	2.088 ^a^	1.910	2.339	1.897	2.361
	6-311G(df,p)	1.770	2.074	1.751	2.096	1.908	2.325	1.895	2.343
**M06-2X**	6-311+G(d,p)	1.770	2.084	1.747	2.102	1.914	2.337	1.901	2.354
	6-311G(2d,p)	1.770	2.071	1.751	2.090	1.911	2.340	1.900	2.359
	6-311G(df,p)	1.770	2.072	1.750	2.095	1.909	2.326	1.897	2.343
**QCISD**	6-311+G(2df,p)	1.771	2.069	1.750	2.086	1.914	2.329	1.902	2.359

^a^ Had one negligible imaginary frequency.

**Table 3:** Selected optimized bond lengths (in ångstroms; Å) for CH_3_XYH and CH_3_XY^−^ (X = S, Se; Y = Se, S).

		CH_3_SSeH		CH_3_SSe^−^		CH_3_SeSH		CH_3_SeS^−^	
Method	Basis Set	C–S	S–Se	C–S	S–Se	C–Se	Se-S	C–Se	Se-S
**B3PW91**	6-311+G(d,p)	1.821	2.214	1.820	2.256	1.959	2.218	1.971	2.221
	6-311G(2d,p)	1.819	2.204	1.819	2.244	1.957	2.208	1.968	2.220
	6-311G(df,p)	1.819	2.199	1.819	2.241	1.953	2.204	1.964	2.212
**ωB97XD**	6-311+G(d,p)	1.820	2.208	1.817	2.252	1.953	2.213	1.962	2.218
	6-311G(2d,p)	1.818	2.199	1.817	2.242	1.951	2.204	1.959	2.216
	6-311G(df,p)	1.818	2.195	1.817	2.239	1.948	2.200	1.957	2.209
**M06-2X**	6-311+G(d,p)	1.819	2.208	1.817	2.250	1.956	2.212	1.964	2.220
	6-311G(2d,p)	1.817	2.200	1.816	2.239	1.954	2.203	1.960	2.221
	6-311G(df,p)	1.817	2.194	1.816	2.237	1.951	2.198	1.959	2.212
**QCISD**	6-311+G(2df,p)	1.819	2.198	1.816	2.239	1.952	2.202	1.961	2.207

**Table 4:** Selected optimized bond lengths (in ångstroms; Å) for RSSSH (R = CH_3_, CH_2_CH).

		CH_3_SSSH		CH_3_SSS^−^		CH_2_CHSSSH		CH_2_CHSSS^−^	
Method	Basis Set	S_1_–S_2_	S_2_–S_3_	S_1_–S_2_	S_2_–S_3_	S_1_–S_2_	S_2_–S_3_	S_1_–S_2_	S_2_–S_3_
**B3PW91**	6-311+G(d,p)	2.069	2.102	2.147	2.067	2.082	2.094	2.170	2.052
	6-311G(2d,p)	2.057	2.086	2.120	2.058	2.071	2.079	2.136	2.047
	6-311G(df,p)	2.060	2.091	2.130	2.063	2.073	2.084	2.149	2.050
**ωB97XD**	6-311+G(d,p)	2.068	2.093	2.124	2.073	2.078	2.086	2.134	2.062
	6-311G(2d,p)	2.057	2.080	2.105	2.063	2.070	2.074	2.115	2.054
	6-311G(df,p)	2.059	2.084	2.111	2.070	2.071	2.077	2.123	2.060
**M06-2X**	6-311+G(d,p)	2.066	2.090	2.121	2.074	2.079	2.083	2.143	2.063
	6-311G(2d,p)	2.056	2.078	2.103	2.065	2.070	2.071	2.118	2.055
	6-311G(df,p)	2.057	2.080	2.107	2.069	2.070	2.073	2.123	2.060
**QCISD**	6-311+G(2df,p)	2.056	2.078	2.096	2.065	2.066	2.073	2.107	2.057

**Table 5:** Homolytic S−S bond dissociation enthalpy (BDE) of RSSH, proton affinity (PA) and gas-phase basicity (GPB) of RSS^−^, and hydrogen affinity (HA) of RSS^•^ (R = CH_3_, CH_2_CH). All energies calculated at 298.15K and in kJ mol^−1^ (see Section 3).

		**CH_3_SSH**	**CH_3_SS^−^**		**CH_3_SS^•^**
**Method**	**Basis Set**	**BDE(S—S)**	**PA**	**GPB**	**HA**
**B3PW91**	6-31G(d)	235.4	1455.2	1424.8	274.6
	6-311G(2d,p)	250.3	1447.7	1417.3	283.8
	6-311+G(2df,p)	254.7	1445.8	1415.4	279.5
	6-311++G(3df,3pd)	255.8	1448.7	1418.4	281.6
**ωB97XD**	6-31G(d)	244.1	1455.0	1425.1	284.0
	6-311G(2d,p)	258.5	1447.3	1417.5	292.0
	6-311+G(2df,p)	264.6	1445.5	1415.8	288.0
	6-311++G(3df,3pd)	265.6	1449.2	1419.6	289.8
**M06-2X**	6-31G(d)	252.0	1440.0	1411.4	285.4
	6-311G(2d,p)	264.9	1430.0	1401.6	294.4
	6-311+G(2df,p)	270.3	1428.5	1400.1	291.0
	6-311++G(3df,3pd)	272.3	1432.3	1404.2	293.9
**QCISD**	6-311+G(2df,p)	**236.3**	**1445.4**	**1415.1**	**282.7**
		**CH_2_CHSSH**	**CH_2_CHSS^−^**		**CH_2_CHSS^•^**
		**BDE(S—S)**	**PA**	**GPB**	**HA**
**B3PW91**	6-31G(d)	238.0	1427.6	1396.5	272.8
	6-311G(2d,p)	253.0	1423.1	1391.5	282.1
	6-311+G(2df,p)	255.7	1417.7	1386.8	278.4
	6-311++G(3df,3pd)	256.8	1420.5	1389.7	280.1
**ωB97XD**	6-31G(d)	244.1	1429.8	1397.3	283.7
	6-311G(2d,p)	258.5	1422.4 ^a^	1395.9 ^a^	291.5
	6-311+G(2df,p)	262.7	1420.2	1382.0	288.3
	6-311++G(3df,3pd)	263.5	1423.6	1388.7	289.7
**M06-2X**	6-31G(d)	249.8	1414.5	1381.1	283.7
	6-311G(2d,p)	263.0	1408.2	1374.4	292.8
	6-311+G(2df,p)	267.4	1403.4	1366.4	290.2
	6-311++G(3df,3pd)	269.1	1406.7	1368.1	292.6
**QCISD**	6-311+G(2df,p)	**242.0**	**1421.8**	**1390.7**	**283.5**

^a^ One of the required species for determining this value had one negligible imaginary frequency.

**Table 6:** Calculated homolytic Se−Se bond dissociation enthalpies (BDE) of RSeSeH, proton affinity (PA) and gas-phase basicities (GPB) of RSeSe^−^, and Se−H homolytic bond dissociation enthalpies (HA) of RSeSe^•^ (R = CH_3_, CH_2_CH). All energies in kJ mol^−1^.

		**CH_3_SeSeH**	**CH_3_SeSe^−^**		**CH_3_SeSe^•^**
**Method**	**Basis Set**	**BDE(Se—Se)**	**PA**	**GPB**	**HA**
**B3PW91**	6-311+G(d,p)	219.2	1409.0	1378.7	270.5
	6-311+G(2df,p)	222.9	1411.4	1381.1	266.0
**ωB97XD**	6-311+G(d,p)	218.0	1410.8	1380.7	276.5
	6-311+G(2df,p)	222.6	1413.3	1383.4	270.7
**QCISD**	6-311+G(2df,p)	**204.1**	**1404.3**	**1374.0**	**267.3**
		**CH_2_CHSeSeH**	**CH_2_CHSeSe^−^**		**CH_2_CHSeSe^•^**
		**BDE(Se—Se)**	**PA**	**GPB**	**HA**
**B3PW91**	6-311+G(d,p)	222.1	1388.6	1357.4	270.2
	6-311+G(2df,p)	225.3	1390.7	1359.7	265.4
**ωB97XD**	6-311+G(d,p)	221.3	1391.4	1359.6	277.1
	6-311+G(2df,p)	225.3	1393.8	1361.1	271.0
**QCISD**	6-311+G(2df,p)	**208.3**	**1386.7**	**1355.7**	**265.9**
		**CH_3_SSeH**	**CH_3_SSe^−^**		**CH_3_SSe^•^**
		**BDE(S—Se)**	**PA**	**GPB**	**HA**
**B3PW91**	6-311+G(d,p)	221.7	1413.2	1382.8	273.8
	6-311+G(2df,p)	233.3	1415.7	1385.5	265.3
**ωB97XD**	6-311+G(d,p)	219.6	1414.0	1384.2	279.9
	6-311+G(2df,p)	232.5	1417.2	1387.5	270.9
**QCISD**	6-311+G(2df,p)	**218.7**	**1411.3**	**1381.1**	**267.5**
		**CH_3_SeSH**	**CH_3_SeS^−^**		**CH_3_SeS^•^**
		**BDE(Se—S)**	**PA**	**GPB**	**HA**
**B3PW91**	6-311+G(d,p)	229.4	1435.0	1404.8	287.1
	6-311+G(2df,p)	241.6	1441.7	1411.4	283.6
**ωB97XD**	6-311+G(d,p)	231.4	1435.6	1405.8	295.6
	6-311+G(2df,p)	245.0	1442.2	1412.4	290.8
**QCISD**	6-311+G(2df,p)	**224.5**	**1438.9**	**1408.6**	**286.1**

**Table 7:** Homolytic RS−SH bond dissociation enthalpy (BDE) of RSSSH, proton affinity (PA) and gas-phase basicity (GPB) of RSSS^−^, and hydrogen affinity (HA) of RSSS^•^ (R = CH_3_, CH_2_CH). All energies calculated at 298.15K and in kJ mol^−1^ (see Section 3).

		**CH_3_SSSH**	**CH_3_SSS^−^**		**CH_3_SSS^•^**
**Method**	**Basis Set**	**BDE(RSS—SH)**	**PA**	**GPB**	**HA**
**B3PW91**	6-311+G(2df,p)	193.8	1413.9	1382.4	287.9
**ωB97XD**	6-311+G(2df,p)	200.4	1415.0	1385.1	297.7
**QCISD**	6-311+G(2df,p)	**183.0**	**1413.9**	**1384.1**	**289.4**
		**CH_2_CHSSSH**	**CH_2_CHSSS^−^**		**CH_2_CHSSS^•^**
		**BDE(RSS—SH)**	**PA**	**GPB**	**HA**
**B3PW91**	6-311+G(2df,p)	192.8	1399.5	1367.0	290.5
**ωB97XD**	6-311+G(2df,p)	201.0	1403.0	1370.9	302.0
**QCISD**	6-311+G(2df,p)	**185.7**	**1403.7**	**1371.2**	**295.0**

**Table 8:** Selected optimized bond lengths in ångström (Å) obtained at the ωB97XD/6-311G(2d,p) level of theory for _Cys_SS*_n_*H (*n* = 0–3) and _Cys_SS*_n_*^−^ (*n* = 0–3).

Molecule	C–S_1_	S_1_–S_2_	S_2_–S_3_	S_3_–S_4_
**_Cys_SH**	1.827			
**_Cys_S–SH**	1.822	2.073		
**_Cys_S–S–SH**	1.825	2.056	2.084	
**_Cys_S–S–S–SH**	1.827	2.060	2.080	2.084
**_Cys_S^−^**	1.832			
**_Cys_S–S^−^**	1.820	2.098		
**_Cys_S–S–S^−^**	1.829	2.091	2.064	
**_Cys_S–S–S–S^−^**	1.833	2.088	2.080	2.061

## Supplementary Materials

Tables of selected optimized bond lengths obtained (i) at the MP2 level of theory for CH_3_XXH and CH_3_XX^−^ (X = S, Se); and using several DFT functionals for (ii) CH_2_CHSSH and CH_2_CHSS^−^, and CH_2_CHSeSeH and CH_2_CHSeSe^−^; (iii) CH_3_XYH and CH_3_XY^−^ (X = S, Se; Y = Se, S); (iv) RSSSH/**^−^** (R = CH_3_, CH_2_ = CH). Table summarizing Mulliken charges on each sulfur atom in RSSSH/**^−^** (R = CH_3_, CH_2_CH) as obtained at several selected levels of theory. Tables of homolytic S−S bond dissociation enthalpy (BDE) of RXYH, proton affinity (PA) and gas-phase basicity (GPB) of RXY^−^, and hydrogen affinity (HA) of RXY^•^ (R = CH_3_, CH_2_CH; X = S, Se / Y = S, Se). Table of Homolytic S−S bond dissociation enthalpy (BDE) of RSSSH, proton affinity (PA) and gas-phase basicity (GPB) of RSSS^−^, and hydrogen affinity (HA) of RSSS^•^ (R = CH_3_, CH_2_CH). Gaussian archive entry for each molecule considered in this present study.
