# Supplementary material for: An Assessment of Computational Methods for Calculating Accurate Structures and Energies of Bio-Relevant Polysulfur/Selenium-Containing Compounds
Source: Molecules. 2018 Dec 14;23(12):3323. doi: 10.3390/molecules23123323 (PMC6320821; doi:10.3390/molecules23123323)
Supplement: Supplementary file 1 [file molecules-23-03323-s001.pdf]

Supporting Information

An Assessment of Computational Methods for the Calculation of Accurate Structures and Energies of Bio-Relevant Polysulfur/Selenium-Containing Compounds

Sahar Nikoo,[§] Paul Meister,[§] John J. Hayward, James W. Gauld*

Department of Chemistry and Biochemistry, University of Windsor, Windsor, Ontario, N9B 3P4, Canada.

§ These authors contributed equally.

Corresponding Author

* Email: gauld@uwindsor.ca. Phone: +1 253-3000 Ext. 3992

Supporting Information

(**Tables S1-S9, Optimization archives**; total pages: 270)

Table S1. Selected optimized bond lengths in ångström (Å) for CH_3XXH and CH_3XX^- (X=S,Se) using MP2.

		CH_3SSH			CH_3SS^-		CH_3SeSeH			CH_3SeSe^-	
**Method**	**Basis set**	C–S	S–S	S–H	C–S	S–S	C–Se	Se-Se	*Se*–H	C–Se	Se-Se
**MP2**	6-31G(d)	1.812	2.062	1.346	1.812	2.089	1.962	2.328	1.489	1.965	2.353
	6-311G(d)	1.808	2.073	1.346	1.807	2.104	1.961	2.348	1.483	1.965	2.374
	6-311G(d,p)	1.805	2.073	1.338	1.804	2.103	1.957	2.348	1.464	1.961	2.374
	6-311+G(d,p)	1.805	2.074	1.339	1.805	2.098	1.957	2.348	1.464	1.962	2.371
	6-311G(2d,p)	1.818	2.082	1.337	1.819	2.107	1.952	2.350	1.464	1.958	2.380
	6-31G11(df,p)	1.799	2.044	1.341	1.798	2.067	1.947	2.321	1.470	1.952	2.343
	6-311+G(2df,p)	1.809	2.053	1.345	1.810	2.069	1.948	2.319	1.469	1.954	2.340
	6-311++G(3df,3pd)	1.802	2.042	1.338	1.802	2.059	1.946	2.322	1.465	1.952	2.341

Table S2a. C–S and S–S bond lengths (ångström, Å) for CH_2CHSSH and CH_2CHSS^-.

		CH_2CHSSH		CH_2CHSS^-	
**Method**	**Basis set**	C–S	S–S	C–S	S–S⁻
**B3LYP**	6-31G(d)	1.781	2.099	1.764	2.111
	6-311G(d)	1.779	2.111	1.762	2.131
	6-311+G(d)	1.778	2.109	1.756	2.124
	6-311+G(d,p)	1.778	2.110	1.755	2.123
	6-311G(2d,p)	1.777	2.096	1.759	2.109
	6-311+G(df,p)	1.776	2.096	1.753	2.109
	6-311+G(2df,p)	1.771	2.078	1.747	2.081
	6-311++G(3df,3pd)	1.767	2.069	1.742	2.076
**B3PW91**	6-31G(d)	1.772	2.078	1.753	2.088
	6-311G(d)	1.769	2.088	1.751	2.105
	6-311+G(d)	1.768	2.086	1.745	2.098
	6-311+G(d,p)	1.768	2.088	1.745	2.098
	6-311G(2d,p)	1.767	2.074	1.747	2.084
	6-311+G(df,p)	1.766	2.075	1.743	2.084
	6-311+G(2df,p)	1.762	2.058	1.737	2.063
	6-311++G(3df,3pd)	1.758	2.050	1.733	2.054
**ωB97XD**	6-31G(d)	1.766	2.075	1.755	2.090
	6-311G(d)	1.762	2.058	1.753	2.108
	6-311+G(d)	1.758	2.050	1.748	2.102
	6-311+G(d,p)	1.766	2.075	1.748	2.101
	6-311G(2d,p)	1.762	2.058	1.750	2.088
	6-311+G(df,p)	1.769	2.072	1.746	2.088
	6-311+G(2df,p)	1.764	2.057	1.740	2.069
	6-311++G(3df,3pd)	1.760	2.049	1.736	2.061
**M06-2X**	6-31G(d)	1.773	2.075	1.754	2.092
	6-311G(d)	1.772	2.083	1.752	2.108
	6-311+G(d)	1.770	2.083	1.747	2.102
	6-311+G(d,p)	1.770	2.084	1.747	2.102
	6-311G(2d,p)	1.770	2.071	1.751	2.090
	6-311+G(df,p)	1.768	2.071	1.744	2.086
	6-311+G(2df,p)	1.764	2.056	1.739	2.069
	6-311++G(3df,3pd)	1.761	2.050	1.735	2.061
**M08-HX**	6-31G(d)	1.773	2.073	1.755	2.091
	6-311G(d)	1.771	2.082	1.753	2.106
	6-311+G(d)	1.770	2.080	1.748	2.100
	6-311+G(d,p)	1.770	2.081	1.747	2.100
	6-311G(2d,p)	1.769	2.071	1.750	2.090
	6-311+G(df,p)	1.768	2.070	1.745	2.085
	6-311+G(2df,p)	1.763	2.055	1.739	2.068
	6-311++G(3df,3pd)	1.805	2.041	1.736	2.060
**QCISD**	6-311+G(2df,p)	1.771	2.069	1.750	2.086

Table S2b. C–Se and Se–Se bond lengths (ångström, Å) for $CH_2CHSeSeH$ and $CH_2CHSeSe^-$.

		$CH_2CHSeSeH$		$CH_2CHSeSe^-$	
**Method**	**Basis set**	C–Se	Se–Se	C–Se	$Se–Se^-$
**B3LYP**	6-31G(d)	1.921	2.347	1.908	2.362
	6-311G(d)	1.927	2.372	1.915	2.388
	6-311G(d,p)	1.926	2.373	1.914	2.388
	6-311+G(d,p)	1.926	2.373	1.912	2.386
	6-311G(2d,p)	1.922	2.375	1.911	2.391
	6-311G(df,p)	1.921	2.360	1.908	2.373
	6-311+G(2df,p)	1.919	2.358	1.905	2.371
	6-311++G(3df,3pd)	1.918	2.359	1.905	2.371
**B3PW91**	6-31G(d)	1.908	2.324	1.894	2.337
	6-311G(d)	1.914	2.347	1.902	2.360
	6-311G(d,p)	1.913	2.347	1.901	2.360
	6-311+G(d,p)	1.913	2.347	1.899	2.359
	6-311G(2d,p)	1.910	2.350	1.898	2.364
	6-311G(df,p)	1.908	2.334	1.894	2.346
	6-311+G(2df,p)	1.906	2.333	1.892	2.344
	6-311++G(3df,3pd)	1.906	2.331	1.892	2.344
**ωB97XD**	6-31G(d)	1.908	2.310	1.892	2.333
	6-311G(d)	1.914	2.337	1.901	2.358
	6-311G(d,p)	1.913	2.338	1.900	2.358
	6-311+G(d,p)	1.913	2.338	1.898	2.357
	6-311G(2d,p)	1.910	2.339	1.897	2.361
	6-311G(df,p)	1.908	2.325	1.895	2.343
	6-311+G(2df,p)	1.906	2.324	1.893	2.341
	6-311++G(3df,3pd)	1.906	2.325	1.893	2.341
**M06-2X**	6-31G(d)	1.906	2.313	1.893	2.331
	6-311G(d)	1.914	2.336	1.903	2.356
	6-311G(d,p)	1.914	2.337	1.902	2.356
	6-311+G(d,p)	1.914	2.337	1.901	2.354
	6-311G(2d,p)	1.911	2.340	1.900	2.359
	6-311G(df,p)	1.909	2.326	1.897	2.343
	6-311+G(2df,p)	1.907	2.325	1.895	2.340
	6-311++G(3df,3pd)	1.907	2.326	1.895	2.341
**M08-HX**	6-31G(d)	1.910	2.306	1.892	2.326
	6-311G(d)	1.915	2.332	1.901	2.349
	6-311G(d,p)	1.915	2.333	1.901	2.349
	6-311+G(d,p)	1.914	2.333	1.904	2.372
	6-311G(2d,p)	1.911	2.335	1.897	2.353
	6-311G(df,p)	1.910	2.320	1.896	2.338
	6-311+G(2df,p)	1.908	2.321	1.893	2.334
	6-311++G(3df,3pd)	1.907	2.321	1.893	2.335
**QCISD**	6-311+G(2df,p)	1.914	2.329	1.902	2.359

Table S3. C–Se, C–S and S–Se bond lengths (ångström, Å) for CH_3XYH and CH_3XY^- (X=S,Se; Y=Se,S).

		CH_3SSeH		CH_3SSe^-		CH_3SeSH		CH_3SeS^-	
**Method**	**Basis set**	C–S	S–Se	C–S	S–Se	C–Se	Se-S	C–Se	Se-S
**B3LYP**	6-31G(d)	1.837	2.219	1.836	2.266	1.973	2.226	1.985	2.234
	6-311G(d)	1.833	2.235	1.832	2.286	1.976	2.240	1.986	2.253
	6-311G(d,p)	1.834	2.237	1.833	2.286	1.975	2.242	1.986	2.253
	6-311+G(d,p)	1.834	2.237	1.833	2.281	1.975	2.241	1.988	2.244
	6-311G(2d,p)	1.832	2.227	1.832	2.270	1.973	2.231	1.983	2.244
	6-311G(df,p)	1.832	2.221	1.832	2.266	1.969	2.226	1.980	2.236
	6-311+G(2df,p)	1.826	2.213	1.826	2.250	1.968	2.216	1.981	2.221
	6-311++G(3df,3pd)	1.822	2.212	1.822	2.248	1.968	2.215	1.980	2.219
**B3PW91**	6-31G(d)	1.824	2.199	1.823	2.242	1.957	2.205	1.969	2.212
	6-311G(d)	1.820	2.212	1.820	2.260	1.960	2.217	1.970	2.228
	6-311G(d,p)	1.821	2.214	1.820	2.260	1.959	2.219	1.970	2.228
	6-311+G(d,p)	1.821	2.214	1.820	2.256	1.959	2.218	1.971	2.221
	6-311G(2d,p)	1.819	2.204	1.819	2.244	1.957	2.208	1.968	2.220
	6-311G(df,p)	1.819	2.199	1.819	2.241	1.953	2.204	1.964	2.212
	6-311+G(2df,p)	1.814	2.191	1.813	2.226	1.953	2.195	1.965	2.198
	6-311++G(3df,3pd)	1.810	2.190	1.809	2.224	1.953	2.194	1.964	2.196
**ωB97XD**	6-31G(d)	1.823	2.194	1.821	2.238	1.951	2.201	1.960	2.209
	6-311G(d)	1.820	2.207	1.818	2.256	1.947	2.190	1.962	2.224
	6-311G(d,p)	1.820	2.209	1.818	2.256	1.953	2.213	1.961	2.224
	6-311+G(d,p)	1.820	2.208	1.817	2.252	1.953	2.213	1.962	2.218
	6-311G(2d,p)	1.818	2.199	1.817	2.242	1.951	2.204	1.959	2.216
	6-311G(df,p)	1.818	2.195	1.817	2.239	1.948	2.200	1.957	2.209
	6-311+G(2df,p)	1.813	2.187	1.811	2.224	1.947	2.191	1.956	2.196
	6-311++G(3df,3pd)	1.809	2.186	1.807	2.223	1.947	2.190	1.956	2.195
**M06-2X**	6-31G(d)	1.822	2.193	1.820	2.236	1.950	2.199	1.958	2.210
	6-311G(d)	1.819	2.207	1.817	2.254	1.957	2.211	1.963	2.228
	6-311G(d,p)	1.819	2.208	1.818	2.254	1.956	2.212	1.963	2.228
	6-311+G(d,p)	1.819	2.208	1.817	2.250	1.956	2.212	1.964	2.220
	6-311G(2d,p)	1.817	2.200	1.816	2.239	1.954	2.203	1.960	2.221
	6-311G(df,p)	1.817	2.194	1.816	2.237	1.951	2.198	1.959	2.212
	6-311+G(2df,p)	1.812	2.188	1.811	2.221	1.950	2.190	1.959	2.198
	6-311++G(3df,3pd)	1.809	2.187	1.808	2.221	1.950	2.190	1.958	2.197
**M08-HX**	6-31G(d)	1.820	2.194	1.818	2.227	1.951	2.198	1.957	2.218
	6-311G(d)	1.818	2.207	1.816	2.245	1.957	2.209	1.962	2.232
	6-311G(d,p)	1.819	2.208	1.816	2.245	1.956	2.210	1.962	2.232
	6-311+G(d,p)	1.818	2.208	1.816	2.240	1.956	2.210	1.964	2.224
	6-311G(2d,p)	1.816	2.200	1.814	2.229	1.954	2.201	1.959	2.225
	6-311G(df,p)	1.817	2.195	1.815	2.228	1.951	2.197	1.958	2.217
	6-311+G(2df,p)	1.811	2.188	1.809	2.213	1.950	2.190	1.958	2.203
	6-311++G(3df,3pd)	1.809	2.187	1.807	2.212	1.951	2.189	1.958	2.201
**QCISD**	6-311+G(2df,p)	1.819	2.198	1.816	2.239	1.952	2.202	1.961	2.207

Table S4. Optimized S–S bond lengths (ångström, Å) for RSSSH/⁻ (R=CH_3, CH_2=CH).

Method	Basis set	CH_3SSSH		CH_3SSS^-		$CH_2CHSSSH$		CH_2CHSSS^-	
		S_1–S_2	S_2–S_3	S_1–S_2	S_2–S_3	S_1–S_2	S_2–S_3	S_1–S_2	S_2–S_3
**B3LYP**	6-31G(d)	2.079	2.113	2.176	2.073	2.095	2.105	2.206	2.059
	6-311G(d)	2.089	2.123	2.183	2.088	2.108	2.114	2.213	2.071
	6-311+G(d)	2.088	2.122	2.176	2.085	2.104	2.113	2.206	2.069
	6-311+G(d,p)	2.088	2.124	2.176	2.085	2.103	2.115	2.206	2.068
	6-311G(2d,p)	2.075	2.108	2.147	2.077	2.091	2.101	2.169	2.064
	6-311+G(df,p)	2.076	2.112	2.152	2.077	2.089	2.104	2.179	2.062
	6-311+G(2df,p)	2.060	2.094	2.122	2.061	2.075	2.086	2.147	2.047
	6-311++G(3df,3pd)	2.052	2.087	2.110	2.053	2.066	2.080	2.135	2.040
**B3PW91**	6-31G(d)	2.062	2.092	2.145	2.057	2.076	2.085	2.169	2.044
	6-311G(d)	2.070	2.100	2.151	2.070	2.086	2.091	2.174	2.055
	6-311+G(d)	2.070	2.099	2.147	2.067	2.083	2.091	2.171	2.052
	6-311+G(d,p)	2.069	2.102	2.147	2.067	2.082	2.094	2.170	2.052
	6-311G(2d,p)	2.057	2.086	2.120	2.058	2.071	2.079	2.136	2.047
	6-311+G(df,p)	2.058	2.090	2.126	2.059	2.070	2.084	2.145	2.046
	6-311+G(2df,p)	2.044	2.073	2.097	2.043	2.056	2.066	2.117	2.032
	6-311++G(3df,3pd)	2.036	2.067	2.087	2.036	2.048	2.061	2.105	2.025
**ωB97XD**	6-31G(d)	2.061	2.084	2.122	2.063	2.073	2.077	2.136	2.053
	6-311G(d)	2.069	2.091	2.127	2.077	2.084	2.084	2.139	2.067
	6-311+G(d)	2.069	2.091	2.124	2.074	2.080	2.084	2.137	2.063
	6-311+G(d,p)	2.068	2.093	2.124	2.073	2.078	2.086	2.134	2.062
	6-311G(2d,p)	2.057	2.080	2.105	2.063	2.070	2.074	2.115	2.054
	6-311+G(df,p)	2.058	2.083	2.108	2.065	2.067	2.077	2.118	2.055
	6-311+G(2df,p)	2.044	2.068	2.087	2.048	2.054	2.062	2.098	2.039
	6-311++G(3df,3pd)	2.037	2.062	2.078	2.042	2.047	2.056	2.089	2.032
**M06-2X**	6-31G(d)	2.060	2.082	2.121	2.063	2.072	2.076	2.144	2.053
	6-311G(d)	2.067	2.088	2.124	2.078	2.083	2.081	2.144	2.067
	6-311+G(d)	2.067	2.088	2.121	2.074	2.080	2.082	2.143	2.063
	6-311+G(d,p)	2.066	2.090	2.121	2.074	2.079	2.083	2.143	2.063
	6-311G(2d,p)	2.056	2.078	2.103	2.065	2.070	2.071	2.118	2.055
	6-311+G(df,p)	2.055	2.079	2.104	2.065	2.067	2.074	2.119	2.055
	6-311+G(2df,p)	2.043	2.065	2.085	2.049	2.056	2.059	2.100	2.039
	6-311++G(3df,3pd)	2.036	2.060	2.077	2.042	2.048	2.055	2.092	2.032
**M08-HX**	6-31G(d)	2.063	2.080	2.124	2.063	2.081	2.071	2.144	2.053
	6-311G(d)	2.071	2.088	2.128	2.076	2.089	2.078	2.148	2.065
	6-311+G(d)	2.070	2.087	2.126	2.073	2.085	2.079	2.148	2.061
	6-311+G(d,p)	2.070	2.089	2.126	2.073	2.084	2.081	2.149	2.061
	6-311G(2d,p)	2.060	2.077	2.110	2.064	2.075	2.069	2.125	2.055
	6-311+G(df,p)	2.059	2.079	2.110	2.064	2.072	2.071	2.130	2.053
	6-311+G(2df,p)	2.046	2.064	2.090	2.049	2.060	2.057	2.108	2.039
	6-311++G(3df,3pd)	2.041	2.059	2.083	2.041	2.053	2.053	2.101	2.032
**QCISD**	6-311+G(2df,p)	2.056	2.078	2.096	2.065	2.107	2.057	2.066	2.073

Table S5. Mulliken charges on every S of RSSSH/⁻ (R=CH_3, CH_2=CH).

Method/Basis Set	CH3SSSH			CH_3SSS^-		
	S_1	S_2	S_3	S_1	S_2	S_3
QCISD/6-311+G(2df,p)	0.15	0.05	-0.25	-0.05	-0.10	-0.77
	$CH_2CHSSSH$			CH_2CHSSS^-		
QCISD/6-311+G(2df,p)	-0.01	0.04	-0.20	-0.23	-0.08	-0.74
	CYS-SSSH			$CYS\text{-}SSS^-$		
ωB9XD/6-311G(2d,p)	0.00	-0.03	-0.12	-0.12	-0.13	-0.66

Table S6. Homolytic S−S bond dissociation enthalpy (BDE) of RSSH, proton affinity (PA) and gas-phase basicity (GPB) of RSS^-, and hydrogen affinity (HA) of $RSS^•$ (R=CH_3, CH_2CH). All energies calculated at 298.15K and in kJ mol^{-1}.

		CH_3SSH	CH_3SS^-		$CH_3SS^•$	CH_2CHSSH	CH_2CHSS^-		$CH_2CHSS^•$
**Methods**	**Basis set**	BDE(S—S)	PA	GPB	HA	BDE(S—S)	PA	GPB	HA
**B3LYP**	6-31G(d)	224.8	1456.4	1426.1	276.4	228.1	1430.0	1398.6	274.7
	6-311G(2d,p)	235.6	1440.5	1410.1	285.6	239.2	1418.0	1386.0	259.6
	6-311G+(2df,p)	240.3	1437.7	1407.3	281.1	241.9	1411.5	1380.2	280.2
	6-311G++(3df,3pd)	241.6	1440.9	1410.6	283.5	243.2	1414.7	1383.5	282.1
**B3PW91**	6-31G(d)	235.4	1455.2	1424.8	274.6	238.0	1427.6	1396.5	272.8
	6-311G(2d,p)	250.3	1447.7	1417.3	283.8	253.0	1423.1	1391.5	254.5
	6-311G+(2df,p)	254.7	1445.8	1415.4	279.5	255.7	1417.7	1386.8	278.4
	6-311G++(3df,3pd)	255.8	1448.7	1418.4	281.6	256.8	1420.5	1389.7	280.1
**WB97XD**	6-31G(d)	244.1	1455.0	1425.1	284.0	244.1	1429.8	1397.3	283.7
	6-311G(2d,p)	258.5	1447.3	1417.5	292.0	258.5	1422.4	1395.5	291.5
	6-311G+(2df,p)	264.6	1445.5	1415.8	288.0	262.7	1420.2	1382.0	288.3
	6-311G++(3df,3pd)	265.6	1449.2	1419.6	289.8	263.5	1423.6	1388.6	289.7
**M062X**	6-31G(d)	252.0	1440.0	1411.4	285.4	249.8	1414.5	1381.1	283.7
	6-311G(2d,p)	264.9	1430.0	1401.6	294.4	263.0	1408.2	1374.4	292.8
	6-311G+(2df,p)	270.3	1428.5	1400.1	291.0	267.4	1403.4	1366.4	290.2
	6-311G++(3df,3pd)	272.3	1432.3	1404.2	293.9	269.1	1406.7	1368.1	292.6
**M08HX**	6-31G(d)	258.0	1446.1	1414.2	295.4	255.5	1419.6	1387.9	291.6
	6-311G(2d,p)	268.3	1435.5	1405.6	298.2	268.7	1414.3	1382.0	297.1
	6-311G+(2df,p)	273.6	1433.5	1404.3	294.0	274.0	1411.4	1376.4	296.2
	6-311G++(3df,3pd)	275.2	1437.7	1408.6	296.4	272.7	1412.9	1383.4	295.7
**QCISD**	6-311G+(2df,p)	236.3	1445.4	1415.1	282.7	242.0	1421.8	1390.7	283.5

Table S7. Homolytic S−S bond dissociation enthalpy (BDE) of RSeSeH, proton affinity (PA) and gas-phase basicity (GPB) of $RSeSe^-$, and hydrogen affinity (HA) of $RSeSe^•$ (R=CH_3, CH_2CH). All energies calculated at 298.15K and in kJ mol^{-1}.

		CH_3SeSeH	CH_3SeSe^-		$CH_3SeSe^•$	$CH_2CHSeSeH$	$CH_2CHSeSe^-$		CH_2CH $SeSe^•$
**Methods**	**Basis set**	BDE(Se—Se)	PA	GPB	HA	BDE(Se—Se)	PA	GPB	HA
**B3LYP**	6-311+G(d,p)	208.0	1401.6	1371.3	272.2	211.5	1382.6	1350.9	272.0
	6-311G(df,p)	212.9	1421.0	1390.7	267.0	193.2	1400.0	1369.1	266.2
	6-311G+(2df,p)	212.0	1404.0	1373.7	267.7	214.9	1384.8	1353.2	267.1
	6-311G++(3df,3pd)	212.7	1404.3	1374.1	268.5	215.4	1385.5	1353.9	268.0
**B3PW91**	6-311+G(d,p)	219.2	1409.0	1378.7	270.5	222.1	1388.6	1357.4	270.2
	6-311G(df,p)	223.7	1424.2	1393.8	265.3	204.7	1401.9	1371.0	264.5
	6-311G+(2df,p)	222.9	1411.4	1381.1	266.0	225.3	1390.7	1359.7	265.4
	6-311G++(3df,3pd)	223.6	1411.8	1381.5	266.7	226.6	1392.1	1360.7	266.7
**WB97XD**	6-311+G(d,p)	218.0	1410.8	1380.7	276.5	221.3	1391.4	1359.6	277.1
	6-311G(df,p)	223.7	1426.5	1396.4	270.3	226.2	1405.4	1373.9	270.5
	6-311G+(2df,p)	222.6	1413.3	1383.4	270.7	225.3	1393.8	1361.1	271.0
	6-311G++(3df,3pd)	223.7	1413.6	1383.7	271.4	226.3	1391.9	1366.1	271.8
**M062X**	6-311+G(d,p)	203.5	1384.2	1353.6	275.3	206.2	1367.6	1335.5	277.3
	6-311G(df,p)	209.0	1397.8	1367.0	271.6	212.0	1377.0	1352.2	273.0
	6-311G+(2df,p)	207.7	1386.0	1355.3	272.2	211.0	1369.0	1336.1	273.6
	6-311G++(3df,3pd)	223.4	1387.4	1356.8	273.4	212.4	1370.5	1337.8	272.3
**M08HX**	6-311+G(d,p)	233.7	1380.0	1349.7	291.4	235.9	*	*	291.8
	6-311G(df,p)	237.6	1393.8	1363.4	288.1	240.7	1373.8	1339.7	288.5
	6-311G+(2df,p)	223.4	1381.9	1351.6	288.8	224.9	1362.6	1329.0	288.5
	6-311G++(3df,3pd)	223.4	1383.4	1353.2	289.5	225.7	1362.8	1337.3	290.0
**QCISD**	6-311G+(2df,p)	204.1	1404.3	1374.0	267.3	208.3	1386.7	1355.7	265.9

* Poor optimization of anion led to high energy conformation.

Table S8. Homolytic S−S bond dissociation enthalpy (BDE) of RXYH, proton affinity (PA) and gas-phase basicity (GPB) of RXY^{-}, and hydrogen affinity (HA) of $RXY^{\bullet}$ (R=CH_3, CH_2CH; X=S,Se, Y=Se,S). All energies calculated at 298.15K and in kJ mol^{-1}.

		**CH_3SSeH**	**CH_3SSe^-**		**$CH_3SSe^\bullet$**	**CH_3SeSH**	**CH_3SeS^-**		**$CH_3SeS^\bullet$**
**Methods**	**Basis set**	BDE(S—Se)	PA	GPB	HA	BDE(Se—S)	PA	GPB	HA
**B3LYP**	6-311+G(d,p)	209.8	1405.0	1374.8	275.2	217.3	1427.8	1397.6	289.2
	6-311G(df,p)	215.2	1421.2	1391.0	269.8	223.4	1435.1	1404.8	283.5
	6-311G+(2df,p)	220.7	1407.8	1377.7	267.0	229.0	1434.1	1403.9	285.3
	6-311G++(3df,3pd)	220.7	1408.0	1377.9	267.6	229.1	1437.2	1407.0	288.6
**B3PW91**	6-311+G(d,p)	221.7	1413.2	1382.8	273.8	229.4	1435.0	1404.8	287.1
	6-311G(df,p)	227.1	1425.8	1395.7	268.2	235.2	1440.5	1410.1	281.7
	6-311G+(2df,p)	233.3	1415.7	1385.5	265.3	241.6	1441.7	1411.4	283.6
	6-311G++(3df,3pd)	233.2	1415.9	1385.8	265.9	241.5	1444.5	1414.3	286.5
**WB97XD**	6-311+G(d,p)	219.6	1414.0	1384.2	279.9	231.4	1435.6	1405.8	295.6
	6-311G(df,p)	226.1	1427.3	1397.5	273.9	238.5	1440.7	1410.9	289.3
	6-311G+(2df,p)	232.5	1417.2	1387.5	270.9	245.0	1442.2	1412.4	290.8
	6-311G++(3df,3pd)	232.3	1417.6	1388.0	271.6	244.9	1445.2	1415.7	293.6
**M062X**	6-311+G(d,p)	222.3	1390.1	1359.8	277.7	226.8	1415.5	1384.6	297.8
	6-311G(df,p)	229.2	1400.9	1370.7	268.1	234.5	1420.3	1389.4	294.9
	6-311G+(2df,p)	234.3	1392.1	1362.0	270.5	240.0	1422.3	1391.7	296.3
	6-311G++(3df,3pd)	235.0	1393.2	1363.2	271.9	254.9	1425.7	1395.1	299.5
**M08HX**	6-311+G(d,p)	231.7	1386.7	1357.9	293.8	242.0	1420.6	1390.1	309.5
	6-311G(df,p)	236.8	1397.4	1368.2	288.3	246.5	1426.9	1396.1	306.6
	6-311G+(2df,p)	228.4	1387.9	1359.1	285.2	203.1	1382.7	1260.1	263.3
	6-311G++(3df,3pd)	227.9	1388.9	1359.7	285.8	259.9	1431.8	1401.2	311.8
**QCISD**	6-311G+(2df,p)	218.7	1411.3	1381.1	267.5	224.5	1438.9	1408.6	286.1

Table S9. Homolytic S−S bond dissociation enthalpy (BDE) of RSSSH, proton affinity (PA) and gas-phase basicity (GPB) of $RSSS^-$, and hydrogen affinity (HA) of $RSSS^•$ (R=CH_3, CH_2CH). All energies calculated at 298.15K and in kJ mol^{-1}.

		CH_3SSSH	CH_3SSS^-		$CH_3SSS^•$	$CH_2CHSSSH$	CH_2CHSSS^-		$CH_2CHSSS^•$
**Methods**	**Basis set**	BDE(SS—S)	PA	GPB	HA	BDE(SS—S)	PA	GPB	HA
**B3LYP**	6-31G(d)	167.6	1415.2	1383.7	283.9	166.9	1402.9	1369.7	288.1
	6-311G(2d,p)	178.4	1407.1	1375.6	292.8	177.7	1384.9	1351.4	297.2
	6-311G+(2df,p)	180.4	1406.7	1375.2	289.5	179.7	1392.7	1360.0	292.9
	6-311G++(3df,3pd)	180.1	1410.1	1378.7	291.9	179.0	1396.1	1363.4	294.9
**B3PW91**	6-31G(d)	177.9	1413.9	1382.6	282.6	176.8	1427.5	1394.2	287.1
	6-311G(2d,p)	191.5	1413.9	1382.3	290.9	190.5	1401.8	1369.3	293.9
	6-311G+(2df,p)	193.8	1413.9	1382.4	287.9	192.8	1399.5	1367.0	290.5
	6-311G++(3df,3pd)	195.9	1419.4	1387.9	292.3	192.0	1402.4	1370.1	292.2
**WB97XD**	6-31G(d)	184.2	1415.6	1385.2	293.1	185.0	1406.0	1376.3	297.8
	6-311G(2d,p)	197.3	1415.1	1385.0	300.8	198.0	1405.6	1375.1	305.5
	6-311G+(2df,p)	200.4	1415.0	1385.1	297.7	201.0	1403.0	1370.9	302.0
	6-311G++(3df,3pd)	199.6	1418.7	1388.8	299.7	199.4	1406.3	1375.3	303.8
**M062X**	6-31G(d)	192.6	1398.7	1369.0	292.5	192.7	1389.5	1357.5	297.3
	6-311G(2d,p)	204.7	1395.6	1365.4	300.9	205.2	1387.3	1356.3	306.5
	6-311G+(2df,p)	208.6	1395.7	1365.3	298.4	208.7	1385.4	1356.1	303.2
	6-311G++(3df,3pd)	209.1	1399.2	1368.7	300.9	208.6	1388.6	1358.7	305.7
**M08HX**	6-31G(d)	196.0	1402.8	1376.5	300.8	195.7	1393.8	1362.2	305.8
	6-311G(2d,p)	206.8	1400.7	1371.2	304.5	206.9	1392.9	1361.8	310.1
	6-311G+(2df,p)	209.6	1400.6	1371.4	301.3	209.4	1389.8	1359.3	306.1
	6-311G++(3df,3pd)	209.8	1404.8	1376.2	303.7	208.8	1393.8	1363.8	308.3
**QCISD**	6-311G+(2df,p)	183.0	1413.9	1384.1	289.4	185.7	1403.7	1371.2	295.0

Gaussian Archives

CH_3SH B3LYP

1\1\GINC-ORC175\FOpt\RB3LYP\6-31G(d)\C1H4S1\SNIKOO\03-Jun-2017\0\\# op
t b3lyp/6-31g(d)\\CH3-SH\\0,1\S,-0.0000340067,-0.0316151797,0.10893486
09\H,-0.0026348188,-1.0731944227,-0.7507611157\C,-0.0000295108,1.29872
03598,-1.1543384739\H,-0.8966838647,1.259893795,-1.7776960893\H,0.0020
066216,2.24272181,-0.6038848984\H,0.8947625211,1.2574975276,-1.7802131
637\Version=EM64L-G16RevA.03\State=1-A'\HF=-438.698343\RMSD=3.206e-09\
RMSF=2.166e-04\Dipole=-0.0006831,0.1802845,-0.6578706\Quadrupole=-1.41
58433,1.7363798,-0.3205365,0.0048567,0.0021488,0.4560875\PG=CS [SG(C1H
2S1),X(H2)]\\@

1\1\GINC-ORC155\FOpt\RB3LYP\6-311G(d)\C1H4S1\SNIKOO\03-Jun-2017\0\\# o
pt b3lyp/6-311g(d)\\CH3-SH\\0,1\S,-0.0000316966,-0.0298037799,0.108857
817\H,-0.0026353685,-1.0723132816,-0.7519931174\C,-0.0000309877,1.2980
748533,-1.1547748858\H,-0.8946109092,1.2603693261,-1.7756994239\H,0.00
19994393,2.239718179,-0.606138588\H,0.8926964634,1.2579785932,-1.77821
06819\Version=EM64L-G16RevA.03\State=1-A'\HF=-438.7337105\RMSD=6.561e-
09\RMSF=2.266e-04\Dipole=-0.0007004,0.1740036,-0.6642237\Quadrupole=-1
.5229632,1.8204105,-0.2974474,0.0052098,0.0024245,0.5254523\PG=CS [SG(
C1H2S1),X(H2)]\\@

1\1\GINC-ORC175\FOpt\RB3LYP\6-311+G(d)\C1H4S1\SNIKOO\03-Jun-2017\0\\#
opt b3lyp/6-311+g(d)\\CH3-SH\\0,1\S,-0.666580638,-0.0884656231,0.\H,-0
.905532019,1.2420052942,0.\C,1.1638158112,0.0198216621,0.\H,1.53481586
36,0.5212082403,0.8933394314\H,1.5298461186,-1.0064498137,0.\H,1.53481
58636,0.5212082403,-0.8933394314\\Version=EM64L-G16RevA.03\State=1-A'\
HF=-438.734426\RMSD=3.329e-09\RMSF=5.080e-05\Dipole=0.5594698,0.394005
1,0.\Quadrupole=0.3948196,1.0915004,-1.48632,-1.1161083,0.,0.\PG=CS [S
G(C1H2S1),X(H2)]\\@

1\1\GINC-ORC41\FOpt\RB3LYP\6-311+G(d,p)\C1H4S1\SNIKOO\18-Jul-2017\0\\#
opt freq b3lyp/6-311+g(d,p)\\CH3-SH\\0,1\S,0.0458419114,-0.6698137192
,0.\H,-1.29272126,-0.8326330546,0.\C,0.0484644,1.164430449,0.\H,-0.430
6889901,1.5621191368,0.8941428014\H,1.0956639288,1.4666560512,0.\H,-0.
4306889901,1.5621191368,-0.8941428014\\Version=EM64L-G09RevD.01\State=
1-A'\HF=-438.7432184\RMSD=1.922e-09\RMSF=1.674e-04\Dipole=-0.3468154,0
.5729855,0.\Quadrupole=1.1921595,0.2615631,-1.4537225,1.0472705,0.,0.\
PG=CS [SG(C1H2S1),X(H2)]\\@

1\1\GINC-ORC110\FOpt\RB3LYP\6-311G(2d,p)\C1H4S1\SNIKOO\26-Jul-2017\0\\
opt freq b3lyp/6-311g(2d,p)\\CH3-SH\\0,1\S,0.0440144172,-0.667206184
4,0.\H,-1.2896163171,-0.8309865221,0.\C,0.0476791338,1.1655524805,0.\H
,-0.4305731085,1.5605230763,0.8941720479\H,1.0949399832,1.4644720735,0
.\H,-0.4305731085,1.5605230763,-0.8941720479\\Version=EM64L-G09RevD.01
\State=1-A'\HF=-438.7463674\RMSD=3.863e-09\RMSF=2.642e-04\Dipole=-0.30
52033,0.566623,0.\Quadrupole=1.1880871,0.3647153,-1.5528024,0.9776251,
0.,0.\PG=CS [SG(C1H2S1),X(H2)]\\@

1\1\GINC-ORC34\FOpt\RB3LYP\6-311+G(df,p)\C1H4S1\SNIKOO\18-Jul-2017\0\\
opt freq b3lyp/6-311+g(df,p)\\CH3-SH\\0,1\S,0.0454779087,-0.66869146
59,0.\H,-1.2926507882,-0.8336077074,0.\C,0.0486636234,1.1641461063,0.\
H,-0.4306773931,1.5624121175,0.8936164488\H,1.0957350422,1.466206832,0
.\H,-0.4306773931,1.5624121175,-0.8936164488\\Version=EM64L-G09RevD.01
\State=1-A'\HF=-438.7452777\RMSD=2.423e-09\RMSF=8.602e-05\Dipole=-0.34

75511,0.5748207,0.\Quadrupole=1.1589211,0.232764,-1.3916851,1.0438589,
0.,0.\PG=CS [SG(C1H2S1),X(H2)]\\@

1\1\GINC-ORC33\FOpt\RB3LYP\6-311+G(2df,p)\C1H4S1\SNIKOO\18-Jul-2017\0\
\# opt freq b3lyp/6-311+g(2df,p)\\CH3-SH\\0,1\S,0.0449220905,-0.664106
8793,0.\H,-1.2897632289,-0.8287296992,0.\C,0.0481193638,1.1638148261,0
.\H,-0.4312186918,1.5588984362,0.8937314195\H,1.0950301581,1.464102879
9,0.\H,-0.4312186918,1.5588984362,-0.8937314195\\Version=EM64L-G09RevD
.01\State=1-A'\HF=-438.7495649\RMSD=9.708e-09\RMSF=6.340e-05\Dipole=-0
.30496,0.5570565,0.\Quadrupole=1.1829423,0.2756874,-1.4586296,0.962651
,0.,0.\PG=CS [SG(C1H2S1),X(H2)]\\@

1\1\GINC-ORC1\FOpt\RB3LYP\6-311++G(3df,3pd)\C1H4S1\SNIKOO\26-Jul-2017\
0\\# opt freq b3lyp/6-311++g(3df,3pd)\\CH3-SH\\0,1\S,0.0436178996,-0.6
615677616,0.\H,-1.2882173082,-0.8276174307,0.\C,0.0482197772,1.1635503
941,0.\H,-0.4306540266,1.5574723588,0.8923277214\H,1.0935586847,1.4635
680807,0.\H,-0.4306540266,1.5574723588,-0.8923277214\\Version=EM64L-G0
9RevD.01\State=1-A'\HF=-438.7536221\RMSD=3.611e-09\RMSF=1.380e-04\Dipo
le=-0.2605787,0.5504566,0.\Quadrupole=1.1061449,0.2770222,-1.3831671,0
.8789122,0.,0.\PG=CS [SG(C1H2S1),X(H2)]\\@

CH_3SH B3PW91

1\1\GINC-ORC187\FOpt\RB3PW91\6-31G(d)\C1H4S1\SNIKOO\20-Jul-2017\0\\# o
pt freq b3pw91/6-31g(d) geom=connectivity\\CH3-SH\\0,1\S,-0.6602981287
,-0.0868032304,0.\H,-0.9018911874,1.2390356951,0.\C,1.1601770159,0.020
259877,0.\H,1.5325992692,0.5227166933,0.8958710366\H,1.5279947617,-1.0
085977284,0.\H,1.5325992692,0.5227166933,-0.8958710366\\Version=EM64L-
G09RevD.01\State=1-A'\HF=-438.6337986\RMSD=8.319e-09\RMSF=1.259e-04\Di
pole=0.5708784,0.400393,0.\Quadrupole=0.4252201,1.0026214,-1.4278415,-
1.1237894,0.,0.\PG=CS [SG(C1H2S1),X(H2)]\\@

1\1\GINC-ORC179\FOpt\RB3PW91\6-311G(d)\C1H4S1\SNIKOO\20-Jul-2017\0\\#
opt freq b3pw91/6-311g(d) geom=connectivity\\CH3-SH\\0,1\S,-0.65860596
61,-0.0887290552,0.\H,-0.9007556652,1.2402794002,0.\C,1.1600364019,0.0
209596919,0.\H,1.5316099914,0.5210498279,0.8940561652\H,1.5272862465,-
1.0052816926,0.\H,1.5316099914,0.5210498279,-0.8940561652\\Version=EM6
4L-G09RevD.01\State=1-A'\HF=-438.6665606\RMSD=8.150e-09\RMSF=1.693e-04
\Dipole=0.5644309,0.408849,0.\Quadrupole=0.4134348,1.1060751,-1.519509
9,-1.1575341,0.,0.\PG=CS [SG(C1H2S1),X(H2)]\\@

1\1\GINC-ORC165\FOpt\RB3PW91\6-311+G(d)\C1H4S1\SNIKOO\20-Jul-2017\0\\#
opt freq b3pw91/6-311+g(d) geom=connectivity\\CH3-SH\\0,1\S,-0.658699
5637,-0.0887232252,0.\H,-0.8997477136,1.2404008387,0.\C,1.1594074865,0
.020357381,0.\H,1.5310819953,0.5213986846,0.8938254428\H,1.5280568003,
-1.0055043637,0.\H,1.5310819953,0.5213986846,-0.8938254428\\Version=EM
64L-G09RevD.01\State=1-A'\HF=-438.6673912\RMSD=1.850e-09\RMSF=1.738e-0
4\Dipole=0.5627267,0.407622,0.\Quadrupole=0.3897286,1.0931956,-1.48292
42,-1.1438638,0.,0.\PG=CS [SG(C1H2S1),X(H2)]\\@

1\1\GINC-ORC163\FOpt\RB3PW91\6-311+G(d,p)\C1H4S1\SNIKOO\20-Jul-2017\0\
\# opt freq b3pw91/6-311+g(d,p) geom=connectivity\\CH3-SH\\0,1\S,-0.65
81147538,-0.0868701754,0.\H,-0.9002709236,1.2384260621,0.\C,1.16145447
44,0.0206199761,0.\H,1.5308118298,0.5219746037,0.8947377573\H,1.526488
5433,-1.0067970702,0.\H,1.5308118298,0.5219746037,-0.8947377573\\Versi
on=EM64L-G09RevD.01\State=1-A'\HF=-438.6761783\RMSD=3.094e-09\RMSF=1.4
15e-04\Dipole=0.5545834,0.391988,0.\Quadrupole=0.3820518,1.0649142,-1.

4469659,-1.114669,0.,0.\PG=CS [SG(C1H2S1),X(H2)]\\@

1\1\GINC-ORC14\FOpt\RB3PW91\6-311G(2d,p)\C1H4S1\SNIKOO\26-Jul-2017\0\\
opt freq b3pw91/6-311g(2d,p)\\CH3-SH\\0,1\S,-0.6555146864,-0.0850503
681,0.\H,-0.8986576252,1.2355995028,0.\C,1.1630236712,0.0216852239,0.\
H,1.5291314922,0.5217510274,0.8951749104\H,1.5240666559,-1.0064084134,
0.\H,1.5291314922,0.5217510274,-0.8951749104\\Version=EM64L-G09RevD.01
\State=1-A'\HF=-438.679566\RMSD=1.406e-09\RMSF=1.258e-04\Dipole=0.5493
72,0.3469904,0.\Quadrupole=0.4760711,1.0728101,-1.5488812,-1.0343751,0
.,0.\PG=CS [SG(C1H2S1),X(H2)]\\@

1\1\GINC-ORC159\FOpt\RB3PW91\6-311+G(df,p)\C1H4S1\SNIKOO\20-Jul-2017\0
\\# opt freq b3pw91/6-311+g(df,p) geom=connectivity\\CH3-SH\\0,1\S,-0.
6567744745,-0.0864599133,0.\H,-0.9016056978,1.2381454434,0.\C,1.160916
4705,0.020510377,0.\H,1.5315106577,0.5219867446,0.894056652\H,1.525623
3864,-1.0068413963,0.\H,1.5315106577,0.5219867446,-0.894056652\\Versio
n=EM64L-G09RevD.01\State=1-A'\HF=-438.6781958\RMSD=4.419e-09\RMSF=1.70
7e-04\Dipole=0.556523,0.392865,0.\Quadrupole=0.3524194,1.0296499,-1.38
20693,-1.1103665,0.,0.\PG=CS [SG(C1H2S1),X(H2)]\\@

1\1\GINC-ORC157\FOpt\RB3PW91\6-311+G(2df,p)\C1H4S1\SNIKOO\20-Jul-2017\
0\\# opt freq b3pw91/6-311+g(2df,p) geom=connectivity\\CH3-SH\\0,1\S,-
0.6520139104,-0.0854155883,0.\H,-0.897570445,1.2358469583,0.\C,1.16074
67629,0.0211142036,0.\H,1.5281817905,0.522074789,0.8944307958\H,1.5236
550115,-1.0063671515,0.\H,1.5281817905,0.522074789,-0.8944307958\\Vers
ion=EM64L-G09RevD.01\State=1-A'\HF=-438.6828433\RMSD=4.678e-09\RMSF=2.
138e-04\Dipole=0.5407495,0.3459764,0.\Quadrupole=0.3892342,1.0641529,-
1.4533871,-1.0259116,0.,0.\PG=CS [SG(C1H2S1),X(H2)]\\@

1\1\GINC-ORC93\FOpt\RB3PW91\6-311++G(3df,3pd)\C1H4S1\SNIKOO\26-Jul-201
7\0\\# opt freq b3pw91/6-311++g(3df,3pd)\\CH3-SH\\0,1\S,-0.649541884,-
0.0845728876,0.\H,-0.8967201602,1.2342449564,0.\C,1.159152489,0.021657
5103,0.\H,1.5279481664,0.5212131752,0.8930768712\H,1.5223942224,-1.004
4279296,0.\H,1.5279481664,0.5212131752,-0.8930768712\\Version=EM64L-G1
6RevA.03\State=1-A'\HF=-438.6864487\RMSD=2.929e-09\RMSF=3.466e-05\Dipo
le=0.5368805,0.3002499,0.\Quadrupole=0.3825839,0.9974596,-1.3800436,-0
.9380666,0.,0.\PG=CS [SG(C1H2S1),X(H2)]\\@

CH_3SH wB97XD

1\1\GINC-ORC108\FOpt\RwB97XD\6-31G(d)\C1H4S1\SNIKOO\21-Jul-2017\0\\# o
pt freq rwb97xd/6-31g(d)\\CH3-SH\\0,1\S,0.0461137425,-0.666462238,0.\H
,-1.2876400793,-0.8291299036,0.\C,0.0484231138,1.1555314609,0.\H,-0.43
17670487,1.5558455001,0.894927163\H,1.0962463205,1.4630246806,0.\H,-0.
4317670487,1.5558455001,-0.894927163\\Version=EM64L-G09RevD.01\State=1
-A'\HF=-438.6641348\RMSD=7.003e-09\RMSF=8.444e-05\Dipole=-0.3711091,0.
5974268,0.\Quadrupole=1.134159,0.2862948,-1.4204538,1.0964206,0.,0.\PG
=CS [SG(C1H2S1),X(H2)]\\@

1\1\GINC-ORC96\FOpt\RwB97XD\6-311G(d)\C1H4S1\SNIKOO\21-Jul-2017\0\\# o
pt freq rwb97xd/6-311g(d)\\CH3-SH\\0,1\S,0.0480471017,-0.665454922,0.\
H,-1.2884781921,-0.8273918572,0.\C,0.0477320236,1.1551601637,0.\H,-0.4
30403806,1.5548356933,0.8933857746\H,1.0931156789,1.4626702289,0.\H,-0
.430403806,1.5548356933,-0.8933857746\\Version=EM64L-G09RevD.01\State=
1-A'\HF=-438.6968009\RMSD=8.262e-09\RMSF=3.410e-05\Dipole=-0.3817991,0
.5906841,0.\Quadrupole=1.2380153,0.2770203,-1.5150357,1.1250647,0.,0.\
PG=CS [SG(C1H2S1),X(H2)]\\@

1\1\GINC-ORC96\FOpt\RwB97XD\6-311+G(d)\C1H4S1\SNIKOO\21-Jul-2017\0\\#
opt freq rwb97xd/6-311+g(d)\\CH3-SH\\0,1\S,0.0480091,-0.6655035385,0.\
H,-1.2885030365,-0.8264787124,0.\C,0.0482210933,1.154557814,0.\H,-0.43
07175962,1.5543696628,0.8931853673\H,1.0933170357,1.4633401112,0.\H,-0
.4307175962,1.5543696628,-0.8931853673\\Version=EM64L-G09RevD.01\State
=1-A'\HF=-438.6976545\RMSD=4.045e-09\RMSF=4.450e-05\Dipole=-0.3802748,
0.5899473,0.\Quadrupole=1.2233059,0.2597802,-1.4830861,1.1105722,0.,0.
\PG=CS [SG(C1H2S1),X(H2)]\\@

1\1\GINC-ORC93\FOpt\RwB97XD\6-311+G(d,p)\C1H4S1\SNIKOO\21-Jul-2017\0\\
opt freq rwb97xd/6-311+g(d,p)\\CH3-SH\\0,1\S,0.0468600702,-0.6646445
086,0.\H,-1.2866451641,-0.8261979658,0.\C,0.0479430695,1.1559900922,0.
\H,-0.4313517988,1.5539684847,0.8938254838\H,1.0941546219,1.4615704129
,0.\H,-0.4313517988,1.5539684847,-0.8938254838\\Version=EM64L-G09RevD.
01\State=1-A'\HF=-438.7062762\RMSD=7.716e-09\RMSF=5.820e-05\Dipole=-0.
3636323,0.5804214,0.\Quadrupole=1.190516,0.2570566,-1.4475727,1.077216
1,0.,0.\PG=CS [SG(C1H2S1),X(H2)]\\@

1\1\GINC-ORC46\FOpt\RwB97XD\6-311G(2d,p)\C1H4S1\SNIKOO\26-Jul-2017\0\\
opt freq rwb97xd/6-311g(2d,p)\\CH3-SH\\0,1\S,0.0451601254,-0.6621473
714,0.\H,-1.2841251184,-0.825579564,0.\C,0.0469929496,1.1577266034,0.\
H,-0.4309677948,1.5526200637,0.8941948738\H,1.093516633,1.4594152046,0
.\H,-0.4309677948,1.5526200637,-0.8941948738\\Version=EM64L-G09RevD.01
\State=1-A'\HF=-438.7097655\RMSD=4.742e-09\RMSF=4.955e-05\Dipole=-0.32
04227,0.5704111,0.\Quadrupole=1.1897727,0.3581805,-1.5479532,1.0059892
,0.,0.\PG=CS [SG(C1H2S1),X(H2)]\\@

1\1\GINC-ORC92\FOpt\RwB97XD\6-311+G(df,p)\C1H4S1\SNIKOO\21-Jul-2017\0\
\# opt freq rwb97xd/6-311+g(df,p)\\CH3-SH\\0,1\S,0.0464189496,-0.66335
32748,0.\H,-1.286549318,-0.8274603977,0.\C,0.0481066198,1.1557303763,0
.\H,-0.4313113076,1.554378572,0.8933092007\H,1.0942553639,1.4609811522
,0.\H,-0.4313113076,1.554378572,-0.8933092007\\Version=EM64L-G09RevD.0
1\State=1-A'\HF=-438.7082826\RMSD=9.393e-09\RMSF=1.051e-04\Dipole=-0.3
643318,0.5825981,0.\Quadrupole=1.1551503,0.2258667,-1.381017,1.0732204
,0.,0.\PG=CS [SG(C1H2S1),X(H2)]\\@

1\1\GINC-ORC84\FOpt\RwB97XD\6-311+G(2df,p)\C1H4S1\SNIKOO\21-Jul-2017\0
\\# opt freq rwb97xd/6-311+g(2df,p)\\CH3-SH\\0,1\S,0.0456557691,-0.658
8296658,0.\H,-1.2843130851,-0.8248728545,0.\C,0.0473629527,1.155533992
1,0.\H,-0.431264502,1.5519428103,0.8934587143\H,1.0934323672,1.4589379
075,0.\H,-0.431264502,1.5519428103,-0.8934587143\\Version=EM64L-G09Rev
D.01\State=1-A'\HF=-438.7130501\RMSD=6.615e-09\RMSF=7.438e-05\Dipole=-
0.3201922,0.5629555,0.\Quadrupole=1.179924,0.2764855,-1.4564095,0.9930
693,0.,0.\PG=CS [SG(C1H2S1),X(H2)]\\@

1\1\GINC-ORC41\FOpt\RwB97XD\6-311++G(3df,3pd)\C1H4S1\SNIKOO\26-Jul-201
7\0\\# opt freq rwb97xd/6-311++g(3df,3pd)\\CH3-SH\\0,1\S,0.0446891606,
-0.6561949366,0.\H,-1.2830071005,-0.8242301514,0.\C,0.0471720097,1.154
9595327,0.\H,-0.4304457932,1.5509707016,0.8920394016\H,1.0916465166,1.
458179152,0.\H,-0.4304457932,1.5509707016,-0.8920394016\\Version=EM64L
-G09RevD.01\State=1-A'\HF=-438.717063\RMSD=6.936e-09\RMSF=1.139e-04\Di
pole=-0.2763582,0.557495,0.\Quadrupole=1.1126399,0.2786081,-1.391248,0
.9132534,0.,0.\PG=CS [SG(C1H2S1),X(H2)]\\@

CH_3SH M062X

1\1\GINC-ORC201\FOpt\RM062X\6-31G(d)\C1H4S1\SNIKOO\03-Jun-2017\0\\# op

t m062x/6-31g(d)\\CH3-SH\\0,1\S,-0.000034299,-0.0254739086,0.102892115
\H,-0.0026206835,-1.0577808852,-0.7553847183\C,-0.0000321118,1.2940397
172,-1.1517321932\H,-0.8953666765,1.2538369952,-1.7732801467\H,0.00199
91701,2.2379577345,-0.604660421\H,0.8934415416,1.2514442367,-1.7757935
159\\Version=EM64L-G16RevA.03\State=1-A\HF=-438.6274914\RMSD=5.412e-09
\RMSF=9.522e-05\Dipole=-0.0006968,0.1730744,-0.6607427\Quadrupole=-1.4
058407,1.7452403,-0.3393996,0.0048844,0.0021361,0.4769652\PG=C01 [X(C1
H4S1)]\\@

1\1\GINC-ORC183\FOpt\RM062X\6-311G(d)\C1H4S1\SNIKOO\03-Jun-2017\0\\# o
pt m062x/6-311g(d)\\CH3-SH\\0,1\S,-0.6586642289,-0.0861154312,0.\H,-0.
8913964333,1.237186208,0.\C,1.1591455378,0.0205158705,0.\H,1.526759638
9,0.5208673111,0.8930412825\H,1.5248528464,-1.0044662694,0.\H,1.526759
6389,0.5208673111,-0.8930412825\\Version=EM64L-G16RevA.03\State=1-A'\H
F=-438.6668835\RMSD=3.120e-09\RMSF=1.438e-04\Dipole=0.5621376,0.411462
8,0.\Quadrupole=0.3971388,1.1187521,-1.5158909,-1.1618048,0.,0.\PG=CS
[SG(C1H2S1),X(H2)]\\@

1\1\GINC-ORC183\FOpt\RM062X\6-311+G(d)\C1H4S1\SNIKOO\03-Jun-2017\0\\#
opt m062x/6-311+g(d)\\CH3-SH\\0,1\S,0.0484112974,-0.6668086546,0.\H,-1
.2865180333,-0.8199721393,0.\C,0.0480226118,1.1534559139,0.\H,-0.43044
45539,1.5504212262,0.8928962729\H,1.0925472319,1.4594954276,0.\H,-0.43
04445539,1.5504212262,-0.8928962729\\Version=EM64L-G16RevA.03\State=1-
A'\HF=-438.6677745\RMSD=6.177e-09\RMSF=7.235e-05\Dipole=-0.3759194,0.5
82889,0.\Quadrupole=1.2353999,0.2435258,-1.4789257,1.0934303,0.,0.\PG=
CS [SG(C1H2S1),X(H2)]\\@

1\1\GINC-ORC52\FOpt\RM062X\6-311G(2d,p)\C1H4S1\SNIKOO\26-Jul-2017\0\\#
opt freq m062x/6-311g(2d,p)\\CH3-SH\\0,1\S,0.0452448983,-0.6633310723
,0.\H,-1.2830979957,-0.8171316103,0.\C,0.0474181735,1.1558886357,0.\H,
-0.4308720278,1.5482068577,0.8935856953\H,1.0928429795,1.4571883316,0.
\H,-0.4308720278,1.5482068577,-0.8935856953\\Version=EM64L-G09RevD.01\
State=1-A'\HF=-438.6783409\RMSD=5.252e-09\RMSF=2.303e-04\Dipole=-0.315
9701,0.5648008,0.\Quadrupole=1.205954,0.3424549,-1.5484089,0.9855169,0
.,0.\PG=CS [SG(C1H2S1),X(H2)]\\@

1\1\GINC-ORC274\FOpt\RM062X\6-311+G(df,p)\C1H4S1\SNIKOO\18-Jul-2017\0\
\# opt freq m062x/6-311+g(df,p)\\CH3-SH\\0,1\S,0.0464527895,-0.6651544
584,0.\H,-1.2861972611,-0.820864299,0.\C,0.0483762127,1.1542618007,0.\
H,-0.4308201818,1.5509234841,0.8929215281\H,1.0936726224,1.4589379887,
0.\H,-0.4308201818,1.5509234841,-0.8929215281\\Version=EM64L-G09RevD.0
1\State=1-A'\HF=-438.6771721\RMSD=6.687e-09\RMSF=5.088e-05\Dipole=-0.3
617012,0.579742,0.\Quadrupole=1.1669863,0.2044351,-1.3714214,1.0609946
,0.,0.\PG=CS [SG(C1H2S1),X(H2)]\\@

1\1\GINC-ORC272\FOpt\RM062X\6-311+G(2df,p)\C1H4S1\SNIKOO\18-Jul-2017\0
\\# opt freq m062x/6-311+g(2df,p)\\CH3-SH\\0,1\S,0.0460110218,-0.66067
7623,0.\H,-1.2837342629,-0.8170954902,0.\C,0.0476998582,1.1543143245,0
.\H,-0.4311212394,1.5479096607,0.8931022858\H,1.0929298617,1.456667467
3,0.\H,-0.4311212394,1.5479096607,-0.8931022858\\Version=EM64L-G09RevD
.01\State=1-A'\HF=-438.6816707\RMSD=8.085e-09\RMSF=3.412e-05\Dipole=-0
.3137061,0.5572811,0.\Quadrupole=1.1883252,0.2568293,-1.4451544,0.9678
542,0.,0.\PG=CS [SG(C1H2S1),X(H2)]\\@

1\1\GINC-ORC46\FOpt\RM062X\6-311++G(3df,3pd)\C1H4S1\SNIKOO\26-Jul-2017
\0\\# opt freq m062x/6-311++g(3df,3pd)\\CH3-SH\\0,1\S,0.0449853618,-0.
6585395223,0.\H,-1.2824289298,-0.8163352388,0.\C,0.0476655271,1.154123

0858,0.\H,-0.4303458103,1.5468589065,0.8915170387\H,1.0911336615,1.456
0618623,0.\H,-0.4303458103,1.5468589065,-0.8915170387\\Version=EM64L-G
09RevD.01\State=1-A'\HF=-438.6860955\RMSD=6.030e-09\RMSF=4.848e-05\Dip
ole=-0.2687788,0.5488808,0.\Quadrupole=1.1141042,0.2673275,-1.3814317,
0.8824041,0.,0.\PG=CS [SG(C1H2S1),X(H2)]\\@

CH_3SH M08HX

1\1\GINC-ORC329\FOpt\RM08HX\6-31G(d)\C1H4S1\SNIKOO\25-Jul-2017\0\\# op
t freq 6-31g(d) m08hx\\CH3-SH\\0,1\S,0.0480965167,-0.6678263292,0.\H,-
1.2877179584,-0.8237649087,0.\C,0.0481100938,1.1516096493,0.\H,-0.4334
526696,1.5548685925,0.8975033418\H,1.0980256871,1.4648994036,0.\H,-0.4
334526696,1.5548685925,-0.8975033418\\Version=EM64L-G16RevA.03\State=1
-A'\HF=-438.6428352\RMSD=1.782e-09\RMSF=8.150e-05\Dipole=-0.3634317,0.
5732116,0.\Quadrupole=1.1441209,0.2697569,-1.4138777,1.0744892,0.,0.\P
G=CS [SG(C1H2S1),X(H2)]\\@

1\1\GINC-ORC245\FOpt\RM08HX\6-311G(d)\C1H4S1\SNIKOO\25-Jul-2017\0\\# o
pt freq 6-311g(d) m08hx\\CH3-SH\\0,1\S,0.0501093409,-0.6665838933,0.\H
,-1.2887230792,-0.8231960455,0.\C,0.0472235495,1.1529295873,0.\H,-0.43
20060157,1.5540166462,0.8960350597\H,1.0950112203,1.463472059,0.\H,-0.
4320060157,1.5540166462,-0.8960350597\\Version=EM64L-G16RevA.03\State=
1-A'\HF=-438.684735\RMSD=7.552e-09\RMSF=5.895e-05\Dipole=-0.3791819,0.
5754519,0.\Quadrupole=1.2481253,0.255841,-1.5039664,1.1148955,0.,0.\PG
=CS [SG(C1H2S1),X(H2)]\\@

1\1\GINC-ORC329\FOpt\RM08HX\6-311+G(d)\C1H4S1\SNIKOO\25-Jul-2017\0\\#
opt freq 6-311+g(d) m08hx\\CH3-SH\\0,1\S,0.0500896068,-0.6668450385,0.
\H,-1.2888987102,-0.8219418327,0.\C,0.0478793887,1.152081,0.\H,-0.4323
605787,1.553361321,0.8958494501\H,1.0952598721,1.4646382294,0.\H,-0.43
23605787,1.553361321,-0.8958494501\\Version=EM64L-G16RevA.03\State=1-A
'\HF=-438.685661\RMSD=8.800e-09\RMSF=5.796e-05\Dipole=-0.3784047,0.573
5887,0.\Quadrupole=1.238286,0.2285578,-1.4668438,1.0998389,0.,0.\PG=CS
[SG(C1H2S1),X(H2)]\\@

1\1\GINC-ORC329\FOpt\RM08HX\6-311+G(d,p)\C1H4S1\SNIKOO\25-Jul-2017\0\\
opt freq 6-311+g(d,p) m08hx\\CH3-SH\\0,1\S,0.0483007205,-0.665928063
5,0.\H,-1.2869089309,-0.8224931406,0.\C,0.0477516196,1.1539682079,0.\H
,-0.4328434566,1.5530912496,0.89640877\H,1.0961525039,1.4629254969,0.\
H,-0.4328434566,1.5530912496,-0.89640877\\Version=EM64L-G16RevA.03\Sta
te=1-A'\HF=-438.6931554\RMSD=5.208e-09\RMSF=4.766e-05\Dipole=-0.363844
1,0.5649017,0.\Quadrupole=1.2072202,0.2263464,-1.4335666,1.0722811,0.,
0.\PG=CS [SG(C1H2S1),X(H2)]\\@

1\1\GINC-ORC209\FOpt\RM08HX\6-311G(2d,p)\C1H4S1\SNIKOO\01-Aug-2017\0\\
opt freq 6-311g(2d,p) m08hx\\CH3-S(-)\\0,1\S,0.6984870937,-0.0540024
043,0.2059202573\C,-1.1092473119,0.034697643,0.0285617259\H,-1.4974604
451,-0.9282862832,0.3665874049\H,-1.5303011563,0.8208318575,0.65736695
02\H,-1.39780845,0.1825018963,-1.0129962725\H,0.9628953096,1.179402940
7,-0.2454839659\\Version=EM64L-G16RevA.03\State=1-A'\HF=-438.6964652\RM
SD=3.397e-09\RMSF=9.601e-05\Dipole=-0.5226156,0.3263698,-0.1663085\Qua
drupole=0.4920679,0.7585016,-1.2505695,1.038645,-0.2369743,-0.7980897\
PG=CS [SG(C1H2S1),X(H2)]\\@

1\1\GINC-ORC174\FOpt\RM08HX\6-311+G(df,p)\C1H4S1\SNIKOO\25-Jul-2017\0\
\# opt freq 6-311+g(df,p) m08hx\\CH3-SH\\0,1\S,0.0480168898,-0.6647111

983,0.\H,-1.2866593182,-0.823846935,0.\C,0.0477616419,1.1530692329,0.\
H,-0.4328303203,1.554027743,0.8957311749\H,1.096150427,1.4620884144,0.
\H,-0.4328303203,1.554027743,-0.8957311749\\Version=EM64L-G16RevA.03\S
tate=1-A'\HF=-438.6954206\RMSD=5.692e-09\RMSF=8.488e-05\Dipole=-0.3652
54,0.565764,0.\Quadrupole=1.1682067,0.1932425,-1.3614492,1.068205,0.,0
.\PG=CS [SG(C1H2S1),X(H2)]\\@

1\1\GINC-ORC342\FOpt\RM08HX\6-311+G(2df,p)\C1H4S1\SNIKOO\25-Jul-2017\0
\\# opt freq 6-311+g(2df,p) m08hx\\CH3-SH\\0,1\S,0.0470261174,-0.65950
01691,0.\H,-1.284486884,-0.8214947214,0.\C,0.0471948659,1.1531782477,0
.\H,-0.4327835457,1.5512476654,0.8958169501\H,1.095441992,1.459976312,
0.\H,-0.4327835457,1.5512476654,-0.8958169501\\Version=EM64L-G16RevA.0
3\State=1-A'\HF=-438.7000063\RMSD=4.948e-09\RMSF=1.279e-04\Dipole=-0.3
215809,0.5406536,0.\Quadrupole=1.1965953,0.2416477,-1.438243,0.9901306
,0.,0.\PG=CS [SG(C1H2S1),X(H2)]\\@

1\1\GINC-ORC205\FOpt\RM08HX\6-311++G(3df,3pd)\C1H4S1\ROOT\01-Aug-2017\
0\\# opt freq 6-311++g(3df,3pd) m08hx\\CH3-S(-)\\0,1\S,0.6932929995,-0
.053418349,0.2057329816\C,-1.1070250025,0.0343003342,0.0288058174\H,-1
.4962929498,-0.926426206,0.3655673598\H,-1.5288727446,0.819150423,0.65
52688339\H,-1.3963369151,0.18291114,-1.0103910249\H,0.9617996525,1.178
6283078,-0.2450278678\\Version=EM64L-G16RevA.03\State=1-A'\HF=-438.7051
332\RMSD=4.085e-09\RMSF=9.433e-05\Dipole=-0.5178714,0.2876343,-0.15132
94\Quadrupole=0.3914229,0.7219723,-1.1133953,0.952178,-0.2243682,-0.72
88093\PG=CS [SG(C1H2S1),X(H2)]\\@

CH_3SH QCISD

1\1\GINC-ORC10\FOpt\RQCISD-FC\6-311+G(2df,p)\C1H4S1\SNIKOO\19-Aug-2017
\0\\# opt freq 6-311+g(2df,p) qcisd\\CH3-SH\\0,1\S,-0.046478747,-0.228
7614911,0.6957069567\C,0.03500016,-0.0326452588,-1.1118648146\H,0.1407
913708,1.0152078233,-1.3927848638\H,0.8479131142,-0.6202982313,-1.5384
418997\H,-0.9111122428,-0.4066883361,-1.5059171009\H,1.1689661847,0.27
3186494,0.9648193923\\Version=EM64L-G09RevD.01\State=1-A'\HF=-437.74825
66\MP2=-438.0955735\MP3=-438.1287128\MP4D=-438.1382573\MP4DQ=-438.1293
036\MP4SDQ=-438.1314024\QCISD=-438.1314719\RMSD=3.644e-09\RMSF=2.706e-
05\Dipole=0.3101914,0.1793296,-0.5069048\PG=CS [SG(C1H2S1),X(H2)]\\@

CH_3S^- B3LYP

1\1\GINC-ORC174\FOpt\RB3LYP\6-31G(d)\C1H3S1(1-)\SNIKOO\03-Jun-2017\0\\
opt b3lyp/6-31g(d)\\CH3-S(-)\\-1,1\S,-0.0000000184,-0.0662867142,0.1
665013755\C,0.0000000291,1.2685720049,-1.1045944619\H,-0.8836695828,1.
2259443907,-1.7653417107\H,0.0019774535,2.2790553083,-0.6594028104\H,0
.8816922494,1.2235824953,-1.7678220216\\Version=EM64L-G16RevA.03\State
=1-A1\HF=-438.1096265\RMSD=6.948e-09\RMSF=2.505e-05\Dipole=0.,0.894265
6,-0.8515487\Quadrupole=0.4160917,-0.2385705,-0.1775211,0.,0.,0.623390
6\PG=C03V [C3(C1S1),3SGV(H1)]\\@

1\1\GINC-ORC172\FOpt\RB3LYP\6-311G(d)\C1H3S1(1-)\SNIKOO\03-Jun-2017\0\
\# opt b3lyp/6-311g(d)\\CH3-S(-)\\-1,1\S,-0.0000000183,-0.0632562647,0
.1636156831\C,0.0000000293,1.2753296745,-1.1110293344\H,-0.8833405104,
1.2228346591,-1.7621193496\H,0.001976717,2.2755534055,-0.6565922933\H,
0.8813639132,1.2204736433,-1.7645987369\\Version=EM64L-G16RevA.03\Stat
e=1-A1\HF=-438.1616368\RMSD=3.594e-09\RMSF=1.038e-04\Dipole=0.,0.98413
69,-0.9371272\Quadrupole=0.3878422,-0.2223734,-0.1654688,0.,0.,0.58106
71\PG=C03V [C3(C1S1),3SGV(H1)]\\@

1\1\GINC-ORC172\FOpt\RB3LYP\6-311+G(d)\C1H3S1(1-)\SNIKOO\03-Jun-2017\0
\\# opt b3lyp/6-311+g(d)\\CH3-S(-)\\-1,1\S,0.,0.,0.7132463728\C,0.,0.,
-1.1338995944\H,0.0000002768,1.0194383582,-1.544046043\H,0.8828593774,
-0.5097194188,-1.544046043\H,-0.8828596542,-0.5097189394,-1.544046043\
\Version=EM64L-G16RevA.03\State=1-A1\HF=-438.1636726\RMSD=2.909e-09\RM
SF=1.526e-05\Dipole=0.,0.,-1.4371416\Quadrupole=0.3791792,0.3791792,-0
.7583584,0.,0.,0.\PG=C03V [C3(C1S1),3SGV(H1)]\\@

1\1\GINC-ORC258\FOpt\RB3LYP\6-311+G(d,p)\C1H3S1(1-)\SNIKOO\18-Jul-2017
\0\\# opt freq b3lyp/6-311+g(d,p)\\CH3-S(-)\\-1,1\S,-0.0000007821,0.00
00029919,0.7132739646\C,-0.0000055843,0.0000146142,-1.1340198383\H,0.0
000108897,1.0201553211,-1.5424337054\H,0.8834056745,-0.5100788706,-1.5
424485069\H,-0.8834101978,-0.5100940566,-1.542443914\\Version=EM64L-G0
9RevD.01\State=1-A\HF=-438.168216\RMSD=7.557e-09\RMSF=1.508e-04\Dipole
=0.0001027,0.0000104,-1.4196058\Quadrupole=0.4097962,0.4100271,-0.8198
233,0.0003009,0.0000544,0.0000984\PG=C01 [X(C1H3S1)]\\@

1\1\GINC-ORC144\FOpt\RB3LYP\6-311G(2d,p)\C1H3S1(1-)\SNIKOO\26-Jul-2017
\0\\# opt freq b3lyp/6-311g(2d,p)\\CH3-S(-)\\-1,1\S,0.,0.,0.7120003619
\C,0.,0.,-1.1354914629\H,0.0000144692,1.0192566634,-1.5415284697\H,0.8
826949288,-0.5096408624,-1.5415284697\H,-0.8827093981,-0.5096158009,-1
.5415284697\\Version=EM64L-G09RevD.01\State=1-A1\HF=-438.1684681\RMSD=
6.924e-09\RMSF=6.781e-05\Dipole=0.,0.,-1.3063029\Quadrupole=0.3953355,
0.3953355,-0.7906709,0.,0.,0.\PG=C03V [C3(C1S1),3SGV(H1)]\\@

1\1\GINC-ORC205\FOpt\RB3LYP\6-311+G(df,p)\C1H3S1(1-)\ROOT\18-Jul-2017\
0\\# opt freq b3lyp/6-311+g(df,p)\\CH3-S(-)\\-1,1\S,-0.0000004462,0.00
00039055,0.7129653336\C,-0.0000031864,0.0000161041,-1.1334366543\H,0.0
00006215,1.0197736389,-1.5425234751\H,0.8830711916,-0.5098924906,-1.54
25399126\H,-0.8830737739,-0.509901158,-1.5425372916\\Version=EM64L-G09
RevD.01\State=1-A\HF=-438.1700538\RMSD=3.850e-09\RMSF=4.757e-05\Dipole
=0.0000715,-0.0000528,-1.4183173\Quadrupole=0.4418901,0.4424701,-0.884
3602,0.0002255,0.000034,0.0000432\PG=C01 [X(C1H3S1)]\\@

1\1\GINC-ORC78\FOpt\RB3LYP\6-311+G(2df,p)\C1H3S1(1-)\SNIKOO\18-Jul-201
7\0\\# opt freq b3lyp/6-311+g(2df,p)\\CH3-S(-)\\-1,1\S,-0.0000016574,0
.000001619,0.7074796312\C,-0.0000117708,0.0000116654,-1.1341759311\H,0
.0000229529,1.0195603917,-1.540453045\H,0.8828963193,-0.5097708364,-1.
5404661759\H,-0.882905844,-0.5098028397,-1.5404564792\\Version=EM64L-G
09RevD.01\State=1-A\HF=-438.172728\RMSD=2.992e-09\RMSF=5.371e-05\Dipol
e=0.0000702,0.0000655,-1.3749313\Quadrupole=0.426134,0.4260633,-0.8521
973,0.0000811,0.0000489,0.0001564\PG=C01 [X(C1H3S1)]\\@

1\1\GINC-ORC154\FOpt\RB3LYP\6-311++G(3df,3pd)\C1H3S1(1-)\SNIKOO\26-Jul
-2017\0\\# opt freq b3lyp/6-311++g(3df,3pd)\\CH3-S(-)\\-1,1\S,-0.00000
20631,0.0000039839,0.7041198203\C,-0.0000146318,0.0000086927,-1.134263
8748\H,0.0000285298,1.0182817591,-1.5393010978\H,0.8817920615,-0.50912
73295,-1.5393194525\H,-0.8818038963,-0.5091671062,-1.5393073953\\Versi
on=EM64L-G09RevD.01\State=1-A\HF=-438.1755122\RMSD=2.042e-09\RMSF=6.64
2e-05\Dipole=0.0001172,0.0000314,-1.3207446\Quadrupole=0.4858483,0.486
0724,-0.9719207,0.0001475,0.0001366,0.0001879\PG=C01 [X(C1H3S1)]\\@

CH_3S^- B3PW91

1\1\GINC-ORC248\FOpt\RB3PW91\6-31G(d)\C1H3S1(1-)\SNIKOO\20-Jul-2017\0\
\# opt freq b3pw91/6-31g(d) geom=connectivity\\CH3-S(-)\\-1,1\S,0.,0.,
0.7046679764\C,0.,0.,-1.1265900258\H,0.0000002318,1.0192607711,-1.5508

933693\H,0.882705605,-0.5096305863,-1.5508933693\H,-0.8827058368,-0.50
96301848,-1.5508933693\\Version=EM64L-G09RevD.01\State=1-A1\HF=-438.04
54645\RMSD=8.521e-09\RMSF=1.511e-04\Dipole=0.,0.,-1.258281\Quadrupole=
0.4057517,0.4057517,-0.8115034,0.,0.,0.\PG=C03V [C3(C1S1),3SGV(H1)]\\@

1\1\GINC-ORC240\FOpt\RB3PW91\6-311G(d)\C1H3S1(1-)\SNIKOO\20-Jul-2017\0
\\# opt freq b3pw91/6-311g(d) geom=connectivity\\CH3-S(-)\\-1,1\S,0.,0
.,0.7005750799\C,0.,0.,-1.1320971482\H,0.0000002317,1.0188253465,-1.54
77019202\H,0.8823285162,-0.5094128739,-1.5477019202\H,-0.8823287479,-0
.5094124726,-1.5477019202\\Version=EM64L-G09RevD.01\State=1-A1\HF=-438
.0918837\RMSD=4.452e-09\RMSF=2.582e-04\Dipole=0.,0.,-1.3658959\Quadrup
ole=0.3767501,0.3767501,-0.7535003,0.,0.,0.\PG=C03V [C3(C1S1),3SGV(H1)
]\\@

1\1\GINC-ORC238\FOpt\RB3PW91\6-311+G(d)\C1H3S1(1-)\SNIKOO\20-Jul-2017\
0\\# opt freq b3pw91/6-311+g(d) geom=connectivity\\CH3-S(-)\\-1,1\S,0.
,0.,0.6995201831\C,0.,0.,-1.1320861712\H,0.0000002318,1.0190622494,-1.
5473545783\H,0.8825336801,-0.5095313254,-1.5473545783\H,-0.8825339119,
-0.509530924,-1.5473545783\\Version=EM64L-G09RevD.01\State=1-A1\HF=-43
8.0934877\RMSD=2.858e-09\RMSF=2.259e-04\Dipole=0.,0.,-1.430511\Quadrup
ole=0.3810706,0.3810706,-0.7621412,0.,0.,0.\PG=C03V [C3(C1S1),3SGV(H1)
]\\@

1\1\GINC-ORC227\FOpt\RB3PW91\6-311+G(d,p)\C1H3S1(1-)\ROOT\20-Jul-2017\
0\\# opt freq b3pw91/6-311+g(d,p) geom=connectivity\\CH3-S(-)\\-1,1\S,
0.,0.,0.6986090441\C,0.,0.,-1.1340161191\H,0.0000002319,1.0196715632,-
1.5464108069\H,0.8830613613,-0.5098359824,-1.5464108069\H,-0.883061593
2,-0.5098355808,-1.5464108069\\Version=EM64L-G09RevD.01\State=1-A1\HF=
-438.0980473\RMSD=1.844e-09\RMSF=1.614e-04\Dipole=0.,0.,-1.4145111\Qua
drupole=0.4127518,0.4127518,-0.8255036,0.,0.,0.\PG=C03V [C3(C1S1),3SGV
(H1)]\\@

1\1\GINC-ORC14\FOpt\RB3PW91\6-311G(2d,p)\C1H3S1(1-)\SNIKOO\26-Jul-2017
\0\\# opt freq b3pw91/6-311g(2d,p)\\CH3-S(-)\\-1,1\S,0.,0.,0.697216955
9\C,0.,0.,-1.1365308991\H,0.0000002318,1.0193931384,-1.5451116803\H,0.
8828202384,-0.50969677,-1.5451116803\H,-0.8828204702,-0.5096963684,-1.
5451116803\\Version=EM64L-G09RevD.01\State=1-A1\HF=-438.0989694\RMSD=1
.262e-09\RMSF=1.820e-05\Dipole=0.,0.,-1.3120236\Quadrupole=0.3855197,0
.3855197,-0.7710395,0.,0.,0.\PG=C03V [C3(C1S1),3SGV(H1)]\\@

1\1\GINC-ORC220\FOpt\RB3PW91\6-311+G(df,p)\C1H3S1(1-)\ROOT\20-Jul-2017
\0\\# opt freq b3pw91/6-311+g(df,p) geom=connectivity\\CH3-S(-)\\-1,1\
S,0.,0.,0.6987158857\C,0.,0.,-1.1323072294\H,0.0000002318,1.0191807702
,-1.547013677\H,0.8826363221,-0.5095905858,-1.547013677\H,-0.882636553
9,-0.5095901843,-1.547013677\\Version=EM64L-G09RevD.01\State=1-A1\HF=-
438.0998722\RMSD=9.562e-09\RMSF=2.746e-04\Dipole=0.,0.,-1.4113969\Quad
rupole=0.4461605,0.4461605,-0.8923211,0.,0.,0.\PG=C03V [C3(C1S1),3SGV(
H1)]\\@

1\1\GINC-ORC205\FOpt\RB3PW91\6-311+G(2df,p)\C1H3S1(1-)\ROOT\20-Jul-201
7\0\\# opt freq b3pw91/6-311+g(2df,p) geom=connectivity\\CH3-S(-)\\-1,
1\S,0.,0.,0.6932568245\C,0.,0.,-1.1330992168\H,0.0000002318,1.01913485
87,-1.5449348548\H,0.8825965617,-0.5095676301,-1.5449348548\H,-0.88259
67934,-0.5095672286,-1.5449348548\\Version=EM64L-G09RevD.01\State=1-A1
\HF=-438.102772\RMSD=6.346e-09\RMSF=2.918e-04\Dipole=0.,0.,-1.3644788\
Quadrupole=0.4284658,0.4284658,-0.8569316,0.,0.,0.\PG=C03V [C3(C1S1),3
SGV(H1)]\\@

1\1\GINC-ORC19\FOpt\RB3PW91\6-311++G(3df,3pd)\C1H3S1(1-)\SNIKOO\26-Jul
-2017\0\\# opt freq b3pw91/6-311++g(3df,3pd)\\CH3-S(-)\\-1,1\S,0.,0.,0
.6902938176\C,0.,0.,-1.1330916397\H,0.0000002315,1.0180415024,-1.54395
11821\H,0.8816496874,-0.5090209517,-1.5439511821\H,-0.8816499189,-0.50
90205507,-1.5439511821\\Version=EM64L-G09RevD.01\State=1-A1\HF=-438.10
52444\RMSD=9.104e-09\RMSF=2.646e-04\Dipole=0.,0.,-1.3086483\Quadrupole
=0.4887454,0.4887454,-0.9774907,0.,0.,0.\PG=C03V [C3(C1S1),3SGV(H1)]\\
@

CH_3S^- wB97XD

1\1\GINC-ORC135\FOpt\RwB97XD\6-31G(d)\C1H3S1(1-)\SNIKOO\21-Jul-2017\0\
\# opt freq rwb97xd/6-31g(d)\\CH3-S(-)\\-1,1\S,0.,0.00000004,0.7087016
783\C,0.,0.00000004,-1.1220976569\H,0.0000084003,1.0186145105,-1.54458
65561\H,0.882141808,-0.5093144701,-1.5445865561\H,-0.8821502083,-0.509
2999204,-1.5445865561\\Version=EM64L-G09RevD.01\State=1-A1\HF=-438.075
7431\RMSD=5.389e-09\RMSF=1.458e-04\Dipole=0.,0.,-1.2805426\Quadrupole=
0.403139,0.403139,-0.8062779,0.,0.,0.\PG=C03V [C3(C1S1),3SGV(H1)]\\@

1\1\GINC-ORC114\FOpt\RwB97XD\6-311G(d)\C1H3S1(1-)\SNIKOO\21-Jul-2017\0
\\# opt freq rwb97xd/6-311g(d)\\CH3-S(-)\\-1,1\S,0.,0.00000004,0.70453
54709\C,0.,0.00000004,-1.1298295024\H,0.000055146,1.0187797389,-1.5406
426509\H,0.8822615271,-0.5094375673,-1.5406426509\H,-0.8823166731,-0.5
093420516,-1.5406426509\\Version=EM64L-G09RevD.01\State=1-A1\HF=-438.1
222335\RMSD=4.834e-09\RMSF=1.889e-05\Dipole=0.,0.,-1.3960479\Quadrupol
e=0.3691138,0.3691138,-0.7382276,0.,0.,0.\PG=C03V [C3(C1S1),3SGV(H1)]\
\@

1\1\GINC-ORC113\FOpt\RwB97XD\6-311+G(d)\C1H3S1(1-)\SNIKOO\21-Jul-2017\
0\\# opt freq rwb97xd/6-311+g(d)\\CH3-S(-)\\-1,1\S,0.0000868298,0.0004
680995,0.7029929603\C,-0.0002059203,-0.000181427,-1.1300087722\H,0.000
2072249,1.0191441567,-1.5393904252\H,0.8822938375,-0.5095705333,-1.540
2835739\H,-0.8823819719,-0.5098600958,-1.540406189\\Version=EM64L-G09R
evD.01\State=1-A\HF=-438.1240305\RMSD=5.590e-09\RMSF=9.835e-05\Dipole=
0.0000264,-0.000166,-1.4716731\Quadrupole=0.3461302,0.3475076,-0.69363
78,0.000764,0.0000912,0.0013786\PG=C01 [X(C1H3S1)]\\@

1\1\GINC-ORC110\FOpt\RwB97XD\6-311+G(d,p)\C1H3S1(1-)\SNIKOO\21-Jul-201
7\0\\# opt freq rwb97xd/6-311+g(d,p)\\CH3-S(-)\\-1,1\S,-0.0001690923,0
.0004265063,0.7023681775\C,0.0002612892,-0.0001690942,-1.1314665095\H,
-0.0002712031,1.0197403816,-1.5387549002\H,0.8830051524,-0.510207268,-
1.5397765124\H,-0.8828261463,-0.5097903258,-1.5394662554\\Version=EM64
L-G09RevD.01\State=1-A\HF=-438.1284073\RMSD=4.180e-09\RMSF=8.977e-05\D
ipole=0.0000141,-0.0001305,-1.4544344\Quadrupole=0.3758968,0.3770387,-
0.7529355,-0.000885,-0.0000868,0.0010691\PG=C01 [X(C1H3S1)]\\@

1\1\GINC-ORC54\FOpt\RwB97XD\6-311G(2d,p)\C1H3S1(1-)\SNIKOO\26-Jul-2017
\0\\# opt freq rwb97xd/6-311g(2d,p)\\CH3-S(-)\\-1,1\S,0.,0.00000004,0.
7015587825\C,0.,0.00000004,-1.1325025908\H,0.0000806219,1.0189100921,-
1.5387668349\H,0.8823616784,-0.5095248067,-1.5387668349\H,-0.882442300
3,-0.5093851655,-1.5387668349\\Version=EM64L-G09RevD.01\State=1-A1\HF=
-438.1292551\RMSD=6.928e-09\RMSF=3.196e-05\Dipole=0.,0.,-1.336215\Quad
rupole=0.3717432,0.3717432,-0.7434865,0.,0.,0.\PG=C03V [C3(C1S1),3SGV(
H1)]\\@

1\1\GINC-ORC109\FOpt\RwB97XD\6-311+G(df,p)\C1H3S1(1-)\SNIKOO\21-Jul-20
17\0\\# opt freq rwb97xd/6-311+g(df,p)\\CH3-S(-)\\-1,1\S,0.0001735584,
0.0004939987,0.7022594593\C,-0.0003392208,-0.0002829831,-1.1302998567\
H,0.0003166014,1.0191047859,-1.5390388658\H,0.8825278047,-0.5094317303
,-1.5398680637\H,-0.8826787437,-0.5098838712,-1.5401486731\\Version=EM
64L-G09RevD.01\State=1-A\HF=-438.130187\RMSD=3.855e-09\RMSF=9.172e-05\
Dipole=-0.0000519,-0.0001343,-1.4517048\Quadrupole=0.410949,0.4113804,
-0.8223294,0.0010739,0.0002089,0.0007585\PG=C01 [X(C1H3S1)]\\@

1\1\GINC-ORC109\FOpt\RwB97XD\6-311+G(2df,p)\C1H3S1(1-)\SNIKOO\21-Jul-2
017\0\\# opt freq rwb97xd/6-311+g(2df,p)\\CH3-S(-)\\-1,1\S,-0.00026665
5,0.0005119103,0.6969414324\C,0.0003066057,-0.0003816589,-1.1311030771
\H,-0.0002218593,1.018738943,-1.5370009197\H,0.8826418761,-0.509615193
3,-1.5382343882\H,-0.8824599675,-0.509253801,-1.5376990474\\Version=EM
64L-G09RevD.01\State=1-A\HF=-438.133175\RMSD=6.946e-09\RMSF=1.154e-04\
Dipole=0.000099,-0.0001333,-1.4026454\Quadrupole=0.3925743,0.3918074,-
0.7843818,-0.0006424,-0.0003797,0.0007962\PG=C01 [X(C1H3S1)]\\@

1\1\GINC-ORC52\FOpt\RwB97XD\6-311++G(3df,3pd)\C1H3S1(1-)\SNIKOO\26-Jul
-2017\0\\# opt freq rwb97xd/6-311++g(3df,3pd)\\CH3-S(-)\\-1,1\S,0.,0.0
0000004,0.6940056534\C,0.,0.00000004,-1.1304773643\H,0.0000962279,1.01
73307529,-1.5368917778\H,0.8809861275,-0.5087486523,-1.5368917778\H,-0
.8810823554,-0.5085819807,-1.5368917778\\Version=EM64L-G09RevD.01\Stat
e=1-A1\HF=-438.1358758\RMSD=1.753e-09\RMSF=2.297e-05\Dipole=0.,0.,-1.3
509867\Quadrupole=0.4441585,0.4441585,-0.8883171,0.,0.,0.\PG=C03V [C3(
C1S1),3SGV(H1)]\\@

CH_3S^- M062X

1\1\GINC-ORC207\FOpt\RM062X\6-31G(d)\C1H3S1(1-)\SNIKOO\03-Jun-2017\0\\
opt m062x/6-31g(d)\\CH3-S(-)\\-1,1\S,-0.0000000181,-0.0585397392,0.1
591244542\C,0.0000000291,1.2684464623,-1.1044749162\H,-0.8819948239,1.
2240771056,-1.7622344218\H,0.0019737058,2.2751921338,-0.6583915322\H,0
.880021238,1.2217196866,-1.7647100319\\Version=EM64L-G16RevA.03\State=
1-A1\HF=-438.044873\RMSD=6.724e-09\RMSF=1.004e-04\Dipole=0.,0.8958736,
-0.85308\Quadrupole=0.4107868,-0.2355289,-0.1752579,0.,0.,0.6154428\PG
=C03V [C3(C1S1),3SGV(H1)]\\@

1\1\GINC-ORC250\FOpt\RM062X\6-311G(d)\C1H3S1(1-)\SNIKOO\03-Jun-2017\0\
\# opt 6-311g(d) m062x\\CH3-S(-)\\-1,1\S,0.,0.,0.7030315978\C,0.,0.,-1
.1340642354\H,0.0000002764,1.0180982785,-1.5405890376\H,0.8816988346,-
0.5090493787,-1.5405890376\H,-0.881699111,-0.5090488999,-1.5405890376\
\Version=EM64L-G16RevA.03\State=1-A1\HF=-438.098362\RMSD=2.638e-09\RMS
F=1.853e-04\Dipole=0.,0.,-1.3725531\Quadrupole=0.3797659,0.3797659,-0.
7595319,0.,0.,0.\PG=C03V [C3(C1S1),3SGV(H1)]\\@

1\1\GINC-ORC201\FOpt\RM062X\6-311+G(d)\C1H3S1(1-)\SNIKOO\03-Jun-2017\0
\\# opt m062x/6-311+g(d)\\CH3-S(-)\\-1,1\S,0.019724202,-0.7022858311,0
.\C,0.0691354764,1.1317600756,0.\H,-0.4290036078,1.5529394337,0.881507
4535\H,1.0972877697,1.5130463746,0.\H,-0.4290036079,1.5529394337,-0.88
15074535\\Version=EM64L-G16RevA.03\State=1-A'\HF=-438.0997842\RMSD=3.4
76e-09\RMSF=4.873e-05\Dipole=0.0380846,1.4247692,0.\Quadrupole=0.37771
78,-0.7562295,0.3785116,-0.0294045,0.,0.\PG=CS [SG(C1H1S1),X(H2)]\\@

1\1\GINC-ORC307\FOpt\RM062X\6-311+G(d,p)\C1H3S1(1-)\SNIKOO\18-Jul-2017
\0\\# opt freq m062x/6-311+g(d,p)\\CH3-S(-)\\-1,1\S,0.0004224874,-0.70
91912631,0.\C,-0.0002896113,1.1262987301,0.\H,-0.5095263238,1.53266176

86,0.8820987958\H,1.0180583356,1.5331858096,0.\H,-0.5095263238,1.53266
17686,-0.8820987958\\Version=EM64L-G09RevD.01\State=1-A'\HF=-438.10301
75\RMSD=4.094e-09\RMSF=1.560e-04\Dipole=-0.0004541,1.4128218,0.\Quadru
pole=0.4031852,-0.8109541,0.4077689,0.0009842,0.,0.\PG=CS [SG(C1H1S1),
X(H2)]\\@

1\1\GINC-ORC52\FOpt\RM062X\6-311G(2d,p)\C1H3S1(1-)\SNIKOO\26-Jul-2017\
0\\# opt freq m062x/6-311g(2d,p)\\CH3-S(-)\\-1,1\S,0.0005455415,-0.707
6188435,0.\C,-0.0003935393,1.1288126266,0.\H,-0.5092634948,1.531379350
8,0.8817096447\H,1.0175304391,1.5316694529,0.\H,-0.5092634948,1.531379
3508,-0.8817096447\\Version=EM64L-G09RevD.01\State=1-A'\HF=-438.104157
4\RMSD=3.088e-09\RMSF=4.991e-05\Dipole=-0.0004643,1.3126699,0.\Quadrup
ole=0.3821872,-0.7669189,0.3847317,0.0004653,0.,0.\PG=CS [SG(C1H1S1),X
(H2)]\\@

1\1\GINC-ORC282\FOpt\RM062X\6-311+G(df,p)\C1H3S1(1-)\SNIKOO\18-Jul-201
7\0\\# opt freq m062x/6-311+g(df,p)\\CH3-S(-)\\-1,1\S,0.000044,-0.7094
58,0.\C,0.000044,1.125253,0.\H,-0.509258,1.532864,0.881507\H,1.017554,
1.53409,-0.0000000002\H,-0.5092580003,1.532864,-0.8815069998\\Version=
EM64L-G09RevD.01\State=1-A'\HF=-438.1049984\RMSD=4.460e-09\RMSF=7.686e
-05\Dipole=-0.0003237,1.409017,0.\Quadrupole=0.4441963,-0.8923112,0.44
81149,0.0018022,0.,0.\PG=CS [SG(C1H1S1),X(H2)]\\@

1\1\GINC-ORC278\FOpt\RM062X\6-311+G(2df,p)\C1H3S1(1-)\SNIKOO\18-Jul-20
17\0\\# opt freq m062x/6-311+g(2df,p)\\CH3-S(-)\\-1,1\S,0.0004176592,-
0.7041507627,0.\C,-0.0002988805,1.1263041923,0.\H,-0.5092790183,1.5309
777364,0.8817288503\H,1.0175906198,1.5315118086,0.\H,-0.5092790183,1.5
309777364,-0.8817288503\\Version=EM64L-G09RevD.01\State=1-A'\HF=-438.1
077676\RMSD=3.259e-09\RMSF=6.924e-05\Dipole=-0.0004105,1.3567366,0.\Qu
adrupole=0.4253991,-0.8554362,0.4300371,0.0010197,0.,0.\PG=CS [SG(C1H1
S1),X(H2)]\\@

1\1\GINC-ORC16\FOpt\RM062X\6-311++G(3df,3pd)\C1H3S1(1-)\SNIKOO\26-Jul-
2017\0\\# opt freq m062x/6-311++g(3df,3pd)\\CH3-S(-)\\-1,1\S,0.0003810
057,-0.7011188365,0.\C,-0.0002781232,1.1262192121,0.\H,-0.508471838,1.
5299727064,0.8803373114\H,1.0159950257,1.530575796,0.\H,-0.508471838,1
.5299727064,-0.8803373114\\Version=EM64L-G09RevD.01\State=1-A'\HF=-438
.1109714\RMSD=6.308e-09\RMSF=3.930e-05\Dipole=-0.0003745,1.3051885,0.\
Quadrupole=0.4733501,-0.9507723,0.4774222,0.0009628,0.,0.\PG=CS [SG(C1
H1S1),X(H2)]\\@

$CH3S^-$ M08HX

1\1\GINC-ORC329\FOpt\RM08HX\6-31G(d)\C1H3S1(1-)\SNIKOO\25-Jul-2017\0\\
opt freq 6-31g(d) m08hx\\CH3-S(-)\\-1,1\S,0.0000027808,-0.0002317769
,0.7097025912\C,0.000005838,0.0001077189,-1.1191944231\H,-0.0000129373
,1.0212862741,-1.5461836425\H,0.8844573607,-0.5105897275,-1.5457052999
\H,-0.8844530422,-0.5105722886,-1.5457152257\\Version=EM64L-G16RevA.03
\State=1-A\HF=-438.0589446\RMSD=8.952e-09\RMSF=7.503e-05\Dipole=-0.000
0159,-0.0000031,-1.2197894\Quadrupole=0.4141779,0.4138951,-0.8280731,-
0.0000927,-0.0000298,-0.0000653\PG=C01 [X(C1H3S1)]\\@

1\1\GINC-ORC245\FOpt\RM08HX\6-311G(d)\C1H3S1(1-)\SNIKOO\25-Jul-2017\0\
\# opt freq 6-311g(d) m08hx\\CH3-S(-)\\-1,1\S,0.,0.00000004,0.70382858
74\C,0.,0.00000004,-1.1273457778\H,0.0000487121,1.0212167653,-1.541682
5486\H,0.8843752708,-0.5106505086,-1.5416825486\H,-0.8844239829,-0.510
5661367,-1.5416825486\\Version=EM64L-G16RevA.03\State=1-A1\HF=-438.113

7449\RMSD=8.726e-09\RMSF=1.347e-04\Dipole=0.,0.,-1.3514145\Quadrupole=
0.3812657,0.3812657,-0.7625314,0.,0.,0.\PG=C03V [C3(C1S1),3SGV(H1)]\\@

1\1\GINC-ORC175\FOpt\RM08HX\6-311+G(d)\C1H3S1(1-)\SNIKOO\25-Jul-2017\0
\\# opt freq 6-311+g(d) m08hx\\CH3-S(-)\\-1,1\S,-0.0000336287,-0.00013
51829,0.7032331047\C,0.0000250718,0.000060217,-1.126438652\H,0.0000179
78,1.0216107405,-1.5414846019\H,0.8847749905,-0.5107546479,-1.54123988
99\H,-0.8847844116,-0.5107809268,-1.5411659609\\Version=EM64L-G16RevA.
03\State=1-A\HF=-438.1152508\RMSD=3.055e-09\RMSF=1.624e-04\Dipole=0.00
00925,0.0000672,-1.3935173\Quadrupole=0.4084368,0.4082318,-0.8166686,0
.0001644,-0.0000584,-0.0002236\PG=C01 [X(C1H3S1)]\\@

1\1\GINC-ORC206\FOpt\RM08HX\6-311+G(d,p)\C1H3S1(1-)\SNIKOO\25-Jul-2017
\0\\# opt freq 6-311+g(d,p) m08hx\\CH3-S(-)\\-1,1\S,0.0000257186,-0.00
0041836,0.7032349554\C,0.0000124607,0.0000277238,-1.128415081\H,-0.000
0594182,1.0220782423,-1.5406987017\H,0.885092728,-0.5110723569,-1.5405
733118\H,-0.8850714892,-0.5109915732,-1.540643861\\Version=EM64L-G16Re
vA.03\State=1-A\HF=-438.1189721\RMSD=6.063e-09\RMSF=2.711e-05\Dipole=-
0.0001159,-0.000099,-1.3791824\Quadrupole=0.4340081,0.4341476,-0.86815
57,-0.0001765,-0.0000607,-0.0001325\PG=C01 [X(C1H3S1)]\\@

1\1\GINC-ORC215\FOpt\RM08HX\6-311G(2d,p)\C1H3S1(1-)\SNIKOO\01-Aug-2017
\0\\# opt freq 6-311g(2d,p) m08hx\\CH3-S(-)\\-1,1\S,0.7073729931,-0.00
00225804,-0.0000017745\C,-1.1237489632,0.0000247621,-0.0000115667\H,-1
.534280305,-0.9109755866,0.4606602012\H,-1.5341193389,0.8545189095,0.5
586747217\H,-1.534190386,0.0565264953,-1.0193705817\\Version=EM64L-G16
RevA.03\State=1-A\HF=-438.1202044\RMSD=3.786e-09\RMSF=1.439e-04\Dipole
=-1.287592,0.0000056,0.0000213\Quadrupole=-0.7655958,0.3827681,0.38282
77,0.0000824,-0.0000288,0.000108\PG=C01 [X(C1H3S1)]\\@

N-N= 4.761137776905D+01 E-N=-1.141537686743D+03 KE= 4.375341005941D+02
1\1\GINC-ORC329\FOpt\RM08HX\6-311+G(df,p)\C1H3S1(1-)\SNIKOO\25-Jul-201
7\0\\# opt freq 6-311+g(df,p) m08hx\\CH3-S(-)\\-1,1\S,-0.0000356042,-0
.0001596061,0.7028940763\C,0.0000152578,0.0000856117,-1.1263258324\H,0
.0000404378,1.0213742487,-1.5414381272\H,0.8845572178,-0.5106208364,-1
.5411548676\H,-0.8845773093,-0.5106792179,-1.5410712491\\Version=EM64L
-G16RevA.03\State=1-A\HF=-438.1210799\RMSD=6.890e-09\RMSF=1.775e-04\Di
pole=-0.0000466,-0.0000379,-1.3732852\Quadrupole=0.4718101,0.4717223,-
0.9435324,0.0000896,-0.0002664,-0.0005502\PG=C01 [X(C1H3S1)]\\@

1\1\GINC-ORC202\FOpt\RM08HX\6-311+G(2df,p)\C1H3S1(1-)\SNIKOO\25-Jul-20
17\0\\# opt freq 6-311+g(2df,p) m08hx\\CH3-S(-)\\-1,1\S,0.000016704,0.
0000762633,0.6976727867\C,-0.0000174351,-0.000090156,-1.1277248682\H,0
.0000010373,1.0210899501,-1.5389457871\H,0.8844393011,-0.5105371484,-1
.5390324747\H,-0.8844396073,-0.510538709,-1.5390656567\\Version=EM64L-
G16RevA.03\State=1-A\HF=-438.124104\RMSD=4.045e-09\RMSF=3.714e-05\Dipo
le=-0.0000084,0.0001182,-1.3179886\Quadrupole=0.4546999,0.4541107,-0.9
088106,0.0000153,0.0000614,0.0002278\PG=C01 [X(C1H3S1)]\\@

N-N= 4.778276627086D+01 E-N=-1.141888145599D+03 KE= 4.375206366921D+02
1\1\GINC-ORC218\FOpt\RM08HX\6-311++G(3df,3pd)\C1H3S1(1-)\ROOT\01-Aug-2
017\0\\# opt freq 6-311++g(3df,3pd) m08hx\\CH3-S(-)\\-1,1\S,0.70013080
61,-0.0000215381,0.0000005934\C,-1.1234218174,0.0000251444,-0.00001775
76\H,-1.5319756214,-0.9095998569,0.4599728023\H,-1.5318121936,0.853227
767,0.5578411523\H,-1.5318871738,0.0564404837,-1.0178457905\\Version=E
M64L-G16RevA.03\State=1-A\HF=-438.127466\RMSD=4.759e-09\RMSF=6.473e-05
\Dipole=-1.2753111,-0.0001221,0.0001027\Quadrupole=-1.0192622,0.509555

```
3,0.509707,-0.0001104,0.0000966,0.0001522\PG=C01 [X(C1H3S1)]\\@
```

CH_3S^- QCISD

```
1\1\GINC-ORC14\FOpt\RQCISD-FC\6-311+G(2df,p)\C1H3S1(1-)\SNIKOO\19-Aug-
 2017\0\\# opt freq 6-311+g(2df,p) qcisd\\CH3-S(-)\\-1,1\S,0.,0.0000000
 4,0.706398519\C,0.,0.00000004,-1.1300481519\H,0.0000028504,1.020018080
 4,-1.5384388975\H,0.8833601101,-0.5100114487,-1.5384388975\H,-0.883362
 9605,-0.5100065117,-1.5384388975\\Version=EM64L-G09RevD.01\State=1-A1\
 HF=-437.1664466\MP2=-437.5205804\MP3=-437.5489367\MP4D=-437.5581267\MP
 4DQ=-437.5485896\MP4SDQ=-437.5508177\QCISD=-437.550735\RMSD=4.536e-09\
 RMSF=1.194e-04\Dipole=0.,0.,-1.343597\PG=C03V [C3(C1S1),3SGV(H1)]\\@
```

$CH_3S^\bullet$ B3LYP

```
1\1\GINC-ORC56\FOpt\UB3LYP\6-31G(d)\C1H3S1(2)\SNIKOO\02-Oct-2017\0\\#
 opt freq=noraman ub3lyp/6-31g(d) geom=check\\ch3s. radical taken from
 Cristins paper\\0,2\S,0.0052537619,-0.7012961629,0.\C,0.0030458717,1.1
 112559839,0.\H,-1.0491163116,1.4281193529,0.\H,0.474868658,1.517309972
 4,0.8991256866\H,0.474868658,1.5173099724,-0.8991256866\\Version=EM64L
 -G09RevD.01\State=2-A'\HF=-438.0597242\S2=0.75249\S2-1=0.\S2A=0.750003
 \RMSD=5.135e-09\RMSF=1.020e-04\Dipole=-0.0380944,0.7048128,0.\Quadrupo
 le=0.7359808,0.4519605,-1.1879414,-0.1208615,0.,0.\PG=CS [SG(C1H1S1),X
 (H2)]\\@
```

```
1\1\GINC-ORC104\FOpt\UB3LYP\6-311G(2d,p)\C1H3S1(2)\SNIKOO\29-Sep-2017\
 0\\# opt freq ub3lyp/6-311g(2d,p)\\ch3s. radical taken from Cristins p
 aper\\0,2\S,0.0043200279,-0.6924297354,0.\C,0.0044096313,1.1146953089,
 0.\H,-1.0467380135,1.4234645066,0.\H,0.4734455044,1.5134896295,0.89880
 68064\H,0.4734455044,1.5134896295,-0.8988068064\\Version=EM64L-G09RevD
 .01\State=2-A'\HF=-438.1020986\S2=0.753435\S2-1=0.\S2A=0.750005\RMSD=5
 .783e-09\RMSF=8.277e-05\Dipole=-0.0298948,0.6922447,0.\Quadrupole=0.75
 10792,0.5411276,-1.2922068,-0.1189323,0.,0.\PG=CS [SG(C1H1S1),X(H2)]\\
 @
```

```
1\1\GINC-ORC5\FOpt\UB3LYP\6-311+G(2df,p)\C1H3S1(2)\SNIKOO\02-Oct-2017\
 0\\# opt freq=noraman ub3lyp/6-311+g(2df,p)\\ch3s. radical taken from
 Cristins paper\\0,2\S,0.0043046565,-0.6894398637,0.\C,0.0040203625,1.1
 134468577,0.\H,-1.0469007931,1.4227926855,0.\H,0.4737312929,1.51295426
 91,0.8983051308\H,0.4737312929,1.5129542691,-0.8983051308\\Version=EM6
 4L-G09RevD.01\State=2-A'\HF=-438.1059012\S2=0.753952\S2-1=0.\S2A=0.750
 007\RMSD=8.818e-09\RMSF=2.394e-04\Dipole=-0.0293557,0.6807209,0.\Quadr
 upole=0.7326018,0.462798,-1.1953998,-0.113703,0.,0.\PG=CS [SG(C1H1S1),
 X(H2)]\\@
```

```
1\1\GINC-ORC72\FOpt\UB3LYP\6-311++G(3df,3pd)\C1H3S1(2)\SNIKOO\02-Oct-2
 017\0\\# opt freq=noraman ub3lyp/6-311++g(3df,3pd)\\ch3s. radical take
 n from Cristins paper\\0,2\S,0.0037820785,-0.6861769423,0.\C,0.0045359
 644,1.1123071672,0.\H,-1.0446623061,1.4216487917,0.\H,0.4726190137,1.5
 124636581,0.8968142275\H,0.4726190137,1.5124636581,-0.8968142275\\Vers
 ion=EM64L-G09RevD.01\State=2-A'\HF=-438.1084726\S2=0.754063\S2-1=0.\S2
 A=0.750008\RMSD=3.891e-09\RMSF=1.536e-04\Dipole=-0.0272689,0.6725413,0
 .\Quadrupole=0.7283803,0.4424306,-1.1708109,-0.1102903,0.,0.\PG=CS [SG
 (C1H1S1),X(H2)]\\@
```

$CH_3S^•$ B3PW91

```
1\1\GINC-ORC23\FOpt\UB3PW91\6-31G(d)\C1H3S1(2)\SNIKOO\02-Oct-2017\0\\#
  opt freq=noraman ub3pw91/6-31g(d)\\ch3s. radical taken from Cristins
 paper\\0,2\S,0.0054823468,-0.6950697332,0.\C,0.0033345766,1.1059873323
 ,0.\H,-1.0488232272,1.423998459,0.\H,0.4747663154,1.5129165905,0.89952
 22203\H,0.4747663154,1.5129165905,-0.8995222203\\Version=EM64L-G09RevD
 .01\State=2-A'\HF=-437.9936828\S2=0.752781\S2-1=0.\S2A=0.750003\RMSD=9
 .489e-09\RMSF=1.052e-04\Dipole=-0.0409948,0.71777,0.\Quadrupole=0.7322
 874,0.4507034,-1.1829908,-0.1269988,0.,0.\PG=CS [SG(C1H1S1),X(H2)]\\@
```

```
1\1\GINC-ORC24\FOpt\UB3PW91\6-311G(2d,p)\C1H3S1(2)\SNIKOO\02-Oct-2017\
 0\\# opt freq=noraman ub3pw91/6-311g(2d,p)\\ch3s. radical taken from C
 ristins paper\\0,2\S,0.0031693259,-0.6856027255,0.\C,0.0056732953,1.10
 96653725,0.\H,-1.0463679529,1.4173490145,0.\H,0.4735101036,1.509673054
 4,0.89983332\H,0.4735101036,1.5096730544,-0.89983332\\Version=EM64L-G0
 9RevD.01\State=2-A'\HF=-438.0336422\S2=0.75394\S2-1=0.\S2A=0.750006\RM
 SD=3.288e-09\RMSF=1.454e-04\Dipole=-0.0317272,0.6986444,0.\Quadrupole=
 0.7285259,0.5448981,-1.273424,-0.1233601,0.,0.\PG=CS [SG(C1H1S1),X(H2)
 ]\\@
```

```
1\1\GINC-ORC23\FOpt\UB3PW91\6-311+G(2df,p)\C1H3S1(2)\SNIKOO\02-Oct-201
 7\0\\# opt freq=noraman ub3pw91/6-311+g(2df,p)\\ch3s. radical taken fr
 om Cristins paper\\0,2\S,0.0026635614,-0.6828992198,0.\C,0.0057937,1.1
 061843792,0.\H,-1.0457187396,1.4169346266,0.\H,0.4733851147,1.51026710
 51,0.8987528903\H,0.4733851147,1.5102671051,-0.8987528903\\Version=EM6
 4L-G09RevD.01\State=2-A'\HF=-438.0376468\S2=0.754605\S2-1=0.\S2A=0.750
 009\RMSD=3.735e-09\RMSF=1.899e-05\Dipole=-0.0314401,0.6875453,0.\Quadr
 upole=0.7055661,0.4712129,-1.1767789,-0.1186852,0.,0.\PG=CS [SG(C1H1S1
 ),X(H2)]\\@
```

```
1\1\GINC-ORC30\FOpt\UB3PW91\6-311++G(3df,3pd)\C1H3S1(2)\SNIKOO\02-Oct-
 2017\0\\# opt freq=noraman ub3pw91/6-311++g(3df,3pd)\\ch3s. radical ta
 ken from Cristins paper\\0,2\S,0.0024229672,-0.6802248837,0.\C,0.00625
 24771,1.1055761814,0.\H,-1.0439441601,1.4161744455,0.\H,0.4723888494,1
 .5096140958,0.897755213\H,0.4723888494,1.5096140958,-0.897755213\\Vers
 ion=EM64L-G09RevD.01\State=2-A'\HF=-438.0399112\S2=0.754779\S2-1=0.\S2
 A=0.75001\RMSD=4.236e-09\RMSF=2.510e-05\Dipole=-0.028995,0.6808512,0.\
 Quadrupole=0.70508,0.4480026,-1.1530827,-0.1160801,0.,0.\PG=CS [SG(C1H
 1S1),X(H2)]\\@
```

$CH_3S^•$ wB97XD

```
1\1\GINC-ORC3\FOpt\ROwB97XD\6-31G(d)\C1H3S1(2)\SNIKOO\20-Sep-2017\0\\#
  opt freq=noraman uwb97xd/6-31g(d)\\CH3-S 1\\0,2\S,0.0457662884,-0.65
 70171471,-0.0000073128\C,0.0434536493,1.1428972285,0.0000264432\H,-0.4
 279760765,1.5477624857,0.8987736663\H,1.0945724995,1.4616576226,-0.000
 0363794\H,-0.4279243606,1.5477528103,-0.8987564174\\Version=EM64L-G09R
 evD.01\State=2-A\HF=-438.0221559\RMSD=8.629e-09\RMSF=1.671e-04\Dipole=
 0.0383728,0.7215061,-0.0000348\Quadrupole=0.7432032,0.4365977,-1.17980
 1,0.1240322,0.0007315,-0.0000968\PG=C01 [X(C1H3S1)]\\@
```

```
1\1\GINC-ORC15\FOpt\ROwB97XD\6-311G(2d,p)\C1H3S1(2)\SNIKOO\20-Sep-2017
 \0\\# opt freq uwb97xd/6-311g(2d,p) geom=connectivity nosymm\\CH3-S 1
 \\0,2\S,0.0476368031,-0.6486352381,-0.0000080528\C,0.0415636803,1.1458
 076098,0.0000192018\H,-0.4267974525,1.5449100977,0.8988991666\H,1.0922
 552173,1.4560727755,-0.0000212203\H,-0.4267672482,1.5448967551,-0.8988
```

```
890953\\Version=EM64L-G09RevD.01\HF=-438.0618516\RMSD=9.626e-09\RMSF=4
.176e-05\Dipole=0.0295039,0.6993315,-0.000017\Quadrupole=0.703429,0.61
17076,-1.3151366,0.1888316,0.0004894,-0.0000354\PG=C01 [X(C1H3S1)]\\@
```

```
1\1\GINC-ORC31\FOpt\ROwB97XD\6-311+G(2df,p)\C1H3S1(2)\SNIKOO\20-Sep-20
17\0\\# opt nosymm freq=noraman uwb97xd/6-311+g(2df,p)\\CH3-S 1\\0,2\
S,0.0480061918,-0.6453774464,-0.0000081997\C,0.0418078451,1.1430842589
,0.0000152498\H,-0.4269476682,1.5450217399,0.8978881945\H,1.0919504155
,1.4553173739,-0.0000155845\H,-0.4269247843,1.5450070737,-0.8978796602
\\Version=EM64L-G09RevD.01\HF=-438.0653837\RMSD=7.331e-09\RMSF=4.474e-
05\Dipole=0.0289269,0.6906071,-0.0000148\Quadrupole=0.6762462,0.543804
9,-1.2200511,0.1836021,0.0004812,-0.0000385\PG=C01 [X(C1H3S1)]\\@
```

```
1\1\GINC-ORC3\FOpt\ROwB97XD\6-311++G(3df,3pd)\C1H3S1(2)\SNIKOO\20-Sep-
2017\0\\# opt freq=noraman uwb97xd/6-311++g(3df,3pd) nosymm\\CH3-S 1\
\0,2\S,0.0482943309,-0.6425201393,-0.0000077729\C,0.0412988067,1.14268
30362,0.0000175606\H,-0.4258255128,1.54425899,0.8967118981\H,1.0899208
129,1.454385173,-0.0000200897\H,-0.4257964377,1.5442459402,-0.89670159
61\\Version=EM64L-G09RevD.01\HF=-438.0679634\RMSD=8.665e-09\RMSF=1.269
e-04\Dipole=0.0258366,0.6851072,-0.0000148\Quadrupole=0.6671691,0.5307
86,-1.1979551,0.1800826,0.0004679,-0.0000412\PG=C01 [X(C1H3S1)]\\@
```

$CH_3S^{\bullet}$ M062X

```
1\1\GINC-ORC56\FOpt\UM062X\6-31G(d)\C1H3S1(2)\SNIKOO\02-Oct-2017\0\\#
OPT FREQ m062x/6-31g(d)\\ch3s. radical taken from Cristins paper\\0,2\
S,0.0057084948,-0.6911267112,0.\C,0.0020963581,1.1111115442,0.\H,-1.04
84095336,1.4254793879,0.\H,0.4747569194,1.5136189622,0.8976093424\H,0.
4747569194,1.5136189622,-0.8976093424\\Version=EM64L-G09RevD.01\State=
2-A'\HF=-437.9909586\S2=0.752694\S2-1=0.\S2A=0.750005\RMSD=4.616e-09\R
MSF=7.390e-05\Dipole=-0.0366154,0.6906458,0.\Quadrupole=0.7679753,0.39
80148,-1.1659901,-0.1130679,0.,0.\PG=CS [SG(C1H1S1),X(H2)]\\@
```

```
1\1\GINC-ORC26\FOpt\UM062X\6-311G(2d,p)\C1H3S1(2)\SNIKOO\02-Oct-2017\0
\\# opt freq m062x/6-311g(2d,p)\\ch3s. radical taken from Cristins pap
er\\0,2\S,0.0063898544,-0.6918533424,0.\C,0.0030601524,1.1059062629,0.
\H,-1.0472954117,1.4103316684,0.\H,0.4715824446,1.5032938816,0.8973695
656\H,0.4715824446,1.5032938816,-0.8973695656\\Version=EM64L-G09RevD.0
1\State=2-A'\HF=-438.0367129\S2=0.753801\S2-1=0.\S2A=0.750009\RMSD=5.4
50e-10\RMSF=7.231e-05\Dipole=-0.0298272,0.6790723,0.\Quadrupole=0.7671
505,0.5087233,-1.2758739,-0.109667,0.,0.\PG=CS [SG(C1H1S1),X(H2)]\\@
```

```
1\1\GINC-ORC19\FOpt\UM062X\6-311+G(2df,p)\C1H3S1(2)\SNIKOO\02-Oct-2017
\0\\# opt freq 6-311+g(2df,p) m062x\\ch3s. radical taken from Cristins
 paper\\0,2\S,0.0058997239,-0.6887942736,0.\C,0.0030756798,1.102891747
2,0.\H,-1.0470465505,1.409627277,0.\H,0.4717093612,1.5036200164,0.8966
127365\H,0.4717093612,1.5036200164,-0.8966127365\\Version=EM64L-G09Rev
D.01\State=2-A'\HF=-438.0403967\S2=0.753916\S2-1=0.\S2A=0.75001\RMSD=5
.657e-09\RMSF=7.257e-05\Dipole=-0.0293338,0.6696691,0.\Quadrupole=0.73
05914,0.4407899,-1.1713813,-0.1052239,0.,0.\PG=CS [SG(C1H1S1),X(H2)]\\
@
```

```
1\1\GINC-ORC11\FOpt\UM062X\6-311++G(3df,3pd)\C1H3S1(2)\SNIKOO\02-Oct-2
017\0\\# opt freq 6-311++g(3df,3pd) m062x\\ch3s. radical taken from Cr
istins paper\\0,2\S,0.0043597165,-0.6893455289,0.\C,0.0049507263,1.100
1497204,0.\H,-1.0429326499,1.4083458437,0.\H,0.4736069874,1.49914402,0
.8952317404\H,0.4736069874,1.49914402,-0.8952317404\\Version=EM64L-G09
```

RevD.01\State=2-A'\HF=-438.0431929\S2=0.753887\S2-1=0.\S2A=0.75001\RMSD=7.999e-09\RMSF=4.229e-05\Dipole=-0.0253219,0.6600195,0.\Quadrupole=0.7202754,0.4292013,-1.1494766,-0.0998449,0.,0.\PG=CS [SG(C1H1S1),X(H2)]\\@

$CH_3S^{\bullet}$ M08HX

1\1\GINC-ORC204\FOpt\ROM08HX\6-31G(d)\C1H3S1(2)\SNIKOO\20-Sep-2017\0\\# opt freq=NORAMAN 6-31g(d) rom08hx\\CH3-S(rad) 1\\0,2\S,-0.6538413209,-0.0841446724,-0.0000555501\C,1.1416830298,0.0228543711,-0.0000818386\H,1.525555366,-1.009607972,0.0007990431\H,1.5202331755,0.5185235736,-0.9014760811\H,1.5203467497,0.5202066997,0.9003194267\\Version=EM64L-G16RevA.03\State=2-A\HF=-437.9994975\RMSD=3.257e-09\RMSF=2.377e-04\Dipole=0.6815655,0.0024433,-0.000055\Quadrupole=0.4285576,0.7404793,-1.1690368,-0.1417006,0.0001997,-0.0032675\PG=C01 [X(C1H3S1)]\\@

1\1\GINC-ORC3\FOpt\ROM08HX\6-311G(2d,p)\C1H3S1(2)\SNIKOO\20-Sep-2017\0\\# opt freq=noraman 6-311g(2d,p) nosymm ROm08hx\\CH3-S(rad) 1\\0,2\S,-0.6450140036,-0.0854306928,-0.000081431\C,1.1452337241,0.0247139742,-0.0000436731\H,1.5195416059,-1.0074397417,0.0007593655\H,1.5170265454,0.5171229156,-0.9011027483\H,1.5171881282,0.5188655447,0.8999744868\\Version=EM64L-G16RevA.03\HF=-438.047696\RMSD=1.807e-09\RMSF=3.772e-05\Dipole=0.6666141,0.0108501,-0.0000886\Quadrupole=0.6054142,0.6854842,-1.2908984,-0.1905086,0.0001454,-0.0045131\PG=C01 [X(C1H3S1)]\\@

1\1\GINC-ORC3\FOpt\ROM08HX\6-311+G(2df,p)\C1H3S1(2)\SNIKOO\20-Sep-2017\0\\# opt freq=noraman 6-311+g(2df,p) rom08hx\\CH3-S(rad) 1\\0,2\S,-0.6423453242,-0.0858150448,-0.0000311845\C,1.1422221885,0.0243735523,-0.0001010013\H,1.5188340969,-1.0072933339,0.0007969738\H,1.5175998742,0.517445939,-0.9002107483\H,1.5176661646,0.5191208874,0.8990509602\\Version=EM64L-G16RevA.03\State=2-A\HF=-438.0516807\RMSD=1.717e-09\RMSF=3.150e-05\Dipole=0.6543742,0.0096867,-0.0000368\Quadrupole=0.4475904,0.6998941,-1.1474845,-0.1282051,0.000144,-0.0024784\PG=C01 [X(C1H3S1)]\\@

1\1\GINC-ORC19\FOpt\ROM08HX\6-311++G(3df,3pd)\C1H3S1(2)\SNIKOO\20-Sep-2017\0\\# opt freq=NORAMAN 6-311++g(3df,3pd) rom08hx\\CH3-S(rad) 1\\0,2\S,-0.6398041858,-0.0856848969,-0.000038354\C,1.1431727531,0.0247454399,-0.0000918857\H,1.5177923099,-1.0050475494,0.0007880855\H,1.5163685494,0.5160677911,-0.8987578892\H,1.5164475734,0.5177512153,0.8976050435\\Version=EM64L-G16RevA.03\State=2-A\HF=-438.0550379\RMSD=2.372e-09\RMSF=1.184e-04\Dipole=0.6570979,0.0136584,-0.000043\Quadrupole=0.4274984,0.697217,-1.1247154,-0.124585,0.0001538,-0.0026994\PG=C01 [X(C1H3S1)]\\@

$CH3S^{\cdot}$ QCISD

1\1\GINC-ORC320\FOpt\UQCISD-FC\6-311+G(2df,p)\C1H3S1(2)\SNIKOO\02-Oct-2017\0\\# opt freq=noraman 6-311+g(2df,p) guess=read qcisd\\ch3s. radical taken from Cristins paper\\0,2\S,0.0057621099,-0.6893471194,0.\C,0.0016990524,1.1137771606,0.\H,-1.0494642478,1.4240176247,0.\H,0.4754400302,1.5121316149,0.8978812286\H,0.4754400302,1.5121316149,-0.8978812286\\Version=EM64L-G09RevD.01\State=2-A'\HF=-437.14419\MP2=-437.4571118\MP3=-437.4894203\MP4D=-437.4985482\MP4DQ=-437.4906251\PUHF=-437.1478563\PMP2-0=-437.459289\PMP3-0=-437.4905474\MP4SDQ=-437.4928442\QCISD=-437.4933178\S2=0.763995\S2-1=0.752596\S2A=0.750062\RMSD=4.620e-09\RMSF=1.820e-04\Dipole=-0.0252823,0.6445234,0.\PG=CS [SG(C1H1S1),X(H2)]\\@

CH_3SSH B3LYP

1\1\GINC-ORC358\FOpt\RB3LYP\6-31G(d)\C1H4S2\SNIKOO\06-Jun-2017\0\\# op
t freq b3lyp/6-31g(d)\\CH3-SS-H\\0,1\S,-1.3775141928,0.2342882801,-0.0
846414729\S,0.4873700237,-0.7068447326,0.0123422404\C,1.6594862743,0.7
036925595,-0.0062333937\H,1.569663752,1.306038346,0.9007905191\H,2.659
3602791,0.2586524908,-0.0459480163\H,1.5053950157,1.3257026522,-0.8903
545422\H,-1.5955081521,0.437266404,1.2390256656\\Version=EM64L-G09RevD
.01\State=1-A\HF=-836.8899907\RMSD=9.185e-09\RMSF=7.317e-06\Dipole=0.4
342203,0.5398984,0.3895762\Quadrupole=1.6142216,-1.4830378,-0.1311839,
0.3298117,-1.4696016,0.4803458\PG=C01 [X(C1H4S2)]\\@

1\1\GINC-ORC318\FOpt\RB3LYP\6-31G(d,p)\C1H4S2\SNIKOO\21-Aug-2017\0\\#
opt freq=noraman rb3lyp/6-31g(d,p)\\CH3-SS-H\\0,1\S,-1.3824460298,0.24
27608508,-0.0847282431\S,0.4739153795,-0.7164798184,0.0125190955\C,1.6
60686206,0.6810581373,-0.0092924384\H,1.5763006909,1.2854142259,0.8955
276777\H,2.6536855517,0.2233768221,-0.0467164892\H,1.5140195881,1.3004
367201,-0.8951468372\H,-1.5942363864,0.4466710622,1.2376672348\\Versio
n=EM64L-G09RevD.01\State=1-A\HF=-836.8972751\RMSD=4.121e-09\RMSF=3.264
e-05\Dipole=0.4368248,0.5361259,0.3843114\Quadrupole=1.6218584,-1.4938
473,-0.1280111,0.3076096,-1.4525404,0.4896238\PG=C01 [X(C1H4S2)]\\@

1\1\GINC-ORC248\FOpt\RB3LYP\6-311G(d)\C1H4S2\SNIKOO\05-Jun-2017\0\\# o
pt freq b3lyp/6-311g(d)\\CH3-SS-H (trans)\\0,1\S,-1.3664383712,-0.0878
252201,-0.6256993537\S,0.5983994778,-0.8097897805,-0.4209400383\C,1.52
31290114,0.7032944617,0.0314757529\H,1.2260094551,1.0711765159,1.01307
50737\H,2.5753520064,0.4115964683,0.0609515608\H,1.3873576321,1.480313
6431,-0.7191671182\H,-1.7789180716,-0.2168221283,0.6605859728\\Version
=EM64L-G09RevD.01\State=1-A\HF=-836.9501027\RMSD=5.161e-09\RMSF=1.872e
-05\Dipole=0.2872055,0.4904254,0.5672924\Quadrupole=1.8474763,-1.52216
06,-0.3253157,0.9512283,-1.1202199,0.0867457\PG=C01 [X(C1H4S2)]\\@

1\1\GINC-ORC114\FOpt\RB3LYP\6-311+G(d)\C1H4S2\SNIKOO\05-Jun-2017\0\\#
opt freq b3lyp/6-311+g(d)\\CH3-SS-H (trans)\\0,1\S,-1.3687002926,-0.07
21246444,-0.6331205612\S,0.5895938204,-0.8071874128,-0.4236935044\C,1.
5264962462,0.6962539805,0.0352430519\H,1.2309021636,1.0636506385,1.017
7659404\H,2.5766212416,0.396540772,0.0653568508\H,1.3977475234,1.47762
57722,-0.712555417\H,-1.7877695625,-0.202815146,0.6512854894\\Version=
EM64L-G09RevD.01\State=1-A\HF=-836.9522407\RMSD=9.302e-09\RMSF=3.944e-
06\Dipole=0.282798,0.4701499,0.5487828\Quadrupole=1.8675621,-1.5254991
,-0.342063,0.930238,-1.1121606,0.1164997\PG=C01 [X(C1H4S2)]\\@

\1\GINC-ORC178\FOpt\RB3LYP\6-311G(d,p)\C1H4S2\SNIKOO\21-Aug-2017\0\\#
opt freq=noraman rb3lyp/6-311g(d,p)\\CH3-SS-H\\0,1\S,-1.3816206096,0.
2511827992,-0.0880612726\S,0.4811456645,-0.7228727933,0.0143150656\C,1
.6581276881,0.6793048697,-0.0080682105\H,1.5703835983,1.2816590647,0.8
956133193\H,2.6522172085,0.2281007446,-0.047348831\H,1.5063146441,1.29
6749341,-0.8922006347\H,-1.5846431939,0.4491139741,1.2355805638\\Versi
on=EM64L-G09RevD.01\State=1-A\HF=-836.9589071\RMSD=6.850e-09\RMSF=1.67
5e-05\Dipole=0.4341299,0.5358426,0.3844058\Quadrupole=1.6305303,-1.572
6028,-0.0579274,0.2317348,-1.435998,0.4851351\PG=C01 [X(C1H4S2)]\\@

1\1\GINC-ORC291\FOpt\RB3LYP\6-311+G(d,p)\C1H4S2\SNIKOO\18-Jul-2017\0\\
opt freq rb3lyp/6-311+g(d,p)\\CH3-SS-H\\0,1\S,-1.3785378615,0.244120
8335,-0.0872367437\S,0.4935500286,-0.7096328824,0.0143473296\C,1.65941
64515,0.7014262898,-0.0050488729\H,1.5684816725,1.3006754076,0.9007332
442\H,2.6571937326,0.2582637352,-0.0469119134\H,1.5018014852,1.3212764

541,-0.8868906981\H,-1.5863705089,0.4353341622,1.2368106544\\Version=E
M64L-G09RevD.01\State=1-A\HF=-836.960886\RMSD=3.825e-09\RMSF=4.491e-05
\Dipole=0.4155391,0.5199127,0.3742269\Quadrupole=1.6166879,-1.5539008,
-0.0627871,0.2557588,-1.4483047,0.4798403\PG=C01 [X(C1H4S2)]\\@

1\1\GINC-ORC140\FOpt\RB3LYP\6-311G(2d,p)\C1H4S2\SNIKOO\26-Jul-2017\0\\
opt freq rb3lyp/6-311g(2d,p)\\CH3-SS-H\\0,1\S,-1.3723491181,0.240008
1818,-0.0863381558\S,0.4882667569,-0.7028965381,0.0155747545\C,1.66097
50504,0.7020860822,-0.0046666584\H,1.5695962573,1.2985393243,0.9009705
03\H,2.6549379116,0.251934143,-0.0450762012\H,1.5051995991,1.317563727
,-0.8879389492\H,-1.5799504572,0.4301270797,1.233233707\\Version=EM64L
-G09RevD.01\State=1-A\HF=-836.9702766\RMSD=2.969e-09\RMSF=1.340e-05\Di
pole=0.4297141,0.5035195,0.3311863\Quadrupole=1.6935158,-1.5368482,-0.
1566676,0.1714922,-1.2547396,0.4316727\PG=C01 [X(C1H4S2)]\\@

1\1\GINC-ORC61\FOpt\RB3LYP\6-311G(df,p)\C1H4S2\SNIKOO\21-Aug-2017\0\\#
opt freq=noraman rb3lyp/6-311g(df,p)\\CH3-SS-H\\0,1\S,-1.3756910222,0
.2453196195,-0.08814253\S,0.4780890279,-0.7199416471,0.0149041408\C,1.
6570084721,0.678615075,-0.0076618767\H,1.571583855,1.2808800453,0.8963
314377\H,2.6505053226,0.2270793788,-0.0493566912\H,1.5041037375,1.2986
551409,-0.8898077865\H,-1.583674393,0.4526303875,1.2335633059\\Version
=EM64L-G09RevD.01\State=1-A\HF=-836.9646012\RMSD=4.964e-09\RMSF=3.653e
-05\Dipole=0.4302964,0.5355756,0.3834631\Quadrupole=1.542268,-1.535575
9,-0.0066921,0.3035734,-1.4317945,0.48926\PG=C01 [X(C1H4S2)]\\@

1\1\GINC-ORC335\FOpt\RB3LYP\6-311+G(df,p)\C1H4S2\SNIKOO\18-Jul-2017\0\
\# opt freq rb3lyp/6-311+g(df,p)\\CH3-SS-H\\0,1\S,-1.3724930208,0.2386
815933,-0.0865571822\S,0.4902731488,-0.7063510899,0.0136933514\C,1.658
2845973,0.7007901855,-0.0049530812\H,1.5688787826,1.3004767946,0.90070
15542\H,2.6554381619,0.257032493,-0.0476447347\H,1.5012237327,1.322840
6947,-0.8853398901\H,-1.5860704024,0.4379933288,1.2359029825\\Version=
EM64L-G09RevD.01\State=1-A\HF=-836.9666819\RMSD=8.124e-09\RMSF=4.206e-
06\Dipole=0.4126109,0.5181096,0.3720415\Quadrupole=1.5303215,-1.514507
6,-0.0158139,0.3231104,-1.4459803,0.4849448\PG=C01 [X(C1H4S2)]\\@

1\1\GINC-ORC298\FOpt\RB3LYP\6-311+G(2df,p)\C1H4S2\SNIKOO\18-Jul-2017\0
\\# opt freq rb3lyp/6-311+g(2df,p)\\CH3-SS-H\\0,1\S,-1.3649711824,0.23
66813682,-0.0860524226\S,0.4834631334,-0.6950484785,0.0150750064\C,1.6
561876765,0.7025405795,-0.0048504146\H,1.5674190771,1.3008793957,0.900
3777926\H,2.6502595939,0.2525617251,-0.0456437528\H,1.5023576651,1.321
1649246,-0.8868686817\H,-1.5791809636,0.4326844854,1.2337654728\\Versi
on=EM64L-G09RevD.01\State=1-A\HF=-836.9778216\RMSD=3.558e-09\RMSF=1.08
0e-05\Dipole=0.4076774,0.4810375,0.3225407\Quadrupole=1.5933611,-1.507
5231,-0.085838,0.2033133,-1.2689836,0.4488367\PG=C01 [X(C1H4S2)]\\@

1\1\GINC-ORC231\FOpt\RB3LYP\6-311++G(3df,3pd)\C1H4S2\SNIKOO\26-Jul-201
7\0\\# opt freq rb3lyp/6-311++g(3df,3pd)\\CH3-SS-H\\0,1\S,-1.359363700
1,0.2346920565,-0.0838323691\S,0.4830695107,-0.6915134234,0.0127432208
\C,1.656189691,0.7003232382,-0.0053272502\H,1.5654510172,1.2989523112,
0.8974644573\H,2.6477642582,0.2484603327,-0.0414066035\H,1.5081841939,
1.317569916,-0.8873441253\H,-1.574618971,0.4288775688,1.23346167\\Vers
ion=EM64L-G09RevD.01\State=1-A\HF=-836.9827594\RMSD=7.493e-09\RMSF=1.6
55e-05\Dipole=0.4155322,0.4616528,0.2863922\Quadrupole=1.6014749,-1.46
06026,-0.1408723,0.2122763,-1.135808,0.4109845\PG=C01 [X(C1H4S2)]\\@

CH_3SSH B3PW91

1\1\GINC-ORC85\FOpt\RB3PW91\6-31G(d)\C1H4S2\SNIKOO\20-Jul-2017\0\\# op
t freq rb3pw91/6-31g(d)\\CH3-SS-H\\0,1\S,-1.3556854419,0.2433803198,-0
.0900530476\S,0.484271305,-0.7023877125,0.0182239263\C,1.6598535413,0.
6886852699,-0.0040116706\H,1.5778498531,1.295781037,0.9010831307\H,2.6
571302635,0.23719501,-0.0428669684\H,1.5131177931,1.3104553768,-0.8901
100899\H,-1.579903314,0.4548796989,1.2286847193\\Version=EM64L-G09RevD
.01\State=1-A\HF=-836.7752234\RMSD=6.061e-09\RMSF=1.195e-05\Dipole=0.4
427787,0.5541289,0.4027984\Quadrupole=1.6444096,-1.5138338,-0.1305759,
0.3146167,-1.512095,0.5095063\PG=C01 [X(C1H4S2)]\\@

1\1\GINC-ORC170\FOpt\RB3PW91\6-31G(d,p)\C1H4S2\SNIKOO\21-Aug-2017\0\\#
opt freq=noraman rb3pw91/6-31g(d,p)\\CH3-SS-H\\0,1\S,-1.3662835172,0.
2410031716,-0.0867612386\S,0.4728831639,-0.7087329464,0.0159747234\C,1
.6524075682,0.6781723864,-0.0076688189\H,1.5741503336,1.2832673099,0.8
977452806\H,2.6460623111,0.2215757877,-0.048802693\H,1.5061138561,1.29
96218431,-0.892740457\H,-1.5834087157,0.4483304477,1.2320832035\\Versi
on=EM64L-G09RevD.01\State=1-A\HF=-836.7822163\RMSD=5.171e-09\RMSF=1.29
6e-05\Dipole=0.442145,0.5520342,0.3972238\Quadrupole=1.6415153,-1.5216
535,-0.1198618,0.3207254,-1.4973378,0.5021736\PG=C01 [X(C1H4S2)]\\@

1\1\GINC-ORC84\FOpt\RB3PW91\6-311G(d)\C1H4S2\SNIKOO\20-Jul-2017\0\\# o
pt freq rb3pw91/6-311g(d) geom=connectivity\\CH3-SS-H\\0,1\S,-1.351196
6667,0.2506920841,-0.0940812366\S,0.4920542275,-0.7092441124,0.0196592
609\C,1.6561477799,0.6861262872,-0.0027720338\H,1.5726184697,1.2907619
188,0.9007885174\H,2.6546251969,0.2435164329,-0.0445315575\H,1.5046333
016,1.3066581027,-0.8856564795\H,-1.572248309,0.4594782868,1.227543529
\\Version=EM64L-G09RevD.01\State=1-A\HF=-836.8320708\RMSD=3.838e-09\RM
SF=7.763e-06\Dipole=0.4331397,0.5527654,0.4137903\Quadrupole=1.6213143
,-1.5773343,-0.0439799,0.2320678,-1.5300376,0.5147188\PG=C01 [X(C1H4S2
)]\\@

1\1\GINC-ORC79\FOpt\RB3PW91\6-311+G(d)\C1H4S2\SNIKOO\20-Jul-2017\0\\#
opt freq rb3pw91/6-311+g(d) geom=connectivity\\CH3-SS-H\\0,1\S,-1.3524
557847,0.2502554196,-0.0941777415\S,0.4913647696,-0.7075359141,0.02074
80221\C,1.6573425539,0.6860249037,-0.0029318039\H,1.5751307464,1.29159
89794,0.9004406371\H,2.6556852287,0.2426927994,-0.0452236905\H,1.50568
95576,1.3073156014,-0.8856509397\H,-1.5761230715,0.4576372107,1.227745
5164\\Version=EM64L-G09RevD.01\State=1-A\HF=-836.834424\RMSD=8.005e-09
\RMSF=8.279e-06\Dipole=0.4229623,0.536034,0.3987887\Quadrupole=1.63032
1,-1.5609424,-0.0693786,0.2354657,-1.5335637,0.5278022\PG=C01 [X(C1H4S
2)]\\@

1\1\GINC-ORC172\FOpt\RB3PW91\6-311G(d,p)\C1H4S2\SNIKOO\21-Aug-2017\0\\
opt freq=noraman rb3pw91/6-311g(d,p)\\CH3-SS-H\\0,1\S,-1.3638779032,
0.24865587,-0.0906464589\S,0.4794962394,-0.714897377,0.0172444564\C,1.
6493120154,0.6763547877,-0.0064495644\H,1.5673203974,1.2796375746,0.89
8202746\H,2.6442249428,0.2255531006,-0.0491169965\H,1.4981257096,1.296
4486794,-0.8899427239\H,-1.5726764014,0.4514853646,1.2305385414\\Versi
on=EM64L-G09RevD.01\State=1-A\HF=-836.8408927\RMSD=5.445e-09\RMSF=2.18
4e-05\Dipole=0.43271,0.5444494,0.395785\Quadrupole=1.6224788,-1.576123
8,-0.046355,0.2515926,-1.4706352,0.4963589\PG=C01 [X(C1H4S2)]\\@

1\1\GINC-ORC78\FOpt\RB3PW91\6-311+G(d,p)\C1H4S2\SNIKOO\20-Jul-2017\0\\
opt freq rb3pw91/6-311+g(d,p) geom=connectivity\\CH3-SS-H\\0,1\S,-1.

3562895884,0.2517331215,-0.0931958572\S,0.4902102281,-0.7045305458,0.0
20098195\C,1.6582032798,0.6880885756,-0.0032029697\H,1.5729130088,1.29
3667282,0.8999192035\H,2.6542298697,0.2390252705,-0.0420024256\H,1.508
5010775,1.3066412476,-0.8883686738\H,-1.5711338754,0.4533640487,1.2277
025278\\Version=EM64L-G09RevD.01\State=1-A\HF=-836.8430572\RMSD=6.928e
-09\RMSF=5.663e-06\Dipole=0.4189021,0.5295659,0.3851758\Quadrupole=1.6
247754,-1.5588369,-0.0659385,0.2471629,-1.4774415,0.5058544\PG=C01 [X(
C1H4S2)]\\@

1\1\GINC-ORC169\FOpt\RB3PW91\6-311G(2d,p)\C1H4S2\SNIKOO\27-Jul-2017\0\
\# opt freq rb3pw91/6-311g(2d,p)\\CH3-SS-H\\0,1\S,-1.3523424643,0.2490
423392,-0.0924521973\S,0.483487215,-0.6947104953,0.0222051968\C,1.6583
544609,0.691449275,-0.0028675684\H,1.5740895996,1.2937811306,0.9007387
375\H,2.6500754872,0.2340517072,-0.0414855133\H,1.5098429471,1.3055120
578,-0.8895789664\H,-1.5668732455,0.4488629854,1.2243903111\\Version=E
M64L-G09RevD.01\State=1-A\HF=-836.8534023\RMSD=7.565e-09\RMSF=1.155e-0
5\Dipole=0.43245,0.5096403,0.3380905\Quadrupole=1.6953388,-1.5401149,-
0.1552239,0.160204,-1.2733152,0.4515734\PG=C01 [X(C1H4S2)]\\@

1\1\GINC-ORC300\FOpt\RB3PW91\6-311G(df,p)\C1H4S2\SNIKOO\21-Aug-2017\0\
\# opt freq=noraman rb3pw91/6-311g(df,p)\\CH3-SS-H\\0,1\S,-1.358502095
,0.2436216007,-0.0902738869\S,0.4766907035,-0.7121001473,0.0170114367\
C,1.6482158613,0.6755010598,-0.0062097886\H,1.5678124163,1.2791837017,
0.8983963116\H,2.642785895,0.224687498,-0.0502523022\H,1.4968304977,1.
297993563,-0.8880432887\H,-1.5719082789,0.4543507241,1.229201518\\Vers
ion=EM64L-G09RevD.01\State=1-A\HF=-836.8467434\RMSD=8.594e-09\RMSF=1.6
47e-05\Dipole=0.4299021,0.5437502,0.3950298\Quadrupole=1.533743,-1.539
1162,0.0053732,0.3268363,-1.4668365,0.4989769\PG=C01 [X(C1H4S2)]\\@

1\1\GINC-ORC73\FOpt\RB3PW91\6-311+G(df,p)\C1H4S2\SNIKOO\20-Jul-2017\0\
\# opt freq rb3pw91/6-311+g(df,p) geom=connectivity\\CH3-SS-H\\0,1\S,-
1.3506970902,0.2463636037,-0.0923810498\S,0.487260794,-0.7013875486,0.
0195917342\C,1.6571116196,0.687379887,-0.0031269943\H,1.5734525522,1.2
934874101,0.899884062\H,2.6526475479,0.237919295,-0.0428990219\H,1.507
7995435,1.3082792963,-0.8868047828\H,-1.5709409671,0.4559470565,1.2266
860526\\Version=EM64L-G09RevD.01\State=1-A\HF=-836.849015\RMSD=7.795e-
09\RMSF=1.038e-05\Dipole=0.41669,0.5279557,0.3828765\Quadrupole=1.5390
124,-1.5194277,-0.0195847,0.3179576,-1.4755217,0.5105153\PG=C01 [X(C1H
4S2)]\\@

1\1\GINC-ORC69\FOpt\RB3PW91\6-311+G(2df,p)\C1H4S2\SNIKOO\20-Jul-2017\0
\\# opt freq rb3pw91/6-311+g(2df,p) geom=connectivity\\CH3-SS-H\\0,1\S
,-1.343924227,0.244573035,-0.092858091\S,0.4803902106,-0.689530189,0.0
217941223\C,1.6555726421,0.6896823612,-0.0027446887\H,1.5735074958,1.2
935493479,0.9006185035\H,2.647469243,0.2325622596,-0.0417598572\H,1.50
8655865,1.3068798534,-0.888162195\H,-1.5650372295,0.4502723319,1.22406
22062\\Version=EM64L-G09RevD.01\State=1-A\HF=-836.8612404\RMSD=6.296e-
09\RMSF=1.816e-05\Dipole=0.4127161,0.4890187,0.3296413\Quadrupole=1.60
14607,-1.5103404,-0.0911202,0.1924082,-1.2856174,0.4687372\PG=C01 [X(C
1H4S2)]\\@

1\1\GINC-ORC165\FOpt\RB3PW91\6-311++G(3df,3pd)\C1H4S2\SNIKOO\27-Jul-20
17\0\\# opt freq rb3pw91/6-311++g(3df,3pd)\\CH3-SS-H\\0,1\S,-1.3407470
888,0.2445540009,-0.0895240557\S,0.4787548375,-0.6844986614,0.01743371
27\C,1.6539340918,0.6897941733,-0.0037975245\H,1.5678766201,1.29423584
95,0.8970574787\H,2.6438161999,0.2311963262,-0.0363447037\H,1.51368737
85,1.3053213297,-0.8897931853\H,-1.560688039,0.4473859819,1.2259182779

```
\\Version=EM64L-G09RevD.01\State=1-A\HF=-836.8656821\RMSD=4.683e-09\RM
SF=2.347e-05\Dipole=0.4223834,0.4697418,0.2931256\Quadrupole=1.6139025
,-1.4701321,-0.1437704,0.201218,-1.1508324,0.431407\PG=C01 [X(C1H4S2)]
\\@
```

CH_3SSH wB97XD

```
1\1\GINC-ORC284\FOpt\RwB97XD\6-31G(d)\C1H4S2\SNIKOO\22-Jul-2017\0\\# o
pt freq wb97xd/6-31g(d)\\CH3-SS-H\\0,1\S,-1.3523247394,0.2444510163,-0
.0913803401\S,0.4809390843,-0.7100539632,0.0193074435\C,1.652434711,0.
6817989624,-0.0030599518\H,1.571288739,1.2833353517,0.9048298545\H,2.6
503721916,0.2356375341,-0.0503766805\H,1.4960742394,1.3067222601,-0.88
43054644\H,-1.5661032259,0.4619798386,1.2224671388\\Version=EM64L-G09R
evD.01\State=1-A\HF=-836.836183\RMSD=2.644e-09\RMSF=7.218e-06\Dipole=0
.4460633,0.5618492,0.4082159\Quadrupole=1.6188159,-1.5147573,-0.104058
6,0.3182648,-1.5306569,0.5417069\PG=C01 [X(C1H4S2)]\\@
```

```
1\1\GINC-ORC318\FOpt\RwB97XD\6-31G(d,p)\C1H4S2\SNIKOO\21-Aug-2017\0\\#
 opt freq=noraman rwb97xd/6-31g(d,p)\\CH3-SS-H\\0,1\S,-1.3583096157,0.
2423848434,-0.0895334667\S,0.4750733398,-0.7139677,0.0185913515\C,1.64
82464934,0.6756042052,-0.0054617379\H,1.5677172866,1.2771571116,0.9013
12514\H,2.643784179,0.2264694854,-0.0521978296\H,1.4927675093,1.298210
0018,-0.8873161318\H,-1.5673541924,0.4573800526,1.2244353004\\Version=
EM64L-G09RevD.01\State=1-A\HF=-836.8431682\RMSD=6.474e-09\RMSF=2.740e-
05\Dipole=0.4437691,0.5593577,0.4011827\Quadrupole=1.6155599,-1.517585
3,-0.0979745,0.324185,-1.509805,0.5336142\PG=C01 [X(C1H4S2)]\\@
```

```
1\1\GINC-ORC279\FOpt\RwB97XD\6-311G(d)\C1H4S2\SNIKOO\22-Jul-2017\0\\#
opt freq wb97xd/6-311g(d)\\CH3-SS-H\\0,1\S,-1.3482785025,0.252144534,-
0.0944538963\S,0.487880662,-0.7169814513,0.0194709651\C,1.6489485907,0
.6794183557,-0.0021357305\H,1.56477245,1.2790528157,0.9040017983\H,2.6
482317572,0.2418259645,-0.0504362727\H,1.489028054,1.3025606498,-0.881
0204983\H,-1.5579020113,0.4658501316,1.2220556343\\Version=EM64L-G09Re
vD.01\State=1-A\HF=-836.893007\RMSD=6.726e-09\RMSF=7.956e-06\Dipole=0.
4371089,0.5596017,0.420246\Quadrupole=1.5997195,-1.576745,-0.0229745,0
.2367216,-1.5515867,0.5505312\PG=C01 [X(C1H4S2)]\\@
```

```
1\1\GINC-ORC278\FOpt\RwB97XD\6-311+G(d)\C1H4S2\SNIKOO\22-Jul-2017\0\\#
 opt freq wb97xd/6-311+g(d)\\CH3-SS-H\\0,1\S,-1.349661029,0.2516079562
,-0.0944623105\S,0.4871485189,-0.715329646,0.0199974208\C,1.6499070913
,0.6792642867,-0.002295784\H,1.5667144037,1.2797922303,0.9036368287\H,
2.6490516416,0.2409439692,-0.0507388399\H,1.4901650823,1.303135063,-0.
8810666038\H,-1.5606447088,0.4644571406,1.2224112887\\Version=EM64L-G0
9RevD.01\State=1-A\HF=-836.8953689\RMSD=9.692e-09\RMSF=1.032e-05\Dipol
e=0.4271815,0.5428408,0.4069666\Quadrupole=1.6222586,-1.5662434,-0.056
0153,0.2440729,-1.5520097,0.5634627\PG=C01 [X(C1H4S2)]\\@
```

```
1\1\GINC-ORC178\FOpt\RwB97XD\6-311G(d,p)\C1H4S2\SNIKOO\21-Aug-2017\0\\
# opt freq=noraman rwb97xd/6-311g(d,p)\\CH3-SS-H\\0,1\S,-1.3561367704,
0.2495460481,-0.0928149398\S,0.4810514127,-0.7205209368,0.018660782\C,
1.644931331,0.6738158883,-0.0044478462\H,1.5597592635,1.2735653239,0.9
015379055\H,2.6419579758,0.2307812798,-0.0513899475\H,1.485666175,1.29
50811493,-0.8849796361\H,-1.5553043877,0.4609692474,1.223263682\\Versi
on=EM64L-G09RevD.01\State=1-A\HF=-836.9016376\RMSD=7.365e-09\RMSF=1.25
3e-05\Dipole=0.4354447,0.551519,0.4003055\Quadrupole=1.5995726,-1.5678
586,-0.0317139,0.2570279,-1.4826054,0.5335663\PG=C01 [X(C1H4S2)]\\@
```

1\1\GINC-ORC286\FOpt\RwB97XD\6-311+G(d,p)\C1H4S2\SNIKOO\22-Jul-2017\0\
\# opt freq wb97xd/6-311+g(d,p)\\CH3-SS-H\\0,1\S,-1.3527538256,0.25257
28202,-0.0938488585\S,0.4864197465,-0.7130987759,0.0188899262\C,1.6503
371127,0.6809557064,-0.0024880902\H,1.5638052977,1.2812834458,0.903282
3457\H,2.6477126361,0.2381553147,-0.0476012714\H,1.492320488,1.3025873
343,-0.8833203284\H,-1.5551604555,0.4614151545,1.2225682765\\Version=E
M64L-G09RevD.01\State=1-A\HF=-836.9038189\RMSD=2.464e-09\RMSF=8.856e-0
6\Dipole=0.4235149,0.5355014,0.3917841\Quadrupole=1.6135048,-1.5588825
,-0.0546223,0.2551143,-1.4877331,0.5418773\PG=C01 [X(C1H4S2)]\\@

1\1\GINC-ORC169\FOpt\RwB97XD\6-311G(2d,p)\C1H4S2\SNIKOO\27-Jul-2017\0\
\# opt freq rwb97xd/6-311g(2d,p)\\CH3-SS-H\\0,1\S,-1.349682487,0.25127
07524,-0.0913096268\S,0.4801566761,-0.7035106632,0.0187706401\C,1.6505
585288,0.6844387486,-0.0030501519\H,1.562570899,1.2827630416,0.9020961
883\H,2.6439875734,0.2336821261,-0.0442493099\H,1.4967482822,1.3007344
698,-0.8865501928\H,-1.5516584726,0.4544925247,1.221774453\\Version=EM
64L-G09RevD.01\State=1-A\HF=-836.9142098\RMSD=7.025e-09\RMSF=2.597e-05
\Dipole=0.4356948,0.5143234,0.3451601\Quadrupole=1.6733458,-1.5371542,
-0.1361916,0.1559527,-1.2914764,0.483876\PG=C01 [X(C1H4S2)]\\@

1\1\GINC-ORC172\FOpt\RwB97XD\6-311G(df,p)\C1H4S2\SNIKOO\21-Aug-2017\0\
\# opt freq=noraman rwb97xd/6-311g(df,p)\\CH3-SS-H\\0,1\S,-1.351422245
9,0.2439258108,-0.0923882607\S,0.4786399499,-0.7178316241,0.0181050487
\C,1.6439085533,0.6729098282,-0.0041335982\H,1.5603920359,1.2729875191
,0.9019786142\H,2.6406403606,0.229859381,-0.0526901762\H,1.4843677041,
1.296838421,-0.8828785364\H,-1.5546013579,0.4645486641,1.2218369087\\V
ersion=EM64L-G09RevD.01\State=1-A\HF=-836.9075192\RMSD=5.199e-09\RMSF=
3.061e-05\Dipole=0.4332107,0.5515675,0.3997352\Quadrupole=1.511648,-1.
53108,0.0194321,0.3312609,-1.479144,0.5376376\PG=C01 [X(C1H4S2)]\\@

1\1\GINC-ORC272\FOpt\RwB97XD\6-311+G(df,p)\C1H4S2\SNIKOO\22-Jul-2017\0
\\# opt freq wb97xd/6-311+g(df,p)\\CH3-SS-H\\0,1\S,-1.3478548032,0.247
1866379,-0.0930604557\S,0.483798244,-0.7094582785,0.0186364043\C,1.649
4496034,0.6804908367,-0.0023984515\H,1.5649081821,1.2811312534,0.90335
18955\H,2.6460036006,0.2366025277,-0.04867614\H,1.4919102644,1.3042692
253,-0.8817923895\H,-1.5555340913,0.4636487975,1.2214211368\\Version=E
M64L-G09RevD.01\State=1-A\HF=-836.9098131\RMSD=3.530e-09\RMSF=2.640e-0
5\Dipole=0.4216146,0.5343474,0.389586\Quadrupole=1.5302789,-1.5226463,
-0.0076327,0.326089,-1.4873319,0.544664\PG=C01 [X(C1H4S2)]\\@

1\1\GINC-ORC271\FOpt\RwB97XD\6-311+G(2df,p)\C1H4S2\SNIKOO\22-Jul-2017\
0\\# opt freq wb97xd/6-311+g(2df,p)\\CH3-SS-H\\0,1\S,-1.3420194339,0.2
468716912,-0.0915526623\S,0.4775372035,-0.6985020622,0.0181554398\C,1.
6477495361,0.682612846,-0.002904261\H,1.5615313569,1.2822075933,0.9019
482829\H,2.6414150664,0.2324704577,-0.0442982507\H,1.4955856299,1.3016
573211,-0.8851903993\H,-1.5491183589,0.4565531529,1.2213238505\\Versio
n=EM64L-G09RevD.01\State=1-A\HF=-836.9220791\RMSD=7.574e-09\RMSF=5.923
e-05\Dipole=0.4167319,0.4939251,0.3390395\Quadrupole=1.5957102,-1.5170
094,-0.0787009,0.1961595,-1.3035293,0.5008053\PG=C01 [X(C1H4S2)]\\@

1\1\GINC-ORC280\FOpt\RwB97XD\6-311++G(3df,3pd)\C1H4S2\SNIKOO\27-Jul-20
17\0\\# opt freq rwb97xd/6-311++g(3df,3pd)\\CH3-SS-H\\0,1\S,-1.3390653
13,0.2456469955,-0.0891982929\S,0.4760378995,-0.6928738836,0.015893726
8\C,1.6463429539,0.6828541819,-0.0035119084\H,1.5591498319,1.282677395
,0.899210397\H,2.6374547356,0.2305684739,-0.0412140578\H,1.4996276729,

1.3008533713,-0.8856230021\H,-1.5468667809,0.4541444661,1.2219251374\\
Version=EM64L-G09RevD.01\State=1-A\HF=-836.9268404\RMSD=4.769e-09\RMSF
=1.050e-05\Dipole=0.4231836,0.477702,0.3022893\Quadrupole=1.6242034,-1
.4879552,-0.1362482,0.200504,-1.1757197,0.4650642\PG=C01 [X(C1H4S2)]\\
@

CH_3SSH M062X

1\1\GINC-ORC116\FOpt\RM062X\6-31G(d)\C1H4S2\SNIKOO\06-Jun-2017\0\\# op
t freq m062x/6-31g(d)\\CH3-SS-H (trans)\\0,1\S,-1.3254815763,-0.090763
2748,-0.6384167293\S,0.5943434935,-0.8241245248,-0.3973790254\C,1.5153
493564,0.6838938819,0.0343001673\H,1.1882590294,1.0807253954,0.9968155
865\H,2.5661376115,0.3898215218,0.1045359504\H,1.4016318711,1.43970756
23,-0.7439238083\H,-1.7364441057,-0.1499316217,0.6443497689\\Version=E
M64L-G09RevD.01\State=1-A\HF=-836.783811\RMSD=4.341e-09\RMSF=3.328e-05
\Dipole=0.2836807,0.5090692,0.558106\Quadrupole=1.7845956,-1.4433309,-
0.3412647,0.9058243,-1.0890407,0.2432521\PG=C01 [X(C1H4S2)]\\@

1\1\GINC-ORC170\FOpt\RM062X\6-31G(d,p)\C1H4S2\SNIKOO\21-Aug-2017\0\\#
opt freq=noraman m062x/6-31g(d,p)\\CH3-SS-H\\0,1\S,-1.3500105386,0.246
8434065,-0.0880135218\S,0.4758763603,-0.7243400251,0.00081139\C,1.6401
208691,0.6727375027,-0.00589586\H,1.5367482422,1.2713289854,0.89980255
12\H,2.6402133338,0.2336563406,-0.0358750026\H,1.4890810864,1.29324106
08,-0.8892895773\H,-1.5301043531,0.4697707291,1.2282900205\\Version=EM
64L-G09RevD.01\State=1-A\HF=-836.7899564\RMSD=8.355e-09\RMSF=3.572e-05
\Dipole=0.4377655,0.5475019,0.3983891\Quadrupole=1.5310722,-1.4758172,
-0.055255,0.2914524,-1.4346849,0.5631063\PG=C01 [X(C1H4S2)]\\@

1\1\GINC-ORC102\FOpt\RM062X\6-311G(d)\C1H4S2\SNIKOO\06-Jun-2017\0\\# o
pt freq m062x/6-311g(d)\\CH3-SS-H (trans)\\0,1\S,-1.3286517977,-0.0406
108052,-0.6241656073\S,0.5816938886,-0.8349550652,-0.4306824628\C,1.52
81888424,0.6449813996,0.0308278324\H,1.2293130776,1.0097780757,1.01213
54514\H,2.5755810575,0.3420756532,0.0633919514\H,1.4012265062,1.426748
5218,-0.7152970156\H,-1.7167505746,-0.1296917798,0.6640718505\\Version
=EM64L-G09RevD.01\State=1-A\HF=-836.8515801\RMSD=2.541e-09\RMSF=2.948e
-05\Dipole=0.2952024,0.4895703,0.5866452\Quadrupole=1.8055667,-1.58102
12,-0.2245454,0.8224838,-1.1494487,0.191734\PG=C01 [X(C1H4S2)]\\@
1\1\GINC-ORC98\FOpt\RM062X\6-311+G(d)\C1H4S2\SNIKOO\06-Jun-2017\0\\# o
pt freq m062x/6-311+g(d)\\CH3-SS-H (trans)\\0,1\S,-1.3262735229,-0.055
2802491,-0.6290382642\S,0.5878771688,-0.8365002017,-0.4218730971\C,1.5
277408229,0.6497279681,0.0322101709\H,1.226740419,1.0184870532,1.01158
69447\H,2.5767179861,0.3520368573,0.0666110821\H,1.3968665219,1.427500
0586,-0.717796943\H,-1.7190683958,-0.1376454865,0.6585821066\\Version=
EM64L-G09RevD.01\State=1-A\HF=-836.8541101\RMSD=2.766e-09\RMSF=2.828e-
05\Dipole=0.2851741,0.4793515,0.5646736\Quadrupole=1.8326653,-1.568244
5,-0.2644207,0.8336755,-1.143273,0.2220766\PG=C01 [X(C1H4S2)]\\@

1\1\GINC-ORC318\FOpt\RM062X\6-311G(d,p)\C1H4S2\SNIKOO\21-Aug-2017\0\\#
opt freq=noraman m062x/6-311g(d,p)\\CH3-SS-H\\0,1\S,-1.3455233567,0.2
518663449,-0.0943900454\S,0.4829002564,-0.7326908842,0.0066500803\C,1.
6368233958,0.67027307,-0.0031087096\H,1.5333940106,1.2644310237,0.9033
357175\H,2.6385302003,0.239981114,-0.0420515847\H,1.4731089759,1.29080
17686,-0.8820155071\H,-1.5173084824,0.4785755629,1.221410049\\Version=
EM64L-G09RevD.01\State=1-A\HF=-836.8589353\RMSD=9.464e-09\RMSF=2.817e-
05\Dipole=0.4337568,0.5498438,0.4014315\Quadrupole=1.5265236,-1.534078
,0.0075545,0.2279688,-1.4197917,0.5824525\PG=C01 [X(C1H4S2)]\\@

1\1\GINC-ORC278\FOpt\RM062X\6-311+G(d,p)\C1H4S2\SNIKOO\18-Jul-2017\0\\
opt freq 6-311+g(d,p) geom=connectivity m062x\\CH3-SS-H\\0,1\S,-1.35
57025975,0.2391974666,-0.0864978575\S,0.4893399664,-0.713459435,0.0139
507483\C,1.6232138,0.705398849,-0.0084407841\H,1.5170757436,1.30164480
98,0.896617411\H,2.6307910518,0.2891555493,-0.0520870355\H,1.444758119
7,1.3206238629,-0.8885710968\H,-1.5260840839,0.4721208973,1.2285496147
\\Version=EM64L-G09RevD.01\State=1-A\HF=-836.8612897\RMSD=6.465e-09\RM
SF=5.113e-05\Dipole=0.4155448,0.5416231,0.3861753\Quadrupole=1.5240236
,-1.5094942,-0.0145294,0.2575083,-1.4426371,0.5836425\PG=C01 [X(C1H4S2
)]\\@

1\1\GINC-ORC142\FOpt\RM062X\6-311G(2d,p)\C1H4S2\SNIKOO\26-Jul-2017\0\\
opt freq m062x/6-311g(2d,p)\\CH3-SS-H\\0,1\S,-1.3517192253,0.2417961
696,-0.0846636072\S,0.4830845224,-0.7024060436,0.0183049293\C,1.625196
2374,0.7095091581,-0.0090058603\H,1.5201305145,1.3047735494,0.89488143
34\H,2.6281900889,0.2837602051,-0.0513780978\H,1.44850853,1.3177175001
,-0.8923978144\H,-1.5299986679,0.4595314613,1.2277800169\\Version=EM64
L-G09RevD.01\State=1-A\HF=-836.8710439\RMSD=9.758e-09\RMSF=2.258e-05\D
ipole=0.4261555,0.5181295,0.3376819\Quadrupole=1.5934518,-1.4978006,-0
.0956511,0.1570966,-1.249816,0.5044873\PG=C01 [X(C1H4S2)]\\@

1\1\GINC-ORC172\FOpt\RM062X\6-311G(df,p)\C1H4S2\SNIKOO\21-Aug-2017\0\\
opt freq=noraman m062x/6-311g(df,p)\\CH3-SS-H\\0,1\S,-1.3409970896,0
.2439407221,-0.0949598934\S,0.4805689693,-0.7299929142,0.0079316815\C,
1.6356986375,0.6692564156,-0.0023172935\H,1.5363891382,1.2631954627,0.
9050435254\H,2.6371741219,0.2391382832,-0.0457379735\H,1.469867931,1.2
935493978,-0.8784620172\H,-1.5167767083,0.4841506329,1.2183319707\\Ver
sion=EM64L-G09RevD.01\State=1-A\HF=-836.8651808\RMSD=9.275e-09\RMSF=3.
141e-05\Dipole=0.4321896,0.551858,0.3999165\Quadrupole=1.4334532,-1.49
41907,0.0607376,0.3108941,-1.415128,0.5868457\PG=C01 [X(C1H4S2)]\\@

1\1\GINC-ORC276\FOpt\RM062X\6-311+G(df,p)\C1H4S2\SNIKOO\18-Jul-2017\0\
\# opt freq 6-311+g(df,p) geom=connectivity m062x\\CH3-SS-H\\0,1\S,-1.
350895362,0.2319608971,-0.0865238459\S,0.4866994839,-0.7100581355,0.01
49789798\C,1.6224325612,0.7046125636,-0.0079678537\H,1.5204669411,1.30
12004904,0.8976407227\H,2.6294826445,0.2877298944,-0.0551663666\H,1.44
29926307,1.3232150983,-0.8858096608\H,-1.5277868993,0.4760211916,1.226
3690245\\Version=EM64L-G09RevD.01\State=1-A\HF=-836.867665\RMSD=5.997e
-09\RMSF=2.811e-05\Dipole=0.4144331,0.5419301,0.3832644\Quadrupole=1.4
352132,-1.4686555,0.0334423,0.3360207,-1.4433665,0.5858279\PG=C01 [X(C
1H4S2)]\\@

1\1\GINC-ORC342\FOpt\RM062X\6-311+G(2df,p)\C1H4S2\SNIKOO\18-Jul-2017\0
\\# opt freq m062x/6-311+g(2df,p) geom=connectivity\\CH3-SS-H\\0,1\S,-
1.3447376579,0.2330379019,-0.086929083\S,0.4807523778,-0.6977059749,0.
0212028361\C,1.6229953342,0.7077278602,-0.0079494901\H,1.5245618416,1.
3034079341,0.8971462319\H,2.626080377,0.282235488,-0.0562734267\H,1.44
39732054,1.3207841288,-0.8883002225\H,-1.5302334781,0.4651946619,1.224
6241541\\Version=EM64L-G09RevD.01\State=1-A\HF=-836.8791976\RMSD=7.349
e-09\RMSF=3.424e-05\Dipole=0.4061403,0.4973021,0.3280327\Quadrupole=1.
5225985,-1.4704852,-0.0521132,0.2024984,-1.2595832,0.527887\PG=C01 [X(
C1H4S2)]\\@

1\1\GINC-ORC139\FOpt\RM062X\6-311++G(3df,3pd)\C1H4S2\SNIKOO\26-Jul-201
7\0\\# opt freq m062x/6-311++g(3df,3pd)\\CH3-SS-H\\0,1\S,-1.3427358336
,0.2344239325,-0.0820194962\S,0.4785642351,-0.6922153327,0.0159581951\
C,1.6220180277,0.7083342438,-0.0099268204\H,1.5171763211,1.3055796538,

0.8910920978\H,2.6225793284,0.2806491492,-0.0480752773\H,1.4526564518,
1.3182701871,-0.8920596794\H,-1.5268665307,0.4596401663,1.2285519803\\
Version=EM64L-G09RevD.01\State=1-A\HF=-836.8845455\RMSD=5.117e-09\RMSF
=1.970e-05\Dipole=0.4106798,0.4768552,0.2913527\Quadrupole=1.5589996,-
1.4419795,-0.1170201,0.2073534,-1.1316929,0.4837479\PG=C01 [X(C1H4S2)]
\\@

CH_3SSH M08HX

1\1\GINC-ORC176\FOpt\RM08HX\6-31G(d)\C1H4S2\SNIKOO\25-Jul-2017\0\\# op
t freq 6-31g(d) m08hx\\CH3-SS-H\\0,1\S,-1.3381968641,0.2558544826,-0.0
931556447\S,0.4805497354,-0.7244606897,0.0044348591\C,1.6403290828,0.6
746814368,-0.0027290355\H,1.5383924631,1.2789183383,0.9059171557\H,2.6
499999717,0.2451191591,-0.0348311757\H,1.486981758,1.3013837185,-0.888
0270237\H,-1.525375147,0.4723745544,1.2258728648\\Version=EM64L-G16Rev
A.03\State=1-A\HF=-836.7929372\RMSD=6.197e-09\RMSF=2.018e-05\Dipole=0.
4326432,0.5402859,0.4070372\Quadrupole=1.5226873,-1.4719422,-0.0507451
,0.2659705,-1.4620415,0.5637995\PG=C01 [X(C1H4S2)]\\@

1\1\GINC-ORC61\FOpt\RM08HX\6-31G(d,p)\C1H4S2\SNIKOO\21-Aug-2017\0\\# o
pt freq=noraman m08hx/6-31g(d,p)\\CH3-SS-H\\0,1\S,-1.3441870613,0.2603
133584,-0.0889008469\S,0.4730121773,-0.7254797793,0.0025927134\C,1.637
6149621,0.6691550432,-0.0063385305\H,1.5341735753,1.2763596535,0.89893
25466\H,2.6439299345,0.2339771591,-0.0329325881\H,1.4894928182,1.29080
14929,-0.8948722425\H,-1.5321114061,0.4581110722,1.2313489479\\Version
=EM64L-G16RevA.03\State=1-A\HF=-836.7991589\RMSD=2.993e-09\RMSF=1.902e
-05\Dipole=0.4324876,0.5361449,0.4046067\Quadrupole=1.5374534,-1.49999
44,-0.037459,0.2872036,-1.4666387,0.5339249\PG=C01 [X(C1H4S2)]\\@

1\1\GINC-ORC175\FOpt\RM08HX\6-311G(d)\C1H4S2\SNIKOO\25-Jul-2017\0\\# o
pt freq 6-311g(d) m08hx\\CH3-SS-H\\0,1\S,-1.331817054,0.2739902279,-0.
0943998132\S,0.4859319279,-0.7275478089,0.0070565049\C,1.6405993644,0.
6735550643,-0.002915618\H,1.5345492029,1.2793942055,0.9008037496\H,2.6
492922165,0.2484436679,-0.0335633379\H,1.4842650808,1.2940753681,-0.88
84915454\H,-1.5301397384,0.4619602753,1.22899206\\Version=EM64L-G16Rev
A.03\State=1-A\HF=-836.8675548\RMSD=4.538e-09\RMSF=2.049e-05\Dipole=0.
42252,0.5348067,0.4254274\Quadrupole=1.5337571,-1.5705407,0.0367836,0.
2192287,-1.526203,0.5329693\PG=C01 [X(C1H4S2)]\\@

1\1\GINC-ORC5\FOpt\RM08HX\6-311+G(d)\C1H4S2\SNIKOO\25-Jul-2017\0\\# op
t freq 6-311+g(d) m08hx\\CH3-SS-H\\0,1\S,-1.3329634708,0.2737165403,-0
.0947405271\S,0.4853635749,-0.7261527787,0.0086210044\C,1.6415250119,0
.6732565342,-0.003070272\H,1.5370176655,1.28000589,0.9006383154\H,2.65
03727887,0.2479023614,-0.0345564078\H,1.4847065972,1.2946052607,-0.888
4622876\H,-1.5333411674,0.4605371921,1.2290521747\\Version=EM64L-G16Re
vA.03\State=1-A\HF=-836.869985\RMSD=6.344e-09\RMSF=2.043e-05\Dipole=0.
4113318,0.5147741,0.4110839\Quadrupole=1.5541247,-1.5676888,0.0135641,
0.2279247,-1.532544,0.5466422\PG=C01 [X(C1H4S2)]\\@

1\1\GINC-ORC10\FOpt\RM08HX\6-311G(d,p)\C1H4S2\SNIKOO\21-Aug-2017\0\\#
opt freq=noraman 6-311g(d,p) m08hx\\CH3-SS-H\\0,1\S,-1.3395192973,0.27
20873388,-0.0922494718\S,0.4788918557,-0.7307398779,0.0062867178\C,1.6
368551657,0.6679848885,-0.005282565\H,1.5295395459,1.2740704096,0.8979
545591\H,2.6431506957,0.2372916,-0.0344645745\H,1.4809805739,1.2863881
613,-0.8924147398\H,-1.5279735397,0.4561554797,1.2300000742\\Version=E
M64L-G16RevA.03\State=1-A\HF=-836.875064\RMSD=6.917e-09\RMSF=2.030e-05

\Dipole=0.4200605,0.5274543,0.4078034\Quadrupole=1.5373885,-1.5643342,
0.0269458,0.2385549,-1.4667384,0.5155277\PG=C01 [X(C1H4S2)]\\@

1\1\GINC-ORC176\FOpt\RM08HX\6-311+G(d,p)\C1H4S2\SNIKOO\25-Jul-2017\0\\
opt freq 6-311+g(d,p) m08hx\\CH3-SS-H\\0,1\S,-1.3356065832,0.2752587
23,-0.0937342415\S,0.4845122384,-0.7237443853,0.0077111467\C,1.6421924
693,0.6749198492,-0.0032849988\H,1.5338588882,1.2814857873,0.899928448
4\H,2.6491392783,0.2451732848,-0.0313643401\H,1.4865484122,1.293772152
,-0.8905869735\H,-1.5279637033,0.4570055889,1.2288129589\\Version=EM64
L-G16RevA.03\State=1-A\HF=-836.8772977\RMSD=7.577e-09\RMSF=2.478e-05\D
ipole=0.4066263,0.5081821,0.3976677\Quadrupole=1.5477439,-1.5611599,0.
013416,0.2364914,-1.4760063,0.5249041\PG=C01 [X(C1H4S2)]\\@

1\1\GINC-ORC220\FOpt\RM08HX\6-311G(2d,p)\C1H4S2\ROOT\01-Aug-2017\0\\#
opt freq 6-311g(2d,p) m08hx\\CH3-SS-H\\0,1\S,-1.3491659621,0.239873079
6,-0.0840645798\S,0.485410482,-0.70657398,0.0127104388\C,1.6189037138,
0.7102060163,-0.0051572754\H,1.4983708739,1.3149369639,0.8955233606\H,
2.6305116091,0.2933864395,-0.0301118775\H,1.4529917519,1.3169771685,-0
.8967580181\H,-1.5409724686,0.4180153122,1.2344059513\\Version=EM64L-G
16RevA.03\State=1-A\HF=-836.8867887\RMSD=3.648e-09\RMSF=2.473e-05\Dipo
le=0.4092011,0.5023169,0.3492627\Quadrupole=1.590323,-1.5170613,-0.073
2616,0.1889104,-1.2939421,0.4440167\PG=C01 [X(C1H4S2)]\\@

1\1\GINC-ORC6\FOpt\RM08HX\6-311G(df,p)\C1H4S2\SNIKOO\21-Aug-2017\0\\#
opt freq=noraman 6-311g(df,p) m08hx\\CH3-SS-H\\0,1\S,-1.335807254,0.26
52238584,-0.0920463182\S,0.4761504238,-0.7279104583,0.0052306221\C,1.6
354444091,0.6669726377,-0.0050047385\H,1.5300011007,1.273556511,0.8984
373561\H,2.641624173,0.2362998617,-0.0352301993\H,1.4802656596,1.28858
29945,-0.8903049717\H,-1.5257535122,0.460512595,1.2287482495\\Version=
EM64L-G16RevA.03\State=1-A\HF=-836.8814019\RMSD=9.098e-09\RMSF=2.246e-
05\Dipole=0.4178214,0.5284164,0.4083297\Quadrupole=1.4423352,-1.527276
2,0.0849409,0.3187416,-1.4606005,0.5233331\PG=C01 [X(C1H4S2)]\\@

1\1\GINC-ORC36\FOpt\RM08HX\6-311+G(df,p)\C1H4S2\SNIKOO\25-Jul-2017\0\\
opt freq 6-311+g(df,p) m08hx\\CH3-SS-H\\0,1\S,-1.3316795037,0.269376
726,-0.0930026764\S,0.481472464,-0.7198250844,0.0071866096\C,1.6412576
1,0.6742475682,-0.0032609126\H,1.5355008417,1.2819165681,0.899813714\H
,2.6475926276,0.2433128118,-0.032147809\H,1.4872111319,1.2958984749,-0
.8891896148\H,-1.5286741715,0.4589439354,1.2280826892\\Version=EM64L-G
16RevA.03\State=1-A\HF=-836.8837466\RMSD=4.576e-09\RMSF=2.737e-05\Dipo
le=0.4043596,0.5075292,0.3966492\Quadrupole=1.4618051,-1.5259846,0.064
1795,0.3135848,-1.477074,0.5285644\PG=C01 [X(C1H4S2)]\\@

1\1\GINC-ORC31\FOpt\RM08HX\6-311+G(2df,p)\C1H4S2\SNIKOO\25-Jul-2017\0\
\# opt freq 6-311+g(2df,p) m08hx\\CH3-SS-H\\0,1\S,-1.3279390943,0.2674
17618,-0.0908666238\S,0.4756198987,-0.707748049,0.0054546925\C,1.64014
97417,0.6770491867,-0.0039255789\H,1.5325720675,1.2834232907,0.8981932
195\H,2.6427055498,0.2379919448,-0.0271622402\H,1.4927104032,1.2935597
837,-0.893006544\H,-1.5231375665,0.452177225,1.2287950749\\Version=EM6
4L-G16RevA.03\State=1-A\HF=-836.8951284\RMSD=6.134e-09\RMSF=2.724e-05\
Dipole=0.3961202,0.4684416,0.3454237\Quadrupole=1.5193812,-1.5171505,-
0.0022306,0.1681781,-1.2967581,0.4876199\PG=C01 [X(C1H4S2)]\\@

1\1\GINC-ORC219\FOpt\RM08HX\6-311++G(3df,3pd)\C1H4S2\SNIKOO\01-Aug-201
7\0\\# opt freq 6-311++g(3df,3pd) m08hx\\CH3-SS-H\\0,1\S,-1.340085392,
0.2351951207,-0.0805167405\S,0.4802529352,-0.6972630845,0.005123177\C,
1.6151682023,0.7091195707,-0.0064732796\H,1.4890678905,1.3149193118,0.

```
8905764806\H,2.6238995923,0.2906668565,-0.022109016\H,1.4593738217,1.3
162894885,-0.8975317353\H,-1.53162705,0.4178937362,1.2374791139\\Versi
on=EM64L-G16RevA.03\State=1-A\HF=-836.9014962\RMSD=8.290e-09\RMSF=2.75
3e-05\Dipole=0.4001168,0.4673822,0.3070861\Quadrupole=1.5507769,-1.482
8154,-0.0679615,0.2382514,-1.1813073,0.4350668\PG=C01 [X(C1H4S2)]\\@
```

CH_3SSH MP2

```
1\1\GINC-ORC279\FOpt\RMP2-FC\6-31G(d)\C1H4S2\SNIKOO\20-Jul-2017\0\\# o
pt freq rmp2/6-31g(d)\\CH3-SS-H\\0,1\S,-1.3340823684,0.2545796822,-0.0
891182135\S,0.487249227,-0.7092055414,0.0134352275\C,1.6491211902,0.68
16059021,-0.0026042002\H,1.5559515105,1.288023832,0.8996129932\H,2.651
5006499,0.246401304,-0.0374941755\H,1.4979208614,1.3038818137,-0.88531
28004\H,-1.5510270706,0.4627020075,1.2224311689\\Version=EM64L-G09RevD
.01\State=1-A\HF=-835.2109864\MP2=-835.5823096\RMSD=8.544e-09\RMSF=1.1
21e-05\Dipole=0.4180249,0.5497593,0.4134725\PG=C01 [X(C1H4S2)]\\@
```

```
1\1\GINC-ORC318\FOpt\RMP2-FC\6-31G(d,p)\C1H4S2\SNIKOO\21-Aug-2017\0\\#
 opt freq=noraman rmp2/6-31g(d,p)\\CH3-SS-H\\0,1\S,-1.3424949781,0.254
0622401,-0.0840193188\S,0.4754405377,-0.7171047632,0.0109140161\C,1.63
88166316,0.6698563322,-0.0056815101\H,1.5486725047,1.2732632261,0.8937
259821\H,2.6362265067,0.2342216131,-0.041975926\H,1.4890530487,1.29127
24966,-0.8840136033\H,-1.5437892512,0.4576668551,1.22088036\\Version=E
M64L-G09RevD.01\State=1-A\HF=-835.2199299\MP2=-835.61722\RMSD=2.686e-0
9\RMSF=7.733e-06\Dipole=0.4039425,0.5270727,0.3876616\PG=C01 [X(C1H4S2
)]\\@
```

```
1\1\GINC-ORC269\FOpt\RMP2-FC\6-311G(d)\C1H4S2\SNIKOO\20-Jul-2017\0\\#
opt freq rmp2/6-311g(d) geom=connectivity\\CH3-SS-H\\0,1\S,-1.32504949
44,0.2665536207,-0.0917389312\S,0.4958700917,-0.7183661149,0.013885632
8\C,1.6444643777,0.6773752727,-0.0018758725\H,1.5454569184,1.283118043
6,0.8986834157\H,2.6506896584,0.254208267,-0.0373300137\H,1.4873050197
,1.2985845396,-0.8826277586\H,-1.5421025716,0.4665153714,1.2219535276\
\Version=EM64L-G09RevD.01\State=1-A\HF=-835.2629875\MP2=-835.6536208\R
MSD=6.157e-09\RMSF=9.675e-06\Dipole=0.4047316,0.546577,0.4313818\PG=C0
1 [X(C1H4S2)]\\@
```

```
1\1\GINC-ORC208\FOpt\RMP2-FC\6-311+G(d)\C1H4S2\SNIKOO\20-Jul-2017\0\\#
 opt freq rmp2/6-311+g(d) geom=connectivity\\CH3-SS-H\\0,1\S,-1.325078
4595,0.2524776974,-0.1005726993\S,0.4991407997,-0.7253760367,0.0204182
179\C,1.6418814929,0.6746303814,0.0029420547\H,1.5551056898,1.27200332
94,0.9108590617\H,2.6503414651,0.2586587114,-0.0530619838\H,1.46621443
75,1.304191997,-0.868853079\H,-1.5309714254,0.4914029201,1.2092184278\
\Version=EM64L-G09RevD.01\State=1-A\HF=-835.2657617\MP2=-835.6582406\R
MSD=9.843e-09\RMSF=3.995e-05\Dipole=0.3965237,0.5421697,0.4108153\PG=C
01 [X(C1H4S2)]\\@
```

```
1\1\GINC-ORC61\FOpt\RMP2-FC\6-311G(d,p)\C1H4S2\SNIKOO\21-Aug-2017\0\\#
 opt freq=noraman rmp2/6-311g(d,p)\\CH3-SS-H\\0,1\S,-1.3351043124,0.26
57435338,-0.088090512\S,0.4819678407,-0.7282698146,0.0067620915\C,1.63
1193519,0.6640391286,-0.0046748726\H,1.5299527317,1.2704325315,0.89652
09032\H,2.6379329253,0.2388543695,-0.0372118777\H,1.47818678,1.2886421
268,-0.8852622675\H,-1.5222044843,0.4637961244,1.2217865351\\Version=E
M64L-G09RevD.01\State=1-A\HF=-835.2731832\MP2=-835.6915841\RMSD=7.132e
-09\RMSF=4.439e-05\Dipole=0.3886067,0.5100022,0.3857745\PG=C01 [X(C1H4
S2)]\\@
```

```
1\1\GINC-ORC196\FOpt\RMP2-FC\6-311+G(d,p)\C1H4S2\SNIKOO\20-Jul-2017\0\
 \# opt freq rmp2/6-311+g(d,p) geom=connectivity\\CH3-SS-H\\0,1\S,-1.32
 65328345,0.2554047501,-0.0977164325\S,0.4972792274,-0.7265671641,0.013
 51738\C,1.6362874014,0.6738325074,0.0027519549\H,1.5399398005,1.273520
 2133,0.9093212285\H,2.6466140994,0.2583379082,-0.0429400619\H,1.468439
 5503,1.303471184,-0.8718739947\H,-1.5053932446,0.4899896011,1.20788992
 56\\Version=EM64L-G09RevD.01\State=1-A\HF=-835.2757276\MP2=-835.695924
 1\RMSD=4.180e-09\RMSF=4.644e-05\Dipole=0.3774597,0.506349,0.3712403\PG
 =C01 [X(C1H4S2)]\\@

1\1\GINC-ORC288\FOpt\RMP2-FC\6-311G(2d,p)\C1H4S2\SNIKOO\27-Jul-2017\0\
 \# opt freq rmp2/6-311g(2d,p)\\CH3-SS-H\\0,1\S,-1.3391184364,0.2703264
 695,-0.0895882408\S,0.4906197454,-0.7172643657,0.01251999\C,1.64878785
 57,0.6840599067,-0.0022394886\H,1.5423954951,1.285904418,0.8984338246\
 H,2.6503480735,0.2502357486,-0.0308085768\H,1.4945264834,1.2968919429,
 -0.8879809915\H,-1.5309252168,0.45783488,1.2206134832\\Version=EM64L-G
 09RevD.01\State=1-A\HF=-835.2852126\MP2=-835.7401947\RMSD=3.962e-09\RM
 SF=9.847e-06\Dipole=0.4093167,0.4994602,0.351595\PG=C01 [X(C1H4S2)]\\@

1\1\GINC-ORC300\FOpt\RMP2-FC\6-311G(df,p)\C1H4S2\SNIKOO\21-Aug-2017\0\
 \# opt freq=noraman rmp2/6-311g(df,p)\\CH3-SS-H\\0,1\S,-1.3205228058,0
 .2534448113,-0.0889327922\S,0.4756529927,-0.7176610825,0.0076636709\C,
 1.6275658315,0.6635470685,-0.0038629236\H,1.5319279126,1.269844867,0.8
 985792903\H,2.632334546,0.2339024699,-0.0401632869\H,1.4748175427,1.29
 16431518,-0.8826063512\H,-1.5198510198,0.468516714,1.2191523927\\Versi
 on=EM64L-G09RevD.01\State=1-A\HF=-835.279956\MP2=-835.7615349\RMSD=3.8
 30e-09\RMSF=1.383e-05\Dipole=0.3901212,0.5242883,0.3945344\PG=C01 [X(C
 1H4S2)]\\@

1\1\GINC-ORC187\FOpt\RMP2-FC\6-311+G(df,p)\C1H4S2\SNIKOO\20-Jul-2017\0
 \\# opt freq rmp2/6-311+g(df,p) geom=connectivity\\CH3-SS-H\\0,1\S,-1.
 3110141352,0.243675302,-0.0981559973\S,0.4904990395,-0.7158268225,0.01
 46994484\C,1.6328387945,0.6730652914,0.0033874325\H,1.5420137457,1.273
 5071435,0.9107665522\H,2.6413509269,0.2533347495,-0.0453548883\H,1.465
 6753912,1.306222642,-0.8696311626\H,-1.5047297627,0.4940106941,1.20523
 86151\\Version=EM64L-G09RevD.01\State=1-A\HF=-835.2826635\MP2=-835.765
 793\RMSD=6.387e-09\RMSF=4.416e-05\Dipole=0.379174,0.5190701,0.3781159\
 PG=C01 [X(C1H4S2)]\\@

1\1\GINC-ORC185\FOpt\RMP2-FC\6-311+G(2df,p)\C1H4S2\SNIKOO\20-Jul-2017\
 0\\# opt freq rmp2/6-311+g(2df,p) geom=connectivity\\CH3-SS-H\\0,1\S,-
 1.3219054454,0.2584643369,-0.0952772911\S,0.4852882219,-0.7086159585,0
 .0144459344\C,1.6425081637,0.6818981317,-0.0005649221\H,1.5415206045,1
 .2827797618,0.9025467415\H,2.644266995,0.2474984871,-0.0345894578\H,1.
 4854384267,1.2994281074,-0.8837331435\H,-1.5204829663,0.4665361336,1.2
 181221386\\Version=EM64L-G09RevD.01\State=1-A\HF=-835.295176\MP2=-835.
 8079884\RMSD=6.576e-09\RMSF=2.265e-05\Dipole=0.3916182,0.4881754,0.346
 3\PG=C01 [X(C1H4S2)]\\@

1\1\GINC-ORC170\FOpt\RMP2-FC\6-311++G(3df,3pd)\C1H4S2\SNIKOO\27-Jul-20
 17\0\\# opt freq rmp2/6-311++g(3df,3pd)\\CH3-SS-H\\0,1\S,-1.3167344321
 ,0.2598173672,-0.0910937704\S,0.4837968695,-0.6980332072,0.0122105184\
 C,1.6412739422,0.6832178904,-0.0012454176\H,1.5366845846,1.2832087605,
 0.8982856441\H,2.637362175,0.2430508829,-0.029774429\H,1.4898222813,1.
 29673281,-0.8845351145\H,-1.5155714205,0.4599944962,1.217102569\\Versi
 on=EM64L-G09RevD.01\State=1-A\HF=-835.2996323\MP2=-835.8309477\RMSD=5.
 914e-09\RMSF=8.857e-06\Dipole=0.4026811,0.4657377,0.2987534\PG=C01 [X(
```

```
 C1H4S2)]\\@
```

CH_3SSH QCISD

```
1\1\GINC-ORC22\FOpt\RQCISD-FC\6-311+G(2df,p)\C1H4S2\SNIKOO\19-Aug-2017
 \0\\# opt freq=noraman 6-311+g(2df,p) qcisd\\CH3-SS-H\\0,1\S,-1.338557
 3961,0.2497618432,-0.0888494705\S,0.4878740422,-0.7098777196,0.0066568
 893\C,1.6424789094,0.6909125109,-0.0029224152\H,1.536813329,1.29065539
 29,0.9019245629\H,2.6476474073,0.2616253026,-0.0352634952\H,1.48562504
 24,1.3112310898,-0.8855862904\H,-1.5352053342,0.4430535802,1.229799219
 1\\Version=EM64L-G09RevD.01\State=1-A\HF=-835.2950564\MP2=-835.8079057
 \MP3=-835.8529533\MP4D=-835.8672475\MP4DQ=-835.8521303\MP4SDQ=-835.855
 5843\QCISD=-835.8555002\RMSD=5.352e-09\RMSF=2.859e-05\Dipole=0.3886664
 ,0.4759813,0.3351925\PG=C01 [X(C1H4S2)]\\@
```

CH_3SS^- B3LYP

```
1\1\GINC-ORC189\FOpt\RB3LYP\6-31G(d)\C1H3S2(1-)\SNIKOO\05-Jun-2017\0\\
 # opt freq b3lyp/6-31g(d)\\CH3-SS(-)\\-1,1\S,0.0189907071,1.2318455592
 ,-0.0061241003\S,0.0106047519,-0.8844189373,-0.0743299868\C,1.26089732
 76,-1.3469909221,1.1895973236\H,0.9706088345,-0.9640104696,2.175285775
 4\H,1.333259032,-2.4438699555,1.232590144\H,2.2412078324,-0.928950065,
 0.9312248869\\Version=EM64L-G09RevD.01\State=1-A'\HF=-836.327744\RMSD=
 7.151e-09\RMSF=1.090e-04\Dipole=0.6910354,-1.4566183,0.6647258\Quadrup
 ole=1.4794312,-2.9936544,1.5142231,0.5373227,0.1875473,0.4274805\PG=CS
  [SG(C1H1S2),X(H2)]\\@
```

```
1\1\GINC-ORC300\FOpt\RB3LYP\6-31G(d,p)\C1H3S2(1-)\SNIKOO\21-Aug-2017\0
 \\# opt freq=noraman rb3lyp/6-31g(d,p)\\CH3-SS(-)\\-1,1\S,1.0647365961
 ,-0.995120494,0.\S,0.0071416037,0.839120377,0.\C,-1.750556862,0.307351
 8415,0.\H,-1.9680839921,-0.2948698062,0.8886960536\H,-2.3903553537,1.2
 006258878,0.\H,-1.9680839921,-0.2948698062,-0.8886960536\\Version=EM64
 L-G09RevD.01\State=1-A'\HF=-836.3320551\RMSD=3.254e-09\RMSF=6.935e-05\
 Dipole=-1.571238,0.7427288,0.\Quadrupole=-0.2206133,-1.1037943,1.32440
 77,2.3933292,0.,0.\PG=CS [SG(C1H1S2),X(H2)]\\@
```

```
1\1\GINC-ORC248\FOpt\RB3LYP\6-311G(d)\C1H3S2(1-)\SNIKOO\05-Jun-2017\0\
 \# opt freq b3lyp/6-311g(d)\\CH3-SS(-)\\-1,1\S,1.0423036562,-1.0039658
 839,0.\S,-0.0090578767,0.8536090756,0.\C,-1.7572811789,0.3088904038,0.
 \H,-1.9660754108,-0.2938396186,0.8876072808\H,-2.400082779,1.197556641
 8,0.\H,-1.9660754108,-0.2938396186,-0.8876072808\\Version=EM64L-G09Rev
 D.01\State=1-A'\HF=-836.4008468\RMSD=5.262e-09\RMSF=4.042e-05\Dipole=-
 1.6765554,0.8515435,0.\Quadrupole=-0.2060664,-1.0743137,1.2803801,2.34
 85291,0.,0.\PG=CS [SG(C1H1S2),X(H2)]\\@
```

```
1\1\GINC-ORC194\FOpt\RB3LYP\6-311+G(d)\C1H3S2(1-)\SNIKOO\06-Jun-2017\0
 \\# opt freq b3lyp/6-311+g(d)\\CH3-SS(-)\\-1,1\S,1.0441425195,-0.99954
 04045,0.\S,-0.0077948469,0.850302363,0.\C,-1.7572228492,0.3073209565,0
 .\H,-1.9695746014,-0.2943584479,0.8878970258\H,-2.3962446205,1.1990449
 807,0.\H,-1.9695746014,-0.2943584479,-0.8878970258\\Version=EM64L-G09R
 evD.01\State=1-A'\HF=-836.4039239\RMSD=8.386e-09\RMSF=3.525e-05\Dipole
 =-1.6388211,0.8722239,0.\Quadrupole=-0.2373005,-1.142094,1.3793945,2.4
 157204,0.,0.\PG=CS [SG(C1H1S2),X(H2)]\\@
```

```
1\1\GINC-ORC318\FOpt\RB3LYP\6-311G(d,p)\C1H3S2(1-)\SNIKOO\21-Aug-2017\
 0\\# opt freq=noraman rb3lyp/6-311g(d,p)\\CH3-SS(-)\\-1,1\S,1.05396439
 3,-1.0039768211,0.\S,-0.0010315813,0.8512431999,0.\C,-1.7509791289,0.3
```

073314372,0.\H,-1.9584118679,-0.2953846482,0.8883180952\H,-2.390331947
,1.1984094802,0.\H,-1.9584118679,-0.2953846482,-0.8883180952\\Version=
EM64L-G09RevD.01\State=1-A'\HF=-836.4052756\RMSD=4.662e-09\RMSF=6.212e
-05\Dipole=-1.6669037,0.8504411,0.\Quadrupole=-0.2453945,-1.0505472,1.
2959417,2.3465785,0.,0.\PG=CS [SG(C1H1S2),X(H2)]\\@

1\1\GINC-ORC291\FOpt\RB3LYP\6-311+G(d,p)\C1H3S2(1-)\SNIKOO\18-Jul-2017
\0\\# opt freq rb3lyp/6-311+g(d,p)\\CH3-SS(-)\\-1,1\S,1.0521003586,-1.
0016866686,0.\S,-0.0008237357,0.8477887342,0.\C,-1.7512161435,0.306594
5327,0.\H,-1.962359515,-0.2948557382,0.888602326\H,-2.3876334494,1.200
2198781,0.\H,-1.962359515,-0.2948557382,-0.888602326\\Version=EM64L-G0
9RevD.01\State=1-A'\HF=-836.4083287\RMSD=4.795e-09\RMSF=1.254e-04\Dipo
le=-1.6248485,0.8729378,0.\Quadrupole=-0.2969609,-1.1070737,1.4040346,
2.4121293,0.,0.\PG=CS [SG(C1H1S2),X(H2)]\\@

1\1\GINC-ORC231\FOpt\RB3LYP\6-311G(2d,p)\C1H3S2(1-)\SNIKOO\26-Jul-2017
\0\\# opt freq rb3lyp/6-311g(2d,p)\\CH3-SS(-)\\-1,1\S,1.0476439911,-0.
9961391772,0.\S,0.000196602,0.8408896141,0.\C,-1.7518628653,0.30761526
31,0.\H,-1.961492251,-0.2913953433,0.8888003208\H,-2.3846342257,1.2023
949865,0.\H,-1.961492251,-0.2913953433,-0.8888003208\\Version=EM64L-G0
9RevD.01\State=1-A'\HF=-836.4141522\RMSD=2.651e-09\RMSF=4.907e-05\Dipo
le=-1.5870153,0.7937896,0.\Quadrupole=-0.2653097,-0.9023177,1.1676274,
2.2208717,0.,0.\PG=CS [SG(C1H1S2),X(H2)]\\@

1\1\GINC-ORC300\FOpt\RB3LYP\6-311G(df,p)\C1H3S2(1-)\SNIKOO\21-Aug-2017
\0\\# opt freq=noraman rb3lyp/6-311g(df,p)\\CH3-SS(-)\\-1,1\S,1.049327
9343,-0.9970005541,0.\S,0.0009968433,0.845357337,0.\C,-1.7494187807,0.
3072773535,0.\H,-1.9588853368,-0.2959059793,0.8876753687\H,-2.38833732
33,1.1984158222,0.\H,-1.9588853368,-0.2959059793,-0.8876753687\\Versio
n=EM64L-G09RevD.01\State=1-A'\HF=-836.4109316\RMSD=4.135e-09\RMSF=5.56
3e-05\Dipole=-1.6540457,0.8443427,0.\Quadrupole=-0.2789932,-1.1462822,
1.4252754,2.4102993,0.,0.\PG=CS [SG(C1H1S2),X(H2)]\\@

1\1\GINC-ORC290\FOpt\RB3LYP\6-311+G(df,p)\C1H3S2(1-)\SNIKOO\18-Jul-201
7\0\\# opt freq rb3lyp/6-311+g(df,p) geom=connectivity\\CH3-SS(-)\\-1,
1\S,1.0485602371,-0.9940094983,0.\S,0.0010647733,0.8412967108,0.\C,-1.
7503886224,0.3061309987,0.\H,-1.9635591346,-0.2955829652,0.8880137376\
H,-2.3844101189,1.2009527192,0.\H,-1.9635591346,-0.2955829652,-0.88801
37376\\Version=EM64L-G09RevD.01\State=1-A'\HF=-836.4140207\RMSD=7.814e
-09\RMSF=2.548e-05\Dipole=-1.6088809,0.8662282,0.\Quadrupole=-0.334201
2,-1.187204,1.5214052,2.469396,0.,0.\PG=CS [SG(C1H1S2),X(H2)]\\@

1\1\GINC-ORC291\FOpt\RB3LYP\6-311+G(2df,p)\C1H3S2(1-)\SNIKOO\18-Jul-20
17\0\\# opt freq rb3lyp/6-311+g(2df,p) geom=connectivity\\CH3-SS(-)\\-
1,1\S,1.0420657283,-0.9824601933,0.\S,-0.0001508377,0.8313435113,0.\C,
-1.7489602959,0.3033564433,0.\H,-1.9638676207,-0.2952786222,0.88866534
2\H,-2.3775113533,1.2015224829,0.\H,-1.9638676207,-0.2952786222,-0.888
665342\\Version=EM64L-G09RevD.01\State=1-A'\HF=-836.4228039\RMSD=6.200
e-09\RMSF=2.421e-05\Dipole=-1.5365071,0.8061606,0.\Quadrupole=-0.38327
9,-0.9991762,1.3824552,2.3212549,0.,0.\PG=CS [SG(C1H1S2),X(H2)]\\@

1\1\GINC-ORC207\FOpt\RB3LYP\6-311++G(3df,3pd)\C1H3S2(1-)\ROOT\26-Jul-2
017\0\\# opt freq rb3lyp/6-311++g(3df,3pd)\\CH3-SS(-)\\-1,1\S,1.037693
0199,-0.9774736515,0.\S,-0.0019326343,0.8272914082,0.\C,-1.7473858921,
0.3041664722,0.\H,-1.9646460016,-0.2928497741,0.8875825043\H,-2.370723
4903,1.2036853193,0.\H,-1.9646460016,-0.2928497741,-0.8875825043\\Vers
ion=EM64L-G09RevD.01\State=1-A'\HF=-836.4264006\RMSD=7.104e-09\RMSF=6.

234e-06\Dipole=-1.452654,0.7650741,0.\Quadrupole=-0.4043096,-0.9876266
,1.3919362,2.2377233,0.,0.\PG=CS [SG(C1H1S2),X(H2)]\\@

CH_3SS^- B3PW91

1\1\GINC-ORC152\FOpt\RB3PW91\6-31G(d)\C1H3S2(1-)\SNIKOO\20-Jul-2017\0\
\# opt freq rb3pw91/6-31g(d)\\CH3-SS(-)\\-1,1\S,1.00013391,-1.01629300
42,0.\S,0.0390483676,0.846314529,0.\C,-1.729679401,0.399278585,0.\H,-1
.9766780366,-0.1935341197,0.889352739\H,-2.3297628035,1.3212561295,0.\
H,-1.9766780366,-0.1935341197,-0.889352739\\Version=EM64L-G09RevD.01\S
tate=1-A'\HF=-836.2133574\RMSD=3.949e-09\RMSF=5.197e-05\Dipole=-1.5671
599,0.8180851,0.\Quadrupole=0.0765605,-1.3570673,1.2805069,2.3037525,0
.,0.\PG=CS [SG(C1H1S2),X(H2)]\\@

1\1\GINC-ORC61\FOpt\RB3PW91\6-31G(d,p)\C1H3S2(1-)\SNIKOO\21-Aug-2017\0
\\# opt freq=noraman rb3pw91/6-31g(d,p)\\CH3-SS(-)\\-1,1\S,1.046725249
7,-0.9843576086,0.\S,0.0019524872,0.8326784943,0.\C,-1.7440943095,0.30
64927985,0.\H,-1.962849453,-0.296146713,0.8887453223\H,-2.3840865213,1
.1997177417,0.\H,-1.962849453,-0.296146713,-0.8887453223\\Version=EM64
L-G09RevD.01\State=1-A'\HF=-836.2174111\RMSD=1.923e-09\RMSF=1.533e-05\
Dipole=-1.5954139,0.7457343,0.\Quadrupole=-0.1619644,-1.1326508,1.2946
151,2.3595715,0.,0.\PG=CS [SG(C1H1S2),X(H2)]\\@

1\1\GINC-ORC150\FOpt\RB3PW91\6-311G(d)\C1H3S2(1-)\SNIKOO\20-Jul-2017\0
\\# opt freq rb3pw91/6-311g(d) geom=connectivity\\CH3-SS(-)\\-1,1\S,0.
9813113456,-1.0244422614,0.\S,0.0324381367,0.8604688547,0.\C,-1.727444
9991,0.399975383,0.\H,-1.9637938388,-0.1942057389,0.8878794028\H,-2.33
23328055,1.3158975015,0.\H,-1.9637938388,-0.1942057389,-0.8878794028\\
Version=EM64L-G09RevD.01\State=1-A'\HF=-836.2800364\RMSD=6.781e-09\RMS
F=8.285e-05\Dipole=-1.646039,0.9339675,0.\Quadrupole=0.060608,-1.31829
93,1.2576913,2.2814822,0.,0.\PG=CS [SG(C1H1S2),X(H2)]\\@

1\1\GINC-ORC147\FOpt\RB3PW91\6-311+G(d)\C1H3S2(1-)\SNIKOO\20-Jul-2017\
0\\# opt freq rb3pw91/6-311+g(d) geom=connectivity\\CH3-SS(-)\\-1,1\S,
0.9847432663,-1.0208710549,0.\S,0.03322142,0.857122016,0.\C,-1.7275590
309,0.398674549,0.\H,-1.9675943086,-0.1943770183,0.8880814389\H,-2.328
8330382,1.3173165266,0.\H,-1.9675943086,-0.1943770183,-0.8880814389\\V
ersion=EM64L-G09RevD.01\State=1-A'\HF=-836.2829469\RMSD=5.852e-09\RMSF
=7.768e-05\Dipole=-1.6055218,0.9443133,0.\Quadrupole=0.0530593,-1.3909
928,1.3379335,2.3272351,0.,0.\PG=CS [SG(C1H1S2),X(H2)]\\@

1\1\GINC-ORC318\FOpt\RB3PW91\6-311G(d,p)\C1H3S2(1-)\SNIKOO\21-Aug-2017
\0\\# opt freq=noraman rb3pw91/6-311g(d,p)\\CH3-SS(-)\\-1,1\S,1.031266
0532,-0.9926394736,0.\S,-0.0057428272,0.8454260566,0.\C,-1.743678055,0
.3064924043,0.\H,-1.951122967,-0.2970252748,0.8886767217\H,-2.38480123
7,1.1970095623,0.\H,-1.951122967,-0.2970252748,-0.8886767217\\Version=
EM64L-G09RevD.01\State=1-A'\HF=-836.2845033\RMSD=1.754e-09\RMSF=9.289e
-05\Dipole=-1.6769451,0.8581896,0.\Quadrupole=-0.1906917,-1.0858182,1.
2765099,2.3314279,0.,0.\PG=CS [SG(C1H1S2),X(H2)]\\@

1\1\GINC-ORC94\FOpt\RB3PW91\6-311+G(d,p)\C1H3S2(1-)\SNIKOO\20-Jul-2017
\0\\# opt freq rb3pw91/6-311+g(d,p) geom=connectivity\\CH3-SS(-)\\-1,1
\S,0.9870360071,-1.020304329,0.\S,0.0322516193,0.8558568892,0.\C,-1.72
98033837,0.3981497381,0.\H,-1.9679508974,-0.1946150203,0.8888201138\H,
-2.3271984477,1.3190157424,0.\H,-1.9679508974,-0.1946150203,-0.8888201
138\\Version=EM64L-G09RevD.01\State=1-A'\HF=-836.2873658\RMSD=8.958e-0

9\RMSF=7.956e-05\Dipole=-1.5925405,0.9429275,0.\Quadrupole=-0.0148224,
-1.3492326,1.3640549,2.3302838,0.,0.\PG=CS [SG(C1H1S2),X(H2)]\\@

1\1\GINC-ORC280\FOpt\RB3PW91\6-311G(2d,p)\C1H3S2(1-)\SNIKOO\27-Jul-201
7\0\\# opt freq rb3pw91/6-311g(2d,p)\\CH3-SS(-)\\-1,1\S,0.9851832419,-
1.013290754,0.\S,0.031716776,0.8480813292,0.\C,-1.7305340085,0.3966894
935,0.\H,-1.9678054943,-0.1934566121,0.8894001688\H,-2.3243710209,1.31
89211554,0.\H,-1.9678054943,-0.1934566121,-0.8894001688\\Version=EM64L
-G09RevD.01\State=1-A'\HF=-836.2944684\RMSD=7.548e-09\RMSF=4.190e-05\D
ipole=-1.5573881,0.8595351,0.\Quadrupole=-0.0299899,-1.1094577,1.13944
76,2.1585656,0.,0.\PG=CS [SG(C1H1S2),X(H2)]\\@

1\1\GINC-ORC170\FOpt\RB3PW91\6-311G(df,p)\C1H3S2(1-)\SNIKOO\21-Aug-201
7\0\\# opt freq=noraman rb3pw91/6-311g(df,p)\\CH3-SS(-)\\-1,1\S,1.0277
370434,-0.9863918377,0.\S,-0.0036200373,0.8399898425,0.\C,-1.741767024
4,0.3068472557,0.\H,-1.9521192426,-0.2975360088,0.8877740102\H,-2.3833
134966,1.1968647572,0.\H,-1.9521192426,-0.2975360088,-0.8877740102\\Ve
rsion=EM64L-G09RevD.01\State=1-A'\HF=-836.2904036\RMSD=9.811e-09\RMSF=
1.310e-05\Dipole=-1.6638489,0.8510318,0.\Quadrupole=-0.221246,-1.18615
02,1.4073962,2.3969338,0.,0.\PG=CS [SG(C1H1S2),X(H2)]\\@

1\1\GINC-ORC222\FOpt\RB3PW91\6-311+G(df,p)\C1H3S2(1-)\ROOT\20-Jul-2017
\0\\# opt freq rb3pw91/6-311+g(df,p) geom=connectivity\\CH3-SS(-)\\-1,
1\S,0.9850278561,-1.0133028557,0.\S,0.0335428421,0.8494644665,0.\C,-1.
7284725796,0.3979121639,0.\H,-1.9697085784,-0.1950577314,0.8881194432\
H,-2.324296962,1.3195296881,0.\H,-1.9697085784,-0.1950577314,-0.888119
4432\\Version=EM64L-G09RevD.01\State=1-A'\HF=-836.2933182\RMSD=8.009e-
09\RMSF=5.388e-05\Dipole=-1.5775028,0.934392,0.\Quadrupole=-0.0435529,
-1.4408669,1.4844198,2.3870951,0.,0.\PG=CS [SG(C1H1S2),X(H2)]\\@

1\1\GINC-ORC90\FOpt\RB3PW91\6-311+G(2df,p)\C1H3S2(1-)\SNIKOO\20-Jul-20
17\0\\# opt freq rb3pw91/6-311+g(2df,p) geom=connectivity\\CH3-SS(-)\\
-1,1\S,0.9798979582,-1.0016318166,0.\S,0.0315902172,0.8394912437,0.\C,
-1.7275240443,0.3947313859,0.\H,-1.9703798732,-0.1946431776,0.88916478
28\H,-2.3168203847,1.3201835421,0.\H,-1.9703798732,-0.1946431776,-0.88
91647828\\Version=EM64L-G09RevD.01\State=1-A'\HF=-836.3030526\RMSD=7.5
68e-09\RMSF=3.155e-05\Dipole=-1.5034979,0.8636663,0.\Quadrupole=-0.111
1484,-1.2281391,1.3392875,2.2421215,0.,0.\PG=CS [SG(C1H1S2),X(H2)]\\@

1\1\GINC-ORC170\FOpt\RB3PW91\6-311++G(3df,3pd)\C1H3S2(1-)\SNIKOO\27-Ju
l-2017\0\\# opt freq rb3pw91/6-311++g(3df,3pd)\\CH3-SS(-)\\-1,1\S,0.97
67766835,-0.9964436166,0.\S,0.0287997499,0.8357076517,0.\C,-1.72628776
96,0.393628614,0.\H,-1.9705047869,-0.1944762319,0.8882626682\H,-2.3118
950899,1.3195478148,0.\H,-1.9705047869,-0.1944762319,-0.8882626682\\Ve
rsion=EM64L-G09RevD.01\State=1-A'\HF=-836.3062906\RMSD=5.443e-09\RMSF=
2.583e-05\Dipole=-1.4246417,0.8126552,0.\Quadrupole=-0.1476967,-1.2011
6,1.3488567,2.1530162,0.,0.\PG=CS [SG(C1H1S2),X(H2)]\\@

CH_3SS^- wB97XD

1\1\GINC-ORC285\FOpt\RwB97XD\6-31G(d)\C1H3S2(1-)\SNIKOO\22-Jul-2017\0\
\# opt freq rwb97xd/6-31g(d)\\CH3-SS(-)\\-1,1\S,1.040659934,-0.9819383
584,0.\S,-0.0027589432,0.8350133683,0.\C,-1.7436515014,0.3005062212,0.
\H,-1.9596208182,-0.3026961096,0.888980177\H,-2.3870728529,1.190860988
1,0.\H,-1.9596208182,-0.3026961096,-0.888980177\\Version=EM64L-G09RevD
.01\State=1-A'\HF=-836.2743071\RMSD=3.003e-09\RMSF=7.210e-05\Dipole=-1
.6414103,0.760118,0.\Quadrupole=-0.0833206,-1.2161471,1.2994677,2.3808

392,0.,0.\PG=CS [SG(C1H1S2),X(H2)]\\@

1\1\GINC-ORC43\FOpt\RwB97XD\6-31G(d,p)\C1H3S2(1-)\SNIKOO\21-Aug-2017\0
\\# opt freq=noraman wb97xd/6-31g(d,p)\\CH3-SS(-)\\-1,1\S,1.0364065783
,-0.9849589039,0.\S,-0.0005585982,0.8360272751,0.\C,-1.7420592405,0.30
64176986,0.\H,-1.9575773069,-0.2960484151,0.8883527612\H,-2.3838361258
,1.1968487604,0.\H,-1.9575773069,-0.2960484151,-0.8883527612\\Version=
EM64L-G09RevD.01\State=1-A'\HF=-836.2783413\RMSD=3.114e-09\RMSF=9.645e
-06\Dipole=-1.6287686,0.7643587,0.\Quadrupole=-0.0961911,-1.2178397,1.
3140308,2.3769117,0.,0.\PG=CS [SG(C1H1S2),X(H2)]\\@

1\1\GINC-ORC286\FOpt\RwB97XD\6-311G(d)\C1H3S2(1-)\SNIKOO\22-Jul-2017\0
\\# opt freq rwb97xd/6-311g(d)\\CH3-SS(-)\\-1,1\S,1.0231053712,-0.9913
35105,0.\S,-0.009646478,0.8483689385,0.\C,-1.74139321,0.3016704405,0.\
H,-1.9469000117,-0.30245498,0.8875729133\H,-2.3903306598,1.185255686,0
.\H,-1.9469000117,-0.30245498,-0.8875729133\\Version=EM64L-G09RevD.01\
State=1-A'\HF=-836.3411108\RMSD=9.059e-09\RMSF=2.839e-05\Dipole=-1.737
3321,0.877498,0.\Quadrupole=-0.0868923,-1.1743098,1.2612021,2.3484601,
0.,0.\PG=CS [SG(C1H1S2),X(H2)]\\@

1\1\GINC-ORC282\FOpt\RwB97XD\6-311+G(d)\C1H3S2(1-)\SNIKOO\22-Jul-2017\
0\\# opt freq rwb97xd/6-311+g(d)\\CH3-SS(-)\\-1,1\S,1.026251784,-0.987
8216033,0.\S,-0.0090159568,0.8452030739,0.\C,-1.7412419245,0.300504791
7,0.\H,-1.9504829594,-0.3027459078,0.8877624335\H,-2.3870929838,1.1866
555533,0.\H,-1.9504829594,-0.3027459078,-0.8877624335\\Version=EM64L-G
09RevD.01\State=1-A'\HF=-836.3440436\RMSD=5.352e-09\RMSF=2.193e-05\Dip
ole=-1.7127177,0.8973116,0.\Quadrupole=-0.0262539,-1.2595089,1.2857628
,2.3822889,0.,0.\PG=CS [SG(C1H1S2),X(H2)]\\@

1\1\GINC-ORC300\FOpt\RwB97XD\6-311G(d,p)\C1H3S2(1-)\SNIKOO\21-Aug-2017
\0\\# opt freq=noraman rwb97xd/6-311g(d,p)\\CH3-SS(-)\\-1,1\S,1.022399
7947,-0.9932228795,0.\S,-0.0074985794,0.8481124048,0.\C,-1.7419638792,
0.3065822406,0.\H,-1.9465045621,-0.2967096978,0.8883260794\H,-2.385130
212,1.1941856296,0.\H,-1.9465045621,-0.2967096978,-0.8883260794\\Versi
on=EM64L-G09RevD.01\State=1-A'\HF=-836.3454151\RMSD=4.624e-09\RMSF=1.1
71e-04\Dipole=-1.7222832,0.8816044,0.\Quadrupole=-0.1142458,-1.1644817
,1.2787275,2.3421965,0.,0.\PG=CS [SG(C1H1S2),X(H2)]\\@

1\1\GINC-ORC281\FOpt\RwB97XD\6-311+G(d,p)\C1H3S2(1-)\SNIKOO\22-Jul-201
7\0\\# opt freq rwb97xd/6-311+g(d,p)\\CH3-SS(-)\\-1,1\S,1.0276034947,-
0.9873350242,0.\S,-0.0098510388,0.8442568655,0.\C,-1.7430084623,0.3000
547343,0.\H,-1.9504677675,-0.3030127283,0.8883522691\H,-2.3858734588,1
.188098881,0.\H,-1.9504677675,-0.3030127283,-0.8883522691\\Version=EM6
4L-G09RevD.01\State=1-A'\HF=-836.3482889\RMSD=7.487e-09\RMSF=2.082e-05
\Dipole=-1.6981852,0.8966783,0.\Quadrupole=-0.0891758,-1.2204062,1.309
582,2.3805639,0.,0.\PG=CS [SG(C1H1S2),X(H2)]\\@

\1\GINC-ORC307\FOpt\RwB97XD\6-311G(2d,p)\C1H3S2(1-)\SNIKOO\27-Jul-201
7\0\\# opt freq rwb97xd/6-311g(2d,p)\\CH3-SS(-)\\-1,1\S,1.0243506012,-
0.9813083889,0.\S,-0.0095217815,0.837645914,0.\C,-1.7435592355,0.29885
84151,0.\H,-1.9496445227,-0.3020148819,0.8888194165\H,-2.3840455386,1.
1878838235,0.\H,-1.9496445227,-0.3020148819,-0.8888194165\\Version=EM6
4L-G09RevD.01\State=1-A'\HF=-836.3553125\RMSD=8.858e-09\RMSF=6.535e-05
\Dipole=-1.6414512,0.8094075,0.\Quadrupole=-0.1653409,-0.9654388,1.130
7797,2.206168,0.,0.\PG=CS [SG(C1H1S2),X(H2)]\\@

1\1\GINC-ORC318\FOpt\RwB97XD\6-311G(df,p)\C1H3S2(1-)\SNIKOO\21-Aug-201
7\0\\# opt freq=noraman rwb97xd/6-311g(df,p)\\CH3-SS(-)\\-1,1\S,1.0190
141618,-0.98816662,0.\S,-0.0062317424,0.8436864739,0.\C,-1.7404696818,
0.306870536,0.\H,-1.9468285775,-0.2971409895,0.8876010202\H,-2.3838575
826,1.1941295892,0.\H,-1.9468285775,-0.2971409895,-0.8876010202\\Versi
on=EM64L-G09RevD.01\State=1-A'\HF=-836.3513614\RMSD=3.203e-09\RMSF=1.2
82e-04\Dipole=-1.7096452,0.8728154,0.\Quadrupole=-0.1454867,-1.265773,
1.4112597,2.4062496,0.,0.\PG=CS [SG(C1H1S2),X(H2)]\\@

1\1\GINC-ORC281\FOpt\RwB97XD\6-311+G(df,p)\C1H3S2(1-)\SNIKOO\22-Jul-20
17\0\\# opt freq rwb97xd/6-311+g(df,p)\\CH3-SS(-)\\-1,1\S,1.0266805265
,-0.9810757146,0.\S,-0.0085586243,0.8381734902,0.\C,-1.7420689153,0.29
98586182,0.\H,-1.9524011895,-0.3034279433,0.8877859931\H,-2.3833156078
,1.1889494927,0.\H,-1.9524011895,-0.3034279433,-0.8877859931\\Version=
EM64L-G09RevD.01\State=1-A'\HF=-836.3542936\RMSD=3.068e-09\RMSF=2.621e
-05\Dipole=-1.6834494,0.8866265,0.\Quadrupole=-0.1244198,-1.3073536,1.
4317734,2.4393261,0.,0.\PG=CS [SG(C1H1S2),X(H2)]\\@

1\1\GINC-ORC279\FOpt\RwB97XD\6-311+G(2df,p)\C1H3S2(1-)\SNIKOO\22-Jul-2
017\0\\# opt freq rwb97xd/6-311+g(2df,p)\\CH3-SS(-)\\-1,1\S,1.01905342
44,-0.9708767907,0.\S,-0.0094723288,0.8297958458,0.\C,-1.7406251718,0.
2972607773,0.\H,-1.9517278134,-0.3030708501,0.8886392684\H,-2.37756529
71,1.1890118678,0.\H,-1.9517278134,-0.3030708501,-0.8886392684\\Versio
n=EM64L-G09RevD.01\State=1-A'\HF=-836.3638991\RMSD=5.095e-09\RMSF=2.69
4e-05\Dipole=-1.6051997,0.8248522,0.\Quadrupole=-0.1768661,-1.10318,1.
280046,2.2765373,0.,0.\PG=CS [SG(C1H1S2),X(H2)]\\@

1\1\GINC-ORC170\FOpt\RwB97XD\6-311++G(3df,3pd)\C1H3S2(1-)\SNIKOO\27-Ju
l-2017\0\\# opt freq rwb97xd/6-311++g(3df,3pd)\\CH3-SS(-)\\-1,1\S,1.01
81495441,-0.9658302393,0.\S,-0.0120040583,0.8252304967,0.\C,-1.7397931
604,0.2960890795,0.\H,-1.9530304213,-0.3026129986,0.8875992206\H,-2.37
23564828,1.1887866604,0.\H,-1.9530304213,-0.3026129986,-0.8875992206\\
Version=EM64L-G09RevD.01\State=1-A'\HF=-836.3671957\RMSD=3.211e-09\RMS
F=9.103e-06\Dipole=-1.5355109,0.7749515,0.\Quadrupole=-0.2083239,-1.07
50196,1.2833435,2.1843638,0.,0.\PG=CS [SG(C1H1S2),X(H2)]\\@

CH_3SS^- M062X

1\1\GINC-ORC104\FOpt\RM062X\6-31G(d)\C1H3S2(1-)\SNIKOO\06-Jun-2017\0\\
opt freq m062x/6-31g(d)\\CH3-SS(-)\\-1,1\S,1.0020757988,-0.988478492
4,0.\S,-0.0080406624,0.8478444191,0.\C,-1.7460343278,0.3100088614,0.\H
,-1.9527437297,-0.2966124118,0.8874300315\H,-2.3987823492,1.1922610355
,0.\H,-1.9527437297,-0.2966124118,-0.8874300315\\Version=EM64L-G09RevD
.01\State=1-A'\HF=-836.2278544\RMSD=4.490e-09\RMSF=4.721e-05\Dipole=-1
.5740869,0.7564625,0.\Quadrupole=-0.0621456,-1.2362687,1.2984143,2.332
2633,0.,0.\PG=CS [SG(C1H1S2),X(H2)]\\@

1\1\GINC-ORC6\FOpt\RM062X\6-31G(d,p)\C1H3S2(1-)\SNIKOO\21-Aug-2017\0\\
opt freq=noraman m062x/6-31g(d,p)\\CH3-SS\\-1,1\S,-1.3655156248,0.27
88300993,-0.0350258112\S,0.473067602,-0.7287349601,-0.0299780332\C,1.6
557913745,0.6542217752,-0.0146564444\H,1.4995041343,1.271048624,0.8752
246807\H,2.6771459831,0.254776333,-0.0099061992\H,1.5119505309,1.28141
41286,-0.8994281927\\Version=EM64L-G09RevD.01\State=1-A'\HF=-836.231292
5\RMSD=1.647e-09\RMSF=6.091e-05\Dipole=1.7306572,0.2024064,0.0117519\Q
uadrupole=-2.5275817,1.2130164,1.3145653,1.5072581,-0.0142888,0.008240
6\PG=CS [SG(C1H1S2),X(H2)]\\@

```
1\1\GINC-ORC100\FOpt\RM062X\6-311G(d)\C1H3S2(1-)\SNIKOO\06-Jun-2017\0\
 \# opt freq m062x/6-311g(d)\\CH3-SS(-)\\-1,1\S,0.9944063467,-0.9988213
 453,0.\S,-0.0057442252,0.8588582301,0.\C,-1.7368528526,0.3103254745,0.
 \H,-1.9330863389,-0.2964686558,0.8860193868\H,-2.3909685911,1.18805995
 24,0.\H,-1.9330863389,-0.2964686558,-0.8860193868\\Version=EM64L-G09Re
 vD.01\State=1-A'\HF=-836.3062399\RMSD=4.364e-09\RMSF=4.585e-05\Dipole=
 -1.6833484,0.8736433,0.\Quadrupole=-0.0266292,-1.2225006,1.2491298,2.2
 917681,0.,0.\PG=CS [SG(C1H1S2),X(H2)]\\@
```

```
1\1\GINC-ORC356\FOpt\RM062X\6-311+G(d)\C1H3S2(1-)\SNIKOO\06-Jun-2017\0
 \\# opt freq m062x/6-311+g(d)\\CH3-SS(-)\\-1,1\S,0.9914009413,-0.99271
 07701,0.\S,-0.0141334358,0.8561581732,0.\C,-1.7457224823,0.3088763418,
 0.\H,-1.9457802237,-0.2967508266,0.8864051544\H,-2.3962535757,1.189588
 9082,0.\H,-1.9457802237,-0.2967508266,-0.8864051544\\Version=EM64L-G09
 RevD.01\State=1-A'\HF=-836.3091316\RMSD=5.043e-09\RMSF=3.224e-05\Dipol
 e=-1.6453397,0.884579,0.\Quadrupole=-0.0064018,-1.2902392,1.2966409,2.
 3169911,0.,0.\PG=CS [SG(C1H1S2),X(H2)]\\@
```

```
1\1\GINC-ORC61\FOpt\RM062X\6-311G(d,p)\C1H3S2(1-)\SNIKOO\21-Aug-2017\0
 \\# opt freq=noraman m062x/6-311g(d,p)\\CH3-SS\\-1,1\S,-1.3549010861,0
 .295821255,-0.0354400074\S,0.4854509591,-0.736051412,-0.0296067626\C,1
 .6563598534,0.653576087,-0.0144654259\H,1.4882946493,1.2643876975,0.87
 45865483\H,2.677158442,0.2592804218,-0.0103292198\H,1.4995811824,1.274
 5419507,-0.8985151325\\Version=EM64L-G09RevD.01\State=1-A'\HF=-836.3095
 241\RMSD=2.109e-09\RMSF=4.451e-05\Dipole=1.8819643,0.1587682,0.012769\
 Quadrupole=-2.4535245,1.1876096,1.2659149,1.4942275,-0.0152757,0.00878
 79\PG=CS [SG(C1H1S2),X(H2)]\\@
```

```
1\1\GINC-ORC294\FOpt\RM062X\6-311+G(d,p)\C1H3S2(1-)\SNIKOO\18-Jul-2017
 \0\\# opt freq 6-311+g(d,p) m062x\\CH3-SS(-)\\-1,1\S,1.0349561334,-0.9
 829758064,0.\S,-0.0009322335,0.8491044472,0.\C,-1.7240459022,0.2753323
 372,0.\H,-1.9127067008,-0.3334386332,0.8869188103\H,-2.3866675961,1.14
 69572883,0.\H,-1.9127067008,-0.3334386332,-0.8869188103\\Version=EM64L
 -G09RevD.01\State=1-A'\HF=-836.3123872\RMSD=3.523e-09\RMSF=1.112e-04\D
 ipole=-1.6487578,0.8583004,0.\Quadrupole=-0.130186,-1.1871436,1.317329
 6,2.3319138,0.,0.\PG=CS [SG(C1H1S2),X(H2)]\\@
```

```
1\1\GINC-ORC231\FOpt\RM062X\6-311G(2d,p)\C1H3S2(1-)\SNIKOO\26-Jul-2017
 \0\\# opt freq m062x/6-311g(2d,p)\\CH3-SS(-)\\-1,1\S,1.0320201336,-0.9
 775397974,0.\S,-0.0012173389,0.8426757528,0.\C,-1.7255858346,0.2739760
 181,0.\H,-1.9112930013,-0.3322405287,0.887545982\H,-2.3847339574,1.146
 910084,0.\H,-1.9112930013,-0.3322405287,-0.887545982\\Version=EM64L-G0
 9RevD.01\State=1-A'\HF=-836.3190619\RMSD=7.156e-09\RMSF=5.472e-05\Dipo
 le=-1.6090619,0.7797683,0.\Quadrupole=-0.1963845,-0.9305507,1.1269351,
 2.1663278,0.,0.\PG=CS [SG(C1H1S2),X(H2)]\\@
```

```
1\1\GINC-ORC10\FOpt\RM062X\6-311G(df,p)\C1H3S2(1-)\SNIKOO\21-Aug-2017\
 0\\# opt freq=noraman m062x/6-311g(df,p)\\CH3-SS\\-1,1\S,-1.3505621014
 ,0.2904588068,-0.0347980685\S,0.4801053637,-0.7314566049,-0.0301666476
 \C,1.6553664082,0.6527693449,-0.0145830782\H,1.4896316949,1.266171891,
 0.8737317046\H,2.6757066593,0.2573105176,-0.0099493583\H,1.5016959753,
 1.2763020446,-0.8980045521\\Version=EM64L-G09RevD.01\State=1-A'\HF=-836
 .3159235\RMSD=5.377e-09\RMSF=4.788e-05\Dipole=1.8667294,0.1611777,0.01
 22207\Quadrupole=-2.5778283,1.159364,1.4184643,1.5547673,-0.0144333,0.
 0078076\PG= CS [X(C1H1S2)]\\@
```

1\1\GINC-ORC286\FOpt\RM062X\6-311+G(df,p)\C1H3S2(1-)\SNIKOO\18-Jul-201
7\0\\# opt freq m062x/6-311+g(df,p) geom=connectivity\\CH3-SS(-)\\-1,1
\S,1.0335754374,-0.9750742354,0.\S,0.0011609535,0.841596577,0.\C,-1.72
32853147,0.2752802113,0.\H,-1.9149364469,-0.3343020127,0.8862022084\H,
-2.3836811825,1.1483424726,0.\H,-1.9149364469,-0.3343020127,-0.8862022
084\\Version=EM64L-G09RevD.01\State=1-A'\HF=-836.3188706\RMSD=5.781e-0
9\RMSF=1.267e-04\Dipole=-1.6323741,0.8474048,0.\Quadrupole=-0.1739335,
-1.2843324,1.4582659,2.3933264,0.,0.\PG=CS [SG(C1H1S2),X(H2)]\\@

1\1\GINC-ORC285\FOpt\RM062X\6-311+G(2df,p)\C1H3S2(1-)\SNIKOO\18-Jul-20
17\0\\# opt freq m062x/6-311+g(2df,p) geom=connectivity\\CH3-SS(-)\\-1
,1\S,1.0302961013,-0.9658340142,0.\S,-0.0003381674,0.8328635102,0.\C,-
1.7230381501,0.2723012382,0.\H,-1.9156934298,-0.3335523367,0.887406258
8\H,-2.3776359242,1.1493149392,0.\H,-1.9156934298,-0.3335523367,-0.887
4062588\\Version=EM64L-G09RevD.01\State=1-A'\HF=-836.3277918\RMSD=7.63
8e-09\RMSF=2.525e-05\Dipole=-1.5560794,0.7866148,0.\Quadrupole=-0.2400
009,-1.0681203,1.3081211,2.2345696,0.,0.\PG=CS [SG(C1H1S2),X(H2)]\\@

1\1\GINC-ORC144\FOpt\RM062X\6-311++G(3df,3pd)\C1H3S2(1-)\SNIKOO\26-Jul
-2017\0\\# opt freq m062x/6-311++g(3df,3pd)\\CH3-SS(-)\\-1,1\S,1.02777
18331,-0.9610863467,0.\S,-0.0023446692,0.8289131337,0.\C,-1.7221397748
,0.2712421625,0.\H,-1.9161670489,-0.3330236602,0.8861720313\H,-2.37305
62912,1.1485193709,0.\H,-1.9161670489,-0.3330236602,-0.8861720313\\Ver
sion=EM64L-G09RevD.01\State=1-A'\HF=-836.3317088\RMSD=7.292e-09\RMSF=3
.055e-05\Dipole=-1.4802375,0.7374075,0.\Quadrupole=-0.2566318,-1.03251
23,1.2891441,2.1355499,0.,0.\PG=CS [SG(C1H1S2),X(H2)]\\@

CH_3SS^- M08HX

1\1\GINC-ORC36\FOpt\RM08HX\6-31G(d)\C1H3S2(1-)\SNIKOO\25-Jul-2017\0\\#
opt freq 6-31g(d) m08hx\\CH3-SS(-)\\-1,1\S,1.0041114505,-0.9875286768
,0.\S,-0.0014454008,0.8496375516,0.\C,-1.7347244037,0.3048704073,0.\H,
-1.9409683344,-0.3066822989,0.8904405932\H,-2.3980699772,1.1854353157,
0.\H,-1.9409683344,-0.3066822989,-0.8904405932\\Version=EM64L-G16RevA.
03\State=1-A'\HF=-836.2355781\RMSD=5.585e-09\RMSF=1.307e-04\Dipole=-1.
5668211,0.7582807,0.\Quadrupole=-0.0670372,-1.2307761,1.2978133,2.3327
813,0.,0.\PG=CS [SG(C1H1S2),X(H2)]\\@

1\1\GINC-ORC300\FOpt\RM08HX\6-31G(d,p)\C1H3S2(1-)\SNIKOO\21-Aug-2017\0
\\# opt freq=noraman 6-31g(d,p) m08hx\\CH3-SS\\-1,1\S,-1.3575704862,0.
2855987774,-0.0348950807\S,0.4735377083,-0.7335029985,-0.0297946864\C,
1.6525758824,0.649720662,-0.0146981929\H,1.4948941951,1.2712056037,0.8
776345742\H,2.6815044796,0.2573331671,-0.0099416891\H,1.5070022208,1.2
812007883,-0.9020749251\\Version=EM64L-G16RevA.03\State=1-A'\HF=-836.23
9112\RMSD=7.309e-09\RMSF=6.977e-05\Dipole=1.7265241,0.1917455,0.011594
4\Quadrupole=-2.5134235,1.2044323,1.3089912,1.5223564,-0.0142823,0.008
4403\PG=CS [SG(C1H1S2),X(H2)]\\@

1\1\GINC-ORC31\FOpt\RM08HX\6-311G(d)\C1H3S2(1-)\SNIKOO\25-Jul-2017\0\\
opt freq 6-311g(d) m08hx\\CH3-SS(-)\\-1,1\S,0.9844983731,-0.99701415
66,0.\S,-0.0069765536,0.8636454643,0.\C,-1.7338240411,0.3064551203,0.\
H,-1.9269561589,-0.3068887252,0.888389833\H,-2.4018504607,1.1797410224
,0.\H,-1.9269561589,-0.3068887252,-0.888389833\\Version=EM64L-G16RevA.
03\State=1-A'\HF=-836.3193931\RMSD=4.009e-09\RMSF=1.823e-05\Dipole=-1.
653064,0.8753352,0.\Quadrupole=-0.0468551,-1.2147668,1.2616218,2.29898

5,0.,0.\PG=CS [SG(C1H1S2),X(H2)]\\@

1\1\GINC-ORC176\FOpt\RM08HX\6-311+G(d)\C1H3S2(1-)\SNIKOO\25-Jul-2017\0
\\# opt freq 6-311+g(d) m08hx\\CH3-SS(-)\\-1,1\S,0.9885612492,-0.99269
7111,0.\S,-0.0062264592,0.8598737942,0.\C,-1.7338465047,0.3047730876,0
.\H,-1.9314219402,-0.3073522384,0.8887289445\H,-2.3977094051,1.1818047
059,0.\H,-1.9314219402,-0.3073522384,-0.8887289445\\Version=EM64L-G16R
evA.03\State=1-A'\HF=-836.3225195\RMSD=9.888e-09\RMSF=6.939e-05\Dipole
=-1.5884691,0.8827304,0.\Quadrupole=-0.0815553,-1.2698572,1.3514126,2.
3074857,0.,0.\PG=CS [SG(C1H1S2),X(H2)]\\@

1\1\GINC-ORC170\FOpt\RM08HX\6-311G(d,p)\C1H3S2(1-)\SNIKOO\21-Aug-2017\
0\\# opt freq=noraman 6-311g(d,p) m08hx\\CH3-SS\\-1,1\S,-1.3460542268,
0.3023049574,-0.0351738461\S,0.4856502993,-0.7412132159,-0.0293017074\
C,1.6528248022,0.6491655642,-0.0145634806\H,1.4836823491,1.2654156265,
0.8764760461\H,2.6809992235,0.2609530608,-0.0101984219\H,1.4948415528,
1.274930007,-0.9010085901\\Version=EM64L-G16RevA.03\State=1-A'\HF=-836.
3230884\RMSD=7.222e-09\RMSF=1.357e-05\Dipole=1.8567931,0.1342306,0.012
3337\Quadrupole=-2.4685114,1.1928277,1.2756837,1.496942,-0.0154278,0.0
089058\ PG=CS [SG(C1H1S2),X(H2)]\\@

1\1\GINC-ORC174\FOpt\RM08HX\6-311+G(d,p)\C1H3S2(1-)\SNIKOO\25-Jul-2017
\0\\# opt freq 6-311+g(d,p) m08hx\\CH3-SS(-)\\-1,1\S,0.9894421176,-0.9
923163445,0.\S,-0.0071994108,0.8590522696,0.\C,-1.7357405321,0.3043924
205,0.\H,-1.9310101391,-0.3075804607,0.8890427644\H,-2.3965468964,1.18
30825759,0.\H,-1.9310101391,-0.3075804607,-0.8890427644\\Version=EM64L
-G16RevA.03\State=1-A'\HF=-836.3261617\RMSD=8.343e-09\RMSF=6.280e-05\D
ipole=-1.5748497,0.8822265,0.\Quadrupole=-0.1388925,-1.2331573,1.37204
98,2.3061457,0.,0.\PG=CS [SG(C1H1S2),X(H2)]\\@

1\1\GINC-ORC220\FOpt\RM08HX\6-311G(2d,p)\C1H3S2(1-)\ROOT\01-Aug-2017\0
\\# opt freq 6-311g(2d,p) m08hx\\CH3-SS(-)\\-1,1\S,1.0301539343,-0.974
9023274,0.\S,-0.0005155768,0.8463952555,0.\C,-1.7187805421,0.264507475
,0.\H,-1.9004483585,-0.3480461962,0.8896144901\H,-2.3939940984,1.13056
99894,0.\H,-1.9004483585,-0.3480461962,-0.8896144901\\Version=EM64L-G1
6RevA.03\State=1-A'\HF=-836.3324674\RMSD=5.407e-09\RMSF=5.004e-05\Dipo
le=-1.5797151,0.7775267,0.\Quadrupole=-0.2273836,-0.9143105,1.1416941,
2.1828066,0.,0.\PG=CS [SG(C1H1S2),X(H2)]\\@

1\1\GINC-ORC318\FOpt\RM08HX\6-311G(df,p)\C1H3S2(1-)\SNIKOO\21-Aug-2017
\0\\# opt freq=noraman 6-311g(df,p) m08hx\\CH3-SS\\-1,1\S,-1.341851063
8,0.2972096042,-0.0344441442\S,0.4801511603,-0.7367601114,-0.029960007
2\C,1.6520576719,0.6480345888,-0.0147062192\H,1.4846569752,1.267023899
3,0.8755057242\H,2.6800970649,0.2593682907,-0.0098027669\H,1.496832191
5,1.2766797283,-0.9003625867\\Version=EM64L-G16RevA.03\State=1-A\HF=-8
36.329621\RMSD=3.279e-09\RMSF=8.330e-05\Dipole=1.841622,0.1359245,0.01
1729\Quadrupole=-2.5899953,1.1675739,1.4224214,1.5519345,-0.0143738,0.
0078252\PG=C01 [X(C1H3S2)]\\@

1\1\GINC-ORC36\FOpt\RM08HX\6-311+G(df,p)\C1H3S2(1-)\SNIKOO\25-Jul-2017
\0\\# opt freq 6-311+g(df,p) m08hx\\CH3-SS(-)\\-1,1\S,0.9902380416,-0.
9850689883,0.\S,-0.0053923717,0.8516183303,0.\C,-1.7347716444,0.304186
0835,0.\H,-1.9342827255,-0.3080300808,0.8884567636\H,-2.3935735744,1.1
843747363,0.\H,-1.9342827255,-0.3080300808,-0.8884567636\\Version=EM64
L-G16RevA.03\State=1-A'\HF=-836.3327555\RMSD=3.030e-09\RMSF=3.709e-05\
Dipole=-1.560475,0.871629,0.\Quadrupole=-0.1856802,-1.3206127,1.506292

```
9,2.36647,0.,0.\PG=CS [SG(C1H1S2),X(H2)]\\@
```

```
1\1\GINC-ORC201\FOpt\RM08HX\6-311+G(2df,p)\C1H3S2(1-)\ROOT\25-Jul-2017
 \0\\# opt freq 6-311+g(2df,p) m08hx\\CH3-SS(-)\\-1,1\S,0.9872801845,-0
 .975283258,0.\S,-0.007222487,0.8421436415,0.\C,-1.7345651638,0.3011882
 95,0.\H,-1.9351997543,-0.3071765757,0.8895787523\H,-2.387158025,1.1853
 544729,0.\H,-1.9351997543,-0.3071765757,-0.8895787523\\Version=EM64L-G
 16RevA.03\State=1-A'\HF=-836.3417099\RMSD=3.751e-09\RMSF=3.744e-05\Dip
 ole=-1.4813703,0.8073851,0.\Quadrupole=-0.2671062,-1.0998262,1.3669324
 ,2.2109254,0.,0.\PG=CS [SG(C1H1S2),X(H2)]\\@
```

```
1\1\GINC-ORC224\FOpt\RM08HX\6-311++G(3df,3pd)\C1H3S2(1-)\ROOT\01-Aug-2
 017\0\\# opt freq 6-311++g(3df,3pd) m08hx\\CH3-SS(-)\\-1,1\S,1.0261230
 159,-0.9573649296,0.\S,-0.0018764391,0.8318030582,0.\C,-1.71690696,0.2
 609615833,0.\H,-1.9053200937,-0.3489866584,0.8882317743\H,-2.380732429
 4,1.1330516049,0.\H,-1.9053200937,-0.3489866584,-0.8882317743\\Version
 =EM64L-G16RevA.03\State=1-A'\HF=-836.3463792\RMSD=4.131e-09\RMSF=9.041
 e-06\Dipole=-1.4115877,0.7223873,0.\Quadrupole=-0.3432679,-0.9928953,1
 .3361632,2.0849837,0.,0.\PG=CS [SG(C1H1S2),X(H2)]\\@
```

CH_3SS^- MP2

```
1\1\GINC-ORC185\FOpt\RMP2-FC\6-31G(d)\C1H3S2(1-)\SNIKOO\20-Jul-2017\0\
 \# opt freq mp2/6-31g(d)\\CH3-SS(-)\\-1,1\S,1.0016849066,-0.9835079769
 ,0.\S,-0.0111510634,0.8432839265,0.\C,-1.7422538257,0.3091430043,0.\H,
 -1.9551879514,-0.2962511858,0.8861147104\H,-2.3941731147,1.1919944178,
 0.\H,-1.9551879514,-0.2962511858,-0.8861147104\\Version=EM64L-G09RevD.
 01\State=1-A'\HF=-834.6520469\MP2=-835.0219757\RMSD=2.675e-09\RMSF=2.4
 52e-05\Dipole=-1.6147689,0.7750593,0.\PG=CS [SG(C1H1S2),X(H2)]\\@
```

```
1\1\GINC-ORC6\FOpt\RMP2-FC\6-31G(d,p)\C1H3S2(1-)\SNIKOO\21-Aug-2017\0\
 \# opt freq=noraman mp2/6-31g(d,p)\\CH3-SS\\-1,1\S,-1.358218454,0.2773
 318622,-0.0349757085\S,0.4742293312,-0.7244867437,-0.0301773054\C,1.64
 99011681,0.6511205902,-0.0146300439\H,1.5021699016,1.2703324822,0.8703
 484651\H,2.6696172289,0.2568054697,-0.0099383865\H,1.5142448242,1.2804
 523394,-0.8943970209\\Version=EM64L-G09RevD.01\State=1-A\HF=-834.65699
 4\MP2=-835.0466625\RMSD=4.100e-09\RMSF=5.376e-05\Dipole=1.7497996,0.18
 82973,0.0117247\PG=C01 [X(C1H3S2)]\\@
```

```
1\1\GINC-ORC179\FOpt\RMP2-FC\6-311G(d)\C1H3S2(1-)\SNIKOO\20-Jul-2017\0
 \\# opt freq mp2/6-311g(d) geom=connectivity\\CH3-SS(-)\\-1,1\S,0.9768
 385324,-0.9942597117,0.\S,-0.0178138587,0.8599672884,0.\C,-1.739412711
 5,0.311574969,0.\H,-1.9380583043,-0.2978824384,0.8848250704\H,-2.39976
 43536,1.1868933312,0.\H,-1.9380583043,-0.2978824384,-0.8848250704\\Ver
 sion=EM64L-G09RevD.01\State=1-A'\HF=-834.7110984\MP2=-835.1046066\RMSD
 =4.978e-09\RMSF=1.448e-05\Dipole=-1.6880837,0.8892479,0.\PG=CS [SG(C1H
 1S2),X(H2)]\\@
```

```
1\1\GINC-ORC158\FOpt\RMP2-FC\6-311+G(d)\C1H3S2(1-)\SNIKOO\20-Jul-2017\
 0\\# opt freq mp2/6-311+g(d) geom=connectivity\\CH3-SS(-)\\-1,1\S,0.97
 80161979,-0.9904642912,0.\S,-0.0168266475,0.8574593989,0.\C,-1.7388956
 799,0.3099284287,0.\H,-1.9411340898,-0.2981575521,0.8857081794\H,-2.39
 6294691,1.1878025678,0.\H,-1.9411340898,-0.2981575521,-0.8857081794\\V
 ersion=EM64L-G09RevD.01\State=1-A'\HF=-834.7144917\MP2=-835.1108939\RM
 SD=2.538e-09\RMSF=2.514e-05\Dipole=-1.6712275,0.9175788,0.\PG=CS [SG(C
 1H1S2),X(H2)]\\@
```

1\1\GINC-ORC170\FOpt\RMP2-FC\6-311G(d,p)\C1H3S2(1-)\SNIKOO\21-Aug-2017
\0\\# opt freq=noraman mp2/6-311g(d,p)\\CH3-SS\\-1,1\S,-1.3422480393,0
.3002910111,-0.0352600531\S,0.4881493984,-0.7360938553,-0.0294559657\C
,1.6478146331,0.6461080943,-0.0145726985\H,1.4857579712,1.2653555114,0
.8724919228\H,2.6756583029,0.2607255773,-0.0103015881\H,1.4968117337,1
.2751696612,-0.8966716175\\Version=EM64L-G09RevD.01\State=1-A\HF=-834.
7159397\MP2=-835.1284802\RMSD=5.534e-09\RMSF=1.761e-05\Dipole=1.857034
,0.118811,0.0123222\PG=C01 [X(C1H3S2)]\\@

1\1\GINC-ORC128\FOpt\RMP2-FC\6-311+G(d,p)\C1H3S2(1-)\SNIKOO\20-Jul-201
7\0\\# opt freq mp2/6-311+g(d,p) geom=connectivity\\CH3-SS(-)\\-1,1\S,
0.9757479057,-0.9901152699,0.\S,-0.0169560489,0.8584121967,0.\C,-1.736
5490012,0.3103742411,0.\H,-1.9411414287,-0.2991537885,0.8856364948\H,-
2.3962289983,1.1880474092,0.\H,-1.9411414287,-0.2991537885,-0.88563649
48\\Version=EM64L-G09RevD.01\State=1-A'\HF=-834.7192386\MP2=-835.13467
65\RMSD=9.622e-09\RMSF=2.728e-05\Dipole=-1.6151487,0.919616,0.\PG=CS [
SG(C1H1S2),X(H2)]\\@

1\1\GINC-ORC280\FOpt\RMP2-FC\6-311G(2d,p)\C1H3S2(1-)\SNIKOO\27-Jul-201
7\0\\# opt freq mp2/6-311g(2d,p)\\CH3-SS(-)\\-1,1\S,0.9903688715,-0.99
45514793,0.\S,-0.0135347433,0.8578342153,0.\C,-1.7461965156,0.30848496
59,0.\H,-1.9442524107,-0.2962220977,0.8872726628\H,-2.3984017913,1.189
0874935,0.\H,-1.9442524107,-0.2962220977,-0.8872726628\\Version=EM64L-
G09RevD.01\State=1-A'\HF=-834.725424\MP2=-835.184861\RMSD=2.659e-09\RM
SF=1.754e-05\Dipole=-1.5971358,0.82585,0.\PG=CS [SG(C1H1S2),X(H2)]\\@

1\1\GINC-ORC318\FOpt\RMP2-FC\6-311G(df,p)\C1H3S2(1-)\SNIKOO\21-Aug-201
7\0\\# opt freq=noraman mp2/6-311g(df,p)\\CH3-SS\\-1,1\S,-1.3234316382
,0.290413929,-0.035049434\S,0.4782869596,-0.723039488,-0.0303879792\C,
1.6443195662,0.6451708443,-0.0145584638\H,1.4856110362,1.2666907468,0.
8725255558\H,2.6702539001,0.254807137,-0.0103800799\H,1.4969041761,1.2
775128309,-0.8959195988\\Version=EM64L-G09RevD.01\State=1-A\HF=-834.72
24453\MP2=-835.2002958\RMSD=6.448e-09\RMSF=6.963e-05\Dipole=1.8243153,
0.1329864,0.0120673\PG=C01 [X(C1H3S2)]\\@

1\1\GINC-ORC69\FOpt\RMP2-FC\6-311+G(df,p)\C1H3S2(1-)\SNIKOO\20-Jul-201
7\0\\# opt freq mp2/6-311+g(df,p) geom=connectivity\\CH3-SS(-)\\-1,1\S
,0.9669733109,-0.971736815,0.\S,-0.0157500756,0.8418713784,0.\C,-1.733
395905,0.309140514,0.\H,-1.9429231091,-0.3006555173,0.8853406747\H,-2.
3882501121,1.1904469571,0.\H,-1.9429231091,-0.3006555173,-0.8853406747
\\Version=EM64L-G09RevD.01\State=1-A'\HF=-834.7258525\MP2=-835.2063704
\RMSD=6.504e-09\RMSF=8.657e-06\Dipole=-1.589618,0.8885104,0.\PG=CS [SG
(C1H1S2),X(H2)]\\@

1\1\GINC-ORC5\FOpt\RMP2-FC\6-311+G(2df,p)\C1H3S2(1-)\SNIKOO\20-Jul-201
7\0\\# opt freq mp2/6-311+g(2df,p) geom=connectivity\\CH3-SS(-)\\-1,1\
S,0.9780556005,-0.9744074599,0.\S,-0.0135201126,0.8419555755,0.\C,-1.7
420244991,0.3056010439,0.\H,-1.9456419656,-0.2984079183,0.8880682509\H
,-2.3874960575,1.1920776772,0.\H,-1.9456419656,-0.2984079183,-0.888068
2509\\Version=EM64L-G09RevD.01\State=1-A'\HF=-834.7356672\MP2=-835.256
3761\RMSD=6.501e-09\RMSF=6.319e-06\Dipole=-1.5448435,0.8187626,0.\PG=C
S [SG(C1H1S2),X(H2)]\\@

1\1\GINC-ORC175\FOpt\RMP2-FC\6-311++G(3df,3pd)\C1H3S2(1-)\SNIKOO\27-Ju
l-2017\0\\# opt freq mp2/6-311++g(3df,3pd)\\CH3-SS(-)\\-1,1\S,0.971571
6169,-0.9677903647,0.\S,-0.0197416329,0.836569627,0.\C,-1.7417249815,0
.3040041768,0.\H,-1.9432186694,-0.2981190702,0.8865519926\H,-2.3799366

637,1.1918657014,0.\H,-1.9432186694,-0.2981190702,-0.8865519926\\Version=EM64L-G09RevD.01\State=1-A'\HF=-834.7387318\MP2=-835.2766606\RMSD=8.017e-09\RMSF=8.878e-06\Dipole=-1.4737954,0.7766279,0.\PG=CS [SG(C1H1S2),X(H2)]\\@

CH_3SS^- QCISD

1\1\GINC-ORC28\FOpt\RQCISD-FC\6-311+G(2df,p)\C1H3S2(1-)\SNIKOO\19-Aug-2017\0\\# opt freq=noraman 6-311+g(2df,p) qcisd\\CH3-SS(-)\\-1,1\S,1.0050037632,-0.985361335,0.\S,-0.002583506,0.8433853729,0.\C,-1.7373339947,0.30770771,0.\H,-1.9473410056,-0.2950837979,0.8887606285\H,-2.3820452512,1.196405848,0.\H,-1.9473410056,-0.2950837979,-0.8887606285\\Version=EM64L-G09RevD.01\State=1-A'\HF=-834.7356516\MP2=-835.2562429\MP3=-835.2956544\MP4D=-835.3096574\MP4DQ=-835.2938144\MP4SDQ=-835.2975895\QCISD=-835.297464\RMSD=5.747e-09\RMSF=6.718e-06\Dipole=-1.5473221,0.8201295,0.\PG=CS [SG(C1H1S2),X(H2)]\\@

$CH_3SS^·$ B3LYP

1\1\GINC-ORC282\FOpt\UB3LYP\6-31G(d)\C1H3S2(2)\SNIKOO\17-Aug-2017\0\\# opt freq ub3lyp/6-31g(d)\\CH3-SSSH\\0,2\S,0.4332290635,-0.7603089932,0.4451633567\S,-1.3121421618,-0.4351870601,-0.4609796316\C,-2.0584852202,1.0173176285,0.3827118192\H,-1.4046430487,1.8857280092,0.2750875158\H,-3.0199611913,1.2099901182,-0.1037354244\H,-2.2132304273,0.7932703039,1.4405803756\\Version=EM64L-G09RevD.01\State=2-A"\HF=-836.2776286\S2=0.752627\S2-1=0.\S2A=0.750005\RMSD=3.583e-09\RMSF=1.444e-04\Dipole=-0.793972,0.6878273,0.0938886\Quadrupole=0.4143615,0.2294727,-0.6438342,-0.8530992,-0.3875528,-0.2084015\PG=CS [SG(C1H1S2),X(H2)]\\@

1\1\GINC-ORC68\FOpt\UB3LYP\6-311G(2d,p)\C1H3S2(2)\SNIKOO\28-Aug-2017\0\\# opt freq ub3lyp/6-311g(2d,p)\\CH3-SS\\0,2\S,1.0539254971,-0.9081076079,0.\S,-0.0015329646,0.7738073184,0.\C,-1.7631610612,0.2667138277,0.\H,-1.9718684976,-0.3176451352,0.8937361093\H,-2.3556294761,1.182771732,0.\H,-1.9718684976,-0.3176451352,-0.8937361093\\Version=EM64L-G09RevD.01\State=2-A"\HF=-836.3525638\S2=0.754179\S2-1=0.\S2A=0.750012\RMSD=7.330e-09\RMSF=1.425e-04\Dipole=-1.0033106,0.2914993,0.\Quadrupole=0.9598821,-0.6088723,-0.3510098,-0.7389379,0.,0.\PG=CS [SG(C1H1S2),X(H2)]\\@

1\1\GINC-ORC68\FOpt\UB3LYP\6-311+G(2df,p)\C1H3S2(2)\SNIKOO\28-Aug-2017\0\\# opt freq ub3lyp/6-311+g(2df,p)\\CH3-SSSH\\0,2\S,1.0516928778,-0.9000202198,0.\S,-0.0015738115,0.7672092119,0.\C,-1.7588614507,0.2638152762,0.\H,-1.970508839,-0.3211030744,0.8931062459\H,-2.3518139375,1.1796118805,0.\H,-1.970508839,-0.3211030744,-0.8931062459\\Version=EM64L-G09RevD.01\State=2-A"\HF=-836.3618487\S2=0.754701\S2-1=0.\S2A=0.750016\RMSD=4.437e-09\RMSF=8.495e-05\Dipole=-0.9610123,0.2809598,0.\Quadrupole=0.9622392,-0.6970537,-0.2651855,-0.65297,0.,0.\PG=CS [SG(C1H1S2),X(H2)]\\@

1\1\GINC-ORC100\FOpt\UB3LYP\6-311++G(3df,3pd)\C1H3S2(2)\SNIKOO\28-Aug-2017\0\\# opt freq ub3lyp/6-311++g(3df,3pd)\\CH3-SSSH\\0,2\S,1.0497803603,-0.8963960522,0.\S,-0.0015877611,0.7624666274,0.\C,-1.7550385107,0.2656240362,0.\H,-1.9691828208,-0.3176138435,0.8917893975\H,-2.3440054469,1.1820770757,0.\H,-1.9691828208,-0.3176138435,-0.8917893975\\Version=EM64L-G09RevD.01\State=2-A"\HF=-836.3657272\S2=0.755116\S2-1=0.\S2A=0.750019\RMSD=6.495e-09\RMSF=9.455e-05\Dipole=-0.945991,0.266831,0.\Quadrupole=0.9903869,-0.676303,-0.3140839,-0.7077367,0.,0.\PG=CS [SG(C1

H1S2),X(H2)]\\@

$CH_3SS^{\cdot}$ B3PW91

1\1\GINC-ORC81\FOpt\UB3PW91\6-31G(d)\C1H3S2(2)\SNIKOO\17-Aug-2017\0\\# opt freq ub3pw91/6-31g(d)\\CH3-SSSH\\0,2\S,0.419942365,-0.7527513347,0.4430294659\S,-1.3119313785,-0.4293209257,-0.4553348605\C,-2.0540716707,1.0123378843,0.3811061299\H,-1.4024990687,1.88307509,0.2746797387\H,-3.0159345476,1.2063820665,-0.1043238025\H,-2.2107386888,0.7910872306,1.4396713416\\Version=EM64L-G09RevD.01\State=2-A"\HF=-836.1615603\S2=0.752606\S2-1=0.\S2A=0.750005\RMSD=4.445e-09\RMSF=1.666e-04\Dipole=-0.805078,0.7028919,0.1003041\Quadrupole=0.4097612,0.2437448,-0.653506,-0.8685455,-0.4050446,-0.2054992\PG=CS [SG(C1H1S2),X(H2)]\\@

1\1\GINC-ORC86\FOpt\UB3PW91\6-311G(2d,p)\C1H3S2(2)\SNIKOO\17-Aug-2017\0\\# opt freq ub3pw91/6-311g(2d,p)\\CH3-SS\\0,2\S,0.4113583647,-0.7458359004,0.4435562003\S,-1.3138479869,-0.4235486022,-0.4512539483\C,-2.0538562863,1.0134592902,0.3823066912\H,-1.3990699421,1.8768917311,0.2720858793\H,-3.0129914589,1.2042858522,-0.1042468232\H,-2.2068256762,0.7855576353,1.4363800119\\Version=EM64L-G09RevD.01\State=2-A"\HF=-836.2344766\S2=0.754333\S2-1=0.\S2A=0.750013\RMSD=3.240e-09\RMSF=2.172e-04\Dipole=-0.8037296,0.6738747,0.0740407\Quadrupole=0.6027294,0.0722604,-0.6749899,-0.8911358,-0.1657335,-0.2108517\PG=CS [SG(C1H1S2),X(H2)]\\@

1\1\GINC-ORC246\FOpt\UB3PW91\6-311+G(2df,p)\C1H3S2(2)\SNIKOO\18-Aug-2017\0\\# opt freq ub3pw91/6-311+g(2df,p)\\CH3-SS\\0,2\S,0.4038856328,-0.744209607,0.4398962037\S,-1.3101336469,-0.421591538,-0.4468426143\C,-2.0518878779,1.0111240028,0.3814833721\H,-1.3994152357,1.877144933,0.2730784417\H,-3.011095785,1.2017411448,-0.1053169003\H,-2.206586074,0.786601072,1.436529509\\Version=EM64L-G09RevD.01\State=2-A"\HF=-836.2440098\S2=0.754982\S2-1=0.\S2A=0.750018\RMSD=3.882e-09\RMSF=2.396e-04\Dipole=-0.7725023,0.64666,0.0701961\Quadrupole=0.5203096,0.1308806,-0.6511902,-0.8587607,-0.2506661,-0.2119388\PG=CS [SG(C1H1S2),X(H2)]\\@

1\1\GINC-ORC234\FOpt\UB3PW91\6-311++G(3df,3pd)\C1H3S2(2)\SNIKOO\18-Aug-2017\0\\# opt freq ub3pw91/6-311++g(3df,3pd)\\CH3-SS\\0,2\S,0.3998793148,-0.7439439924,0.4373656987\S,-1.3089554097,-0.4183369624,-0.4429745563\C,-2.0508918101,1.0106217429,0.3817036309\H,-1.4002945549,1.8764030449,0.2733642216\H,-3.008440714,1.1989429513,-0.1060977297\H,-2.2065298106,0.787123221,1.4354667459\\Version=EM64L-G09RevD.01\State=2-A"\HF=-836.2474984\S2=0.755511\S2-1=0.\S2A=0.750022\RMSD=3.024e-09\RMSF=2.391e-04\Dipole=-0.7534427,0.6410867,0.0781951\Quadrupole=0.5805022,0.1054809,-0.6859831,-0.9057733,-0.2150075,-0.2170692\PG=CS [SG(C1H1S2),X(H2)]\\@

$CH_3SS^{\cdot}$ wB97XD

1\1\GINC-ORC141\FOpt\UwB97XD\6-31G(d)\C1H3S2(2)\SNIKOO\18-Aug-2017\0\\# opt freq uwb97xd/6-31g(d)\\CH3-SS\\0,2\S,0.4119609095,-0.7438131069,0.4458702655\S,-1.3144972517,-0.42946556,-0.4572505646\C,-2.0520917118,1.0101904631,0.3804669179\H,-1.3996192022,1.8792416316,0.2738343357\H,-3.0135677987,1.2068071348,-0.1022833847\H,-2.2074179322,0.7878494455,1.4381904422\\Version=EM64L-G09RevD.01\State=2-A"\HF=-836.2203593\S2=0.753101\S2-1=0.\S2A=0.750007\RMSD=5.970e-09\RMSF=1.564e-04\Dipole=-0.7980281,0.701805,0.1041764\Quadrupole=0.408201,0.2489579,-0.657159,-0.8667197,-0.4234158,-0.2250167\PG=CS [SG(C1H1S2),X(H2)]\\@

1\1\GINC-ORC137\FOpt\UwB97XD\6-311G(2d,p)\C1H3S2(2)\SNIKOO\18-Aug-2017
\0\\# opt freq uwb97xd/6-311g(2d,p)\\CH3-SS\\0,2\S,0.4047798931,-0.737
4423846,0.4468597743\S,-1.3163771375,-0.4243679307,-0.4537765902\C,-2.
0522464446,1.0114253836,0.3815171031\H,-1.3963927029,1.8731135878,0.27
11004174\H,-3.0112588626,1.2057469566,-0.10167525\H,-2.2037377272,0.78
23343894,1.4348025553\\Version=EM64L-G09RevD.01\State=2-A"\HF=-836.293
3344\S2=0.75514\S2-1=0.\S2A=0.750017\RMSD=5.734e-09\RMSF=1.926e-04\Dip
ole=-0.8033839,0.6734842,0.0739146\Quadrupole=0.606658,0.0681241,-0.67
47821,-0.8847415,-0.1692841,-0.2268699\PG=CS [SG(C1H1S2),X(H2)]\\@

1\1\GINC-ORC17\FOpt\UwB97XD\6-311+G(2df,p)\C1H3S2(2)\SNIKOO\18-Aug-201
7\0\\# opt freq uwb97xd/6-311+g(2df,p)\\CH3-SS\\0,2\S,0.397404957,-0.7
363479076,0.4427691354\S,-1.3124344179,-0.4220962545,-0.4489119156\C,-
2.0502659541,1.0091641241,0.3807715553\H,-1.3969681008,1.8736014371,0.
2721605681\H,-3.0092455307,1.2028703553,-0.102974795\H,-2.2037239459,0
.7836182608,1.4350134668\\Version=EM64L-G09RevD.01\State=2-A"\HF=-836.
3027703\S2=0.755554\S2-1=0.\S2A=0.75002\RMSD=2.300e-09\RMSF=2.021e-04\
Dipole=-0.7741456,0.6471227,0.0694897\Quadrupole=0.530066,0.1331919,-0
.6632579,-0.8722913,-0.2588862,-0.2222752\PG=CS [SG(C1H1S2),X(H2)]\\@

1\1\GINC-ORC14\FOpt\UwB97XD\6-311++G(3df,3pd)\C1H3S2(2)\SNIKOO\18-Aug-
2017\0\\# opt freq uwb97xd/6-311++g(3df,3pd)\\CH3-SS\\0,2\S,0.39355240
72,-0.7368606706,0.4396157098\S,-1.3108328048,-0.418563741,-0.44448960
99\C,-2.0491818679,1.0087534057,0.381138684\H,-1.3984235397,1.87318395
85,0.2725657539\H,-3.0062298052,1.1996614283,-0.1038904072\H,-2.204117
3773,0.784635628,1.4338878816\\Version=EM64L-G09RevD.01\State=2-A"\HF=
-836.3066503\S2=0.756113\S2-1=0.\S2A=0.750024\RMSD=5.633e-09\RMSF=1.96
3e-04\Dipole=-0.754346,0.6429344,0.0793003\Quadrupole=0.6036393,0.1006
54,-0.7042933,-0.9281052,-0.2124881,-0.2270891\PG=CS [SG(C1H1S2),X(H2)
]\\@

$CH_3SS^{\cdot}$ M062X

1\1\GINC-ORC155\FOpt\UM062X\6-31G(d)\C1H3S2(2)\SNIKOO\18-Aug-2017\0\\#
opt freq m062x/6-31g(d)\\CH3-SS\\0,2\S,0.4015810174,-0.7287358952,0.4
528013816\S,-1.3183244765,-0.4309756368,-0.461321238\C,-2.0521480772,1
.008122121,0.3784890829\H,-1.3927829103,1.8707122692,0.2718760448\H,-3
.0137375788,1.2113393426,-0.0981529748\H,-2.1998209637,0.78034781,1.43
51357167\\Version=EM64L-G09RevD.01\State=2-A"\HF=-836.1716358\S2=0.753
113\S2-1=0.\S2A=0.750006\RMSD=9.178e-09\RMSF=7.065e-05\Dipole=-0.79197
13,0.6821762,0.0899796\Quadrupole=0.4082567,0.2236964,-0.6319531,-0.83
06252,-0.3887167,-0.2225354\PG=CS [SG(C1H1S2),X(H2)]\\@

1\1\GINC-ORC154\FOpt\UM062X\6-311G(2d,p)\C1H3S2(2)\SNIKOO\18-Aug-2017\
0\\# opt freq m062x/6-311g(2d,p)\\CH3-SS\\0,2\S,0.3946739968,-0.722172
6046,0.4541614824\S,-1.3209113935,-0.4254662943,-0.4579518692\C,-2.052
6680543,1.0098448213,0.3797430848\H,-1.3892610038,1.8640479092,0.26909
91383\H,-3.0113709812,1.2100573539,-0.0977127469\H,-2.1956955534,0.774
4988254,1.4314889237\\Version=EM64L-G09RevD.01\State=2-A"\HF=-836.2541
169\S2=0.754742\S2-1=0.\S2A=0.750013\RMSD=4.011e-09\RMSF=6.887e-05\Dip
ole=-0.8038631,0.6627075,0.0634808\Quadrupole=0.6151827,0.039681,-0.65
48637,-0.8552777,-0.1309627,-0.2266183\PG=CS [SG(C1H1S2),X(H2)]\\@

1\1\GINC-ORC148\FOpt\UM062X\6-311+G(2df,p)\C1H3S2(2)\SNIKOO\18-Aug-201
7\0\\# opt freq m062x/6-311+g(2df,p)\\CH3-SS\\0,2\S,0.3880988783,-0.72
21017352,0.4496662717\S,-1.3164650721,-0.4233018969,-0.4528383656\C,-2

.0506611529,1.0073916386,0.3788359636\H,-1.3902054134,1.8652239214,0.2703478051\H,-3.0098315453,1.207286499,-0.0992419483\H,-2.1961686781,0.7763115763,1.4320582834\\Version=EM64L-G09RevD.01\State=2-A"\HF=-836.2636124\S2=0.754779\S2-1=0.\S2A=0.750014\RMSD=4.220e-09\RMSF=2.185e-04\Dipole=-0.7705367,0.6341156,0.0598015\Quadrupole=0.5392426,0.1096787,-0.6489212,-0.8506495,-0.2275479,-0.222969\PG=CS [SG(C1H1S2),X(H2)]\\@

1\1\GINC-ORC146\FOpt\UM062X\6-311++G(3df,3pd)\C1H3S2(2)\SNIKOO\18-Aug-2017\0\\# opt freq m062x/6-311++g(3df,3pd)\\CH3-SS\\0,2\S,0.3848022984,-0.7222560929,0.4472345088\S,-1.3151205323,-0.4205075847,-0.4492863525\C,-2.0500406469,1.0073228171,0.3792019451\H,-1.3914537291,1.8643731571,0.2707295181\H,-3.0072058995,1.2047922333,-0.0997583098\H,-2.1962144838,0.7770854864,1.4307067058\\Version=EM64L-G09RevD.01\State=2-A"\HF=-836.2677864\S2=0.755464\S2-1=0.\S2A=0.750017\RMSD=5.241e-09\RMSF=2.156e-04\Dipole=-0.7489376,0.6269901,0.0681074\Quadrupole=0.6120975,0.0765404,-0.688638,-0.9056262,-0.178737,-0.2248919\PG=CS [SG(C1H1S2),X(H2)]\\@

$CH_3SS^{\cdot}$ M08HX

1\1\GINC-ORC186\FOpt\UM08HX\6-31G(d)\C1H3S2(2)\SNIKOO\19-Aug-2017\0\\# opt freq=noraman 6-31g(d) m08hx\\CH3-SS\\0,2\S,1.0110283712,-0.907225429,0.\S,-0.0052924164,0.7823510264,0.\C,-1.7510672668,0.2714250099,0.\H,-1.964613685,-0.3215575567,0.8959933246\H,-2.364545318,1.180359506,0.\H,-1.964613685,-0.3215575567,-0.8959933246\\Version=EM64L-G16RevA.03\State=2-A"\HF=-836.1752046\S2=0.753295\S2-1=0.\S2A=0.750008\RMSD=7.810e-09\RMSF=1.890e-05\Dipole=-1.0195928,0.2704865,0.\Quadrupole=1.0181764,-0.8635631,-0.1546134,-0.5441427,0.,0.\PG=CS [SG(C1H1S2),X(H2)]\\@

1\1\GINC-ORC327\FOpt\UM08HX\6-311G(2d,p)\C1H3S2(2)\SNIKOO\19-Aug-2017\0\\# opt freq=noraman 6-311g(2d,p) m08hx\\CH3-SS\\0,2\S,0.9990971448,-0.9060821529,0.\S,-0.0117536687,0.7805882817,0.\C,-1.7535716918,0.2709024093,0.\H,-1.9548493398,-0.3200432004,0.8949409578\H,-2.3631771046,1.1784728628,0.\H,-1.9548493398,-0.3200432004,-0.8949409578\\Version=EM64L-G16RevA.03\State=2-A"\HF=-836.2644125\S2=0.75499\S2-1=0.\S2A=0.750015\RMSD=3.659e-09\RMSF=1.669e-05\Dipole=-0.9876831,0.2915008,0.\Quadrupole=0.938166,-0.5853137,-0.3528524,-0.730524,0.,0.\PG=CS [SG(C1H1S2),X(H2)]\\@

1\1\GINC-ORC28\FOpt\UM08HX\6-311+G(2df,p)\C1H3S2(2)\SNIKOO\19-Aug-2017\0\\# opt freq=noraman 6-311+g(2df,p) m08hx\\CH3-SS\\0,2\S,0.9965716413,-0.8984454697,0.\S,-0.0120335399,0.7733065726,0.\C,-1.7509625048,0.2707206213,0.\H,-1.9565867653,-0.320601435,0.8945273084\H,-2.359506066,1.1794161458,0.\H,-1.9565867653,-0.320601435,-0.8945273084\\Version=EM64L-G16RevA.03\State=2-A"\HF=-836.274509\S2=0.755686\S2-1=0.\S2A=0.750021\RMSD=6.286e-09\RMSF=2.172e-05\Dipole=-0.9443309,0.2845031,0.\Quadrupole=0.9543757,-0.6921759,-0.2621998,-0.6557436,0.,0.\PG=CS [SG(C1H1S2),X(H2)]\\@

1\1\GINC-ORC19\FOpt\UM08HX\6-311++G(3df,3pd)\C1H3S2(2)\SNIKOO\19-Aug-2017\0\\# opt freq=noraman 6-311++g(3df,3pd) m08hx\\CH3-SS\\0,2\S,0.9951562514,-0.8945747522,0.\S,-0.0142995579,0.7691493394,0.\C,-1.7516011928,0.2699417919,0.\H,-1.9562898716,-0.3196458646,0.8930916302\H,-2.3557797576,1.1785703502,0.\H,-1.9562898716,-0.3196458646,-0.8930916302\\Version=EM64L-G16RevA.03\State=2-A"\HF=-836.2797178\S2=0.756368\S2-1=0.\S2A=0.750026\RMSD=7.271e-09\RMSF=9.205e-06\Dipole=-0.9385556,0.2675144,0.\Quadrupole=1.0107288,-0.670185,-0.3405438,-0.7386301,0.,0.\PG=CS

[SG(C1H1S2),X(H2)]\\@

$CH_3SS^{\cdot}$ QCISD

```
1\1\GINC-ORC9\FOpt\UQCISD-FC\6-311+G(2df,p)\C1H3S2(2)\SNIKOO\19-Aug-20
 17\0\\# opt freq=noraman 6-311+g(2df,p) qcisd\\CH3-SS\\0,2\S,1.0100419
 471,-0.906547248,0.\S,-0.0095014399,0.7813324397,0.\C,-1.7534456767,0.
 2691302975,0.\H,-1.9628191337,-0.318870394,0.8937499138\H,-2.360560563
 1,1.1776202989,0.\H,-1.9628191337,-0.318870394,-0.8937499138\\Version=
 EM64L-G09RevD.01\State=2-A"\HF=-834.7064417\MP2=-835.1947287\MP3=-835.
 2362442\MP4D=-835.2498994\MP4DQ=-835.2349488\PUHF=-834.7111472\PMP2-0=
 -835.197645\PMP3-0=-835.2377468\MP4SDQ=-835.2395897\QCISD=-835.2410953
 \S2=0.772405\S2-1=0.755212\S2A=0.750339\RMSD=5.013e-09\RMSF=1.914e-05\
 Dipole=-0.9033376,0.2402574,0.\PG=CS [SG(C1H1S2),X(H2)]\\@
```

CH_3SSSH B3LYP

```
1\1\GINC-ORC101\FOpt\RB3LYP\6-31G(d)\C1H4S3\SNIKOO\27-Jul-2017\0\\# op
 t freq rb3lyp/6-31g(d)\\CH3-SSSH\\0,1\S,-1.273793327,0.1727060744,0.05
 81433415\S,0.5646859027,-0.751680211,-0.2376225605\C,1.765723745,0.621
 1415463,-0.0459102289\H,1.7850186807,0.9796138752,0.9863622427\H,2.745
 9772937,0.2022631613,-0.2951919428\H,1.5358653383,1.4403917012,-0.7304
 792848\S,-1.6825773956,0.2435236674,2.1295498843\H,-1.1365754679,1.453
 1918152,2.4045071486\\Version=EM64L-G09RevD.01\State=1-A\HF=-1235.0845
 904\RMSD=6.693e-09\RMSF=9.736e-05\Dipole=0.8172811,0.7611125,0.1424567
 \Quadrupole=0.9555417,-0.1915021,-0.7640396,1.013966,-0.5712444,1.1453
 972\PG=C01 [X(C1H4S3)]\\@
```

```
1\1\GINC-ORC121\FOpt\RB3LYP\6-311G(d)\C1H4S3\SNIKOO\27-Jul-2017\0\\# o
 pt freq rb3lyp/6-311g(d)\\CH3-SSSH\\0,1\S,-1.2709351363,0.1772075736,0
 .0485220957\S,0.5742204529,-0.7600137599,-0.2379894177\C,1.7615220563,
 0.6191234299,-0.0425960947\H,1.784957131,0.9713190905,0.9885661216\H,2
 .7422831086,0.2139949314,-0.2994895923\H,1.5211972147,1.4374769114,-0.
 7196110345\S,-1.6846473165,0.2490830162,2.1293803381\H,-1.1242727406,1
 .4529604369,2.402576184\\Version=EM64L-G09RevD.01\State=1-A\HF=-1235.1
 692212\RMSD=9.476e-09\RMSF=4.128e-05\Dipole=0.8380974,0.7526175,0.1623
 361\Quadrupole=0.8776697,-0.1214278,-0.7562419,1.0253764,-0.5422149,1.
 1517215\PG=C01 [X(C1H4S3)]\\@
```

```
1\1\GINC-ORC139\FOpt\RB3LYP\6-311+G(d)\C1H4S3\SNIKOO\27-Jul-2017\0\\#
 opt freq rb3lyp/6-311+g(d)\\CH3-SSSH\\0,1\S,-1.2709795649,0.1802724789
 ,0.0522795992\S,0.5726642279,-0.7574311073,-0.2359927841\C,1.764443407
 7,0.6188777571,-0.0457495103\H,1.7850878785,0.9775626988,0.9834195857\
 H,2.7451207357,0.208975455,-0.2961561364\H,1.5293870412,1.4345724265,-
 0.7281968106\S,-1.6890524663,0.246554795,2.1314010467\H,-1.1323464898,
 1.4517671259,2.4083536097\\Version=EM64L-G09RevD.01\State=1-A\HF=-1235
 .1734584\RMSD=4.290e-09\RMSF=2.902e-05\Dipole=0.7981663,0.7335799,0.14
 54054\Quadrupole=0.9029598,-0.1915508,-0.711409,1.0009977,-0.5703604,1
 .1724022\PG=C01 [X(C1H4S3)]\\@
```

```
1\1\GINC-ORC181\FOpt\RB3LYP\6-311+G(d,p)\C1H4S3\SNIKOO\27-Jul-2017\0\\
 # opt freq rb3lyp/6-311+g(d,p)\\CH3-SSSH\\0,1\S,-1.2722845419,0.179637
 1464,0.0536269211\S,0.572053981,-0.7548714725,-0.2350486116\C,1.765763
 8473,0.620878599,-0.0453069552\H,1.7809632058,0.9810243681,0.983459988
 3\H,2.7448912808,0.2041712842,-0.2909679713\H,1.5327562428,1.433082916
 9,-0.7327152963\S,-1.6877078039,0.2462987837,2.1354688053\H,-1.1321114
 42,1.4509300042,2.4008417198\\Version=EM64L-G09RevD.01\State=1-A\HF=-1
```

235.1821316\RMSD=9.187e-09\RMSF=2.541e-05\Dipole=0.7883013,0.7196742,0
.1414794\Quadrupole=0.931588,-0.2088469,-0.7227411,1.0005232,-0.586056
6,1.1172969\PG=C01 [X(C1H4S3)]\\@

1\1\GINC-ORC246\FOpt\RB3LYP\6-311G(2d,p)\C1H4S3\SNIKOO\27-Jul-2017\0\\
opt freq rb3lyp/6-311g(2d,p)\\CH3-SSSH\\0,1\S,0.505552354,-0.6734420
447,0.4458861431\S,-1.3754678072,-0.456590596,-0.4020547423\C,-2.00726
55587,1.0836853517,0.357722677\H,-1.440291678,1.9439900675,0.006042904
7\H,-3.0426782297,1.1763561365,0.0252142275\H,-1.9770660113,1.01582221
08,1.4430316898\S,1.880240028,0.4525814026,-0.688743957\H,1.8011239029
,1.6021894716,0.0102750572\\Version=EM64L-G09RevD.01\State=1-A\HF=-123
5.1973284\RMSD=4.089e-09\RMSF=9.579e-05\Dipole=-0.4483585,0.8701789,0.
3153537\Quadrupole=0.8652295,0.8255203,-1.6907499,-0.5170918,-0.417446
1,0.762475\PG=C01 [X(C1H4S3)]\\@

1\1\GINC-ORC236\FOpt\RB3LYP\6-311+G(df,p)\C1H4S3\SNIKOO\27-Jul-2017\0\
\# opt freq rb3lyp/6-311+g(df,p)\\CH3-SSSH\\0,1\S,0.5048552384,-0.6695
995497,0.4483911828\S,-1.3802981729,-0.462437523,-0.3967578945\C,-2.01
0867262,1.0796175365,0.3588495856\H,-1.4418564274,1.9427048049,0.01296
71404\H,-3.0451991109,1.1781071217,0.0233620002\H,-1.9852306718,1.0171
001863,1.4460692716\S,1.8931604104,0.4457519756,-0.6869245067\H,1.8095
829962,1.6133474477,-0.0085827794\\Version=EM64L-G09RevD.01\State=1-A\
HF=-1235.1914278\RMSD=7.067e-09\RMSF=2.333e-05\Dipole=-0.439354,0.9106
695,0.3461258\Quadrupole=0.7697685,0.9842283,-1.7539968,-0.4463729,-0.
3350455,0.7903249\PG=C01 [X(C1H4S3)]\\@

1\1\GINC-ORC218\FOpt\RB3LYP\6-311+G(2df,p)\C1H4S3\ROOT\27-Jul-2017\0\\
opt freq rb3lyp/6-311+g(2df,p)\\CH3-SSSH\\0,1\S,0.5033978704,-0.6656
85597,0.4434902563\S,-1.3650507176,-0.4524485161,-0.397416311\C,-2.004
4391989,1.0807462568,0.3572494193\H,-1.4358579296,1.9440891661,0.01465
62292\H,-3.0368321569,1.1749485337,0.0157280613\H,-1.9849527695,1.0132
280099,1.4432626079\S,1.870493931,0.4468723368,-0.6862307195\H,1.79738
79712,1.6028418098,0.0066344565\\Version=EM64L-G09RevD.01\State=1-A\HF
=-1235.2093876\RMSD=3.790e-09\RMSF=9.067e-05\Dipole=-0.4209814,0.83192
66,0.3119209\Quadrupole=0.8339371,0.8409716,-1.6749087,-0.4863912,-0.3
984379,0.7351979\PG=C01 [X(C1H4S3)]\\@

1\1\GINC-ORC195\FOpt\RB3LYP\6-311++G(3df,3pd)\C1H4S3\SNIKOO\27-Jul-201
7\0\\# opt freq rb3lyp/6-311++g(3df,3pd)\\CH3-SSSH\\0,1\S,0.5013174585
,-0.659773853,0.4401298173\S,-1.3623520707,-0.4500085314,-0.3920446907
\C,-2.0036146943,1.0795602151,0.3582785857\H,-1.4331383599,1.941065801
7,0.0196191679\H,-3.0323633592,1.1743068001,0.0113734681\H,-1.99022294
02,1.01224869,1.4427346592\S,1.8668392469,0.4456535964,-0.685442216\H,
1.7976817188,1.6015392812,0.0027252084\\Version=EM64L-G09RevD.01\State
=1-A\HF=-1235.2150166\RMSD=7.473e-09\RMSF=9.735e-05\Dipole=-0.4199457,
0.7951096,0.2919154\Quadrupole=0.9335789,0.713937,-1.6475159,-0.576255
5,-0.4667242,0.6781403\PG=C01 [X(C1H4S3)]\\@

CH_3SSSH B3PW91

1\1\GINC-ORC46\FOpt\RB3PW91\6-31G(d)\C1H4S3\SNIKOO\28-Jul-2017\0\\# op
t freq rb3pw91/6-31g(d)\\CH3-SSSH\\0,1\S,0.4310950787,-0.7768758841,0.
4935997009\S,-1.3202957926,-0.3956121717,-0.526260115\C,-2.0380232823,
1.0122207397,0.3793295686\H,-1.4335452605,1.9131965769,0.2454776433\H,
-3.0269980657,1.1831760784,-0.0583686651\H,-2.1488170453,0.7815061674,
1.4413315004\S,1.9380145658,0.46959359,-0.2491503199\H,1.7840748019,1.
5090689034,0.6026406868\\Version=EM64L-G09RevD.01\State=1-A\HF=-1234.9

1956\RMSD=2.741e-09\RMSF=1.936e-05\Dipole=-0.5079581,0.9294994,0.47356
6\Quadrupole=0.897207,0.6710123,-1.5682193,-0.5186535,-0.0457352,1.200
1982\PG=C01 [X(C1H4S3)]\\@

1\1\GINC-ORC32\FOpt\RB3PW91\6-311G(d)\C1H4S3\SNIKOO\28-Jul-2017\0\\# o
pt freq rb3pw91/6-311g(d)\\CH3-SSSH\\0,1\S,0.4218216458,-0.7793295388,
0.499605973\S,-1.3279789268,-0.3935593742,-0.5365577593\C,-2.032114252
3,1.0113150723,0.3759333893\H,-1.4309142512,1.91093484,0.2388102604\H,
-3.0237410712,1.1848932289,-0.0483108184\H,-2.1324108846,0.7784868896,
1.4358104971\S,1.93963028,0.4689883705,-0.2406654404\H,1.7712124605,1.
5145445116,0.6039738983\\Version=EM64L-G09RevD.01\State=1-A\HF=-1235.0
001783\RMSD=8.775e-09\RMSF=3.287e-05\Dipole=-0.508286,0.939166,0.45282
94\Quadrupole=0.7940331,0.7099154,-1.5039485,-0.4771559,-0.0418458,1.2
278784\PG=C01 [X(C1H4S3)]\\@

1\1\GINC-ORC28\FOpt\RB3PW91\6-311+G(d)\C1H4S3\SNIKOO\28-Jul-2017\0\\#
opt freq rb3pw91/6-311+g(d)\\CH3-SSSH\\0,1\S,0.4242309456,-0.775203671
5,0.5027626006\S,-1.325658045,-0.392442668,-0.5338738817\C,-2.03646889
86,1.0107031948,0.3766933983\H,-1.432097778,1.9096034713,0.247417643\H
,-3.0241447451,1.1876320648,-0.0558920469\H,-2.1458269974,0.7758122644
,1.4354813066\S,1.9444095979,0.4657160927,-0.2433554131\H,1.7810609206
,1.5144532515,0.5993663932\\Version=EM64L-G09RevD.01\State=1-A\HF=-123
5.004766\RMSD=9.654e-09\RMSF=1.284e-05\Dipole=-0.4885924,0.9011647,0.4
503546\Quadrupole=0.8705265,0.7030235,-1.5735501,-0.448466,-0.0383913,
1.1865331\PG=C01 [X(C1H4S3)]\\@

1\1\GINC-ORC22\FOpt\RB3PW91\6-311+G(d,p)\C1H4S3\SNIKOO\28-Jul-2017\0\\
opt freq rb3pw91/6-311+g(d,p)\\CH3-SSSH\\0,1\S,0.4272267271,-0.77440
44687,0.5032507488\S,-1.3245064483,-0.3925496822,-0.5290745003\C,-2.03
72335198,1.0123303985,0.3787437853\H,-1.4290317434,1.9087704498,0.2500
558092\H,-3.0220050455,1.1874796595,-0.0614321231\H,-2.1509216335,0.77
53689263,1.4367091461\S,1.9454057609,0.4694094501,-0.248145233\H,1.776
5709025,1.5098692667,0.5984923669\\Version=EM64L-G09RevD.01\State=1-A\
HF=-1235.0134274\RMSD=4.434e-09\RMSF=1.098e-05\Dipole=-0.4842579,0.885
4329,0.4398257\Quadrupole=0.8965263,0.6531006,-1.5496269,-0.4842889,-0
.0654718,1.1558418\PG=C01 [X(C1H4S3)]\\@

1\1\GINC-ORC18\FOpt\RB3PW91\6-311G(2d,p)\C1H4S3\SNIKOO\28-Jul-2017\0\\
opt freq rb3pw91/6-311g(2d,p)\\CH3-SSSH\\0,1\S,0.4271913364,-0.77434
14935,0.5020088832\S,-1.3112350077,-0.3864953985,-0.5269380721\C,-2.03
11716698,1.0164619691,0.3755654486\H,-1.4274530078,1.9130411377,0.2386
58728\H,-3.017060463,1.1801348723,-0.0652232696\H,-2.1412614672,0.7793
055476,1.4324150159\S,1.9254950006,0.471709843,-0.2434857689\H,1.76100
02785,1.4964575224,0.6155990348\\Version=EM64L-G09RevD.01\State=1-A\HF
=-1235.0302873\RMSD=9.217e-09\RMSF=1.433e-05\Dipole=-0.4819903,0.83745
03,0.3973808\Quadrupole=0.9322797,0.5442318,-1.4765114,-0.5799743,-0.1
62311,1.0862727\PG=C01 [X(C1H4S3)]\\@

1\1\GINC-ORC114\FOpt\RB3PW91\6-311G(df,p)\C1H4S3\SNIKOO\11-Sep-2018\0\
\# opt freq b3pw91/6-311g(df,p)\\CH3-SSSH\\0,1\S,0.435525401,-0.792621
1923,0.4807009867\S,-1.3045388129,-0.3755168506,-0.5391313961\C,-1.997
912872,1.0234071908,0.388245595\H,-1.3853161451,1.9175599396,0.2658541
07\H,-2.9852725933,1.2107160306,-0.0399425176\H,-2.1037084643,0.776376
4702,1.4445941523\S,1.9569362747,0.4498203533,-0.2364525364\H,1.794286
212,1.4786670584,0.6246406093\\Version=EM64L-G09RevE.01\State=1-A\HF=-
1235.018703\RMSD=3.740e-09\RMSF=7.955e-06\Dipole=-0.4897955,0.9118822,
0.4579584\Quadrupole=0.7259634,0.6718877,-1.3978511,-0.5807984,-0.0868

261,1.2337668\PG=C01 [X(C1H4S3)]\\@

1\1\GINC-ORC15\FOpt\RB3PW91\6-311+G(df,p)\C1H4S3\SNIKOO\28-Jul-2017\0\ \# opt freq rb3pw91/6-311+g(df,p)\\CH3-SSSH\\0,1\S,0.425933464,-0.7717 562781,0.4969028059\S,-1.3191106561,-0.3911976075,-0.5263042006\C,-2.0 345699801,1.0114947069,0.3790187815\H,-1.4285707126,1.9096637936,0.251 7425131\H,-3.0194800297,1.1851357269,-0.0608649821\H,-2.1477317474,0.7 76016169,1.4374500631\S,1.9373810349,0.4664564041,-0.2461783046\H,1.77 16536268,1.5104610851,0.5968333238\\Version=EM64L-G09RevD.01\State=1-A \HF=-1235.0231011\RMSD=3.497e-09\RMSF=6.695e-06\Dipole=-0.4795106,0.87 75179,0.4422454\Quadrupole=0.8193888,0.6845958,-1.5039846,-0.5224441,- 0.0784736,1.1635991\PG=C01 [X(C1H4S3)]\\@

1\1\GINC-ORC12\FOpt\RB3PW91\6-311+G(2df,p)\C1H4S3\SNIKOO\28-Jul-2017\0 \\# opt freq rb3pw91/6-311+g(2df,p)\\CH3-SSSH\\0,1\S,0.426008953,-0.76 67610576,0.5014027988\S,-1.3025235657,-0.3848973441,-0.5197157408\C,-2 .028285146,1.013335454,0.3758043633\H,-1.422494163,1.9104742723,0.2487 876544\H,-3.0096279734,1.1812139222,-0.0737105877\H,-2.1496049304,0.77 64209863,1.4319840675\S,1.915110037,0.4671411724,-0.2452014026\H,1.756 9217885,1.4993465946,0.6092488472\\Version=EM64L-G09RevD.01\State=1-A\ HF=-1235.0428676\RMSD=5.251e-09\RMSF=1.614e-05\Dipole=-0.4570489,0.801 6308,0.3920763\Quadrupole=0.9022226,0.5664378,-1.4686604,-0.5514962,-0 .1475831,1.0581513\PG=C01 [X(C1H4S3)]\\@

1\1\GINC-ORC32\FOpt\RB3PW91\6-311++G(3df,3pd)\C1H4S3\SNIKOO\28-Jul-201 7\0\\# opt freq rb3pw91/6-311++g(3df,3pd)\\CH3-SSSH\\0,1\S,0.424266624 8,-0.7621420284,0.4981208439\S,-1.3024892681,-0.3857170178,-0.51333388 99\C,-2.0262312486,1.0118331873,0.3770420382\H,-1.4161792684,1.9048154 369,0.2534126392\H,-3.0030074054,1.1837802244,-0.0773259248\H,-2.15372 38213,0.7759166734,1.4312554798\S,1.9093357377,0.4679400511,-0.2461314 698\H,1.7535336493,1.4998474731,0.6055602835\\Version=EM64L-G09RevD.01 \State=1-A\HF=-1235.0479712\RMSD=3.482e-09\RMSF=1.194e-05\Dipole=-0.45 39321,0.7682843,0.3660707\Quadrupole=1.0170774,0.4689664,-1.4860438,-0 .6299339,-0.2188689,0.9701496\PG=C01 [X(C1H4S3)]\\@

CH_3SSSH wB97XD

1\1\GINC-ORC26\FOpt\RwB97XD\6-31G(d)\C1H4S3\SNIKOO\28-Jul-2017\0\\# op t freq rwb97xd/6-31g(d)\\CH3-SSSH\\0,1\S,0.4283247414,-0.7970110478,0. 4939362397\S,-1.3273905931,-0.4221902244,-0.5178695793\C,-2.0051073179 ,1.007644127,0.3782347604\H,-1.3647099442,1.8838894717,0.2508675969\H, -2.980567161,1.2163130613,-0.0701308075\H,-2.1349334422,0.7826090543,1 .4386085315\S,1.8759567741,0.502261579,-0.2533111123\H,1.6939319429,1. 5227579788,0.6082643706\\Version=EM64L-G09RevD.01\State=1-A\HF=-1235.0 10184\RMSD=7.872e-09\RMSF=2.069e-05\Dipole=-0.4879642,0.9326062,0.4833 581\Quadrupole=0.744417,0.7894359,-1.5338529,-0.5881028,-0.0420096,1.2 171757\PG=C01 [X(C1H4S3)]\\@

1\1\GINC-ORC12\FOpt\RwB97XD\6-311G(d)\C1H4S3\SNIKOO\28-Jul-2017\0\\# o pt freq rwb97xd/6-311g(d)\\CH3-SSSH\\0,1\S,0.4204862006,-0.8000830642, 0.4993827332\S,-1.335183107,-0.4209576472,-0.5279585691\C,-1.999002503 8,1.0067460364,0.3751092225\H,-1.3628276172,1.8822323589,0.2416218617\ H,-2.9784688123,1.2166511827,-0.0582093575\H,-2.1158233154,0.780335339 4,1.4342270303\S,1.8767971733,0.5031992082,-0.2453503046\H,1.679526981 8,1.5281505858,0.6097773835\\Version=EM64L-G09RevD.01\State=1-A\HF=-12 35.0908857\RMSD=5.338e-09\RMSF=2.143e-05\Dipole=-0.4886506,0.9406705,0 .4657931\Quadrupole=0.6393854,0.8425063,-1.4818917,-0.5545981,-0.03317

```
 51,1.2438262\PG=C01 [X(C1H4S3)]\\@

1\1\GINC-ORC272\FOpt\RwB97XD\6-311+G(d)\C1H4S3\SNIKOO\28-Jul-2017\0\\#
  opt freq rwb97xd/6-311+g(d)\\CH3-SSSH\\0,1\S,0.4226348095,-0.79619992
 53,0.5011996948\S,-1.3327868366,-0.4189066851,-0.5259665765\C,-2.00315
 26779,1.0059680525,0.3770471276\H,-1.3640050158,1.8807739027,0.2521205
 862\H,-2.9787840706,1.2195132507,-0.0635713874\H,-2.1283529187,0.77652
 20033,1.4348119652\S,1.8822453337,0.4996878311,-0.249245886\H,1.687706
 3765,1.52891557,0.6022044761\\Version=EM64L-G09RevD.01\State=1-A\HF=-1
 235.095415\RMSD=5.399e-09\RMSF=1.904e-05\Dipole=-0.4689473,0.9061093,0
 .4614188\Quadrupole=0.7347249,0.8302381,-1.564963,-0.5267626,-0.037354
 7,1.2072039\PG=C01 [X(C1H4S3)]\\@

1\1\GINC-ORC271\FOpt\RwB97XD\6-311+G(d,p)\C1H4S3\SNIKOO\28-Jul-2017\0\
 \# opt freq rwb97xd/6-311+g(d,p)\\CH3-SSSH\\0,1\S,0.4247888978,-0.7960
 918534,0.5003968402\S,-1.331844535,-0.4178743255,-0.523278513\C,-2.002
 9832738,1.0071662322,0.3798064838\H,-1.3584810595,1.8786741723,0.25906
 51654\H,-2.9747693416,1.2223053055,-0.0686075422\H,-2.1343131233,0.773
 6982807,1.4360267477\S,1.8828349716,0.5022171872,-0.2535017404\H,1.680
 2724638,1.526179001,0.5986925584\\Version=EM64L-G09RevD.01\State=1-A\H
 F=-1235.1038886\RMSD=5.332e-09\RMSF=1.940e-05\Dipole=-0.4639695,0.8883
 445,0.4496462\Quadrupole=0.7573157,0.7854507,-1.5427664,-0.5652654,-0.
 0765545,1.1721106\PG=C01 [X(C1H4S3)]\\@

1\1\GINC-ORC269\FOpt\RwB97XD\6-311G(2d,p)\C1H4S3\SNIKOO\28-Jul-2017\0\
 \# opt freq rwb97xd/6-311g(2d,p)\\CH3-SSSH\\0,1\S,0.4254346719,-0.7925
 772297,0.4983716377\S,-1.321731654,-0.4120999441,-0.5184610399\C,-2.00
 10572453,1.0112580998,0.37896054\H,-1.3610621132,1.8832490126,0.250798
 1944\H,-2.9737677207,1.2149664497,-0.0715033343\H,-2.1304970921,0.7778
 951454,1.433920275\S,1.8682771019,0.5025712684,-0.255118556\H,1.679909
 0515,1.511011198,0.6116322832\\Version=EM64L-G09RevD.01\State=1-A\HF=-
 1235.1208575\RMSD=5.911e-09\RMSF=1.956e-05\Dipole=-0.462051,0.8413515,
 0.407698\Quadrupole=0.7939138,0.6708031,-1.464717,-0.6196787,-0.160884
 4,1.0917763\PG=C01 [X(C1H4S3)]\\@

1\1\GINC-ORC116\FOpt\RwB97XD\6-311G(df,p)\C1H4S3\SNIKOO\11-Sep-2018\0\
 \# opt freq wb97xd/6-311g(df,p)\\CH3-SSSH\\0,1\S,0.4332850437,-0.81364
 74278,0.4825683947\S,-1.3116221377,-0.4034064778,-0.5306158421\C,-1.96
 45484678,1.0179514766,0.3869545324\H,-1.3161043608,1.8868838846,0.2699
 780457\H,-2.9382769397,1.2434854199,-0.0510917702\H,-2.0892245219,0.77
 75731329,1.4422755818\S,1.8962305161,0.4832516559,-0.2386955049\H,1.70
 0259868,1.4963173358,0.6271355626\\Version=EM64L-G09RevE.01\State=1-A\
 HF=-1235.1093836\RMSD=3.266e-09\RMSF=1.903e-05\Dipole=-0.472898,0.9114
 851,0.4669088\Quadrupole=0.576217,0.8047196,-1.3809366,-0.6615426,-0.0
 917085,1.242459\PG=C01 [X(C1H4S3)]\\@

1\1\GINC-ORC270\FOpt\RwB97XD\6-311+G(df,p)\C1H4S3\SNIKOO\28-Jul-2017\0
 \\# opt freq rwb97xd/6-311+g(df,p)\\CH3-SSSH\\0,1\S,0.4230366334,-0.79
 42167746,0.4942487363\S,-1.3270042423,-0.4166745213,-0.5199935107\C,-2
 .0005805005,1.0066279945,0.3795937342\H,-1.3573526661,1.879410926,0.26
 08091469\H,-2.9719132924,1.2210593579,-0.0695853569\H,-2.1327516175,0.
 7749033452,1.4361692346\S,1.8758703528,0.4993562738,-0.251210591\H,1.6
 762003326,1.5258073985,0.5985686066\\Version=EM64L-G09RevD.01\State=1-
 A\HF=-1235.1137475\RMSD=7.906e-09\RMSF=2.025e-05\Dipole=-0.4617826,0.8
 806498,0.4520776\Quadrupole=0.6882609,0.8053211,-1.4935819,-0.6066464,
 -0.088064,1.1821457\PG=C01 [X(C1H4S3)]\\@
```

1\1\GINC-ORC219\FOpt\RwB97XD\6-311+G(2df,p)\C1H4S3\SNIKOO\28-Jul-2017\
0\\# opt freq rwb97xd/6-311+g(2df,p)\\CH3-SSSH\\0,1\S,0.4237371198,-0.
7856617457,0.4974010264\S,-1.3126776809,-0.4087243479,-0.5126520416\C,
-1.9986796776,1.0085404861,0.3792392113\H,-1.3588568384,1.8823579822,0
.2597586676\H,-2.9682049648,1.214319958,-0.0772566478\H,-2.1362063007,
0.7753463869,1.4336836186\S,1.8612547783,0.4961306883,-0.2552932435\H,
1.6751385643,1.5139645921,0.6037194089\\Version=EM64L-G09RevD.01\State
=1-A\HF=-1235.1335663\RMSD=5.547e-09\RMSF=2.384e-05\Dipole=-0.4399803,
0.8085092,0.4015754\Quadrupole=0.7922619,0.6834806,-1.4757425,-0.60061
12,-0.153726,1.0718402\PG=C01 [X(C1H4S3)]\\@

1\1\GINC-ORC116\FOpt\RwB97XD\6-311G(df,p)\C1H4S3\SNIKOO\11-Sep-2018\0\
\# opt freq wb97xd/6-311g(df,p)\\CH3-SSSH\\0,1\S,0.4332850437,-0.81364
74278,0.4825683947\S,-1.3116221377,-0.4034064778,-0.5306158421\C,-1.96
45484678,1.0179514766,0.3869545324\H,-1.3161043608,1.8868838846,0.2699
780457\H,-2.9382769397,1.2434854199,-0.0510917702\H,-2.0892245219,0.77
75731329,1.4422755818\S,1.8962305161,0.4832516559,-0.2386955049\H,1.70
0259868,1.4963173358,0.6271355626\\Version=EM64L-G09RevE.01\State=1-A\
HF=-1235.1093836\RMSD=3.266e-09\RMSF=1.903e-05\Dipole=-0.472898,0.9114
851,0.4669088\Quadrupole=0.576217,0.8047196,-1.3809366,-0.6615426,-0.0
917085,1.242459\PG=C01 [X(C1H4S3)]\\@

CH_3SSSH M062X

1\1\GINC-ORC23\FOpt\RM062X\6-31G(d)\C1H4S3\SNIKOO\27-Jul-2017\0\\# opt
freq 6-31g(d) m062x\\CH3-SSSH\\0,1\S,-1.2611133735,0.1684908518,0.036
7669775\S,0.5524015249,-0.7650803427,-0.2493173161\C,1.7169972709,0.61
11566708,-0.0156511319\H,1.6814222299,0.9723930095,1.0144193814\H,2.71
43793039,0.2120267016,-0.2173337086\H,1.5015910411,1.4227036703,-0.711
9234864\S,-1.5785898896,0.2668883521,2.0922433032\H,-1.0227633378,1.47
25727167,2.320154581\\Version=EM64L-G09RevD.01\State=1-A\HF=-1234.9420
42\RMSD=8.026e-09\RMSF=4.127e-05\Dipole=0.793966,0.7730366,0.1604866\Q
uadrupole=0.9205993,-0.0864388,-0.8341605,1.0446861,-0.4434068,1.15588
85\PG=C01 [X(C1H4S3)]\\@

1\1\GINC-ORC36\FOpt\RM062X\6-311G(d)\C1H4S3\SNIKOO\27-Jul-2017\0\\# op
t freq m062x/6-311g(d)\\CH3-SSSH\\0,1\S,-1.2569089199,0.1714010344,0.0
273693705\S,0.5604403133,-0.7741326456,-0.2503399631\C,1.7111527294,0.
6087007406,-0.0102483178\H,1.6808815088,0.9591979411,1.0208958666\H,2.
7094567934,0.2253587795,-0.2235441748\H,1.4812419447,1.4219497073,-0.6
957041095\S,-1.572778655,0.272608763,2.0893108676\H,-1.0091609447,1.47
60673098,2.3116190605\\Version=EM64L-G09RevD.01\State=1-A\HF=-1235.037
7061\RMSD=2.974e-09\RMSF=4.691e-05\Dipole=0.8094807,0.7733823,0.175334
3\Quadrupole=0.83765,0.0181185,-0.8557686,1.0635627,-0.3678405,1.18216
96\PG=C01 [X(C1H4S3)]\\@

1\1\GINC-ORC23\FOpt\RM062X\6-311+G(d)\C1H4S3\SNIKOO\27-Jul-2017\0\\# o
pt freq m062x/6-311+g(d)\\CH3-SSSH\\0,1\S,-1.2566248359,0.1778547211,0
.0308878497\S,0.5582882481,-0.7713026817,-0.2469634099\C,1.7146993084,
0.6083839734,-0.0145994347\H,1.6757842879,0.9713035396,1.0120866186\H,
2.7132478308,0.2174445185,-0.2135062911\H,1.4948295122,1.4149835948,-0
.7114462596\S,-1.5806002837,0.2702962519,2.0918517575\H,-1.0152992977,
1.4721877126,2.3210477696\\Version=EM64L-G09RevD.01\State=1-A\HF=-1235
.0426413\RMSD=9.005e-09\RMSF=4.311e-05\Dipole=0.7726757,0.7538344,0.16
41957\Quadrupole=0.8874274,-0.0936568,-0.7937706,1.0304528,-0.4359974,
1.2174105\PG=C01 [X(C1H4S3)]\\@

1\1\GINC-ORC60\FOpt\RM062X\6-311+G(d,p)\C1H4S3\SNIKOO\27-Jul-2017\0\\#
 opt freq m062x/6-311+g(d,p)\\CH3-SSSH\\0,1\S,-1.2573829382,0.17764602
93,0.0316217555\S,0.5580035167,-0.7696358632,-0.245176894\C,1.71593474
67,0.6095401837,-0.0140238307\H,1.6713646441,0.9749926137,1.0115480884
\H,2.7132830956,0.2126503259,-0.2075774917\H,1.4986307778,1.4124120719
,-0.7160859177\S,-1.5806156723,0.2712622675,2.0942568402\H,-1.01489340
05,1.4722840012,2.3147960501\\Version=EM64L-G09RevD.01\State=1-A\HF=-1
235.0498375\RMSD=7.502e-09\RMSF=4.140e-05\Dipole=0.7651308,0.7414666,0
.1598893\Quadrupole=0.9188664,-0.1162266,-0.8026398,1.0332965,-0.45095
58,1.1623518\PG=C01 [X(C1H4S3)]\\@

1\1\GINC-ORC50\FOpt\RM062X\6-311G(2d,p)\C1H4S3\SNIKOO\27-Jul-2017\0\\#
 opt freq m062x/6-311g(2d,p)\\CH3-SSSH\\0,1\S,-1.2582927198,0.16862161
27,0.0336372916\S,0.553679806,-0.7654468753,-0.2364212511\C,1.71205922
75,0.6119630959,-0.0074391539\H,1.6663657516,0.9730360273,1.0181048139
\H,2.7066246932,0.2107449986,-0.2026683593\H,1.4933731159,1.4108786101
,-0.7111883852\S,-1.5614190624,0.2732168148,2.0862495927\H,-1.00806604
2,1.4781373458,2.2890840513\\Version=EM64L-G09RevD.01\State=1-A\HF=-12
35.0657722\RMSD=5.422e-09\RMSF=3.693e-05\Dipole=0.7479466,0.6805966,0.
1448287\Quadrupole=0.9386286,-0.1547357,-0.783893,1.0671126,-0.4408959
,0.9753713\PG=C01 [X(C1H4S3)]\\@

1\1\GINC-ORC50\FOpt\RM062X\6-311G(2d,p)\C1H4S3\SNIKOO\27-Jul-2017\0\\#
 opt freq m062x/6-311g(2d,p)\\CH3-SSSH\\0,1\S,-1.2582927198,0.16862161
27,0.0336372916\S,0.553679806,-0.7654468753,-0.2364212511\C,1.71205922
75,0.6119630959,-0.0074391539\H,1.6663657516,0.9730360273,1.0181048139
\H,2.7066246932,0.2107449986,-0.2026683593\H,1.4933731159,1.4108786101
,-0.7111883852\S,-1.5614190624,0.2732168148,2.0862495927\H,-1.00806604
2,1.4781373458,2.2890840513\\Version=EM64L-G09RevD.01\State=1-A\HF=-12
35.0657722\RMSD=5.422e-09\RMSF=3.693e-05\Dipole=0.7479466,0.6805966,0.
1448287\Quadrupole=0.9386286,-0.1547357,-0.783893,1.0671126,-0.4408959
,0.9753713\PG=C01 [X(C1H4S3)]\\@

1\1\GINC-ORC117\FOpt\RM062X\6-311G(df,p)\C1H4S3\SNIKOO\11-Sep-2018\0\\
opt freq=NORAMAN m062x/6-311g(df,p)\\CH3-SSSH\\0,1\S,0.4222257102,-0
.8101515128,0.4902434257\S,-1.3150114127,-0.4109393988,-0.5356299483\C
,-1.9525574404,1.0158816842,0.3838236027\H,-1.2938660347,1.8755465449,
0.263223002\H,-2.9270749916,1.2519474439,-0.0447798164\H,-2.0674201545
,0.7708287831,1.4381397799\S,1.8672983921,0.4950919502,-0.240180622\H,
1.6764049317,1.5002035053,0.6336695764\\Version=EM64L-G09RevE.01\State
=1-A\HF=-1235.0555421\RMSD=8.758e-09\RMSF=3.509e-05\Dipole=-0.4605619,
0.898261,0.4636353\Quadrupole=0.5275437,0.8048642,-1.3324079,-0.652880
2,-0.0631271,1.2431506\PG=C01 [X(C1H4S3)]\\@

1\1\GINC-ORC21\FOpt\RM062X\6-311+G(df,p)\C1H4S3\SNIKOO\27-Jul-2017\0\\
opt freq m062x/6-311+g(df,p)\\CH3-SSSH\\0,1\S,-1.2537193346,0.175914
8083,0.0356390186\S,0.5524375191,-0.7643610571,-0.2430142605\C,1.71459
5256,0.6087358857,-0.0138629008\H,1.6718086211,0.978328771,1.010550872
6\H,2.710881039,0.2087933353,-0.2062671824\H,1.5011333848,1.4125352187
,-0.7164720434\S,-1.5796211723,0.2709408435,2.0869088633\H,-1.01319054
32,1.4702638245,2.3158762327\\Version=EM64L-G09RevD.01\State=1-A\HF=-1
235.0603441\RMSD=2.894e-09\RMSF=4.416e-05\Dipole=0.7607333,0.7397751,0
.1560923\Quadrupole=0.9186372,-0.0837953,-0.8348419,1.0645674,-0.40854
22,1.1286781\PG=C01 [X(C1H4S3)]\\@

1\1\GINC-ORC23\FOpt\RM062X\6-311+G(2df,p)\C1H4S3\SNIKOO\27-Jul-2017\0\
\# opt freq m062x/6-311+g(2df,p)\\CH3-SSSH\\0,1\S,-1.2526274354,0.1720

686833,0.038892423\S,0.5463227971,-0.7573683627,-0.2323099805\C,1.7098
273603,0.6104363312,-0.0088809182\H,1.6613257919,0.9822971345,1.013242
2592\H,2.7039846845,0.2030540623,-0.1945181391\H,1.5015479476,1.407362
4476,-0.7189879032\S,-1.560869327,0.2695934327,2.0783190104\H,-1.00518
70491,1.4737079012,2.2936018483\\Version=EM64L-G09RevD.01\State=1-A\HF
=-1235.079073\RMSD=6.212e-09\RMSF=3.840e-05\Dipole=0.7091488,0.6606751
,0.1362724\Quadrupole=0.9474623,-0.1866253,-0.760837,1.0427624,-0.4550
927,0.9913509\PG=C01 [X(C1H4S3)]\\@

1\1\GINC-ORC238\FOpt\RM062X\6-311++G(3df,3pd)\C1H4S3\SNIKOO\27-Jul-201
7\0\\# opt freq m062x/6-311++g(3df,3pd)\\CH3-SSSH\\0,1\S,-1.2484854963
,0.1733000131,0.0424897156\S,0.5434431817,-0.7526530686,-0.2355833415\
C,1.7081526595,0.610135469,-0.0098172836\H,1.6496744402,0.9861607657,1
.0084204707\H,2.7010168269,0.1989745104,-0.1828354018\H,1.5095308351,1
.4031864488,-0.7241662098\S,-1.5580057803,0.2708313271,2.0763010639\H,
-1.0010018967,1.4712161646,2.2945495865\\Version=EM64L-G09RevD.01\Stat
e=1-A\HF=-1235.0853413\RMSD=8.203e-09\RMSF=4.791e-05\Dipole=0.6910843,
0.621156,0.1309384\Quadrupole=1.057386,-0.3309487,-0.7264372,1.0462335
,-0.5359177,0.8911996\PG=C01 [X(C1H4S3)]\\@

CH_3SSSH M08HX

1\1\GINC-ORC225\FOpt\RM08HX\6-31G(d)\C1H4S3\SNIKOO\01-Aug-2017\0\\# op
t freq 6-31g(d) m08hx\\CH3-SSSH\\0,1\S,0.4308051756,-0.8389068682,0.46
90328056\S,-1.3303430651,-0.4307913212,-0.5240645095\C,-1.9062877806,1
.0277474865,0.3895557645\H,-1.2123135949,1.866466248,0.2495823779\H,-2
.8808160779,1.3066613563,-0.0302092315\H,-2.0203839122,0.8039004664,1.
4559968845\S,1.8299322913,0.5202162352,-0.2538003835\H,1.6404649638,1.
4878773969,0.666722292\\Version=EM64L-G16RevA.03\State=1-A\HF=-1234.94
42884\RMSD=7.185e-09\RMSF=1.837e-05\Dipole=-0.433172,0.8945674,0.49038
41\Quadrupole=0.5476505,0.8025806,-1.3502311,-0.6158653,0.0312733,1.28
38592\PG=C01 [X(C1H4S3)]\\@

1\1\GINC-ORC225\FOpt\RM08HX\6-311G(d)\C1H4S3\SNIKOO\01-Aug-2017\0\\# o
pt freq 6-311g(d) m08hx\\CH3-SSSH\\0,1\S,0.4232713191,-0.8433666377,0.
4699727736\S,-1.3430856695,-0.4352727108,-0.5309606488\C,-1.8987761686
,1.0274373944,0.3843320948\H,-1.2103934677,1.8634527689,0.2248689702\H
,-2.8808618112,1.3026103881,-0.0125819281\H,-1.9873853801,0.8072097696
,1.4511214316\S,1.8200744151,0.5316645526,-0.2484933304\H,1.628214763,
1.489435475,0.6845566372\\Version=EM64L-G16RevA.03\State=1-A\HF=-1235.
0510585\RMSD=4.798e-09\RMSF=1.897e-05\Dipole=-0.4299183,0.9041634,0.48
57031\Quadrupole=0.4141824,0.8441923,-1.2583747,-0.6074774,0.0869361,1
.332734\PG=C01 [X(C1H4S3)]\\@

1\1\GINC-ORC220\FOpt\RM08HX\6-311+G(d)\C1H4S3\ROOT\01-Aug-2017\0\\# op
t freq 6-311+g(d) m08hx\\CH3-SSSH\\0,1\S,0.4249792324,-0.8374630793,0.
4750485683\S,-1.3380799006,-0.430226339,-0.5306359664\C,-1.9050634473,
1.0265529993,0.3874941388\H,-1.2130082555,1.8624488709,0.2421064868\H,
-2.8819212031,1.3063887559,-0.0198282666\H,-2.006604566,0.800249084,1.
4522209394\S,1.8309960654,0.5229391475,-0.2528045387\H,1.6397600746,1.
4922815608,0.6692146385\\Version=EM64L-G16RevA.03\State=1-A\HF=-1235.0
55776\RMSD=5.101e-09\RMSF=1.470e-05\Dipole=-0.4117532,0.8708345,0.4778
129\Quadrupole=0.5444209,0.8270044,-1.3714253,-0.5664867,0.066871,1.29
03929\PG=C01 [X(C1H4S3)]\\@

1\1\GINC-ORC220\FOpt\RM08HX\6-311+G(d,p)\C1H4S3\ROOT\01-Aug-2017\0\\#
opt freq 6-311+g(d,p) m08hx\\CH3-SSSH\\0,1\S,0.4271841182,-0.839122384

3,0.4733150074\S,-1.3389661118,-0.4325150169,-0.5261578858\C,-1.902757
7373,1.0280935836,0.3889429929\H,-1.2040371396,1.8581475575,0.24307028
89\H,-2.8762193983,1.3094525132,-0.0249180024\H,-2.008008881,0.8015120
436,1.4531105851\S,1.8273953918,0.5293306092,-0.2553885856\H,1.6264677
58,1.4882720941,0.6708415995\\Version=EM64L-G16RevA.03\State=1-A\HF=-1
235.0631034\RMSD=2.908e-09\RMSF=2.437e-05\Dipole=-0.4059818,0.8550823,
0.4672067\Quadrupole=0.5576868,0.7873135,-1.3450003,-0.6037549,0.03642
62,1.2586436\PG=C01 [X(C1H4S3)]\\@

1\1\GINC-ORC6\FOpt\RM08HX\6-311G(2d,p)\C1H4S3\SNIKOO\01-Aug-2017\0\\#
opt freq 6-311g(2d,p) m08hx\\CH3-SSSH\\0,1\S,0.429556884,-0.8396833018
,0.4711443105\S,-1.3310247008,-0.4314433319,-0.5177047775\C,-1.8965862
745,1.0307449564,0.389507853\H,-1.1942455042,1.8552875402,0.2416721383
\H,-2.86588442,1.3098203525,-0.0333498037\H,-2.0094501225,0.804261209,
1.4510739441\S,1.8084209486,0.533549656,-0.2557881573\H,1.6102711895,1
.4806339196,0.6762604927\\Version=EM64L-G16RevA.03\State=1-A\HF=-1235.
0784478\RMSD=6.433e-09\RMSF=2.348e-05\Dipole=-0.4061552,0.8143747,0.42
00029\Quadrupole=0.5273651,0.7487171,-1.2760821,-0.6513751,-0.0634613,
1.1514455\PG=C01 [X(C1H4S3)]\\@

1\1\GINC-ORC6\FOpt\RM08HX\6-311+G(df,p)\C1H4S3\SNIKOO\01-Aug-2017\0\\#
opt freq 6-311+g(df,p) m08hx\\CH3-SSSH\\0,1\S,0.4241565438,-0.8340129
799,0.4738575379\S,-1.3286466653,-0.4259983615,-0.5264192952\C,-1.9027
087257,1.0272205583,0.3900225123\H,-1.2050014772,1.8606505337,0.257563
3091\H,-2.8733198352,1.3101353807,-0.0293168028\H,-2.01628986,0.795498
809,1.4525142803\S,1.8311482679,0.5148442812,-0.2487757002\H,1.6217197
518,1.4948327785,0.6533701586\\Version=EM64L-G16RevA.03\State=1-A\HF=-
1235.0736439\RMSD=9.014e-09\RMSF=2.808e-05\Dipole=-0.4092192,0.8520024
,0.4626604\Quadrupole=0.4932812,0.8342599,-1.3275412,-0.6422555,-0.011
7221,1.2498604\PG=C01 [X(C1H4S3)]\\@

1\1\GINC-ORC33\FOpt\RM08HX\6-311+G(2df,p)\C1H4S3\SNIKOO\01-Aug-2017\0\
\# opt freq 6-311+g(2df,p) m08hx\\CH3-SSSH\\0,1\S,0.42790085,-0.831360
3702,0.4690237249\S,-1.3208177084,-0.4257411782,-0.513558799\C,-1.8963
8827,1.0283932652,0.3909130319\H,-1.1943841325,1.8560702576,0.25467089
87\H,-2.8625806996,1.3091287427,-0.0389264988\H,-2.0187951098,0.800466
3455,1.4519246288\S,1.8049541891,0.5238875233,-0.2580385069\H,1.611168
8814,1.4823264141,0.6668075204\\Version=EM64L-G16RevA.03\State=1-A\HF=
-1235.0919501\RMSD=7.673e-09\RMSF=3.289e-05\Dipole=-0.3821166,0.780235
1,0.4132753\Quadrupole=0.5727008,0.7254664,-1.2981672,-0.6163507,-0.06
20102,1.1412207\PG=C01 [X(C1H4S3)]\\@

1\1\GINC-ORC29\FOpt\RM08HX\6-311++G(3df,3pd)\C1H4S3\SNIKOO\01-Aug-2017
\0\\# opt freq 6-311++g(3df,3pd) m08hx\\CH3-SSSH\\0,1\S,0.4254631992,-
0.8294749897,0.4667774115\S,-1.3207490184,-0.4270673578,-0.5099640502\
C,-1.8924252482,1.0274659976,0.3921517152\H,-1.1808652188,1.8452042587
,0.263595028\H,-2.8492054477,1.3174051875,-0.0461243943\H,-2.025369912
7,0.7979462226,1.4489575069\S,1.7986617158,0.5252120557,-0.2542626826\
H,1.5955479308,1.4864796253,0.6616854655\\Version=EM64L-G16RevA.03\Sta
te=1-A\HF=-1235.0996202\RMSD=3.185e-09\RMSF=1.953e-05\Dipole=-0.380843
2,0.7591704,0.3878247\Quadrupole=0.7070829,0.6553867,-1.3624696,-0.703
7729,-0.1603817,1.045673\PG=C01 [X(C1H4S3)]\\@

CH_3SSSH QCISD

1\1\GINC-ORC59\FOpt\RQCISD-FC\6-311+G(2df,p)\C1H4S3\SNIKOO\25-Aug-2017

```
\0\\# opt 6-311+g(2df,p) qcisd\\CH3-SSSH\\0,1\S,0.4349534818,-0.809599
1163,0.4913695182\S,-1.3014958516,-0.3968278047,-0.529685033\C,-1.9444
163038,1.026102109,0.3951180978\H,-1.2806852056,1.8854258494,0.2860318
847\H,-2.9156233958,1.2702527447,-0.0430167412\H,-2.0733391288,0.77544
92178,1.4483539859\S,1.8816261994,0.4828106212,-0.2542106948\H,1.69648
42044,1.4911103788,0.6188629824\\Version=EM64L-G09RevD.01\State=1-A\HF
=-1232.8415577\MP2=-1233.5234118\MP3=-1233.5788185\MP4D=-1233.5981632\
MP4DQ=-1233.5764865\MP4SDQ=-1233.5817407\QCISD=-1233.5815807\RMSD=6.40
2e-09\RMSF=1.107e-06\Dipole=-0.3969343,0.7839181,0.3972771\PG=C01 [X(C
1H4S3)]\\@
```

CH_3SSS^- B3LYP

```
1\1\GINC-ORC218\FOpt\RB3LYP\6-31G(d)\C1H3S3(1-)\ROOT\27-Jul-2017\0\\#
 opt freq rb3lyp/6-31g(d)\\CH3-SSS-\\-1,1\S,-1.3869228922,0.0739470191,
 0.0021196885\S,0.577439773,-0.8139411368,-0.2913315518\C,1.643924226,0
 .6487618441,0.0000910008\H,1.318397403,1.1285632721,0.9297066731\H,2.6
 806645931,0.3086061524,0.1090223355\H,1.5771101384,1.3667124936,-0.824
 1027308\S,-1.6266447212,0.6701470755,1.9733081747\\Version=EM64L-G09Re
 vD.01\State=1-A\HF=-1234.5382252\RMSD=8.235e-09\RMSF=6.314e-06\Dipole=
 1.5176555,0.0340605,-1.0675396\Quadrupole=0.276778,1.6143646,-1.891142
 6,2.8791631,4.3529114,-2.4259014\PG=C01 [X(C1H3S3)]\\@
```

```
1\1\GINC-ORC246\FOpt\RB3LYP\6-311G(d)\C1H3S3(1-)\SNIKOO\27-Jul-2017\0\
 \# opt freq rb3lyp/6-311g(d)\\CH3-SSS-\\-1,1\S,-1.3830170659,0.0901397
 418,-0.0179848018\S,0.5867877313,-0.819411914,-0.2555509968\C,1.650296
 9227,0.6477648784,0.0044056482\H,1.3645082801,1.1259317657,0.943375301
 8\H,2.6891966642,0.3125768135,0.0700038478\H,1.5438884269,1.3613187338
 ,-0.8149895424\S,-1.6676924393,0.6644767008,1.9695541331\\Version=EM64
 L-G09RevD.01\State=1-A\HF=-1234.6337065\RMSD=4.850e-09\RMSF=5.021e-06\
 Dipole=1.6929819,-0.0067887,-1.1939064\Quadrupole=0.1827346,1.6400439,
 -1.8227785,3.0151135,4.687175,-2.5419645\PG=C01 [X(C1H3S3)]\\@
```

```
1\1\GINC-ORC270\FOpt\RB3LYP\6-311+G(d)\C1H3S3(1-)\SNIKOO\27-Jul-2017\0
 \\# opt freq rb3lyp/6-311+g(d)\\Title Card Required\\-1,1\S,0.53476633
 18,-0.7951594105,0.4569337917\S,-1.4164955134,-0.4667449799,-0.4491957
 606\C,-1.9092813149,1.0919735712,0.3760773426\H,-1.1091833822,1.825046
 5244,0.253395179\H,-2.817094595,1.4638049664,-0.1079559323\H,-2.098959
 5192,0.933733706,1.4398808381\S,1.9038879929,0.6176016223,-0.233606458
 4\\Version=EM64L-G09RevD.01\State=1-A\HF=-1234.6379225\RMSD=3.797e-09\
 RMSF=7.596e-06\Dipole=-1.9755448,0.1031526,0.4202724\Quadrupole=-5.796
 639,2.8561806,2.9404584,-4.0078087,0.1134775,0.0802548\PG=C01 [X(C1H3S
 3)]\\@
1\1\GINC-ORC188\FOpt\RB3LYP\6-311+G(d,p)\C1H3S3(1-)\SNIKOO\27-Jul-2017
 \0\\# opt freq rb3lyp/6-311+g(d,p)\\CH3-SSS-\\-1,1\S,-1.3843586958,0.0
 962825734,-0.0114672231\S,0.5799873746,-0.8091451799,-0.2472020044\C,1
 .6577961555,0.6516883393,0.0013245279\H,1.3791364147,1.1378434702,0.93
 89612899\H,2.6921013238,0.301911844,0.0659915974\H,1.5572617519,1.3594
 305126,-0.8242475803\S,-1.6979558047,0.6447851604,1.9754529826\\Versio
 n=EM64L-G09RevD.01\State=1-A\HF=-1234.6422521\RMSD=8.591e-09\RMSF=5.21
 8e-06\Dipole=1.6372299,-0.0041604,-1.1748226\Quadrupole=0.1564209,1.77
 1437,-1.9278579,2.8915119,4.7842664,-2.5108391\PG=C01 [X(C1H3S3)]\\@
```

```
1\1\GINC-ORC273\FOpt\RB3LYP\6-311G(2d,p)\C1H3S3(1-)\SNIKOO\27-Jul-2017
 \0\\# opt freq rb3lyp/6-311g(2d,p)\\CH3-SSS-\\-1,1\S,-1.3733783555,0.0
 93194734,-0.0144324934\S,0.5661219393,-0.8004805536,-0.2401574021\C,1.
 6530302234,0.6545062354,0.0027272044\H,1.3694028647,1.1402984792,0.937
```

3509535\H,2.684508459,0.2997110648,0.0724142577\H,1.5561440324,1.35635
74904,-0.8262958441\S,-1.6718606432,0.6392092698,1.967206914\\Version=
EM64L-G09RevD.01\State=1-A\HF=-1234.6541524\RMSD=6.954e-09\RMSF=1.322e
-05\Dipole=1.6464212,-0.0056233,-1.1450394\Quadrupole=0.0160926,1.6594
059,-1.6754985,2.742326,4.5922565,-2.3189154\PG=C01 [X(C1H3S3)]\\@

1\1\GINC-ORC271\FOpt\RB3LYP\6-311+G(df,p)\C1H3S3(1-)\SNIKOO\27-Jul-201
7\0\\# opt freq rb3lyp/6-311+g(df,p)\\CH3-SSS-\\-1,1\S,-1.3748964755,0
.0976574888,-0.0094050377\S,0.568412699,-0.8000073224,-0.2341832813\C,
1.658729911,0.6524443536,0.0012958504\H,1.3918902796,1.1450002851,0.93
88809304\H,2.6910410331,0.2960213176,0.0582532742\H,1.5569606223,1.358
8266718,-0.8253993325\S,-1.7081695495,0.6328539254,1.9693711865\\Versi
on=EM64L-G09RevD.01\State=1-A\HF=-1234.6512666\RMSD=3.780e-09\RMSF=1.5
72e-05\Dipole=1.6580321,-0.0120192,-1.1992206\Quadrupole=0.0663483,1.9
058232,-1.9721715,2.9107703,4.8205102,-2.5376046\PG=C01 [X(C1H3S3)]\\@

1\1\GINC-ORC268\FOpt\RB3LYP\6-311+G(2df,p)\C1H3S3(1-)\SNIKOO\27-Jul-20
17\0\\# opt freq rb3lyp/6-311+g(2df,p)\\CH3-SSS-\\-1,1\S,-1.3650931731
,0.0990033298,-0.0081398909\S,0.5529603125,-0.7848490189,-0.2179492269
\C,1.6566914192,0.6557593026,0.0021042663\H,1.3991468377,1.1532978938,
0.9383942472\H,2.6848760908,0.2878530112,0.0555605948\H,1.5572353846,1
.3556166595,-0.8289827357\S,-1.7018483516,0.6161155419,1.9578263353\\V
ersion=EM64L-G09RevD.01\State=1-A\HF=-1234.6664043\RMSD=8.525e-09\RMSF
=1.331e-05\Dipole=1.6171781,-0.0144116,-1.1413212\Quadrupole=-0.082963
2,1.8482929,-1.7653298,2.6822223,4.6795548,-2.3305394\PG=C01 [X(C1H3S3
)]\\@

1\1\GINC-ORC258\FOpt\RB3LYP\6-311++G(3df,3pd)\C1H3S3(1-)\SNIKOO\27-Jul
-2017\0\\# opt freq rb3lyp/6-311++g(3df,3pd)\\CH3-SSS-\\-1,1\S,-1.3574
098151,0.0984963403,-0.0069446678\S,0.5508339608,-0.7800420633,-0.2079
883267\C,1.6567594139,0.6557366002,0.0032627595\H,1.4118190241,1.15210
39343,0.9417036433\H,2.6831967351,0.2863482354,0.0443234849\H,1.548727
9358,1.3558128782,-0.8243954971\S,-1.7099587347,0.6143407948,1.9488521
94\\Version=EM64L-G09RevD.01\State=1-A\HF=-1234.670648\RMSD=4.212e-09\
RMSF=4.123e-06\Dipole=1.5853183,-0.0163251,-1.0910704\Quadrupole=-0.22
55779,1.8607173,-1.6351394,2.6015873,4.5640971,-2.2733203\PG=C01 [X(C1
H3S3)]\\@

CH_3SSS^- B3PW91

1\1\GINC-ORC218\FOpt\RB3LYP\6-31G(d)\C1H3S3(1-)\ROOT\27-Jul-2017\0\\#
opt freq rb3lyp/6-31g(d)\\CH3-SSS-\\-1,1\S,-1.3869228922,0.0739470191,
0.0021196885\S,0.577439773,-0.8139411368,-0.2913315518\C,1.643924226,0
.6487618441,0.0000910008\H,1.318397403,1.1285632721,0.9297066731\H,2.6
806645931,0.3086061524,0.1090223355\H,1.5771101384,1.3667124936,-0.824
1027308\S,-1.6266447212,0.6701470755,1.9733081747\\Version=EM64L-G09Re
vD.01\State=1-A\HF=-1234.5382252\RMSD=8.235e-09\RMSF=6.314e-06\Dipole=
1.5176555,0.0340605,-1.0675396\Quadrupole=0.276778,1.6143646,-1.891142
6,2.8791631,4.3529114,-2.4259014\PG=C01 [X(C1H3S3)]\\@

1\1\GINC-ORC20\FOpt\RB3PW91\6-31G(d)\C1H3S3(1-)\SNIKOO\28-Jul-2017\0\\
opt freq rb3pw91/6-31g(d)\\CH3-SSS-\\-1,1\S,0.5328456632,-0.83326592
55,0.4058986974\S,-1.4014265095,-0.4376503455,-0.432787908\C,-1.799028
6118,1.1365218038,0.3930323102\H,-0.9321267195,1.800727225,0.293814074
5\H,-2.6620125859,1.5886408335,-0.1104107758\H,-2.026830102,0.99046912
79,1.45443816\S,1.8872863854,0.5798226608,-0.2255073183\\Version=EM64L
-G09RevD.01\State=1-A\HF=-1234.3736635\RMSD=3.822e-09\RMSF=3.555e-05\D

```
ipole=-1.8394206,0.2007095,0.4127891\Quadrupole=-5.5218153,2.8501971,2
.6716182,-3.5586636,-0.0635312,0.1927976\PG=C01 [X(C1H3S3)]\\@

1\1\GINC-ORC210\FOpt\RB3PW91\6-311G(d)\C1H3S3(1-)\SNIKOO\28-Jul-2017\0
\\# opt freq rb3pw91/6-311g(d)\\CH3-SSS-\\-1,1\S,0.5211130222,-0.83355
60333,0.4297204455\S,-1.3952308387,-0.4187032256,-0.4558367237\C,-1.79
9492902,1.1401684675,0.3871739161\H,-0.9573260914,1.8275646484,0.27211
70571\H,-2.6855959955,1.5724156797,-0.087524291\H,-1.992984826,0.97758
64196,1.4501834226\S,1.9082251513,0.5597894237,-0.2173565867\\Version=
EM64L-G09RevD.01\State=1-A\HF=-1234.4619459\RMSD=6.278e-09\RMSF=3.963e
-05\Dipole=-2.0459744,0.1749209,0.4196891\Quadrupole=-5.9059292,3.0668
54,2.8390752,-3.674917,-0.0599995,0.0828163\PG=C01 [X(C1H3S3)]\\@

1\1\GINC-ORC28\FOpt\RB3PW91\6-311+G(d)\C1H3S3(1-)\SNIKOO\28-Jul-2017\0
\\# opt freq rb3pw91/6-311+g(d)\\CH3-SSS-\\-1,1\S,0.5272505887,-0.8261
790217,0.4299149043\S,-1.3860423893,-0.4138645353,-0.4527750957\C,-1.8
053810018,1.1416795673,0.3902741572\H,-0.9663645239,1.8348494305,0.284
7878133\H,-2.6890797225,1.5703308929,-0.0928970707\H,-2.0106103587,0.9
778547458,1.4512709628\S,1.9289349274,0.5405943005,-0.2320984312\\Vers
ion=EM64L-G09RevD.01\State=1-A\HF=-1234.4663338\RMSD=6.040e-09\RMSF=3.
131e-05\Dipole=-1.9817956,0.16965,0.4233774\Quadrupole=-5.9134669,3.05
39061,2.8595608,-3.6762019,0.0878561,0.1182104\PG=C01 [X(C1H3S3)]\\@

1\1\GINC-ORC23\FOpt\RB3PW91\6-311+G(d,p)\C1H3S3(1-)\SNIKOO\28-Jul-2017
\0\\# opt freq rb3pw91/6-311+g(d,p)\\CH3-SSS-\\-1,1\S,0.5280338119,-0.
828319418,0.4271227987\S,-1.3873857912,-0.4144044333,-0.4500705504\C,-
1.8052216688,1.1432521534,0.391936936\H,-0.9621201888,1.8319463931,0.2
857903495\H,-2.6865288593,1.5707114019,-0.096122255\H,-2.0130356116,0.
9775984904,1.4520713767\S,1.9249658278,0.5444807925,-0.2322514155\\Ver
sion=EM64L-G09RevD.01\State=1-A\HF=-1234.4706832\RMSD=7.959e-09\RMSF=6
.890e-06\Dipole=-1.9705577,0.1573666,0.4199888\Quadrupole=-5.8998985,3
.0283658,2.8715327,-3.6433507,0.1092247,0.1025875\PG=C01 [X(C1H3S3)]\\
@

1\1\GINC-ORC12\FOpt\RB3PW91\6-311G(2d,p)\C1H3S3(1-)\SNIKOO\28-Jul-2017
\0\\# opt freq rb3pw91/6-311g(2d,p)\\CH3-SSS-\\-1,1\S,0.5176411963,-0.
826712998,0.4245959262\S,-1.3722430503,-0.4145485155,-0.4424999514\C,-
1.7989948997,1.1430239558,0.3949841869\H,-0.9496247234,1.8227368878,0.
2935418226\H,-2.6723409976,1.5723381584,-0.1043425096\H,-2.0187228782,
0.9751550421,1.450902608\S,1.8929928729,0.5532728494,-0.2387048426\\Ve
rsion=EM64L-G09RevD.01\State=1-A\HF=-1234.4844456\RMSD=6.427e-09\RMSF=
3.127e-05\Dipole=-1.9519614,0.1798583,0.4018192\Quadrupole=-5.6831573,
2.9684346,2.7147227,-3.3506952,0.1010515,0.0337773\PG=C01 [X(C1H3S3)]\
\@

1\1\GINC-ORC117\FOpt\RB3PW91\6-311G(df,p)\C1H3S3(1-)\SNIKOO\11-Sep-201
8\0\\# opt freq=noraman b3pw91/6-311g(df,p)\\CH3-SSS-\\-1,1\S,0.531500
3729,-0.838311976,0.4125738212\S,-1.3639256442,-0.4195240413,-0.464029
2052\C,-1.7940913383,1.1215651684,0.3987320575\H,-0.958515359,1.819669
8378,0.2990273539\H,-2.6827961454,1.5469182647,-0.076566703\H,-1.99151
85058,0.9383499116,1.4577016277\S,1.9183176198,0.5534348349,-0.2156959
52\\Version=EM64L-G09RevE.01\State=1-A\HF=-1234.4758853\RMSD=8.963e-09
\RMSF=1.624e-05\Dipole=-2.0700747,0.1424486,0.4202276\Quadrupole=-6.00
29848,3.028225,2.9747598,-3.7137643,-0.1128329,0.1375626\PG=C01 [X(C1H
3S3)]\\@
```

1\1\GINC-ORC20\FOpt\RB3PW91\6-311+G(df,p)\C1H3S3(1-)\SNIKOO\28-Jul-201
7\0\\# opt freq rb3pw91/6-311+g(df,p)\\CH3-SSS-\\-1,1\S,0.5220379233,-
0.8217538086,0.4260003148\S,-1.3699334502,-0.407225243,-0.4495427172\C
,-1.8062065913,1.1445512666,0.392287343\H,-0.9714212599,1.8435303106,0
.2892620398\H,-2.6919961275,1.5626984479,-0.0954145641\H,-2.0131309103
,0.976097893,1.4522454049\S,1.929357936,0.5273665136,-0.2363605811\\Ve
rsion=EM64L-G09RevD.01\State=1-A\HF=-1234.4802181\RMSD=8.539e-09\RMSF=
1.243e-05\Dipole=-1.9988129,0.1517683,0.4250906\Quadrupole=-6.0184779,
3.0469847,2.9714931,-3.6274423,0.118225,0.1734625\PG=C01 [X(C1H3S3)]\\
@

1\1\GINC-ORC18\FOpt\RB3PW91\6-311+G(2df,p)\C1H3S3(1-)\SNIKOO\28-Jul-20
17\0\\# opt freq rb3pw91/6-311+g(2df,p)\\CH3-SSS-\\-1,1\S,0.5162718406
,-0.8161500712,0.4235883296\S,-1.3472738085,-0.3983199617,-0.443342783
9\C,-1.8030521746,1.1457201264,0.3958190843\H,-0.9714000575,1.84795712
14,0.2999137972\H,-2.6857162413,1.5573710377,-0.1027760912\H,-2.022012
7257,0.9720896396,1.4515263583\S,1.9118906869,0.5165974876,-0.24625145
43\\Version=EM64L-G09RevD.01\State=1-A\HF=-1234.4970501\RMSD=5.642e-09
\RMSF=4.117e-05\Dipole=-1.9190996,0.1679831,0.3961238\Quadrupole=-5.79
30378,2.9653363,2.8277014,-3.2936918,0.1881881,0.0985992\PG=C01 [X(C1H
3S3)]\\@

1\1\GINC-ORC20\FOpt\RB3PW91\6-311++G(3df,3pd)\C1H3S3(1-)\SNIKOO\28-Jul
-2017\0\\# opt freq rb3pw91/6-311++g(3df,3pd)\\CH3-SSS-\\-1,1\S,0.5098
97626,-0.8126344297,0.419114608\S,-1.3413973301,-0.3916604185,-0.44664
70379\C,-1.8016896269,1.1455046537,0.3952906412\H,-0.9764403741,1.8528
024531,0.2961875263\H,-2.6887156147,1.5512065607,-0.0972230092\H,-2.01
25215312,0.9681086624,1.4505463666\S,1.909574371,0.5119378982,-0.23879
1855\\Version=EM64L-G09RevD.01\State=1-A\HF=-1234.5009718\RMSD=5.816e-
09\RMSF=9.527e-06\Dipole=-1.860082,0.1828681,0.3681115\Quadrupole=-5.6
784902,2.8665674,2.8119228,-3.1273939,0.1338631,0.0886238\PG=C01 [X(C1
H3S3)]\\@

CH_3SSS^- wB97XD

1\1\GINC-ORC22\FOpt\RwB97XD\6-31G(d)\C1H3S3(1-)\SNIKOO\28-Jul-2017\0\\
opt freq rwb97xd/6-31g(d)\\CH3-SSS-\\-1,1\S,0.508363423,-0.845005844
8,0.4059323901\S,-1.3951760931,-0.4368005337,-0.4372572252\C,-1.783973
9532,1.1351393948,0.3901931886\H,-0.9185374054,1.7988181111,0.28234003
98\H,-2.6527238512,1.583803331,-0.1035446079\H,-1.9983061004,0.9843230
328,1.4525783937\S,1.8390615003,0.6049878888,-0.211764939\\Version=EM6
4L-G09RevD.01\State=1-A\HF=-1234.4635593\RMSD=6.804e-09\RMSF=2.309e-05
\Dipole=-1.9446004,0.1516793,0.4121831\Quadrupole=-5.4217148,2.7773255
,2.6443893,-3.7323117,-0.063876,0.2425891\PG=C01 [X(C1H3S3)]\\@

1\1\GINC-ORC20\FOpt\RwB97XD\6-311G(d)\C1H3S3(1-)\SNIKOO\28-Jul-2017\0\
\# opt freq rwb97xd/6-311g(d)\\CH3-SSS-\\-1,1\S,0.4977648149,-0.845669
9784,0.4302061886\S,-1.3888193981,-0.4218980521,-0.4550092657\C,-1.784
1800928,1.1380015988,0.3851751565\H,-0.939244162,1.8205374459,0.266521
2678\H,-2.6696775943,1.5705659943,-0.0879936547\H,-1.9740958455,0.9744
881752,1.4478205223\S,1.8569597978,0.5892401964,-0.2082429748\\Version
=EM64L-G09RevD.01\State=1-A\HF=-1234.5522358\RMSD=4.925e-09\RMSF=3.370
e-05\Dipole=-2.1664484,0.1192508,0.4233399\Quadrupole=-5.8014825,3.014
3705,2.787112,-3.8757107,-0.0187455,0.1412324\PG=C01 [X(C1H3S3)]\\@

1\1\GINC-ORC18\FOpt\RwB97XD\6-311+G(d)\C1H3S3(1-)\SNIKOO\28-Jul-2017\0
\\# opt freq rwb97xd/6-311+g(d)\\CH3-SSS-\\-1,1\S,0.503177401,-0.83781

```
91928,0.4332039888\S,-1.3793995415,-0.4155129696,-0.4546150399\C,-1.79
03629572,1.1395726381,0.387087931\H,-0.951555524,1.8310516684,0.274949
8792\H,-2.6768532621,1.5662535489,-0.0903297714\H,-1.9866928814,0.9739
500365,1.4485765669\S,1.8803942851,0.5677696503,-0.2203963145\\Version
=EM64L-G09RevD.01\State=1-A\HF=-1234.5565527\RMSD=7.050e-09\RMSF=4.200
e-05\Dipole=-2.1164089,0.1166248,0.4223952\Quadrupole=-5.7748551,3.007
9439,2.7669113,-3.8882387,0.1081213,0.1855745\PG=C01 [X(C1H3S3)]\\@

1\1\GINC-ORC15\FOpt\RwB97XD\6-311+G(d,p)\C1H3S3(1-)\SNIKOO\28-Jul-2017
 \0\\# opt freq rwb97xd/6-311+g(d,p)\\CH3-SSS-\\-1,1\S,0.503218554,-0.8
 408714977,0.4292977207\S,-1.3837377339,-0.4188876456,-0.450919112\C,-1
 .7882960992,1.1401750156,0.388242613\H,-0.9428209413,1.824040404,0.274
 7495625\H,-2.6709169489,1.5684323922,-0.0941204548\H,-1.9874827699,0.9
 744676165,1.4490577924\S,1.8687434592,0.577909095,-0.2178308818\\Versi
 on=EM64L-G09RevD.01\State=1-A\HF=-1234.560736\RMSD=8.611e-09\RMSF=1.08
 1e-04\Dipole=-2.095826,0.0991117,0.4166618\Quadrupole=-5.7347025,2.964
 8816,2.7698209,-3.8743702,0.1222504,0.1703956\PG=C01 [X(C1H3S3)]\\@

1\1\GINC-ORC15\FOpt\RwB97XD\6-311G(2d,p)\C1H3S3(1-)\SNIKOO\28-Jul-2017
 \0\\# opt freq rwb97xd/6-311g(2d,p)\\CH3-SSS-\\-1,1\S,0.4955673418,-0.
 8403092984,0.4263116861\S,-1.3708406937,-0.4152604413,-0.4496683929\C,
 -1.7844530351,1.1409166331,0.3893241681\H,-0.9394355789,1.8229131165,0
 .2732671403\H,-2.667417143,1.565606596,-0.094542527\H,-1.9838765966,0.
 972480739,1.4482796723\S,1.8491632255,0.5789180351,-0.2144945068\\Vers
 ion=EM64L-G09RevD.01\State=1-A\HF=-1234.5746393\RMSD=3.178e-09\RMSF=2.
 434e-05\Dipole=-2.0455224,0.1391181,0.3953996\Quadrupole=-5.5923521,2.
 9380253,2.6543268,-3.5259318,0.0677724,0.0601692\PG=C01 [X(C1H3S3)]\\@

1\1\GINC-ORC117\FOpt\RwB97XD\6-311G(df,p)\C1H3S3(1-)\SNIKOO\11-Sep-201
 8\0\\# opt freq=noraman wb97xd/6-311g(df,p)\\CH3-SSS-\\-1,1\S,0.510188
 8618,-0.851059171,0.4142894652\S,-1.3626323077,-0.4257064243,-0.462950
 5664\C,-1.7781902949,1.117936067,0.3952452808\H,-0.9380334538,1.809099
 1584,0.2897173006\H,-2.6657551011,1.5464683877,-0.0769449784\H,-1.9706
 466695,0.9370740779,1.4547277458\S,1.8640399652,0.5882899043,-0.202341
 2475\\Version=EM64L-G09RevE.01\State=1-A\HF=-1234.5663201\RMSD=8.153e-
 09\RMSF=2.084e-05\Dipole=-2.1670499,0.0877937,0.4201793\Quadrupole=-5.
 8672436,2.9487308,2.9185127,-3.9271411,-0.0990465,0.1871677\PG=C01 [X(
 C1H3S3)]\\@

1\1\GINC-ORC12\FOpt\RwB97XD\6-311+G(df,p)\C1H3S3(1-)\SNIKOO\28-Jul-201
 7\0\\# opt freq rwb97xd/6-311+g(df,p)\\CH3-SSS-\\-1,1\S,0.4994217176,-
 0.83494021,0.428398116\S,-1.3684582546,-0.4104978744,-0.452030668\C,-1
 .7905763255,1.1412976144,0.388056296\H,-0.9551174908,1.8374357956,0.27
 64526754\H,-2.679309934,1.5600031213,-0.0914521366\H,-1.9864851815,0.9
 736110633,1.4494062125\S,1.8792329888,0.5583558698,-0.2203532552\\Vers
 ion=EM64L-G09RevD.01\State=1-A\HF=-1234.5705814\RMSD=5.393e-09\RMSF=2.
 881e-05\Dipole=-2.1132254,0.1030434,0.4212636\Quadrupole=-5.8630852,2.
 9882434,2.8748418,-3.8502746,0.1045486,0.23449\PG=C01 [X(C1H3S3)]\\@

1\1\GINC-ORC353\FOpt\RwB97XD\6-311+G(2df,p)\C1H3S3(1-)\SNIKOO\28-Jul-2
 017\0\\# opt freq rwb97xd/6-311+g(2df,p)\\CH3-SSS-\\-1,1\S,0.494653486
 7,-0.8302755606,0.4251011961\S,-1.3486856352,-0.3993662181,-0.45285213
 92\C,-1.7884770414,1.1425055032,0.3895790207\H,-0.9608155716,1.8464166
 114,0.2782510048\H,-2.6808913609,1.5511614471,-0.0915483235\H,-1.98532
 40775,0.9685732528,1.4486894659\S,1.8682477199,0.5462503443,-0.2187429
 848\\Version=EM64L-G09RevD.01\State=1-A\HF=-1234.5873766\RMSD=9.911e-0
 9\RMSF=3.906e-05\Dipole=-2.0216601,0.129585,0.384977\Quadrupole=-5.655
```

4938,2.9273206,2.7281732,-3.5110585,0.1154875,0.1377407\PG=C01 [X(C1H3
S3)]\\@

1\1\GINC-ORC265\FOpt\RwB97XD\6-311++G(3df,3pd)\C1H3S3(1-)\SNIKOO\28-Ju
l-2017\0\\# opt freq rwb97xd/6-311++g(3df,3pd)\\CH3-SSS-\\-1,1\S,0.490
3029535,-0.826300716,0.421004322\S,-1.3450649861,-0.3945649222,-0.4530
459287\C,-1.7873114037,1.1420563398,0.38952876\H,-0.9641933236,1.84878
51927,0.276992124\H,-2.6810607445,1.547288049,-0.088146907\H,-1.979885
622,0.9666240874,1.4474547829\S,1.8659206464,0.5413773493,-0.215309913
2\\Version=EM64L-G09RevD.01\State=1-A\HF=-1234.5913282\RMSD=5.544e-09\
RMSF=4.194e-05\Dipole=-1.9591543,0.1542261,0.3618037\Quadrupole=-5.516
2113,2.816126,2.7000853,-3.3533518,0.0668246,0.1256129\PG=C01 [X(C1H3S
3)]\\@

CH_3SSS^- M062X

1\1\GINC-ORC66\FOpt\RM062X\6-31G(d)\C1H3S3(1-)\SNIKOO\27-Jul-2017\0\\#
 opt freq 6-31g(d) m062x\\CH3-SSS-\\-1,1\S,-1.3405014481,0.0594778294,
-0.0214947982\S,0.5714205679,-0.8169913936,-0.2914509901\C,1.605412529
8,0.6451421272,0.0132333118\H,1.2564970734,1.1096820354,0.9423946789\H
,2.6440549622,0.3216544445,0.1292193303\H,1.528044186,1.3626365071,-0.
8076379818\S,-1.4809593512,0.7011951701,1.934550039\\Version=EM64L-G09
RevD.01\State=1-A\HF=-1234.401874\RMSD=7.138e-09\RMSF=6.132e-05\Dipole
=1.5286614,-0.04544,-1.143057\Quadrupole=0.6618828,1.4468524,-2.108735
2,2.8243458,4.054168,-2.5131598\PG=C01 [X(C1H3S3)]\\@

1\1\GINC-ORC53\FOpt\RM062X\6-311G(d)\C1H3S3(1-)\SNIKOO\27-Jul-2017\0\\
opt freq m062x/6-311g(d)\\CH3-SSS-\\-1,1\S,-1.3345911547,0.071243747
,-0.0445570314\S,0.5787440309,-0.8247329235,-0.2657187652\C,1.60694301
48,0.643125993,0.0197575515\H,1.2895013044,1.1026323918,0.9581466745\H
,2.6486385729,0.3270481728,0.0986074383\H,1.4933498802,1.3583843449,-0
.7951130209\S,-1.4986171285,0.7050949939,1.9276907433\\Version=EM64L-G
09RevD.01\State=1-A\HF=-1234.506117\RMSD=6.003e-09\RMSF=4.461e-05\Dipo
le=1.7001129,-0.1062478,-1.2908756\Quadrupole=0.7111616,1.4378909,-2.1
490525,3.0003152,4.3965099,-2.6428444\PG=C01 [X(C1H3S3)]\\@

1\1\GINC-ORC48\FOpt\RM062X\6-311+G(d)\C1H3S3(1-)\SNIKOO\27-Jul-2017\0\
\# opt freq m062x/6-311+g(d)\\CH3-SSS-\\-1,1\S,-1.3361797251,0.0757001
717,-0.0382196624\S,0.5740722457,-0.8183154581,-0.2656005271\C,1.61037
81401,0.6444685762,0.0165737427\H,1.2921056141,1.1110993371,0.95162296
57\H,2.6494817352,0.3211157195,0.1029784312\H,1.5084064212,1.356987233
1,-0.802721339\S,-1.5142959112,0.6917411405,1.9341799788\\Version=EM64
L-G09RevD.01\State=1-A\HF=-1234.5106414\RMSD=4.922e-09\RMSF=4.736e-05\
Dipole=1.6308551,-0.0941805,-1.2542878\Quadrupole=0.8086895,1.4880812,
-2.2967707,2.8961703,4.3611314,-2.5772899\PG=C01 [X(C1H3S3)]\\@

1\1\GINC-ORC66\FOpt\RM062X\6-311+G(d,p)\C1H3S3(1-)\SNIKOO\27-Jul-2017\
0\\# opt freq m062x/6-311+g(d,p)\\CH3-SSS-\\-1,1\S,-1.336719149,0.0743
81883,-0.0376694856\S,0.5742641065,-0.8169018712,-0.2673199396\C,1.610
9347463,0.6461410405,0.0166573481\H,1.2883013976,1.1110588,0.951547307
9\H,2.6486714299,0.3192863154,0.1051632201\H,1.5095510599,1.3569383791
,-0.8042376727\S,-1.5110350712,0.6918921732,1.9346728117\\Version=EM64
L-G09RevD.01\State=1-A\HF=-1234.5138652\RMSD=6.974e-09\RMSF=4.004e-05\
Dipole=1.6209907,-0.0981134,-1.2547212\Quadrupole=0.7887744,1.4941404,
-2.2829147,2.8627319,4.3619581,-2.5735542\PG=C01 [X(C1H3S3)]\\@

```
1\1\GINC-ORC195\FOpt\RM062X\6-311G(2d,p)\C1H3S3(1-)\SNIKOO\27-Jul-2017
 \0\\# opt freq m062x/6-311g(2d,p)\\CH3-SSS-\\-1,1\S,-1.329738055,0.080
 9264996,-0.0381836622\S,0.5629442665,-0.8085232689,-0.2624767793\C,1.6
 08571616,0.64890243,0.0165612437\H,1.2763199035,1.1157648034,0.9453934
 912\H,2.6424684584,0.3165567044,0.116299394\H,1.5154824012,1.350815800
 1,-0.81063766\S,-1.4920800706,0.6783537515,1.9318575625\\Version=EM64L
 -G09RevD.01\State=1-A\HF=-1234.5269532\RMSD=8.463e-09\RMSF=9.233e-05\D
 ipole=1.6180881,-0.076971,-1.2046942\Quadrupole=0.5691871,1.4254666,-1
 .9946537,2.6949582,4.2878137,-2.3999857\PG=C01 [X(C1H3S3)]\\@
```

```
1\1\GINC-ORC116\FOpt\RM062X\6-311G(df,p)\C1H3S3(1-)\SNIKOO\11-Sep-2018
 \0\\# opt freq=noraman m062x/6-311g(df,p)\\CH3-SSS-\\-1,1\S,0.49113459
 ,-0.8537212745,0.4167355604\S,-1.3752033026,-0.4370809148,-0.467071000
 9\C,-1.7631959496,1.1139125536,0.3901770169\H,-0.9112613335,1.78752682
 81,0.2719088116\H,-2.6499203172,1.552638262,-0.0706081535\H,-1.9412242
 327,0.9326237875,1.4507909119\S,1.8086415455,0.626202758,-0.1801901463
 \\Version=EM64L-G09RevE.01\State=1-A\HF=-1234.5199592\RMSD=8.658e-09\R
 MSF=3.084e-05\Dipole=-2.1059659,0.0578875,0.3937682\Quadrupole=-5.6573
 092,2.7991934,2.8581158,-3.994236,-0.1939532,0.1737538\PG=C01 [X(C1H3S
 3)]\\@
```

```
1\1\GINC-ORC66\FOpt\RM062X\6-311+G(df,p)\C1H3S3(1-)\SNIKOO\27-Jul-2017
 \0\\# opt freq m062x/6-311+g(df,p)\\CH3-SSS-\\-1,1\S,-1.3300354726,0.0
 759199796,-0.035603471\S,0.5651264706,-0.8097774005,-0.2567082718\C,1.
 6126509139,0.6458103494,0.0168427098\H,1.3017967077,1.1155081833,0.953
 3978822\H,2.6498103328,0.3146826759,0.0958987957\H,1.5082617062,1.3577
 392252,-0.803203108\S,-1.5236421387,0.6829137071,1.928189053\\Version=
 EM64L-G09RevD.01\State=1-A\HF=-1234.5244748\RMSD=3.323e-09\RMSF=2.700e
 -05\Dipole=1.6293993,-0.0975751,-1.2612895\Quadrupole=0.6983515,1.6091
 413,-2.3074928,2.9044372,4.3689683,-2.6356599\PG=C01 [X(C1H3S3)]\\@
```

```
1\1\GINC-ORC23\FOpt\RM062X\6-311+G(2df,p)\C1H4S3\SNIKOO\27-Jul-2017\0\
 \# opt freq m062x/6-311+g(2df,p)\\CH3-SSSH\\0,1\S,-1.2526274354,0.1720
 686833,0.038892423\S,0.5463227971,-0.7573683627,-0.2323099805\C,1.7098
 273603,0.6104363312,-0.0088809182\H,1.6613257919,0.9822971345,1.013242
 2592\H,2.7039846845,0.2030540623,-0.1945181391\H,1.5015479476,1.407362
 4476,-0.7189879032\S,-1.560869327,0.2695934327,2.0783190104\H,-1.00518
 70491,1.4737079012,2.2936018483\\Version=EM64L-G09RevD.01\State=1-A\HF
 =-1235.079073\RMSD=6.212e-09\RMSF=3.840e-05\Dipole=0.7091488,0.6606751
 ,0.1362724\Quadrupole=0.9474623,-0.1866253,-0.760837,1.0427624,-0.4550
 927,0.9913509\PG=C01 [X(C1H4S3)]\\@
```

```
1\1\GINC-ORC48\FOpt\RM062X\6-311++G(3df,3pd)\C1H3S3(1-)\SNIKOO\27-Jul-
 2017\0\\# opt freq m062x/6-311++g(3df,3pd)\\CH3-SSS-\\-1,1\S,-1.318473
 2833,0.0825051531,-0.0308002647\S,0.5524111347,-0.7945913357,-0.243437
 4439\C,1.6109213951,0.6483225599,0.0170450816\H,1.3057701234,1.1203815
 115,0.9511440433\H,2.6426856508,0.3077758578,0.0952947419\H,1.50961740
 75,1.3528116476,-0.8057993396\S,-1.5189639083,0.6655913259,1.915366771
 4\\Version=EM64L-G09RevD.01\State=1-A\HF=-1234.5449574\RMSD=7.686e-09\
 RMSF=4.667e-05\Dipole=1.5325513,-0.0754504,-1.1148464\Quadrupole=0.444
 8073,1.5098759,-1.9546832,2.5824633,4.101677,-2.3504677\PG=C01 [X(C1H3
 S3)]\\@
```

CH_3SSS^- M08HX

```
1\1\GINC-ORC25\FOpt\RM08HX\6-31G(d)\C1H3S3(1-)\SNIKOO\01-Aug-2017\0\\#
  opt freq 6-31g(d) m08hx\\CH3-SSS-\\-1,1\S,-1.3405670685,0.0550633594,
```

-0.021904644\S,0.5715221239,-0.8230045163,-0.3112823842\C,1.5922499944
,0.6410349861,0.014395839\H,1.219590302,1.1069755104,0.9408959745\H,2.
6355221197,0.3263723148,0.1549268957\H,1.5300121797,1.3640202202,-0.80
97147929\S,-1.4243611312,0.7123348453,1.9314967019\\Version=EM64L-G16R
evA.03\State=1-A\HF=-1234.4028543\RMSD=6.811e-09\RMSF=1.357e-05\Dipole
=1.4895922,-0.0671102,-1.1422341\Quadrupole=0.8108041,1.3873028,-2.198
1069,2.7647359,3.9519368,-2.5323057\PG=C01 [X(C1H3S3)]\\@

1\1\GINC-ORC25\FOpt\RM08HX\6-311G(d)\C1H3S3(1-)\SNIKOO\01-Aug-2017\0\\
opt freq 6-311g(d) m08hx\\CH3-SSS-\\-1,1\S,-1.3326114087,0.064491878
6,-0.0448539476\S,0.5817705821,-0.8339455775,-0.2864667275\C,1.5934086
771,0.6381622148,0.0218327046\H,1.2514412595,1.0944911842,0.9599862255
\H,2.6419255873,0.3347438516,0.1211331476\H,1.4883992148,1.3614258559,
-0.7939805242\S,-1.440365392,0.7234273124,1.9211627116\\Version=EM64L-
G16RevA.03\State=1-A\HF=-1234.5169122\RMSD=4.845e-09\RMSF=1.205e-05\Di
pole=1.6334419,-0.1405047,-1.2847192\Quadrupole=0.8522326,1.3670138,-2
.2192464,2.9550364,4.2993109,-2.684468\PG=C01 [X(C1H3S3)]\\@

1\1\GINC-ORC12\FOpt\RM08HX\6-311+G(d)\C1H3S3(1-)\SNIKOO\01-Aug-2017\0\
\# opt freq 6-311+g(d) m08hx\\CH3-SSS-\\-1,1\S,-1.3346791903,0.0676044
768,-0.0391398663\S,0.5772631968,-0.8283643687,-0.2885971584\C,1.59533
36119,0.639384594,0.0188777377\H,1.2502377911,1.1022864915,0.953290097
2\H,2.6413695204,0.3294178801,0.1280943248\H,1.504076208,1.360391368,-
0.8012820357\S,-1.4496326179,0.7120762784,1.9275704907\\Version=EM64L-
G16RevA.03\State=1-A\HF=-1234.5216179\RMSD=8.218e-09\RMSF=1.274e-05\Di
pole=1.5495995,-0.1248166,-1.234301\Quadrupole=0.9467838,1.4058683,-2.
3526522,2.8422388,4.2276662,-2.636226\PG=C01 [X(C1H3S3)]\\@

1\1\GINC-ORC6\FOpt\RM08HX\6-311+G(d,p)\C1H3S3(1-)\SNIKOO\01-Aug-2017\0
\\# opt freq 6-311+g(d,p) m08hx\\CH3-SSS-\\-1,1\S,-1.3353456672,0.0650
464547,-0.0379042024\S,0.577659692,-0.8269357128,-0.2936772885\C,1.594
7837544,0.6412144229,0.0190552139\H,1.2407115916,1.1014544643,0.951879
7746\H,2.6387443266,0.327961793,0.1342717921\H,1.5069589446,1.36028341
48,-0.8028556619\S,-1.4395441219,0.713771883,1.9280439622\\Version=EM6
4L-G16RevA.03\State=1-A\HF=-1234.5252189\RMSD=7.139e-09\RMSF=1.188e-05
\Dipole=1.5305707,-0.1313959,-1.2333206\Quadrupole=0.9388476,1.4086115
,-2.347459,2.7990406,4.2156098,-2.6314988\PG=C01 [X(C1H3S3)]\\@

1\1\GINC-ORC6\FOpt\RM08HX\6-311G(2d,p)\C1H3S3(1-)\SNIKOO\01-Aug-2017\0
\\# opt freq 6-311g(2d,p) m08hx\\CH3-SSS-\\-1,1\S,-1.3293991892,0.0687
409411,-0.0356899577\S,0.5676583652,-0.8183912173,-0.2936340662\C,1.59
07929387,0.6442941288,0.0177621923\H,1.2211429293,1.1065555781,0.94194
8869\H,2.6297755301,0.3255503699,0.1517288406\H,1.5180101722,1.3546500
021,-0.8107015331\S,-1.4140122263,0.7013969173,1.9273992452\\Version=E
M64L-G16RevA.03\State=1-A\HF=-1234.5376522\RMSD=4.139e-09\RMSF=1.249e-
05\Dipole=1.5315695,-0.1094961,-1.1939046\Quadrupole=0.7586283,1.34430
2,-2.1029303,2.6454609,4.1640541,-2.4321429\PG=C01 [X(C1H3S3)]\\@

1\1\GINC-ORC9\FOpt\RM08HX\6-311+G(df,p)\C1H3S3(1-)\SNIKOO\01-Aug-2017\
0\\# opt freq 6-311+g(df,p) m08hx\\CH3-SSS-\\-1,1\S,-1.3289427648,0.06
66903751,-0.0355811723\S,0.5688216943,-0.8200318391,-0.2858677541\C,1.
5953166307,0.6410472895,0.0186222658\H,1.2486315769,1.1067013408,0.951
5268575\H,2.6383261851,0.3237163005,0.129894384\H,1.5087557875,1.36025
80486,-0.8035709747\S,-1.4469405897,0.7044152045,1.9237899838\\Version
=EM64L-G16RevA.03\State=1-A\HF=-1234.535881\RMSD=4.879e-09\RMSF=2.852e
-05\Dipole=1.5351142,-0.1306209,-1.2401522\Quadrupole=0.8652256,1.5193
929,-2.3846185,2.8261083,4.2120625,-2.6997589\PG=C01 [X(C1H3S3)]\\@

1\1\GINC-ORC9\FOpt\RM08HX\6-311+G(2df,p)\C1H3S3(1-)\SNIKOO\01-Aug-2017
\0\\# opt freq 6-311+g(2df,p) m08hx\\CH3-SSS-\\-1,1\S,-1.3231493646,0.
072435723,-0.0310321305\S,0.5564119451,-0.8061098443,-0.2816848291\C,1
.592426574,0.6444331813,0.0171054924\H,1.2379349298,1.1159717774,0.942
8007873\H,2.6296128953,0.3162498855,0.143010132\H,1.5220036659,1.35450
02211,-0.8125980459\S,-1.4312721255,0.6853157761,1.9212121838\\Version
=EM64L-G16RevA.03\State=1-A\HF=-1234.5513355\RMSD=5.306e-09\RMSF=1.232
e-04\Dipole=1.4691996,-0.11323,-1.1601054\Quadrupole=0.7276289,1.46185
05,-2.1894794,2.5816472,4.0829166,-2.4511315\PG=C01 [X(C1H3S3)]\\@

1\1\GINC-ORC6\FOpt\RM08HX\6-311++G(3df,3pd)\C1H3S3(1-)\SNIKOO\01-Aug-2
017\0\\# opt freq 6-311++g(3df,3pd) m08hx\\CH3-SSS-\\-1,1\S,-1.3150566
271,0.0715507321,-0.0304293815\S,0.5574462041,-0.8059069152,-0.2799351
63\C,1.5905433261,0.6440327898,0.018934514\H,1.2371987088,1.1082726552
,0.9462067889\H,2.6264596062,0.3188989981,0.1387809994\H,1.5116116706,
1.3544086651,-0.8062295291\S,-1.4242343687,0.6915397949,1.9114853613\\
Version=EM64L-G16RevA.03\State=1-A\HF=-1234.5573554\RMSD=5.800e-09\RMS
F=1.287e-04\Dipole=1.4233732,-0.0986544,-1.0843087\Quadrupole=0.667094
7,1.3786331,-2.0457278,2.5058298,3.903765,-2.4088723\PG=C01 [X(C1H3S3)
]\\@

$CH_3SSS^{\cdot}$ B3LYP

1\1\GINC-ORC330\FOpt\UB3LYP\6-31G(d)\C1H3S3(2)\SNIKOO\20-Aug-2017\0\\#
opt freq ub3lyp/6-31g(d)\\CH3-SSS\\0,2\S,0.5909599412,-0.8545595326,0
.2526372707\S,-1.4357619347,-0.5653631685,-0.3791189427\C,-1.830817432
8,1.0719028779,0.3329430763\H,-1.087130536,1.8089521349,0.019147499\H,
-2.8045241195,1.349150143,-0.080719291\H,-1.8912389916,1.0248538625,1.
422547269\S,1.6953390734,0.7558056827,-0.0629468814\\Version=EM64L-G09
RevD.01\State=2-A\HF=-1234.4694176\S2=0.752836\S2-1=0.\S2A=0.750007\RM
SD=9.317e-09\RMSF=9.533e-06\Dipole=-0.682642,0.3546125,0.2398426\Quadr
upole=0.212752,0.460057,-0.672809,-2.4540642,-0.7428553,0.3348119\PG=C
01 [X(C1H3S3)]\\@

1\1\GINC-ORC330\FOpt\UB3LYP\6-311G(2d,p)\C1H3S3(2)\SNIKOO\20-Aug-2017\
0\\# opt freq ub3lyp/6-311g(2d,p)\\CH3-SSS\\0,2\S,0.5854910475,-0.8686
788506,0.2062125195\S,-1.4539192583,-0.5696856899,-0.3721340749\C,-1.8
159128075,1.0735384297,0.3273044068\H,-1.0845327234,1.7976092011,-0.02
73161504\H,-2.8021559086,1.3399795841,-0.0534138414\H,-1.8332094953,1.
0368491285,1.4148285373\S,1.6410651455,0.781130197,0.009008603\\Versio
n=EM64L-G09RevD.01\State=2-A\HF=-1234.5770032\S2=0.75427\S2-1=0.\S2A=0
.750014\RMSD=8.982e-09\RMSF=1.542e-05\Dipole=-0.7090344,0.3045921,0.19
76688\Quadrupole=0.1382103,0.7971134,-0.9353236,-2.270949,-0.6092668,0
.2662189\PG=C01 [X(C1H3S3)]\\@

1\1\GINC-ORC164\FOpt\UB3LYP\6-311+G(2df,p)\C1H3S3(2)\SNIKOO\20-Aug-201
7\0\\# opt freq ub3lyp/6-311+g(2df,p) geom=connectivity\\Title Card Re
quired\\0,2\S,0.5869598983,-0.8771475005,0.2875181247\S,-1.4152344473,
-0.4829593021,-0.2954105392\C,-1.6607400439,1.2239728063,0.2804084247\
H,-0.8876436577,1.8714995601,-0.1301317265\H,-2.6309258201,1.528424314
6,-0.1130508948\H,-1.6700189868,1.2719631061,1.3679248904\S,1.77557005
75,0.6092030155,-0.1678752792\\Version=EM64L-G09RevD.01\State=2-A\HF=-
1234.5903696\S2=0.754766\S2-1=0.\S2A=0.750018\RMSD=4.228e-09\RMSF=8.59
2e-06\Dipole=-0.6357105,0.3654437,0.2012871\Quadrupole=-0.095039,0.925
6035,-0.8305645,-2.2372063,-0.4467876,0.2533481\PG=C01 [X(C1H3S3)]\\@

1\1\GINC-ORC113\FOpt\UB3LYP\6-311++G(3df,3pd)\C1H3S3(2)\SNIKOO\20-Aug-
2017\0\\# opt freq ub3lyp/6-311++g(3df,3pd)\\CH3-SSS\\0,2\S,0.57833820
83,-0.8454064437,0.2251266462\S,-1.4324555463,-0.5596850927,-0.3652586
226\C,-1.8189745793,1.0694151291,0.3320195449\H,-1.0784475118,1.796355
9826,0.0070346331\H,-2.791100136,1.340092194,-0.0762174314\H,-1.869764
3013,1.0278834047,1.4169681608\S,1.6492298664,0.762086826,-0.035182930
9\\Version=EM64L-G09RevD.01\State=2-A\HF=-1234.5949302\S2=0.75511\S2-1
=0.\S2A=0.75002\RMSD=4.025e-09\RMSF=1.377e-05\Dipole=-0.6666476,0.3094
476,0.1956466\Quadrupole=0.3756752,0.4938118,-0.869487,-2.134241,-0.60
85098,0.2724201\PG=C01 [X(C1H3S3)]\\@

$CH_3SSS^{\cdot}$ B3PW91

1\1\GINC-ORC8\FOpt\UB3PW91\6-31G(d)\C1H3S3(2)\SNIKOO\20-Aug-2017\0\\#
opt freq ub3pw91/6-31g(d)\\CH3-SSS\\0,2\S,0.5757567239,-0.8457437399,0
.2459694881\S,-1.4306568177,-0.5599935672,-0.3761009463\C,-1.820817775
4,1.0656101379,0.3306170491\H,-1.0812410033,1.8061034875,0.0124925969\
H,-2.7969209379,1.3434252157,-0.0777726375\H,-1.8768836657,1.022403837
1,1.4210679527\S,1.667589476,0.7589366289,-0.0517835029\\Version=EM64L
-G09RevD.01\State=2-A\HF=-1234.302955\S2=0.752778\S2-1=0.\S2A=0.750006
\RMSD=3.718e-09\RMSF=3.476e-05\Dipole=-0.7062727,0.366981,0.2382685\Qu
adrupole=0.2192573,0.443337,-0.6625943,-2.4924007,-0.7575923,0.3326219
\PG=C01 [X(C1H3S3)]\\@

1\1\GINC-ORC330\FOpt\UB3PW91\6-311G(2d,p)\C1H3S3(2)\SNIKOO\20-Aug-2017
\0\\# opt freq ub3pw91/6-311g(2d,p)\\CH3-SSS\\0,2\S,0.5774184972,-0.85
83709786,0.1616530757\S,-1.4643920238,-0.5837045091,-0.3357846385\C,-1
.8008835005,1.0624896805,0.3292130599\H,-1.0435295438,1.763762888,-0.0
225579832\H,-2.7728415903,1.3497527478,-0.0743937075\H,-1.8378003186,1
.0445989108,1.417733791\S,1.5788544799,0.8122132606,0.0286264026\\Vers
ion=EM64L-G09RevD.01\State=2-A\HF=-1234.4087667\S2=0.754321\S2-1=0.\S2
A=0.750015\RMSD=5.248e-09\RMSF=1.616e-05\Dipole=-0.7223785,0.2977729,0
.1839309\Quadrupole=0.1321916,0.813502,-0.9456936,-2.2896469,-0.571498
2,0.2729483\PG=C01 [X(C1H3S3)]\\@

1\1\GINC-ORC5\FOpt\UB3PW91\6-311+G(2df,p)\C1H3S3(2)\SNIKOO\20-Aug-2017
\0\\# opt freq ub3pw91/6-311+g(2df,p)\\CH3-SSS\\0,2\S,0.5694208217,-0.
8427951069,0.2088103246\S,-1.4367517776,-0.5657251201,-0.3515323151\C,
-1.8070991973,1.062222246,0.3313044801\H,-1.0550693132,1.7816147141,0.
0052842051\H,-2.7757986453,1.3434548914,-0.0848021754\H,-1.8652627968,
1.0293122629,1.4189407238\S,1.6073869084,0.7826581126,-0.0235152431\\V
ersion=EM64L-G09RevD.01\State=2-A\HF=-1234.4225676\S2=0.754962\S2-1=0.
\S2A=0.75002\RMSD=5.142e-09\RMSF=2.169e-05\Dipole=-0.6844534,0.2969968
,0.1976226\Quadrupole=0.2693833,0.5452299,-0.8146132,-2.259902,-0.6028
406,0.2650296\PG=C01 [X(C1H3S3)]\\@

1\1\GINC-ORC330\FOpt\UB3PW91\6-311++G(3df,3pd)\C1H3S3(2)\SNIKOO\20-Aug
-2017\0\\# opt freq ub3pw91/6-311++g(3df,3pd)\\CH3-SSS\\0,2\S,0.569672
7617,-0.8361422929,0.1904700947\S,-1.4418313103,-0.5728965669,-0.33303
85934\C,-1.8051611391,1.0586355048,0.3343892158\H,-1.0377789956,1.7634
279256,0.0153052928\H,-2.7614788027,1.3511621851,-0.0980134939\H,-1.87
78888307,1.0329539815,1.4198137552\S,1.5912923167,0.7936012628,-0.0244
362711\\Version=EM64L-G09RevD.01\State=2-A\HF=-1234.4267857\S2=0.75539
5\S2-1=0.\S2A=0.750023\RMSD=3.537e-09\RMSF=1.128e-05\Dipole=-0.6816108
,0.309556,0.1875757\Quadrupole=0.3766728,0.4954684,-0.8721412,-2.13912
15,-0.5755112,0.2671052\PG=C01 [X(C1H3S3)]\\@

$CH_3SSS^{\cdot}$ wB97XD

1\1\GINC-ORC330\FOpt\UwB97XD\6-31G(d)\C1H3S3(2)\SNIKOO\20-Aug-2017\0\\
 # opt freq uwb97xd/6-31g(d)\\CH3-SSS\\0,2\S,0.5502516049,-0.7809926968
 ,0.3993585472\S,-1.348862007,-0.4940320529,-0.4443826349\C,-1.84481971
 6,1.0718840039,0.3351331031\H,-1.1357189836,1.8636675485,0.0821925999\
 H,-2.8213526048,1.3224771753,-0.0872787681\H,-1.9331520402,0.963781270
 1,1.417838501\S,1.7704797466,0.6439567519,-0.1983713482\\Version=EM64L
 -G09RevD.01\State=2-A\HF=-1234.390954\S2=0.753343\S2-1=0.\S2A=0.750009
 \RMSD=5.377e-09\RMSF=2.084e-05\Dipole=-0.68825,0.4085168,0.2923516\Qua
 drupole=0.4047177,0.3414678,-0.7461855,-2.4205697,-0.85423,0.4908584\P
 G=C01 [X(C1H3S3)]\\@

1\1\GINC-ORC23\FOpt\UwB97XD\6-311G(2d,p)\C1H3S3(2)\SNIKOO\20-Aug-2017\
 0\\# opt freq uwb97xd/6-311g(2d,p)\\CH3-SSS\\0,2\S,0.5437750626,-0.801
 8780264,0.3686681248\S,-1.368505331,-0.5026019284,-0.4389659821\C,-1.8
 299733146,1.0728863993,0.3316575669\H,-1.1221613909,1.8496579521,0.047
 3940437\H,-2.8130748015,1.3189168143,-0.0708776559\H,-1.8909911874,0.9
 72724359,1.4135240422\S,1.7177569628,0.6810364301,-0.1469101396\\Versi
 on=EM64L-G09RevD.01\State=2-A\HF=-1234.4967669\S2=0.755359\S2-1=0.\S2A
 =0.75002\RMSD=8.475e-09\RMSF=3.097e-05\Dipole=-0.6947586,0.34911,0.253
 3886\Quadrupole=0.2948588,0.566087,-0.8609458,-2.1972927,-0.7136085,0.
 3164386\PG=C01 [X(C1H3S3)]\\@

1\1\GINC-ORC20\FOpt\UwB97XD\6-311+G(2df,p)\C1H3S3(2)\SNIKOO\20-Aug-201
 7\0\\# opt freq uwb97xd/6-311+g(2df,p)\\CH3-SSS\\0,2\S,0.5432500084,-0
 .7821300527,0.3826020286\S,-1.3511765997,-0.4932134542,-0.4352656738\C
 ,-1.8342452274,1.070983823,0.334451025\H,-1.124382827,1.8538803206,0.0
 713240239\H,-2.8099185821,1.3177892045,-0.0854240651\H,-1.9170606025,0
 .9658933363,1.4147734526\S,1.7303598302,0.6575388225,-0.1779707913\\Ve
 rsion=EM64L-G09RevD.01\State=2-A\HF=-1234.5106995\S2=0.755729\S2-1=0.\
 S2A=0.750023\RMSD=6.492e-09\RMSF=3.319e-05\Dipole=-0.661332,0.3393739,
 0.2565527\Quadrupole=0.4352823,0.3974806,-0.8327629,-2.1953656,-0.7127
 262,0.3475953\PG=C01 [X(C1H3S3)]\\@

1\1\GINC-ORC330\FOpt\UwB97XD\6-311++G(3df,3pd)\C1H3S3(2)\SNIKOO\20-Aug
 -2017\0\\# opt freq uwb97xd/6-311++g(3df,3pd) geom=connectivity\\Title
  Card Required\\0,2\S,0.5610793459,-0.8137223191,0.3727385709\S,-1.342
 6713382,-0.4698380874,-0.3860027623\C,-1.7322068302,1.134305718,0.3428
 132302\H,-0.9866418196,1.8690213155,0.0472880745\H,-2.6977169598,1.420
 5400634,-0.0711963425\H,-1.8031881603,1.065093204,1.4252621172\S,1.774
 1357623,0.5859531056,-0.203302888\\Version=EM64L-G09RevD.01\State=2-A\
 HF=-1234.5151718\S2=0.756246\S2-1=0.\S2A=0.750027\RMSD=8.609e-09\RMSF=
 1.534e-05\Dipole=-0.6345889,0.3831705,0.2377299\Quadrupole=0.3331209,0
 .549385,-0.8825058,-2.0946927,-0.6727679,0.3554908\PG=C01 [X(C1H3S3)]\
 \@

$CH_3SSS^{\cdot}$ M062X

1\1\GINC-ORC19\FOpt\UM062X\6-31G(d)\C1H3S3(2)\SNIKOO\20-Aug-2017\0\\#
 opt freq m062x/6-31g(d)\\CH3-SSS\\0,2\S,0.5463170533,-0.8325989887,0.3
 896059652\S,-1.3774946737,-0.4535906646,-0.3602796021\C,-1.6517496832,
 1.2243495098,0.2775710532\H,-0.8836208998,1.9005258023,-0.1035367703\H
 ,-2.6254734202,1.5375711492,-0.1051762112\H,-1.666319498,1.2302599281,
 1.3685085166\S,1.7814781216,0.5608702641,-0.2272809513\\Version=EM64L-
 G09RevD.01\State=2-A\HF=-1234.3269663\S2=0.753606\S2-1=0.\S2A=0.75001\
 RMSD=6.266e-09\RMSF=2.729e-05\Dipole=-0.6292589,0.4332145,0.2533524\Qu

adrupole=-0.1496576,0.8583105,-0.7086529,-2.3881628,-0.5916882,0.41547
47\PG=C01 [X(C1H3S3)]\\@

1\1\GINC-ORC20\FOpt\UM062X\6-311G(2d,p)\C1H3S3(2)\SNIKOO\20-Aug-2017\0
\\# opt freq m062x/6-311g(2d,p)\\CH3-SSS\\0,2\S,0.534475664,-0.8595825
027,0.3544327847\S,-1.3983371602,-0.4595710717,-0.3559324786\C,-1.6343
588301,1.2256854932,0.2657592786\H,-0.8877525103,1.8876543317,-0.16645
29676\H,-2.6260440093,1.5256132775,-0.06999235\H,-1.5906944451,1.24018
67804,1.352197292\S,1.7258482909,0.6074006917,-0.1405995591\\Version=E
M64L-G09RevD.01\State=2-A\HF=-1234.4462832\S2=0.75519\S2-1=0.\S2A=0.75
0018\RMSD=5.708e-09\RMSF=2.180e-05\Dipole=-0.6577832,0.3791476,0.21037
82\Quadrupole=-0.24436,1.1343198,-0.8899598,-2.1604932,-0.4754443,0.20
28096\PG=C01 [X(C1H3S3)]\\@

1\1\GINC-ORC264\FOpt\UM062X\6-311+G(2df,p)\C1H3S3(2)\SNIKOO\20-Aug-201
7\0\\# opt freq m062x/6-311+g(2df,p)\\CH3-SSS\\0,2\S,0.536414751,-0.83
87391207,0.3686429071\S,-1.3807380322,-0.4528868421,-0.3503833553\C,-1
.6400633424,1.2235744955,0.2721627769\H,-0.8804641538,1.8904680028,-0.
1308749579\H,-2.619964391,1.527515523,-0.0939515249\H,-1.6317853596,1.
2354925919,1.360035435\S,1.739737528,0.5819623496,-0.1862192808\\Versi
on=EM64L-G09RevD.01\State=2-A\HF=-1234.4605687\S2=0.755209\S2-1=0.\S2A
=0.750018\RMSD=2.986e-09\RMSF=1.862e-05\Dipole=-0.6188375,0.3693803,0.
2175983\Quadrupole=-0.0603625,0.9057787,-0.8454162,-2.1805347,-0.48725
92,0.2579382\PG=C01 [X(C1H3S3)]\\@

1\1\GINC-ORC325\FOpt\UM062X\6-311++G(3df,3pd)\C1H3S3(2)\SNIKOO\20-Aug-
2017\0\\# opt freq m062x/6-311++g(3df,3pd)\\CH3-SSS\\0,2\S,0.534029172
9,-0.842044053,0.3495691163\S,-1.3871521851,-0.4555687528,-0.342762204
7\C,-1.6369898746,1.2226423819,0.2705828405\H,-0.8790956456,1.88232085
97,-0.1423595956\H,-2.6174079888,1.5261096179,-0.088714421\H,-1.617698
3462,1.2408647101,1.3563367221\S,1.7274518674,0.5930622363,-0.16324045
77\\Version=EM64L-G09RevD.01\State=2-A\HF=-1234.4656792\S2=0.755852\S2
-1=0.\S2A=0.750022\RMSD=7.009e-09\RMSF=2.099e-05\Dipole=-0.6142892,0.3
807341,0.1968169\Quadrupole=0.0713425,0.8511932,-0.9225357,-2.0638877,
-0.4840288,0.2448539\PG=C01 [X(C1H3S3)]\\@

$CH_3SSS^{\cdot}$ M08HX

1\1\GINC-ORC5\FOpt\UM08HX\6-31G(d)\C1H3S3(2)\SNIKOO\20-Aug-2017\0\\# o
pt freq 6-31g(d) m08hx\\CH3-SSS\\0,2\S,0.5414009157,-0.8318080636,0.39
51874745\S,-1.3808722392,-0.4609890364,-0.3615266491\C,-1.6453651769,1
.217901191,0.2748630911\H,-0.8708141756,1.894748213,-0.1062285997\H,-2
.6205819819,1.5437786839,-0.1063942575\H,-1.6600810807,1.2274833565,1.
3704755436\S,1.7594507386,0.5762726557,-0.2269646029\\Version=EM64L-G1
6RevA.03\State=2-A\HF=-1234.3237611\S2=0.753742\S2-1=0.\S2A=0.750011\R
MSD=9.516e-09\RMSF=9.485e-06\Dipole=-0.638445,0.4201397,0.2470531\Quad
rupole=-0.1597967,0.8662942,-0.7064975,-2.3579135,-0.5634479,0.4117245
\PG=C01 [X(C1H3S3)]\\@

1\1\GINC-ORC8\FOpt\UM08HX\6-311G(2d,p)\C1H3S3(2)\SNIKOO\20-Aug-2017\0\
\# opt freq m08hx/6-311g(2d,p)\\CH3-SSS\\0,2\S,0.5353730665,-0.8593674
994,0.3465738058\S,-1.410152399,-0.4732174764,-0.3396639078\C,-1.62744
55795,1.2173234716,0.2663889144\H,-0.8564869482,1.8643331129,-0.157284
7079\H,-2.6089648189,1.5400298753,-0.090392108\H,-1.6030770521,1.24209
07664,1.3574892501\S,1.6938907312,0.6361947495,-0.1436992466\\Version=
EM64L-G16RevA.03\State=2-A\HF=-1234.4538103\S2=0.755497\S2-1=0.\S2A=0.
75002\RMSD=4.554e-09\RMSF=9.798e-06\Dipole=-0.6461446,0.3628536,0.1997

823\Quadrupole=-0.2352736,1.117235,-0.8819614,-2.1243416,-0.434539,0.1
982206\PG=C01 [X(C1H3S3)]\\@

1\1\GINC-ORC19\FOpt\UM08HX\6-311+G(2df,p)\C1H3S3(2)\SNIKOO\20-Aug-2017
\0\\# opt freq 6-311+g(2df,p) m08hx\\CH3-SSS\\0,2\S,0.5355445312,-0.83
82211924,0.3618013505\S,-1.392192399,-0.4663748251,-0.3373733656\C,-1.
6321207433,1.2151760153,0.2731950994\H,-0.8496791929,1.8682956583,-0.1
21256882\H,-2.6025641581,1.5419664798,-0.1101638482\H,-1.6407494364,1.
2353516491,1.3653309902\S,1.7048983986,0.6111932149,-0.1921213443\\Ver
sion=EM64L-G16RevA.03\State=2-A\HF=-1234.4687004\S2=0.756103\S2-1=0.\S
2A=0.750026\RMSD=6.065e-09\RMSF=1.020e-05\Dipole=-0.608162,0.3504621,0
.2100103\Quadrupole=-0.076807,0.8901165,-0.8133095,-2.139588,-0.450746
8,0.25573\PG=C01 [X(C1H3S3)]\\@

1\1\GINC-ORC264\FOpt\UM08HX\6-311++G(3df,3pd)\C1H3S3(2)\SNIKOO\20-Aug-
2017\0\\# opt freq 6-311++g(3df,3pd) m08hx\\CH3-SSS\\0,2\S,0.534164538
,-0.8378618133,0.3439691679\S,-1.3992356943,-0.4703774869,-0.326206951
5\C,-1.6295453456,1.213729039,0.2738284382\H,-0.8413492385,1.854133861
8,-0.1240109895\H,-2.5945639692,1.5445587386,-0.1124989301\H,-1.636700
0905,1.2391342177,1.3634087254\S,1.6903668001,0.6240704433,-0.17907746
03\\Version=EM64L-G16RevA.03\State=2-A\HF=-1234.4752635\S2=0.756763\S2
-1=0.\S2A=0.750032\RMSD=5.446e-09\RMSF=3.416e-05\Dipole=-0.6044088,0.3
6819,0.1948972\Quadrupole=0.0617701,0.8334434,-0.8952135,-2.0469992,-0
.4557741,0.2377536\PG=C01 [X(C1H3S3)]\\@

$CH_3SSS^{\cdot}$ QCISD

1\1\GINC-ORC280\FOpt\UQCISD-FC\6-311+G(2df,p)\C1H3S3(2)\SNIKOO\26-Aug-
2017\0\\# opt 6-311+g(2df,p) qcisd\\CH3-SSSH\\0,2\S,0.4548848275,-0.83
87869825,0.3915971061\S,-1.3912770472,-0.4754926618,-0.5160743141\C,-1
.9016789882,1.0149053078,0.3855868458\H,-1.1903968223,1.8227357963,0.2
096833599\H,-2.8752340369,1.2983327418,-0.0209227525\H,-1.9976359642,0
.8107637073,1.4524304409\S,1.7025720314,0.6011370912,-0.1084756862\\Ve
rsion=EM64L-G09RevD.01\State=2-A\HF=-1232.2497138\MP2=-1232.9066469\MP
3=-1232.9585959\MP4D=-1232.977425\MP4DQ=-1232.9559479\PUHF=-1232.25424
91\PMP2-0=-1232.9094595\PMP3-0=-1232.9600838\MP4SDQ=-1232.9624458\QCIS
D=-1232.9642548\S2=0.771323\S2-1=0.75498\S2A=0.750318\RMSD=7.489e-09\R
MSF=4.115e-05\Dipole=-0.61131,0.335279,0.2591658\PG=C01 [X(C1H3S3)]\\@

CH_2CHSH B3LYP

1\1\GINC-ORC287\FOpt\RB3LYP\6-31G(d)\C2H4S1\SNIKOO\12-Jun-2017\0\\# op
t freq b3lyp/6-31g(d)\\CH2=CH-SH\\0,1\S,-1.1619243493,-0.0536587352,0.
\C,0.5317372706,0.4730460121,0.\H,0.599370209,1.5585583088,0.\C,1.6301
496041,-0.283898911,0.\H,2.6127854201,0.1783082588,0.\H,1.5961239575,-
1.3702732784,0.\H,-0.926187932,-1.3837815152,0.\\Version=EM64L-G09RevD
.01\State=1-A'\HF=-476.7767511\RMSD=3.162e-09\RMSF=3.256e-05\Dipole=0.
3575827,-0.195968,0.\Quadrupole=-0.1005171,2.639495,-2.5389779,0.97405
59,0.,0.\PG=CS [SG(C2H4S1)]\\@

1\1\GINC-ORC206\FOpt\RB3LYP\6-311G(d)\C2H4S1\SNIKOO\12-Jun-2017\0\\# o
pt freq b3lyp/6-311g(d)\\CH2=CH-SH\\0,1\S,-1.1638973226,-0.0501488633,
0.\C,0.5308258197,0.4693708577,0.\H,0.5982582514,1.5531450099,0.\C,1.6
266309591,-0.2842181907,0.\H,2.6073013743,0.1783159637,0.\H,1.59563879
29,-1.3691354584,0.\H,-0.9317177948,-1.3826931689,0.\\Version=EM64L-G0
9RevD.01\State=1-A'\HF=-476.8210722\RMSD=1.803e-09\RMSF=3.027e-05\Dipo
le=0.3410194,-0.2076701,0.\Quadrupole=0.0198943,2.8056694,-2.8255637,1

.0108176,0.,0.\PG=CS [SG(C2H4S1)]\\@

1\1\GINC-ORC206\FOpt\RB3LYP\6-311+G(d)\C2H4S1\SNIKOO\12-Jun-2017\0\\#
opt freq b3lyp/6-311+g(d)\\CH2=CH-SH\\0,1\S,-1.1633350152,-0.052250509
4,0.\C,0.5273128237,0.4699301924,0.\H,0.5946029424,1.5539104776,0.\C,1
.6262098559,-0.2826063266,0.\H,2.6059567908,0.1820260987,0.\H,1.597419
3837,-1.3676531061,0.\H,-0.9292937213,-1.3845703565,0.\\Version=EM64L-
G09RevD.01\State=1-A'\HF=-476.8235942\RMSD=2.357e-09\RMSF=3.631e-05\Di
pole=0.3176119,-0.2051235,0.\Quadrupole=-0.040331,2.8754765,-2.8351455
,0.9779199,0.,0.\PG=CS [SG(C2H4S1)]\\@

1\1\GINC-ORC225\FOpt\RB3LYP\6-311+G(d,p)\C2H4S1\SNIKOO\18-Jul-2017\0\\
opt freq rb3lyp/6-311+g(d,p)\\CH2=CH-SH\\0,1\S,-0.6886160545,-0.8651
421706,0.\C,0.0005128238,0.7648573576,0.\H,-0.7846239952,1.514201077,0
.\C,1.284125717,1.119425018,0.\H,1.5590913764,2.167387284,0.\H,2.09283
59191,0.3969118245,0.\H,0.4778872133,-1.5434693906,0.\\Version=EM64L-G
09RevD.01\State=1-A'\HF=-476.8334581\RMSD=2.613e-09\RMSF=1.111e-04\Dip
ole=0.350499,0.1070438,0.\Quadrupole=0.6809223,2.0579127,-2.738835,-1.
5706306,0.,0.\PG=CS [SG(C2H4S1)]\\@

1\1\GINC-ORC280\FOpt\RB3LYP\6-311G(2d,p)\C2H4S1\SNIKOO\27-Jul-2017\0\\
opt freq rb3lyp/6-311g(2d,p)\\CH2=CH-SH\\0,1\S,-0.6852169193,-0.8649
688532,0.\C,0.0035998358,0.7656936268,0.\H,-0.7816417459,1.5138550847,
0.\C,1.2817409915,1.1186714668,0.\H,1.5568076667,2.1658335294,0.\H,2.0
881808452,0.3951429897,0.\H,0.477742326,-1.5400568441,0.\\Version=EM64
L-G09RevD.01\State=1-A'\HF=-476.8361496\RMSD=3.067e-09\RMSF=6.762e-05\
Dipole=0.3262436,0.1303237,0.\Quadrupole=0.6892979,2.0300319,-2.719329
9,-1.4248939,0.,0.\PG=CS [SG(C2H4S1)]\\@

1\1\GINC-ORC225\FOpt\RB3LYP\6-311+G(df,p)\C2H4S1\SNIKOO\18-Jul-2017\0\
\# opt freq rb3lyp/6-311+g(df,p)\\CH2=CH-SH\\0,1\S,-0.68688178,-0.8631
528745,0.\C,0.0015801335,0.7648366338,0.\H,-0.783220148,1.5142041079,0
.\C,1.2825001153,1.1188514469,0.\H,1.5578603085,2.1664908131,0.\H,2.09
12044766,0.3966762968,0.\H,0.4781698941,-1.5437354241,0.\\Version=EM64
L-G09RevD.01\State=1-A'\HF=-476.8379643\RMSD=7.738e-09\RMSF=1.092e-04\
Dipole=0.3533618,0.1108395,0.\Quadrupole=0.6291589,2.0104973,-2.639656
2,-1.5702697,0.,0.\PG=CS [SG(C2H4S1)]\\@

1\1\GINC-ORC98\FOpt\RB3LYP\6-311+G(2df,p)\C2H4S1\SNIKOO\18-Jul-2017\0\
\# opt freq rb3lyp/6-311+g(2df,p) geom=connectivity\\CH2=CH-SH\\0,1\S,
-0.6841276494,-0.8600442325,0.\C,0.0021575859,0.7626282828,0.\H,-0.781
8168162,1.5124963173,0.\C,1.281507643,1.1177595672,0.\H,1.5563461765,2
.1648759009,0.\H,2.0889200554,0.3953385463,0.\H,0.4782260049,-1.538883
3821,0.\\Version=EM64L-G09RevD.01\State=1-A'\HF=-476.8428413\RMSD=6.00
3e-09\RMSF=1.242e-04\Dipole=0.3104226,0.1065108,0.\Quadrupole=0.714243
6,1.9656983,-2.6799418,-1.5077564,0.,0.\PG=CS [SG(C2H4S1)]\\@

1\1\GINC-ORC280\FOpt\RB3LYP\6-311G(2d,p)\C2H4S1\SNIKOO\27-Jul-2017\0\\
opt freq rb3lyp/6-311g(2d,p)\\CH2=CH-SH\\0,1\S,-0.6852169193,-0.8649
688532,0.\C,0.0035998358,0.7656936268,0.\H,-0.7816417459,1.5138550847,
0.\C,1.2817409915,1.1186714668,0.\H,1.5568076667,2.1658335294,0.\H,2.0
881808452,0.3951429897,0.\H,0.477742326,-1.5400568441,0.\\Version=EM64
L-G09RevD.01\State=1-A'\HF=-476.8361496\RMSD=3.067e-09\RMSF=6.762e-05\
Dipole=0.3262436,0.1303237,0.\Quadrupole=0.6892979,2.0300319,-2.719329
9,-1.4248939,0.,0.\PG=CS [SG(C2H4S1)]\\@

CH_2CHSH B3PW91

```
1\1\GINC-ORC152\FOpt\RB3PW91\6-31G(d)\C2H4S1\SNIKOO\20-Jul-2017\0\\# o
 pt freq rb3pw91/6-31g(d)\\CH2=CH-SH\\0,1\S,-0.6889438229,-0.8592234522
 ,0.\C,-0.0006480293,0.764385406,0.\H,-0.7844501969,1.5189795623,0.\C,1
 .2863377672,1.1138078843,0.\H,1.5668424069,2.1630353295,0.\H,2.0938085
 232,0.3858705757,0.\H,0.4765823519,-1.5359603056,0.\\Version=EM64L-G09
 RevD.01\State=1-A'\HF=-476.695743\RMSD=2.267e-09\RMSF=1.109e-04\Dipole
 =0.3921763,0.1434543,0.\Quadrupole=0.57761,2.076097,-2.653707,-1.53592
 37,0.,0.\PG=CS [SG(C2H4S1)]\\@

1\1\GINC-ORC128\FOpt\RB3PW91\6-311G(d)\C2H4S1\SNIKOO\20-Jul-2017\0\\#
 opt freq rb3pw91/6-311g(d) geom=connectivity\\CH2=CH-SH\\0,1\S,-0.6915
 053482,-0.8561372258,0.\C,0.0025643441,0.7627807152,0.\H,-0.7802651823
 ,1.5165592396,0.\C,1.2854361338,1.1136973319,0.\H,1.5634244025,2.16230
 23228,0.\H,2.0944158184,0.3891340188,0.\H,0.4754588316,-1.5374414025,0
 .\\Version=EM64L-G09RevD.01\State=1-A'\HF=-476.7366384\RMSD=1.424e-09\
 RMSF=2.002e-05\Dipole=0.3842474,0.1129296,0.\Quadrupole=0.6589245,2.22
 11073,-2.8800318,-1.5671328,0.,0.\PG=CS [SG(C2H4S1)]\\@

1\1\GINC-ORC122\FOpt\RB3PW91\6-311+G(d)\C2H4S1\SNIKOO\20-Jul-2017\0\\#
  opt freq rb3pw91/6-311+g(d) geom=connectivity\\CH2=CH-SH\\0,1\S,-0.69
 08473876,-0.8550540643,0.\C,0.0011389713,0.7620093852,0.\H,-0.78097532
 42,1.5168732409,0.\C,1.28550789,1.1131349414,0.\H,1.5637496378,2.16181
 99129,0.\H,2.0945093734,0.3884097428,0.\H,0.4764458394,-1.5362981588,0
 .\\Version=EM64L-G09RevD.01\State=1-A'\HF=-476.739001\RMSD=2.021e-09\R
 MSF=2.434e-05\Dipole=0.3704337,0.1023508,0.\Quadrupole=0.6909535,2.183
 0823,-2.8740358,-1.6134125,0.,0.\PG=CS [SG(C2H4S1)]\\@

1\1\GINC-ORC116\FOpt\RB3PW91\6-311+G(d,p)\C2H4S1\SNIKOO\20-Jul-2017\0\
 \# opt freq rb3pw91/6-311+g(d,p) geom=connectivity\\CH2=CH-SH\\0,1\S,-
 0.6887516659,-0.855421619,0.\C,0.0017730562,0.7629368813,0.\H,-0.78060
 4105,1.5166585291,0.\C,1.2862787478,1.1132196396,0.\H,1.5625036448,2.1
 61582548,0.\H,2.0921295465,0.386426011,0.\H,0.4761997757,-1.53450699,0
 .\\Version=EM64L-G09RevD.01\State=1-A'\HF=-476.7488004\RMSD=1.628e-09\
 RMSF=2.572e-05\Dipole=0.3584549,0.1003379,0.\Quadrupole=0.6715934,2.10
 11553,-2.7727487,-1.5803017,0.,0.\PG=CS [SG(C2H4S1)]\\@

1\1\GINC-ORC306\FOpt\RB3PW91\6-311G(2d,p)\C2H4S1\SNIKOO\27-Jul-2017\0\
 \# opt freq rb3pw91/6-311g(2d,p)\\CH2=CH-SH\\0,1\S,-0.6855356129,-0.85
 52607497,0.\C,0.0043706848,0.7636866059,0.\H,-0.7781938596,1.516258141
 4,0.\C,1.2837566894,1.1124767154,0.\H,1.5610803805,2.1599511107,0.\H,2
 .0877194489,0.3848790288,0.\H,0.4763312688,-1.5310958524,0.\\Version=E
 M64L-G09RevD.01\State=1-A'\HF=-476.7520132\RMSD=7.417e-09\RMSF=2.690e-
 05\Dipole=0.3283113,0.118316,0.\Quadrupole=0.7014982,2.0721395,-2.7736
 377,-1.4423934,0.,0.\PG=CS [SG(C2H4S1)]\\@
1\1\GINC-ORC107\FOpt\RB3PW91\6-311+G(df,p)\C2H4S1\SNIKOO\20-Jul-2017\0
 \\# opt freq rb3pw91/6-311+g(df,p) geom=connectivity\\CH2=CH-SH\\0,1\S
 ,-0.6876292289,-0.8533355909,0.\C,0.0029355083,0.7623337716,0.\H,-0.77
 93417234,1.5160041536,0.\C,1.284886715,1.1130218505,0.\H,1.5616592125,
 2.161093224,0.\H,2.0916821915,0.3874364275,0.\H,0.4753363251,-1.535658
 8364,0.\\Version=EM64L-G09RevD.01\State=1-A'\HF=-476.7530693\RMSD=2.02
 4e-09\RMSF=2.432e-05\Dipole=0.3609783,0.1031981,0.\Quadrupole=0.618999
 4,2.0508247,-2.6698241,-1.5765374,0.,0.\PG=CS [SG(C2H4S1)]\\@

1\1\GINC-ORC105\FOpt\RB3PW91\6-311+G(2df,p)\C2H4S1\SNIKOO\20-Jul-2017\
 0\\# opt freq rb3pw91/6-311+g(2df,p) geom=connectivity\\CH2=CH-SH\\0,1
```

\S,-0.6848619348,-0.8503787296,0.\C,0.0033082696,0.7600856277,0.\H,-0.
7776595685,1.5149490508,0.\C,1.283528795,1.1117680583,0.\H,1.560070600
5,2.159380929,0.\H,2.0890472879,0.3857243562,0.\H,0.4760955504,-1.5306
342924,0.\\Version=EM64L-G09RevD.01\State=1-A'\HF=-476.7582996\RMSD=2.
659e-09\RMSF=3.338e-05\Dipole=0.3149256,0.0996604,0.\Quadrupole=0.7086
098,2.004548,-2.7131578,-1.5133243,0.,0.\PG=CS [SG(C2H4S1)]\\@

1\1\GINC-ORC306\FOpt\RB3PW91\6-311++G(3df,3pd)\C2H4S1\SNIKOO\27-Jul-20
17\0\\# opt freq rb3pw91/6-311++g(3df,3pd)\\CH2=CH-SH\\0,1\S,-0.682733
5571,-0.8486230633,0.\C,0.0033901906,0.7592087286,0.\H,-0.7758008221,1
.514012245,0.\C,1.2834992317,1.111802572,0.\H,1.5587493884,2.158280209
3,0.\H,2.0875255474,0.3863618811,0.\H,0.474899021,-1.5301475728,0.\\Ve
rsion=EM64L-G09RevD.01\State=1-A'\HF=-476.7623336\RMSD=9.618e-09\RMSF=
3.526e-05\Dipole=0.2914972,0.1083892,0.\Quadrupole=0.7179459,1.8983534
,-2.6162993,-1.4394793,0.,0.\PG=CS [SG(C2H4S1)]\\@

CH_2CHSH wB97XD

1\1\GINC-ORC265\FOpt\RwB97XD\6-31G(d)\C2H4S1\SNIKOO\22-Jul-2017\0\\# o
pt freq rwb97xd/6-31g(d)\\CH2=CH-SH\\0,1\S,-0.687616317,-0.8611022073,
0.\C,-0.0014329521,0.7652357039,0.\H,-0.7798488525,1.5235616505,0.\C,1
.284398492,1.1066078386,0.\H,1.5707362809,2.1531666965,0.\H,2.08508632
63,0.3720747278,0.\H,0.4779680224,-1.5307884101,0.\\Version=EM64L-G09R
evD.01\State=1-A'\HF=-476.7271357\RMSD=5.964e-09\RMSF=6.498e-05\Dipole
=0.4130386,0.1722057,0.\Quadrupole=0.5737231,2.0877594,-2.6614825,-1.5
495819,0.,0.\PG=CS [SG(C2H4S1)]\\@

1\1\GINC-ORC265\FOpt\RwB97XD\6-311G(d)\C2H4S1\SNIKOO\22-Jul-2017\0\\#
opt freq rwb97xd/6-311g(d)\\CH2=CH-SH\\0,1\S,-0.6895776702,-0.85870818
22,0.\C,0.0013942669,0.7642673065,0.\H,-0.7764748868,1.521635729,0.\C,
1.2834991292,1.1064360301,0.\H,1.5679399061,2.1524637769,0.\H,2.085354
0284,0.3746222002,0.\H,0.4771562263,-1.5319608604,0.\\Version=EM64L-G0
9RevD.01\State=1-A'\HF=-476.7681102\RMSD=4.770e-09\RMSF=5.221e-05\Dipo
le=0.405019,0.1398348,0.\Quadrupole=0.6508761,2.2350242,-2.8859003,-1.
5884546,0.,0.\PG=CS [SG(C2H4S1)]\\@

1\1\GINC-ORC264\FOpt\RwB97XD\6-311+G(d)\C2H4S1\SNIKOO\22-Jul-2017\0\\#
opt freq rwb97xd/6-311+g(d)\\CH2=CH-SH\\0,1\S,-0.6890055015,-0.857773
23,0.\C,0.000136801,0.7637031583,0.\H,-0.7773322992,1.5217891674,0.\C,
1.283697297,1.1060107106,0.\H,1.5683533098,2.152149886,0.\H,2.08562956
07,0.3741059922,0.\H,0.4778118322,-1.5312296846,0.\\Version=EM64L-G09R
evD.01\State=1-A'\HF=-476.7704286\RMSD=4.134e-09\RMSF=1.838e-05\Dipole
=0.3922042,0.1303878,0.\Quadrupole=0.6912455,2.1971579,-2.8884034,-1.6
291788,0.,0.\PG=CS [SG(C2H4S1)]\\@

1\1\GINC-ORC263\FOpt\RwB97XD\6-311+G(d,p)\C2H4S1\SNIKOO\22-Jul-2017\0\
\# opt freq rwb97xd/6-311+g(d,p)\\CH2=CH-SH\\0,1\S,-0.6870514345,-0.85
80051969,0.\C,0.0005033783,0.7645019325,0.\H,-0.7769307519,1.521832292
2,0.\C,1.2840844046,1.1058595884,0.\H,1.5673376992,2.1515250581,0.\H,2
.0828361278,0.371814386,0.\H,0.4785115765,-1.5287720602,0.\\Version=EM
64L-G09RevD.01\State=1-A'\HF=-476.7800056\RMSD=6.303e-09\RMSF=4.136e-0
5\Dipole=0.3784501,0.1293125,0.\Quadrupole=0.6759734,2.1118763,-2.7878
497,-1.5920234,0.,0.\PG=CS [SG(C2H4S1)]\\@

1\1\GINC-ORC261\FOpt\RwB97XD\6-311G(2d,p)\C2H4S1\SNIKOO\27-Jul-2017\0\
\# opt freq rwb97xd/6-311g(2d,p)\\CH2=CH-SH\\0,1\S,-0.6841784525,-0.85
79122676,0.\C,0.0034407721,0.7650589525,0.\H,-0.7737711254,1.521470492

8,0.\C,1.2818424642,1.1052793966,0.\H,1.5650937155,2.1502832068,0.\H,2 .0790169882,0.3709377566,0.\H,0.4778466379,-1.5263615377,0.\\Version=E M64L-G09RevD.01\State=1-A'\HF=-476.783261\RMSD=3.523e-09\RMSF=6.862e-0 5\Dipole=0.3465464,0.1409378,0.\Quadrupole=0.7007991,2.087219,-2.78801 8,-1.4541398,0.,0.\PG=CS [SG(C2H4S1)]\\@

1\1\GINC-ORC262\FOpt\RwB97XD\6-311+G(df,p)\C2H4S1\SNIKOO\22-Jul-2017\0 \\# opt freq rwb97xd/6-311+g(df,p)\\CH2=CH-SH\\0,1\S,-0.6858095726,-0. 8560370112,0.\C,0.001535298,0.764106154,0.\H,-0.7757584525,1.521357686 2,0.\C,1.2827267726,1.1055176773,0.\H,1.5664317084,2.1509300234,0.\H,2 .0821358507,0.3724068824,0.\H,0.4780293955,-1.5295254122,0.\\Version=E M64L-G09RevD.01\State=1-A'\HF=-476.7842483\RMSD=4.850e-09\RMSF=1.089e- 05\Dipole=0.3812013,0.1327263,0.\Quadrupole=0.6231575,2.0594932,-2.682 6507,-1.5899157,0.,0.\PG=CS [SG(C2H4S1)]\\@

1\1\GINC-ORC261\FOpt\RwB97XD\6-311+G(2df,p)\C2H4S1\SNIKOO\22-Jul-2017\ 0\\# opt freq rwb97xd/6-311+g(2df,p)\\CH2=CH-SH\\0,1\S,-0.6833291062,- 0.8535754084,0.\C,0.0023303318,0.7624271189,0.\H,-0.7739655991,1.52026 00061,0.\C,1.2816770008,1.1045424075,0.\H,1.5646033445,2.149641554,0.\ H,2.0800274246,0.3713854509,0.\H,0.4779476036,-1.525925129,0.\\Version =EM64L-G09RevD.01\State=1-A'\HF=-476.7894931\RMSD=5.573e-09\RMSF=8.959 e-05\Dipole=0.335551,0.1250346,0.\Quadrupole=0.7170854,2.0188978,-2.73 59832,-1.5230081,0.,0.\PG=CS [SG(C2H4S1)]\\@

1\1\GINC-ORC177\FOpt\RwB97XD\6-311++G(3df,3pd)\C2H4S1\SNIKOO\27-Jul-20 17\0\\# opt freq rwb97xd/6-311++g(3df,3pd)\\CH2=CH-SH\\0,1\S,-0.681141 219,-0.8517738985,0.\C,0.0024473952,0.7616963571,0.\H,-0.7719324563,1. 5192295348,0.\C,1.2816175319,1.1045351738,0.\H,1.5632165933,2.14821421 51,0.\H,2.0780718012,0.3719890238,0.\H,0.4770113536,-1.525134406,0.\\V ersion=EM64L-G09RevD.01\State=1-A'\HF=-476.7940079\RMSD=2.907e-09\RMSF =1.483e-04\Dipole=0.312232,0.1306832,0.\Quadrupole=0.7428053,1.921769, -2.6645743,-1.4439256,0.,0.\PG=CS [SG(C2H4S1)]\\@

CH_2CHSH M062X

1\1\GINC-ORC200\FOpt\RM062X\6-31G(d)\C2H4S1\SNIKOO\13-Jun-2017\0\\# op t freq 6-31g(d) m062x\\CH2=CH-SH\\0,1\S,-0.682406497,-0.8617353012,0.\ C,-0.0044967443,0.7672957272,0.\H,-0.7848230802,1.5236660353,0.\C,1.28 06951126,1.1085914612,0.\H,1.5690233469,2.154042428,0.\H,2.0786672058, 0.3715112285,0.\H,0.4928696562,-1.512476579,0.\\Version=EM64L-G09RevD. 01\State=1-A'\HF=-476.6916393\RMSD=2.821e-09\RMSF=2.917e-05\Dipole=0.3 969349,0.1705871,0.\Quadrupole=0.6183627,2.0251514,-2.6435142,-1.50757 45,0.,0.\PG=CS [SG(C2H4S1)]\\@

1\1\GINC-ORC214\FOpt\RM062X\6-311G(d)\C2H4S1\SNIKOO\13-Jun-2017\0\\# o pt freq 6-311g(d) m062x\\CH2=CH-SH\\0,1\S,-0.6791983871,-0.8644097106, 0.\C,-0.0055472671,0.7656913476,0.\H,-0.7889708763,1.5163634045,0.\C,1 .2748768232,1.1137207929,0.\H,1.5562343061,2.1597508742,0.\H,2.0772829 865,0.3837512456,0.\H,0.5000894147,-1.5108049541,0.\\Version=EM64L-G09 RevD.01\State=1-A'\HF=-476.7398489\RMSD=3.365e-09\RMSF=2.861e-05\Dipol e=0.4004896,0.1459816,0.\Quadrupole=0.740034,2.1820673,-2.9221013,-1.5 689292,0.,0.\PG=CS [SG(C2H4S1)]\\@

1\1\GINC-ORC299\FOpt\RM062X\6-311+G(d)\C2H4S1\SNIKOO\13-Jun-2017\0\\# opt freq 6-311+g(d) m062x\\CH2=CH-SH\\0,1\S,-0.6802243767,-0.862326739 7,0.\C,-0.0054121365,0.764539484,0.\H,-0.7867112834,1.5177246176,0.\C,

```
1.2771668893,1.1105604698,0.\H,1.5605209622,2.1561614526,0.\H,2.078214
1934,0.378963994,0.\H,0.4976587516,-1.5114522782,0.\\Version=EM64L-G09
RevD.01\State=1-A'\HF=-476.742294\RMSD=5.272e-09\RMSF=2.928e-05\Dipole
=0.3870781,0.134777,0.\Quadrupole=0.7665248,2.1404093,-2.9069341,-1.61
00767,0.,0.\PG=CS [SG(C2H4S1)]\\@
```

```
1\1\GINC-ORC20\FOpt\RM062X\6-311+G(d,p)\C2H4S1\SNIKOO\19-Jul-2017\0\\#
 opt freq 6-311+g(d,p) m062x\\CH2=CH-SH\\0,1\S,-0.690874393,-0.8548165
107,0.\C,0.0004823045,0.765404177,0.\H,-0.7722932782,1.5267093837,0.\C
,1.2865847567,1.0966574605,0.\H,1.5801143408,2.1387683756,0.\H,2.07733
65628,0.3550139065,0.\H,0.4776867065,-1.5158567926,0.\\Version=EM64L-G
09RevD.01\State=1-A'\HF=-476.750545\RMSD=3.270e-09\RMSF=1.150e-04\Dipo
le=0.3790483,0.1310206,0.\Quadrupole=0.7177178,2.1098038,-2.8275216,-1
.5693145,0.,0.\PG=CS [SG(C2H4S1)]\\@
```

```
1\1\GINC-ORC296\FOpt\RM062X\6-311G(2d,p)\C2H4S1\SNIKOO\27-Jul-2017\0\\
# opt freq m062x/6-311g(2d,p)\\CH2=CH-SH\\0,1\S,-0.6874075172,-0.85487
44112,0.\C,0.0030406988,0.7665872555,0.\H,-0.7695905749,1.5266852062,0
.\C,1.2842404573,1.0958967698,0.\H,1.5782069927,2.1369787315,0.\H,2.07
20385389,0.3527593923,0.\H,0.4785084044,-1.5121529441,0.\\Version=EM64
L-G09RevD.01\State=1-A'\HF=-476.7540173\RMSD=7.480e-09\RMSF=7.413e-05\
Dipole=0.3453639,0.1438138,0.\Quadrupole=0.7557784,2.0871224,-2.842900
8,-1.4194168,0.,0.\PG=CS [SG(C2H4S1)]\\@
```

```
1\1\GINC-ORC20\FOpt\RM062X\6-311+G(df,p)\C2H4S1\SNIKOO\19-Jul-2017\0\\
# opt freq m062x/6-311+g(df,p) geom=connectivity\\CH2=CH-SH\\0,1\S,-0.
6897589803,-0.8530029204,0.\C,0.001701809,0.7650614097,0.\H,-0.7709072
103,1.5266189059,0.\C,1.285185344,1.0963735221,0.\H,1.5788580525,2.138
5469919,0.\H,2.0767308432,0.3554351202,0.\H,0.4772271419,-1.5171530294
,0.\\Version=EM64L-G09RevD.01\State=1-A'\HF=-476.754952\RMSD=9.899e-09
\RMSF=1.354e-04\Dipole=0.3822292,0.1356161,0.\Quadrupole=0.6585584,2.0
48395,-2.7069534,-1.5643408,0.,0.\PG=CS [SG(C2H4S1)]\\@
```

```
1\1\GINC-ORC13\FOpt\RM062X\6-311+G(2df,p)\C2H4S1\SNIKOO\19-Jul-2017\0\
\# opt freq m062x/6-311+g(2df,p) geom=connectivity\\CH2=CH-SH\\0,1\S,-
0.6870842429,-0.8504000284,0.\C,0.0020902534,0.7635371556,0.\H,-0.7696
604264,1.5253818439,0.\C,1.2841780007,1.0952889343,0.\H,1.5775425228,2
.1367592693,0.\H,2.0738133431,0.3536650192,0.\H,0.4781575493,-1.512352
1939,0.\\Version=EM64L-G09RevD.01\State=1-A'\HF=-476.7604124\RMSD=3.70
0e-09\RMSF=1.091e-04\Dipole=0.331991,0.1269674,0.\Quadrupole=0.7629881
,2.0035887,-2.7665768,-1.4839085,0.,0.\PG=CS [SG(C2H4S1)]\\@
```

```
1\1\GINC-ORC296\FOpt\RM062X\6-311++G(3df,3pd)\C2H4S1\SNIKOO\27-Jul-201
7\0\\# opt freq m062x/6-311++g(3df,3pd)\\CH2=CH-SH\\0,1\S,-0.685185273
,-0.8489896185,0.\C,0.0022015999,0.7630820339,0.\H,-0.7677349701,1.524
035392,0.\C,1.2842630805,1.095438318,0.\H,1.5765096447,2.1353143574,0.
\H,2.0718113078,0.3544702732,0.\H,0.4771716101,-1.5114707559,0.\\Versi
on=EM64L-G09RevD.01\State=1-A'\HF=-476.7648504\RMSD=9.784e-09\RMSF=1.5
00e-04\Dipole=0.305843,0.1311864,0.\Quadrupole=0.7821537,1.8971827,-2.
6793364,-1.3987504,0.,0.\PG=CS [SG(C2H4S1)]\\@
```

CH_2CHSH M08HX

```
1\1\GINC-ORC166\FOpt\RM08HX\6-31G(d)\C2H4S1\SNIKOO\25-Jul-2017\0\\# op
t freq 6-31g(d) m08hx\\CH2=CH-SH\\0,1\S,-0.6861440232,-0.8627579734,0.
\C,-0.0058166788,0.7645414275,0.\H,-0.7851332265,1.528648557,0.\C,1.28
12786304,1.1030807521,0.\H,1.5746602831,2.151744707,0.\H,2.0813061181,
```

0.3612237695,0.\H,0.4891398969,-1.5177252398,0.\\Version=EM64L-G16RevA
.03\State=1-A'\HF=-476.7095624\RMSD=5.973e-09\RMSF=2.838e-05\Dipole=0.
4013143,0.1630447,0.\Quadrupole=0.63149,2.0691805,-2.7006704,-1.511366
8,0.,0.\PG=CS [SG(C2H4S1)]\\@

1\1\GINC-ORC201\FOpt\RM08HX\6-311G(d)\C2H4S1\ROOT\25-Jul-2017\0\\# opt
freq 6-311g(d) m08hx\\CH2=CH-SH\\0,1\S,-0.6876108876,-0.8600742105,0.
\C,-0.0032582665,0.7646113017,0.\H,-0.782569496,1.5263097179,0.\C,1.28
05862307,1.1030340462,0.\H,1.5725644449,2.1506075061,0.\H,2.0802808291
,0.3628780288,0.\H,0.4892981454,-1.5186103901,0.\\Version=EM64L-G16Rev
A.03\State=1-A'\HF=-476.7600953\RMSD=9.019e-09\RMSF=2.804e-05\Dipole=0
.4021669,0.1358752,0.\Quadrupole=0.6975702,2.2089991,-2.9065693,-1.569
066,0.,0.\PG=CS [SG(C2H4S1)]\\@

1\1\GINC-ORC158\FOpt\RM08HX\6-311+G(d)\C2H4S1\SNIKOO\25-Jul-2017\0\\#
opt freq 6-311+g(d) m08hx\\CH2=CH-SH\\0,1\S,-0.6870808388,-0.859138480
4,0.\C,-0.0047936564,0.7638405248,0.\H,-0.7835020153,1.5266976129,0.\C
,1.2806959791,1.1024325096,0.\H,1.5730665001,2.1501494305,0.\H,2.08064
63831,0.3622914406,0.\H,0.4902586481,-1.5175170379,0.\\Version=EM64L-G
16RevA.03\State=1-A'\HF=-476.7627218\RMSD=3.469e-09\RMSF=2.047e-05\Dip
ole=0.3883505,0.124426,0.\Quadrupole=0.7334889,2.1677413,-2.9012302,-1
.6110751,0.,0.\PG=CS [SG(C2H4S1)]\\@

1\1\GINC-ORC276\FOpt\RM08HX\6-311+G(d,p)\C2H4S1\SNIKOO\25-Jul-2017\0\\
opt freq 6-311+g(d,p) m08hx\\CH2=CH-SH\\0,1\S,-0.6847746237,-0.85946
21931,0.\C,-0.0043270888,0.7647944572,0.\H,-0.783005744,1.5266286097,0
.\C,1.2813344408,1.1023526791,0.\H,1.5718131126,2.1495606619,0.\H,2.07
75020531,0.3597585338,0.\H,0.4907488499,-1.5148767487,0.\\Version=EM64
L-G16RevA.03\State=1-A'\HF=-476.7711184\RMSD=2.422e-09\RMSF=2.590e-05\
Dipole=0.3768369,0.124027,0.\Quadrupole=0.7144718,2.092226,-2.8066977,
-1.5803495,0.,0.\PG=CS [SG(C2H4S1)]\\@

1\1\GINC-ORC292\FOpt\RM08HX\6-311G(2d,p)\C2H4S1\SNIKOO\01-Aug-2017\0\\
opt freq 6-311g(2d,p) m08hx\\CH2=CH-SH\\0,1\S,-0.6891980032,-0.85238
93393,0.\C,0.0024722503,0.7668242104,0.\H,-0.7693099848,1.533805514,0.
\C,1.2853677826,1.0919151617,0.\H,1.5857350573,2.1352212891,0.\H,2.072
1612207,0.3413344128,0.\H,0.4772356769,-1.5143842487,0.\\Version=EM64L
-G16RevA.03\State=1-A'\HF=-476.7746737\RMSD=3.702e-09\RMSF=4.438e-05\D
ipole=0.3446754,0.1321703,0.\Quadrupole=0.7157772,2.0889591,-2.8047363
,-1.4294803,0.,0.\PG=CS [SG(C2H4S1)]\\@

1\1\GINC-ORC260\FOpt\RM08HX\6-311+G(df,p)\C2H4S1\SNIKOO\25-Jul-2017\0\
\# opt freq 6-311+g(df,p) m08hx\\CH2=CH-SH\\0,1\S,-0.6841341396,-0.857
3742636,0.\C,-0.0028591552,0.7639344747,0.\H,-0.7817521024,1.525726059
9,0.\C,1.2799488469,1.1021811154,0.\H,1.5708917529,2.1493711714,0.\H,2
.0778809011,0.3613127705,0.\H,0.4893148962,-1.5163953283,0.\\Version=E
M64L-G16RevA.03\State=1-A'\HF=-476.7758148\RMSD=2.493e-09\RMSF=2.555e-
05\Dipole=0.3801215,0.1269083,0.\Quadrupole=0.6602749,2.0344831,-2.694
7581,-1.5749252,0.,0.\PG=CS [SG(C2H4S1)]\\@

1\1\GINC-ORC346\FOpt\RM08HX\6-311+G(2df,p)\C2H4S1\SNIKOO\25-Jul-2017\0
\\# opt freq 6-311+g(2df,p) m08hx\\CH2=CH-SH\\0,1\S,-0.6804980665,-0.8
543667067,0.\C,-0.002845712,0.762400864,0.\H,-0.7798312061,1.525240979
3,0.\C,1.2783188672,1.1010100302,0.\H,1.5694383923,2.1471819877,0.\H,2
.073736015,0.3590327489,0.\H,0.49097271,-1.5117439033,0.\\Version=EM64
L-G16RevA.03\State=1-A'\HF=-476.7814222\RMSD=1.892e-09\RMSF=2.797e-05\
Dipole=0.3302845,0.1139655,0.\Quadrupole=0.7470982,1.9963592,-2.743457

```
4,-1.5089524,0.,0.\PG=CS [SG(C2H4S1)]\\@
```

```
1\1\GINC-ORC291\FOpt\RM08HX\6-311++G(3df,3pd)\C2H4S1\SNIKOO\01-Aug-201
 7\0\\# opt freq 6-311++g(3df,3pd) m08hx\\CH2=CH-SH\\0,1\S,-0.687193469
 4,-0.8468574168,0.\C,0.001610063,0.7638001329,0.\H,-0.7674105586,1.531
 1152657,0.\C,1.2855831555,1.0913867538,0.\H,1.5840611628,2.1328513874,
 0.\H,2.0717950839,0.3436185105,0.\H,0.4760185628,-1.5135876333,0.\\Ver
 sion=EM64L-G16RevA.03\State=1-A'\HF=-476.7868303\RMSD=5.228e-09\RMSF=1
 .262e-04\Dipole=0.3115752,0.1251977,0.\Quadrupole=0.7549855,1.9350601,
 -2.6900456,-1.429942,0.,0.\PG=CS [SG(C2H4S1)]\\@
```

CH_2CHSH QCISD

```
1\1\GINC-ORC281\FOpt\RQCISD-FC\6-311+G(2df,p)\C2H4S1\SNIKOO\19-Aug-201
 7\0\\# opt freq=noraman 6-311+g(2df,p) qcisd\\CH2=CH-SH\\0,1\S,-0.6840
 036893,-0.8656759033,0.\C,-0.0083507368,0.7621287695,0.\H,-0.786558726
 8,1.5199931221,0.\C,1.2772265062,1.1094352171,0.\H,1.5590494881,2.1559
 637179,0.\H,2.0779792712,0.3772770614,0.\H,0.4886698874,-1.5216849847,
 0.\\Version=EM64L-G09RevD.01\State=1-A'\HF=-475.6025846\MP2=-476.09295
 03\MP3=-476.127333\MP4D=-476.1406139\MP4DQ=-476.1262571\MP4SDQ=-476.13
 04037\QCISD=-476.1307161\RMSD=6.627e-09\RMSF=2.034e-05\Dipole=0.343490
 9,0.1398321,0.\PG=CS [SG(C2H4S1)]\\@
```

CH_2CHS^- B3LYP

```
1\1\GINC-ORC283\FOpt\RB3LYP\6-31G(d)\C2H3S1(1-)\SNIKOO\12-Jun-2017\0\\
 # opt freq b3lyp/6-31g(d)\\CH2=CH-S(-)\\-1,1\S,-0.0111294725,0.0362828
 724,0.6466078647\C,-0.1597535256,-0.3535523148,-1.0507779446\H,0.79041
 54731,-0.5236656849,-1.578959865\C,-1.2685732037,-0.4791462327,-1.8197
 477616\H,-1.1967926817,-0.730749153,-2.8801423915\H,-2.2679097491,-0.3
 346666975,-1.4114241543\\Version=EM64L-G09RevD.01\State=1-A\HF=-476.21
 01331\RMSD=4.100e-09\RMSF=1.935e-05\Dipole=-0.1287056,-0.2325471,-1.02
 09191\Quadrupole=1.252873,0.9795018,-2.2323747,-0.5507797,-2.324023,-0
 .6678491\PG=C01 [X(C2H3S1)]\\@
```

```
1\1\GINC-ORC287\FOpt\RB3LYP\6-311G(d)\C2H3S1(1-)\SNIKOO\12-Jun-2017\0\
 \# opt freq b3lyp/6-311g(d)\\CH2=CH-S(-)\\-1,1\S,-0.0146895333,0.03557
 53009,0.6428380967\C,-0.1612286939,-0.3550777934,-1.0576069206\H,0.791
 9252251,-0.5225745627,-1.5739801071\C,-1.2681682402,-0.4792166302,-1.8
 199638143\H,-1.1978289872,-0.7303989831,-2.8788626585\H,-2.2637529294,
 -0.3338045294,-1.4068688513\\Version=EM64L-G09RevD.01\State=1-A\HF=-47
 6.2683329\RMSD=5.697e-09\RMSF=1.790e-05\Dipole=-0.149103,-0.2545572,-1
 .119267\Quadrupole=1.2987773,0.7033303,-2.0021076,-0.5562415,-2.273865
 5,-0.5450362\PG=C01 [X(C2H3S1)]\\@
1\1\GINC-ORC287\FOpt\RB3LYP\6-311+G(d)\C2H3S1(1-)\SNIKOO\12-Jun-2017\0
 \\# opt freq b3lyp/6-311+g(d)\\CH2=CH-S(-)\\-1,1\S,-0.0156006985,0.034
 4691888,0.6379193643\C,-0.1581361429,-0.3544975296,-1.0544788736\H,0.7
 93707891,-0.5224991573,-1.5732846933\C,-1.2699745729,-0.4790980878,-1.
 8198351944\H,-1.1982354231,-0.7301772353,-2.8780000617\H,-2.2655042133
 ,-0.3336943916,-1.406764793\\Version=EM64L-G09RevD.01\State=1-A\HF=-47
 6.2738612\RMSD=2.793e-09\RMSF=1.897e-05\Dipole=-0.1148999,-0.2400431,-
 1.0500687\Quadrupole=1.4491588,0.6214199,-2.0705787,-0.6264452,-2.5237
 441,-0.5279105\PG=C01 [X(C2H3S1)]\\@
```

```
1\1\GINC-ORC335\FOpt\RB3LYP\6-311+G(d,p)\C2H3S1(1-)\SNIKOO\18-Jul-2017
 \0\\# opt freq rb3lyp/6-311+g(d,p)\\CH2=CH-S(-)\\-1,1\S,-1.1301737851,
 -0.1002406067,0.\C,0.5206744209,0.4557775865,0.\H,0.6424472903,1.54505
```

16868,0.\C,1.6715773168,-0.2608641472,0.\H,2.6399146304,0.2366867452,0
.\H,1.6650811267,-1.3473622647,0.\\Version=EM64L-G09RevD.01\State=1-A'
\HF=-476.2794855\RMSD=6.039e-09\RMSF=1.286e-04\Dipole=1.0158542,0.3193
945,0.\Quadrupole=-3.5626839,2.7459104,0.8167734,0.4719681,0.,0.\PG=CS
[SG(C2H3S1)]\\@

1\1\GINC-ORC306\FOpt\RB3LYP\6-311G(2d,p)\C2H3S1(1-)\SNIKOO\27-Jul-2017
\0\\# opt freq rb3lyp/6-311g(2d,p)\\CH2=CH-S(-)\\-1,1\S,-1.133317542,-
0.0998968833,0.\C,0.5263552976,0.4523667397,0.\H,0.6466919553,1.540368
6769,0.\C,1.6690956748,-0.2588468465,0.\H,2.6365808111,0.2396423378,0.
\H,1.6641148031,-1.3445850246,0.\\Version=EM64L-G09RevD.01\State=1-A'\
HF=-476.2771881\RMSD=3.384e-09\RMSF=1.073e-05\Dipole=1.0891605,0.30488
79,0.\Quadrupole=-3.3256441,2.3760052,0.9496389,0.3861937,0.,0.\PG=CS
[SG(C2H3S1)]\\@

1\1\GINC-ORC342\FOpt\RB3LYP\6-311+G(df,p)\C2H3S1(1-)\SNIKOO\18-Jul-201
7\0\\# opt freq rb3lyp/6-311+g(df,p)\\CH2=CH-S(-)\\-1,1\S,-1.128122882
3,-0.1003567268,0.\C,0.5213247236,0.4547925171,0.\H,0.6437726914,1.543
8363994,0.\C,1.6700753467,-0.2601027234,0.\H,2.6382321671,0.2372680742
,0.\H,1.6642389535,-1.3463885404,0.\\Version=EM64L-G09RevD.01\State=1-
A'\HF=-476.2835418\RMSD=6.947e-09\RMSF=9.186e-05\Dipole=1.0187138,0.32
13752,0.\Quadrupole=-3.6251047,2.7493909,0.8757139,0.448593,0.,0.\PG=C
S [SG(C2H3S1)]\\@

1\1\GINC-ORC244\FOpt\RB3LYP\6-311+G(2df,p)\C2H3S1(1-)\SNIKOO\18-Jul-20
17\0\\# opt freq rb3lyp/6-311+g(2df,p) geom=connectivity\\CH2=CH-S(-)\
\-1,1\S,-1.1244486755,-0.0982232884,0.\C,0.520788336,0.4532024005,0.\H
,0.6443054628,1.5412724634,0.\C,1.6690239104,-0.2600728472,0.\H,2.6355
591917,0.2386002979,0.\H,1.6642927747,-1.3457300262,0.\\Version=EM64L-
G09RevD.01\State=1-A'\HF=-476.2868171\RMSD=8.742e-10\RMSF=1.124e-04\Di
pole=0.987701,0.3045676,0.\Quadrupole=-3.5904202,2.7112631,0.8791571,0
.4837452,0.,0.\PG=CS [SG(C2H3S1)]\\@

1\1\GINC-ORC306\FOpt\RB3LYP\6-311++G(3df,3pd)\C2H3S1(1-)\SNIKOO\27-Jul
-2017\0\\# opt freq rb3lyp/6-311++g(3df,3pd)\\CH2=CH-S(-)\\-1,1\S,-1.1
219080322,-0.0975549591,0.\C,0.5205421616,0.4532410591,0.\H,0.64469531
2,1.5392391004,0.\C,1.6688549541,-0.2602423653,0.\H,2.6333574855,0.238
6158936,0.\H,1.6639791191,-1.3442497288,0.\\Version=EM64L-G09RevD.01\S
tate=1-A'\HF=-476.2902732\RMSD=4.406e-09\RMSF=1.586e-04\Dipole=0.96012
96,0.2971697,0.\Quadrupole=-3.7269499,2.6882731,1.0386768,0.4589652,0.
,0.\PG=CS [SG(C2H3S1)]\\@

CH_2CHS^- B3PW91

1\1\GINC-ORC128\FOpt\RB3PW91\6-31G(d)\C2H3S1(1-)\SNIKOO\20-Jul-2017\0\
\# opt freq rb3pw91/6-31g(d)\\CH2=CH-S(-)\\-1,1\S,-1.1272340755,-0.099
4245997,0.\C,0.5219687289,0.452362616,0.\H,0.6525085794,1.5450803564,0
.\C,1.6746655706,-0.2595136197,0.\H,2.6471290656,0.2380877324,0.\H,1.6
72779131,-1.3489484854,0.\\Version=EM64L-G09RevD.01\State=1-A'\HF=-476
.1297307\RMSD=2.217e-09\RMSF=7.457e-05\Dipole=1.0213996,0.2970847,0.\Q
uadrupole=-3.4488762,2.4704881,0.9783881,0.3343342,0.,0.\PG=CS [SG(C2H
3S1)]\\@

1\1\GINC-ORC122\FOpt\RB3PW91\6-311G(d)\C2H3S1(1-)\SNIKOO\20-Jul-2017\0
\\# opt freq rb3pw91/6-311g(d) geom=connectivity\\CH2=CH-S(-)\\-1,1\S,
-1.1224927683,-0.1003737942,0.\C,0.5274920286,0.4530688976,0.\H,0.6488
703908,1.5442554091,0.\C,1.674229814,-0.2593271917,0.\H,2.6455516362,0

.2377481721,0.\H,1.6681658988,-1.3477274929,0.\\Version=EM64L-G09RevD.
01\State=1-A'\HF=-476.1819366\RMSD=4.157e-09\RMSF=4.952e-05\Dipole=1.0
990871,0.312736,0.\Quadrupole=-3.2196773,2.5039171,0.7157603,0.3898219
,0.,0.\PG=CS [SG(C2H3S1)]\\@

1\1\GINC-ORC116\FOpt\RB3PW91\6-311+G(d)\C2H3S1(1-)\SNIKOO\20-Jul-2017\
0\\# opt freq rb3pw91/6-311+g(d) geom=connectivity\\CH2=CH-S(-)\\-1,1\
S,-1.1180605886,-0.099784802,0.\C,0.5241995496,0.4546382908,0.\H,0.646
7994607,1.5457463837,0.\C,1.6746979017,-0.2606319468,0.\H,2.6454933944
,0.2367390921,0.\H,1.6686872823,-1.3490630176,0.\\Version=EM64L-G09Rev
D.01\State=1-A'\HF=-476.1865403\RMSD=6.778e-09\RMSF=1.342e-04\Dipole=1
.028017,0.318082,0.\Quadrupole=-3.4326097,2.770733,0.6618767,0.4627171
,0.,0.\PG=CS [SG(C2H3S1)]\\@

1\1\GINC-ORC109\FOpt\RB3PW91\6-311+G(d,p)\C2H3S1(1-)\SNIKOO\20-Jul-201
7\0\\# opt freq rb3pw91/6-311+g(d,p) geom=connectivity\\CH2=CH-S(-)\\-
1,1\S,-1.1169821007,-0.0995565624,0.\C,0.5248596282,0.4545402625,0.\H,
0.6470892598,1.5448432754,0.\C,1.6754158344,-0.2613569538,0.\H,2.64381
78365,0.2378763301,0.\H,1.6676165417,-1.3487023517,0.\\Version=EM64L-G
09RevD.01\State=1-A'\HF=-476.1921613\RMSD=9.800e-09\RMSF=1.550e-04\Dip
ole=1.0087358,0.3167745,0.\Quadrupole=-3.5081618,2.7386747,0.7694871,0
.4618242,0.,0.\PG=CS [SG(C2H3S1)]\\@

1\1\GINC-ORC272\FOpt\RB3PW91\6-311G(2d,p)\C2H3S1(1-)\SNIKOO\27-Jul-201
7\0\\# opt freq rb3pw91/6-311g(2d,p)\\CH2=CH-S(-)\\-1,1\S,-1.119540393
9,-0.0995950099,0.\C,0.5295112734,0.4515439837,0.\H,0.6517756826,1.540
5986004,0.\C,1.6727550702,-0.2593488568,0.\H,2.6404242021,0.2405095241
,0.\H,1.6668911655,-1.3460642416,0.\\Version=EM64L-G09RevD.01\State=1-
A'\HF=-476.1909636\RMSD=4.343e-09\RMSF=6.712e-05\Dipole=1.0736902,0.30
05129,0.\Quadrupole=-3.2747317,2.4079358,0.8667959,0.4002667,0.,0.\PG=
CS [SG(C2H3S1)]\\@

1\1\GINC-ORC237\FOpt\RB3PW91\6-311+G(df,p)\C2H3S1(1-)\SNIKOO\20-Jul-20
17\0\\# opt freq rb3pw91/6-311+g(df,p) geom=connectivity\\CH2=CH-S(-)\
\-1,1\S,-1.1156237695,-0.0993247311,0.\C,0.525171238,0.4534937014,0.\H
,0.6483367461,1.5437349745,0.\C,1.6739650454,-0.2606101196,0.\H,2.6423
991143,0.2382306648,0.\H,1.6675686258,-1.34788049,0.\\Version=EM64L-G0
9RevD.01\State=1-A'\HF=-476.196032\RMSD=8.307e-09\RMSF=1.658e-04\Dipol
e=1.0107917,0.3185943,0.\Quadrupole=-3.5740357,2.7429196,0.8311161,0.4
368935,0.,0.\PG=CS [SG(C2H3S1)]\\@

1\1\GINC-ORC119\FOpt\RB3PW91\6-311+G(2df,p)\C2H3S1(1-)\SNIKOO\20-Jul-2
017\0\\# opt freq rb3pw91/6-311+g(2df,p) geom=connectivity\\CH2=CH-S(-
)\\-1,1\S,-1.1119006174,-0.0977762591,0.\C,0.5248220356,0.4521757426,0
.\H,0.6489519308,1.541523017,0.\C,1.6727119631,-0.2604248389,0.\H,2.63
99255852,0.2393389169,0.\H,1.6673061027,-1.3471925785,0.\\Version=EM64
L-G09RevD.01\State=1-A'\HF=-476.19949\RMSD=3.208e-09\RMSF=2.267e-04\Di
pole=0.9805431,0.3010088,0.\Quadrupole=-3.5388238,2.7045399,0.8342839,
0.4740514,0.,0.\PG=CS [SG(C2H3S1)]\\@

1\1\GINC-ORC266\FOpt\RB3PW91\6-311++G(3df,3pd)\C2H3S1(1-)\SNIKOO\27-Ju
l-2017\0\\# opt freq rb3pw91/6-311++g(3df,3pd)\\CH2=CH-S(-)\\-1,1\S,-1
.1097097737,-0.0968552795,0.\C,0.5237954371,0.4515459684,0.\H,0.650646
3874,1.5392159634,0.\C,1.6727112272,-0.2607472923,0.\H,2.6373935236,0.
2406444325,0.\H,1.6669801984,-1.3461597925,0.\\Version=EM64L-G09RevD.0
1\State=1-A'\HF=-476.2025814\RMSD=5.159e-09\RMSF=2.517e-05\Dipole=0.95

02713,0.2908683,0.\Quadrupole=-3.6711185,2.6895935,0.981525,0.4516353,
0.,0.\PG=CS [SG(C2H3S1)]\\@

CH_2CHS^- wB97XD

1\1\GINC-ORC270\FOpt\RwB97XD\6-31G(d)\C2H3S1(1-)\SNIKOO\22-Jul-2017\0\
\# opt freq wb97xd/6-31g(d)\\CH2=CH-S(-)\\-1,1\S,-0.6636672072,-0.9187
225862,0.\C,0.0017362194,0.6913925615,0.\H,-0.7352835094,1.5063809567,
0.\C,1.292344866,1.0882537653,0.\H,1.5574246196,2.1467889313,0.\H,2.10
83330116,0.3675073714,0.\\Version=EM64L-G09RevD.01\State=1-A'\HF=-476.
159825\RMSD=5.386e-09\RMSF=6.907e-05\Dipole=0.4835104,1.0337078,0.\Qua
drupole=-0.394441,-0.5497972,0.9442382,-2.9914418,0.,0.\PG=CS [SG(C2H3
S1)]\\@

1\1\GINC-ORC269\FOpt\RwB97XD\6-311G(d)\C2H3S1(1-)\SNIKOO\22-Jul-2017\0
\\# opt freq wb97xd/6-311g(d)\\CH2=CH-S(-)\\-1,1\S,-0.6599892828,-0.91
63688179,0.\C,0.0049179002,0.6966494381,0.\H,-0.7382366268,1.502453619
3,0.\C,1.2921680099,1.088554358,0.\H,1.5579896528,2.1458644202,0.\H,2.
1040383467,0.3644479823,0.\\Version=EM64L-G09RevD.01\State=1-A'\HF=-47
6.212443\RMSD=4.158e-09\RMSF=5.615e-06\Dipole=0.5304125,1.1129886,0.\Q
uadrupole=-0.3419006,-0.3361619,0.6780625,-2.9084664,0.,0.\PG=CS [SG(C
2H3S1)]\\@

1\1\GINC-ORC269\FOpt\RwB97XD\6-311+G(d)\C2H3S1(1-)\SNIKOO\22-Jul-2017\
0\\# opt freq wb97xd/6-311+g(d)\\CH2=CH-S(-)\\-1,1\S,-0.6582462486,-0.
9125222538,0.\C,0.0015733754,0.6945062428,0.\H,-0.7395287908,1.5023502
827,0.\C,1.2933472253,1.0877038621,0.\H,1.557543137,2.1450202231,0.\H,
2.1061993017,0.3645426431,0.\\Version=EM64L-G09RevD.01\State=1-A'\HF=-
476.2169708\RMSD=4.106e-09\RMSF=9.800e-05\Dipole=0.4881258,1.0799665,0
.\Quadrupole=-0.3376668,-0.2526114,0.5902782,-3.1174473,0.,0.\PG=CS [S
G(C2H3S1)]\\@

1\1\GINC-ORC268\FOpt\RwB97XD\6-311+G(d,p)\C2H3S1(1-)\SNIKOO\22-Jul-201
7\0\\# opt freq wb97xd/6-311+g(d,p)\\CH2=CH-S(-)\\-1,1\S,-0.657259437,
-0.9117037669,0.\C,0.0020493821,0.6949637263,0.\H,-0.738504707,1.50230
37468,0.\C,1.2942591402,1.0878036967,0.\H,1.5554973904,2.1445615087,0.
\H,2.1048462313,0.3636720883,0.\\Version=EM64L-G09RevD.01\State=1-A'\H
F=-476.222342\RMSD=4.969e-09\RMSF=1.088e-04\Dipole=0.4754365,1.0645189
,0.\Quadrupole=-0.3877804,-0.3063071,0.6940875,-3.1390597,0.,0.\PG=CS
[SG(C2H3S1)]\\

1\1\GINC-ORC303\FOpt\RwB97XD\6-311G(2d,p)\C2H3S1(1-)\SNIKOO\27-Jul-201
7\0\\# opt freq rwb97xd/6-311g(2d,p)\\CH2=CH-S(-)\\-1,1\S,-0.658348106
8,-0.913515127,0.\C,0.0074808921,0.697240754,0.\H,-0.7334875606,1.5022
706309,0.\C,1.2913575475,1.0874737381,0.\H,1.5522257102,2.1439835663,0
.\H,2.1016595176,0.3641474377,0.\\Version=EM64L-G09RevD.01\State=1-A'\
HF=-476.2213161\RMSD=4.249e-09\RMSF=6.682e-06\Dipole=0.5199769,1.07908
04,0.\Quadrupole=-0.426913,-0.389326,0.8162389,-2.8762334,0.,0.\PG=CS
[SG(C2H3S1)]\\@

1\1\GINC-ORC267\FOpt\RwB97XD\6-311+G(df,p)\C2H3S1(1-)\SNIKOO\22-Jul-20
17\0\\# opt freq wb97xd/6-311+g(df,p)\\CH2=CH-S(-)\\-1,1\S,-0.65689752
86,-0.9107094893,0.\C,0.0030625928,0.6944003701,0.\H,-0.7367728788,1.5
025362294,0.\C,1.2927916673,1.0872279211,0.\H,1.5543059214,2.143817422
6,0.\H,2.1043982259,0.364328546,0.\\Version=EM64L-G09RevD.01\State=1-A
'\HF=-476.2261405\RMSD=6.121e-09\RMSF=1.103e-04\Dipole=0.4758042,1.067

2952,0.\Quadrupole=-0.390202,-0.368716,0.7589181,-3.1707866,0.,0.\PG=C
S [SG(C2H3S1)]\\@

1\1\GINC-ORC266\FOpt\RwB97XD\6-311+G(2df,p)\C2H3S1(1-)\SNIKOO\22-Jul-2
017\0\\# opt freq wb97xd/6-311+g(2df,p)\\CH2=CH-S(-)\\-1,1\S,-0.655921
5363,-0.9069064023,0.\C,0.0037801564,0.6931975727,0.\H,-0.734637311,1.
5014754027,0.\C,1.291947899,1.0864014552,0.\H,1.5517626324,2.142729466
7,0.\H,2.1039561595,0.364703505,0.\\Version=EM64L-G09RevD.01\State=1-A
'\HF=-476.2296744\RMSD=6.074e-09\RMSF=7.728e-05\Dipole=0.4671765,1.027
1357,0.\Quadrupole=-0.4275626,-0.3257037,0.7532664,-3.1308279,0.,0.\PG
=CS [SG(C2H3S1)]\\@

1\1\GINC-ORC270\FOpt\RwB97XD\6-311++G(3df,3pd)\C2H3S1(1-)\SNIKOO\27-Ju
l-2017\0\\# opt freq rwb97xd/6-311++g(3df,3pd)\\CH2=CH-S(-)\\-1,1\S,-0
.6546513473,-0.9046631109,0.\C,0.0036980255,0.6930490149,0.\H,-0.73280
19632,1.5005878719,0.\C,1.292043571,1.0861970136,0.\H,1.5501598924,2.1
411632819,0.\H,2.1024398216,0.3652669286,0.\\Version=EM64L-G09RevD.01\
State=1-A'\HF=-476.2330629\RMSD=5.423e-09\RMSF=1.051e-04\Dipole=0.4616
315,0.9970947,0.\Quadrupole=-0.4480119,-0.4127439,0.8607558,-3.1635316
,0.,0.\PG=CS [SG(C2H3S1)]\\@

CH_2CHS^- M062X

1\1\GINC-ORC214\FOpt\RM062X\6-31G(d)\C2H3S1(1-)\SNIKOO\13-Jun-2017\0\\
opt freq 6-31g(d) m062x\\CH2=CH-S(-)\\-1,1\S,-1.1212231657,-0.107710
4666,0.\C,0.5259702027,0.453984322,0.\H,0.6563291411,1.5448466706,0.\C
,1.6727422996,-0.2562761618,0.\H,2.6448874103,0.2365001277,0.\H,1.6631
11112,-1.343700492,0.\\Version=EM64L-G09RevD.01\State=1-A'\HF=-476.130
1736\RMSD=3.924e-09\RMSF=7.477e-05\Dipole=1.068617,0.3012424,0.\Quadru
pole=-3.3808344,2.4381217,0.9427127,0.3007084,0.,0.\PG=CS [SG(C2H3S1)]
\\@

1\1\GINC-ORC200\FOpt\RM062X\6-311G(d)\C2H3S1(1-)\SNIKOO\13-Jun-2017\0\
\# opt freq 6-311g(d) m062x\\CH2=CH-S(-)\\-1,1\S,-1.1205731897,-0.1068
060266,0.\C,0.5292006574,0.4557637612,0.\H,0.6487162895,1.5442722351,0
.\C,1.6695692805,-0.2579657091,0.\H,2.6412248574,0.2327600686,0.\H,1.6
531591049,-1.3441643293,0.\\Version=EM64L-G09RevD.01\State=1-A'\HF=-47
6.1905676\RMSD=3.025e-09\RMSF=9.156e-05\Dipole=1.1703999,0.3230314,0.\
Quadrupole=-3.1357545,2.5242968,0.6114577,0.3818067,0.,0.\PG=CS [SG(C2
H3S1)]\\@

1\1\GINC-ORC302\FOpt\RM062X\6-311+G(d)\C2H3S1(1-)\SNIKOO\13-Jun-2017\0
\\# opt freq 6-311+g(d) m062x\\CH2=CH-S(-)\\-1,1\S,-1.1183013308,-0.10
74969416,0.\C,0.5230287133,0.4570850039,0.\H,0.6437511176,1.5456748982
,0.\C,1.6684628368,-0.2577607534,0.\H,2.6384191177,0.235560073,0.\H,1.
6541605453,-1.34401328,0.\\Version=EM64L-G09RevD.01\State=1-A'\HF=-476
.1949692\RMSD=6.828e-09\RMSF=7.071e-05\Dipole=1.1029402,0.331801,0.\Qu
adrupole=-3.3190284,2.7718196,0.5472088,0.4361633,0.,0.\PG=CS [SG(C2H3
S1)]\\@

\1\GINC-ORC26\FOpt\RM062X\6-311+G(d,p)\C2H3S1(1-)\SNIKOO\19-Jul-2017\
0\\# opt freq 6-311+g(d,p) geom=connectivity m062x\\CH2=CH-S(-)\\-1,1\
S,-0.6680747554,-0.9091843586,0.\C,0.0002961851,0.69231443,0.\H,-0.732
3409801,1.5056671771,0.\C,1.296073357,1.072540953,0.\H,1.5704606251,2.
1245146254,0.\H,2.0959225683,0.3386391732,0.\\Version=EM64L-G09RevD.01
\State=1-A'\HF=-476.199216\RMSD=6.492e-09\RMSF=1.414e-04\Dipole=0.4758

899,1.0356488,0.\Quadrupole=-0.39454,-0.2353493,0.6298893,-3.099981,0.
,0.\PG=CS [SG(C2H3S1)]\\@

1\1\GINC-ORC303\FOpt\RM062X\6-311G(2d,p)\C2H3S1(1-)\SNIKOO\27-Jul-2017
\0\\# opt freq 6-311g(2d,p) m062x\\CH2=CH-S(-)\\-1,1\S,-0.6697856061,-
0.9113230213,0.\C,0.0054667928,0.6942092406,0.\H,-0.7259181387,1.50671
34263,0.\C,1.2931232307,1.0710520862,0.\H,1.5665483639,2.1224236131,0.
\H,2.0915583574,0.336983655,0.\\Version=EM64L-G09RevD.01\State=1-A'\HF
=-476.1985484\RMSD=3.065e-09\RMSF=1.324e-04\Dipole=0.5302565,1.0631044
,0.\Quadrupole=-0.4248648,-0.2967484,0.7216132,-2.8252016,0.,0.\PG=CS
[SG(C2H3S1)]\\@

1\1\GINC-ORC24\FOpt\RM062X\6-311+G(df,p)\C2H3S1(1-)\SNIKOO\19-Jul-2017
\0\\# opt freq m062x/6-311+g(df,p) geom=connectivity\\CH2=CH-S(-)\\-1,
1\S,-0.6677895034,-0.9082771075,0.\C,0.0014153992,0.6916046421,0.\H,-0
.7304321363,1.5059811545,0.\C,1.2946688747,1.0720671814,0.\H,1.5690215
892,2.1241424796,0.\H,2.0954527766,0.3389736499,0.\\Version=EM64L-G09R
evD.01\State=1-A'\HF=-476.203231\RMSD=2.632e-09\RMSF=1.232e-04\Dipole=
0.4747551,1.0366444,0.\Quadrupole=-0.3992308,-0.3067536,0.7059844,-3.1
359964,0.,0.\PG=CS [SG(C2H3S1)]\\@

1\1\GINC-ORC21\FOpt\RM062X\6-311+G(2df,p)\C2H3S1(1-)\SNIKOO\19-Jul-201
7\0\\# opt freq m062x/6-311+g(2df,p) geom=connectivity\\CH2=CH-S(-)\\-
1,1\S,-0.6666022666,-0.9047956927,0.\C,0.0022543663,0.6907124849,0.\H,
-0.7282849281,1.5050484916,0.\C,1.2940596313,1.0714015912,0.\H,1.56631
25285,2.1229630662,0.\H,2.0945976687,0.3391620587,0.\\Version=EM64L-G0
9RevD.01\State=1-A'\HF=-476.2069408\RMSD=3.742e-09\RMSF=9.323e-05\Dipo
le=0.4680231,0.9965915,0.\Quadrupole=-0.4305318,-0.2556208,0.6861526,-
3.0900005,0.,0.\PG=CS [SG(C2H3S1)]\\@

1\1\GINC-ORC292\FOpt\RM062X\6-311++G(3df,3pd)\C2H3S1(1-)\SNIKOO\27-Jul
-2017\0\\# opt freq m062x/6-311++g(3df,3pd)\\CH2=CH-S(-)\\-1,1\S,-0.66
72299235,-0.9025697889,0.\C,0.0018600946,0.690011636,0.\H,-0.725831669
6,1.5040614799,0.\C,1.2942161977,1.0700684043,0.\H,1.5652235437,2.1201
520649,0.\H,2.0927547571,0.3383352039,0.\\Version=EM64L-G09RevD.01\Sta
te=1-A'\HF=-476.2103781\RMSD=2.275e-09\RMSF=1.154e-04\Dipole=0.4579131
,0.9637913,0.\Quadrupole=-0.464084,-0.3479842,0.8120682,-3.1260593,0.,
0.\PG=CS [SG(C2H3S1)]\\@

CH_2CHS^- M08HX

1\1\GINC-ORC268\FOpt\RM08HX\6-31G(d)\C2H3S1(1-)\SNIKOO\25-Jul-2017\0\\
opt freq M08HX/6-31g(d)\\CH2=CH-S(-)\\-1,1\S,-0.6599453365,-0.923643
5665,0.\C,-0.0025730832,0.6886711494,0.\H,-0.7434368472,1.5076347669,0
.\C,1.2872229231,1.0880091704,0.\H,1.5557275214,2.148656087,0.\H,2.104
4328224,0.3636193928,0.\\Version=EM64L-G16RevA.03\State=1-A'\HF=-476.1
46518\RMSD=3.214e-09\RMSF=6.524e-05\Dipole=0.4855218,0.9991727,0.\Quad
rupole=-0.3320364,-0.544534,0.8765704,-2.8981709,0.,0.\PG=CS [SG(C2H3S
1)]\\@

1\1\GINC-ORC207\FOpt\RM08HX\6-311G(d)\C2H3S1(1-)\ROOT\25-Jul-2017\0\\#
opt freq 6-311g(d) m08hx\\CH2=CH-S(-)\\-1,1\S,-0.6561998251,-0.919592
1456,0.\C,-0.0001110465,0.6933817461,0.\H,-0.7464564175,1.503130553,0.
\C,1.2878588175,1.0879473654,0.\H,1.5552383564,2.1478785601,0.\H,2.101
0981151,0.3602009209,0.\\Version=EM64L-G16RevA.03\State=1-A'\HF=-476.2
084877\RMSD=1.679e-09\RMSF=5.789e-05\Dipole=0.5103682,1.0639683,0.\Qua
drupole=-0.2823356,-0.3209811,0.6033167,-2.8382068,0.,0.\PG=CS [SG(C2H

```
3S1)]\\@

1\1\GINC-ORC168\FOpt\RM08HX\6-311+G(d)\C2H3S1(1-)\SNIKOO\25-Jul-2017\0
 \\# opt freq 6-311+g(d) m08hx\\CH2=CH-S(-)\\-1,1\S,-0.6539763778,-0.91
 55450389,0.\C,-0.0037831264,0.691473747,0.\H,-0.7493555084,1.502493999
 4,0.\C,1.2893203426,1.0873550537,0.\H,1.5562117746,2.1471836355,0.\H,2
 .1030108954,0.3599856033,0.\\Version=EM64L-G16RevA.03\State=1-A'\HF=-4
 76.2133731\RMSD=8.864e-09\RMSF=9.529e-05\Dipole=0.4358146,0.9916034,0.
 \Quadrupole=-0.2728551,-0.2786352,0.5514903,-3.077581,0.,0.\PG=CS [SG(
 C2H3S1)]\\@

1\1\GINC-ORC201\FOpt\RM08HX\6-311+G(d,p)\C2H3S1(1-)\ROOT\25-Jul-2017\0
 \\# opt freq 6-311+g(d,p) m08hx\\CH2=CH-S(-)\\-1,1\S,-0.6529505121,-0.
 914792791,0.\C,-0.0032428557,0.6920048296,0.\H,-0.747970633,1.50242323
 47,0.\C,1.2902754766,1.087537849,0.\H,1.554013893,2.1465467224,0.\H,2.
 1013026311,0.3592271553,0.\\Version=EM64L-G16RevA.03\State=1-A'\HF=-47
 6.2179787\RMSD=6.406e-09\RMSF=9.940e-05\Dipole=0.4263794,0.9806626,0.\
 Quadrupole=-0.3208628,-0.3288348,0.6496977,-3.0965846,0.,0.\PG=CS [SG(
 C2H3S1)]\\@

1\1\GINC-ORC219\FOpt\RM08HX\6-311G(2d,p)\C2H3S1(1-)\SNIKOO\01-Aug-2017
 \0\\# opt freq 6-311g(2d,p) m08hx\\CH2=CH-S(-)\\-1,1\S,-0.6705752779,-
 0.9101132616,0.\C,0.0055929374,0.6928584473,0.\H,-0.7264784684,1.51209
 75465,0.\C,1.2948625359,1.0685461918,0.\H,1.5714931055,2.123069752,0.\
 H,2.0952061674,0.3302923239,0.\\Version=EM64L-G16RevA.03\State=1-A'\HF
 =-476.217113\RMSD=3.525e-09\RMSF=1.353e-04\Dipole=0.5196682,1.0317459,
 0.\Quadrupole=-0.4309201,-0.3081669,0.739087,-2.799383,0.,0.\PG=CS [SG
 (C2H3S1)]\\@

1\1\GINC-ORC268\FOpt\RM08HX\6-311+G(df,p)\C2H3S1(1-)\SNIKOO\25-Jul-201
 7\0\\# opt freq 6-311+g(df,p) m08hx\\CH2=CH-S(-)\\-1,1\S,-0.6529876124
 ,-0.913608537,0.\C,-0.0020404884,0.6910366446,0.\H,-0.7460674373,1.502
 6864116,0.\C,1.2886667913,1.0868862997,0.\H,1.552590839,2.1459032969,0
 .\H,2.1012659078,0.3600428841,0.\\Version=EM64L-G16RevA.03\State=1-A'\
 HF=-476.2222897\RMSD=4.488e-09\RMSF=1.207e-04\Dipole=0.4251208,0.98052
 63,0.\Quadrupole=-0.3218625,-0.3939343,0.7157968,-3.1286535,0.,0.\PG=C
 S [SG(C2H3S1)]\\@

1\1\GINC-ORC260\FOpt\RM08HX\6-311+G(2df,p)\C2H3S1(1-)\SNIKOO\25-Jul-20
 17\0\\# opt freq 6-311+g(2df,p) m08hx\\CH2=CH-S(-)\\-1,1\S,-0.65116242
 43,-0.9099367816,0.\C,-0.0012715863,0.690249455,0.\H,-0.7434507623,1.5
 018322915,0.\C,1.2875707421,1.086145394,0.\H,1.5499884504,2.1442344438
 ,0.\H,2.0997535805,0.3604221972,0.\\Version=EM64L-G16RevA.03\State=1-A
 '\HF=-476.2262269\RMSD=3.559e-09\RMSF=1.745e-04\Dipole=0.4197771,0.943
 0253,0.\Quadrupole=-0.3559486,-0.3578343,0.7137829,-3.0786252,0.,0.\PG
 =CS [SG(C2H3S1)]\\@

1\1\GINC-ORC218\FOpt\RM08HX\6-311++G(3df,3pd)\C2H3S1(1-)\ROOT\01-Aug-2
 017\0\\# opt freq 6-311++g(3df,3pd) m08hx\\CH2=CH-S(-)\\-1,1\S,-0.6677
 109459,-0.9003997024,0.\C,0.0019320341,0.6886817699,0.\H,-0.7264181847
 ,1.5087051606,0.\C,1.2964884787,1.06769831,0.\H,1.5699675252,2.1201667
 977,0.\H,2.0958420926,0.3318986641,0.\\Version=EM64L-G16RevA.03\State=
 1-A'\HF=-476.2302555\RMSD=6.531e-09\RMSF=1.363e-04\Dipole=0.41654,0.90
 86939,0.\Quadrupole=-0.4645678,-0.3605984,0.8251662,-3.1471269,0.,0.\P
 G=CS [SG(C2H3S1)]\\@
```

CH_2CHS^- QCISD

1\1\GINC-ORC285\FOpt\RQCISD-FC\6-311+G(2df,p)\C2H3S1(1-)\SNIKOO\19-Aug
-2017\0\\# opt freq=noraman 6-311+g(2df,p) qcisd\\CH2=CH-S(-)\\-1,1\S,
-0.6627323766,-0.9196342262,0.\C,-0.0020419982,0.6898081493,0.\H,-0.73
78247122,1.502469497,0.\C,1.2917277179,1.0831373609,0.\H,1.5536500253,
2.1400189163,0.\H,2.1046713439,0.3613743027,0.\\Version=EM64L-G09RevD.
01\State=1-A'\HF=-475.0398029\MP2=-475.5396155\MP3=-475.5675065\MP4D=-
475.5804561\MP4DQ=-475.5651696\MP4SDQ=-475.569981\QCISD=-475.5701444\R
MSD=8.997e-09\RMSF=3.713e-05\Dipole=0.4944769,1.0275285,0.\PG=CS [SG(C
2H3S1)]\\@

$CH_2CHS^{\cdot}$ B3LYP

1\1\GINC-ORC3\FOpt\UB3LYP\6-31G(d)\C2H3S1(2)\SNIKOO\19-Aug-2017\0\\# o
pt freq ub3lyp/6-31g(d)\\CH2=CH-SS\\0,2\C,1.260870827,-0.4658153757,-0
.0000025733\H,0.8719085932,-1.4866400991,-0.0015905634\C,2.5784076815,
-0.2406925985,-0.0003632234\H,3.27216098,-1.0755204952,-0.0022458154\H
,3.0037663338,0.7583273802,0.0011650482\S,-0.0328714159,0.7199331896,0
.0028493283\\Version=EM64L-G09RevD.01\State=2-A'\HF=-476.1367874\S2=0.
752757\S2-1=0.\S2A=0.750004\RMSD=8.099e-09\RMSF=9.179e-05\Dipole=0.237
5363,-0.3112768,-0.0006898\Quadrupole=0.7944115,1.636804,-2.4312155,0.
5937677,-0.0008073,0.0068801\PG=CS [SG(C2H3S1)]\\@

1\1\GINC-ORC186\FOpt\UB3LYP\6-311G(2d,p)\C2H3S1(2)\SNIKOO\19-Aug-2017\
0\\# opt freq ub3lyp/6-311g(2d,p)\\CH2=CH-SS\\0,2\C,1.2660701358,-0.46
28919775,-0.0000003834\H,0.8749203879,-1.4796910625,-0.0015799636\C,2.
5760735275,-0.2411002098,-0.0003626001\H,3.2656699474,-1.0751121986,-0
.0022413445\H,2.9993430566,0.7551458537,0.0011619521\S,-0.0278340557,0
.713241596,0.0028345404\\Version=EM64L-G09RevD.01\State=2-A'\HF=-476.1
909169\S2=0.753658\S2-1=0.\S2A=0.750006\RMSD=5.967e-09\RMSF=1.227e-04\
Dipole=0.2060367,-0.2814201,-0.0006186\Quadrupole=0.8349612,1.7176178,
-2.5525791,0.4964556,-0.0010735,0.0072952\PG=CS [SG(C2H3S1)]\\@

1\1\GINC-ORC32\FOpt\UB3LYP\6-311+G(2df,p)\C2H3S1(2)\SNIKOO\19-Aug-2017
\0\\# opt freq ub3lyp/6-311+g(2df,p)\\CH2=CH-SS\\0,2\C,1.2632427281,-0
.4597885354,0.0000067584\H,0.8753321234,-1.4784944676,-0.0015780767\C,
2.5757493931,-0.2404648805,-0.000361285\H,3.2644203202,-1.0750706765,-
0.0022405496\H,3.0011656028,0.7550090993,0.0011606575\S,-0.025667168,0
.7084014621,0.0028246963\\Version=EM64L-G09RevD.01\State=2-A'\HF=-476.
1981611\S2=0.754192\S2-1=0.\S2A=0.750009\RMSD=7.134e-09\RMSF=1.246e-04
\Dipole=0.1779079,-0.26544,-0.000574\Quadrupole=0.7717941,1.7403761,-2
.5121702,0.5592845,-0.0009022,0.0072276\PG=CS [SG(C2H3S1)]\\@

1\1\GINC-ORC185\FOpt\UB3LYP\6-311++G(3df,3pd)\C2H3S1(2)\SNIKOO\19-Aug-
2017\0\\# opt freq ub3lyp/6-311++g(3df,3pd)\\CH2=CH-SS\\0,2\C,1.262537
6321,-0.4586369757,0.0000092099\H,0.8774135518,-1.4767769933,-0.001576
2285\C,2.5755799537,-0.2402482532,-0.0003608026\H,3.2619890168,-1.0745
11255,-0.0022381533\H,3.0006332837,0.7534954778,0.0011582773\S,-0.0239
104384,0.7062700008,0.0028198981\\Version=EM64L-G09RevD.01\State=2-A'\
HF=-476.201366\S2=0.754278\S2-1=0.\S2A=0.750009\RMSD=5.476e-09\RMSF=1.
183e-04\Dipole=0.18076,-0.2581009,-0.0005626\Quadrupole=0.735456,1.697
1355,-2.4325915,0.5683098,-0.0008193,0.0070043\PG=CS [SG(C2H3S1)]\\@

$CH_2CHS^{\cdot}$ B3PW91

1\1\GINC-ORC142\FOpt\UB3PW91\6-31G(d)\C2H3S1(2)\SNIKOO\19-Aug-2017\0\\

opt freq ub3pw91/6-31g(d)\\CH2=CH-SS\\0,2\C,1.2597877801,-0.46287141
8,0.0000032787\H,0.8725367147,-1.4849366424,-0.0015879014\C,2.57728303
5,-0.2394203238,-0.0003603155\H,3.2687183582,-1.0763462057,-0.00224529
47\H,3.0035948012,0.7593682935,0.0011669953\S,-0.0276776898,0.71379829
77,0.0028354385\\Version=EM64L-G09RevD.01\State=2-A'\HF=-476.0543631\S
2=0.753138\S2-1=0.\S2A=0.750004\RMSD=8.624e-09\RMSF=1.038e-04\Dipole=0
.2397877,-0.3140275,-0.0006959\Quadrupole=0.84239,1.6900634,-2.5324534
,0.5903542,-0.0008995,0.0071564\PG=CS [SG(C2H3S1)]\\@

1\1\GINC-ORC157\FOpt\UB3PW91\6-311G(2d,p)\C2H3S1(2)\SNIKOO\19-Aug-2017
\0\\# opt freq ub3pw91/6-311g(2d,p)\\CH2=CH-SS\\0,2\C,1.2648053202,-0.
4600431316,0.0000054046\H,0.8746835523,-1.4785485313,-0.0015777984\C,2
.5750839011,-0.2396647371,-0.0003594803\H,3.2628265295,-1.0763297326,-
0.0022418653\H,2.9991805234,0.7571483409,0.0011656013\S,-0.022336827,0
.7070297931,0.0028203389\\Version=EM64L-G09RevD.01\State=2-A'\HF=-476.
1052145\S2=0.754267\S2-1=0.\S2A=0.750007\RMSD=3.633e-09\RMSF=1.343e-04
\Dipole=0.198875,-0.278823,-0.0006098\Quadrupole=0.8484758,1.7460101,-
2.5944858,0.4880003,-0.0011205,0.0074249\PG=CS [SG(C2H3S1)]\\@

1\1\GINC-ORC162\FOpt\UB3PW91\6-311+G(2df,p)\C2H3S1(2)\SNIKOO\19-Aug-20
17\0\\# opt freq ub3pw91/6-311+g(2df,p)\\CH2=CH-SS\\0,2\C,1.2623790935
,-0.4572751925,0.0000117192\H,0.8750338134,-1.4775728299,-0.0015762681
\C,2.5747505155,-0.2391510803,-0.0003583759\H,3.2618354884,-1.07629041
61,-0.0022412236\H,3.0008352788,0.7570435514,0.0011644603\S,-0.0205911
901,0.7028379689,0.002811889\\Version=EM64L-G09RevD.01\State=2-A'\HF=-
476.112153\S2=0.754949\S2-1=0.\S2A=0.750011\RMSD=3.956e-09\RMSF=1.385e
-04\Dipole=0.1767428,-0.2671042,-0.0005762\Quadrupole=0.7811042,1.7525
068,-2.533611,0.5445164,-0.0009462,0.0072957\PG=CS [SG(C2H3S1)]\\@

1\1\GINC-ORC167\FOpt\UB3PW91\6-311++G(3df,3pd)\C2H3S1(2)\SNIKOO\19-Aug
-2017\0\\# opt freq ub3pw91/6-311++g(3df,3pd)\\CH2=CH-SS\\0,2\C,1.2616
435416,-0.4559885588,0.0000144281\H,0.8772916208,-1.4760819565,-0.0015
749241\C,2.5745254058,-0.2389486563,-0.0003578866\H,3.2594660273,-1.07
59744529,-0.0022392952\H,3.0004711428,0.7556823573,0.0011622536\S,-0.0
191547388,0.7009032686,0.002807625\\Version=EM64L-G09RevD.01\State=2-A
'\HF=-476.1149606\S2=0.755086\S2-1=0.\S2A=0.750012\RMSD=9.545e-09\RMSF
=1.312e-04\Dipole=0.181146,-0.2602273,-0.0005666\Quadrupole=0.7506761,
1.7152283,-2.4659045,0.5558856,-0.0008693,0.0071028\PG=CS [SG(C2H3S1)]
\\@

$CH_2CHS^{\cdot}$ wB97XD

1\1\GINC-ORC119\FOpt\UwB97XD\6-31G(d)\C2H3S1(2)\SNIKOO\19-Aug-2017\0\\
opt freq uwb97xd/6-31g(d)\\CH2=CH-SS\\0,2\C,1.2614736779,-0.46760278
25,-0.0000060947\H,0.8747279564,-1.4880630801,-0.0015947169\C,2.574562
3172,-0.2403499888,-0.000360396\H,3.2681484454,-1.0745138692,-0.002241
7125\H,2.996267712,0.7597796624,0.0011719542\S,-0.0209371094,0.7203420
597,0.0028431669\\Version=EM64L-G09RevD.01\State=2-A'\HF=-476.0847833\
S2=0.752958\S2-1=0.\S2A=0.750004\RMSD=9.846e-09\RMSF=1.319e-04\Dipole=
0.2682283,-0.3328826,-0.0007458\Quadrupole=0.8672369,1.6903783,-2.5576
152,0.618228,-0.0008788,0.0071855\PG=CS [SG(C2H3S1)]\\@

1\1\GINC-ORC32\FOpt\UwB97XD\6-311G(2d,p)\C2H3S1(2)\SNIKOO\19-Aug-2017\
0\\# opt freq uwb97xd/6-311g(2d,p)\\CH2=CH-SS\\0,2\C,1.2663862366,-0.4
644196522,-0.0000032782\H,0.8784911208,-1.4819871249,-0.0015861009\C,2
.5727452767,-0.2405159487,-0.000359642\H,3.2611896844,-1.0755627236,-0
.0022395589\H,2.9917455809,0.7578673551,0.0011711686\S,-0.0163148999,0

```
.7142100958,0.0028296122\\Version=EM64L-G09RevD.01\State=2-A'\HF=-476.
1353852\S2=0.753738\S2-1=0.\S2A=0.750007\RMSD=7.286e-09\RMSF=3.111e-05
\Dipole=0.2219425,-0.2935813,-0.0006493\Quadrupole=0.8725868,1.7515808
,-2.6241676,0.5108213,-0.001111,0.0074743\PG=CS [SG(C2H3S1)]\\@
```

```
1\1\GINC-ORC164\FOpt\UwB97XD\6-311+G(2df,p)\C2H3S1(2)\SNIKOO\19-Aug-20
 17\0\\# opt freq uwb97xd/6-311+g(2df,p)\\CH2=CH-SS\\0,2\C,1.2641059241
 ,-0.4615895778,0.0000030625\H,0.8787093559,-1.4807985989,-0.0015841166
 \C,2.572508113,-0.239939854,-0.0003584823\H,3.2601132367,-1.0756221675
 ,-0.0022390432\H,2.9935008901,0.7577880492,0.0011700148\S,-0.014694520
 2,0.7097541504,0.0028207656\\Version=EM64L-G09RevD.01\State=2-A'\HF=-4
 76.1420476\S2=0.754174\S2-1=0.\S2A=0.750009\RMSD=8.240e-09\RMSF=3.394e
 -05\Dipole=0.2021932,-0.2837342,-0.0006205\Quadrupole=0.8158739,1.7520
 675,-2.5679414,0.563191,-0.0009529,0.0073451\PG=CS [SG(C2H3S1)]\\@
```

```
1\1\GINC-ORC32\FOpt\UwB97XD\6-311++G(3df,3pd)\C2H3S1(2)\SNIKOO\19-Aug-
 2017\0\\# opt freq uwb97xd/6-311++g(3df,3pd)\\CH2=CH-SS\\0,2\C,1.26350
 26402,-0.4603241081,0.0000056575\H,0.8810308295,-1.4790937104,-0.00158
 24293\C,2.5723792394,-0.2398035481,-0.0003581659\H,3.2575942247,-1.075
 2193663,-0.0022368743\H,2.9927735011,0.7562769527,0.0011677517\S,-0.01
 30374355,0.7077557816,0.0028162612\\Version=EM64L-G09RevD.01\State=2-A
 '\HF=-476.1452888\S2=0.75425\S2-1=0.\S2A=0.750009\RMSD=5.638e-09\RMSF=
 3.811e-05\Dipole=0.2049055,-0.2736633,-0.0006041\Quadrupole=0.8004816,
 1.7177236,-2.5182053,0.5659496,-0.0009104,0.0071942\PG=CS [SG(C2H3S1)]
 \\@
```

$CH_2CHS^\bullet$ M062X

```
1\1\GINC-ORC180\FOpt\UM062X\6-31G(d)\C2H3S1(2)\SNIKOO\19-Aug-2017\0\\#
  opt freq=noraman m062x/6-31g(d)\\CH2=CH-S\\0,2\C,1.260854459,-0.47447
 99903,-0.0000179478\H,0.8722882319,-1.4935249116,-0.0016030064\C,2.572
 232089,-0.2424174168,-0.0003627219\H,3.2721327415,-1.0702684675,-0.002
 2364742\H,2.9832723297,0.7620210934,0.0011834335\S,-0.0065368516,0.728
 261694,0.0028489177\\Version=EM64L-G09RevD.01\State=2-A'\HF=-476.05371
 21\S2=0.75296\S2-1=0.\S2A=0.750006\RMSD=6.850e-09\RMSF=5.852e-05\Dipol
 e=0.249527,-0.3237764,-0.0007189\Quadrupole=0.8956557,1.639442,-2.5350
 977,0.6140182,-0.0008897,0.0070575\PG=CS [SG(C2H3S1)]\\@
```

```
1\1\GINC-ORC167\FOpt\UM062X\6-311G(2d,p)\C2H3S1(2)\SNIKOO\19-Aug-2017\
 0\\# opt freq=noraman m062x/6-311g(2d,p)\\CH2=CH-S\\0,2\C,1.265619479,
 -0.4726142471,-0.0000173851\H,0.8754194454,-1.4878393301,-0.0015947187
 \C,2.5705904359,-0.2424557063,-0.0003618424\H,3.2659556113,-1.07053230
 92,-0.0022333778\H,2.9787593472,0.7597349258,0.0011819788\S,-0.0021013
 192,0.7232986683,0.0028375462\\Version=EM64L-G09RevD.01\State=2-A'\HF=
 -476.1112181\S2=0.753969\S2-1=0.\S2A=0.75001\RMSD=3.271e-09\RMSF=2.849
 e-05\Dipole=0.2169423,-0.2901475,-0.0006404\Quadrupole=0.9405268,1.735
 0721,-2.6755989,0.5159414,-0.0011708,0.0075334\PG=CS [SG(C2H3S1)]\\@
```

```
1\1\GINC-ORC164\FOpt\UM062X\6-311+G(2df,p)\C2H3S1(2)\SNIKOO\19-Aug-201
 7\0\\# opt freq=noraman m062x/6-311+g(2df,p)\\CH2=CH-S\\0,2\C,1.263023
 1673,-0.469397105,-0.0000101748\H,0.8758618514,-1.4867911223,-0.001593
 113\C,2.5702128667,-0.2418098111,-0.0003604778\H,3.2647728999,-1.07077
 42989,-0.002233125\H,2.9809753739,0.7597398911,0.0011807088\S,-0.00060
 31596,0.7186244475,0.0028283826\\Version=EM64L-G09RevD.01\State=2-A'\H
 F=-476.1179021\S2=0.754155\S2-1=0.\S2A=0.750011\RMSD=9.456e-09\RMSF=2.
 915e-05\Dipole=0.1950642,-0.2796659,-0.0006091\Quadrupole=0.8772315,1.
```

7222539,-2.5994854,0.5639806,-0.0010051,0.0073477\PG=CS [SG(C2H3S1)]\\
@

1\1\GINC-ORC161\FOpt\UM062X\6-311++G(3df,3pd)\C2H3S1(2)\SNIKOO\19-Aug-
2017\0\\# opt freq=noraman m062x/6-311++g(3df,3pd)\\CH2=CH-S\\0,2\C,1.
2626012926,-0.4687255046,-0.0000087389\H,0.8777473674,-1.4850723213,-0
.0015911494\C,2.5702044783,-0.2417453739,-0.0003603585\H,3.262638473,-
1.0701097926,-0.0022307134\H,2.9798597474,0.758197049,0.0011786133\S,0
.0011916408,0.7170479447,0.0028245477\\Version=EM64L-G09RevD.01\State=
2-A'\HF=-476.1208624\S2=0.754066\S2-1=0.\S2A=0.750011\RMSD=4.601e-09\R
MSF=2.742e-05\Dipole=0.194704,-0.2703108,-0.0005923\Quadrupole=0.86284
3,1.6713125,-2.5341555,0.565778,-0.0009559,0.0071403\PG=CS [SG(C2H3S1)
]\\@

$CH_2CHS^{\cdot}$ M08HX

1\1\GINC-ORC25\FOpt\UM08HX\6-31G(d)\C2H3S1(2)\SNIKOO\19-Aug-2017\0\\#
opt freq=noraman m08hx/6-31g(d)\\CH2=CH-S\\0,2\C,1.2594806909,-0.46934
33031,-0.0000080349\H,0.8728341682,-1.4946561732,-0.00160533\C,2.57265
56457,-0.2412736095,-0.0003609355\H,3.2726546989,-1.0751325878,-0.0022
454116\H,2.9921183789,0.7644843593,0.0011827019\S,-0.015500583,0.72551
33157,0.002849211\\Version=EM64L-G16RevA.03\State=2-A'\HF=-476.0656945
\S2=0.75304\S2-1=0.\S2A=0.750005\RMSD=6.009e-09\RMSF=1.057e-04\Dipole=
0.2494559,-0.3142409,-0.0007019\Quadrupole=0.9236697,1.6741914,-2.5978
611,0.5945669,-0.0009766,0.0072419\PG=CS [SG(C2H3S1)]\\@

1\1\GINC-ORC25\FOpt\UM08HX\6-311G(2d,p)\C2H3S1(2)\SNIKOO\19-Aug-2017\0
\\# opt freq=noraman 6-311g(2d,p) m08hx\\CH2=CH-S\\0,2\C,1.2646672967,
-0.4682094959,-0.000009015\H,0.8750160635,-1.4886302136,-0.0015958901\
C,2.5707220195,-0.2417970736,-0.000360749\H,3.2670023266,-1.0744668874
,-0.0022409677\H,2.9854050133,0.7622099671,0.001182538\S,-0.00856972,0
.7204857047,0.0028362847\\Version=EM64L-G16RevA.03\State=2-A'\HF=-476.
1257081\S2=0.753793\S2-1=0.\S2A=0.750009\RMSD=5.681e-09\RMSF=1.260e-04
\Dipole=0.2098769,-0.2824441,-0.0006226\Quadrupole=0.9225143,1.7168734
,-2.6393877,0.4968904,-0.0011734,0.0074478\PG=CS [SG(C2H3S1)]\\@

1\1\GINC-ORC25\FOpt\UM08HX\6-311G(2d,p)\C2H3S1(2)\SNIKOO\19-Aug-2017\0
\\# opt freq=noraman 6-311g(2d,p) m08hx\\CH2=CH-S\\0,2\C,1.2646672967,
-0.4682094959,-0.000009015\H,0.8750160635,-1.4886302136,-0.0015958901\
C,2.5707220195,-0.2417970736,-0.000360749\H,3.2670023266,-1.0744668874
,-0.0022409677\H,2.9854050133,0.7622099671,0.001182538\S,-0.00856972,0
.7204857047,0.0028362847\\Version=EM64L-G16RevA.03\State=2-A'\HF=-476.
1257081\S2=0.753793\S2-1=0.\S2A=0.750009\RMSD=5.681e-09\RMSF=1.260e-04
\Dipole=0.2098769,-0.2824441,-0.0006226\Quadrupole=0.9225143,1.7168734
,-2.6393877,0.4968904,-0.0011734,0.0074478\PG=CS [SG(C2H3S1)]\\@

1\1\GINC-ORC19\FOpt\UM08HX\6-311+G(2df,p)\C2H3S1(2)\SNIKOO\19-Aug-2017
\0\\# opt freq=noraman 6-311+g(2df,p) m08hx\\CH2=CH-S\\0,2\C,1.2621878
339,-0.4651542699,-0.0000021596\H,0.8752307805,-1.4877077498,-0.001594
3762\C,2.5704147712,-0.2411133621,-0.0003593578\H,3.266054166,-1.07460
66388,-0.0022406686\H,2.9880445137,0.7621494435,0.0011809073\S,-0.0076
890658,0.7160245784,0.0028278558\\Version=EM64L-G16RevA.03\State=2-A'\
HF=-476.1332271\S2=0.754471\S2-1=0.\S2A=0.750012\RMSD=2.626e-09\RMSF=1
.353e-04\Dipole=0.1879024,-0.270215,-0.0005882\Quadrupole=0.8595355,1.
7125822,-2.5721177,0.5449126,-0.0010129,0.007293\PG=CS [SG(C2H3S1)]\\@

1\1\GINC-ORC338\FOpt\UM08HX\6-311+G(3df,3pd)\C2H3S1(2)\SNIKOO\19-Aug-2
017\0\\# opt freq=noraman 6-311+g(3df,3pd) m08hx\\CH2=CH-S\\0,2\C,1.26
19921053,-0.4645003452,-0.0000008856\H,0.8775520369,-1.4855012833,-0.0
015917983\C,2.5706664002,-0.2410855643,-0.0003594536\H,3.2634712919,-1
.0736608737,-0.0022374989\H,2.9869921478,0.7598960748,0.0011775138\S,-
0.0064309826,0.7144439932,0.0028243235\\Version=EM64L-G16RevA.03\State
=2-A'\HF=-476.1370106\S2=0.754626\S2-1=0.\S2A=0.750013\RMSD=9.133e-09\
RMSF=1.238e-04\Dipole=0.1960654,-0.2672035,-0.0005876\Quadrupole=0.855
4447,1.690679,-2.5461237,0.5570251,-0.0009741,0.007201\PG=CS [SG(C2H3S
1)]\\@

$CH_2CHS^{\cdot}$ QCISD

1\1\GINC-ORC213\FOpt\UQCISD-FC\6-311+G(2df,p)\C2H3S1(2)\SNIKOO\20-Aug-
2017\0\\# opt freq=noraman 6-311+g(2df,p) qcisd\\1\\0,2\C,0.0185393543
,0.7192344805,0.\H,-0.7102146361,1.5287466804,0.\C,1.3235469086,0.9793
05119,0.\H,1.6713229937,2.0054196425,0.\H,2.0729859346,0.1957243127,0.
\S,-0.739087555,-0.856994235,0.\\Version=EM64L-G09RevD.01\State=2-A'\H
F=-474.9977714\MP2=-475.4524678\MP3=-475.4863835\MP4D=-475.4991752\MP4
DQ=-475.4859566\PUHF=-475.0018091\PMP2-0=-475.4552617\PMP3-0=-475.4880
736\MP4SDQ=-475.4902576\QCISD=-475.4912766\S2=0.770598\S2-1=0.757811\S
2A=0.750262\RMSD=1.889e-09\RMSF=6.667e-05\Dipole=0.0961583,0.335403,0.
\PG=CS [SG(C2H3S1)]\\@

CH_2CHSSH B3LYP

1\1\GINC-ORC289\FOpt\RB3LYP\6-31G(d)\C2H4S2\SNIKOO\13-Jun-2017\0\\# op
t freq b3lyp/6-31g(d)\\CH2=CH-SS(-)\\0,1\C,1.3117985231,-0.6064697249,
-0.4510998254\H,1.141023323,-1.5181567376,-1.0187328691\C,2.4839625277
,-0.3265277641,0.1216216971\H,3.3290859175,-1.0003004989,0.0080908007\
H,2.6451697176,0.5802031856,0.6982053808\S,-0.0377350826,0.5563246775,
-0.4425876911\S,-1.7117690128,-0.6718367851,-0.1327130706\H,-1.6554051
537,-0.7818343826,1.2176261077\\Version=EM64L-G09RevD.01\State=1-A\HF=
-874.9672391\RMSD=8.800e-09\RMSF=5.376e-06\Dipole=0.4974804,-0.3402393
,0.4094156\Quadrupole=1.2471041,-1.0527776,-0.1943265,0.0535171,-1.081
3624,0.2790842\PG=C01 [X(C2H4S2)]\\@

1\1\GINC-ORC299\FOpt\RB3LYP\6-311G(d)\C2H4S2\SNIKOO\13-Jun-2017\0\\# o
pt freq b3lyp/6-311g(d)\\CH2=CH-SSH\\0,1\C,-1.2743149739,0.4090992019,
-0.345591062\H,-1.0468819516,1.1723348721,-1.0833452465\C,-2.453202899
5,0.3309726992,0.2653183449\H,-3.2501551691,1.0250078291,0.0189442371\
H,-2.6690935348,-0.4273819561,1.0105467773\S,-0.0052546635,-0.81407250
08,-0.1023983935\S,1.731392186,0.3790249521,0.0313438862\H,1.634035006
4,0.7388069026,1.3353254566\\Version=EM64L-G09RevD.01\State=1-A\HF=-87
5.0369646\RMSD=3.816e-09\RMSF=2.690e-05\Dipole=-0.4852162,0.4454425,0.
3499335\Quadrupole=1.144569,-1.1507081,0.006139,0.0948103,0.9247176,-0
.189901\PG=C01 [X(C2H4S2)]\\@

1\1\GINC-ORC290\FOpt\RB3LYP\6-311+G(d)\C2H4S2\SNIKOO\13-Jun-2017\0\\#
opt freq b3lyp/6-311+g(d)\\CH2=CH-SSH\\0,1\C,1.2718081195,-0.403587916
7,-0.3246699554\H,1.0201029609,-1.1511036546,-1.0708601344\C,2.4705985
786,-0.3430065555,0.2516901446\H,3.2557525841,-1.0342220832,-0.0366547
351\H,2.7146201411,0.3964233989,1.007432\S,0.0109803252,0.8107721617,-
0.0127675516\S,-1.7422916385,-0.3609328132,-0.0073104345\H,-1.69353607
09,-0.8032765373,1.2744486665\\Version=EM64L-G09RevD.01\State=1-A\HF=-
875.0402548\RMSD=5.893e-09\RMSF=6.974e-06\Dipole=0.4066384,-0.4386925,
0.3035515\Quadrupole=1.2133581,-1.05696,-0.1563982,0.2624392,-0.962007

1,0.2379506\PG=C01 [X(C2H4S2)]\\@

1\1\GINC-ORC96\FOpt\RB3LYP\6-311+G(d,p)\C2H4S2\SNIKOO\19-Jul-2017\0\\#
opt freq rb3lyp/6-311+g(d,p) geom=connectivity\\CH2=CH-SSH\\0,1\C,1.2
692224175,-0.4277046371,-0.3085632728\H,1.0061116915,-1.2221644767,-0.
9991415952\C,2.476851302,-0.3277124701,0.2432116731\H,3.2566846579,-1.
036010331,-0.0127161297\H,2.7289480497,0.4603283138,0.9439357559\S,0.0
108564507,0.8030096737,-0.0556476889\S,-1.7463378169,-0.3626081214,0.0
117092477\H,-1.6937547524,-0.7276849512,1.3140200099\\Version=EM64L-G0
9RevD.01\State=1-A\HF=-875.0499219\RMSD=4.880e-09\RMSF=2.478e-05\Dipol
e=0.3871058,-0.4149677,0.3133694\Quadrupole=1.2005057,-1.0011752,-0.19
93305,0.2477297,-0.9710677,0.2717459\PG=C01 [X(C2H4S2)]\\@

1\1\GINC-ORC173\FOpt\RB3LYP\6-311G(2d,p)\C2H4S2\SNIKOO\27-Jul-2017\0\\
opt freq rb3lyp/6-311g(2d,p)\\CH2=CH-SSH\\0,1\C,1.2709831982,-0.4281
909794,-0.3363052703\H,1.0324511273,-1.2087251475,-1.0495541181\C,2.45
628715,-0.3349180723,0.2519082533\H,3.2481224858,-1.0331853851,0.00770
21802\H,2.6802113119,0.4406381248,0.9744746511\S,0.0035980772,0.794215
0619,-0.0954942696\S,-1.7363240254,-0.3670276678,0.0404737498\H,-1.646
7473251,-0.7033529346,1.3436028237\\Version=EM64L-G09RevD.01\State=1-A
\HF=-875.0593716\RMSD=3.304e-09\RMSF=1.059e-05\Dipole=0.43771,-0.41066
33,0.2784575\Quadrupole=1.2320726,-1.0917351,-0.1403375,0.174157,-0.78
74599,0.1902676\PG=C01 [X(C2H4S2)]\\@

1\1\GINC-ORC85\FOpt\RB3LYP\6-311+G(df,p)\C2H4S2\SNIKOO\19-Jul-2017\0\\
opt freq rb3lyp/6-311+g(df,p) geom=connectivity\\CH2=CH-SSH\\0,1\C,1
.2692562232,-0.4285536005,-0.2948507236\H,0.9964117316,-1.2295916574,-
0.9739284385\C,2.482236895,-0.3241963726,0.2373814617\H,3.2583661788,-
1.0349551907,-0.0218463558\H,2.7453428974,0.4698908606,0.926918047\S,0
.0111468904,0.7978169666,-0.0330458706\S,-1.741354072,-0.3517821971,-0
.0004011374\H,-1.7128247443,-0.7391758089,1.2965810172\\Version=EM64L-
G09RevD.01\State=1-A\HF=-875.0579459\RMSD=4.408e-09\RMSF=1.291e-05\Dip
ole=0.3689977,-0.4163428,0.3068063\Quadrupole=1.1619139,-0.9429728,-0.
2189411,0.2355721,-1.0056623,0.2636883\PG=C01 [X(C2H4S2)]\\@

1\1\GINC-ORC87\FOpt\RB3LYP\6-311+G(2df,p)\C2H4S2\SNIKOO\19-Jul-2017\0\
\# opt freq rb3lyp/6-311+g(2df,p) geom=connectivity\\CH2=CH-SSH\\0,1\C
,1.2676395698,-0.4309171676,-0.2938078406\H,0.9984765202,-1.2314320924
,-0.9739024088\C,2.480285829,-0.3243712679,0.236435794\H,3.2590484007,
-1.0300751424,-0.0260163161\H,2.7405183619,0.4690473217,0.926936221\S,
0.0085183184,0.7852089141,-0.0250712064\S,-1.7353398742,-0.3450328093,
-0.0007986859\H,-1.7105651258,-0.7329747561,1.2930324428\\Version=EM64
L-G09RevD.01\State=1-A\HF=-875.0696256\RMSD=6.653e-09\RMSF=2.324e-05\D
ipole=0.3421962,-0.3921835,0.2556761\Quadrupole=1.2200794,-0.9310672,-
0.2890122,0.3044961,-0.8322642,0.276507\PG=C01 [X(C2H4S2)]\\@

1\1\GINC-ORC175\FOpt\RB3LYP\6-311++G(3df,3pd)\C2H4S2\SNIKOO\27-Jul-201
7\0\\# opt freq rb3lyp/6-311++g(3df,3pd)\\CH2=CH-SSH\\0,1\C,1.26686893
51,-0.4338378446,-0.2805657107\H,0.9911728923,-1.2386468174,-0.9504403
185\C,2.487198091,-0.3212428035,0.2310930659\H,3.261887475,-1.02588860
37,-0.038925732\H,2.7555731499,0.4760456655,0.9112571909\S,0.011293288
3,0.7773509861,-0.000473331\S,-1.7340973418,-0.3343075526,-0.010559180
9\H,-1.7313144897,-0.7400200298,1.2754220164\\Version=EM64L-G09RevD.01
\State=1-A\HF=-875.0751444\RMSD=4.549e-09\RMSF=3.201e-05\Dipole=0.3279
266,-0.3782692,0.2226369\Quadrupole=1.2889749,-0.8894263,-0.3995486,0.
3203775,-0.7418571,0.2930943\PG=C01 [X(C2H4S2)]\\@

CH_2CHSSH B3PW91

```
1\1\GINC-ORC38\FOpt\RB3PW91\6-31G(d)\C2H4S2\SNIKOO\20-Jul-2017\0\\# op
 t freq b3pw91/6-31g(d)\\CH2=CH-SS(-)\\0,1\C,-1.2705720246,0.4182036564
 ,-0.3205302083\H,-1.0301811195,1.1942111889,-1.0444177245\C,-2.4690942
 035,0.3230337875,0.2567670959\H,-3.2649373842,1.0138860283,-0.00888769
 95\H,-2.7003205856,-0.4474352896,0.9876410836\S,-0.0059201486,-0.79303
 02415,-0.0524999058\S,1.7250124103,0.3557147536,0.0088752911\H,1.68253
 70556,0.7492081165,1.3031960676\\Version=EM64L-G09RevD.01\State=1-A\HF
 =-874.8357923\RMSD=4.686e-09\RMSF=2.832e-05\Dipole=-0.4693913,0.459359
 4,0.3361721\Quadrupole=1.2938307,-1.1303941,-0.1634367,0.1546251,1.090
 9164,-0.1986342\PG=C01 [X(C2H4S2)]\\@

1\1\GINC-ORC37\FOpt\RB3PW91\6-311G(d)\C2H4S2\SNIKOO\20-Jul-2017\0\\# o
 pt freq b3pw91/6-311g(d) geom=connectivity\\CH2=CH-SS(-)\\0,1\C,-1.266
 4464485,0.4106399164,-0.3256783967\H,-1.0246134275,1.1829098066,-1.050
 8981477\C,-2.4571162403,0.3259741366,0.2602974462\H,-3.247950719,1.024
 4187423,0.0046674111\H,-2.688024187,-0.4417363823,0.9924177051\S,-0.00
 88140534,-0.8071826159,-0.0721133205\S,1.7157526348,0.3668077533,0.011
 9848242\H,1.6437364408,0.7519606431,1.3094664783\\Version=EM64L-G09Rev
 D.01\State=1-A\HF=-874.9014054\RMSD=3.134e-09\RMSF=2.219e-05\Dipole=-0
 .452123,0.455533,0.3580514\Quadrupole=1.1761206,-1.0822224,-0.0938983,
 0.1610792,0.9739854,-0.2225601\PG=C01 [X(C2H4S2)]\\@

1\1\GINC-ORC25\FOpt\RB3PW91\6-311+G(d)\C2H4S2\SNIKOO\20-Jul-2017\0\\#
 opt freq rb3pw91/6-311+g(d) geom=connectivity\\CH2=CH-SS(-)\\0,1\C,-1.
 26753158,0.4130392697,-0.2998415322\H,-1.0068016844,1.1980399621,-1.00
 49060814\C,-2.4754375405,0.3185154044,0.2508267508\H,-3.258416286,1.02
 09242249,-0.0179201715\H,-2.7288493354,-0.4599319766,0.964232159\S,-0.
 0121615221,-0.8016896499,-0.0272249506\S,1.7222968067,0.3577217892,-0.
 013756565\H,1.6934251417,0.7671729761,1.278734391\\Version=EM64L-G09Re
 vD.01\State=1-A\HF=-874.9046875\RMSD=9.294e-09\RMSF=3.224e-05\Dipole=-
 0.3876731,0.4362763,0.3289307\Quadrupole=1.2541645,-0.9887502,-0.26541
 44,0.287499,1.0336502,-0.2930366\PG=C01 [X(C2H4S2)]\\@

1\1\GINC-ORC17\FOpt\RB3PW91\6-311+G(d,p)\C2H4S2\SNIKOO\20-Jul-2017\0\\
 # opt freq rb3pw91/6-311+g(d,p) geom=connectivity\\CH2=CH-SS(-)\\0,1\C
 ,-1.2681972789,0.4146931255,-0.2905931721\H,-0.9999818008,1.2025677309
 ,-0.9887582294\C,-2.4811925483,0.316335447,0.2480183226\H,-3.259864943
 7,1.0200751942,-0.0262805946\H,-2.7382968195,-0.4658655691,0.954810195
 3\S,-0.013587988,-0.7991526939,-0.0100228767\S,1.7254495201,0.35552163
 4,-0.0223952876\H,1.7021958592,0.7696171314,1.2653656424\\Version=EM64
 L-G09RevD.01\State=1-A\HF=-874.9143114\RMSD=6.356e-09\RMSF=3.059e-05\D
 ipole=-0.3649082,0.4317919,0.3133557\Quadrupole=1.2531759,-0.9578859,-
 0.29529,0.3316062,1.0095379,-0.2743089\PG=C01 [X(C2H4S2)]\\@

1\1\GINC-ORC189\FOpt\RB3PW91\6-311G(2d,p)\C2H4S2\SNIKOO\27-Jul-2017\0\
 \# opt freq rb3pw91/6-311g(2d,p)\\CH2=CH-SS(-)\\0,1\C,-1.2697498603,0.
 4164489183,-0.3172728123\H,-1.0250528748,1.1904673576,-1.0373194753\C,
 -2.462242,0.3237148993,0.2560654615\H,-3.2541734269,1.0160144153,-0.00
 75777429\H,-2.6914396165,-0.4468356294,0.98382278\S,-0.0078661183,-0.7
 904001578,-0.0470864111\S,1.7178457121,0.3586426285,0.0045505167\H,1.6
 592021846,0.7457395681,1.2949616833\\Version=EM64L-G09RevD.01\State=1-
 A\HF=-874.9249299\RMSD=8.137e-09\RMSF=5.731e-05\Dipole=-0.4072593,0.42
 20208,0.2747595\Quadrupole=1.2687093,-1.0385077,-0.2302017,0.2585463,0
 .8050704,-0.2114459\PG=C01 [X(C2H4S2)]\\@
```

```
1\1\GINC-ORC325\FOpt\RB3PW91\6-311G(df,p)\C2H4S2\SNIKOO\11-Sep-2018\0\
 \# opt freq=noraman b3pw91/6-311g(df,p)\\CH2=CH-SSH\\0,1\C,1.258594911
 2,-0.4140914741,-0.3088578499\H,1.0005921611,-1.1984186449,-1.01432820
 88\C,2.4592523721,-0.3183696355,0.2483742665\H,3.2437595429,-1.0202844
 015,-0.0129178316\H,2.7035104165,0.4619731557,0.9612285749\S,0.0024330
 072,0.801197766,-0.0449522128\S,-1.7228649415,-0.3541463377,0.00120832
 68\H,-1.6710004695,-0.7528924282,1.2925299349\\Version=EM64L-G09RevE.0
 1\State=1-A\HF=-874.9190094\RMSD=3.034e-09\RMSF=1.897e-05\Dipole=0.418
 6136,-0.44822,0.3354175\Quadrupole=1.1332308,-1.008776,-0.1244547,0.16
 68292,-0.9850249,0.2028646\PG=C01 [X(C2H4S2)]\\@
```

```
1\1\GINC-ORC14\FOpt\RB3PW91\6-311+G(df,p)\C2H4S2\SNIKOO\20-Jul-2017\0\
 \# opt freq rb3pw91/6-311+g(df,p) geom=connectivity\\CH2=CH-SS(-)\\0,1
 \C,-1.2684216849,0.4160858666,-0.276252121\H,-0.9907851893,1.210979252
 5,-0.9626439276\C,-2.4871354108,0.3124096183,0.2419411825\H,-3.2623063
 443,1.018208938,-0.03594842\H,-2.7554425674,-0.4763323978,0.9369947297
 \S,-0.0137838019,-0.7927596936,0.0140640011\S,1.7209840747,0.344332909
 5,-0.0344242977\H,1.7234149238,0.7808675065,1.246412853\\Version=EM64L
 -G09RevD.01\State=1-A\HF=-874.9223124\RMSD=7.251e-09\RMSF=4.957e-05\Di
 pole=-0.3469214,0.4331987,0.3061599\Quadrupole=1.2200677,-0.8994581,-0
 .3206096,0.3225014,1.0493809,-0.2664808\PG=C01 [X(C2H4S2)]\\@
```

```
1\1\GINC-ORC157\FOpt\RB3PW91\6-311+G(2df,p)\C2H4S2\SNIKOO\20-Jul-2017\
 0\\# opt freq rb3pw91/6-311+g(2df,p) geom=connectivity\\CH2=CH-SS(-)\\
 0,1\C,-1.2674628183,0.4189270532,-0.2775188352\H,-0.9937729881,1.21254
 46161,-0.9659018174\C,-2.4845905571,0.3130266116,0.2412848069\H,-3.263
 291612,1.0133916735,-0.0387007292\H,-2.7482324069,-0.4744654503,0.9388
 632605\S,-0.011432132,-0.7798717592,0.0206438545\S,1.7157304001,0.3371
 851073,-0.0334518142\H,1.7195761142,0.7730541479,1.2449252742\\Version
 =EM64L-G09RevD.01\State=1-A\HF=-874.9350675\RMSD=1.938e-09\RMSF=8.416e
 -06\Dipole=-0.3235425,0.4062867,0.2518952\Quadrupole=1.2649471,-0.8903
 852,-0.3745619,0.3771411,0.8510402,-0.2882077\PG=C01 [X(C2H4S2)]\\@
```

```
1\1\GINC-ORC94\FOpt\RB3PW91\6-311++G(3df,3pd)\C2H4S2\SNIKOO\27-Jul-201
 7\0\\# opt freq rb3pw91/6-311++g(3df,3pd)\\CH2=CH-SS(-)\\0,1\C,-1.2668
 404731,0.4219928713,-0.2636339778\H,-0.9860839756,1.2209428248,-0.9408
 683614\C,-2.491520547,0.3093666686,0.2361265882\H,-3.2660057364,1.0092
 629981,-0.050860337\H,-2.7637281287,-0.4830624245,0.9224211823\S,-0.01
 41459879,-0.7717842362,0.0456098075\S,1.7149822152,0.3255959428,-0.043
 7736178\H,1.7398666335,0.7814773551,1.2251227162\\Version=EM64L-G09Rev
 D.01\State=1-A\HF=-874.9400363\RMSD=3.302e-09\RMSF=1.179e-05\Dipole=-0
 .3117375,0.39181,0.2176832\Quadrupole=1.3418386,-0.8488898,-0.4929489,
 0.3928608,0.7478163,-0.3073856\PG=C01 [X(C2H4S2)]\\@
```

CH_2CHSSH wB97XD

```
1\1\GINC-ORC265\FOpt\RwB97XD\6-31G(d)\C2H4S2\SNIKOO\22-Jul-2017\0\\# o
 pt freq rwb97xd/6-31g(d)\\CH2=CH-SS(-)\\0,1\C,1.2632507454,-0.42208754
 29,-0.3216972394\H,1.0213327618,-1.2164261331,-1.023303836\C,2.4585949
 521,-0.3140586209,0.2514780526\H,3.2492754166,-1.0174062805,0.00832629
 43\H,2.6913631025,0.4753711993,0.960410713\S,-0.0032846644,0.796441640
 5,-0.0789651942\S,-1.7201006681,-0.3633525797,0.0247668059\H,-1.658847
 6459,-0.7192586827,1.3232294038\\Version=EM64L-G09RevD.01\State=1-A\HF
 =-874.8978659\RMSD=2.468e-09\RMSF=2.982e-05\Dipole=0.4879882,-0.465634
```

4,0.3542567\Quadrupole=1.2774571,-1.1302839,-0.1471732,0.09677,-1.0885
677,0.2033803\PG=C01 [X(C2H4S2)]\\@

1\1\GINC-ORC265\FOpt\RwB97XD\6-311G(d)\C2H4S2\SNIKOO\22-Jul-2017\0\\#
opt freq rwb97xd/6-311g(d)\\CH2=CH-SS(-)\\0,1\C,1.2599293185,-0.415156
4729,-0.3304450834\H,1.0208856395,-1.2037512461,-1.0374060694\C,2.4453
852187,-0.3180089401,0.2564746195\H,3.2334224217,-1.0259868987,0.02280
40867\H,2.6742823741,0.4667765879,0.9701552801\S,-0.001904254,0.808063
8776,-0.1033370462\S,-1.7106798766,-0.3764349911,0.0316685325\H,-1.619
7368419,-0.7162789166,1.3343306802\\Version=EM64L-G09RevD.01\State=1-A
\HF=-874.9636129\RMSD=9.300e-09\RMSF=4.259e-05\Dipole=0.4732423,-0.457
5444,0.3780693\Quadrupole=1.158254,-1.1043927,-0.0538613,0.0936815,-0.
9854311,0.2364654\PG=C01 [X(C2H4S2)]\\@

1\1\GINC-ORC263\FOpt\RwB97XD\6-311+G(d)\C2H4S2\SNIKOO\22-Jul-2017\0\\#
opt freq rwb97xd/6-311+g(d)\\CH2=CH-SS(-)\\0,1\C,1.2608608302,-0.4171
048906,-0.3059682038\H,1.0036260973,-1.2177532373,-0.9932351247\C,2.46
3173121,-0.3109192152,0.2469199508\H,3.2440341356,-1.0228949478,0.0013
75431\H,2.713260394,0.4836694642,0.9428704092\S,0.0019576718,0.8042074
83,-0.0601927742\S,-1.7183476713,-0.3676784498,0.0069100487\H,-1.66698
05785,-0.7323032066,1.305565263\\Version=EM64L-G09RevD.01\State=1-A\HF
=-874.9668671\RMSD=6.858e-09\RMSF=3.400e-05\Dipole=0.4118639,-0.439714
9,0.3497218\Quadrupole=1.2617184,-1.0234776,-0.2382408,0.212619,-1.042
9331,0.2948954\PG=C01 [X(C2H4S2)]\\@

1\1\GINC-ORC262\FOpt\RwB97XD\6-311+G(d,p)\C2H4S2\SNIKOO\22-Jul-2017\0\
\# opt freq rwb97xd/6-311+g(d,p)\\CH2=CH-SS(-)\\0,1\C,1.2613215064,-0.
4188104977,-0.2948344581\H,0.9955248573,-1.2232847118,-0.9734351204\C,
2.4697474982,-0.3085035439,0.2434465531\H,3.2459019511,-1.0222146889,-
0.0078863384\H,2.7248413898,0.4911094956,0.930560428\S,0.0043869559,0.
8026616396,-0.0402290718\S,-1.7219132932,-0.3636955653,-0.003496444\H,
-1.6782268656,-0.7380391277,1.2901194516\\Version=EM64L-G09RevD.01\Sta
te=1-A\HF=-874.9762347\RMSD=7.737e-09\RMSF=4.214e-05\Dipole=0.3884817,
-0.4358945,0.3312369\Quadrupole=1.2670493,-0.9793298,-0.2877194,0.2642
307,-1.0189969,0.2739226\PG=C01 [X(C2H4S2)]\\@

1\1\GINC-ORC258\FOpt\RwB97XD\6-311G(2d,p)\C2H4S2\SNIKOO\27-Jul-2017\0\
\# opt freq rwb97xd/6-311g(2d,p)\\CH2=CH-SS(-)\\0,1\C,1.2632504185,-0.
4192310089,-0.3287850704\H,1.026340897,-1.2073905821,-1.034558015\C,2.
4463528691,-0.3169227341,0.2542293564\H,3.2362702208,-1.0194807706,0.0
163915435\H,2.6688374682,0.4673620299,0.9684922534\S,-0.0033921475,0.7
941637853,-0.0903133316\S,-1.7116383403,-0.3717428812,0.0303884813\H,-
1.6244373859,-0.7075348382,1.3283997825\\Version=EM64L-G09RevD.01\Stat
e=1-A\HF=-874.9868479\RMSD=4.504e-09\RMSF=2.900e-05\Dipole=0.4334865,-
0.4228982,0.2967947\Quadrupole=1.2429239,-1.0714009,-0.171523,0.185357
,-0.8047611,0.215483\PG=C01 [X(C2H4S2)]\\@

1\1\GINC-ORC322\FOpt\RwB97XD\6-311G(df,p)\C2H4S2\SNIKOO\11-Sep-2018\0\
\# opt freq=noraman wb97xd/6-311g(df,p)\\CH2=CH-SSH\\0,1\C,1.257663265
4,-0.4109792092,-0.3165982658\H,1.0053311336,-1.1932160367,-1.02523414
48\C,2.4504083386,-0.3197736411,0.2503472581\H,3.2324038034,-1.0263191
688,-0.0025476986\H,2.6900005179,0.4583317319,0.9665801614\S,-0.000770
0246,0.8060613426,-0.0548988535\S,-1.7145659002,-0.3603146393,0.008269
1752\H,-1.646194134,-0.7488223793,1.296367368\\Version=EM64L-G09RevE.0
1\State=1-A\HF=-874.9809842\RMSD=4.399e-09\RMSF=2.392e-05\Dipole=0.441
2718,-0.4610812,0.3440162\Quadrupole=1.1250918,-1.0414102,-0.0836816,0
.1132355,-0.9766116,0.1897568\PG=C01 [X(C2H4S2)]\\@

1\1\GINC-ORC260\FOpt\RwB97XD\6-311+G(df,p)\C2H4S2\SNIKOO\22-Jul-2017\0
\\# opt freq rwb97xd/6-311+g(df,p)\\CH2=CH-SS(-)\\0,1\C,1.2616892613,-
0.4198291789,-0.279207251\H,0.9852630183,-1.2315485212,-0.9448962114\C
,2.4766336826,-0.3047564189,0.2367404923\H,3.2488456746,-1.0207828625,
-0.0192295432\H,2.7443285524,0.5015818263,0.9109120176\S,0.0056348913,
0.7971584604,-0.0133100081\S,-1.7186344758,-0.3519435291,-0.0169794366
\H,-1.7021766046,-0.750656776,1.2702149404\\Version=EM64L-G09RevD.01\S
tate=1-A\HF=-874.9842602\RMSD=3.467e-09\RMSF=2.087e-05\Dipole=0.370276
7,-0.4391821,0.3232872\Quadrupole=1.2409106,-0.9191587,-0.321752,0.260
6012,-1.0638867,0.2626051\PG=C01 [X(C2H4S2)]\\@

1\1\GINC-ORC259\FOpt\RwB97XD\6-311+G(2df,p)\C2H4S2\SNIKOO\22-Jul-2017\
0\\# opt freq rwb97xd/6-311+g(2df,p)\\CH2=CH-SS(-)\\0,1\C,1.2605320792
,-0.4215140045,-0.2885157936\H,0.9941958918,-1.2292691294,-0.961880750
7\C,2.4692083517,-0.3066970015,0.2394474819\H,3.2470597264,-1.01653952
41,-0.0146476712\H,2.7259100168,0.4951491462,0.9224431051\S,0.00212311
54,0.7863609867,-0.0224021875\S,-1.7122783325,-0.3497217844,-0.0088867
499\H,-1.6851668487,-0.7385456888,1.2786875659\\Version=EM64L-G09RevD.
01\State=1-A\HF=-874.9969916\RMSD=8.688e-09\RMSF=8.287e-06\Dipole=0.35
27319,-0.4100156,0.2742412\Quadrupole=1.2696982,-0.928777,-0.3409212,0
.2992109,-0.8559906,0.2782341\PG=C01 [X(C2H4S2)]\\@

1\1\GINC-ORC256\FOpt\RwB97XD\6-311++G(3df,3pd)\C2H4S2\SNIKOO\27-Jul-20
17\0\\# opt freq rwb97xd/6-311++g(3df,3pd)\\CH2=CH-SSH\\0,1\C,1.259755
8768,-0.4243574313,-0.2708601381\H,0.9845041323,-1.2392821435,-0.92945
83358\C,2.4781621908,-0.3024964115,0.2331238225\H,3.2505628728,-1.0124
432416,-0.0294627175\H,2.7458995827,0.5061060106,0.9010283957\S,0.0060
508091,0.7790295264,0.0081877233\S,-1.7127687745,-0.335923739,-0.02287
94039\H,-1.7105826899,-0.75140957,1.2545656537\\Version=EM64L-G09RevD.
01\State=1-A\HF=-875.002353\RMSD=7.703e-09\RMSF=2.569e-05\Dipole=0.333
0444,-0.3983372,0.2385451\Quadrupole=1.3692738,-0.8742507,-0.4950231,0
.3434075,-0.7625795,0.2929279\PG=C01 [X(C2H4S2)]\\@

CH_2CHSSH M062X

1\1\GINC-ORC290\FOpt\RM062X\6-31G(d)\C2H4S2\SNIKOO\13-Jun-2017\0\\# op
t freq 6-31g(d) m062x\\CH2=CH-SSH\\0,1\C,-1.3209130768,0.5654644318,-0
.4877266238\H,-1.1639027398,1.4343344707,-1.1215055561\C,-2.4695437483
,0.3313932874,0.1396522757\H,-3.3142094662,1.0015652122,0.0136072988\H
,-2.6041798041,-0.5344459088,0.7810672181\S,0.0191311043,-0.5941664308
,-0.4303973983\S,1.6422365803,0.6598786578,-0.1135096264\H,1.523343150
6,0.8143382797,1.2191434118\\Version=EM64L-G09RevD.01\State=1-A\HF=-87
4.8465897\RMSD=8.025e-09\RMSF=7.558e-05\Dipole=-0.5316785,0.3664473,0.
4172311\Quadrupole=1.1650403,-1.1519953,-0.013045,-0.0294461,0.9230607
,-0.2706816\PG=C01 [X(C2H4S2)]\\@

1\1\GINC-ORC288\FOpt\RM062X\6-311G(d)\C2H4S2\SNIKOO\13-Jun-2017\0\\# o
pt freq 6-311g(d) m062x\\CH2=CH-SSH\\0,1\C,-1.2554724966,0.3775443519,
-0.3731891338\H,-1.0396687735,1.0839840623,-1.167911163\C,-2.413855185
,0.352519842,0.2723643149\H,-3.209997111,1.0377122302,0.0039925244\H,-
2.6063741633,-0.3533166818,1.0728508301\S,0.0072270016,-0.8277912572,-
0.0693195689\S,1.6920077134,0.3951565346,0.0205299104\H,1.5617060145,0
.807372918,1.2964852859\\Version=EM64L-G09RevD.01\State=1-A\HF=-874.92
39366\RMSD=2.164e-09\RMSF=6.158e-05\Dipole=-0.4974246,0.472622,0.35467
4\Quadrupole=1.0727369,-1.2183134,0.1455765,0.0274144,0.8417894,-0.125
4941\PG=C01 [X(C2H4S2)]\\@

```
1\1\GINC-ORC227\FOpt\RM062X\6-311+G(d)\C2H4S2\SNIKOO\13-Jun-2017\0\\#
 opt freq 6-311+g(d) m062x\\CH2=CH-SSH\\0,1\C,1.2545212192,-0.381746494
 9,-0.3526913195\H,1.0202650266,-1.1035332871,-1.1286991173\C,2.4291318
 67,-0.3452701016,0.2650758048\H,3.2190110188,-1.0345277944,-0.01118622
 97\H,2.63986378,0.3726629358,1.0503868145\S,-0.0017609049,0.8243952649
 ,-0.0339987322\S,-1.7024794254,-0.3771672891,0.0008264387\H,-1.5941255
 812,-0.8279952336,1.2660893407\\Version=EM64L-G09RevD.01\State=1-A\HF=
 -874.9274083\RMSD=9.061e-09\RMSF=4.689e-05\Dipole=0.4414445,-0.4570671
 ,0.3230694\Quadrupole=1.1432906,-1.113532,-0.0297586,0.1295767,-0.8592
 132,0.1675896\PG=C01 [X(C2H4S2)]\\@

1\1\GINC-ORC134\FOpt\RM062X\6-311+G(d,p)\C2H4S2\SNIKOO\19-Jul-2017\0\\
 # opt freq 6-311+g(d,p) geom=connectivity m062x\\CH2=CH-SSH\\0,1\C,1.2
 486441432,-0.401974646,-0.3436121745\H,1.000663377,-1.1508077495,-1.08
 85281199\C,2.4324275473,-0.3445849478,0.2546677898\H,3.2154829396,-1.0
 45257488,-0.0093815338\H,2.6532487347,0.4018297877,1.0091637897\S,-0.0
 004482287,0.8175966736,-0.0491763304\S,-1.7094278506,-0.3728902078,0.0
 153077508\H,-1.5949116626,-0.7926964223,1.2884988283\\Version=EM64L-G0
 9RevD.01\State=1-A\HF=-874.9355037\RMSD=5.845e-09\RMSF=4.035e-05\Dipol
 e=0.4281755,-0.4458272,0.3195223\Quadrupole=1.1370171,-1.0633896,-0.07
 36274,0.1326089,-0.8461866,0.1927756\PG=C01 [X(C2H4S2)]\\@

1\1\GINC-ORC188\FOpt\RM062X\6-311G(2d,p)\C2H4S2\SNIKOO\27-Jul-2017\0\\
 # opt freq m062x/6-311g(2d,p)\\CH2=CH-SSH\\0,1\C,1.2522510307,-0.40231
 54591,-0.3614456446\H,1.0222205177,-1.1391667226,-1.1218999476\C,2.419
 838702,-0.3492901848,0.2587356446\H,3.2120040547,-1.040914632,0.000886
 1534\H,2.6202736094,0.3869069901,1.0275081872\S,-0.0068320309,0.807264
 1886,-0.069722512\S,-1.7012086141,-0.3797387551,0.033086773\H,-1.57286
 82695,-0.7715304251,1.309791346\\Version=EM64L-G09RevD.01\State=1-A\HF
 =-874.9458752\RMSD=7.521e-09\RMSF=4.255e-05\Dipole=0.450321,-0.4348739
 ,0.2791401\Quadrupole=1.1781747,-1.111215,-0.0669597,0.1252527,-0.6773
 469,0.1572377\PG=C01 [X(C2H4S2)]\\@

1\1\GINC-ORC277\FOpt\RM062X\6-311G(df,p)\C2H4S2\SNIKOO\11-Sep-2018\0\\
 # opt freq=noraman m062x/6-311g(df,p)\\CH2=CH-SSH\\0,1\C,1.2526054824,
 -0.4055615016,-0.3502414136\H,1.019190049,-1.172886092,-1.0808142357\C
 ,2.4234439484,-0.329939495,0.2629630104\H,3.2136990395,-1.0340543776,0
 .0311989987\H,2.6309350833,0.436686419,1.0007618156\S,-0.0049354167,0.
 8162610353,-0.1096467191\S,-1.6934728649,-0.3762210336,0.0379087438\H,
 -1.5671883209,-0.7293169544,1.3301547998\\Version=EM64L-G09RevE.01\Sta
 te=1-A\HF=-874.9403788\RMSD=9.054e-09\RMSF=5.704e-05\Dipole=0.4781109,
 -0.4478795,0.3591417\Quadrupole=1.0247252,-1.1259421,0.101217,-0.04709
 48,-0.8415604,0.1967075\PG=C01 [X(C2H4S2)]\\@

1\1\GINC-ORC128\FOpt\RM062X\6-311+G(df,p)\C2H4S2\SNIKOO\19-Jul-2017\0\
 \# opt freq m062x/6-311+g(df,p) geom=connectivity\\CH2=CH-SSH\\0,1\C,1
 .2479351203,-0.4037835398,-0.3271569916\H,0.9874562186,-1.162494406,-1
 .0579958275\C,2.4398492222,-0.3398835396,0.2484182889\H,3.2184207289,-
 1.0443414398,-0.0188813905\H,2.675942489,0.4156150288,0.989274847\S,0.
 0006640685,0.8124308466,-0.0238460387\S,-1.7087154429,-0.3564391451,0.
 001742368\H,-1.6158734044,-0.8098888051,1.2653847445\\Version=EM64L-G0
 9RevD.01\State=1-A\HF=-874.9438898\RMSD=4.373e-09\RMSF=4.207e-05\Dipol
 e=0.4116393,-0.4516232,0.3108717\Quadrupole=1.1071933,-0.9858642,-0.12
 13291,0.1272826,-0.8776259,0.1799902\PG=C01 [X(C2H4S2)]\\@
```

1\1\GINC-ORC134\FOpt\RM062X\6-311+G(2df,p)\C2H4S2\SNIKOO\19-Jul-2017\0
\\# opt freq m062x/6-311+g(2df,p) geom=connectivity\\CH2=CH-SSH\\0,1\C
,1.2472664787,-0.4069141667,-0.3217051483\H,0.9851161848,-1.1673861926
,-1.0487336255\C,2.4419099254,-0.3386338264,0.2454412617\H,3.221327172
8,-1.0392905228,-0.0262550286\H,2.6792861362,0.4187050323,0.98294\S,-0
.0001759051,0.8002222333,-0.0073788753\S,-1.7063931277,-0.3475837645,-
0.0033112699\H,-1.6226578651,-0.8079037926,1.2559426858\\Version=EM64L
-G09RevD.01\State=1-A\HF=-874.9563574\RMSD=8.304e-09\RMSF=3.159e-05\Di
pole=0.3717017,-0.4224231,0.2533533\Quadrupole=1.1955798,-0.9443581,-0
.2512217,0.22451,-0.6933808,0.2054902\PG=C01 [X(C2H4S2)]\\@

1\1\GINC-ORC121\FOpt\RM062X\6-311++G(3df,3pd)\C2H4S2\SNIKOO\27-Jul-201
7\0\\# opt freq m062x/6-311++g(3df,3pd)\\CH2=CH-SSH\\0,1\C,1.246901594
1,-0.4093334763,-0.313038052\H,0.9806062317,-1.1728966382,-1.032641268
5\C,2.4470104538,-0.3366148711,0.2423385966\H,3.2234621112,-1.03542448
81,-0.0350532367\H,2.6898774472,0.4234579962,0.9723792332\S,0.00167473
57,0.7934365154,0.0080117855\S,-1.7066569725,-0.3391259093,-0.00896245
9\H,-1.637196601,-0.8122841286,1.243905401\\Version=EM64L-G09RevD.01\S
tate=1-A\HF=-874.961791\RMSD=6.608e-09\RMSF=3.875e-05\Dipole=0.35758,-
0.4061811,0.2214389\Quadrupole=1.2907815,-0.9281676,-0.362614,0.241445
7,-0.6042417,0.2232809\PG=C01 [X(C2H4S2)]\\@

CH_2CHSSH M08HX

1\1\GINC-ORC36\FOpt\RM08HX\6-31G(d)\C2H4S2\SNIKOO\25-Jul-2017\0\\# opt
freq 6-31g(d) m08hx\\CH2=CH-SS(-)\\0,1\C,1.2546077511,-0.4164271199,-
0.3300816985\H,1.0087828818,-1.2130438973,-1.0353883931\C,2.4453446799
,-0.3180513467,0.2553694678\H,3.2383911563,-1.0291720873,0.0240076007\
H,2.6723048203,0.4758957712,0.9680993372\S,-0.0027178496,0.8106071989,
-0.0925010195\S,-1.7017632648,-0.3701057044,0.0275824015\H,-1.61336617
5,-0.7204798144,1.327157304\\Version=EM64L-G16RevA.03\State=1-A\HF=-87
4.8582447\RMSD=9.964e-09\RMSF=1.188e-05\Dipole=0.4716808,-0.4494412,0.
3562555\Quadrupole=1.1937919,-1.072945,-0.1208469,0.0690683,-0.9865867
,0.1924681\PG=C01 [X(C2H4S2)]\\@

1\1\GINC-ORC35\FOpt\RM08HX\6-311G(d)\C2H4S2\SNIKOO\25-Jul-2017\0\\# op
t freq 6-311g(d) m08hx\\CH2=CH-SSH\\0,1\C,1.2510671127,-0.4099252378,-
0.3326349496\H,1.0002972825,-1.2026424312,-1.0375367681\C,2.4358680534
,-0.3206060233,0.2582457929\H,3.2231151054,-1.0381879123,0.0345007865\
H,2.663839405,0.4716163395,0.9701618755\S,-0.0002032772,0.8236043524,-
0.1070400262\S,-1.6927329456,-0.3806713294,0.0285354588\H,-1.579666736
2,-0.723964758,1.3300128301\\Version=EM64L-G16RevA.03\State=1-A\HF=-87
4.9421273\RMSD=7.164e-09\RMSF=2.823e-05\Dipole=0.4548243,-0.4495553,0.
3822002\Quadrupole=1.0905667,-1.0310257,-0.0595411,0.0896404,-0.899579
5,0.2039214\PG=C01 [X(C2H4S2)]\\@

1\1\GINC-ORC15\FOpt\RM08HX\6-311+G(d)\C2H4S2\SNIKOO\25-Jul-2017\0\\# o
pt freq 6-311+g(d) m08hx\\CH2=CH-SSH\\0,1\C,1.2521059045,-0.4126351714
,-0.3051818592\H,0.9819329405,-1.2192315062,-0.9874729784\C,2.45551787
57,-0.3122155077,0.2480749356\H,3.2355604307,-1.03385166,0.0118871076\
H,2.7064847424,0.4916121304,0.9393529962\S,0.0043559275,0.8192119828,-
0.0601435359\S,-1.7021140392,-0.3690230797,0.000549702\H,-1.6322597822
,-0.7446441882,1.2971786322\\Version=EM64L-G16RevA.03\State=1-A\HF=-87
4.9456466\RMSD=4.328e-09\RMSF=1.689e-05\Dipole=0.390579,-0.4298979,0.3
510327\Quadrupole=1.2039238,-0.9404235,-0.2635002,0.2099517,-0.9692002
,0.253876\PG=C01 [X(C2H4S2)]\\@

```
1\1\GINC-ORC1\FOpt\RM08HX\6-311+G(d,p)\C2H4S2\SNIKOO\25-Jul-2017\0\\#
 opt freq 6-311+g(d,p) m08hx\\CH2=CH-SSH\\0,1\C,1.2525762289,-0.4143390
 051,-0.2955582181\H,0.9751294839,-1.223856325,-0.9703175622\C,2.461215
 1318,-0.3101237004,0.2453909623\H,3.2369263566,-1.0332007452,0.0040174
 136\H,2.7155587142,0.4977033646,0.9292623106\S,0.0062988646,0.81767985
 98,-0.0438776598\S,-1.7054488157,-0.365448102,-0.0080773341\H,-1.64067
 19642,-0.7491923466,1.2834050877\\Version=EM64L-G16RevA.03\State=1-A\H
 F=-874.9538333\RMSD=4.172e-09\RMSF=1.592e-05\Dipole=0.3722974,-0.42764
 38,0.3357664\Quadrupole=1.212753,-0.9068352,-0.3059179,0.2509808,-0.94
 95194,0.233846\PG=C01 [X(C2H4S2)]\\@
```

```
1\1\GINC-ORC288\FOpt\RM08HX\6-311G(2d,p)\C2H4S2\SNIKOO\01-Aug-2017\0\\
 # opt freq 6-311g(2d,p) m08hx\\CH2=CH-SSH\\0,1\C,1.2420247649,-0.43809
 64533,-0.3269608106\H,0.978117552,-1.2534870036,-0.9972397516\C,2.4339
 023139,-0.3294166598,0.2378687747\H,3.2156943965,-1.0531126706,0.02319
 71011\H,2.6700095539,0.4861084161,0.9173080392\S,-0.0047128225,0.79863
 28069,-0.1131906699\S,-1.7063719727,-0.3701496441,0.0499364924\H,-1.59
 2044786,-0.6801107916,1.3526008246\\Version=EM64L-G16RevA.03\State=1-A
 \HF=-874.9639642\RMSD=3.959e-09\RMSF=3.447e-05\Dipole=0.4102606,-0.411
 7055,0.3091483\Quadrupole=1.1686729,-0.9646345,-0.2040383,0.1754119,-0
 .763233,0.1969422\PG=C01 [X(C2H4S2)]\\@
```

```
1\1\GINC-ORC36\FOpt\RM08HX\6-311+G(df,p)\C2H4S2\SNIKOO\25-Jul-2017\0\\
 # opt freq 6-311+g(df,p) m08hx\\CH2=CH-SSH\\0,1\C,1.2548550431,-0.4158
 772377,-0.2806132499\H,0.9690504208,-1.2302305918,-0.9462783157\C,2.46
 97016021,-0.3062657774,0.2382921044\H,3.2432370356,-1.0290413203,-0.01
 09037685\H,2.7356727636,0.5053637245,0.9133040955\S,0.0062741039,0.808
 2951632,-0.0163649444\S,-1.7017179216,-0.3604456696,-0.0219534466\H,-1
 .6754890476,-0.7525752909,1.2687625251\\Version=EM64L-G16RevA.03\State
 =1-A\HF=-874.9624935\RMSD=4.330e-09\RMSF=1.392e-05\Dipole=0.3542398,-0
 .4264743,0.3308582\Quadrupole=1.2071826,-0.8951119,-0.3120707,0.238642
 5,-1.0248711,0.2490605\PG=C01 [X(C2H4S2)]\\@
```

```
1\1\GINC-ORC35\FOpt\RM08HX\6-311+G(2df,p)\C2H4S2\SNIKOO\25-Jul-2017\0\
 \# opt freq 6-311+g(2df,p) m08hx\\CH2=CH-SSH\\0,1\C,1.2539827943,-0.41
 83778448,-0.2852297387\H,0.9744994942,-1.2310536889,-0.9538189897\C,2.
 465355364,-0.3076287739,0.2391116328\H,3.243176951,-1.024546639,-0.009
 7008039\H,2.7229603788,0.5021948943,0.9181580678\S,0.0050350237,0.7973
 844313,-0.0176880856\S,-1.6984337826,-0.3521726206,-0.0170735915\H,-1.
 6649922233,-0.7465767585,1.2704865087\\Version=EM64L-G16RevA.03\State=
 1-A\HF=-874.974811\RMSD=8.941e-09\RMSF=1.887e-05\Dipole=0.325668,-0.40
 03194,0.277657\Quadrupole=1.2266286,-0.8718662,-0.3547624,0.3074603,-0
 .8271664,0.2491965\PG=C01 [X(C2H4S2)]\\@
```

```
1\1\GINC-ORC288\FOpt\RM08HX\6-311++G(3df,3pd)\C2H4S2\SNIKOO\01-Aug-201
 7\0\\# opt freq 6-311++g(3df,3pd) m08hx\\CH2=CH-SSH\\0,1\C,1.241262080
 9,-0.4427471996,-0.2806791617\H,0.9497589867,-1.2757007782,-0.91451465
 32\C,2.4612663955,-0.3176091196,0.2205207381\H,3.2309514146,-1.0433492
 365,-0.0167669444\H,2.7308890569,0.5129788779,0.8647177056\S,0.0014994
 156,0.7829434521,-0.0350994821\S,-1.7069346374,-0.3482619575,0.0051305
 881\H,-1.6720737128,-0.7078860384,1.3002112097\\Version=EM64L-G16RevA.
 03\State=1-A\HF=-874.9814929\RMSD=9.353e-09\RMSF=2.054e-05\Dipole=0.32
 76674,-0.3834049,0.2549958\Quadrupole=1.3124322,-0.8562308,-0.4562013,
 0.272606,-0.7514752,0.2938615\PG=C01 [X(C2H4S2)]\\@
```

CH_2CHSSH QCISD


```
1\1\GINC-ORC295\FOpt\RQCISD-FC\6-311+G(2df,p)\C2H4S2\SNIKOO\19-Aug-201
 7\0\\# opt freq=noraman 6-311+g(2df,p) qcisd\\CH2=CH-SSH\\0,1\C,1.2559
 382836,-0.4216638217,-0.3259196913\H,1.0052563721,-1.2133034813,-1.025
 5440933\C,2.4532139456,-0.3317003649,0.2499663761\H,3.2343825947,-1.04
 51894182,0.0109044535\H,2.6910187156,0.4549464943,0.9583896119\S,0.000
 8913026,0.8014329467,-0.0733881672\S,-1.7045242681,-0.3667029091,0.022
 1659992\H,-1.6275949461,-0.7183664458,1.320233511\\Version=EM64L-G09Re
 vD.01\State=1-A\HF=-873.1488631\MP2=-873.8044276\MP3=-873.850573\MP4D=
 -873.868754\MP4DQ=-873.8481709\MP4SDQ=-873.8535906\QCISD=-873.8537534\
 RMSD=4.558e-09\RMSF=1.582e-05\Dipole=0.3762393,-0.3987053,0.2806508\PG
 =C01 [X(C2H4S2)]\\@
```


CH_2CHSS^- B3LYP


```
1\1\GINC-ORC289\FOpt\RB3LYP\6-31G(d)\C2H3S2(1-)\SNIKOO\13-Jun-2017\0\\
 # opt freq b3lyp/6-31g(d)\\CH2=CH-SS(-)\\-1,1\C,0.4974569502,0.4439225
 225,0.0362380332\H,0.5200234996,1.5324322329,-0.017482087\C,1.64304911
 89,-0.2617781203,0.097359325\H,2.6076936417,0.241339457,0.0942177569\H
 ,1.6554912188,-1.348736007,0.1518427512\S,-1.1434231785,-0.2028789919,
 0.0314654107\S,-2.4347266607,1.4634743868,-0.0821920199\\Version=EM64L
 -G09RevD.01\State=1-A\HF=-874.4148641\RMSD=7.978e-09\RMSF=2.654e-05\Di
 pole=1.6340449,-0.6380342,0.069604\Quadrupole=-4.4933703,2.1083483,2.3
 85022,4.5933848,-0.387953,0.120413\PG=C01 [X(C2H3S2)]\\@
```

```
1\1\GINC-ORC289\FOpt\RB3LYP\6-311G(d)\C2H3S2(1-)\SNIKOO\13-Jun-2017\0\
 \# opt freq b3lyp/6-311g(d)\\CH2=CH-SS(-)\\-1,1\C,-1.254348887,-0.4701
 13947,0.0002117028\H,-0.8626082044,-1.4847472127,0.0007738785\C,-2.577
 190843,-0.2433970252,-0.0002071719\H,-3.2816064461,-1.0702698561,0.000
 0845605\H,-2.9959695022,0.7596992443,-0.000824236\S,0.0245925356,0.741
 2090471,-0.000011422\S,1.8435583472,-0.3692372503,0.0000246882\\Versio
 n=EM64L-G09RevD.01\State=1-A\HF=-874.4953603\RMSD=4.904e-09\RMSF=2.954
 e-05\Dipole=-1.9287495,0.0429462,0.000104\Quadrupole=-6.8187658,4.6231
 744,2.1955914,1.136578,-0.0001055,-0.0012809\PG=C01 [X(C2H3S2)]\\@
1\1\GINC-ORC299\FOpt\RB3LYP\6-311+G(d)\C2H3S2(1-)\SNIKOO\13-Jun-2017\0
 \\# opt freq b3lyp/6-311+g(d)\\CH2=CH-SS(-)\\-1,1\C,-1.2423219565,-0.4
 785092031,0.0001524512\H,-0.8483007245,-1.492646263,0.0006871924\C,-2.
 570772293,-0.2581802883,-0.0001891199\H,-3.2699681509,-1.0892419615,0.
 0001164966\H,-2.995072077,0.7425361386,-0.0007581336\S,0.0238454305,0.
 7380086838,-0.0001597474\S,1.8502407714,-0.3460481064,0.0001628606\\Ve
 rsion=EM64L-G09RevD.01\State=1-A\HF=-874.5010573\RMSD=3.425e-09\RMSF=9
 .221e-05\Dipole=-1.8284939,0.0731011,-0.0000503\Quadrupole=-7.0113975,
 4.8506235,2.160774,1.0432085,-0.0006797,-0.001344\PG=C01 [X(C2H3S2)]\\
 @
```

```
1\1\GINC-ORC123\FOpt\RB3LYP\6-311+G(d,p)\C2H3S2(1-)\SNIKOO\19-Jul-2017
 \0\\# opt freq rb3lyp/6-311+g(d,p)\\CH2=CH-SS(-)\\-1,1\C,-1.2437781725
 ,-0.4768287152,0.0000740895\H,-0.8512000585,-1.4909252653,0.0002946635
 \C,-2.5716598115,-0.2535823997,-0.0000902103\H,-3.2702251596,-1.083619
 8439,0.0000322482\H,-2.9922558713,0.747488071,-0.0003295543\S,0.024530
 6481,0.7366570178,0.0000088212\S,1.8493624252,-0.3490388647,-0.0000050
 578\\Version=EM64L-G09RevD.01\State=1-A\HF=-874.5066926\RMSD=2.265e-09
 \RMSF=1.217e-04\Dipole=-1.8104051,0.0804362,0.0000491\Quadrupole=-7.09
 40822,4.8214449,2.2726373,1.0324821,-0.0000317,-0.0005693\PG=C01 [X(C2
 H3S2)]\\@
```

1\1\GINC-ORC177\FOpt\RB3LYP\6-311G(2d,p)\C2H3S2(1-)\SNIKOO\27-Jul-2017
\0\\# opt freq rb3lyp/6-311g(2d,p)\\CH2=CH-SS(-)\\-1,1\C,-1.2486799789
,-0.4781721843,0.0000697492\H,-0.8564746649,-1.4910445922,0.0002881031
\C,-2.5690070262,-0.254165492,-0.0000863463\H,-3.2686518018,-1.0825276
36,0.00003272\H,-2.9875598952,0.7469599857,-0.0003192596\S,0.029032017
5,0.7302254175,-0.0000099264\S,1.8461153495,-0.3411254987,0.00000996\\
Version=EM64L-G09RevD.01\State=1-A\HF=-874.5116392\RMSD=3.556e-09\RMSF
=3.434e-05\Dipole=-1.7941603,0.0219675,0.0000366\Quadrupole=-6.7523384
,4.5084533,2.2438851,0.8855036,-0.0000934,-0.0004377\PG=C01 [X(C2H3S2)
]\\@

1\1\GINC-ORC113\FOpt\RB3LYP\6-311+G(df,p)\C2H3S2(1-)\SNIKOO\19-Jul-201
7\0\\# opt freq rb3lyp/6-311+g(df,p) geom=connectivity\\CH2=CH-SS(-)\\
-1,1\C,-1.2441866624,-0.477531709,0.0000663496\H,-0.8538307079,-1.4924
686345,0.000274108\C,-2.5692571397,-0.2534163383,-0.0000802249\H,-3.26
86754146,-1.0822795029,0.0000347121\H,-2.9880509779,0.7480126445,-0.00
03073361\S,0.0264423649,0.7297319172,-0.0000365199\S,1.8423325377,-0.3
418983771,0.0000339112\\Version=EM64L-G09RevD.01\State=1-A\HF=-874.514
8081\RMSD=4.029e-09\RMSF=1.218e-04\Dipole=-1.7890331,0.0772669,0.00000
45\Quadrupole=-7.2080811,4.7836098,2.4244713,1.0779826,-0.0001657,-0.0
00499\PG=C01 [X(C2H3S2)]\\@

1\1\GINC-ORC108\FOpt\RB3LYP\6-311+G(2df,p)\C2H3S2(1-)\SNIKOO\19-Jul-20
17\0\\# opt freq rb3lyp/6-311+g(2df,p) geom=connectivity\\CH2=CH-SS(-)
\\-1,1\C,-1.2421661084,-0.4778397317,0.0000602342\H,-0.856540234,-1.49
40035647,0.0002724798\C,-2.5671237185,-0.2526176397,-0.0000820729\H,-3
.2667648038,-1.0804244803,0.0000414751\H,-2.9851585335,0.7486290293,-0
.0003080701\S,0.0302973157,0.7187894272,-0.0000359316\S,1.8322300827,-
0.3323830402,0.0000368854\\Version=EM64L-G09RevD.01\State=1-A\HF=-874.
5244317\RMSD=9.837e-09\RMSF=3.292e-05\Dipole=-1.6722776,0.0601184,0.00
00095\Quadrupole=-7.1339541,4.787952,2.3460021,0.8620935,-0.0001895,-0
.0005007\PG=C01 [X(C2H3S2)]\\@

1\1\GINC-ORC270\FOpt\RB3LYP\6-311++G(3df,3pd)\C2H3S2(1-)\SNIKOO\27-Jul
-2017\0\\# opt freq rb3lyp/6-311++g(3df,3pd)\\CH2=CH-SS(-)\\-1,1\C,-1.
2406977321,-0.4791797007,0.0000596675\H,-0.859259077,-1.4952869323,0.0
002728144\C,-2.5658132242,-0.2520756726,-0.0000824915\H,-3.2648090645,
-1.078119363,0.0000428495\H,-2.9812261686,0.7483517592,-0.0003089294\S
,0.0296125111,0.7130577072,-0.0000349896\S,1.8269667553,-0.3265977978,
0.0000360792\\Version=EM64L-G09RevD.01\State=1-A\HF=-874.5286907\RMSD=
3.269e-09\RMSF=4.318e-05\Dipole=-1.6061568,0.0568824,0.0000073\Quadrup
ole=-7.1119111,4.6556777,2.4562334,0.7838551,-0.0001842,-0.0004595\PG=
C01 [X(C2H3S2)]\\@

CH_2CHSS^- B3PW91

1\1\GINC-ORC81\FOpt\RB3PW91\6-31G(d)\C2H3S2(1-)\SNIKOO\20-Jul-2017\0\\
opt freq rb3pw91/6-31g(d)\\CH2=CH-SS(-)\\-1,1\C,1.2491543095,-0.4755
250272,-0.0001508791\H,0.8670394906,-1.4971725394,-0.0006919402\C,2.57
53664592,-0.2410203041,0.000184547\H,3.2848319879,-1.0658753304,-0.000
1381797\H,2.9885441861,0.7660270793,0.0007588161\S,-0.0326775727,0.720
7559784,0.0001636193\S,-1.8286858604,-0.3440468565,-0.0001779834\\Vers
ion=EM64L-G09RevD.01\State=1-A\HF=-874.2842519\RMSD=8.687e-09\RMSF=2.4
95e-05\Dipole=1.7573685,-0.0095117,0.0000189\Quadrupole=-6.6604843,4.3
962273,2.264257,-1.0908472,-0.0008088,0.0010879\PG=C01 [X(C2H3S2)]\\@

```
1\1\GINC-ORC69\FOpt\RB3PW91\6-311G(d)\C2H3S2(1-)\SNIKOO\20-Jul-2017\0\
 \# opt freq rb3pw91/6-311g(d) geom=connectivity\\CH2=CH-SS(-)\\-1,1\C,
 1.2495965953,-0.4695501699,-0.0001968272\H,0.8581704871,-1.4854955281,
 -0.0007713409\C,2.5726088914,-0.2418987324,0.0002136291\H,3.2763982152
 ,-1.0700336618,-0.0001013009\H,2.9903278167,0.7622242085,0.0008283273\
 S,-0.0234240519,0.7325138584,0.000004643\S,-1.8201049538,-0.3646169746
 ,-0.0000291304\\Version=EM64L-G09RevD.01\State=1-A\HF=-874.3578023\RMS
 D=4.712e-09\RMSF=1.158e-04\Dipole=1.9073028,0.0498704,-0.0001125\Quadr
 upole=-6.789849,4.6746533,2.1151956,-1.127844,-0.0001685,0.0013606\PG=
 C01 [X(C2H3S2)]\\@

1\1\GINC-ORC67\FOpt\RB3PW91\6-311+G(d)\C2H3S2(1-)\SNIKOO\20-Jul-2017\0
 \\# opt freq rb3pw91/6-311+g(d) geom=connectivity\\CH2=CH-SS(-)\\-1,1\
 C,1.2468715406,-0.4708920864,-0.0001477445\H,0.8606145883,-1.489330548
 8,-0.0006810432\C,2.5731714503,-0.240759762,0.0001805649\H,3.277402468
 2,-1.0683733843,-0.0001345814\H,2.9895084347,0.7640134179,0.000747078\
 S,-0.0235714209,0.7259844275,0.0001769961\S,-1.8204240611,-0.357499064
 ,-0.00019327\\Version=EM64L-G09RevD.01\State=1-A\HF=-874.3629952\RMSD=
 8.126e-09\RMSF=3.689e-05\Dipole=1.8023037,0.0831874,0.0000717\Quadrupo
 le=-6.9228188,4.8307888,2.09203,-1.1232544,-0.0008054,0.0013525\PG=C01
  [X(C2H3S2)]\\@

1\1\GINC-ORC64\FOpt\RB3PW91\6-311+G(d,p)\C2H3S2(1-)\SNIKOO\20-Jul-2017
 \0\\# opt freq rb3pw91/6-311+g(d,p) geom=connectivity\\CH2=CH-SS(-)\\-
 1,1\C,1.2477834974,-0.4710150569,-0.0001404334\H,0.859412346,-1.488214
 6172,-0.0006689707\C,2.574299791,-0.2403267584,0.0001769838\H,3.275673
 6333,-1.0688581515,-0.0001400373\H,2.9879329208,0.7643260884,0.0007368
 411\S,-0.0224070029,0.7251800764,0.0001977039\S,-1.8191221855,-0.35794
 85808,-0.0002140875\\Version=EM64L-G09RevD.01\State=1-A\HF=-874.368629
 5\RMSD=4.010e-09\RMSF=4.117e-05\Dipole=1.7828444,0.0883884,0.0000929\Q
 uadrupole=-7.0133467,4.8046826,2.2086641,-1.0916574,-0.0008999,0.00126
 12\PG=C01 [X(C2H3S2)]\\@

1\1\GINC-ORC280\FOpt\RB3PW91\6-311G(2d,p)\C2H3S2(1-)\SNIKOO\27-Jul-201
 7\0\\# opt freq rb3pw91/6-311g(2d,p)\\CH2=CH-SS(-)\\-1,1\C,1.251900270
 3,-0.4722166625,-0.0001806234\H,0.8642719356,-1.4882941427,-0.00072846
 45\C,2.5715984439,-0.2408451839,0.0001965796\H,3.2745949454,-1.0673541
 63,-0.0001168643\H,2.984319068,0.7634839668,0.0007926819\S,-0.02689479
 07,0.7185936947,0.0000879415\S,-1.8162168726,-0.3502245094,-0.00010325
 09\\Version=EM64L-G09RevD.01\State=1-A\HF=-874.3752015\RMSD=5.944e-09\
 RMSF=3.795e-05\Dipole=1.7573946,0.0348129,-0.000029\Quadrupole=-6.7003
 993,4.5367602,2.1636391,-0.9207048,-0.0004415,0.0011757\PG=C01 [X(C2H3
 S2)]\\@

1\1\GINC-ORC322\FOpt\RB3PW91\6-311G(df,p)\C2H3S2(1-)\SNIKOO\11-Sep-201
 8\0\\# opt freq=noraman b3pw91/6-311g(df,p)\\CH2=CH-SS(-)\\-1,1\C,-1.2
 376701639,-0.4667252268,0.0000920015\H,-0.8464405964,-1.4826550882,0.0
 003768961\C,-2.5591760612,-0.2400759616,-0.0001111911\H,-3.2602163741,
 -1.0689396993,0.0000449458\H,-2.9758565067,0.7632434387,-0.0004212923\
 S,0.0375339237,0.7290967524,-0.0000306807\S,1.8258707786,-0.3533222152
 ,0.0000293208\\Version=EM64L-G09RevE.01\State=1-A\HF=-874.3715356\RMSD
 =2.202e-09\RMSF=7.797e-05\Dipole=-1.8716475,0.0487651,0.0000309\Quadru
 pole=-6.9995703,4.5921128,2.4074575,1.1426273,-0.0001667,-0.0006101\PG
 =C01 [X(C2H3S2)]\\@

1\1\GINC-ORC62\FOpt\RB3PW91\6-311+G(df,p)\C2H3S2(1-)\SNIKOO\20-Jul-201
 7\0\\# opt freq rb3pw91/6-311+g(df,p) geom=connectivity\\CH2=CH-SS(-)\
```

```
\-1,1\C,1.2478328528,-0.4717934059,-0.0001389515\H,0.8623146162,-1.490
1897283,-0.0006645563\C,2.5719684853,-0.2402890619,0.0001745192\H,3.27
51655344,-1.0670914499,-0.0001433568\H,2.9848117455,0.7645651702,0.000
7351056\S,-0.0248468191,0.7185550272,0.0002059678\S,-1.813673415,-0.35
06135514,-0.000220728\\Version=EM64L-G09RevD.01\State=1-A\HF=-874.3768
441\RMSD=6.168e-09\RMSF=4.825e-05\Dipole=1.7583566,0.0840623,0.0001001
\Quadrupole=-7.1293725,4.7637584,2.3656141,-1.1366259,-0.0009667,0.001
1791\PG=C01 [X(C2H3S2)]\\@
```

```
1\1\GINC-ORC59\FOpt\RB3PW91\6-311+G(2df,p)\C2H3S2(1-)\SNIKOO\20-Jul-20
17\0\\# opt freq rb3pw91/6-311+g(2df,p) geom=connectivity\\CH2=CH-SS(-
)\\-1,1\C,1.2462405246,-0.4721668591,-0.0001281795\H,0.8655159865,-1.4
918065998,-0.00067221\C,2.5699942727,-0.2394730862,0.0001820602\H,3.27
32710629,-1.0654453076,-0.0001524663\H,2.9821713329,0.7652010539,0.000
7395129\S,-0.0285985497,0.7080536553,0.0001947422\S,-1.8050216299,-0.3
412198565,-0.0002154596\\Version=EM64L-G09RevD.01\State=1-A\HF=-874.38
74304\RMSD=4.937e-09\RMSF=1.704e-05\Dipole=1.6335421,0.0625217,0.00006
9\Quadrupole=-7.0432794,4.7637506,2.2795288,-0.9088599,-0.0009477,0.00
1226\PG=C01 [X(C2H3S2)]\\@
```

```
1\1\GINC-ORC262\FOpt\RB3PW91\6-311++G(3df,3pd)\C2H3S2(1-)\SNIKOO\27-Ju
l-2017\0\\# opt freq rb3pw91/6-311++g(3df,3pd)\\CH2=CH-SS(-)\\-1,1\C,1
.2449134538,-0.4733192734,-0.0001269365\H,0.8684017483,-1.4932665981,-
0.0006751581\C,2.5688235203,-0.2389084487,0.0001838421\H,3.2714998743,
-1.0634899697,-0.000155486\H,2.978704291,0.7650970523,0.0007425062\S,-
0.0279854284,0.7026532125,0.0001907597\S,-1.8007844593,-0.3356229749,-
0.0002115275\\Version=EM64L-G09RevD.01\State=1-A\HF=-874.3912885\RMSD=
6.773e-09\RMSF=2.115e-05\Dipole=1.5657197,0.0557004,0.0000648\Quadrupo
le=-6.9999593,4.6304846,2.3694748,-0.8186556,-0.000922,0.0011324\PG=C0
1 [X(C2H3S2)]\\@
```

CH_2CHSS^- wB97XD

```
1\1\GINC-ORC270\FOpt\RwB97XD\6-31G(d)\C2H3S2(1-)\SNIKOO\22-Jul-2017\0\
\# opt freq rwb97xd/6-31g(d)\\CH2=CH-SS(-)\\-1,1\C,-1.2427326541,-0.47
29456475,0.0000623251\H,-0.8615108868,-1.4939200947,0.0002639923\C,-2.
5633795168,-0.2383834412,-0.000072895\H,-3.2732774083,-1.0617216657,0.
0000322181\H,-2.973729505,0.7692322946,-0.00029001\S,0.0332129215,0.73
15038207,-0.000027843\S,1.8212360495,-0.3512092663,0.0000212124\\Versi
on=EM64L-G09RevD.01\State=1-A\HF=-874.345357\RMSD=6.423e-09\RMSF=1.193
e-04\Dipole=-1.8608918,-0.0261362,0.0000261\Quadrupole=-6.488819,4.293
6402,2.1951787,1.2339992,-0.0001229,-0.0004255\PG=C01 [X(C2H3S2)]\\@
```

```
1\1\GINC-ORC271\FOpt\RwB97XD\6-311G(d)\C2H3S2(1-)\SNIKOO\22-Jul-2017\0
\\# opt freq rwb97xd/6-311g(d)\\CH2=CH-SS(-)\\-1,1\C,-1.2434392146,-0.
4668481721,0.0000631043\H,-0.8525214544,-1.4822002943,0.0002676341\C,-
2.5613258183,-0.239247394,-0.0000742642\H,-3.2658469188,-1.0658872166,
0.000028\H,-2.9767210067,0.7654622214,-0.0002914001\S,0.0253211696,0.7
427246384,-0.0000176079\S,1.8143522432,-0.3714477828,0.0000135338\\Ver
sion=EM64L-G09RevD.01\State=1-A\HF=-874.4193011\RMSD=6.315e-09\RMSF=1.
128e-05\Dipole=-2.0118422,0.0371802,0.0000298\Quadrupole=-6.6150257,4.
5703773,2.0446484,1.2658831,-0.0000589,-0.000471\PG=C01 [X(C2H3S2)]\\@
```

```
1\1\GINC-ORC269\FOpt\RwB97XD\6-311+G(d)\C2H3S2(1-)\SNIKOO\22-Jul-2017\
0\\# opt freq rwb97xd/6-311+g(d)\\CH2=CH-SS(-)\\-1,1\C,-1.2409371558,-
0.469286421,0.0000484605\H,-0.8553549774,-1.4871314562,0.0002460785\C,
-2.5615651689,-0.2382472198,-0.0000678297\H,-3.267344653,-1.0637634409
```

,0.0000394279\H,-2.9745887466,0.7675480546,-0.0002727555\S,0.024667210
4,0.7370385655,-0.0000577923\S,1.8149424911,-0.3636020821,0.0000534106
\\Version=EM64L-G09RevD.01\State=1-A\HF=-874.42444\RMSD=7.280e-09\RMSF
=1.225e-05\Dipole=-1.9292368,0.0676576,-0.0000155\Quadrupole=-6.649817
4,4.679387,1.9704304,1.2659362,-0.0002384,-0.0004841\PG=C01 [X(C2H3S2)
]\\@

1\1\GINC-ORC268\FOpt\RwB97XD\6-311+G(d,p)\C2H3S2(1-)\SNIKOO\22-Jul-201
7\0\\# opt freq rwb97xd/6-311+g(d,p)\\CH2=CH-SS(-)\\-1,1\C,-1.24185267
86,-0.469299629,0.0000454854\H,-0.854267115,-1.4860254371,0.000241433\
C,-2.5626317109,-0.2378861528,-0.0000667398\H,-3.2654936604,-1.0644177
37,0.0000416748\H,-2.9727395943,0.7679217482,-0.0002685414\S,0.0233853
902,0.7364232935,-0.0000659661\S,1.8134183691,-0.3641600858,0.00006165
41\\Version=EM64L-G09RevD.01\State=1-A\HF=-874.4298016\RMSD=9.076e-09\
RMSF=1.822e-05\Dipole=-1.9106448,0.072978,-0.0000236\Quadrupole=-6.737
6747,4.65488,2.0827947,1.2377238,-0.0002791,-0.0004509\PG=C01 [X(C2H3S
2)]\\@

1\1\GINC-ORC273\FOpt\RwB97XD\6-311G(2d,p)\C2H3S2(1-)\SNIKOO\27-Jul-201
7\0\\# opt freq rwb97xd/6-311g(2d,p)\\CH2=CH-SS(-)\\-1,1\C,-1.24606387
22,-0.4707799918,0.0000673085\H,-0.8596515404,-1.4864128745,0.00027140
7\C,-2.560275087,-0.2383377665,-0.0000752769\H,-3.2650113752,-1.062517
6957,0.0000278468\H,-2.969618279,0.767093843,-0.0002960937\S,0.0279963
337,0.7290194194,-0.0000122756\S,1.8124428201,-0.3555089338,0.00000608
38\\Version=EM64L-G09RevD.01\State=1-A\HF=-874.4362088\RMSD=9.986e-09\
RMSF=3.654e-05\Dipole=-1.8653395,0.024095,0.000034\Quadrupole=-6.52723
71,4.4531716,2.0740655,1.042653,-0.0000392,-0.0004337\PG=C01 [X(C2H3S2
)]\\@

1\1\GINC-ORC323\FOpt\RwB97XD\6-311G(df,p)\C2H3S2(1-)\SNIKOO\11-Sep-201
8\0\\# opt=calcfc freq=noraman wb97xd/6-311g(df,p) nosymm\\CH2=CH-SS(-
)1\\-1,1\C,-1.2447948509,-0.4671448771,0.007651961\H,-0.8545678366,-1.
4819776317,0.0382154922\C,-2.5603100089,-0.238523641,-0.0009762642\H,-
3.2639214344,-1.0640123474,0.0228964446\H,-2.9726418017,0.7657186071,-
0.032466648\S,0.0268150861,0.7357329172,-0.0237736526\S,1.8092388464,-
0.367237027,-0.0115583331\\Version=EM64L-G09RevE.01\HF=-874.4327785\RM
SD=4.240e-09\RMSF=1.419e-05\Dipole=-1.9812491,0.0357337,0.0102723\Quad
rupole=-6.7561678,4.4514935,2.3046743,1.3052929,0.0547481,-0.0743562\P
G=C01 [X(C2H3S2)]\\@

1\1\GINC-ORC267\FOpt\RwB97XD\6-311+G(df,p)\C2H3S2(1-)\SNIKOO\22-Jul-20
17\0\\# opt freq rwb97xd/6-311+g(df,p)\\CH2=CH-SS(-)\\-1,1\C,-1.242105
1123,-0.4702946082,0.0000595139\H,-0.8580334206,-1.4884388906,0.000257
9833\C,-2.5603537218,-0.2378239683,-0.0000710831\H,-3.2652619011,-1.06
24630812,0.0000343765\H,-2.9699202866,0.7680910934,-0.0002862058\S,0.0
260050392,0.7297445876,-0.00003484\S,1.8094884031,-0.3562591325,0.0000
292552\\Version=EM64L-G09RevD.01\State=1-A\HF=-874.4380164\RMSD=7.333e
-09\RMSF=8.595e-05\Dipole=-1.8885345,0.0657333,0.0000055\Quadrupole=-6
.8463492,4.606225,2.2401241,1.2818653,-0.0001428,-0.0004699\PG=C01 [X(
C2H3S2)]\\@

1\1\GINC-ORC266\FOpt\RwB97XD\6-311+G(2df,p)\C2H3S2(1-)\SNIKOO\22-Jul-2
017\0\\# opt freq rwb97xd/6-311+g(2df,p)\\CH2=CH-SS(-)\\-1,1\C,-1.2409
07108,-0.4707729287,0.0000564294\H,-0.860008835,-1.4893739609,0.000254
2221\C,-2.5586575183,-0.2372491743,-0.0000702238\H,-3.2639309126,-1.06
06978358,0.0000366321\H,-2.9670560393,0.7686848331,-0.0002820863\S,0.0
287425662,0.7197206456,-0.0000431414\S,1.8016368469,-0.3477555791,0.00

00371679\\Version=EM64L-G09RevD.01\State=1-A\HF=-874.4483295\RMSD=6.61
9e-09\RMSF=1.851e-05\Dipole=-1.7685627,0.0503522,0.0000007\Quadrupole=
-6.7616871,4.6165431,2.145144,1.0491713,-0.00016,-0.0004615\PG=C01 [X(
C2H3S2)]\\@

1\1\GINC-ORC23\FOpt\RwB97XD\6-311++G(3df,3pd)\C2H3S2(1-)\SNIKOO\21-Sep
-2017\0\\# opt=calcfc freq=noraman wb97xd/6-311++g(3df,3pd) nosymm geo
m=connectivity\\CH2=CH-SS(-)\\-1,1\C,1.2274094704,-0.4343254835,-0.127
5652661\H,0.9128054674,-1.2709701738,-0.74453446\C,2.5178666642,-0.279
6450389,0.193657274\H,3.2572524735,-0.9884163706,-0.1564268104\H,2.864
3332797,0.5438558385,0.807222824\S,-0.0920872481,0.5914802368,0.341041
4973\S,-1.782985107,-0.2290040086,-0.5056060587\\Version=EM64L-G09RevD
.01\HF=-874.4523459\RMSD=6.518e-09\RMSF=1.237e-05\Dipole=1.681156,-0.0
185808,0.2768833\Quadrupole=-6.4170179,3.8847251,2.5322927,-0.6240568,
-2.0439947,0.9959716\PG=C01 [X(C2H3S2)]\\@

CH_2CHSS^- M062X

1\1\GINC-ORC292\FOpt\RM062X\6-31G(d)\C2H3S2(1-)\SNIKOO\07-Sep-2017\0\\
opt=calcfc freq=noraman m062x/6-31g(d) nosymm\\CH2=CH-SS(-) new\\-1,
1\C,-1.2422814191,-0.4759911136,0.0378995513\H,-0.857875663,-1.4955166
812,0.0617477683\C,-2.5605531318,-0.2397016447,-0.0177355749\H,-3.2731
723192,-1.0593365327,-0.023408491\H,-2.9622312622,0.7697246958,-0.0551
608403\S,0.0280637572,0.7316272342,0.1039440692\S,1.8078690381,-0.3482
499578,-0.1072974827\\Version=EM64L-G09RevD.01\HF=-874.2997278\RMSD=9.
422e-09\RMSF=3.462e-05\Dipole=-1.8267093,-0.0250081,0.096031\Quadrupol
e=-6.2745884,4.1742464,2.100342,1.185061,0.4124682,-0.1352785\PG=C01 [
X(C2H3S2)]\\@

1\1\GINC-ORC37\FOpt\RM062X\6-311G(d)\C2H3S2(1-)\SNIKOO\21-Sep-2017\0\\
opt=calcfc nosymm freq=noraman m062x/6-311g(d)\\CH2=CH-SS(-)\\-1,1\C
,1.2160931684,-0.4334521187,-0.1351232572\H,0.8828971753,-1.2303490543
,-0.7954730589\C,2.5053426352,-0.3060434056,0.1973913602\H,3.246222939
4,-0.9999115479,-0.1849620994\H,2.8543007345,0.4851424889,0.8539563027
\S,-0.1018242454,0.5911137258,0.3976129673\S,-1.7925584073,-0.20147808
81,-0.5807032147\\Version=EM64L-G09RevD.01\HF=-874.3858084\RMSD=6.288e
-09\RMSF=2.449e-05\Dipole=1.9525971,-0.0742353,0.3485259\Quadrupole=-6
.1321064,3.7647383,2.3673681,-0.6698018,-2.4033015,1.1643519\PG=C01 [X
(C2H3S2)]\\@

1\1\GINC-ORC11\FOpt\RM062X\6-311+G(d)\C2H3S2(1-)\SNIKOO\12-Sep-2017\0\
\# opt=calcfc freq=noraman m062x/6-311+g(d) nosymm\\CH2=CH-SS(-) new\\
-1,1\C,-1.2401389034,-0.4728710796,0.0372885482\H,-0.8528298032,-1.489
1117942,0.0586614368\C,-2.5592709591,-0.2392178833,-0.0163629578\H,-3.
2667266041,-1.0612291418,-0.0235806223\H,-2.9627256766,0.7682004168,-0
.052548911\S,0.0196566588,0.7363023681,0.099726025\S,1.8018552876,-0.3
59517886,-0.1031935189\\Version=EM64L-G09RevD.01\HF=-874.3909975\RMSD=
9.804e-09\RMSF=3.085e-05\Dipole=-1.889593,0.0624578,0.0959878\Quadrupo
le=-6.3856491,4.5512094,1.8344397,1.2354716,0.4307142,-0.1332391\PG=C0
1 [X(C2H3S2)]\\@

1\1\GINC-ORC83\FOpt\RM062X\6-311+G(d,p)\C2H3S2(1-)\SNIKOO\12-Sep-2017\
0\\# opt=calcfc freq=noraman m062x/6-311+g(d,p) nosymm\\CH2=CH-SS(-) n
ew\\-1,1\C,-1.2410796661,-0.4730119143,0.035471966\H,-0.8520608623,-1.
4883070501,0.0556419362\C,-2.5604412806,-0.2388689702,-0.0155274455\H,
-3.2651276369,-1.0619814031,-0.0221853628\H,-2.961008329,0.7687547703,
-0.0499536089\S,0.0182717614,0.7360424928,0.0944631608\S,1.8012660134,

```
-0.3600729254,-0.0979206457\\Version=EM64L-G09RevD.01\HF=-874.3953062\
RMSD=8.033e-09\RMSF=3.463e-05\Dipole=-1.8770456,0.0647393,0.0907353\Qu
adrupole=-6.4639546,4.5392386,1.924716,1.2154593,0.4059575,-0.1252338\
PG=C01 [X(C2H3S2)]\\@

1\1\GINC-ORC38\FOpt\RM062X\6-311G(2d,p)\C2H3S2(1-)\SNIKOO\07-Sep-2017\
0\\# opt=calcfc nosymm freq=noraman m062x/6-311g(2d,p)\\CH2=CH-SS(-) n
ew\\-1,1\C,-1.2459659263,-0.4768143779,-0.0327020338\H,-0.8582065293,-
1.4911678366,-0.0258607937\C,-2.5581110335,-0.238583409,0.0175015046\H
,-3.2657701114,-1.0576556008,0.0488295947\H,-2.9556156412,0.7699027156
,0.0247623717\S,0.0226640988,0.7253674195,-0.1312419757\S,1.800824143,
-0.3484929108,0.0987003322\\Version=EM64L-G09RevD.01\HF=-874.4016449\R
MSD=6.259e-09\RMSF=4.923e-05\Dipole=-1.8550233,0.0097185,-0.090043\Qua
drupole=-6.2415278,4.3477554,1.8937724,1.0140969,-0.366246,0.0041183\P
G=C01 [X(C2H3S2)]\\@

1\1\GINC-ORC319\FOpt\RM062X\6-311G(df,p)\C2H3S2(1-)\SNIKOO\11-Sep-2018
\0\\# opt=calcfc freq=noraman m062x/6-311g(df,p) nosymm\\CH2=CH-SS(-)1
\\-1,1\C,-1.2440870029,-0.4718900238,-0.0322446627\H,-0.8528563353,-1.
486395966,-0.0258153753\C,-2.5579788042,-0.238999937,0.0166970346\H,-3
.2637397669,-1.0609892224,0.0485776941\H,-2.9609119003,0.7683764084,0.
0242698783\S,0.0229615671,0.7316707604,-0.12973675\S,1.7964302425,-0.3
592160196,0.098240181\\Version=EM64L-G09RevE.01\HF=-874.3987379\RMSD=6
.238e-09\RMSF=3.128e-05\Dipole=-1.9591814,0.0229562,-0.0942914\Quadrup
ole=-6.5444929,4.3713823,2.1731106,1.2672944,-0.4186048,0.0560614\PG=C
01 [X(C2H3S2)]\\@

1\1\GINC-ORC199\FOpt\RM062X\6-311+G(df,p)\C2H3S2(1-)\SNIKOO\22-Aug-201
7\0\\# opt freq m062x/6-311+g(df,p)\\CH2=CH-SS(-) new\\-1,1\C,-1.22476
90836,-0.471947357,0.0001638629\H,-0.8376598104,-1.4887143672,0.000707
6874\C,-2.5433522123,-0.2391006252,-0.0001904863\H,-3.2486684162,-1.06
18438259,0.0001083936\H,-2.9462152094,0.7684880682,-0.0007757238\S,0.0
372679768,0.7320665427,-0.0001414023\S,1.819897755,-0.3518944356,0.000
1346685\\Version=EM64L-G09RevD.01\State=1-A\HF=-874.4040386\RMSD=3.518
e-09\RMSF=7.108e-05\Dipole=-1.847826,0.0588451,-0.0000277\Quadrupole=-
6.7209638,4.5436972,2.1772666,1.2603299,-0.000587,-0.0012729\PG=C01 [X
(C2H3S2)]\\@

1\1\GINC-ORC136\FOpt\RM062X\6-311+G(2df,p)\C2H3S2(1-)\SNIKOO\19-Jul-20
17\0\\# opt freq m062x/6-311+g(2df,p) geom=connectivity\\CH2=CH-SS(-)\
\-1,1\C,-1.2237681329,-0.4793544545,0.0002291636\H,-0.8327628313,-1.49
33864219,0.0008072364\C,-2.5426806578,-0.2522704231,-0.0002236487\H,-3
.2437529434,-1.0773036946,0.0000697449\H,-2.949422155,0.7527919838,-0.
0008723129\S,0.0337168173,0.7220613031,0.0000505133\S,1.811782903,-0.3
359232928,-0.0000526966\\Version=EM64L-G09RevD.01\State=1-A\HF=-874.41
41131\RMSD=3.710e-09\RMSF=9.581e-05\Dipole=-1.7321391,0.0361196,0.0001
509\Quadrupole=-6.6340865,4.565116,2.0689705,0.9706299,0.0002503,-0.00
15067\PG=C01 [X(C2H3S2)]\\@

1\1\GINC-ORC252\FOpt\RM062X\6-311++G(3df,3pd)\C2H3S2(1-)\SNIKOO\28-Aug
-2017\0\\# opt=calcfc freq=noraman m062x/6-311++g(3df,3pd)\\CH2=CH-SS(
-)\\-1,1\C,1.24566815,-0.4766626198,0.0005069994\H,0.8649324619,-1.492
9438074,-0.0005446055\C,2.5640032384,-0.2416415258,-0.0001201746\H,3.2
678149727,-1.0620882994,-0.0012010896\H,2.9639890709,0.7642643986,0.00
05377337\S,-0.0165276887,0.7142607582,0.0032735468\S,-1.7863072051,-0.
3420459044,-0.0025044102\\Version=EM64L-G09RevD.01\State=1-A\HF=-874.4
1826\RMSD=4.644e-09\RMSF=6.165e-06\Dipole=1.6631903,0.0416196,0.002005
```

9\Quadrupole=-6.5717663,4.4349984,2.136768,-0.9241858,-0.0107538,0.000
6207\PG=C01 [X(C2H3S2)]\\@

CH_2CHSS^- M08HX

1\1\GINC-ORC342\FOpt\RM08HX\6-31G(d)\C2H3S2(1-)\SNIKOO\25-Jul-2017\0\\
opt freq 6-31g(d) m08hx\\CH2=CH-SS(-)\\-1,1\C,-1.2399493137,-0.46901
81569,0.0000537831\H,-0.8494610355,-1.4919630123,0.0002538799\C,-2.561
5071499,-0.2396976765,-0.0000724068\H,-3.2725918694,-1.066370042,0.000
0369803\H,-2.9728352668,0.7713521117,-0.0002790291\S,0.0304842304,0.74
178671,-0.000045139\S,1.8056794049,-0.3635339341,0.0000409316\\Version
=EM64L-G16RevA.03\State=1-A\HF=-874.3097558\RMSD=6.421e-09\RMSF=1.558e
-05\Dipole=-1.8358253,-0.0087532,0.0000086\Quadrupole=-6.2686975,4.193
0608,2.0756366,1.2386519,-0.000203,-0.0004036\PG=C01 [X(C2H3S2)]\\@

1\1\GINC-ORC36\FOpt\RM08HX\6-311G(d)\C2H3S2(1-)\SNIKOO\25-Jul-2017\0\\
opt freq 6-311g(d) m08hx\\CH2=CH-SS(-)\\-1,1\C,-1.2402008522,-0.4628
48911,0.0000682124\H,-0.8398370835,-1.4794849416,0.000274446\C,-2.5597
727714,-0.2403812517,-0.0000787977\H,-3.2643233803,-1.071073028,0.0000
244861\H,-2.9752271438,0.7678777913,-0.0002965325\S,0.0230339578,0.752
2900195,-0.0000044477\S,1.7961462734,-0.3838236784,0.0000016334\\Versi
on=EM64L-G16RevA.03\State=1-A\HF=-874.4013342\RMSD=3.267e-09\RMSF=2.92
2e-05\Dipole=-1.962786,0.0550339,0.0000404\Quadrupole=-6.4193196,4.473
2158,1.9461038,1.2767271,-0.0000114,-0.0004809\PG=C01 [X(C2H3S2)]\\@

1\1\GINC-ORC23\FOpt\RM08HX\6-311+G(d)\C2H3S2(1-)\SNIKOO\25-Jul-2017\0\
\# opt freq 6-311+g(d) m08hx\\CH2=CH-SS(-)\\-1,1\C,-1.2375023471,-0.46
59825535,0.0000469555\H,-0.8432587404,-1.4855576579,0.0002417357\C,-2.
5601571425,-0.2391931416,-0.000069525\H,-3.2667849332,-1.068162848,0.0
000414526\H,-2.9731528332,0.7702631046,-0.0002696493\S,0.0220719173,0.
7454354741,-0.0000617411\S,1.7986030791,-0.3742463777,0.0000597716\\Ve
rsion=EM64L-G16RevA.03\State=1-A\HF=-874.406893\RMSD=5.860e-09\RMSF=1.
642e-05\Dipole=-1.8443669,0.0908372,-0.0000194\Quadrupole=-6.485314,4.
584956,1.900358,1.2568016,-0.0002649,-0.0004757\PG=C01 [X(C2H3S2)]\\@

1\1\GINC-ORC158\FOpt\RM08HX\6-311+G(d,p)\C2H3S2(1-)\SNIKOO\25-Jul-2017
\0\\# opt freq 6-311+g(d,p) m08hx\\CH2=CH-SS(-)\\-1,1\C,-1.2385373203,
-0.4662463756,0.0000427143\H,-0.8428023538,-1.4845874581,0.0002349964\
C,-2.5612881978,-0.2388450552,-0.0000677732\H,-3.2647691681,-1.0686715
229,0.0000447933\H,-2.9709039342,0.7705445433,-0.0002641066\S,0.020759
1767,0.7447438653,-0.0000727805\S,1.7973607975,-0.3743819968,0.0000711
563\\Version=EM64L-G16RevA.03\State=1-A\HF=-874.4115376\RMSD=6.068e-09
\RMSF=2.193e-05\Dipole=-1.8303932,0.0942949,-0.0000303\Quadrupole=-6.5
651409,4.5612729,2.003868,1.2337008,-0.0003186,-0.00044\PG=C01 [X(C2H3
S2)]\\@

1\1\GINC-ORC291\FOpt\RM08HX\6-311G(2d,p)\C2H3S2(1-)\SNIKOO\01-Aug-2017
\0\\# opt freq 6-311g(2d,p) m08hx\\CH2=CH-SS(-)\\-1,1\C,1.2225913118,-
0.4765698676,-0.000034387\H,0.8190577194,-1.4896751861,-0.0002185924\C
,2.5398117379,-0.2605161229,0.0000578066\H,3.2366802195,-1.0944270965,
-0.0000541597\H,2.9570818798,0.7443559881,0.0002407063\S,-0.0340125495
,0.7409028665,0.0001056796\S,-1.8176283189,-0.3489275816,-0.0001100534
\\Version=EM64L-G16RevA.03\State=1-A\HF=-874.4175343\RMSD=8.815e-09\RM
SF=1.563e-04\Dipole=1.8304645,0.0212232,0.0000578\Quadrupole=-6.352962
5,4.3746638,1.9782988,-0.9602713,-0.000426,0.0003642\PG=C01 [X(C2H3S2)
]\\@

1\1\GINC-ORC258\FOpt\RM08HX\6-311+G(df,p)\C2H3S2(1-)\SNIKOO\25-Jul-201
7\0\\# opt freq 6-311+g(df,p) m08hx\\CH2=CH-SS(-)\\-1,1\C,-1.238697696
1,-0.4673654807,0.000062944\H,-0.84746611,-1.487874911,0.0002618673\C,
-2.5587944628,-0.238428086,-0.000074369\H,-3.2647113154,-1.0664874789,
0.000032196\H,-2.968779954,0.7709369636,-0.0002885858\S,0.0241010707,0
.7367518393,-0.0000282047\S,1.7941674676,-0.3649768463,0.0000231522\\V
ersion=EM64L-G16RevA.03\State=1-A\HF=-874.420542\RMSD=5.173e-09\RMSF=8
.048e-05\Dipole=-1.8070742,0.0865215,0.0000118\Quadrupole=-6.6865071,4
.5121299,2.1743772,1.2706386,-0.0001102,-0.0004793\PG=C01 [X(C2H3S2)]\
\@

1\1\GINC-ORC36\FOpt\RM08HX\6-311+G(2df,p)\C2H3S2(1-)\SNIKOO\25-Jul-201
7\0\\# opt freq 6-311+g(2df,p) m08hx\\CH2=CH-SS(-)\\-1,1\C,-1.23819924
36,-0.4679411665,0.0000556682\H,-0.8497870742,-1.4883169591,0.00025210
3\C,-2.5576909637,-0.2380308367,-0.0000717483\H,-3.2635135097,-1.06451
32337,0.0000373062\H,-2.9654275114,0.7712038803,-0.0002794725\S,0.0263
404768,0.7265290691,-0.0000462347\S,1.7880968259,-0.3563747534,0.00004
13781\\Version=EM64L-G16RevA.03\State=1-A\HF=-874.4306121\RMSD=5.335e-
09\RMSF=1.413e-05\Dipole=-1.6890059,0.0699748,-0.0000021\Quadrupole=-6
.6158439,4.5253022,2.0905418,1.0364141,-0.0001716,-0.0004549\PG=C01 [X
(C2H3S2)]\\@

1\1\GINC-ORC290\FOpt\RM08HX\6-311++G(3df,3pd)\C2H3S2(1-)\SNIKOO\01-Aug
-2017\0\\# opt freq 6-311++g(3df,3pd) m08hx\\CH2=CH-SS(-)\\-1,1\C,1.21
67544196,-0.4781661947,-0.0000423602\H,0.821357147,-1.4933416782,-0.00
02272429\C,2.538425879,-0.2587048023,0.0000589216\H,3.2348437526,-1.08
96656691,-0.0000478104\H,2.9518915546,0.7452306933,0.0002474192\S,-0.0
362651789,0.7237764154,0.0000918307\S,-1.803425574,-0.3339857642,-0.00
00937581\\Version=EM64L-G16RevA.03\State=1-A\HF=-874.4356384\RMSD=4.20
5e-09\RMSF=4.320e-05\Dipole=1.6251406,0.0475181,0.000047\Quadrupole=-6
.5158879,4.3892064,2.1266814,-0.8515719,-0.0003454,0.0003664\PG=C01 [X
(C2H3S2)]\\@

CH_2CHSS^- QCISD

1\1\GINC-ORC52\FOpt\RQCISD-FC\6-311+G(2df,p)\C2H3S2(1-)\SNIKOO\21-Aug-
2017\0\\# opt freq=noraman 6-311+g(2df,p) qcisd\\CH2=CH-SS(-)\\-1,1\C,
1.224705,-0.472874,-0.000101\H,0.836392,-1.489505,-0.000329\C,2.549587
,-0.249418,0.000094\H,3.248904,-1.079563,-0.00001\H,2.966151,0.754125,
0.000346\S,-0.039046,0.737457,-0.00005\S,-1.817029,-0.353164,0.000052\
\Version=EM64L-G09RevD.01\State=1-A\HF=-872.5971704\MP2=-873.2613726\M
P3=-873.3013769\MP4D=-873.3192005\MP4DQ=-873.2977294\MP4SDQ=-873.30390
01\QCISD=-873.3039953\RMSD=5.031e-09\RMSF=2.554e-05\Dipole=1.8143534,0
.04151,-0.0000835\PG=C01 [X(C2H3S2)]\\@

$CH_2CHSS^{\bullet}$ B3LYP

1\1\GINC-ORC90\FOpt\UB3LYP\6-31G(d)\C2H3S2(2)\SNIKOO\18-Aug-2017\0\\#
opt freq ub3lyp/6-31g(d)\\CH2=CH-SS\\0,2\C,1.7569529856,0.3874000892,0
.4778606574\H,1.5531618472,0.2722782795,1.5393868306\C,2.7970547678,1.
0741010909,-0.0052367951\H,3.4987154293,1.5573821701,0.6680183536\H,2.
9838724579,1.1781191533,-1.070933236\S,0.5895649283,-0.4152469064,-0.5
977206407\S,-0.759235416,-1.2919798666,0.5808408302\\Version=EM64L-G09
RevD.01\State=2-A\HF=-874.3554983\S2=0.756245\S2-1=0.\S2A=0.750025\RMS
D=4.107e-09\RMSF=7.418e-05\Dipole=0.9221807,0.6172265,0.0293787\Quadru
pole=0.4259816,-0.6529681,0.2269865,1.315036,0.0785138,0.0869846\PG=C0
1 [X(C2H3S2)]\\@

```
1\1\GINC-ORC90\FOpt\UB3LYP\6-311G(2d,p)\C2H3S2(2)\SNIKOO\18-Aug-2017\0
 \\# opt freq ub3lyp/6-311g(2d,p)\\CH2=CH-SS\\0,2\C,1.7600726915,0.3893
 741553,0.4785746737\H,1.5552668998,0.2733797232,1.5359606531\C,2.79377
 66843,1.0716771296,-0.0036552317\H,3.494886985,1.5541936005,0.66564232
 93\H,2.975708403,1.1725447053,-1.067252559\S,0.5947969457,-0.411076494
 4,-0.5917677971\S,-0.7544216092,-1.2880388095,0.5747139317\\Version=EM
 64L-G09RevD.01\State=2-A\HF=-874.4422239\S2=0.757494\S2-1=0.\S2A=0.750
 041\RMSD=4.111e-09\RMSF=7.163e-05\Dipole=0.8918791,0.5963494,0.0145091
 \Quadrupole=0.4165696,-0.7521066,0.335537,1.4127262,-0.0882782,-0.0190
 587\PG=C01 [X(C2H3S2)]\\@
```

```
1\1\GINC-ORC84\FOpt\UB3LYP\6-311+G(2df,p)\C2H3S2(2)\SNIKOO\18-Aug-2017
 \0\\# opt freq ub3lyp/6-311+g(2df,p)\\CH2=CH-SS\\0,2\C,1.7572594147,0.
 3873981861,0.4774090874\H,1.5556395555,0.272563356,1.5361991385\C,2.79
 19486898,1.0711391201,-0.0040007589\H,3.4930803283,1.5532841625,0.6653
 13397\H,2.9742115875,1.1730539601,-1.0675274726\S,0.5933426333,-0.4115
 914673,-0.5861582224\S,-0.7453952091,-1.2837933076,0.5709808309\\Versi
 on=EM64L-G09RevD.01\State=2-A\HF=-874.4540266\S2=0.757808\S2-1=0.\S2A=
 0.750046\RMSD=3.238e-09\RMSF=6.018e-05\Dipole=0.8359428,0.559368,0.013
 9496\Quadrupole=0.3983323,-0.7428222,0.3444899,1.3837036,-0.0411431,0.
 0102898\PG=C01 [X(C2H3S2)]\\@
```

```
1\1\GINC-ORC77\FOpt\UB3LYP\6-311++G(3df,3pd)\C2H3S2(2)\SNIKOO\18-Aug-2
 017\0\\# opt freq=noraman ub3lyp/6-311++g(3df,3pd)\\CH2=CH-SS\\0,2\C,1
 .7566088777,0.3866191185,0.4800073958\H,1.5596023342,0.272722862,1.538
 2633139\C,2.790606635,1.0704151069,-0.0043365972\H,3.4925504035,1.5513
 678737,0.6622475793\H,2.9687128585,1.1715973694,-1.0668786278\S,0.5947
 837872,-0.4083404755,-0.5800038823\S,-0.7427778961,-1.282327845,0.5629
 168182\\Version=EM64L-G09RevD.01\State=2-A\HF=-874.4586609\S2=0.758398
 \S2-1=0.\S2A=0.750053\RMSD=2.851e-09\RMSF=5.039e-05\Dipole=0.8279666,0
 .5539379,0.0235403\Quadrupole=0.4527699,-0.7223842,0.2696143,1.4210569
 ,-0.0671624,-0.0123329\PG=C01 [X(C2H3S2)]\\@
```

$CH_2CHSS^{\bullet}$ B3PW91

```
1\1\GINC-ORC99\FOpt\UB3PW91\6-31G(d)\C2H3S2(2)\SNIKOO\18-Aug-2017\0\\#
  opt freq=noraman ub3pw91/6-31g(d)\\CH2=CH-SS\\0,2\C,1.753798557,0.385
 1125086,0.477191468\H,1.5520291992,0.2702643865,1.5398990126\C,2.79317
 8299,1.0721832721,-0.0063147484\H,3.4943999813,1.5547945831,0.66811761
 63\H,2.978852642,1.1765552571,-1.0723879671\S,0.5927954234,-0.41251137
 5,-0.5911197206\S,-0.744967102,-1.2843446224,0.5768303392\\Version=EM6
 4L-G09RevD.01\State=2-A\HF=-874.2227914\S2=0.757329\S2-1=0.\S2A=0.7500
 31\RMSD=4.117e-09\RMSF=7.179e-05\Dipole=0.9356128,0.6268028,0.03291\Qu
 adrupole=0.4403778,-0.7058161,0.2654383,1.3987902,0.0811828,0.09004\PG
 =C01 [X(C2H3S2)]\\@
```

```
1\1\GINC-ORC19\FOpt\UB3PW91\6-311G(2d,p)\C2H3S2(2)\SNIKOO\18-Aug-2017\
 0\\# opt freq=noraman ub3pw91/6-311g(2d,p)\\CH2=CH-SS\\0,2\C,1.7569865
 444,0.3869730737,0.4796827472\H,1.5542533463,0.2703998629,1.5386746409
 \C,2.7897350561,1.069719846,-0.0046531964\H,3.4923216307,1.5521561631,
 0.6646226815\H,2.9682016418,1.1702216135,-1.0698250568\S,0.5987815328,
 -0.4070186841,-0.5846630443\S,-0.7401927521,-1.2803978651,0.568377228\
 \Version=EM64L-G09RevD.01\State=2-A\HF=-874.3066116\S2=0.758803\S2-1=0
 .\S2A=0.750053\RMSD=1.973e-09\RMSF=6.226e-05\Dipole=0.8871338,0.593651
 8,0.021161\Quadrupole=0.4205409,-0.7899077,0.3693668,1.4654188,-0.0825
 983,-0.0170504\PG=C01 [X(C2H3S2)]\\@
```

1\1\GINC-ORC9\FOpt\UB3PW91\6-311+G(2df,p)\C2H3S2(2)\SNIKOO\18-Aug-2017
\0\\# opt freq=noraman ub3pw91/6-311+g(2df,p)\\CH2=CH-SS\\0,2\C,1.7544
6243,0.3853165689,0.4784556612\H,1.5541809983,0.2695505034,1.538757810
4\C,2.7880835342,1.06929302,-0.0049369103\H,3.4907165091,1.5514248802,
0.6643940776\H,2.9670292083,1.1707044819,-1.0700943885\S,0.5973559136,
-0.4074784012,-0.5798170103\S,-0.7317415936,-1.2767570433,0.5654567599
\\Version=EM64L-G09RevD.01\State=2-A\HF=-874.3182326\S2=0.759257\S2-1=
0.\S2A=0.750062\RMSD=3.090e-09\RMSF=5.289e-05\Dipole=0.838088,0.561338
2,0.0221829\Quadrupole=0.4078806,-0.7707257,0.3628452,1.4304964,-0.031
7278,0.0147688\PG=C01 [X(C2H3S2)]\\@

1\1\GINC-ORC14\FOpt\UB3PW91\6-311++G(3df,3pd)\C2H3S2(2)\SNIKOO\18-Aug-
2017\0\\# opt freq=noraman ub3pw91/6-311++g(3df,3pd)\\CH2=CH-SS\\0,2\C
,1.7539896755,0.384610648,0.4812560329\H,1.5580663522,0.2695602641,1.5
412298347\C,2.7868209765,1.06861777,-0.0052616542\H,3.4906019996,1.549
7675595,0.6612533798\H,2.9614046111,1.1692250944,-1.0697157232\S,0.599
0086551,-0.4041130767,-0.5739041541\S,-0.7298052701,-1.2756142493,0.55
73582842\\Version=EM64L-G09RevD.01\State=2-A\HF=-874.3224267\S2=0.7600
3\S2-1=0.\S2A=0.750072\RMSD=2.609e-09\RMSF=4.498e-05\Dipole=0.8312515,
0.5566133,0.0331348\Quadrupole=0.4700672,-0.7557563,0.2856891,1.483954
6,-0.0614333,-0.0103126\PG=C01 [X(C2H3S2)]\\@

$CH_2CHSS^\bullet$ wB97XD

1\1\GINC-ORC84\FOpt\UwB97XD\6-31G(d)\C2H3S2(2)\SNIKOO\18-Aug-2017\0\\#
opt freq=noraman uwb97xd/6-31g(d)\\CH2=CH-SS\\0,2\C,1.7538085102,0.38
66670446,0.4772217952\H,1.5507353014,0.2749159592,1.5390855616\C,2.789
5460537,1.0703548034,-0.0050960454\H,3.4879487402,1.5543734028,0.66947
20681\H,2.9758632218,1.1706532185,-1.0709585713\S,0.5950917014,-0.4151
272243,-0.5967410607\S,-0.7329065288,-1.2797831942,0.5792322525\\Versi
on=EM64L-G09RevD.01\State=2-A\HF=-874.2819986\S2=0.757682\S2-1=0.\S2A=
0.750036\RMSD=8.425e-09\RMSF=3.029e-05\Dipole=0.9046754,0.6080631,0.04
46036\Quadrupole=0.4438556,-0.6891063,0.2452506,1.3863268,0.0614295,0.
0846307\PG=C01 [X(C2H3S2)]\\@

1\1\GINC-ORC9\FOpt\UwB97XD\6-311G(2d,p)\C2H3S2(2)\SNIKOO\18-Aug-2017\0
\\# opt freq=noraman uwb97xd/6-311g(2d,p)\\CH2=CH-SS\\0,2\C,1.75698731
11,0.3887626682,0.4790756264\H,1.5532913388,0.2753110275,1.5373492317\
C,2.786757751,1.0681251758,-0.0034756809\H,3.4864095688,1.5513584254,0
.6667540356\H,2.9665943125,1.1652332075,-1.068342558\S,0.599668111,-0.
4095373677,-0.5909482515\S,-0.7296213933,-1.2771991266,0.5718035969\\V
ersion=EM64L-G09RevD.01\State=2-A\HF=-874.3659439\S2=0.759859\S2-1=0.\
S2A=0.750065\RMSD=8.606e-09\RMSF=3.613e-05\Dipole=0.8654018,0.5808051,
0.0241046\Quadrupole=0.415696,-0.7843497,0.3686537,1.4536837,-0.107539
2,-0.0254116\PG=C01 [X(C2H3S2)]\\@

1\1\GINC-ORC9\FOpt\UwB97XD\6-311G(2d,p)\C2H3S2(2)\SNIKOO\18-Aug-2017\0
\\# opt freq=noraman uwb97xd/6-311g(2d,p)\\CH2=CH-SS\\0,2\C,1.75698731
11,0.3887626682,0.4790756264\H,1.5532913388,0.2753110275,1.5373492317\
C,2.786757751,1.0681251758,-0.0034756809\H,3.4864095688,1.5513584254,0
.6667540356\H,2.9665943125,1.1652332075,-1.068342558\S,0.599668111,-0.
4095373677,-0.5909482515\S,-0.7296213933,-1.2771991266,0.5718035969\\V
ersion=EM64L-G09RevD.01\State=2-A\HF=-874.3659439\S2=0.759859\S2-1=0.\
S2A=0.750065\RMSD=8.606e-09\RMSF=3.613e-05\Dipole=0.8654018,0.5808051,
0.0241046\Quadrupole=0.415696,-0.7843497,0.3686537,1.4536837,-0.107539
2,-0.0254116\PG=C01 [X(C2H3S2)]\\@

1\1\GINC-ORC9\FOpt\UwB97XD\6-311++G(3df,3pd)\C2H3S2(2)\SNIKOO\18-Aug-2
017\0\\# opt freq=noraman uwb97xd/6-311++g(3df,3pd)\\CH2=CH-SS\\0,2\C,
1.7545075762,0.385905355,0.4815254116\H,1.5591873775,0.2739369422,1.54
08260904\C,2.7837596582,1.0669519518,-0.0040837665\H,3.4849133642,1.54
87510553,0.6626765935\H,2.9579209457,1.1646114783,-1.0682303892\S,0.60
04888566,-0.4064580189,-0.5789205392\S,-0.7206907784,-1.2716447537,0.5
584225994\\Version=EM64L-G09RevD.01\State=2-A\HF=-874.3821723\S2=0.761
079\S2-1=0.\S2A=0.750083\RMSD=6.548e-09\RMSF=2.901e-05\Dipole=0.814267
2,0.5463611,0.0366778\Quadrupole=0.485892,-0.7658315,0.2799395,1.51909
13,-0.0912163,-0.0241994\PG=C01 [X(C2H3S2)]\\@

$CH_2CHSS^{\bullet}$ M062X

1\1\GINC-ORC14\FOpt\UM062X\6-31G(d)\C2H3S2(2)\SNIKOO\18-Aug-2017\0\\#
opt freq=noraman m062x/6-31g(d)\\CH2=CH-SS\\0,2\C,1.7547976745,0.38726
64147,0.4829739561\H,1.5476956537,0.2720616002,1.5435001907\C,2.788399
3971,1.0696775737,-0.0036241243\H,3.4909589176,1.555900835,0.664726949
3\H,2.9658022745,1.164816754,-1.071331076\S,0.5997932748,-0.4116936352
,-0.5980239114\S,-0.7273601924,-1.2759755323,0.5739940157\\Version=EM6
4L-G09RevD.01\State=2-A\HF=-874.2346371\S2=0.755048\S2-1=0.\S2A=0.7500
19\RMSD=5.938e-09\RMSF=2.106e-05\Dipole=0.8948476,0.601211,0.0393356\Q
uadrupole=0.4431237,-0.6932558,0.2501321,1.3905603,0.0596786,0.0821017
\PG=C01 [X(C2H3S2)]\\@

1\1\GINC-ORC10\FOpt\UM062X\6-311G(2d,p)\C2H3S2(2)\SNIKOO\18-Aug-2017\0
\\# opt freq=noraman m062x/6-311g(2d,p)\\CH2=CH-SS\\0,2\C,1.7576749072
,0.3893536168,0.4845507655\H,1.5484954611,0.2724976833,1.5406815349\C,
2.7860071048,1.0677019545,-0.0020440215\H,3.488594738,1.5534822179,0.6
620776452\H,2.9576728465,1.1591988626,-1.0681551906\S,0.6048064425,-0.
4071071529,-0.5935284382\S,-0.7231645001,-1.2730731722,0.5686337048\\V
ersion=EM64L-G09RevD.01\State=2-A\HF=-874.32907\S2=0.7568\S2-1=0.\S2A=
0.750034\RMSD=1.607e-09\RMSF=4.551e-05\Dipole=0.86526,0.5808449,0.0203
389\Quadrupole=0.419744,-0.8194916,0.3997477,1.5040528,-0.1158152,-0.0
28155\PG=C01 [X(C2H3S2)]\\@

1\1\GINC-ORC9\FOpt\UM062X\6-311+G(2df,p)\C2H3S2(2)\SNIKOO\18-Aug-2017\
0\\# opt freq=noraman m062x/6-311+g(2df,p)\\CH2=CH-SS\\0,2\C,1.7555545
496,0.3874132304,0.4838689458\H,1.5500165951,0.2711641186,1.5417490077
\C,2.7844876917,1.0673005198,-0.002431465\H,3.4878449219,1.5527506111,
0.6613814366\H,2.95567763,1.1604079981,-1.0688532267\S,0.6035106544,-0
.4069485929,-0.5874654137\S,-0.7170050427,-1.2700338751,0.5639667152\\
Version=EM64L-G09RevD.01\State=2-A\HF=-874.3407634\S2=0.756831\S2-1=0.
\S2A=0.750035\RMSD=7.240e-09\RMSF=4.565e-05\Dipole=0.8170761,0.5487068
,0.0222843\Quadrupole=0.4286483,-0.794836,0.3661877,1.4903034,-0.05826
27,0.0053309\PG=C01 [X(C2H3S2)]\\@

1\1\GINC-ORC19\FOpt\UM062X\6-311++G(3df,3pd)\C2H3S2(2)\SNIKOO\18-Aug-2
017\0\\# opt freq=noraman m062x/6-311++g(3df,3pd)\\CH2=CH-SS\\0,2\C,1.
7554041288,0.3868066841,0.4859861066\H,1.5536540256,0.2710595351,1.542
9783945\C,2.7837231395,1.0669440221,-0.00271295\H,3.4874210822,1.55112
67514,0.6585908745\H,2.9509887938,1.1595002,-1.0678856203\S,0.60472752
4,-0.4043301475,-0.5816632744\S,-0.7158316939,-1.2690530352,0.55692246
92\\Version=EM64L-G09RevD.01\State=2-A\HF=-874.3452112\S2=0.757785\S2-
1=0.\S2A=0.750044\RMSD=7.174e-09\RMSF=4.070e-05\Dipole=0.8090498,0.543
1027,0.0298929\Quadrupole=0.498152,-0.7815592,0.2834072,1.5553889,-0.0
97737,-0.0264432\PG=C01 [X(C2H3S2)]\\@

$CH_2CHSS^{\bullet}$ M08HX


```
1\1\GINC-ORC27\FOpt\UM08HX\6-31G(d)\C2H3S2(2)\SNIKOO\18-Aug-2017\0\\#
 opt freq=noraman m08hx/6-31g(d)\\CH2=CH-SS\\0,2\C,1.7509013088,0.38530
 8384,0.4759527701\H,1.5403369402,0.27021437,1.5409131579\C,2.787741598
 3,1.0695038533,-0.0041187642\H,3.4890568667,1.5570713245,0.6716987637\
 H,2.9729701491,1.1673287148,-1.0748778769\S,0.5988515443,-0.4146865988
 ,-0.6030920891\S,-0.7197714075,-1.2726860377,0.5857400384\\Version=EM6
 4L-G16RevA.03\State=2-A\HF=-874.2408189\S2=0.755169\S2-1=0.\S2A=0.7500
 21\RMSD=4.107e-09\RMSF=2.780e-05\Dipole=0.8908759,0.5993718,0.0295132\
 Quadrupole=0.4302184,-0.7170479,0.2868296,1.4065849,0.0441131,0.078466
 8\PG=C01 [X(C2H3S2)]\\@
```

```
1\1\GINC-ORC14\FOpt\UM08HX\6-311G(2d,p)\C2H3S2(2)\SNIKOO\18-Aug-2017\0
 \\# opt freq=noraman 6-311g(2d,p) m08hx\\CH2=CH-SS\\0,2\C,1.7549193164
 ,0.3876499767,0.4795675894\H,1.5424861214,0.2700136542,1.5394143139\C,
 2.7852380457,1.0674350726,-0.0020667922\H,3.4880693456,1.5547359933,0.
 667219889\H,2.961022264,1.1602455279,-1.0714979615\S,0.6058090496,-0.4
 086706625,-0.5970277196\S,-0.7174571428,-1.2693555523,0.5766066811\\Ve
 rsion=EM64L-G16RevA.03\State=2-A\HF=-874.3419162\S2=0.757032\S2-1=0.\S
 2A=0.750037\RMSD=7.844e-09\RMSF=3.063e-05\Dipole=0.8455255,0.5678889,0
 .0207807\Quadrupole=0.419664,-0.7993264,0.3796624,1.4813874,-0.1212783
 ,-0.0302577\PG=C01 [X(C2H3S2)]\\@
```

```
1\1\GINC-ORC10\FOpt\UM08HX\6-311+G(2df,p)\C2H3S2(2)\SNIKOO\19-Aug-2017
 \0\\# opt freq=noraman 6-311+g(2df,p) m08hx\\CH2=CH-SS\\0,2\C,1.752814
 6136,0.385792642,0.4789548868\H,1.5435603866,0.2689297649,1.5405249146
 \C,2.7836701477,1.0670921907,-0.0025678933\H,3.4870528542,1.5543304637
 ,0.6665172127\H,2.9596667755,1.1614352279,-1.0722306302\S,0.6042322304
 ,-0.4093628945,-0.5912306486\S,-0.710910008,-1.2661633847,0.572248158\
 \Version=EM64L-G16RevA.03\State=2-A\HF=-874.3543057\S2=0.757618\S2-1=0
 .\S2A=0.750045\RMSD=9.913e-09\RMSF=2.803e-05\Dipole=0.7983939,0.536425
 9,0.0187208\Quadrupole=0.4150775,-0.7768365,0.361759,1.4549002,-0.0610
 901,0.0061002\PG=C01 [X(C2H3S2)]\\@
```

```
1\1\GINC-ORC19\FOpt\UM08HX\6-311++G(3df,3pd)\C2H3S2(2)\SNIKOO\19-Aug-2
 017\0\\# opt freq=noraman 6-311++g(3df,3pd) m08hx\\CH2=CH-SS\\0,2\C,1.
 7533136038,0.3853909156,0.4818750661\H,1.5462869754,0.2683771864,1.541
 716994\C,2.7831965233,1.066909308,-0.0027137109\H,3.4868539203,1.55314
 00129,0.6627952185\H,2.9546753384,1.1599218088,-1.0706408235\S,0.60521
 42819,-0.4066402486,-0.585983312\S,-0.709453643,-1.2650449731,0.565166
 5678\\Version=EM64L-G16RevA.03\State=2-A\HF=-874.3599702\S2=0.758518\S
 2-1=0.\S2A=0.750056\RMSD=4.596e-09\RMSF=5.879e-05\Dipole=0.8005152,0.5
 378375,0.0294033\Quadrupole=0.4930855,-0.7774655,0.28438,1.5491141,-0.
 0958683,-0.0208475\PG=C01 [X(C2H3S2)]\\@
```


$CH_2CHSS^{\bullet}$ QCISD


```
1\1\GINC-ORC28\FOpt\UQCISD-FC\6-311+G(2df,p)\C2H3S2(2)\SNIKOO\20-Aug-2
 017\0\\# opt freq=noraman 6-311+g(2df,p) qcisd\\CH2=CH-SS\\0,2\C,-1.22
 57853733,-0.5186149033,0.000226508\H,-0.8871748001,-1.5498114263,0.000
 8947536\C,-2.5093112121,-0.1658745205,-0.0003697297\H,-3.2873010199,-0
 .9206953232,-0.0001812862\H,-2.822614616,0.8733456537,-0.0010859795\S,
 0.0664590338,0.6905597047,-0.0000039789\S,1.7507709876,-0.334013185,0.
 0001417128\\Version=EM64L-G09RevD.01\State=2-A\HF=-872.5609454\MP2=-87
 3.1859295\MP3=-873.2298758\MP4D=-873.247053\MP4DQ=-873.2271492\PUHF=-8
 72.5711847\PMP2-0=-873.1945295\PMP3-0=-873.236058\MP4SDQ=-873.2345299\
```

QCISD=-873.2388118\S2=0.876082\S2-1=0.834497\S2A=0.756393\RMSD=8.045e-09\RMSF=9.924e-05\Dipole=-0.8828662,-0.1299428,-0.0000118\PG=C01 [X(C2H3S2)]\\@

CH_2CHSSSH B3LYP

1\1\GINC-ORC133\FOpt\RB3LYP\6-31G(d)\C2H4S3\SNIKOO\31-Jul-2017\0\\# opt freq rb3lyp/6-31g(d)\\CH2=CH-SSSH\\0,1\C,1.8514299029,0.4091041807,0.6461664396\H,1.6066785734,0.8689336846,1.6009702157\C,2.855123791,0.8425488752,-0.1194447637\H,3.4855990662,1.665141995,0.2082361751\H,3.0902546788,0.3855600916,-1.076574692\S,0.901830236,-1.0341428003,0.2152855464\S,-1.0678188948,-0.4317903703,0.5999849957\S,-1.7920016591,0.6278365653,-1.0689126798\H,-1.3855668644,1.8705518382,-0.713132407\\Version=EM64L-G09RevD.01\State=1-A\HF=-1273.1621941\RMSD=9.917e-09\RMSF=9.456e-06\Dipole=0.6588389,0.8323207,0.105793\Quadrupole=0.3603274,0.8962175,-1.2565449,0.9301687,-0.2745669,0.33314\PG=C01 [X(C2H4S3)]\\@

1\1\GINC-ORC167\FOpt\RB3LYP\6-311G(d)\C2H4S3\SNIKOO\31-Jul-2017\0\\# opt freq rb3lyp/6-311g(d)\\CH2=CH-SSSH\\0,1\C,1.8744913508,0.1972032942,0.5234983878\H,1.836917711,-0.0092658164,1.5888622696\C,2.6487380669,1.1480689037,0.0071526525\H,3.2962221942,1.7416458586,0.6447279065\H,2.674903395,1.3539862098,-1.0574829194\S,0.916069999,-0.8936771223,-0.5035242311\S,-0.967732055,-0.8296713749,0.4395380642\S,-1.9886484072,0.8936874418,-0.2350218414\H,-1.5121292546,1.7753636054,0.6778067112\\Version=EM64L-G09RevD.01\State=1-A\HF=-1273.2565808\RMSD=3.118e-09\RMSF=1.276e-05\Dipole=0.6505313,0.7490622,0.4324759\Quadrupole=-0.1584202,0.3620703,-0.2036501,1.2888232,0.2190417,0.960234\PG=C01 [X(C2H4S3)]\\@

1\1\GINC-ORC165\FOpt\RB3LYP\6-311+G(d)\C2H4S3\SNIKOO\31-Jul-2017\0\\# opt freq rb3lyp/6-311+g(d)\\CH2=CH-SSSH\\0,1\C,1.8606000323,0.2451474828,0.4990326201\H,1.7504577107,0.0806350912,1.5669266718\C,2.7196742378,1.1312340686,-0.0012885568\H,3.3636692756,1.7066527818,0.6559623855\H,2.82203074,1.3000485971,-1.0680941109\S,0.899316147,-0.8300069428,-0.540614994\S,-0.9637701431,-0.8415457808,0.4364799033\S,-2.0653652091,0.8493141898,-0.1905888434\H,-1.6077797912,1.7358615123,0.7277419245\\Version=EM64L-G09RevD.01\State=1-A\HF=-1273.2617275\RMSD=2.299e-09\RMSF=5.362e-06\Dipole=0.5642015,0.6743021,0.4240919\Quadrupole=-0.0259453,0.1435132,-0.1175678,1.11834,0.0287724,1.0586774\PG=C01 [X(C2H4S3)]\\@

1\1\GINC-ORC163\FOpt\RB3LYP\6-311+G(d,p)\C2H4S3\SNIKOO\31-Jul-2017\0\\# opt freq rb3lyp/6-311+g(d,p)\\CH2=CH-SSSH\\0,1\C,1.8580100667,0.2572055021,0.4951676276\H,1.7311761323,0.1034087202,1.5618901398\C,2.7340410145,1.1275111224,-0.0034608504\H,3.3757833895,1.6998460018,0.6569290123\H,2.8483440903,1.2832174422,-1.070127509\S,0.8962580081,-0.8145633434,-0.5479493506\S,-0.963009046,-0.8433296429,0.4340904427\S,-2.0783906835,0.8438493475,-0.182808855\H,-1.6233799719,1.72019585,0.7418263426\\Version=EM64L-G09RevD.01\State=1-A\HF=-1273.2713766\RMSD=7.738e-09\RMSF=9.542e-05\Dipole=0.5468025,0.6574053,0.4164927\Quadrupole=0.0415497,0.0656682,-0.107218,1.0536759,0.0069531,1.0661334\PG=C01 [X(C2H4S3)]\\@

1\1\GINC-ORC162\FOpt\RB3LYP\6-311G(2d,p)\C2H4S3\SNIKOO\31-Jul-2017\0\\# opt freq rb3lyp/6-311g(2d,p)\\CH2=CH-SSSH\\0,1\C,1.8699598817,0.2168607616,0.5186487515\H,1.8101436197,0.026622189,1.5842691998\C,2.6715175749,1.1405759027,0.0039810041\H,3.3222180927,1.724558926,0.6445594932

\H,2.7170473226,1.3308167486,-1.061413265\S,0.8988431195,-0.860023835,
-0.5073856246\S,-0.96847066,-0.8262077481,0.4334900551\S,-1.9978114322
,0.8806390569,-0.2307332925\H,-1.544614519,1.7434989983,0.7001406785\\
Version=EM64L-G09RevD.01\State=1-A\HF=-1273.2867749\RMSD=5.645e-09\RMS
F=2.065e-05\Dipole=0.578325,0.6519847,0.3956393\Quadrupole=0.0667356,0
.1850128,-0.2517484,1.1471649,0.2940899,0.8576351\PG=C01 [X(C2H4S3)]\\
@

1\1\GINC-ORC161\FOpt\RB3LYP\6-311+G(df,p)\C2H4S3\SNIKOO\31-Jul-2017\0\
\# opt freq rb3lyp/6-311+g(df,p)\\CH2=CH-SSSH\\0,1\C,1.8518849917,0.27
01226542,0.4870267234\H,1.7060345699,0.1292564904,1.5532519665\C,2.745
7586384,1.121782941,-0.0041793811\H,3.3844232198,1.692422966,0.6604387
38\H,2.8811616948,1.2661160985,-1.0699096028\S,0.8870097249,-0.7965204
952,-0.5552947904\S,-0.955628423,-0.8434735693,0.4280946446\S,-2.08594
56833,0.8283325096,-0.1683994474\H,-1.6358657332,1.7093014048,0.754528
1491\\Version=EM64L-G09RevD.01\State=1-A\HF=-1273.2828978\RMSD=9.264e-
09\RMSF=2.740e-05\Dipole=0.5354304,0.6424338,0.4183948\Quadrupole=0.03
19917,0.0172697,-0.0492614,1.0574254,-0.0256481,1.1126987\PG=C01 [X(C2
H4S3)]\\@

1\1\GINC-ORC160\FOpt\RB3LYP\6-311+G(2df,p)\C2H4S3\SNIKOO\31-Jul-2017\0
\\# opt freq rb3lyp/6-311+g(2df,p)\\CH2=CH-SSSH\\0,1\C,1.849961503,0.2
644585725,0.4932114944\H,1.7184613631,0.1177387164,1.5597752325\C,2.73
51641159,1.1221211616,-0.0009821312\H,3.381005478,1.6885599693,0.65924
34398\H,2.8569339948,1.2736305956,-1.0667450919\S,0.8767751731,-0.7903
069254,-0.5436947534\S,-0.9580992899,-0.833033267,0.4234154878\S,-2.05
70301258,0.8319319886,-0.1876080651\H,-1.6243392123,1.7022401883,0.748
9413871\\Version=EM64L-G09RevD.01\State=1-A\HF=-1273.3013161\RMSD=7.73
1e-09\RMSF=1.474e-05\Dipole=0.4877398,0.587334,0.3875215\Quadrupole=0.
0843679,-0.0148294,-0.0695386,1.0229482,0.1050544,0.9703178\PG=C01 [X(
C2H4S3)]\\@

1\1\GINC-ORC159\FOpt\RB3LYP\6-311++G(3df,3pd)\C2H4S3\SNIKOO\31-Jul-201
7\0\\# opt freq rb3lyp/6-311++g(3df,3pd)\\CH2=CH-SSSH\\0,1\C,1.8471380
31,0.2745087087,0.4888553222\H,1.7033328484,0.1393730805,1.5537321585\
C,2.7479035746,1.1174647356,-0.0029432711\H,3.3929356783,1.6795763804,
0.6589357616\H,2.8823281888,1.2584281828,-1.0668925849\S,0.8735516003,
-0.7732218628,-0.5478532312\S,-0.9531750084,-0.8305442448,0.4152409838
\S,-2.0673328514,0.8224767738,-0.1777574189\H,-1.6478490617,1.68927924
6,0.7642392799\\Version=EM64L-G09RevD.01\State=1-A\HF=-1273.3075129\RM
SD=2.835e-09\RMSF=1.897e-05\Dipole=0.4736673,0.5612702,0.3710099\Quadr
upole=0.2451915,-0.1368131,-0.1083784,1.0005893,0.1226001,0.9112379\PG
=C01 [X(C2H4S3)]\\@

$CH_2CHSSSH$ B3PW91

1\1\GINC-ORC81\FOpt\RB3PW91\6-31G(d)\C2H4S3\SNIKOO\31-Jul-2017\0\\# op
t freq rb3pw91/6-31g(d)\\CH2=CH-SSSH\\0,1\C,1.8586629763,0.2340565352,
0.5127738077\H,1.7746956238,0.0592200003,1.5836668873\C,2.6995311603,1
.133711486,-0.0003093124\H,3.3553395495,1.7091879004,0.6481430598\H,2.
7740646893,1.3097576927,-1.0700685919\S,0.8921965326,-0.8318255768,-0.
5197007505\S,-0.9594323621,-0.8240067015,0.4189940346\S,-2.0258059364,
0.8537359384,-0.2091504908\H,-1.5904192334,1.7335037252,0.7212083562\\
Version=EM64L-G09RevD.01\State=1-A\HF=-1272.9804701\RMSD=6.344e-09\RMS
F=5.929e-06\Dipole=0.647659,0.7395939,0.4720535\Quadrupole=0.1198326,0
.1276279,-0.2474605,1.1519811,0.0412421,1.0603568\PG=C01 [X(C2H4S3)]\\
@

1\1\GINC-ORC32\FOpt\RB3PW91\6-311G(d)\C2H4S3\SNIKOO\31-Jul-2017\0\\# o
pt freq rb3pw91/6-311g(d)\\CH2=CH-SSSH\\0,1\C,1.8558738421,0.214922365
7,0.5132976295\H,1.7918920418,0.0220234682,1.58105412\C,2.6612782812,1
.1420360526,0.0026508564\H,3.3074813437,1.7290027764,0.6484971579\H,2.
7150806174,1.3331891546,-1.0644979487\S,0.9042210841,-0.8644730078,-0.
5147603199\S,-0.9516486486,-0.8251687526,0.4359969979\S,-1.9904878893,
0.8701639768,-0.2124241509\H,-1.5148576723,1.755644966,0.6957426579\\V
ersion=EM64L-G09RevD.01\State=1-A\HF=-1273.0699462\RMSD=3.652e-09\RMSF
=2.850e-05\Dipole=0.6336059,0.7568318,0.4381569\Quadrupole=-0.181973,0
.3052932,-0.1233202,1.2677965,0.1316476,1.0368129\PG=C01 [X(C2H4S3)]\\
@

1\1\GINC-ORC24\FOpt\RB3PW91\6-311+G(d)\C2H4S3\SNIKOO\31-Jul-2017\0\\#
opt freq rb3pw91/6-311+g(d)\\CH2=CH-SSSH\\0,1\C,1.8439732541,0.2576233
152,0.491495935\H,1.7154997297,0.1038106163,1.560082289\C,2.7225469926
,1.1265409502,-0.0030917173\H,3.3637725408,1.6990457453,0.6603842782\H
,2.8436568073,1.2831870716,-1.070586496\S,0.8902683347,-0.8082487319,-
0.5477122778\S,-0.9482840582,-0.8359583332,0.4316409729\S,-2.056132026
4,0.8308014602,-0.1748738257\H,-1.5964685745,1.7205389064,0.7382178416
\\Version=EM64L-G09RevD.01\State=1-A\HF=-1273.0752535\RMSD=8.112e-09\R
MSF=1.961e-05\Dipole=0.5584965,0.6895842,0.43286\Quadrupole=-0.0277056
,0.1040867,-0.0763811,1.0986149,-0.0296414,1.1300096\PG=C01 [X(C2H4S3)
]\\@

1\1\GINC-ORC18\FOpt\RB3PW91\6-311+G(d,p)\C2H4S3\SNIKOO\31-Jul-2017\0\\
opt freq rb3pw91/6-311+g(d,p)\\CH2=CH-SSSH\\0,1\C,1.8409506066,0.270
8469884,0.4865357502\H,1.6935726307,0.1284657936,1.5536744328\C,2.7383
659559,1.1224010514,-0.0041592235\H,3.376540802,1.691100756,0.66416973
95\H,2.8749451523,1.2650696844,-1.0710149145\S,0.8865082987,-0.7915685
955,-0.5561443156\S,-0.9473444792,-0.8378815152,0.4279489532\S,-2.0709
425445,0.8251559484,-0.1679516072\H,-1.6137634225,1.7037508884,0.75249
81849\\Version=EM64L-G09RevD.01\State=1-A\HF=-1273.0848553\RMSD=2.182e
-09\RMSF=1.451e-05\Dipole=0.5414459,0.670031,0.4248288\Quadrupole=0.04
94659,0.017234,-0.0666999,1.0323634,-0.0528663,1.1389804\PG=C01 [X(C2H
4S3)]\\@

1\1\GINC-ORC14\FOpt\RB3PW91\6-311G(2d,p)\C2H4S3\SNIKOO\31-Jul-2017\0\\
opt freq rb3pw91/6-311g(2d,p)\\CH2=CH-SSSH\\0,1\C,1.8521335371,0.233
5959702,0.5106615244\H,1.7673174681,0.0560773814,1.5780016706\C,2.6828
824358,1.1340908996,0.0019154259\H,3.3331187275,1.7115202382,0.6502271
112\H,2.7536738057,1.3095506073,-1.0656810132\S,0.8877501934,-0.830107
8802,-0.518360412\S,-0.9549229999,-0.8211709886,0.4263261993\S,-1.9970
050498,0.8602945639,-0.2143423193\H,-1.5461151179,1.7234902081,0.71680
8813\\Version=EM64L-G09RevD.01\State=1-A\HF=-1273.1020974\RMSD=4.418e-
09\RMSF=8.216e-06\Dipole=0.5592654,0.6559607,0.4016006\Quadrupole=0.05
40352,0.1164894,-0.1705246,1.121836,0.2276108,0.9220285\PG=C01 [X(C2H4
S3)]\\@

1\1\GINC-ORC280\FOpt\RB3PW91\6-311G(df,p)\C2H4S3\SNIKOO\11-Sep-2018\0\
\# opt freq=noraman b3pw91/6-311g(df,p)\\CH2=CH-SSSH\\0,1\C,1.83874275
95,0.2189809636,0.5087934949\H,1.7386983773,0.048744957,1.5770238376\C
,2.6827712891,1.110667364,0.0041529619\H,3.3273748213,1.6902243144,0.6
566219874\H,2.7701891526,1.27797698,-1.0639431127\S,0.881693335,-0.850
5778815,-0.5223069874\S,-0.9621795268,-0.8247712156,0.4239949693\S,-2.
0058104803,0.8646417425,-0.2070894583\H,-1.5317047278,1.7403757756,0.7
067753073\\Version=EM64L-G09RevE.01\State=1-A\HF=-1273.0912963\RMSD=5.
526e-09\RMSF=1.535e-05\Dipole=0.6123289,0.7183239,0.4312927\Quadrupole

=-0.0900173,0.1546502,-0.0646329,1.231693,0.090702,1.0731917\PG=C01 [X
(C2H4S3)]\\@

1\1\GINC-ORC8\FOpt\RB3PW91\6-311+G(df,p)\C2H4S3\SNIKOO\31-Jul-2017\0\\
opt freq rb3pw91/6-311+g(df,p)\\CH2=CH-SSSH\\0,1\C,1.8364297967,0.28
39367788,0.4773923672\H,1.6743933514,0.153948045,1.5439683588\C,2.7480
212195,1.1187811384,-0.0089258121\H,3.3844481918,1.6871565883,0.660951
0304\H,2.9001956421,1.2503488191,-1.0749331849\S,0.8779322172,-0.77421
87423,-0.5629166089\S,-0.9388261498,-0.8402442044,0.4259819825\S,-2.07
81900205,0.8072594361,-0.1472193718\H,-1.6255712483,1.6903731411,0.771
2582387\\Version=EM64L-G09RevD.01\State=1-A\HF=-1273.0965684\RMSD=5.77
2e-09\RMSF=1.715e-05\Dipole=0.5303516,0.6564219,0.4261499\Quadrupole=0
.0355189,-0.0269693,-0.0085496,1.0395339,-0.0852018,1.179932\PG=C01 [X
(C2H4S3)]\\@

1\1\GINC-ORC3\FOpt\RB3PW91\6-311+G(2df,p)\C2H4S3\SNIKOO\31-Jul-2017\0\
\# opt freq rb3pw91/6-311+g(2df,p)\\CH2=CH-SSSH\\0,1\C,1.8341121988,0.
2767648238,0.4869328109\H,1.6847968875,0.1403862365,1.5538569911\C,2.7
38103959,1.1170287707,-0.001938003\H,3.3822882571,1.6804209721,0.66399
66557\H,2.8771603109,1.25589802,-1.0683781216\S,0.8675019344,-0.767532
8859,-0.5507277961\S,-0.9447486972,-0.8278493443,0.4175376051\S,-2.047
2205084,0.8159977248,-0.1756056343\H,-1.613161342,1.6862266822,0.75988
24922\\Version=EM64L-G09RevD.01\State=1-A\HF=-1273.1167689\RMSD=2.416e
-09\RMSF=1.414e-05\Dipole=0.4812319,0.5968345,0.3953655\Quadrupole=0.0
875033,-0.0637826,-0.0237208,1.0084387,0.0615833,1.0278212\PG=C01 [X(C
2H4S3)]\\@

1\1\GINC-ORC13\FOpt\RB3PW91\6-311++G(3df,3pd)\C2H4S3\SNIKOO\31-Jul-201
7\0\\# opt freq rb3pw91/6-311++g(3df,3pd)\\CH2=CH-SSSH\\0,1\C,1.831517
7679,0.2856072046,0.4829545668\H,1.6713852084,0.1611612234,1.548414851
\C,2.7475151221,1.1137099349,-0.0046902757\H,3.3901329721,1.6753049403
,0.6619016989\H,2.8971519553,1.2418129784,-1.0695574099\S,0.8658549947
,-0.7543992961,-0.5531804583\S,-0.9396505632,-0.8269395944,0.410474557
9\S,-2.0542247204,0.8074688966,-0.165852713\H,-1.630849737,1.673614712
3,0.7750921823\\Version=EM64L-G09RevD.01\State=1-A\HF=-1273.122372\RMS
D=3.570e-09\RMSF=2.201e-05\Dipole=0.4689561,0.5724638,0.3777556\Quadru
pole=0.2452522,-0.1733323,-0.0719198,1.0030037,0.0840766,0.9643115\PG=
C01 [X(C2H4S3)]\\@

CH_2CHSSSH wB97XD

1\1\GINC-ORC47\FOpt\RwB97XD\6-31G(d)\C2H4S3\SNIKOO\31-Jul-2017\0\\# op
t freq rwb97xd/6-31g(d)\\CH2=CH-SSSH\\0,1\C,1.8558347698,0.1725365334,
0.538890435\H,1.8499483262,-0.0362030319,1.6059207259\C,2.57782497,1.1
573607054,0.0105802492\H,3.2130352381,1.7751464866,0.6390053605\H,2.56
92087245,1.3660402219,-1.0552472418\S,0.9083632296,-0.9301768066,-0.47
7648968\S,-0.959647701,-0.8213752183,0.4143135053\S,-1.8596808286,0.92
47301023,-0.2611345959\H,-1.3760537284,1.7692820071,0.6708775299\\Vers
ion=EM64L-G09RevD.01\State=1-A\HF=-1273.0722248\RMSD=5.937e-09\RMSF=1.
861e-05\Dipole=0.6723804,0.7733996,0.4813667\Quadrupole=-0.1719143,0.4
696459,-0.2977316,1.4518919,0.2639768,0.9348326\PG=C01 [X(C2H4S3)]\\@

1\1\GINC-ORC50\FOpt\RwB97XD\6-311G(d)\C2H4S3\SNIKOO\31-Jul-2017\0\\# o
pt freq rwb97xd/6-311g(d)\\CH2=CH-SSSH\\0,1\C,1.8508300476,0.153174467
2,0.5413545907\H,1.8679616972,-0.0708606533,1.6038770882\C,2.530516103
4,1.1652256916,0.0173500396\H,3.1530649708,1.7964448211,0.6435157157\H
,2.4995592056,1.3852573398,-1.0446313547\S,0.9218241658,-0.9661332417,

```
-0.4709740082\S,-0.9541250441,-0.8206984059,0.4238730684\S,-1.80925743
29,0.9474480809,-0.2715315591\H,-1.2815407134,1.7874829002,0.642723419
3\\Version=EM64L-G09RevD.01\State=1-A\HF=-1273.1621037\RMSD=8.663e-09\
RMSF=2.804e-05\Dipole=0.6617853,0.7856583,0.4505388\Quadrupole=-0.4759
058,0.6574965,-0.1815907,1.6017959,0.3708419,0.9084186\PG=C01 [X(C2H4S
3)]\\@
```

```
1\1\GINC-ORC51\FOpt\RwB97XD\6-311+G(d)\C2H4S3\SNIKOO\31-Jul-2017\0\\#
 opt freq rwb97xd/6-311+g(d)\\CH2=CH-SSSH\\0,1\C,1.8325827918,0.2106921
 78,0.5125004079\H,1.7557176781,0.0317623505,1.5812769104\C,2.624273416
 5,1.1475030459,0.0041834399\H,3.2464823288,1.7547802231,0.6536716036\H
 ,2.69005485,1.3287354956,-1.0637726685\S,0.9023594627,-0.8906580374,-0
 .516346209\S,-0.9508407272,-0.8413657667,0.4263945418\S,-1.9164193116,
 0.8915678798,-0.2125039453\H,-1.4053774892,1.7443236312,0.7001529191\\
 Version=EM64L-G09RevD.01\State=1-A\HF=-1273.1672503\RMSD=6.262e-09\RMS
 F=1.729e-05\Dipole=0.5841949,0.7131915,0.4426016\Quadrupole=-0.2401959
 ,0.3963051,-0.1561092,1.3967118,0.1398581,1.0248455\PG=C01 [X(C2H4S3)]
 \\@
```

```
1\1\GINC-ORC54\FOpt\RwB97XD\6-311+G(d,p)\C2H4S3\SNIKOO\31-Jul-2017\0\\
 # opt freq rwb97xd/6-311+g(d,p)\\CH2=CH-SSSH\\0,1\C,1.8298284611,0.223
 4700201,0.5081475805\H,1.7351411451,0.0541587819,1.5765108003\C,2.6418
 075791,1.1438398796,0.0021695551\H,3.2630433543,1.7461976202,0.6557694
 584\H,2.7221111514,1.3132224908,-1.0660253336\S,0.8981661394,-0.873546
 9756,-0.5241673461\S,-0.9502576511,-0.8439036833,0.4255692919\S,-1.935
 1116389,0.8840428475,-0.2045566963\H,-1.4258955405,1.7298600187,0.7121
 396898\\Version=EM64L-G09RevD.01\State=1-A\HF=-1273.1765846\RMSD=7.046
 e-09\RMSF=2.506e-05\Dipole=0.5677392,0.6926452,0.4322608\Quadrupole=-0
 .148494,0.2987507,-0.1502567,1.3296929,0.1219805,1.0242472\PG=C01 [X(C
 2H4S3)]\\@
```

```
1\1\GINC-ORC53\FOpt\RwB97XD\6-311G(2d,p)\C2H4S3\SNIKOO\31-Jul-2017\0\\
 # opt freq rwb97xd/6-311g(2d,p)\\CH2=CH-SSSH\\0,1\C,1.8457863259,0.172
 91091,0.5435243661\H,1.8324901827,-0.0358201385,1.6076236356\C,2.55957
 1398,1.1581998195,0.02316399\H,3.1832977893,1.7800695465,0.6551771679\
 H,2.5552025404,1.3632924769,-1.0409580787\S,0.910731627,-0.933922702,-
 0.4737610386\S,-0.9561103828,-0.8183171688,0.411980718\S,-1.8267695205
 ,0.92995822,-0.2847038234\H,-1.32536696,1.7609700365,0.6435100631\\Ver
 sion=EM64L-G09RevD.01\State=1-A\HF=-1273.1939301\RMSD=5.264e-09\RMSF=1
 .819e-05\Dipole=0.5849686,0.6904928,0.4093315\Quadrupole=-0.2263537,0.
 4731827,-0.2468291,1.4179929,0.4382634,0.7836895\PG=C01 [X(C2H4S3)]\\@
```

```
1\1\GINC-ORC272\FOpt\RwB97XD\6-311G(df,p)\C2H4S3\SNIKOO\11-Sep-2018\0\
 \# opt freq=noraman wb97xd/6-311g(df,p)\\CH2=CH-SSSH\\0,1\C,1.83203346
 3,0.1576928444,0.5345789879\H,1.8097707033,-0.0463583184,1.6004997538\
 C,2.5567463103,1.1361436582,0.0138096446\H,3.1800566666,1.7580487896,0
 .6467861164\H,2.5626837089,1.3364912955,-1.0517167377\S,0.8976848958,-
 0.9510726329,-0.4809436438\S,-0.9645849094,-0.8239414729,0.4173026881\
 S,-1.8327247076,0.9384746729,-0.2562291839\H,-1.3018911308,1.770784163
 6,0.6599353748\\Version=EM64L-G09RevE.01\State=1-A\HF=-1273.1833272\RM
 SD=7.698e-09\RMSF=1.924e-05\Dipole=0.6432295,0.7457164,0.4403369\Quadr
 upole=-0.3437337,0.4592688,-0.1155351,1.5777381,0.3206238,0.9392507\PG
 =C01 [X(C2H4S3)]\\@
```

```
1\1\GINC-ORC55\FOpt\RwB97XD\6-311+G(df,p)\C2H4S3\SNIKOO\31-Jul-2017\0\
 \# opt freq rwb97xd/6-311+g(df,p)\\CH2=CH-SSSH\\0,1\C,1.8243980254,0.2
 34154679,0.5047053345\H,1.7119001353,0.0758637837,1.5730142584\C,2.654
```

69492,1.1376660441,0.0046072172\H,3.2737979962,1.7381183656,0.66155926
19\H,2.7540182692,1.2977943526,-1.0632387788\S,0.891510547,-0.85805401
18,-0.5279175902\S,-0.9460367224,-0.8428398202,0.4189912419\S,-1.94408
8132,0.870336597,-0.199453924\H,-1.4413620386,1.7243010099,0.713289979
2\\Version=EM64L-G09RevD.01\State=1-A\HF=-1273.1884614\RMSD=5.384e-09\
RMSF=1.608e-05\Dipole=0.5590669,0.6806261,0.434914\Quadrupole=-0.13858
01,0.2403814,-0.1018012,1.3304966,0.0965426,1.0616859\PG=C01 [X(C2H4S3
)]\\@

1\1\GINC-ORC56\FOpt\RwB97XD\6-311+G(2df,p)\C2H4S3\SNIKOO\31-Jul-2017\0
\\# opt freq rwb97xd/6-311+g(2df,p)\\CH2=CH-SSSH\\0,1\C,1.824905287,0.
2314954662,0.5094540166\H,1.7231908906,0.0705407651,1.5776530932\C,2.6
480889396,1.1386027297,0.0067984947\H,3.2725105143,1.737004149,0.65971
4648\H,2.7369364369,1.3024998394,-1.0609404963\S,0.8839439908,-0.85115
24372,-0.5178366871\S,-0.9460567703,-0.8339060677,0.4153195796\S,-1.92
16593608,0.8686820018,-0.2173997928\H,-1.443026928,1.7135745537,0.7127
941441\\Version=EM64L-G09RevD.01\State=1-A\HF=-1273.2086822\RMSD=3.635
e-09\RMSF=1.227e-05\Dipole=0.5055981,0.6252354,0.4022685\Quadrupole=-0
.0920574,0.2076433,-0.1155859,1.2618945,0.1997463,0.9275582\PG=C01 [X(
C2H4S3)]\\@

1\1\GINC-ORC56\FOpt\RwB97XD\6-311++G(3df,3pd)\C2H4S3\SNIKOO\31-Jul-201
7\0\\# opt freq rwb97xd/6-311++g(3df,3pd)\\CH2=CH-SSSH\\0,1\C,1.823240
1811,0.2422609747,0.5049285571\H,1.7078741018,0.093018133,1.5718296431
\C,2.6655282919,1.1330212803,0.0045424748\H,3.2906780476,1.7254023247,
0.6592735239\H,2.7686519172,1.2867790325,-1.0616346954\S,0.8804173998,
-0.8325493735,-0.5216595607\S,-0.9417545049,-0.8311425019,0.4101777043
\S,-1.9395983095,0.857106219,-0.2076412834\H,-1.476204125,1.7034449111
,0.7257406363\\Version=EM64L-G09RevD.01\State=1-A\HF=-1273.2146216\RMS
D=9.575e-09\RMSF=1.663e-05\Dipole=0.4895022,0.6044387,0.3843146\Quadru
pole=0.0776095,0.0790657,-0.1566752,1.2196486,0.2129966,0.8745303\PG=C
01 [X(C2H4S3)]\\@

CH_2CHSSSH M062X

1\1\GINC-ORC76\FOpt\RM062X\6-31G(d)\C2H4S3\SNIKOO\31-Jul-2017\0\\# opt
freq m062x/6-31g(d)\\CH2=CH-SSSH\\0,1\C,1.8319465314,0.176636919,0.54
54245328\H,1.8019460089,-0.0193948535,1.6143861352\C,2.554111247,1.159
7360294,0.0150988616\H,3.1723068269,1.7945868576,0.6427121206\H,2.5605
805646,1.3455020189,-1.0546570436\S,0.9189906897,-0.9445558241,-0.4803
679468\S,-0.9576019663,-0.8330133747,0.3917269899\S,-1.8021799172,0.94
43480891,-0.2708257266\H,-1.3012669851,1.7534951385,0.6820590767\\Vers
ion=EM64L-G09RevD.01\State=1-A\HF=-1273.0054245\RMSD=7.513e-09\RMSF=4.
525e-05\Dipole=0.6414315,0.7426361,0.4748489\Quadrupole=-0.2585922,0.4
710976,-0.2125055,1.5145576,0.2446296,0.9865499\PG=C01 [X(C2H4S3)]\\@

1\1\GINC-ORC75\FOpt\RM062X\6-311G(d)\C2H4S3\SNIKOO\31-Jul-2017\0\\# op
t freq m062x/6-311g(d)\\CH2=CH-SSSH\\0,1\C,1.8164972629,0.1587297055,0
.5443866947\H,1.8011685895,-0.0518669891,1.6091387902\C,2.5000682325,1
.169031232,0.0216781108\H,3.0990835228,1.8177978279,0.6515433918\H,2.4
941477151,1.3653417551,-1.0447093257\S,0.9283288698,-0.9815925319,-0.4
794100966\S,-0.951871474,-0.8356614537,0.4041009042\S,-1.7456538556,0.
9671871207,-0.2670774575\H,-1.162935863,1.7683743333,0.6459059881\\Ver
sion=EM64L-G09RevD.01\State=1-A\HF=-1273.1110231\RMSD=4.351e-09\RMSF=3
.707e-05\Dipole=0.6486365,0.7585047,0.4391998\Quadrupole=-0.5905401,0.
692601,-0.1020609,1.7930969,0.3679291,0.9544228\PG=C01 [X(C2H4S3)]\\@

```
1\1\GINC-ORC69\FOpt\RM062X\6-311+G(d)\C2H4S3\SNIKOO\31-Jul-2017\0\\# o
 pt freq m062x/6-311+g(d)\\CH2=CH-SSSH\\0,1\C,1.8132613906,0.1805810772
 ,0.5311177985\H,1.7639107162,-0.0146477331,1.5980629461\C,2.5414410385
 ,1.1634560972,0.0142046262\H,3.1446958502,1.799945495,0.6525168419\H,2
 .5672781326,1.3496806068,-1.0537708784\S,0.918004028,-0.9471605318,-0.
 4998064642\S,-0.9497732756,-0.8457222462,0.4108389539\S,-1.800534276,0
 .9402228025,-0.236921337\H,-1.2194506043,1.7509854325,0.6693145129\\Ve
 rsion=EM64L-G09RevD.01\State=1-A\HF=-1273.1165357\RMSD=5.930e-09\RMSF=
 3.648e-05\Dipole=0.5859248,0.6983136,0.4283673\Quadrupole=-0.4108283,0
 .5963392,-0.1855109,1.6293866,0.2975299,0.9469925\PG=C01 [X(C2H4S3)]\\
 @

1\1\GINC-ORC10\FOpt\RM062X\6-311+G(d,p)\C2H4S3\SNIKOO\12-Sep-2017\0\\#
  opt=calcfc freq=noraman 6-311+g(d,p) nosymm geom=connectivity m062x\\
 CH2=CH-SSSH 1\\0,1\C,1.766557248,0.2188271406,0.5261230519\H,1.7091896
 965,0.0327658287,1.5939079643\C,2.4982412658,1.1963106704,0.0042482767
 \H,3.0964469649,1.8375264231,0.6415021119\H,2.5285346328,1.3706079707,
 -1.0650491316\S,0.8795175732,-0.9190362982,-0.5011128248\S,-0.98363880
 86,-0.8499036293,0.4196170785\S,-1.872002817,0.9205670395,-0.225994148
 5\H,-1.3007177556,1.7351618546,0.6799926214\\Version=EM64L-G09RevD.01\
 HF=-1273.1245668\RMSD=7.778e-09\RMSF=3.520e-05\Dipole=0.5699405,0.6929
 871,0.4180585\Quadrupole=-0.3622972,0.5422923,-0.1799952,1.5713025,0.2
 748693,0.9412051\PG=C01 [X(C2H4S3)]\\@

1\1\GINC-ORC66\FOpt\RM062X\6-311G(2d,p)\C2H4S3\SNIKOO\31-Jul-2017\0\\#
  opt freq m062x/6-311g(2d,p)\\CH2=CH-SSSH\\0,1\C,1.8255652263,0.165175
 3366,0.5446696243\H,1.8098525369,-0.0408900681,1.6086391973\C,2.513873
 1758,1.1657538157,0.0182029581\H,3.1197787437,1.8093340691,0.644642811
 \H,2.5029604365,1.3574951601,-1.0475933003\S,0.9200606835,-0.963497912
 2,-0.4743786901\S,-0.9521999682,-0.827238992,0.3990398519\S,-1.7432967
 428,0.962498646,-0.2795367765\H,-1.2177610917,1.7487109449,0.671871324
 2\\Version=EM64L-G09RevD.01\State=1-A\HF=-1273.1412646\RMSD=5.826e-09\
 RMSF=2.190e-05\Dipole=0.5742324,0.6772529,0.404999\Quadrupole=-0.38096
 37,0.5256008,-0.1446371,1.5741144,0.4483589,0.8056377\PG=C01 [X(C2H4S3
 )]\\@

1\1\GINC-ORC272\FOpt\RM062X\6-311G(df,p)\C2H4S3\SNIKOO\11-Sep-2018\0\\
 # opt freq=noraman m062x/6-311g(df,p)\\CH2=CH-SSSH\\0,1\C,1.8027352286
 ,0.1570353688,0.536586759\H,1.7669718107,-0.0415207835,1.6029877365\C,
 2.5128870228,1.1458749196,0.0148281604\H,3.11490907,1.7886462725,0.647
 0246141\H,2.5253717733,1.3307733522,-1.0530764764\S,0.905187755,-0.974
 7569922,-0.485627804\S,-0.9594581631,-0.8401792376,0.4036018255\S,-1.7
 605509126,0.9581538906,-0.2463478667\H,-1.1682785848,1.7522362096,0.66
 40460516\\Version=EM64L-G09RevE.01\State=1-A\HF=-1273.1315495\RMSD=3.6
 01e-09\RMSF=3.688e-05\Dipole=0.6393985,0.7280285,0.4296307\Quadrupole=
 -0.4667934,0.5144139,-0.0476205,1.7956025,0.3648348,0.9530881\PG=C01 [
 X(C2H4S3)]\\@

1\1\GINC-ORC102\FOpt\RM062X\6-311+G(df,p)\C2H4S3\SNIKOO\22-Aug-2017\0\
 \# opt freq 6-311+g(df,p) m062x\\CH2=CH-SSSH new\\0,1\C,1.7581290533,0
 .2216669735,0.5104182727\H,1.6729641682,0.0473676183,1.5787656614\C,2.
 5302902386,1.1692309896,-0.0018197856\H,3.1348356953,1.7973305714,0.64
 24072064\H,2.5907663339,1.3352729901,-1.0713640666\S,0.856899597,-0.89
 83804601,-0.5200514643\S,-0.9854437561,-0.8544894,0.4159110028\S,-1.89
 6700879,0.9072449423,-0.1887220218\H,-1.3247224511,1.7205957749,0.7185
 23195\\Version=EM64L-G09RevD.01\State=1-A\HF=-1273.1370142\RMSD=5.188e
```

-09\RMSF=3.219e-05\Dipole=0.5652174,0.6722795,0.4225504\Quadrupole=-0.
2929986,0.3907129,-0.0977142,1.5706677,0.2212788,1.0043265\PG=C01 [X(C
2H4S3)]\\@

1\1\GINC-ORC65\FOpt\RM062X\6-311+G(2df,p)\C2H4S3\SNIKOO\31-Jul-2017\0\
\# opt freq m062x/6-311+g(2df,p)\\CH2=CH-SSSH\\0,1\C,1.8151326467,0.19
31940085,0.5275139876\H,1.7577661712,0.0098987178,1.5950709029\C,2.561
148259,1.1563122435,0.007898174\H,3.1726371545,1.7859175974,0.64301036
55\H,2.5926523548,1.3320428308,-1.0605614541\S,0.9020448057,-0.9184916
988,-0.4941654514\S,-0.9460325332,-0.8338624838,0.4034166919\S,-1.7961
588204,0.9266222326,-0.2428118062\H,-1.2803570384,1.7257075521,0.70618
55897\\Version=EM64L-G09RevD.01\State=1-A\HF=-1273.1565066\RMSD=9.319e
-09\RMSF=2.446e-05\Dipole=0.5078526,0.6208237,0.3950255\Quadrupole=-0.
2678594,0.3704653,-0.1026059,1.4629292,0.3358321,0.8480748\PG=C01 [X(C
2H4S3)]\\@

1\1\GINC-ORC64\FOpt\RM062X\6-311++G(3df,3pd)\C2H4S3\SNIKOO\31-Jul-2017
\0\\# opt freq m062x/6-311++g(3df,3pd)\\CH2=CH-SSSH\\0,1\C,1.808432644
8,0.228573522,0.5102986339\H,1.7021561264,0.0742356636,1.5769771289\C,
2.617603933,1.1443039107,-0.0006815946\H,3.2318453652,1.7580787774,0.6
437048005\H,2.6996830542,1.2931325898,-1.0687000045\S,0.8906528226,-0.
8689765418,-0.5159689453\S,-0.9405858127,-0.8405786035,0.4007909361\S,
-1.8612063454,0.8924906581,-0.2076801366\H,-1.3697487882,1.6960810238,
0.7468161816\\Version=EM64L-G09RevD.01\State=1-A\HF=-1273.162733\RMSD=
6.128e-09\RMSF=2.931e-05\Dipole=0.4811412,0.5909023,0.3778533\Quadrupo
le=-0.0212236,0.1437297,-0.122506,1.3457592,0.2523006,0.8626365\PG=C01
[X(C2H4S3)]\\@

CH_2CHSSSH M08HX

1\1\GINC-ORC37\FOpt\RM08HX\6-31G(d)\C2H4S3\SNIKOO\31-Jul-2017\0\\# opt
freq 6-31g(d) m08hx\\CH2=CH-SSSH\\0,1\C,1.8372171451,0.0805679253,0.5
797963436\H,1.9406249045,-0.1579116599,1.6402570588\C,2.3550924131,1.1
833523921,0.0414692008\H,2.9319440859,1.8848694765,0.6462510455\H,2.23
11360893,1.408072768,-1.0189385694\S,0.9468184915,-1.0879907195,-0.415
3346473\S,-0.9552143999,-0.7952823272,0.377457133\S,-1.5504695577,1.04
86498517,-0.353439392\H,-0.9583161718,1.813013293,0.5880388269\\Versio
n=EM64L-G16RevA.03\State=1-A\HF=-1273.0102398\RMSD=6.729e-09\RMSF=1.34
7e-05\Dipole=0.6532168,0.7545931,0.4634213\Quadrupole=-0.6709105,0.957
3133,-0.2864028,1.7659244,0.63188,0.7989682\PG=C01 [X(C2H4S3)]\\@

1\1\GINC-ORC37\FOpt\RM08HX\6-311G(d)\C2H4S3\SNIKOO\31-Jul-2017\0\\# op
t freq 6-311g(d) m08hx\\CH2=CH-SSSH\\0,1\C,1.8076564198,0.1211383905,0
.5587406011\H,1.8289816678,-0.0954574269,1.6270741292\C,2.4161812521,1
.1764387309,0.0293361105\H,2.9878547049,1.8629291901,0.6526737184\H,2.
3748448888,1.3771339609,-1.0403438748\S,0.9458650999,-1.050049674,-0.4
532100228\S,-0.9524467068,-0.8222017732,0.3875648445\S,-1.6154875744,1
.0207423058,-0.3067445964\H,-1.0146167521,1.7866672959,0.6304660903\\V
ersion=EM64L-G16RevA.03\State=1-A\HF=-1273.1265832\RMSD=5.003e-09\RMSF
=1.590e-05\Dipole=0.6357826,0.751784,0.4398841\Quadrupole=-0.8033142,0
.8725417,-0.0692275,1.8929883,0.4311971,0.9366101\PG=C01 [X(C2H4S3)]\\
@

1\1\GINC-ORC34\FOpt\RM08HX\6-311+G(d)\C2H4S3\SNIKOO\31-Jul-2017\0\\# o
pt freq 6-311+g(d) m08hx\\CH2=CH-SSSH\\0,1\C,1.8012264971,0.1493024496
,0.5438908353\H,1.773064867,-0.0482539343,1.6163287426\C,2.4744131475,
1.1691042154,0.0214371276\H,3.052590048,1.8394431553,0.6563914774\H,2.

```
4800486504,1.3572093401,-1.0514881364\S,0.9343031114,-1.0081778977,-0.
4783673174\S,-0.9511156396,-0.8367461457,0.3962562774\S,-1.6900514922,
0.9869623758,-0.2740610643\H,-1.0956461895,1.7684974416,0.6551690579\\
Version=EM64L-G16RevA.03\State=1-A\HF=-1273.1320519\RMSD=5.535e-09\RMS
F=1.340e-05\Dipole=0.5722247,0.6935847,0.429113\Quadrupole=-0.6006184,
0.755339,-0.1547206,1.7108035,0.3261179,0.9420286\PG=C01 [X(C2H4S3)]\\
@

1\1\GINC-ORC34\FOpt\RM08HX\6-311+G(d,p)\C2H4S3\SNIKOO\31-Jul-2017\0\\#
 opt freq 6-311+g(d,p) m08hx\\CH2=CH-SSSH\\0,1\C,1.7996370333,0.163501
0967,0.5382365936\H,1.7525893262,-0.0263594361,1.6107420113\C,2.497790
4092,1.1668756984,0.0172213349\H,3.0781645261,1.8297660576,0.656291327
6\H,2.5192438499,1.3445190582,-1.056443398\S,0.930091026,-0.9893111778
,-0.4871965673\S,-0.949880211,-0.8411031658,0.3988935496\S,-1.71809457
67,0.9759041097,-0.2624899295\H,-1.130708383,1.753548759,0.6703020777\
\Version=EM64L-G16RevA.03\State=1-A\HF=-1273.1401609\RMSD=4.816e-09\RM
SF=3.539e-05\Dipole=0.5605504,0.679394,0.4201937\Quadrupole=-0.4916779
,0.6419311,-0.1502532,1.6373655,0.2929102,0.946323\PG=C01 [X(C2H4S3)]\
\@

1\1\GINC-ORC33\FOpt\RM08HX\6-311G(2d,p)\C2H4S3\SNIKOO\31-Jul-2017\0\\#
 opt freq 6-311g(2d,p) m08hx\\CH2=CH-SSSH\\0,1\C,1.8133313161,0.132227
7558,0.5557441967\H,1.8290305599,-0.0807418053,1.6228608673\C,2.435090
813,1.1751300901,0.0264494173\H,3.0161361028,1.8517206053,0.6490533399
\H,2.3942890693,1.3722211441,-1.0421451848\S,0.9334593224,-1.023679991
3,-0.4542073466\S,-0.9514598291,-0.820370733,0.3903565222\S,-1.6350579
957,1.0053950169,-0.3039105703\H,-1.0559863586,1.7654389175,0.64135575
84\\Version=EM64L-G16RevA.03\State=1-A\HF=-1273.1562792\RMSD=9.474e-09
\RMSF=1.447e-05\Dipole=0.5675426,0.6724073,0.3981553\Quadrupole=-0.563
1141,0.7434885,-0.1803744,1.6727385,0.5278655,0.7590944\PG=C01 [X(C2H4
S3)]\\@

1\1\GINC-ORC33\FOpt\RM08HX\6-311+G(df,p)\C2H4S3\SNIKOO\31-Jul-2017\0\\
# opt freq 6-311+g(df,p) m08hx\\CH2=CH-SSSH\\0,1\C,1.7936182644,0.1776
922971,0.5294512946\H,1.7250747792,-0.0015515828,1.6028950855\C,2.5171
922552,1.1622270956,0.0139351216\H,3.0985382386,1.820572752,0.65677249
88\H,2.5614098657,1.3326347417,-1.0602789469\S,0.9211053921,-0.9692238
837,-0.496099195\S,-0.9440378361,-0.8455180707,0.3973661324\S,-1.73784
83478,0.957155137,-0.2438506172\H,-1.1562196114,1.7433525138,0.6853656
263\\Version=EM64L-G16RevA.03\State=1-A\HF=-1273.1528536\RMSD=6.478e-0
9\RMSF=1.005e-05\Dipole=0.5537963,0.6682233,0.42224\Quadrupole=-0.4593
983,0.5420453,-0.082647,1.635378,0.2503283,0.9909246\PG=C01 [X(C2H4S3)
]\\@

1\1\GINC-ORC32\FOpt\RM08HX\6-311+G(2df,p)\C2H4S3\SNIKOO\31-Jul-2017\0\
\# opt freq 6-311+g(2df,p) m08hx\\CH2=CH-SSSH\\0,1\C,1.8008411208,0.16
79186003,0.534624659\H,1.7600181717,-0.0213334552,1.606547813\C,2.5017
874513,1.165116707,0.014850255\H,3.092837752,1.8206113229,0.6503848222
\H,2.5176747851,1.3466854636,-1.0574567044\S,0.9121005169,-0.966582114
5,-0.4825825763\S,-0.9445613718,-0.8333985118,0.3991873595\S,-1.715178
9211,0.9574138658,-0.2580360838\H,-1.1466865048,1.7409091219,0.6780374
557\\Version=EM64L-G16RevA.03\State=1-A\HF=-1273.1719484\RMSD=4.413e-0
9\RMSF=1.048e-05\Dipole=0.5002768,0.6175431,0.3886695\Quadrupole=-0.43
00045,0.5689029,-0.1388984,1.5299417,0.3901324,0.8201119\PG=C01 [X(C2H
4S3)]\\@
```

1\1\GINC-ORC31\FOpt\RM08HX\6-311++G(3df,3pd)\C2H4S3\SNIKOO\31-Jul-2017
\0\\# opt freq 6-311++g(3df,3pd) m08hx\\CH2=CH-SSSH\\0,1\C,1.799929907
3,0.1800024085,0.5286897618\H,1.7414670437,-0.0007873733,1.5988617756\
C,2.5247462707,1.1616170715,0.0115309336\H,3.1190722144,1.8072081081,0
.6497457227\H,2.5574675753,1.3354704297,-1.0590207575\S,0.907082008,-0
.945649666,-0.4899454741\S,-0.9401547003,-0.8341865215,0.3994451752\S,
-1.7437391382,0.9417974453,-0.243757929\H,-1.1870381808,1.7318690977,0
.6900077918\\Version=EM64L-G16RevA.03\State=1-A\HF=-1273.1797957\RMSD=
8.928e-09\RMSF=9.570e-06\Dipole=0.4942731,0.6058393,0.3768104\Quadrupo
le=-0.2465147,0.433882,-0.1873673,1.487858,0.410795,0.7629077\PG=C01 [
X(C2H4S3)]\\@

$CH_2CHSSSH$ QCISD

1\1\GINC-ORC296\FOpt\RQCISD-FC\6-311+G(2df,p)\C2H4S3\SNIKOO\20-Aug-201
7\0\\# opt freq 6-311+g(2df,p) qcisd\\CH2=CH-SSSH\\0,1\C,1.8303733378,
0.1556718425,0.5471389109\H,1.7665435296,-0.0058699598,1.6193949351\C,
2.613316882,1.0925906467,0.0152182317\H,3.2384787345,1.7144927102,0.64
76189336\H,2.6622122436,1.2541708787,-1.0563688071\S,0.8877007539,-0.9
548006005,-0.4593624279\S,-0.9672938841,-0.8060215453,0.4385660451\S,-
1.8186194915,0.9352646423,-0.2971651593\H,-1.2902731058,1.7881183852,0
.601339338\\Version=EM64L-G09RevD.01\State=1-A\HF=-1270.6957842\MP2=-1
271.5207177\MP3=-1271.5768654\MP4D=-1271.6001683\MP4DQ=-1271.5729628\M
P4SDQ=-1271.5801717\QCISD=-1271.5802513\RMSD=9.097e-09\RMSF=3.225e-05\
Dipole=0.5155637,0.6265731,0.3689404\PG=C01 [X(C2H4S3)]\\@

CH_2CHSSS^- B3LYP

1\1\GINC-ORC302\FOpt\RB3LYP\6-31G(d)\C2H3S3(1-)\SNIKOO\31-Jul-2017\0\\
opt freq b3lyp/6-31g(d)\\CH2=CH-SSS(-)\\-1,1\C,1.3364568419,-0.39784
89053,0.1548551891\H,0.9020173479,-1.3970532508,0.1383756685\C,2.64962
0793,-0.2143825059,-0.0386543577\H,3.3100702823,-1.062659117,-0.207575
9284\H,3.1081345542,0.7720148239,-0.0232864534\S,0.156928744,0.8696384
761,0.52174853\S,-1.7382518502,-0.086601037,-0.0791750402\S,-2.3680659
132,-1.484971754,1.2943524522\\Version=EM64L-G09RevD.01\State=1-A\HF=-
1272.6203834\RMSD=4.280e-09\RMSF=5.195e-06\Dipole=1.7988855,0.5627871,
-0.6646017\Quadrupole=-4.9045974,2.0937919,2.8108055,-5.2705249,3.0781
417,1.9732471\PG=C01 [X(C2H3S3)]\\@

1\1\GINC-ORC299\FOpt\RB3LYP\6-311G(d)\C2H3S3(1-)\SNIKOO\31-Jul-2017\0\
\# opt freq b3lyp/6-311g(d)\\CH2=CH-SSS(-)\\-1,1\C,1.3442516104,-0.403
2440794,0.1568445073\H,0.9151343706,-1.4022111837,0.1405358781\C,2.651
5223654,-0.2095768047,-0.0377503166\H,3.3172480156,-1.0519214082,-0.20
51530773\H,3.1001462721,0.7798941505,-0.0273255282\S,0.1569976615,0.85
58374735,0.519787252\S,-1.7223587305,-0.1249764991,-0.1138413114\S,-2.
4060307652,-1.4456649189,1.3275426561\\Version=EM64L-G09RevD.01\State=
1-A\HF=-1272.724708\RMSD=2.531e-09\RMSF=1.242e-04\Dipole=1.982028,0.63
83146,-0.7427278\Quadrupole=-5.2315742,2.3958226,2.8357517,-5.4593992,
3.373131,2.1637834\PG=C01 [X(C2H3S3)]\\@

1\1\GINC-ORC298\FOpt\RB3LYP\6-311+G(d)\C2H3S3(1-)\SNIKOO\31-Jul-2017\0
\\# opt freq b3lyp/6-311+g(d)\\CH2=CH-SSS(-)\\-1,1\C,1.3455023948,-0.4
014019643,0.0929536395\H,0.9175040734,-1.3942423125,-0.0238076262\C,2.
6661234292,-0.2058653613,-0.0109683355\H,3.3337356884,-1.0402017121,-0
.2069570579\H,3.1212329191,0.7739395487,0.1037624676\S,0.1430931003,0.
8380994663,0.4593889162\S,-1.7393121076,-0.1749500378,-0.0862462692\S,
-2.4309686975,-1.397240897,1.4325143254\\Version=EM64L-G09RevD.01\Stat

e=1-A\HF=-1272.7309807\RMSD=3.874e-09\RMSF=2.241e-06\Dipole=1.8520801,
0.5961374,-0.8036842\Quadrupole=-5.4655126,2.9250562,2.5404564,-5.1706
801,3.9724358,2.6001016\PG=C01 [X(C2H3S3)]\\@

1\1\GINC-ORC298\FOpt\RB3LYP\6-311+G(d)\C2H3S3(1-)\SNIKOO\31-Jul-2017\0
\\# opt freq b3lyp/6-311+g(d)\\CH2=CH-SSS(-)\\-1,1\C,1.3455023948,-0.4
014019643,0.0929536395\H,0.9175040734,-1.3942423125,-0.0238076262\C,2.
6661234292,-0.2058653613,-0.0109683355\H,3.3337356884,-1.0402017121,-0
.2069570579\H,3.1212329191,0.7739395487,0.1037624676\S,0.1430931003,0.
8380994663,0.4593889162\S,-1.7393121076,-0.1749500378,-0.0862462692\S,
-2.4309686975,-1.397240897,1.4325143254\\Version=EM64L-G09RevD.01\Stat
e=1-A\HF=-1272.7309807\RMSD=3.874e-09\RMSF=2.241e-06\Dipole=1.8520801,
0.5961374,-0.8036842\Quadrupole=-5.4655126,2.9250562,2.5404564,-5.1706
801,3.9724358,2.6001016\PG=C01 [X(C2H3S3)]\\@

1\1\GINC-ORC298\FOpt\RB3LYP\6-311+G(d,p)\C2H3S3(1-)\SNIKOO\31-Jul-2017
\0\\# opt freq b3lyp/6-311+g(d,p)\\CH2=CH-SSS(-)\\-1,1\C,1.3452602698,
-0.399091083,0.0713726112\H,0.914705606,-1.3863446013,-0.0742114442\C,
2.6686987496,-0.2064920748,0.00049197\H,3.3342105052,-1.0399480499,-0.
1996609643\H,3.122289156,0.7686161669,0.1461307582\S,0.1383053387,0.83
83498949,0.4282831872\S,-1.7439257556,-0.193310058,-0.0805939406\S,-2.
4226330696,-1.3836434647,1.4688278824\\Version=EM64L-G09RevD.01\State=
1-A\HF=-1272.7365012\RMSD=6.301e-09\RMSF=2.772e-06\Dipole=1.8295334,0.
589312,-0.8303144\Quadrupole=-5.5118372,2.9759244,2.5359128,-5.0681809
,4.1548362,2.7179344\PG=C01 [X(C2H3S3)]\\@

1\1\GINC-ORC296\FOpt\RB3LYP\6-311G(2d,p)\C2H3S3(1-)\SNIKOO\31-Jul-2017
\0\\# opt freq b3lyp/6-311g(2d,p)\\CH2=CH-SSS(-)\\-1,1\C,1.3440456996,
-0.4131458998,0.1344740653\H,0.9176702128,-1.4096952388,0.0752435888\C
,2.650585747,-0.207327984,-0.0250587115\H,3.3219790313,-1.0399300169,-
0.2057879988\H,3.091201984,0.7823579677,0.0283907847\S,0.1397983972,0.
8270353681,0.5032970028\S,-1.7112782979,-0.1266757178,-0.1050865476\S,
-2.3970919741,-1.4144817486,1.3551678764\\Version=EM64L-G09RevD.01\Sta
te=1-A\HF=-1272.7477533\RMSD=5.674e-09\RMSF=1.049e-05\Dipole=1.9336835
,0.618707,-0.7351982\Quadrupole=-5.4366667,2.5342916,2.9023751,-5.1288
43,3.4854162,2.1504629\PG=C01 [X(C2H3S3)]\\@

1\1\GINC-ORC296\FOpt\RB3LYP\6-311+G(df,p)\C2H3S3(1-)\SNIKOO\31-Jul-201
7\0\\# opt freq b3lyp/6-311+g(df,p)\\CH2=CH-SSS(-)\\-1,1\C,1.342049841
4,-0.4016391596,0.0572077136\H,0.9146058951,-1.386654797,-0.1105291101
\C,2.6633513067,-0.2074474943,0.0094109602\H,3.3337705365,-1.035443339
7,-0.1958998442\H,3.1142125565,0.7649557178,0.1788655791\S,0.127843167
8,0.8279857246,0.4100369919\S,-1.7359515726,-0.1921892701,-0.071422542
4\S,-2.4029709314,-1.3714306517,1.4829703119\\Version=EM64L-G09RevD.01
\State=1-A\HF=-1272.7477232\RMSD=4.620e-09\RMSF=6.473e-06\Dipole=1.857
1896,0.6120764,-0.8673142\Quadrupole=-5.4568142,3.0098661,2.4469481,-5
.1253338,4.2688236,2.8871814\PG=C01 [X(C2H3S3)]\\@

1\1\GINC-ORC295\FOpt\RB3LYP\6-311+G(2df,p)\C2H3S3(1-)\SNIKOO\31-Jul-20
17\0\\# opt freq b3lyp/6-311+g(2df,p)\\CH2=CH-SSS(-)\\-1,1\C,1.3368786
847,-0.4110247956,0.0905040082\H,0.9136445329,-1.4024063249,-0.0420366
484\C,2.6532205536,-0.2068986826,-0.0029761833\H,3.3259652547,-1.03306
39416,-0.2049558885\H,3.0984330453,0.7736233999,0.1270241034\S,0.12609
99849,0.8108202771,0.4587625403\S,-1.7158741571,-0.1519198231,-0.08091
98538\S,-2.3814570989,-1.3809933791,1.4152379821\\Version=EM64L-G09Rev
D.01\State=1-A\HF=-1272.7634929\RMSD=8.834e-09\RMSF=2.254e-05\Dipole=1
.8128572,0.6156782,-0.7761543\Quadrupole=-5.5657321,2.9192104,2.646521

7,-4.9575232,3.8719206,2.516518\PG=C01 [X(C2H3S3)]\\@

1\1\GINC-ORC289\FOpt\RB3LYP\6-311++G(3df,3pd)\C2H3S3(1-)\SNIKOO\31-Jul
-2017\0\\# opt freq b3lyp/6-311++g(3df,3pd)\\CH2=CH-SSS(-)\\-1,1\C,1.3
34981936,-0.4125648811,0.0784584846\H,0.9156257865,-1.4012388112,-0.07
26357915\C,2.6525756814,-0.2067911776,0.003675916\H,3.3265788462,-1.02
87936471,-0.2017377702\H,3.0944150383,0.7706632588,0.1526712146\S,0.12
18268418,0.8028585655,0.4427281841\S,-1.7138740533,-0.1571419079,-0.07
25508538\S,-2.3752192769,-1.3688546695,1.4300306761\\Version=EM64L-G09
RevD.01\State=1-A\HF=-1272.7683366\RMSD=9.289e-09\RMSF=1.938e-05\Dipol
e=1.7838135,0.5925457,-0.7563786\Quadrupole=-5.5739448,2.9188978,2.655
047,-4.7871622,3.847628,2.5192104\PG=C01 [X(C2H3S3)]\\@

CH_2CHSSS^- B3PW91

1\1\GINC-ORC175\FOpt\RB3PW91\6-31G(d)\C2H3S3(1-)\SNIKOO\31-Jul-2017\0\
\# opt freq b3pw91/6-31g(d)\\CH2=CH-SSS(-)\\-1,1\C,1.3291699265,-0.401
6182946,0.1360649684\H,0.8958306479,-1.400924644,0.087646786\C,2.64510
79414,-0.2112962252,-0.0255597572\H,3.3093983176,-1.0549278111,-0.2022
923734\H,3.1004613607,0.7756558953,0.0240525037\S,0.1451960665,0.84952
56765,0.5024613699\S,-1.7159813588,-0.1022842236,-0.0773682795\S,-2.35
22721018,-1.4559936432,1.315634842\\Version=EM64L-G09RevD.01\State=1-A
\HF=-1272.4391174\RMSD=7.519e-09\RMSF=9.453e-06\Dipole=1.8617789,0.573
6023,-0.6970527\Quadrupole=-4.803065,2.2263766,2.5766884,-5.1952618,3.
146377,2.1354428\PG=C01 [X(C2H3S3)]\\@

1\1\GINC-ORC174\FOpt\RB3PW91\6-311G(d)\C2H3S3(1-)\SNIKOO\31-Jul-2017\0
\\# opt freq b3pw91/6-311g(d)\\CH2=CH-SSS(-)\\-1,1\C,1.333896042,-0.40
39102124,0.134433668\H,0.9026012134,-1.4016600287,0.0805596096\C,2.645
6804034,-0.2079612524,-0.0227254082\H,3.3120889043,-1.048274823,-0.199
4920921\H,3.0958875106,0.7801033016,0.0286976453\S,0.145183207,0.84021
21419,0.4971809843\S,-1.6999326097,-0.1383924876,-0.1064960722\S,-2.37
8493871,-1.4219799094,1.3484817252\\Version=EM64L-G09RevD.01\State=1-A
\HF=-1272.5356534\RMSD=8.240e-09\RMSF=6.931e-06\Dipole=2.0104555,0.664
583,-0.7783129\Quadrupole=-5.1524244,2.5328013,2.6196232,-5.3557703,3.
5007961,2.3559559\PG=C01 [X(C2H3S3)]\\@

1\1\GINC-ORC172\FOpt\RB3PW91\6-311+G(d)\C2H3S3(1-)\SNIKOO\31-Jul-2017\
0\\# opt freq b3pw91/6-311+g(d)\\CH2=CH-SSS(-)\\-1,1\C,1.3359324055,-0
.4014382482,0.0709144193\H,0.9078856893,-1.3907480169,-0.0810179747\C,
2.6581501593,-0.2056876865,0.004445911\H,3.3283002015,-1.0368172788,-0
.1994581668\H,3.1112779713,0.770530971,0.157861839\S,0.1318589947,0.82
49689193,0.431635349\S,-1.7184517653,-0.1896408726,-0.0779999409\S,-2.
3980428563,-1.3730310573,1.4542586242\\Version=EM64L-G09RevD.01\State=
1-A\HF=-1272.5417521\RMSD=5.844e-09\RMSF=1.232e-05\Dipole=1.8800511,0.
6063453,-0.8372607\Quadrupole=-5.3026967,2.9934639,2.3092329,-5.044524
6,4.0651567,2.765753\PG=C01 [X(C2H3S3)]\\@

1\1\GINC-ORC171\FOpt\RB3PW91\6-311+G(d,p)\C2H3S3(1-)\SNIKOO\31-Jul-201
7\0\\# opt freq b3pw91/6-311+g(d,p)\\CH2=CH-SSS(-)\\-1,1\C,1.335983334
1,-0.3996083629,0.0556049778\H,0.9055922678,-1.3840227074,-0.117297270
8\C,2.6596963935,-0.2063203681,0.0128092124\H,3.3278199423,-1.03679548
97,-0.1945584621\H,3.1103912321,0.7660583431,0.1886558716\S,0.12852740
33,0.8251341252,0.4090512677\S,-1.7205981381,-0.2033521351,-0.07412316
47\S,-2.390501635,-1.3629566751,1.4804976281\\Version=EM64L-G09RevD.01
\State=1-A\HF=-1272.5472651\RMSD=3.700e-09\RMSF=1.033e-05\Dipole=1.860
6584,0.602832,-0.8570008\Quadrupole=-5.3555387,3.0223088,2.3332299,-4.

9619785,4.1974152,2.8375606\PG=C01 [X(C2H3S3)]\\@

1\1\GINC-ORC169\FOpt\RB3PW91\6-311G(2d,p)\C2H3S3(1-)\SNIKOO\31-Jul-201
7\0\\# opt freq b3pw91/6-311g(2d,p)\\CH2=CH-SSS(-)\\-1,1\C,1.334267380
7,-0.413707646,0.1155765725\H,0.9050846211,-1.407720708,0.0210520067\C
,2.6440963761,-0.2067565736,-0.0125103318\H,3.3161905542,-1.037135693,
-0.2048466179\H,3.0852515437,0.7808343157,0.0791308828\S,0.1313505419,
0.8127988497,0.4880918717\S,-1.6925770671,-0.133939693,-0.0946935184\S
,-2.3667531506,-1.3962361218,1.3688391944\\Version=EM64L-G09RevD.01\St
ate=1-A\HF=-1272.5607242\RMSD=7.682e-09\RMSF=7.690e-06\Dipole=1.937191
7,0.6313538,-0.7563891\Quadrupole=-5.3373935,2.6228384,2.7145551,-5.00
86063,3.5468709,2.2868168\PG=C01 [X(C2H3S3)]\\@

1\1\GINC-ORC291\FOpt\RB3PW91\6-311G(df,p)\C2H3S3(1-)\SNIKOO\11-Sep-201
8\0\\# opt freq=noraman b3pw91/6-311g(df,p)\\CH2=CH-SSS(-)\\-1,1\C,1.7
755587224,0.5537796279,0.2200260757\H,1.1365836149,1.273532067,0.72681
97952\C,3.0928740976,0.7385083032,0.1310356747\H,3.5547274391,1.621918
5457,0.5612549751\H,3.7445634342,0.0315877383,-0.3736906694\S,0.875317
3627,-0.7892833742,-0.4687266528\S,-1.0043365095,-0.6448430928,0.56334
65595\S,-2.2224611615,0.7703331849,-0.2829507581\\Version=EM64L-G09Rev
E.01\State=1-A\HF=-1272.5526777\RMSD=8.723e-09\RMSF=7.868e-06\Dipole=2
.2428349,-0.2861417,0.3452349\Quadrupole=-8.5828133,3.9365148,4.646298
6,3.5793636,-1.0048917,1.0770682\PG=C01 [X(C2H3S3)]\\@

1\1\GINC-ORC167\FOpt\RB3PW91\6-311+G(df,p)\C2H3S3(1-)\SNIKOO\31-Jul-20
17\0\\# opt freq b3pw91/6-311+g(df,p)\\CH2=CH-SSS(-)\\-1,1\C,1.3336241
537,-0.4024804301,0.0465351915\H,0.9069454363,-1.385571686,-0.14226305
8\C,2.6547693307,-0.2069454061,0.0186743967\H,3.3272334166,-1.03253329
17,-0.1934279402\H,3.1023041401,0.7635759601,0.2113340107\S,0.12006301
19,0.8149374472,0.3985419336\S,-1.7127684577,-0.1987894086,-0.06594828
6\S,-2.3752602315,-1.3540564547,1.4871938117\\Version=EM64L-G09RevD.01
\State=1-A\HF=-1272.5588397\RMSD=4.287e-09\RMSF=2.240e-05\Dipole=1.884
4321,0.6234425,-0.8860911\Quadrupole=-5.323992,3.0466877,2.2773043,-5.
0387362,4.2734705,2.9689277\PG=C01 [X(C2H3S3)]\\@

1\1\GINC-ORC165\FOpt\RB3PW91\6-311+G(2df,p)\C2H3S3(1-)\SNIKOO\31-Jul-2
017\0\\# opt freq b3pw91/6-311+g(2df,p)\\CH2=CH-SSS(-)\\-1,1\C,1.32816
46128,-0.4118312075,0.0799773243\H,0.9035837544,-1.4011762281,-0.07349
40438\C,2.6451422508,-0.2066548277,0.0063144692\H,3.3195934027,-1.0308
989303,-0.2024320745\H,3.0882452293,0.7725048203,0.1597131201\S,0.1189
1646,0.7989745151,0.4483235527\S,-1.6954205629,-0.1565674455,-0.076167
6116\S,-2.3513143471,-1.3662139662,1.4184053235\\Version=EM64L-G09RevD
.01\State=1-A\HF=-1272.5762995\RMSD=7.762e-09\RMSF=1.548e-05\Dipole=1.
8215713,0.6196554,-0.7852892\Quadrupole=-5.4276326,2.9375151,2.4901175
,-4.854525,3.8543035,2.5849132\PG=C01 [X(C2H3S3)]\\@

1\1\GINC-ORC163\FOpt\RB3PW91\6-311++G(3df,3pd)\C2H3S3(1-)\SNIKOO\31-Ju
l-2017\0\\# opt freq b3pw91/6-311++g(3df,3pd)\\CH2=CH-SSS(-)\\-1,1\C,1
.3257439838,-0.412323193,0.0701063942\H,0.9041388992,-1.3992153569,-0.
0980001936\C,2.6437358638,-0.2069421864,0.0123099527\H,3.3187903939,-1
.0283238423,-0.1981678425\H,3.0843050042,0.7692482384,0.1807622817\S,0
.1152001131,0.7937803255,0.4328185771\S,-1.6938196544,-0.1595533038,-0
.0686346086\S,-2.3411838037,-1.3585339514,1.4294454989\\Version=EM64L-
G09RevD.01\State=1-A\HF=-1272.5807655\RMSD=4.339e-09\RMSF=1.502e-05\Di
pole=1.7869044,0.5933587,-0.7568801\Quadrupole=-5.3971406,2.906074,2.4
910666,-4.6787262,3.794569,2.5649271\PG=C01 [X(C2H3S3)]\\@

CH_2CHSSS^- wB97XD

```
1\1\GINC-ORC63\FOpt\RwB97XD\6-31G(d)\C2H3S3(1-)\SNIKOO\31-Jul-2017\0\\
 # OPT freq rwb97xd/6-31g(d)\\CH2=CH-SSS(-)\\-1,1\C,1.7042151213,0.5943
 141803,0.1757861684\H,1.0066931422,1.3647876064,0.504424384\C,3.024046
 027,0.7922804696,0.2003880264\H,3.4352563196,1.7352556344,0.5520206875
 \H,3.7308747275,0.0364901634,-0.134087163\S,0.9022060785,-0.8430610461
 ,-0.4546720614\S,-0.9970164737,-0.7322954314,0.5163632158\S,-2.0653788
 224,0.8546471435,-0.2295352577\\Version=EM64L-G09RevD.01\State=1-A\HF=
 -1272.5291457\RMSD=7.555e-09\RMSF=5.067e-05\Dipole=2.1312881,-0.261609
 1,0.2959171\Quadrupole=-7.0137904,3.443512,3.5702784,3.7030898,-0.7194
 694,0.812364\PG=C01 [X(C2H3S3)]\\@
```

```
1\1\GINC-ORC63\FOpt\RwB97XD\6-311G(d)\C2H3S3(1-)\SNIKOO\31-Jul-2017\0\
 \# opt freq rwb97xd/6-311g(d)\\CH2=CH-SSS(-)\\-1,1\C,1.7080002527,0.60
 05162139,0.1723600364\H,1.0153801118,1.3701487076,0.5065000892\C,3.025
 4881118,0.7885343595,0.1988336986\H,3.4416750826,1.7259573121,0.556150
 6449\H,3.7266788718,0.0293023953,-0.1370478341\S,0.8980607212,-0.82734
 65424,-0.4655674879\S,-0.9816719207,-0.7167688043,0.549968619\S,-2.092
 7151111,0.8320750782,-0.250509766\\Version=EM64L-G09RevD.01\State=1-A\
 HF=-1272.626161\RMSD=3.705e-09\RMSF=4.548e-05\Dipole=2.3392956,-0.3423
 24,0.3217558\Quadrupole=-7.5370143,3.7536395,3.7833748,3.8763797,-0.90
 72544,0.9045128\PG=C01 [X(C2H3S3)]\\@
```

```
1\1\GINC-ORC62\FOpt\RwB97XD\6-311+G(d)\C2H3S3(1-)\SNIKOO\31-Jul-2017\0
 \\# opt freq rwb97xd/6-311+g(d)\\CH2=CH-SSS(-)\\-1,1\C,1.7184070234,0.
 5969174951,0.2039727933\H,1.0403509385,1.3594973194,0.5816718125\C,3.0
 377182497,0.7903964325,0.1840144826\H,3.4631190795,1.7214868093,0.5469
 781108\H,3.7288805826,0.0426131993,-0.1960025916\S,0.8915541057,-0.823
 2163941,-0.4230085545\S,-1.0020672885,-0.6827155787,0.5569079728\S,-2.
 1370665709,0.7974394371,-0.323846026\\Version=EM64L-G09RevD.01\State=1
 -A\HF=-1272.632059\RMSD=8.183e-09\RMSF=8.498e-06\Dipole=2.2452584,-0.3
 300177,0.3769292\Quadrupole=-7.7185072,3.928951,3.7895562,3.682473,-1.
 4263121,1.1494495\PG=C01 [X(C2H3S3)]\\@
```

```
1\1\GINC-ORC60\FOpt\RwB97XD\6-311+G(d,p)\C2H3S3(1-)\SNIKOO\31-Jul-2017
 \0\\# opt freq rwb97xd/6-311+g(d,p)\\CH2=CH-SSS(-)\\-1,1\C,1.731719859
 8,0.5812728134,0.2620434755\H,1.070450067,1.3135473898,0.7190359391\C,
 3.0434349923,0.7994451925,0.1570996186\H,3.4763486169,1.7217617511,0.5
 29835419\H,3.7134865452,0.0820931656,-0.306962486\S,0.8880660314,-0.83
 71874393,-0.3456834732\S,-1.0319787149,-0.6324789536,0.5636108287\S,-2
 .1506312777,0.7739648006,-0.4482913217\\Version=EM64L-G09RevD.01\State
 =1-A\HF=-1272.637313\RMSD=5.077e-09\RMSF=1.592e-05\Dipole=2.2353455,-0
 .3224194,0.4870227\Quadrupole=-7.9347001,3.9443101,3.99039,3.5754287,-
 2.1331813,1.4333234\PG=C01 [X(C2H3S3)]\\@
```

```
1\1\GINC-ORC60\FOpt\RwB97XD\6-311G(2d,p)\C2H3S3(1-)\SNIKOO\31-Jul-2017
 \0\\# opt freq rwb97xd/6-311g(2d,p)\\CH2=CH-SSS(-)\\-1,1\C,1.715062907
 5,0.6058313502,0.1925096178\H,1.0330388523,1.368867185,0.5573689527\C,
 3.0304324414,0.7878408111,0.1894098017\H,3.4574397263,1.7166817633,0.5
 517563199\H,3.7170302128,0.0307974433,-0.1755816244\S,0.8814017357,-0.
 8096933213,-0.4393010661\S,-0.9826361665,-0.6936846617,0.5541414722\S,
 -2.1108735895,0.7957781502,-0.2996154737\\Version=EM64L-G09RevD.01\Sta
 te=1-A\HF=-1272.651055\RMSD=8.182e-09\RMSF=5.735e-05\Dipole=2.2325495,
 -0.2796683,0.3328216\Quadrupole=-7.6915958,3.8039782,3.8876176,3.38278
 48,-1.1378608,0.8672516\PG=C01 [X(C2H3S3)]\\@
```

```
1\1\GINC-ORC289\FOpt\RwB97XD\6-311G(df,p)\C2H3S3(1-)\SNIKOO\11-Sep-201
 8\0\\# opt freq=noraman wb97xd/6-311g(df,p)\\CH2=CH-SSS(-)\\-1,1\C,1.7
 397047412,0.5580574111,0.1784136143\H,1.0712090856,1.3032982446,0.6032
 148277\C,3.0558514637,0.7373689859,0.149181996\H,3.4963749582,1.643001
 7304,0.5527496062\H,3.730266175,0.0016799146,-0.2780887964\S,0.8846859
 485,-0.8167691738,-0.5105097168\S,-0.9598413141,-0.7325338677,0.537996
 3745\S,-2.065424058,0.8614297549,-0.1558429055\\Version=EM64L-G09RevE.
 01\State=1-A\HF=-1272.6432899\RMSD=9.996e-09\RMSF=1.678e-05\Dipole=2.3
 378865,-0.4054995,0.2554749\Quadrupole=-7.6411712,3.4721727,4.1689986,
 4.1464673,-0.4230601,0.8424761\PG=C01 [X(C2H3S3)]\\@

1\1\GINC-ORC58\FOpt\RwB97XD\6-311+G(df,p)\C2H3S3(1-)\SNIKOO\31-Jul-201
 7\0\\# opt freq rwb97xd/6-311+g(df,p)\\CH2=CH-SSS(-)\\-1,1\C,1.7321271
 601,0.579912735,0.2725975053\H,1.0765035028,1.3083790447,0.7434385513\
 C,3.0396308581,0.8005210467,0.152642805\H,3.4774532772,1.7198186267,0.
 5266945781\H,3.7047404188,0.0880230284,-0.3255444516\S,0.8822500354,-0
 .8357660974,-0.3284005137\S,-1.0308563103,-0.626830727,0.5555983551\S,
 -2.1409528221,0.768361063,-0.4663388295\\Version=EM64L-G09RevD.01\Stat
 e=1-A\HF=-1272.6492925\RMSD=5.631e-09\RMSF=1.168e-05\Dipole=2.2400454,
 -0.3276735,0.5100058\Quadrupole=-7.9780443,3.9346017,4.0434426,3.64319
 89,-2.2255353,1.5401121\PG=C01 [X(C2H3S3)]\\@
1\1\GINC-ORC57\FOpt\RwB97XD\6-311+G(2df,p)\C2H3S3(1-)\SNIKOO\31-Jul-20
 17\0\\# opt freq rwb97xd/6-311+g(2df,p)\\CH2=CH-SSS(-)\\-1,1\C,1.72401
 50487,0.5897784536,0.2542143445\H,1.0665107702,1.329107303,0.703364220
 1\C,3.0352206672,0.7951160137,0.1608959318\H,3.4748236194,1.7145883474
 ,0.5309565448\H,3.7024945618,0.0693072866,-0.2927156365\S,0.8707527817
 ,-0.8169061246,-0.3508405958\S,-1.0132821353,-0.6399189471,0.554899875
 2\S,-2.1196391938,0.7613463874,-0.4300866841\\Version=EM64L-G09RevD.01
 \State=1-A\HF=-1272.666796\RMSD=8.468e-09\RMSF=2.444e-05\Dipole=2.1399
 326,-0.2962654,0.4343112\Quadrupole=-7.852891,3.8982788,3.9546122,3.24
 59709,-1.9408445,1.3186944\PG=C01 [X(C2H3S3)]\\@

1\1\GINC-ORC57\FOpt\RwB97XD\6-311++G(3df,3pd)\C2H3S3(1-)\SNIKOO\31-Jul
 -2017\0\\# opt freq rwb97xd/6-311++g(3df,3pd)\\CH2=CH-SSS(-)\\-1,1\C,1
 .7258675345,0.5871472397,0.2686479434\H,1.0775198166,1.3205201308,0.73
 68570392\C,3.0350026859,0.7966402854,0.1538596992\H,3.4777433068,1.712
 1373713,0.5248162898\H,3.6939989293,0.0778429201,-0.3183441728\S,0.867
 9360183,-0.8159745667,-0.3270059934\S,-1.0177402951,-0.6262470323,0.55
 10817398\S,-2.1194318762,0.7503523716,-0.4592245452\\Version=EM64L-G09
 RevD.01\State=1-A\HF=-1272.6714337\RMSD=6.132e-09\RMSF=2.311e-05\Dipol
 e=2.0953327,-0.2600089,0.4268669\Quadrupole=-7.7581511,3.8188322,3.939
 3189,3.0369833,-1.999658,1.3375019\PG=C01 [X(C2H3S3)]\\@
```

CH_2CHSSS^- M062X

```
1\1\GINC-ORC183\FOpt\RM062X\6-31G(d)\C2H3S3(1-)\SNIKOO\31-Jul-2017\0\\
 # opt freq m062x/6-31g(d)\\CH2=CH-SSS(-)\\-1,1\C,1.2738874682,-0.38058
 22185,0.210499062\H,0.8061442705,-1.3617121906,0.3134762149\C,2.579827
 4257,-0.2383832929,-0.0214957988\H,3.223608989,-1.108454109,-0.1150592
 421\H,3.0454337487,0.7389686044,-0.117239874\S,0.1446733146,0.94091795
 35,0.4845537543\S,-1.685018168,0.0012286318,-0.119855966\S,-2.03164624
 87,-1.5938466489,1.1257619098\\Version=EM64L-G09RevD.01\State=1-A\HF=-
 1272.4683578\RMSD=6.148e-09\RMSF=4.639e-05\Dipole=1.7756631,0.7610655,
 -0.5970107\Quadrupole=-3.1047634,0.83171,2.2730534,-5.2331951,2.311250
 2,1.7413386\PG=C01 [X(C2H3S3)]\\@
1\1\GINC-ORC185\FOpt\RM062X\6-311G(d)\C2H3S3(1-)\SNIKOO\31-Jul-2017\0\
 \# opt freq m062x/6-311g(d)\\CH2=CH-SSS(-)\\-1,1\C,1.2781258033,-0.386
```

3161576,0.2216455021\H,0.8167847984,-1.3671757585,0.3255708023\C,2.576
8319357,-0.2359810551,-0.0266165103\H,3.2217865941,-1.1021202625,-0.13
22436197\H,3.0338527687,0.7434443252,-0.1273732016\S,0.1477767853,0.92
99129075,0.5131159972\S,-1.6623956926,-0.007306191,-0.1503222091\S,-2.
0558521928,-1.576321078,1.1368632992\\Version=EM64L-G09RevD.01\State=1
-A\HF=-1272.5815461\RMSD=7.265e-09\RMSF=2.465e-05\Dipole=1.9611878,0.8
977339,-0.673639\Quadrupole=-3.252499,0.9674348,2.2850642,-5.6177927,2
.5121591,1.8554344\PG=C01 [X(C2H3S3)]\\@

1\1\GINC-ORC225\FOpt\RM062X\6-311+G(d)\C2H3S3(1-)\SNIKOO\08-Sep-2017\0
\\# opt=calcfc freq=noraman 6-311+g(d) m062x nosymm\\CH2=CH-SSS(-)\\-1
,1\C,1.6010161818,0.6271609274,0.1234810692\H,0.8658385613,1.399904660
8,0.3442667071\C,2.9115114805,0.8509235981,0.223716744\H,3.282150026,1
.8230575888,0.531812458\H,3.6458107047,0.0835233079,-0.0007361434\S,0.
8735272766,-0.8643365494,-0.4524658114\S,-1.0282927703,-0.7444979867,0
.5274305205\S,-2.0569484604,0.8546064531,-0.272072544\\Version=EM64L-G
09RevD.01\HF=-1272.587636\RMSD=7.249e-09\RMSF=2.068e-05\Dipole=2.09187
14,-0.3528666,0.298892\Quadrupole=-6.7748345,3.5955154,3.1793191,3.655
3724,-0.9841254,0.7858562\PG=C01 [X(C2H3S3)]\\@

1\1\GINC-ORC213\FOpt\RM062X\6-311+G(d,p)\C2H3S3(1-)\SNIKOO\08-Sep-2017
\0\\# opt=calcfc freq=noraman m062x/6-311+g(d,p) nosymm\\CH2=CH-SSS(-)
\\-1,1\C,1.6015235177,0.6245091675,0.1302630686\H,0.8637578024,1.39249
64487,0.3584032061\C,2.9120939774,0.8524207247,0.2213601229\H,3.278675
1593,1.8247526074,0.5305530607\H,3.6448459534,0.0876481485,-0.01270994
99\S,0.8757410949,-0.8680044712,-0.4447312469\S,-1.0318731774,-0.74338
36144,0.5232435421\S,-2.0501513277,0.8599029888,-0.2809488035\\Version
=EM64L-G09RevD.01\HF=-1272.5918727\RMSD=5.269e-09\RMSF=2.088e-05\Dipol
e=2.0762649,-0.3611532,0.304989\Quadrupole=-6.7985614,3.5540889,3.2444
724,3.640247,-1.0529599,0.7890302\PG=C01 [X(C2H3S3)]\\@

1\1\GINC-ORC174\FOpt\RM062X\6-311G(2d,p)\C2H3S3(1-)\SNIKOO\31-Jul-2017
\0\\# opt freq m062x/6-311g(2d,p)\\CH2=CH-SSS(-)\\-1,1\C,1.2905182524,
-0.4012680765,0.1812670734\H,0.837732467,-1.3887133691,0.2192911046\C,
2.5923946649,-0.2255761595,-0.0105995931\H,3.2504794741,-1.0780003828,
-0.1302149599\H,3.036549757,0.7625233248,-0.0448382273\S,0.1301972152,
0.8866490198,0.4799888651\S,-1.6590493467,-0.0514904133,-0.1552854082\
S,-2.1219116837,-1.5059872133,1.2210312055\\Version=EM64L-G09RevD.01\S
tate=1-A\HF=-1272.6051395\RMSD=6.586e-09\RMSF=6.531e-05\Dipole=1.92481
44,0.7806428,-0.6907721\Quadrupole=-3.8553537,1.5438367,2.311517,-5.21
87088,2.8910837,1.9818531\PG=C01 [X(C2H3S3)]\\@

1\1\GINC-ORC285\FOpt\RM062X\6-311G(df,p)\C2H3S3(1-)\SNIKOO\11-Sep-2018
\0\\# opt freq=noraman m062x/6-311g(df,p)\\CH2=CH-SSS(-)\\-1,1\C,1.710
7757202,0.5610912694,0.127462477\H,1.0134374051,1.3197185581,0.4787177
353\C,3.0269619175,0.7360136034,0.1684266645\H,3.4479469532,1.65624864
61,0.5579890416\H,3.7176362601,-0.0216244502,-0.1861827208\S,0.8978371
05,-0.8362274804,-0.5626825774\S,-0.923080625,-0.7805997084,0.52809545
64\S,-1.9386877362,0.920912562,-0.0347110764\\Version=EM64L-G09RevE.01
\State=1-A\HF=-1272.5983976\RMSD=7.661e-09\RMSF=3.138e-05\Dipole=2.228
8181,-0.4485812,0.1401858\Quadrupole=-6.7602181,3.0639181,3.6963,4.239
9967,0.2024232,0.5255283\PG=C01 [X(C2H3S3)]\\@

1\1\GINC-ORC172\FOpt\RM062X\6-311+G(df,p)\C2H3S3(1-)\SNIKOO\31-Jul-201
7\0\\# opt freq m062x/6-311+g(df,p)\\CH2=CH-SSS(-)\\-1,1\C,1.286805078
6,-0.3951108354,0.1584800723\H,0.8331802873,-1.3846140825,0.163728347\
C,2.5966476915,-0.2251900144,-0.0059489713\H,3.2493341867,-1.080788372

8,-0.1391903237\H,3.052613181,0.759381658,-0.0025971066\S,0.1323314195
,0.8900943577,0.466154319\S,-1.6740664613,-0.0480921663,-0.1225020877\
S,-2.1199345834,-1.5175438143,1.2425158109\\Version=EM64L-G09RevD.01\S
tate=1-A\HF=-1272.6046237\RMSD=6.256e-09\RMSF=5.126e-05\Dipole=1.87208
23,0.8367745,-0.7487682\Quadrupole=-3.659987,1.543548,2.116439,-5.4256
392,3.0464545,2.3647524\PG=C01 [X(C2H3S3)]\\@

1\1\GINC-ORC171\FOpt\RM062X\6-311+G(2df,p)\C2H3S3(1-)\SNIKOO\31-Jul-20
17\0\\# opt freq m062x/6-311+g(2df,p)\\CH2=CH-SSS(-)\\-1,1\C,1.2881529
517,-0.4023562904,0.1547764316\H,0.8414553181,-1.3939352396,0.15116767
74\C,2.5971319855,-0.2219839568,0.0017260291\H,3.2580155172,-1.0707258
537,-0.1281350049\H,3.0439751122,0.7659217676,0.0114516039\S,0.1210047
046,0.8691750124,0.4523056534\S,-1.6633776154,-0.0674079983,-0.1386739
376\S,-2.1294471739,-1.4805507111,1.2560216072\\Version=EM64L-G09RevD.
01\State=1-A\HF=-1272.6214509\RMSD=7.249e-09\RMSF=2.150e-05\Dipole=1.8
121173,0.7643457,-0.7062728\Quadrupole=-3.9529028,1.8416175,2.1112853,
-5.0720908,3.0570542,2.2083095\PG=C01 [X(C2H3S3)]\\@

1\1\GINC-ORC169\FOpt\RM062X\6-311++G(3df,3pd)\C2H3S3(1-)\SNIKOO\31-Jul
-2017\0\\# opt freq m062x/6-311++g(3df,3pd)\\CH2=CH-SSS(-)\\-1,1\C,1.2
88442811,-0.4037004864,0.1480525432\H,0.8482145822,-1.3962639849,0.144
1229312\C,2.5991345571,-0.2191440253,0.012182146\H,3.2637797757,-1.064
7459922,-0.1031370829\H,3.0407503435,0.7690976772,0.0224404612\S,0.113
761094,0.862291424,0.4200721192\S,-1.661859323,-0.085207437,-0.1494854
227\S,-2.1353130405,-1.4641904454,1.2663923649\\Version=EM64L-G09RevD.
01\State=1-A\HF=-1272.6263761\RMSD=4.420e-09\RMSF=1.689e-05\Dipole=1.7
813493,0.7200431,-0.675733\Quadrupole=-4.0277806,1.906922,2.1208585,-4
.8530953,3.0254171,2.1570876\PG=C01 [X(C2H3S3)]\\@

CH_2CHSSS^- M08HX

1\1\GINC-ORC46\FOpt\RM08HX\6-31G(d)\C2H3S3(1-)\SNIKOO\31-Jul-2017\0\\#
opt freq 6-31g(d) m08hx\\CH2=CH-SSS(-)\\-1,1\C,1.6119646558,0.5565361
291,0.1006439445\H,0.9339071302,1.175867906,0.7024506973\C,2.92319286,
0.7990302242,0.0386655721\H,3.3618440304,1.6288694746,0.5947287095\H,3
.5980475392,0.1928834677,-0.5684131344\S,0.7635587376,-0.681392598,-0.
8180860508\S,-0.9459085423,-0.9517241377,0.4475117337\S,-1.9298274109,
0.8469265342,0.550526528\\Version=EM64L-G16RevA.03\State=1-A\HF=-1272.
4717029\RMSD=7.528e-09\RMSF=4.144e-05\Dipole=1.9648049,-0.3051215,-0.2
439183\Quadrupole=-5.6959181,2.8294799,2.8664382,3.3326583,2.4010858,-
0.1267542\PG=C01 [X(C2H3S3)]\\@

1\1\GINC-ORC45\FOpt\RM08HX\6-311G(d)\C2H3S3(1-)\SNIKOO\31-Jul-2017\0\\
opt freq 6-311g(d) m08hx\\CH2=CH-SSS(-)\\-1,1\C,1.6083281048,0.55883
68212,0.0926083755\H,0.9252396312,1.1893312474,0.6716354765\C,2.916230
4082,0.8009355788,0.0324549795\H,3.347971624,1.6455868783,0.568997899\
H,3.5944470544,0.1789839539,-0.5524817138\S,0.7698655805,-0.7036373947
,-0.7986131583\S,-0.9239643868,-0.9552910019,0.4984505149\S,-1.9213390
163,0.8522509169,0.5349756267\\Version=EM64L-G16RevA.03\State=1-A\HF=-
1272.5946525\RMSD=3.712e-09\RMSF=4.119e-05\Dipole=2.1082974,-0.4136376
,-0.2886467\Quadrupole=-5.9911669,3.0045593,2.9866076,3.6767431,2.3918
148,-0.1371898\PG=C01 [X(C2H3S3)]\\@

1\1\GINC-ORC44\FOpt\RM08HX\6-311+G(d)\C2H3S3(1-)\SNIKOO\31-Jul-2017\0\
\# opt freq 6-311+g(d) m08hx\\CH2=CH-SSS(-)\\-1,1\C,1.6126546669,0.553
5844521,0.0993822844\H,0.9369538917,1.1853287268,0.686283201\C,2.92036

23995,0.8056262289,0.0192934033\H,3.3525999621,1.6562411628,0.54617190
3\H,3.5958511983,0.1877055899,-0.5732888918\S,0.7716169726,-0.71968532
86,-0.7680737915\S,-0.9288709274,-0.9478578235,0.5235475964\S,-1.94438
91637,0.8460539918,0.5147122952\\Version=EM64L-G16RevA.03\State=1-A\HF
=-1272.6010404\RMSD=4.099e-09\RMSF=2.078e-05\Dipole=1.9821843,-0.41362
26,-0.2521363\Quadrupole=-6.0751347,2.9987017,3.0764329,3.5748849,2.11
19823,0.0202709\PG=C01 [X(C2H3S3)]\\@

1\1\GINC-ORC41\FOpt\RM08HX\6-311+G(d,p)\C2H3S3(1-)\SNIKOO\31-Jul-2017\
0\\# opt freq 6-311+g(d,p) m08hx\\CH2=CH-SSS(-)\\-1,1\C,1.6106491243,0
.5489264493,0.0983768779\H,0.9282953971,1.1790907338,0.6790977156\C,2.
916451791,0.8100517249,0.0146222156\H,3.3397290138,1.6676324683,0.5349
184983\H,3.5931876818,0.1918578941,-0.574060511\S,0.7787011532,-0.7377
585803,-0.7568087046\S,-0.9298361182,-0.9501586541,0.5296358239\S,-1.9
20399043,0.857354964,0.5222460843\\Version=EM64L-G16RevA.03\State=1-A\
HF=-1272.6056297\RMSD=6.031e-09\RMSF=3.806e-05\Dipole=1.9478576,-0.426
9015,-0.2573085\Quadrupole=-6.0275318,2.9507495,3.0767822,3.5549357,2.
0944033,-0.0557294\PG=C01 [X(C2H3S3)]\\@

1\1\GINC-ORC39\FOpt\RM08HX\6-311G(2d,p)\C2H3S3(1-)\SNIKOO\31-Jul-2017\
0\\# opt freq 6-311g(2d,p) m08hx\\CH2=CH-SSS(-)\\-1,1\C,1.6186803433,0
.5604624536,0.1109705944\H,0.9498799865,1.174147416,0.7198892596\C,2.9
215308214,0.8024004312,0.0233511116\H,3.3661078994,1.6341763449,0.5651
706828\H,3.5799327331,0.1916588758,-0.5911511282\S,0.7545870277,-0.684
5423611,-0.7781818915\S,-0.9139823553,-0.9401006284,0.512778455\S,-1.9
599574561,0.828794468,0.4852009163\\Version=EM64L-G16RevA.03\State=1-A
\HF=-1272.6181543\RMSD=7.456e-09\RMSF=1.515e-05\Dipole=2.0402652,-0.34
52584,-0.2392316\Quadrupole=-6.3512847,3.1158905,3.2353942,3.2869865,2
.1202793,-0.0911021\PG=C01 [X(C2H3S3)]\\@

1\1\GINC-ORC39\FOpt\RM08HX\6-311+G(df,p)\C2H3S3(1-)\SNIKOO\31-Jul-2017
\0\\# opt freq 6-311+g(df,p) m08hx\\CH2=CH-SSS(-)\\-1,1\C,1.6159634192
,0.5476563216,0.1105682158\H,0.9449981994,1.165381411,0.716916609\C,2.
9181773344,0.8088707973,0.0140951951\H,3.3517772441,1.6529136784,0.547
9441397\H,3.5859455251,0.2046198741,-0.5989183836\S,0.7673650381,-0.71
80741238,-0.7566374937\S,-0.928875896,-0.9411337944,0.5113928593\S,-1.
9385718643,0.8467628358,0.5026668584\\Version=EM64L-G16RevA.03\State=1
-A\HF=-1272.6185646\RMSD=5.324e-09\RMSF=2.971e-05\Dipole=1.9771171,-0.
4295875,-0.2451662\Quadrupole=-6.1846818,2.8721457,3.3125361,3.6096728
,2.1531102,-0.017946\PG=C01 [X(C2H3S3)]\\@

1\1\GINC-ORC38\FOpt\RM08HX\6-311+G(2df,p)\C2H3S3(1-)\SNIKOO\31-Jul-201
7\0\\# opt freq 6-311+g(2df,p) m08hx\\CH2=CH-SSS(-)\\-1,1\C,1.62107589
47,0.5514069278,0.1264932217\H,0.9681488611,1.1427245287,0.7748535621\
C,2.9242701407,0.8005687271,0.0245947564\H,3.3781451043,1.6094211693,0
.5930191855\H,3.5727955467,0.2194800544,-0.6285871447\S,0.7437624261,-
0.6552986338,-0.7874354986\S,-0.9250366527,-0.927220648,0.4712003588\S
,-1.9663823208,0.8259148745,0.4738895587\\Version=EM64L-G16RevA.03\Sta
te=1-A\HF=-1272.6350778\RMSD=9.500e-09\RMSF=2.712e-05\Dipole=1.9228383
,-0.3715242,-0.2289214\Quadrupole=-6.4026735,2.9724591,3.4302145,3.187
7005,2.0548968,0.0689803\PG=C01 [X(C2H3S3)]\\@

1\1\GINC-ORC38\FOpt\RM08HX\6-311++G(3df,3pd)\C2H3S3(1-)\SNIKOO\31-Jul-
2017\0\\# opt freq 6-311++g(3df,3pd) m08hx\\CH2=CH-SSS(-)\\-1,1\C,1.61
9394583,0.5512093879,0.1262053662\H,0.9672162912,1.1425234908,0.771806
8351\C,2.9229330258,0.8004383828,0.0243743458\H,3.3746167633,1.6090346

749,0.5899376749\H,3.5691903904,0.2191754251,-0.6263896376\S,0.7425331
882,-0.6544470685,-0.7846107126\S,-0.9214275365,-0.9242332023,0.468729
559\S,-1.9576777054,0.8232959093,0.4779745692\\Version=EM64L-G16RevA.0
3\State=1-A\HF=-1272.641342\RMSD=3.793e-09\RMSF=1.281e-05\Dipole=1.865
2244,-0.3240509,-0.2281517\Quadrupole=-6.2031401,2.8731687,3.3299715,2
.9621661,2.0428633,0.0358599\PG=C01 [X(C2H3S3)]\\@

CH_2CHSSS^- QCISD

1\1\GINC-ORC292\FOpt\RQCISD-FC\6-311+G(2df,p)\C2H3S3(1-)\SNIKOO\21-Aug
-2017\0\\# opt freq=noraman 6-311+g(2df,p) qcisd\\CH2=CH-SSS(-)\\-1,1\
C,1.675525,0.597973,0.170149\H,0.997938,1.363528,0.54247\C,3.000734,0.
771804,0.185508\H,3.431705,1.692957,0.565924\H,3.686329,0.015516,-0.18
5524\S,0.843005,-0.806634,-0.489216\S,-0.989646,-0.713592,0.546587\S,-
2.114205,0.814559,-0.248421\\Version=EM64L-G09RevD.01\State=1-A\HF=-12
70.1507344\MP2=-1270.985227\MP3=-1271.0348696\MP4D=-1271.0580058\MP4DQ
=-1271.0298107\MP4SDQ=-1271.0379421\QCISD=-1271.0381756\RMSD=4.677e-09
\RMSF=3.833e-05\Dipole=2.1669051,-0.3158763,0.2782074\PG=C01 [X(C2H3S3
)]\\@

$CH_2CHSSS^{\bullet}$ B3LYP

1\1\GINC-ORC327\FOpt\UB3LYP\6-31G(d)\C2H3S3(2)\SNIKOO\19-Aug-2017\0\\#
 opt freq ub3lyp/6-31g(d)\\CH2=CH-SSS\\0,2\C,1.7034848424,0.6888953748
,-0.0498411434\H,1.0802426914,1.5727252052,0.0662776473\C,3.0040657182
,0.6861574218,0.2500071726\H,3.4842973268,1.5916351596,0.6119056196\H,
3.6279153018,-0.1946253178,0.129047409\S,0.9079619184,-0.7082579178,-0
.8048796491\S,-0.8894523147,-0.9238121911,0.377229311\S,-1.8685234843,
0.7863842652,0.5174246331\\Version=EM64L-G09RevD.01\State=2-A\HF=-1272
.5454489\S2=0.752937\S2-1=0.\S2A=0.750007\RMSD=4.214e-09\RMSF=1.248e-0
5\Dipole=0.7551115,0.1775801,0.1033955\Quadrupole=1.7348256,0.4481448,
-2.1829704,1.7318189,1.1128718,0.478299\PG=C01 [X(C2H3S3)]\\@

1\1\GINC-ORC333\FOpt\UB3LYP\6-311G(2d,p)\C2H3S3(2)\SNIKOO\28-Aug-2017\
0\\# opt=calcfc freq=noraman b3lyp/6-311g(2d,p)\\CH2=CH-SSS n1\\0,2\C,
1.5754917788,0.6608938043,-0.0817620212\H,0.9419677108,1.5022224479,-0
.3379533179\C,2.8164546398,0.8096988492,0.3635578111\H,3.2400661131,1.
8012254266,0.4724837334\H,3.4475343875,-0.0335206484,0.6151186554\S,0.
8853209771,-0.9304404471,-0.4346473924\S,-1.101781747,-0.7766393172,0.
3941272612\S,-1.9471378601,0.9311268847,-0.0899457296\\Version=EM64L-G
09RevD.01\State=2-A\HF=-1272.6647371\S2=0.754342\S2-1=0.\S2A=0.750015\
RMSD=7.183e-09\RMSF=5.878e-06\Dipole=0.6971445,0.1419847,0.1209104\Qua
drupole=1.122678,0.9637032,-2.0863813,1.8575657,0.8581614,-0.1915084\P
G=C01 [X(C2H3S3)]\\@

1\1\GINC-ORC32\FOpt\UB3LYP\6-311+G(2df,p)\C2H3S3(2)\SNIKOO\19-Aug-2017
\0\\# opt freq ub3lyp/6-311+g(2df,p)\\Title Card Required\\0,2\C,1.530
4921558,0.6088137022,0.0529639524\H,0.7524007981,1.3664533523,0.000735
5028\C,2.822262759,0.9242064237,0.1057747491\H,3.1200646673,1.96501599
42,0.0970817485\H,3.6106315133,0.1838538709,0.1581378303\S,0.931483837
7,-1.0331839617,0.0609654649\S,-1.2127063175,-0.8037024875,-0.04513230
16\S,-1.7807454137,1.0542341058,-0.1114349464\\Version=EM64L-G09RevD.0
1\State=2-A\HF=-1272.6808861\S2=0.755565\S2-1=0.\S2A=0.750027\RMSD=7.5
98e-09\RMSF=8.506e-06\Dipole=0.5358175,-0.0088835,0.0251362\Quadrupole
=1.1946379,1.1775395,-2.3721775,1.6044614,0.1310949,-0.0015275\PG=C01
[X(C2H3S3)]\\@

1\1\GINC-ORC113\FOpt\UB3LYP\6-311++G(3df,3pd)\C2H3S3(2)\SNIKOO\19-Aug-
2017\0\\# opt freq ub3lyp/6-311++g(3df,3pd)\\CH2=CH-SSS\\0,2\C,1.69267
89537,0.4980295807,0.1461979914\H,0.958767358,1.2309692632,0.467622854
9\C,3.0001608409,0.7454517004,0.1819696761\H,3.3532925627,1.7021878663
,0.5402064586\H,3.7455308361,0.0288647945,-0.1336051397\S,1.0064794595
,-1.0050815751,-0.410594317\S,-1.1153648734,-0.7208705613,-0.199309949
9\S,-1.5915531375,1.0195509312,0.5046834255\\Version=EM64L-G09RevD.01\
State=2-A\HF=-1272.6861731\S2=0.756021\S2-1=0.\S2A=0.750031\RMSD=9.918
e-09\RMSF=7.429e-06\Dipole=0.5442014,-0.0104797,-0.0296294\Quadrupole=
1.3993925,0.5813672,-1.9807597,1.4133412,0.3758898,1.0989202\PG=C01 [X
(C2H3S3)]\\@

$CH_2CHSSS^{\bullet}$ B3PW91

1\1\GINC-ORC6\FOpt\UB3PW91\6-31G(d)\C2H3S3(2)\SNIKOO\19-Aug-2017\0\\#
OPT freq ub3pw91/6-31g(d)\\CH2=CH-SSS\\0,2\C,1.692636007,0.6714072897,
-0.0302310465\H,1.0555925294,1.5427743335,0.1115542056\C,2.9979746749,
0.6879922495,0.2445278543\H,3.4657956824,1.5967872844,0.6143347438\H,3
.6363476694,-0.1783651377,0.0957605502\S,0.9129313173,-0.7188051797,-0
.7895066937\S,-0.8947136124,-0.9117769719,0.3340284456\S,-1.8165722679
,0.8090881322,0.5167029406\\Version=EM64L-G09RevD.01\State=2-A\HF=-127
2.3621283\S2=0.752844\S2-1=0.\S2A=0.750007\RMSD=9.586e-09\RMSF=8.021e-
06\Dipole=0.7661443,0.1701089,0.0912304\Quadrupole=1.7933864,0.438809,
-2.2321954,1.7334338,1.1079158,0.5664699\PG=C01 [X(C2H3S3)]\\@

1\1\GINC-ORC338\FOpt\UB3PW91\6-311G(2d,p)\C2H3S3(2)\SNIKOO\19-Aug-2017
\0\\# opt freq ub3pw91/6-311g(2d,p)\\CH2=CH-SSS\\0,2\C,1.6850481814,0.
4935565875,0.1402257813\H,0.9367167062,1.2227725697,0.4492539574\C,2.9
885462292,0.7531628691,0.1575602227\H,3.3341529633,1.7248890855,0.4900
29401\H,3.7395438462,0.0342307727,-0.1478390756\S,1.0100799238,-1.0295
81598,-0.3728389682\S,-1.1017896023,-0.7356361381,-0.1435761791\S,-1.5
423062477,1.0357078515,0.5243558606\\Version=EM64L-G09RevD.01\State=2-
A\HF=-1272.4792589\S2=0.755668\S2-1=0.\S2A=0.750027\RMSD=4.126e-09\RMS
F=8.207e-06\Dipole=0.612965,-0.0346166,-0.0482163\Quadrupole=1.3964861
,0.8116488,-2.2081349,1.7059349,0.3843572,1.1386701\PG=C01 [X(C2H3S3)]
\\@

1\1\GINC-ORC13\FOpt\UB3PW91\6-311+G(2df,p)\C2H3S3(2)\SNIKOO\19-Aug-201
7\0\\# opt freq ub3pw91/6-311+g(2df,p)\\CH2=CH-SSS\\0,2\C,1.6838875224
,0.4931336093,0.1413851052\H,0.9410307202,1.2255051707,0.4576055025\C,
2.9892780747,0.7490540389,0.1660189139\H,3.3373039931,1.715609473,0.51
06640726\H,3.7395196646,0.0316286854,-0.1451980419\S,1.003828961,-1.01
47875539,-0.3889490158\S,-1.0970337853,-0.7256860361,-0.1631655408\S,-
1.5478231508,1.0246446127,0.5188100045\\Version=EM64L-G09RevD.01\State
=2-A\HF=-1272.4953313\S2=0.756202\S2-1=0.\S2A=0.750033\RMSD=8.666e-09\
RMSF=7.216e-06\Dipole=0.5517125,-0.0300993,-0.0415469\Quadrupole=1.404
1105,0.6338677,-2.0379782,1.5415482,0.3692479,1.062698\PG=C01 [X(C2H3S
3)]\\@

1\1\GINC-ORC32\FOpt\UB3PW91\6-311++G(3df,3pd)\C2H3S3(2)\SNIKOO\19-Aug-
2017\0\\# opt freq ub3pw91/6-311++g(3df,3pd)\\CH2=CH-SSS\\0,2\C,1.6829
989095,0.4941376874,0.1409701932\H,0.9430444581,1.229829881,0.45265497
61\C,2.9891478446,0.7485360276,0.1609562765\H,3.3385242698,1.716082333
5,0.4968724105\H,3.7363378026,0.0281757489,-0.1456494597\S,1.001595765
5,-1.0137579133,-0.3744214758\S,-1.0923003489,-0.7260736896,-0.1497266
708\S,-1.5493567012,1.0221719245,0.5155147501\\Version=EM64L-G09RevD.0
1\State=2-A\HF=-1272.5001675\S2=0.756783\S2-1=0.\S2A=0.750039\RMSD=9.1

45e-09\RMSF=1.898e-05\Dipole=0.5630426,-0.0113854,-0.0355024\Quadrupol
e=1.4523686,0.6322764,-2.084645,1.4134391,0.2988207,1.0533569\PG=C01 [
X(C2H3S3)]\\@

$CH_2CHSSS^{\bullet}$ wB97XD

1\1\GINC-ORC278\FOpt\UwB97XD\6-31G(d)\C2H3S3(2)\SNIKOO\20-Aug-2017\0\\
opt freq=noraman uwb97xd/6-31g(d)\\CH2=CH-SSS\\0,2\C,1.6131146152,0.
7602191872,-0.113238306\H,1.0485641123,1.6558105998,-0.3608580164\C,2.
8577295436,0.8103003096,0.3528008801\H,3.3534623631,1.767115452,0.4885
408308\H,3.4186735671,-0.0868121418,0.5968735117\S,0.8246748577,-0.780
5863388,-0.5028930486\S,-0.9624520459,-0.6291920968,0.6079215216\S,-2.
2008190132,0.6063240287,-0.2937133732\\Version=EM64L-G09RevD.01\State=
2-A\HF=-1272.4512729\S2=0.753371\S2-1=0.\S2A=0.750009\RMSD=8.781e-09\R
MSF=2.169e-05\Dipole=0.8084812,0.3265461,0.1948707\Quadrupole=1.645475
8,0.6700044,-2.3154802,2.0585453,1.1356538,-0.000915\PG=C01 [X(C2H3S3)
]\\@

1\1\GINC-ORC248\FOpt\UwB97XD\6-311G(2d,p)\C2H3S3(2)\SNIKOO\20-Aug-2017
\0\\# opt freq=noraman uwb97xd/6-311g(2d,p)\\CH2=CH-SSS\\0,2\C,1.60790
8243,0.748198616,-0.0921688892\H,1.0249676236,1.6335387671,-0.32061814
85\C,2.8553751472,0.8123786688,0.3424396267\H,3.3389502466,1.773552963
2,0.4730457396\H,3.4305606772,-0.0780118505,0.5664647537\S,0.830471194
6,-0.7960393073,-0.4750395139\S,-0.9682972716,-0.6148348065,0.60759762
3\S,-2.1669878607,0.6243959492,-0.3262871914\\Version=EM64L-G09RevD.01
\State=2-A\HF=-1272.5680085\S2=0.755326\S2-1=0.\S2A=0.75002\RMSD=9.721
e-09\RMSF=2.151e-05\Dipole=0.744135,0.2643835,0.1804746\Quadrupole=1.3
624458,0.817457,-2.1799028,1.7614753,0.9903501,-0.1182491\PG=C01 [X(C2
H3S3)]\\@

1\1\GINC-ORC84\FOpt\UwB97XD\6-311+G(2df,p)\C2H3S3(2)\SNIKOO\20-Aug-201
7\0\\# opt freq=noraman uwb97xd/6-311+g(2df,p)\\CH2=CH-SSS\\0,2\C,1.59
79351134,0.7325811523,-0.0485880988\H,0.9891350251,1.6122126801,-0.229
8385327\C,2.8646172388,0.815419925,0.3257377882\H,3.3341359128,1.78401
83648,0.4504653375\H,3.4711607598,-0.0649289214,0.5029446427\S,0.82949
88277,-0.8123377315,-0.4185402072\S,-0.998168205,-0.605029994,0.583065
3256\S,-2.1353666726,0.6412435247,-0.3898122552\\Version=EM64L-G09RevD
.01\State=2-A\HF=-1272.5840565\S2=0.75571\S2-1=0.\S2A=0.750023\RMSD=4.
538e-09\RMSF=2.185e-05\Dipole=0.6534575,0.2331276,0.1785549\Quadrupole
=1.4521304,0.7908191,-2.2429496,1.6490864,0.8292037,-0.0147102\PG=C01
[X(C2H3S3)]\\@

1\1\GINC-ORC225\FOpt\UwB97XD\6-311++G(3df,3pd)\C2H3S3(2)\SNIKOO\20-Aug
-2017\0\\# opt freq=noraman uwb97xd/6-311++g(3df,3pd)\\CH2=CH-SSS\\0,2
\C,1.5928400262,0.7232157982,-0.0353645714\H,0.9747118015,1.59689022,-
0.2052181797\C,2.8641100647,0.8181158863,0.3204846046\H,3.3248660334,1
.7898028859,0.4377408085\H,3.4813156349,-0.0549439185,0.4861722682\S,0
.8339312847,-0.8239143226,-0.3939187576\S,-1.0097062075,-0.6105475068,
0.563982052\S,-2.1091206379,0.6645599576,-0.3984442247\\Version=EM64L-
G09RevD.01\State=2-A\HF=-1272.5890563\S2=0.756186\S2-1=0.\S2A=0.750026
\RMSD=7.421e-09\RMSF=9.124e-06\Dipole=0.6352816,0.2341199,0.1568001\Qu
adrupole=1.5131031,0.7388249,-2.251928,1.5333529,0.8253841,-0.0324011\
PG=C01 [X(C2H3S3)]\\@

$CH_2CHSSS^{\bullet}$ M062X

1\1\GINC-ORC5\FOpt\UM062X\6-31G(d)\C2H3S3(2)\SNIKOO\08-Sep-2017\0\\# o

```
pt=calcfc freq=noraman m062x/6-31g(d) nosymm\\CH2=CH-SSS new\\0,2\C,1.
6753066744,0.6283527235,-0.0910257928\H,1.111087996,1.5381345176,-0.28
27324034\C,2.9312695171,0.6398228437,0.3443418931\H,3.4418720457,1.582
2022654,0.517507332\H,3.4846751136,-0.2761314996,0.5270533768\S,0.8596
190335,-0.88025151,-0.5371814287\S,-0.9474968937,-0.6825965769,0.53846
12098\S,-1.9109434866,0.8963962363,-0.1106811867\\Version=EM64L-G09Rev
D.01\HF=-1272.3882536\S2=0.75369\S2-1=0.\S2A=0.75001\RMSD=4.596e-09\RM
SF=1.082e-05\Dipole=0.770982,0.1940551,0.1768166\Quadrupole=1.8823362,
0.3555595,-2.2378957,1.9689561,1.1457997,0.0079951\PG=C01 [X(C2H3S3)]\
\@
```

```
1\1\GINC-ORC334\FOpt\UM062X\6-311G(2d,p)\C2H3S3(2)\SNIKOO\28-Aug-2017\
0\\# opt=calcfc freq=noraman 6-311g(2d,p) m062x\\CH2=CH-SSS new2\\0,2\
C,1.5720271134,0.6822697675,-0.0775209746\H,0.9502549728,1.5397696977,
-0.3077809289\C,2.8173274698,0.7883091412,0.3548558369\H,3.2669193963,
1.7646595413,0.4880978032\H,3.4213273256,-0.0825958792,0.5760766612\S,
0.8525549006,-0.8882022764,-0.4588927772\S,-1.0048710612,-0.7084991107
,0.5265638075\S,-1.9431311174,0.8524981187,-0.1783154281\\Version=EM64
L-G09RevD.01\State=2-A\HF=-1272.5194006\S2=0.755247\S2-1=0.\S2A=0.7500
19\RMSD=7.765e-09\RMSF=8.659e-06\Dipole=0.7093058,0.1684913,0.1593552\
Quadrupole=1.2465442,0.921987,-2.1685313,1.8255313,0.9260177,-0.169251
7\PG=C01 [X(C2H3S3)]\\@
```

```
1\1\GINC-ORC227\FOpt\UM062X\6-311+G(2df,p)\C2H3S3(2)\ROOT\22-Aug-2017\
0\\# opt freq=noraman 6-311+g(2df,p) m062x\\CH2=CH-SSS new\\0,2\C,1.56
6504687,0.6726268918,-0.0164389614\H,0.9092464141,1.5313806792,-0.1038
888402\C,2.8474761273,0.7776766214,0.2995604588\H,3.2870977449,1.75161
59072,0.4755751114\H,3.4927736289,-0.088773426,0.3760670431\S,0.839304
468,-0.8711657353,-0.4579614003\S,-1.0190715228,-0.714760659,0.4975764
133\S,-1.9561625474,0.8395437207,-0.1917038246\\Version=EM64L-G09RevD.
01\State=2-A\HF=-1272.5359307\S2=0.755249\S2-1=0.\S2A=0.750019\RMSD=6.
553e-09\RMSF=1.502e-05\Dipole=0.6139761,0.1416519,0.1544293\Quadrupole
=1.4382453,0.8182614,-2.2565067,1.6064718,0.7158811,0.1324265\PG=C01 [
X(C2H3S3)]\\@
```

```
1\1\GINC-ORC8\FOpt\UM062X\6-311++G(3df,3pd)\C2H3S3(2)\SNIKOO\20-Aug-20
17\0\\# opt freq=noraman m062x/6-311++g(3df,3pd)\\CH2=CH-SSS\\0,2\C,1.
6384231097,0.6199064714,0.0040596365\H,1.0100755971,1.4953495437,-0.10
65897161\C,2.9193147634,0.6920380168,0.3301808996\H,3.3860948156,1.653
9922171,0.4897688084\H,3.5358723153,-0.1904820965,0.4296642002\S,0.866
4046676,-0.9061575323,-0.4081983606\S,-1.0091969565,-0.6570343452,0.47
91893211\S,-1.8659813122,0.9154617251,-0.252972789\\Version=EM64L-G09R
evD.01\State=2-A\HF=-1272.5410734\S2=0.755837\S2-1=0.\S2A=0.750022\RMS
D=9.982e-09\RMSF=3.695e-05\Dipole=0.6065929,0.1327226,0.1436201\Quadru
pole=1.5796489,0.6740302,-2.253679,1.4492514,0.73943,0.0537085\PG=C01
[X(C2H3S3)]\\@
```

$CH_2CHSSS^{\bullet}$ M08HX

```
1\1\GINC-ORC278\FOpt\UM08HX\6-31G(d)\C2H3S3(2)\SNIKOO\20-Aug-2017\0\\#
 opt freq=noraman m08hx/6-31g(d)\\CH2=CH-SSS\\0,2\C,1.6438919377,0.644
4568641,-0.0684644355\H,1.0518871192,1.5452677695,-0.2486122573\C,2.90
20257922,0.6861517181,0.3616098846\H,3.3911856176,1.6432893464,0.54419
65876\H,3.4844464933,-0.2198826717,0.5318108537\S,0.8663647166,-0.8817
138981,-0.5221901009\S,-0.9591670922,-0.6897805526,0.519216887\S,-1.89
96275844,0.8952854242,-0.1524654192\\Version=EM64L-G16RevA.03\State=2-
A\HF=-1272.3874744\S2=0.753803\S2-1=0.\S2A=0.750011\RMSD=6.307e-09\RMS
```

```
F=1.759e-05\Dipole=0.7611068,0.1796626,0.1791929\Quadrupole=1.6651822,
0.5270441,-2.1922263,1.9384026,1.1087449,0.0952873\PG=C01 [X(C2H3S3)]\
\@
```

```
1\1\GINC-ORC90\FOpt\UM08HX\6-311G(2d,p)\C2H3S3(2)\SNIKOO\20-Aug-2017\0
\\# opt freq=noraman 6-311g(2d,p) m08hx\\CH2=CH-SSS\\0,2\C,1.636725117
8,0.6297929785,-0.048100327\H,1.0204759538,1.5127160095,-0.2088801487\
C,2.8961798887,0.6915039439,0.3521432527\H,3.3689931769,1.653890534,0.
5304690398\H,3.4936286214,-0.2040462166,0.5022668509\S,0.8777799152,-0
.9034227603,-0.4917430701\S,-0.9731834361,-0.6897900365,0.495437748\S,
-1.8395922378,0.9324295476,-0.1664913457\\Version=EM64L-G16RevA.03\Sta
te=2-A\HF=-1272.5292385\S2=0.755504\S2-1=0.\S2A=0.75002\RMSD=8.638e-09
\RMSF=1.368e-05\Dipole=0.6845201,0.123442,0.1573695\Quadrupole=1.35020
04,0.8107363,-2.1609367,1.6964976,0.8888753,-0.0043604\PG=C01 [X(C2H3S
3)]\\@
```

```
1\1\GINC-ORC20\FOpt\UM08HX\6-311+G(2df,p)\C2H3S3(2)\SNIKOO\20-Aug-2017
\0\\# opt freq=noraman 6-311+g(2df,p) m08hx\\CH2=CH-SSS\\0,2\C,1.63201
85675,0.6184842924,-0.0168546526\H,1.0026522321,1.4985577876,-0.145359
4478\C,2.9056318668,0.6923609547,0.3375810477\H,3.372579481,1.65895291
34,0.5086484049\H,3.5219308707,-0.1956668514,0.4556684078\S,0.87112935
52,-0.9128473236,-0.4409667863\S,-0.9922363702,-0.6698116591,0.4851398
962\S,-1.8326990032,0.933043886,-0.2187548699\\Version=EM64L-G16RevA.0
3\State=2-A\HF=-1272.5465711\S2=0.756126\S2-1=0.\S2A=0.750027\RMSD=4.8
04e-09\RMSF=2.005e-05\Dipole=0.6066124,0.1069455,0.1560188\Quadrupole=
1.4672787,0.7146133,-2.181892,1.5764477,0.7774936,0.0725918\PG=C01 [X(
C2H3S3)]\\@
```

```
1\1\GINC-ORC19\FOpt\UM08HX\6-311++G(3df,3pd)\C2H3S3(2)\SNIKOO\20-Aug-2
017\0\\# opt freq=noraman 6-311++g(3df,3pd) m08hx\\CH2=CH-SSS\\0,2\C,1
.6284984933,0.608772681,-0.0061869915\H,0.9890498659,1.4801133143,-0.1
270284516\C,2.9058382485,0.6951724303,0.332466169\H,3.3631197774,1.664
9242913,0.4955959244\H,3.5317365849,-0.1840283219,0.4420584913\S,0.874
3150555,-0.9262308607,-0.4148594517\S,-1.0049879027,-0.6694068189,0.46
29278525\S,-1.8065631228,0.9537572846,-0.2198715423\\Version=EM64L-G16
RevA.03\State=2-A\HF=-1272.5533251\S2=0.756748\S2-1=0.\S2A=0.750032\RM
SD=3.781e-09\RMSF=1.156e-05\Dipole=0.5989507,0.1156759,0.1367913\Quadr
upole=1.5441791,0.6956335,-2.2398126,1.4495326,0.7503605,0.0472853\PG=
C01 [X(C2H3S3)]\\@
```

$CH_2CHSSS^{\bullet}$ QCISD

```
1\1\GINC-ORC85\FOpt\UQCISD-FC\6-311+G(2df,p)\C2H3S3(2)\SNIKOO\26-Aug-2
017\0\\# opt 6-311+g(2df,p) geom=connectivity qcisd\\Title Card Requir
ed\\0,2\C,1.6715205367,0.6841810017,-0.0341622454\H,1.1075509553,1.609
9080957,-0.1021704484\C,2.953567651,0.6580343445,0.3225887976\H,3.4790
960367,1.5799867928,0.548363924\H,3.5126279152,-0.2691232568,0.3873877
899\S,0.8119889654,-0.7762230562,-0.551529876\S,-0.9233494047,-0.60929
86064,0.6182022173\S,-2.1277846557,0.6995866847,-0.226727159\\Version=
EM64L-G09RevD.01\State=2-A\HF=-1270.1027818\MP2=-1270.9018573\MP3=-127
0.9549688\MP4D=-1270.9777895\MP4DQ=-1270.9508624\PUHF=-1270.1073374\PM
P2-0=-1270.9047233\PMP3-0=-1270.9565154\MP4SDQ=-1270.9592797\QCISD=-12
70.9612788\S2=0.771905\S2-1=0.755503\S2A=0.750349\RMSD=3.285e-09\RMSF=
1.261e-05\Dipole=0.6501249,0.216902,0.1694094\PG=C01 [X(C2H3S3)]\\@
```

Cys-SH wB97XD

1\1\GINC-ORC64\FOpt\RwB97XD\6-311G(2d,p)\C3H7N1O2S1\SNIKOO\25-Aug-2017
\0\\# opt freq=noraman rwb97xd/6-311g(2d,p)\\CYS-1S G4\\0,1\C,1.161099
9249,-0.6826868416,0.0059685872\C,0.4702336302,0.6509101211,0.35577206
55\C,-0.806952764,0.8941981267,-0.4644904043\S,-2.237882826,-0.1581906
114,-0.0353773155\N,1.3468186264,1.7931993886,0.1873378838\O,2.2964916
471,-0.7311367981,-0.3676853414\O,0.4365200318,-1.797126566,0.15528165
86\H,0.185129381,0.5986527544,1.4123335882\H,-0.629171264,0.7130986417
,-1.5267589368\H,-1.0868528127,1.9387417187,-0.3501412983\H,2.10769754
07,1.7220317884,0.8537908312\H,1.8016051148,1.7174954478,-0.7181488595
\H,-0.4858110668,-1.5745768305,0.3660791321\H,-2.4562541635,0.36797066
01,1.1782134092\\Version=EM64L-G09RevD.01\State=1-A\HF=-721.9400248\RM
SD=6.617e-09\RMSF=2.637e-05\Dipole=-0.8863282,0.7582517,0.5229189\Quad
rupole=-2.2842489,-2.7830779,5.0673269,3.2689537,-0.5814781,-0.7973165
\PG=C01 [X(C3H7N1O2S1)]\\@

Cys-S$^-$ wB97XD

1\1\GINC-ORC66\FOpt\RwB97XD\6-311G(2d,p)\C3H6N1O2S1(1-)\SNIKOO\25-Aug-
2017\0\\# opt freq=noraman rwb97xd/6-311g(2d,p)\\CYS-1S-NEG G4\\-1,1\C
,-1.0582810732,-0.7167733524,-0.0179525287\C,-0.4704184185,0.663377481
,-0.3657165157\C,0.836861949,0.9813605929,0.3692591687\S,2.2401739792,
-0.1066273254,-0.0763961189\N,-1.4588687157,1.7254981983,-0.1573707746
\O,-2.249844788,-0.8682521398,0.1565453723\O,-0.2196719075,-1.72757101
05,0.0344671574\H,-0.228487401,0.6146672948,-1.4347921471\H,0.64741321
91,0.9282691737,1.4498132533\H,1.0813643865,2.0209451783,0.1366346487\
H,-2.3683835813,1.2731297919,-0.2301026094\H,-1.3981262643,1.999164951
9,0.8204810794\H,0.7373766158,-1.3771618347,-0.0523279854\\Version=EM6
4L-G09RevD.01\State=1-A\HF=-721.401893\RMSD=9.517e-09\RMSF=6.357e-05\D
ipole=-1.5410515,0.6875535,0.274097\Quadrupole=-11.9300985,2.6991838,9
.2309147,-2.0713223,0.0275823,2.7212986\PG=C01 [X(C3H6N1O2S1)]\\@

Cys-S$^•$ wB97XD

1\1\GINC-ORC49\FOpt\UwB97XD\6-311G(2d,p)\C3H6N1O2S1(2)\SNIKOO\25-Aug-2
017\0\\# opt freq=noraman uwb97xd/6-311g(2d,p)\\CYS-1S-NEG G4\\0,2\C,-
1.0261999053,-0.7052644591,0.180974766\C,-0.4666982225,0.6448764296,-0
.3027100899\C,0.8536552152,1.0012518245,0.4026715141\S,2.2897577504,-0
.0019957059,0.0053758227\N,-1.4001945566,1.7347827993,-0.1034788847\O,
-2.0610328874,-0.7927451996,0.7745314378\O,-0.29502913,-1.7915071286,-
0.0955292436\H,-0.2562865688,0.5556620234,-1.3728979144\H,0.7383741014
,0.9622357411,1.4929810666\H,1.0956565449,2.040864466,0.1691965772\H,-
2.1243013227,1.6894980881,-0.8108025657\H,-1.8868595309,1.5766049424,0
.7746344012\H,0.5581563123,-1.5311938913,-0.4771590773\\Version=EM64L-
G09RevD.01\State=2-A\HF=-721.2932136\S2=0.753677\S2-1=0.\S2A=0.750007\
RMSD=2.493e-09\RMSF=1.896e-05\Dipole=0.5566768,0.7009905,-0.529251\Qua
drupole=-1.8261342,-2.2290218,4.055156,-3.4458947,1.8950785,1.1358058\
PG=C01 [X(C3H6N1O2S1)]\\@

Cys-SSH wB97XD

1\1\GINC-ORC60\FOpt\RwB97XD\6-311G(2d,p)\C3H7N1O2S2\SNIKOO\25-Aug-2017
\0\\# opt freq wb97xd/6-311g(2d,p)\\cys-per g4\\0,1\C,1.723575658,0.73
04291528,0.0477282287\C,0.9880034906,-0.5598257405,-0.3494525007\C,-0.
0383207097,-0.9259827005,0.7492126649\S,-1.7728030658,-1.0402692308,0.
2046730322\S,-2.2007674895,0.9061012455,-0.3648190963\N,1.8892293226,-

1.6690201194,-0.5862428027\O,2.8859243559,0.7543384346,0.3280664573\O,
0.9775163168,1.8414450907,0.0907601583\H,0.0073320457,-0.2635299893,1.
6139202791\H,0.1845829494,-1.939987618,1.0864087522\H,2.3377043037,-1.
5508453163,-1.4873620762\H,2.6452638311,-1.6064775805,0.0908291024\H,0
.4461795275,-0.357539105,-1.2775616986\H,0.061165675,1.6521166446,-0.1
699079132\H,-2.5357172113,1.4097678321,0.8365824126\\Version=EM64L-G09
RevD.01\State=1-A\HF=-1120.141436\RMSD=5.124e-09\RMSF=1.531e-05\Dipole
=-0.8162316,-0.1426146,0.0255097\Quadrupole=-3.9833294,-0.8449393,4.82
82687,-6.7168089,-3.6711326,-0.85836\PG=C01 [X(C3H7N1O2S2)]\\@

Cys-SS^{-} wB97XD

1\1\GINC-ORC62\FOpt\RwB97XD\6-311G(2d,p)\C3H6N1O2S2(1-)\SNIKOO\25-Aug-
2017\0\\# opt freq=noraman rwb97xd/6-311g(2d,p)\\cys-per neg g4\\-1,1\
C,1.6060965889,0.7357682881,0.0751517381\C,0.9348999556,-0.582143741,-
0.33957373\C,-0.0852790627,-1.030243556,0.7324681994\S,-1.8193310288,-
1.0083919499,0.1809042814\S,-2.0963208877,1.0044245903,-0.3426794705\N
,1.9140266406,-1.6334650172,-0.6025534096\O,2.7966503326,0.8077894957,
0.2828993978\O,0.8173840912,1.7810079869,0.2104356024\H,-0.0057214564,
-0.4282615774,1.6408722314\H,0.1332154317,-2.0705092304,0.9821763467\H
,2.3743232437,-1.4162492956,-1.4809098341\H,2.6537601619,-1.5135767713
,0.0860910409\H,0.3756099147,-0.3853580844,-1.2581119767\H,-0.14710992
54,1.5546378622,0.0107745828\\Version=EM64L-G09RevD.01\State=1-A\HF=-1
119.6164404\RMSD=8.502e-09\RMSF=1.861e-05\Dipole=1.6979862,-1.0269001,
0.0260217\Quadrupole=-11.9035763,1.3710623,10.532514,-1.1149968,-2.782
2474,-1.3718077\PG=C01 [X(C3H6N1O2S2)]\\@

Cys-SS$^{\bullet}$ wB97XD

1\1\GINC-ORC50\FOpt\UwB97XD\6-311G(2d,p)\C3H6N1O2S2(2)\SNIKOO\25-Aug-2
017\0\\# opt freq=noraman uwb97xd/6-311g(2d,p)\\cys-per neg g4\\0,2\C,
1.6518842729,0.7394858222,0.1229544235\C,0.9473156208,-0.5487326114,-0
.3375468964\C,-0.0648492559,-0.9937219841,0.7449341503\S,-1.8120200424
,-1.0168040458,0.2136348266\S,-2.2035123233,0.8031201574,-0.4373574367
\N,1.872336505,-1.6235151542,-0.6274551395\O,2.8204877936,0.774824948,
0.3763255027\O,0.8770123432,1.81869233,0.2523680127\H,-0.0224276205,-0
.3850258142,1.6488112916\H,0.1509712965,-2.0300855247,1.0062613453\H,2
.2856454327,-1.4746832386,-1.5404755727\H,2.6487753829,-1.5525039056,0
.0252891085\H,0.398286308,-0.3079463699,-1.2521065932\H,-0.0418898036,
1.6253333209,-0.0062491926\\Version=EM64L-G09RevD.01\State=2-A\HF=-111
9.521676\S2=0.755272\S2-1=0.\S2A=0.750018\RMSD=9.698e-09\RMSF=2.533e-0
5\Dipole=-0.5202295,-0.766939,-0.1386296\Quadrupole=-4.8213105,-0.4917
274,5.3130379,-3.802712,-2.9857844,-2.163773\PG=C01 [X(C3H6N1O2S2)]\\@

Cys-SSSH wB97XD

1\1\GINC-ORC56\FOpt\RwB97XD\6-311G(2d,p)\C3H7N1O2S3\SNIKOO\25-Aug-2017
\0\\# opt freq wb97xd/6-311g(2d,p)\\CYS -3S G2\\0,1\C,-2.9226519269,0.
0553534287,-0.0758954343\C,-1.4628071005,0.0397208091,0.4184050629\N,-
0.8974563533,1.3790448705,0.3055854285\C,-0.694034206,-1.0828662877,-0
.2669905252\S,0.9026958469,-1.4792188425,0.5236529297\S,2.3376470411,-
0.508978313,-0.5846817965\S,2.7017852728,1.3616960729,0.258868352\O,-3
.5181528863,-0.9366623724,-0.3817159546\O,-3.4669223774,1.267854913,-0
.0924857095\H,-1.5365430499,-0.1898781422,1.4863557597\H,-0.1396672017
,1.5128743225,0.9636521262\H,-0.5189677837,1.5263315669,-0.6238741897\
H,-1.2799670919,-2.0012125792,-0.2199669384\H,-0.5157765502,-0.8614290
836,-1.3206989311\H,-2.7494131102,1.8785248331,0.1671707237\H,1.932534

4772,2.094629804,-0.5628639034\\Version=EM64L-G09RevD.01\State=1-A\HF=
-1518.3560923\RMSD=3.419e-09\RMSF=1.707e-05\Dipole=0.9184699,0.9469577
,0.0928429\Quadrupole=-12.8709153,6.3737467,6.4971686,-0.6492151,-3.07
37003,-1.0947648\PG=C01 [X(C3H7N1O2S3)]\\@

Cys-SSS^{-} wB97XD

1\1\GINC-ORC57\FOpt\RwB97XD\6-311G(2d,p)\C3H6N1O2S3(1-)\SNIKOO\25-Aug-
2017\0\\# opt freq=noraman wb97xd/6-311g(2d,p)\\CYS-SSS(-) G2\\-1,1\C,
-2.8227164726,0.1274626514,-0.0738067703\C,-1.4056378904,-0.0107023496
,0.5184840809\N,-0.7781171173,1.3075060204,0.5214953483\C,-0.643215650
6,-1.0433512212,-0.3111558693\S,0.9410393027,-1.5824061649,0.427208871
5\S,2.3942002567,-0.4888191669,-0.6053798258\S,2.5516494785,1.40840548
59,0.1922878662\O,-3.6433552028,-0.7521882799,-0.0528472595\O,-3.03328
48521,1.3157567993,-0.6240212446\H,-1.5320679344,-0.4167076813,1.52664
56129\H,-0.7755968923,1.6995875001,1.4542610829\H,0.2101117677,1.29991
84555,0.236066352\H,-1.271631882,-1.928485982,-0.4196450614\H,-0.42496
32846,-0.6483655583,-1.3059601816\H,-2.1748526267,1.7855804916,-0.4304
860023\\Version=EM64L-G09RevD.01\State=1-A\HF=-1517.8338803\RMSD=7.418
e-09\RMSF=1.737e-05\Dipole=-1.8057534,-0.3589347,0.3867306\Quadrupole=
-23.6085666,8.0358434,15.5727232,-6.8748986,-3.4640715,2.0910078\PG=C0
1 [X(C3H6N1O2S3)]\\@

Cys-SSS$^{\bullet}$ wB97XD

1\1\GINC-ORC52\FOpt\UwB97XD\6-311G(2d,p)\C3H6N1O2S3(2)\SNIKOO\25-Aug-2
017\0\\# opt freq=noraman wb97xd/6-311g(2d,p)\\CYS-SSS-RADICAL G2\\0,2
\C,-2.7232297677,0.1776098016,-0.3484213485\C,-1.4480282289,0.02326911
89,0.5069963833\N,-0.875795729,1.3350699953,0.7782734595\C,-0.52170828
67,-0.9892109692,-0.1663342698\S,0.9137801516,-1.4102425357,0.87853158
\S,2.5678711031,-0.7494659272,-0.2352774472\S,2.2706111958,1.035778476
1,-0.9738374438\O,-3.2446817514,-0.745258194,-0.9056256413\O,-3.195352
9109,1.4164937166,-0.3748536258\H,-1.8011118369,-0.3941971483,1.454600
3557\H,-0.4434114507,1.3679274716,1.6915730467\H,-0.1518097578,1.56083
30803,0.1024379194\H,-1.0628777099,-1.9091644029,-0.3768726443\H,-0.14
80602154,-0.5911352548,-1.1112375042\H,-2.5446338053,1.9448837716,0.13
31941802\\Version=EM64L-G09RevD.01\State=2-A\HF=-1517.7329337\S2=0.755
537\S2-1=0.\S2A=0.750021\RMSD=6.119e-09\RMSF=9.053e-06\Dipole=1.017427
3,0.2456562,0.9790249\Quadrupole=-9.200177,4.3612264,4.8389506,-1.5550
436,-4.5466721,0.6573513\PG=C01 [X(C3H6N1O2S3)]\\@

Cys-SSSSH wB97XD

1\1\GINC-ORC70\FOpt\RwB97XD\6-311G(2d,p)\C3H7N1O2S4\SNIKOO\25-Aug-2017
\0\\# opt freq=noraman rwb97xd/6-311g(2d,p)\\CYS-4S G2\\0,1\C,3.242706
9751,0.3719437567,-0.0617474352\C,1.8898806584,-0.0786209561,0.5183051
811\C,1.2988699595,-1.1957145145,-0.332670365\S,-0.1683627405,-1.97470
64009,0.4271551603\S,-1.7922418887,-1.2012670031,-0.5769135614\S,-2.67
11728655,0.3314654898,0.521219699\S,-2.1527790254,2.1484519364,-0.3483
932275\N,1.0286423261,1.0808798996,0.7161046296\O,4.0066018271,-0.3818
495764,-0.5910065496\O,3.4936266367,1.6652348315,0.1154319567\H,2.1305
890738,-0.4809409609,1.5076084925\H,2.0332396105,-1.9904921865,-0.4615
063756\H,1.0233999064,-0.8366188389,-1.3255822994\H,0.5034506444,1.270
5477156,-0.1321781483\H,0.3503429255,0.89332456,1.4444995043\H,2.68974
08964,2.0304502998,0.5344090137\H,-1.2424949199,2.5500139479,0.5541873
248\\Version=EM64L-G09RevD.01\State=1-A\HF=-1916.5610945\RMSD=8.150e-0
9\RMSF=8.926e-06\Dipole=-0.8976313,0.470784,0.6546307\Quadrupole=-12.9

956468,5.985206,7.0104409,-1.3648026,3.8964071,0.8094342\PG=C01 [X(C3H
7N1O2S4)]\\@

Cys-SSSS^{-} wB97XD

1\1\GINC-ORC78\FOpt\RwB97XD\6-311G(2d,p)\C3H6N1O2S4(1-)\SNIKOO\25-Aug-
2017\0\\# opt freq=noraman rwb97xd/6-311g(2d,p)\\CYS-4S NEG G2\\-1,1\C
,3.0719237001,0.2162916068,-0.1558321524\C,1.7587301607,-0.2564752471,
0.4975689246\C,0.9452425447,-1.1130713992,-0.4704134148\S,-0.395921682
3,-2.0112970201,0.3983659766\S,-2.1504736691,-1.2307509227,-0.42134354
75\S,-2.5177193597,0.6385425169,0.412661598\S,-1.501721805,2.068119145
9,-0.6696373508\N,1.0860313991,0.8970598583,1.0754016555\O,3.753433570
1,-0.4708184078,-0.8690359276\O,3.3998489381,1.4518091213,0.1993769503
\H,2.0926664455,-0.9005967816,1.3203527168\H,1.6005400184,-1.841401590
8,-0.9466445968\H,0.4811077572,-0.4941244502,-1.2384683255\H,0.4101345
274,1.31668196,0.4174697054\H,0.5384446432,0.6267256189,1.8819172573\H
,2.6283038116,1.7287949914,0.7595995309\\Version=EM64L-G09RevD.01\Stat
e=1-A\HF=-1916.0495743\RMSD=4.956e-09\RMSF=2.431e-05\Dipole=0.9638121,
-1.3796675,1.2150232\Quadrupole=-15.2354628,1.0456135,14.1898493,4.337
8809,2.6668888,2.3564591\PG=C01 [X(C3H6N1O2S4)]\\@

Cys-SSSS$^{\bullet}$ wB97XD

1\1\GINC-ORC306\FOpt\UwB97XD\6-311G(2d,p)\C3H6N1O2S4(2)\SNIKOO\28-Aug-
2017\0\\# opt=(calcfc,maxstep=1) freq=noraman uwb97xd/6-311g(2d,p)\\1\
\0,2\C,3.1935925199,0.4549086597,0.0068063093\C,1.7527395759,0.0804472
729,0.4216573772\C,1.3206443517,-1.1575236817,-0.3530278946\S,-0.12729
35861,-2.0462647399,0.309661432\S,-1.749562632,-1.2382986069,-0.640832
9232\S,-2.5047613056,0.2409535417,0.6995745726\S,-2.6060477125,1.94215
366,-0.2611653907\N,0.8939312605,1.2523232419,0.3116373664\O,4.0360205
959,-0.3660456787,-0.2179258919\O,3.4081707148,1.7622308976,-0.0392108
247\H,1.831066112,-0.1778037461,1.4819813101\H,2.1270022054,-1.8922045
22,-0.3179466185\H,1.1337879779,-0.9273237297,-1.4032803639\H,0.414567
8235,1.2858704798,-0.5810968435\H,0.1903620703,1.2771517966,1.03605037
96\H,2.5371930285,2.1674491548,0.1558060037\\Version=EM64L-G09RevD.01\
State=2-A\HF=-1915.9386395\S2=0.755447\S2-1=0.\S2A=0.750021\RMSD=5.313
e-09\RMSF=6.530e-06\Dipole=-1.1194363,0.0769871,0.2999472\Quadrupole=-
12.3254428,4.3678547,7.9575881,-0.3767059,1.877708,0.0688771\PG=C01 [X
(C3H6N1O2S4)]\\@

CH_3SeH B3LYP

1\1\GINC-ORC72\FOpt\RB3LYP\6-31G(d)\C1H4Se1\MEISTERP\26-May-2017\0\\#
opt freq b3lyp/6-31g(d)\\Methyl Selenide\\0,1\C,-0.3585995289,1.942675
7478,0.0182246124\H,0.0075646784,0.9160952461,0.0393810489\H,0.0075781
945,2.4742811207,-0.8602404981\H,-1.4503969765,1.940086048,0.01372273\
Se,0.1745781835,2.8916848722,1.661960052\H,1.6365780335,2.7527267029,1
.4213000803\\Version=EM64L-G16RevA.03\State=1-A'\HF=-2439.8843556\RMSD
=3.811e-09\RMSF=1.412e-04\Dipole=0.0532937,-0.2850206,-0.493668\Quadru
pole=1.303483,-1.2740435,-0.0294394,0.2592204,0.4490298,1.077857\PG=CS
[SG(C1H2Se1),X(H2)]\\@

1\1\GINC-ORC72\FOpt\RB3LYP\6-311G(d)\C1H4Se1\MEISTERP\26-May-2017\0\\#
opt freq b3lyp/6-311g(d)\\Methyl Selenide\\0,1\C,-0.3583034939,1.9428
424991,0.0185134378\H,0.0041045874,0.9172693249,0.0333578155\H,0.00411
8043,2.4684778484,-0.8622353447\H,-1.4477093537,1.939864069,0.01333829
24\Se,0.1796236991,2.8940369653,1.6660340612\H,1.6354691037,2.75505903

```
32,1.4253397669\\Version=EM64L-G16RevA.03\State=1-A'\HF=-2442.067407\R
MSD=4.386e-09\RMSF=1.204e-04\Dipole=0.0555217,-0.2822196,-0.4888164\Qu
adrupole=1.3901877,-1.3432429,-0.0469448,0.2536866,0.4394477,1.1226257
\PG=CS [SG(C1H2Se1),X(H2)]\\@
```

```
1\1\GINC-ORC321\FOpt\RB3LYP\6-311G(d,p)\C1H4Se1\MEISTERP\26-May-2017\0
\\# opt freq b3lyp/6-311g(d,p)\\Methyl Selenide\\0,1\C,-0.3582131249,1
.9416532262,0.0164535638\H,0.0049586141,0.9164580054,0.0339317298\H,0.
0049720846,2.4693805204,-0.8626510066\H,-1.4475401023,1.9420748994,0.0
171675549\Se,0.1832335964,2.892454089,1.6632925009\H,1.6298915138,2.75
55289933,1.4261536757\\Version=EM64L-G16RevA.03\State=1-A'\HF=-2442.07
54532\RMSD=3.036e-09\RMSF=1.264e-04\Dipole=0.0628216,-0.2813528,-0.487
3151\Quadrupole=1.3396349,-1.3089771,-0.0306578,0.2692746,0.4664454,1.
1070551\PG=CS [SG(C1H2Se1),X(H2)]\\@
```

```
1\1\GINC-ORC72\FOpt\RB3LYP\6-311+G(d,p)\C1H4Se1\MEISTERP\26-May-2017\0
\\# opt freq b3lyp/6-311+g(d,p)\\Methyl Selenide\\0,1\C,-0.3582439116,
1.9415503579,0.0162753908\H,0.004862022,0.9162713716,0.0337457182\H,0.
0048754934,2.4693127472,-0.8629056426\H,-1.4476731452,1.9422012058,0.0
173863214\Se,0.1832783742,2.8923703359,1.6631474374\H,1.6302037541,2.7
558437233,1.4266988073\\Version=EM64L-G16RevA.03\State=1-A'\HF=-2442.0
760258\RMSD=2.994e-09\RMSF=1.253e-04\Dipole=0.0628227,-0.2917651,-0.50
53496\Quadrupole=1.392738,-1.3571046,-0.0356334,0.2667049,0.4619964,1.
144426\PG=CS [SG(C1H2Se1),X(H2)]\\@
```

```
1\1\GINC-ORC321\FOpt\RB3LYP\6-311G(2d,p)\C1H4Se1\MEISTERP\26-May-2017\
0\\# opt freq b3lyp/6-311g(2d,p)\\Methyl Selenide\\0,1\C,-0.3582600163
,1.9416098285,0.0163783963\H,0.005015954,0.9177751924,0.0348393638\H,0
.0050294141,2.4695079641,-0.861056472\H,-1.4465586513,1.942255558,0.01
74804787\Se,0.1832443046,2.8910248919,1.660817066\H,1.6288315795,2.755
376303,1.4258891932\\Version=EM64L-G16RevA.03\State=1-A'\HF=-2442.0746
597\RMSD=1.907e-09\RMSF=1.295e-04\Dipole=0.0310797,-0.2708143,-0.46906
23\Quadrupole=1.311408,-1.3009993,-0.0104087,0.2538853,0.4397899,1.117
6829\PG=CS [SG(C1H2Se1),X(H2)]\\@
```

```
1\1\GINC-ORC321\FOpt\RB3LYP\6-311G(df,p)\C1H4Se1\MEISTERP\26-May-2017\
0\\# opt freq b3lyp/6-311g(df,p)\\Methyl Selenide\\0,1\C,-0.3575686367
,1.9423908982,0.0177312552\H,0.0050185362,0.9170520932,0.0337538078\H,
0.0050319976,2.4689293918,-0.8622254708\H,-1.4467814742,1.9428220006,0
.0184615799\Se,0.1815775279,2.8904973157,1.6599032548\H,1.6300246305,2
.7558580326,1.4267235889\\Version=EM64L-G16RevA.03\State=1-A'\HF=-2442
.078676\RMSD=2.205e-09\RMSF=1.144e-04\Dipole=0.0589943,-0.283539,-0.49
11017\Quadrupole=1.305647,-1.2563181,-0.0493289,0.2646631,0.4584562,1.
0452813\PG=CS [SG(C1H2Se1),X(H2)]\\@
```

```
1\1\GINC-ORC321\FOpt\RB3LYP\6-311+G(2df,p)\C1H4Se1\MEISTERP\26-May-201
7\0\\# opt freq b3lyp/6-311+g(2df,p)\\Methyl Selenide\\0,1\C,-0.357696
6031,1.942357907,0.0176741111\H,0.0048411746,0.9180700795,0.0341973242
\H,0.0048546261,2.4688045001,-0.8611221117\H,-1.4460528189,1.942852022
4,0.01851359\Se,0.1822276712,2.8894920407,1.658162082\H,1.6291285342,2
.7559731876,1.4269230292\\Version=EM64L-G16RevA.03\State=1-A'\HF=-2442
.077927\RMSD=9.034e-09\RMSF=1.145e-04\Dipole=0.0297798,-0.2812348,-0.4
871112\Quadrupole=1.3342225,-1.3115273,-0.0226952,0.2478586,0.429352,1
.1161601\PG=CS [SG(C1H2Se1),X(H2)]\\@
```

```
1\1\GINC-ORC351\FOpt\RB3LYP\6-311++G(3df,3pd)\C1H4Se1\MEISTERP\26-May-
2017\0\\# opt freq b3lyp/6-311++g(3df,3pd)\\Methyl Selenide\\0,1\C,-0.
```

3580291033,1.941864756,0.0168199459\H,0.0044666219,0.9194435858,0.0343
229795\H,0.0044800565,2.4682265755,-0.8598697942\H,-1.4446707579,1.942
9696817,0.0187174021\Se,0.1823257588,2.8890360846,1.6573723466\H,1.628
730009,2.756009055,1.4269851472\\Version=EM64L-G16RevA.03\State=1-A'\H
F=-2442.0836667\RMSD=2.943e-09\RMSF=1.192e-04\Dipole=0.023675,-0.27600
83,-0.4780586\Quadrupole=1.2684571,-1.2410831,-0.0273739,0.2441196,0.4
228733,1.0511015\PG=CS [SG(C1H2Se1),X(H2)]\\@

CH_3SeH B3PW91

1\1\GINC-ORC141\FOpt\RB3PW91\6-31G(d)\C1H4Se1\MEISTERP\26-May-2017\0\\
opt freq b3pw91/6-31g(d)\\Methyl Selenide\\0,1\C,-0.355322229,1.9453
645444,0.022881781\H,0.0094324077,0.9179276663,0.0426425712\H,0.009445
9244,2.4761894552,-0.8570228051\H,-1.4473363171,1.941538534,0.01623854
84\Se,0.1712576898,2.8870744197,1.6539744865\H,1.6298251096,2.74945511
93,1.4156334457\\Version=EM64L-G16RevA.03\State=1-A'\HF=-2439.8506523\
RMSD=3.076e-09\RMSF=9.811e-05\Dipole=0.0660271,-0.2937317,-0.5087558\Q
uadrupole=1.3491103,-1.3001619,-0.0489484,0.2684502,0.4650173,1.083580
6\PG=CS [SG(C1H2Se1),X(H2)]\\@

1\1\GINC-ORC72\FOpt\RB3PW91\6-311G(d)\C1H4Se1\MEISTERP\26-May-2017\0\\
opt freq b3pw91/6-311g(d)\\Methyl Selenide\\0,1\C,-0.3549583326,1.94
55767504,0.0232493369\H,0.0060102299,0.9186152127,0.0363505404\H,0.006
0236905,2.470396662,-0.8595733995\H,-1.4450717491,1.9413276931,0.01587
33962\Se,0.1759243023,2.8897076175,1.6585353763\H,1.6293744455,2.75192
58056,1.4199127813\\Version=EM64L-G16RevA.03\State=1-A'\HF=-2442.03241
05\RMSD=3.712e-09\RMSF=8.906e-05\Dipole=0.0692222,-0.2894921,-0.501412
6\Quadrupole=1.4228516,-1.3633915,-0.0594602,0.2631029,0.455758,1.1292
36\PG=CS [SG(C1H2Se1),X(H2)]\\@

1\1\GINC-ORC341\FOpt\RB3PW91\6-311G(d,p)\C1H4Se1\MEISTERP\26-May-2017\
0\\# opt freq b3pw91/6-311g(d,p)\\Methyl Selenide\\0,1\C,-0.3548596475
,1.9443758015,0.0211692397\H,0.0067208885,0.9176948141,0.0367500945\H,
0.0067343641,2.4712028745,-0.8601707077\H,-1.4449455102,1.9435855916,0
.0197841821\Se,0.1794713778,2.8880236362,1.6556186965\H,1.6241811127,2
.7526670221,1.4211965242\\Version=EM64L-G16RevA.03\State=1-A'\HF=-2442
.040444\RMSD=1.968e-09\RMSF=9.338e-05\Dipole=0.0751229,-0.288438,-0.49
95867\Quadrupole=1.3687364,-1.3270263,-0.0417101,0.2784684,0.4823702,1
.1131143\PG=CS [SG(C1H2Se1),X(H2)]\\@

1\1\GINC-ORC247\FOpt\RB3PW91\6-311+G(d,p)\C1H4Se1\MEISTERP\26-May-2017
\0\\# opt freq b3pw91/6-311+g(d,p)\\Methyl Selenide\\0,1\C,-0.35487608
69,1.9443754318,0.0211685992\H,0.0066681129,0.9175964643,0.0365971066\
H,0.0066815886,2.4711195584,-0.8603323752\H,-1.4450659049,1.9436285078
,0.0198585133\Se,0.1794974575,2.8879748617,1.6555342172\H,1.6243974179
,2.7528549149,1.4215219665\\Version=EM64L-G16RevA.03\State=1-A'\HF=-24
42.0409029\RMSD=1.835e-09\RMSF=9.177e-05\Dipole=0.074128,-0.2968138,-0
.5140941\Quadrupole=1.4077776,-1.3615572,-0.0462204,0.2756368,0.477467
1,1.1391131\PG=CS [SG(C1H2Se1),X(H2)]\\@

1\1\GINC-ORC164\FOpt\RB3PW91\6-311G(2d,p)\C1H4Se1\MEISTERP\26-May-2017
\0\\# opt freq b3pw91/6-311g(2d,p)\\Methyl Selenide\\0,1\C,-0.35498239
77,1.9442951702,0.0210295808\H,0.0068725661,0.9188735016,0.0377452686\
H,0.0068860338,2.4714753785,-0.8586523468\H,-1.4441931753,1.9437385219
,0.0200490759\Se,0.1795206689,2.8866643313,1.6532643186\H,1.6231988883
,2.7525028336,1.4209121274\\Version=EM64L-G16RevA.03\State=1-A'\HF=-24

42.039533\RMSD=2.156e-09\RMSF=9.728e-05\Dipole=0.0401181,-0.2778383,-0
.4812281\Quadrupole=1.3386474,-1.319423,-0.0192245,0.2614858,0.4529551
,1.1260034\PG=CS [SG(C1H2Se1),X(H2)]\\@

1\1\GINC-ORC146\FOpt\RB3PW91\6-311G(df,p)\C1H4Se1\MEISTERP\26-May-2017
\0\\# opt freq b3pw91/6-311g(df,p)\\Methyl Selenide\\0,1\C,-0.35421786
04,1.9450929277,0.022411345\H,0.0068178067,0.9182762452,0.036567034\H,
0.0068312733,2.4707536237,-0.859758702\H,-1.4442056539,1.9443708791,0.
0211443474\Se,0.1778568069,2.8860579709,1.6522140495\H,1.6242202121,2.
7529980917,1.4217699526\\Version=EM64L-G16RevA.03\State=1-A'\HF=-2442.
0437167\RMSD=7.867e-09\RMSF=8.019e-05\Dipole=0.0713041,-0.2908894,-0.5
038327\Quadrupole=1.3329647,-1.2727548,-0.0602099,0.2741733,0.4749291,
1.0500923\PG=CS [SG(C1H2Se1),X(H2)]\\@

1\1\GINC-ORC145\FOpt\RB3PW91\6-311+G(2df,p)\C1H4Se1\MEISTERP\26-May-20
17\0\\# opt freq b3pw91/6-311+g(2df,p)\\Methyl Selenide\\0,1\C,-0.3544
500161,1.9450503878,0.0223376605\H,0.0067659729,0.9191770974,0.0371462
985\H,0.0067794321,2.4708048586,-0.8586889094\H,-1.4437365568,1.944293
1649,0.0210097499\Se,0.1785529028,2.8852136979,1.6507517404\H,1.623390
85,2.7530105316,1.4217914866\\Version=EM64L-G16RevA.03\State=1-A'\HF=-
2442.042715\RMSD=8.644e-09\RMSF=8.220e-05\Dipole=0.0378559,-0.2861999,
-0.4957108\Quadrupole=1.3473482,-1.3166042,-0.030744,0.2557577,0.44303
38,1.1135861\PG=CS [SG(C1H2Se1),X(H2)]\\@

1\1\GINC-ORC144\FOpt\RB3PW91\6-311++G(3df,3pd)\C1H4Se1\MEISTERP\26-May
-2017\0\\# opt freq b3pw91/6-311++g(3df,3pd)\\Methyl Selenide\\0,1\C,-
0.3547293299,1.9446302428,0.0216099458\H,0.0064268774,0.9203528625,0.0
37270332\H,0.0064403223,2.4703243993,-0.8576086515\H,-1.4425823843,1.9
443792052,0.0211587929\Se,0.1785266336,2.8847871442,1.6500129293\H,1.6
232204687,2.753075889,1.4219046861\\Version=EM64L-G16RevA.03\State=1-A
'\HF=-2442.0483442\RMSD=3.694e-09\RMSF=8.775e-05\Dipole=0.0323909,-0.2
807995,-0.4863571\Quadrupole=1.2832992,-1.2454874,-0.0378118,0.2521708
,0.4368184,1.0458761\PG=CS [SG(C1H2Se1),X(H2)]\\@

CH3SeH wB97XD

1\1\GINC-ORC164\FOpt\RwB97XD\6-31G(d)\C1H4Se1\MEISTERP\26-May-2017\0\\
opt freq wb97xd/6-31g(d)\\Methyl Selenide\\0,1\C,-0.3551637091,1.946
7268134,0.0252412958\H,0.010471135,0.9203694267,0.0444176012\H,0.01048
46334,2.4765057899,-0.8540206565\H,-1.4465688124,1.9407463605,0.014866
4791\Se,0.1720808315,2.8858610921,1.6518729598\H,1.6259985056,2.747340
2538,1.411970344\\Version=EM64L-G16RevA.03\State=1-A'\HF=-2439.8869069
\RMSD=2.180e-09\RMSF=9.063e-05\Dipole=0.0702999,-0.2932433,-0.5079099\
Quadrupole=1.3676488,-1.3037734,-0.0638755,0.2728931,0.4727129,1.07378
09\PG=CS [SG(C1H2Se1),X(H2)]\\@

1\1\GINC-ORC146\FOpt\RwB97XD\6-311G(d)\C1H4Se1\MEISTERP\26-May-2017\0\
\# opt freq wb97xd/6-311g(d)\\Methyl Selenide\\0,1\C,-0.3547452021,1.9
469654867,0.0256546954\H,0.0069440241,0.9207864055,0.0378766522\H,0.00
69574678,2.4706327113,-0.856930029\H,-1.4444714643,1.940629031,0.01466
32904\Se,0.1768413733,2.8884437291,1.6563462772\H,1.6257763877,2.75009
23775,1.4167371454\\Version=EM64L-G16RevA.03\State=1-A'\HF=-2442.07498
3\RMSD=3.411e-09\RMSF=8.693e-05\Dipole=0.0735589,-0.2871565,-0.4973671
\Quadrupole=1.4394549,-1.3678638,-0.0715911,0.2701424,0.4679511,1.1226
032\PG=CS [SG(C1H2Se1),X(H2)]\\@

1\1\GINC-ORC145\FOpt\RwB97XD\6-311G(d,p)\C1H4Se1\MEISTERP\26-May-2017\
0\\# opt freq wb97xd/6-311g(d,p)\\Methyl Selenide\\0,1\C,-0.3545491746
,1.9458588164,0.0237378944\H,0.0074367296,0.9198843885,0.0379471905\H,
0.0074501857,2.4711447996,-0.8576759271\H,-1.4442124996,1.9428434249,0
.0184987263\Se,0.1804303347,2.8868593704,1.6536021491\H,1.6207470085,2
.7509589373,1.4182379912\\Version=EM64L-G16RevA.03\State=1-A'\HF=-2442
.082942\RMSD=9.579e-09\RMSF=8.625e-05\Dipole=0.0778524,-0.2858821,-0.4
951598\Quadrupole=1.3781248,-1.3283481,-0.0497767,0.2852408,0.4941004,
1.1072729\PG=CS [SG(C1H2Se1),X(H2)]\\@

1\1\GINC-ORC144\FOpt\RwB97XD\6-311+G(d,p)\C1H4Se1\MEISTERP\26-May-2017
\0\\# opt freq wb97xd/6-311+g(d,p)\\Methyl Selenide\\0,1\C,-0.35456080
6,1.9458656672,0.0237497601\H,0.0074061134,0.9198274482,0.0378626945\H
,0.0074195695,2.4711000942,-0.857767487\H,-1.4442998271,1.942872259,0.
018548667\Se,0.1804518145,2.8867942478,1.653489354\H,1.6208857208,2.75
10900223,1.4184650386\\Version=EM64L-G16RevA.03\State=1-A'\HF=-2442.08
33842\RMSD=2.291e-09\RMSF=8.511e-05\Dipole=0.0767452,-0.2953905,-0.511
6288\Quadrupole=1.417158,-1.3656491,-0.0515089,0.2835211,0.4911232,1.1
380766\PG=CS [SG(C1H2Se1),X(H2)]\\@

1\1\GINC-ORC144\FOpt\RwB97XD\6-311G(2d,p)\C1H4Se1\MEISTERP\26-May-2017
\0\\# opt freq wb97xd/6-311g(2d,p)\\Methyl Selenide\\0,1\C,-0.35446033
03,1.9459169735,0.0238386264\H,0.0074138865,0.9210123246,0.0386597966\
H,0.0074273332,2.47119797,-0.856342803\H,-1.443270284,1.9430125693,0.0
187917064\Se,0.1803620341,2.8855537362,1.6513407296\H,1.6198299454,2.7
508561644,1.4180599701\\Version=EM64L-G16RevA.03\State=1-A'\HF=-2442.0
820969\RMSD=8.481e-09\RMSF=8.742e-05\Dipole=0.045135,-0.2755603,-0.477
2825\Quadrupole=1.3552601,-1.3264565,-0.0288036,0.2681627,0.4645202,1.
1237986\PG=CS [SG(C1H2Se1),X(H2)]\\@

1\1\GINC-ORC143\FOpt\RwB97XD\6-311G(df,p)\C1H4Se1\MEISTERP\26-May-2017
\0\\# opt freq wb97xd/6-311g(df,p)\\Methyl Selenide\\0,1\C,-0.35398594
37,1.9464696358,0.0247958702\H,0.007494827,0.9203771083,0.03770705\H,0
.0075082748,2.4706904719,-0.8573692879\H,-1.4435633938,1.943584425,0.0
197821824\Se,0.178863281,2.8850812208,1.6505222887\H,1.6209855381,2.75
13468745,1.4189099198\\Version=EM64L-G16RevA.03\State=1-A'\HF=-2442.08
62473\RMSD=8.971e-09\RMSF=7.809e-05\Dipole=0.0743755,-0.288226,-0.4992
196\Quadrupole=1.343712,-1.2752116,-0.0685004,0.2810544,0.4868476,1.04
504\PG=CS [SG(C1H2Se1),X(H2)]\\@

1\1\GINC-ORC142\FOpt\RwB97XD\6-311+G(2df,p)\C1H4Se1\MEISTERP\26-May-20
17\0\\# opt freq wb97xd/6-311+g(2df,p)\\Methyl Selenide\\0,1\C,-0.3540
027112,1.9465850717,0.0249958102\H,0.0072534744,0.9212828767,0.0380170
263\H,0.0072669127,2.4705060405,-0.8564298827\H,-1.442859677,1.9435149
184,0.0196618042\Se,0.1794712884,2.8842384605,1.6490625982\H,1.6201732
966,2.7514223686,1.419040667\\Version=EM64L-G16RevA.03\State=1-A'\HF=-
2442.085261\RMSD=8.187e-09\RMSF=7.775e-05\Dipole=0.0428209,-0.2848866,
-0.4934361\Quadrupole=1.3628317,-1.3252532,-0.0375785,0.2635702,0.4565
658,1.1151573\PG=CS [SG(C1H2Se1),X(H2)]\\@

1\1\GINC-ORC142\FOpt\RwB97XD\6-311++G(3df,3pd)\C1H4Se1\MEISTERP\26-May
-2017\0\\# opt freq wb97xd/6-311++g(3df,3pd)\\Methyl Selenide\\0,1\C,-
0.354298278,1.9460842238,0.0241283142\H,0.0067960739,0.9226402708,0.03
8123728\H,0.0068094954,2.4699197587,-0.8552009961\H,-1.4414478334,1.94
36074464,0.0198220882\Se,0.1794903436,2.8837787033,1.6482662779\H,1.61
9952783,2.7515193339,1.4192086119\\Version=EM64L-G16RevA.03\State=1-A'
\HF=-2442.0912171\RMSD=5.238e-09\RMSF=8.205e-05\Dipole=0.0384035,-0.28

14216,-0.4874346\Quadrupole=1.3048074,-1.2620127,-0.0427947,0.2615964,
0.4531448,1.0558718\PG=CS [SG(C1H2Se1),X(H2)]\\@

CH_3SeH M062X

1\1\GINC-ORC341\FOpt\RM062X\6-31G(d)\C1H4Se1\MEISTERP\26-May-2017\0\\#
opt freq m062x/6-31g(d)\\Methyl Selenide\\0,1\C,-0.3550329685,1.94592
89663,0.02385939\H,0.0107891722,0.9212133128,0.0457746208\H,0.01080266
97,2.4772590596,-0.8526113193\H,-1.4450898457,1.9414675691,0.016115667
8\Se,0.1707536903,2.8850232171,1.6504217018\H,1.6250798674,2.746657614
,1.4107879666\\Version=EM64L-G16RevA.03\State=1-A'\HF=-2439.8923496\RM
SD=6.412e-09\RMSF=1.007e-04\Dipole=0.0764655,-0.2986411,-0.5172589\Qua
drupole=1.369812,-1.2926689,-0.0771431,0.2736644,0.4740485,1.0526739\P
G=CS [SG(C1H2Se1),X(H2)]\\@

1\1\GINC-ORC72\FOpt\RM062X\6-311G(d)\C1H4Se1\MEISTERP\26-May-2017\0\\#
opt freq m062x/6-311g(d)\\Methyl Selenide\\0,1\C,-0.3550737298,1.9457
845506,0.0236092547\H,0.0073462937,0.921620978,0.039362264\H,0.0073597
378,2.471502001,-0.8554644613\H,-1.4432595385,1.9410173048,0.015335816
8\Se,0.176096651,2.8884603176,1.6563749981\H,1.6248331697,2.7491645846
,1.4151301513\\Version=EM64L-G16RevA.03\State=1-A'\HF=-2442.0341401\RM
SD=7.598e-09\RMSF=9.910e-05\Dipole=0.0831235,-0.2969034,-0.514249\Quad
rupole=1.4616178,-1.3771423,-0.0844755,0.2724099,0.4718788,1.1194804\P
G=CS [SG(C1H2Se1),X(H2)]\\@

1\1\GINC-ORC321\FOpt\RM062X\6-311G(d,p)\C1H4Se1\MEISTERP\26-May-2017\0
\\# opt freq m062x/6-311g(d,p)\\Methyl Selenide\\0,1\C,-0.3550752803,1
.9446636824,0.0216678595\H,0.0079058902,0.9207838788,0.0396414193\H,0.
0079193472,2.4721622975,-0.8560498301\H,-1.4432275608,1.9430032236,0.0
187755199\Se,0.1795539046,2.8870055723,1.6538553642\H,1.6202262837,2.7
499310827,1.4164576921\\Version=EM64L-G16RevA.03\State=1-A'\HF=-2442.0
407215\RMSD=3.540e-09\RMSF=9.865e-05\Dipole=0.0895917,-0.2971857,-0.51
47379\Quadrupole=1.4128633,-1.3438183,-0.069045,0.2869886,0.4971284,1.
1039836\PG=CS [SG(C1H2Se1),X(H2)]\\@

1\1\GINC-ORC321\FOpt\RM062X\6-311+G(d,p)\C1H4Se1\MEISTERP\26-May-2017\
0\\# opt freq m062x/6-311+g(d,p)\\Methyl Selenide\\0,1\C,-0.3551060009
,1.9446709305,0.0216804131\H,0.007882156,0.9207298519,0.0395428588\H,0
.007895613,2.4721039551,-0.8561458991\H,-1.4433404438,1.9430169849,0.0
187993534\Se,0.1795750229,2.8869674684,1.6537893668\H,1.6203962373,2.7
50060547,1.4166819326\\Version=EM64L-G16RevA.03\State=1-A'\HF=-2442.04
11207\RMSD=3.757e-09\RMSF=9.737e-05\Dipole=0.0889587,-0.3055892,-0.529
2933\Quadrupole=1.4505953,-1.3777305,-0.0728648,0.2844995,0.4928186,1.
1300446\PG=CS [SG(C1H2Se1),X(H2)]\\@

1\1\GINC-ORC164\FOpt\RM062X\6-311G(2d,p)\C1H4Se1\MEISTERP\26-May-2017\
0\\# opt freq m062x/6-311g(2d,p)\\Methyl Selenide\\0,1\C,-0.3552613406
,1.9445245988,0.0214269575\H,0.0080461743,0.9220551559,0.040647119\H,0
.0080596224,2.4723976225,-0.8544460216\H,-1.4423700494,1.943192941,0.0
191041321\Se,0.1794205596,2.8855859745,1.6513965537\H,1.6194076171,2.7
497934428,1.4162192811\\Version=EM64L-G16RevA.03\State=1-A'\HF=-2442.0
404235\RMSD=3.409e-09\RMSF=1.028e-04\Dipole=0.0562325,-0.2844579,-0.49
26932\Quadrupole=1.3883309,-1.3394149,-0.048916,0.2682715,0.4647093,1.
117603\PG=CS [SG(C1H2Se1),X(H2)]\\@

1\1\GINC-ORC351\FOpt\RM062X\6-311G(df,p)\C1H4Se1\MEISTERP\26-May-2017\
0\\# opt freq m062x/6-311g(df,p)\\Methyl Selenide\\0,1\C,-0.3544626153

,1.9453602431,0.0228743439\H,0.0077254067,0.9210418788,0.0388242421\H,
0.0077388543,2.4713256031,-0.8562349835\H,-1.4426820979,1.9438252293,0
.0201992797\Se,0.1780571667,2.885230609,1.6507810237\H,1.6209258679,2.
7507661718,1.4179041153\\Version=EM64L-G16RevA.03\State=1-A'\HF=-2442.
0440136\RMSD=3.549e-09\RMSF=8.818e-05\Dipole=0.0829036,-0.2986594,-0.5
172906\Quadrupole=1.3596679,-1.2739897,-0.0856782,0.2812685,0.4872185,
1.0291052\PG=CS [SG(C1H2Se1),X(H2)]\\@

1\1\GINC-ORC247\FOpt\RM062X\6-311+G(2df,p)\C1H4Se1\MEISTERP\26-May-201
7\0\\# opt freq m062x/6-311+g(2df,p)\\Methyl Selenide\\0,1\C,-0.354790
1256,1.9452002953,0.022597302\H,0.007728812,0.9220655374,0.0395731235\
H,0.0077422519,2.4714623271,-0.8549740288\H,-1.4421185865,1.9437183555
,0.0200141779\Se,0.1786457719,2.8844572912,1.6494416107\H,1.6200944623
,2.7506459341,1.4176958454\\Version=EM64L-G16RevA.03\State=1-A'\HF=-24
42.0435567\RMSD=2.939e-09\RMSF=9.158e-05\Dipole=0.0514891,-0.2917521,-
0.5053273\Quadrupole=1.3791513,-1.3230039,-0.0561475,0.2628646,0.45534
37,1.097128\PG=CS [SG(C1H2Se1),X(H2)]\\@

1\1\GINC-ORC72\FOpt\RM062X\6-311++G(3df,3pd)\C1H4Se1\MEISTERP\26-May-2
017\0\\# opt freq m062x/6-311++g(3df,3pd)\\Methyl Selenide\\0,1\C,-0.3
550188197,1.944882496,0.0220468556\H,0.0074299466,0.9237795226,0.03972
65404\H,0.0074433653,2.4707382052,-0.8534129661\H,-1.4405346877,1.9436
922098,0.019968916\Se,0.1787218085,2.8841459914,1.6489024262\H,1.61926
09695,2.7503113093,1.4171162474\\Version=EM64L-G16RevA.03\State=1-A'\H
F=-2442.04972\RMSD=8.392e-09\RMSF=9.584e-05\Dipole=0.0446115,-0.286223
7,-0.4957519\Quadrupole=1.3037521,-1.2498476,-0.0539045,0.2588835,0.44
84455,1.0357151\PG=CS [SG(C1H2Se1),X(H2)]\\@

CH_3SeH M08HX

1\1\GINC-ORC321\FOpt\RM08HX\6-31G(d)\C1H4Se1\MEISTERP\26-May-2017\0\\#
opt freq m08hx/6-31g(d)\\Methyl Selenide\\0,1\C,-0.3546374749,1.94690
72998,0.0255539146\H,0.0124213994,0.9175898242,0.0461816934\H,0.012434
9471,2.4794233107,-0.8555458092\H,-1.4489925458,1.9410925335,0.0154660
301\Se,0.170533674,2.8866393007,1.6532208294\H,1.6255425847,2.74589746
88,1.4094713672\\Version=EM64L-G16RevA.03\State=1-A'\HF=-2439.930858\R
MSD=7.272e-09\RMSF=9.729e-05\Dipole=0.0757451,-0.3040246,-0.5265835\Qu
adrupole=1.3723767,-1.3017369,-0.0706398,0.2735916,0.4739226,1.0661592
\PG=CS [SG(C1H2Se1),X(H2)]\\@

1\1\GINC-ORC164\FOpt\RM08HX\6-311G(d)\C1H4Se1\MEISTERP\26-May-2017\0\\
opt freq m08hx/6-311g(d)\\Methyl Selenide\\0,1\C,-0.3548913515,1.946
4198657,0.0247096524\H,0.0091020389,0.9181131157,0.04027441\H,0.009115
5355,2.4740458447,-0.8580462791\H,-1.4471708852,1.9407387836,0.0148533
465\Se,0.1756555152,2.8898297775,1.6587469589\H,1.6254917313,2.7484023
496,1.4138099351\\Version=EM64L-G16RevA.03\State=1-A'\HF=-2442.0527941
\RMSD=6.718e-09\RMSF=9.997e-05\Dipole=0.0792327,-0.3014245,-0.5220799\
Quadrupole=1.4494251,-1.3824254,-0.0669997,0.2717807,0.4707891,1.13919
03\PG=CS [SG(C1H2Se1),X(H2)]\\@

1\1\GINC-ORC351\FOpt\RM08HX\6-311G(d,p)\C1H4Se1\MEISTERP\26-May-2017\0
\\# opt freq m08hx/6-311g(d,p)\\Methyl Selenide\\0,1\C,-0.3549265209,1
.9452119998,0.0226175726\H,0.0096828765,0.9173834559,0.0406622461\H,0.
0096963855,2.4747465419,-0.8584842623\H,-1.4470528826,1.9427825948,0.0
183933232\Se,0.1789980901,2.8882557633,1.6560207443\H,1.6209046374,2.7
49169386,1.4151384085\\Version=EM64L-G16RevA.03\State=1-A'\HF=-2442.05
93919\RMSD=3.060e-09\RMSF=1.004e-04\Dipole=0.0822831,-0.3002289,-0.520

009\Quadrupole=1.3928608,-1.3451082,-0.0477526,0.2843341,0.4925307,1.1
235406\PG=CS [SG(C1H2Se1),X(H2)]\\@

1\1\GINC-ORC247\FOpt\RM08HX\6-311+G(d,p)\C1H4Se1\MEISTERP\26-May-2017\
0\\# opt freq m08hx/6-311+g(d,p)\\Methyl Selenide\\0,1\C,-0.3549203062
,1.9452479959,0.0226799197\H,0.0095635949,0.9172473776,0.0403957526\H,
0.0095771037,2.4745837918,-0.8587353568\H,-1.4472035669,1.9428043546,0
.0184310098\Se,0.1790336249,2.8882261023,1.6559693705\H,1.6212521354,2
.749440117,1.4156073322\\Version=EM64L-G16RevA.03\State=1-A'\HF=-2442.
0600161\RMSD=3.177e-09\RMSF=9.805e-05\Dipole=0.0824063,-0.3073418,-0.5
323289\Quadrupole=1.433577,-1.375628,-0.057949,0.2794006,0.4839868,1.1
411414\PG=CS [SG(C1H2Se1),X(H2)]\\@

1\1\GINC-ORC134\FOpt\RM08HX\6-311G(2d,p)\C1H4Se1\MEISTERP\26-May-2017\
0\\# opt freq m08hx/6-311g(2d,p)\\Methyl Selenide\\0,1\C,-0.3550449722
,1.9453028661,0.0227749555\H,0.0098368756,0.9188990674,0.0416762731\H,
0.0098503725,2.4748669117,-0.8566646898\H,-1.4460778588,1.9428151242,0
.0184496803\Se,0.1786996407,2.8868202085,1.6535342929\H,1.6200385253,2
.7488455587,1.4145775116\\Version=EM64L-G16RevA.03\State=1-A'\HF=-2442
.0593923\RMSD=3.071e-09\RMSF=1.041e-04\Dipole=0.0489725,-0.2855387,-0.
4945655\Quadrupole=1.3706955,-1.339381,-0.0313145,0.2638223,0.457003,1
.1328173\PG=CS [SG(C1H2Se1),X(H2)]\\@

1\1\GINC-ORC136\FOpt\RM08HX\6-311G(df,p)\C1H4Se1\MEISTERP\26-May-2017\
0\\# opt freq m08hx/6-311g(df,p)\\Methyl Selenide\\0,1\C,-0.3543235037
,1.9459912678,0.0239673098\H,0.0094014587,0.9176650121,0.0396652437\H,
0.0094149565,2.4737423374,-0.8587389283\H,-1.4465303084,1.9435727439,0
.0197619055\Se,0.1775663217,2.886406531,1.6528177674\H,1.6217736575,2.
750171843,1.4168747231\\Version=EM64L-G16RevA.03\State=1-A'\HF=-2442.0
629743\RMSD=3.341e-09\RMSF=8.800e-05\Dipole=0.0781245,-0.299318,-0.518
4314\Quadrupole=1.341445,-1.2699014,-0.0715436,0.2769442,0.4797282,1.0
378058\PG=CS [SG(C1H2Se1),X(H2)]\\@

1\1\GINC-ORC341\FOpt\RM08HX\6-311+G(2df,p)\C1H4Se1\MEISTERP\26-May-201
7\0\\# opt freq m08hx/6-311+g(2df,p)\\Methyl Selenide\\0,1\C,-0.354589
7937,1.9459997034,0.0239819166\H,0.0093301946,0.9187712416,0.040307595
\H,0.0093436828,2.4737455191,-0.8574597304\H,-1.4459354477,1.943325885
7,0.0193343447\Se,0.1779721128,2.8856888346,1.6515746903\H,1.621181836
2,2.7500185539,1.4166092105\\Version=EM64L-G16RevA.03\State=1-A'\HF=-2
442.0630476\RMSD=3.144e-09\RMSF=9.092e-05\Dipole=0.0460226,-0.2891596,
-0.5008371\Quadrupole=1.3671279,-1.3175017,-0.0496262,0.2545802,0.4409
944,1.0980109\PG=CS [SG(C1H2Se1),X(H2)]\\@
1\1\GINC-ORC241\FOpt\RM08HX\6-311++G(3df,3pd)\C1H4Se1\MEISTERP\26-May-
2017\0\\# opt freq m08hx/6-311++g(3df,3pd)\\Methyl Selenide\\0,1\C,-0.
355071093,1.9452809569,0.0227370074\H,0.0090657184,0.9209354364,0.0409
006864\H,0.0090791829,2.4731770627,-0.8552889377\H,-1.4439313463,1.943
3890261,0.0194437369\Se,0.178316615,2.8852088602,1.6507433577\H,1.6198
435082,2.7495583966,1.4158121769\\Version=EM64L-G16RevA.03\State=1-A'\
HF=-2442.0694153\RMSD=2.124e-09\RMSF=9.922e-05\Dipole=0.0448128,-0.287
0559,-0.4971933\Quadrupole=1.3038479,-1.2525174,-0.0513305,0.2544163,0
.4407082,1.0402565\PG=CS [SG(C1H2Se1),X(H2)]\\@

CH_3SeH QCISD

1\1\GINC-ORC264\FOpt\RQCISD-FC\6-311+G(2df,p)\C1H4Se1\MEISTERP\20-Aug-
2017\0\\# opt freq=noraman qcisd/6-311+g(2df,p)\\Methyl Selenide\\0,1\
C,-0.3553286611,1.9457910007,0.0236204228\H,0.0065219947,0.9188076491,

```
0.0359673306\H,0.0065354499,2.4699685676,-0.8595983447\H,-1.4461345327
,1.94294479,0.0186742665\Se,0.1804844145,2.8862816299,1.6526014767\H,1
.6252239191,2.7537561003,1.4230828734\\Version=EM64L-G09RevE.01\State=
1-A'\HF=-2440.0044521\MP2=-2440.3345503\MP3=-2440.3655458\MP4D=-2440.3
746293\MP4DQ=-2440.3662485\MP4SDQ=-2440.3683911\QCISD=-2440.3684828\RM
SD=2.256e-09\RMSF=8.452e-05\Dipole=0.0501059,-0.2814557,-0.4874935\PG=
CS [SG(C1H2Se1),X(H2)]\\@
```


CH_3Se^- B3LYP


```
1\1\GINC-ORC341\FOpt\RB3LYP\6-31G(d)\C1H3Se1(1-)\MEISTERP\26-May-2017\
0\\# opt freq b3lyp/6-31g(d)\\Methyl Selenide\\-1,1\C,-0.392664419,1.9
400246977,0.0136323602\H,-0.0441843103,0.8978421605,-0.0189873492\H,-0
.044171001,2.4328597163,-0.9052326215\H,-1.4914039645,1.9212017513,-0.
0189863489\Se,0.2713488839,2.8790606772,1.6400958195\\Version=EM64L-G1
6RevA.03\State=1-A1\HF=-2439.2984194\RMSD=3.471e-09\RMSF=6.615e-05\Dip
ole=-0.2062638,-0.2916947,-0.5052317\Quadrupole=-0.0337129,-0.016857,0
.0505699,0.0238393,0.0412911,0.0583931\PG=C03V [C3(C1Se1),3SGV(H1)]\\@
```

```
1\1\GINC-ORC341\FOpt\RB3LYP\6-311G(d)\C1H3Se1(1-)\MEISTERP\26-May-2017
\0\\# opt freq b3lyp/6-311g(d)\\Methyl Selenide\\-1,1\C,-0.3951131425,
1.9365617558,0.0076343488\H,-0.0439571019,0.8991235708,-0.0178765211\H
,-0.0439438009,2.4331810175,-0.9035674727\H,-1.4902715621,1.9218430798
,-0.0178755215\Se,0.2722085411,2.8802763894,1.6422015019\\Version=EM64
L-G16RevA.03\State=1-A1\HF=-2441.5007321\RMSD=2.910e-09\RMSF=1.265e-04
\Dipole=-0.2286151,-0.3233034,-0.5599798\Quadrupole=-0.0638218,-0.0319
12,0.0957338,0.0451302,0.078168,0.1105438\PG=C03V [C3(C1Se1),3SGV(H1)]
\\@
```

```
1\1\GINC-ORC321\FOpt\RB3LYP\6-311G(d,p)\C1H3Se1(1-)\MEISTERP\26-May-20
17\0\\# opt freq b3lyp/6-311g(d,p)\\Methyl Selenide\\-1,1\C,-0.3957665
402,1.9356377322,0.0060338876\H,-0.0431133635,0.8989098384,-0.01662209
75\H,-0.0431000503,2.4343742365,-0.9031253551\H,-1.4907543021,1.922567
3283,-0.016621097\Se,0.2716533448,2.8794912405,1.6408415796\\Version=E
M64L-G16RevA.03\State=1-A1\HF=-2441.505491\RMSD=8.284e-09\RMSF=1.303e-
04\Dipole=-0.2253721,-0.3187173,-0.5520364\Quadrupole=-0.0472725,-0.02
36371,0.0709095,0.0334277,0.0578986,0.0818792\PG=C03V [C3(C1Se1),3SGV(
H1)]\\@
```

```
1\1\GINC-ORC341\FOpt\RB3LYP\6-311+G(d,p)\C1H3Se1(1-)\MEISTERP\26-May-2
017\0\\# opt freq b3lyp/6-311+g(d,p)\\Methyl Selenide\\-1,1\C,-0.39778
53608,1.9327827515,0.0010888995\H,-0.0415738467,0.8990886927,-0.014004
8209\H,-0.0415605162,2.4365514209,-0.9016618181\H,-1.4910988176,1.9240
784216,-0.014003819\Se,0.2709236918,2.8784593779,1.6390543352\\Version
=EM64L-G16RevA.03\State=1-A1\HF=-2441.5151216\RMSD=2.147e-09\RMSF=1.95
0e-04\Dipole=-0.3116823,-0.4407757,-0.7634484\Quadrupole=-0.2638241,-0
.1319168,0.3957409,0.1865574,0.323128,0.4569619\PG=C03V [C3(C1Se1),3SG
V(H1)]\\@
```

```
1\1\GINC-ORC351\FOpt\RB3LYP\6-311G(2d,p)\C1H3Se1(1-)\MEISTERP\26-May-2
017\0\\# opt freq b3lyp/6-311g(2d,p)\\Methyl Selenide\\-1,1\C,-0.39597
53783,1.935342397,0.0055223504\H,-0.0431697883,0.9001440493,-0.0160016
899\H,-0.0431564865,2.4342944239,-0.9017462935\H,-1.4895718612,1.92292
55111,-0.0160006902\Se,0.2707874654,2.878266729,1.6387206564\\Version=
EM64L-G16RevA.03\State=1-A1\HF=-2441.5043732\RMSD=6.898e-09\RMSF=1.287
e-04\Dipole=-0.214784,-0.3037438,-0.5261014\Quadrupole=-0.0101141,-0.0
050572,0.0151714,0.007152,0.0123876,0.0175184\PG=C03V [C3(C1Se1),3SGV(
```

H1)]\\@

1\1\GINC-ORC341\FOpt\RB3LYP\6-311G(df,p)\C1H3Se1(1-)\MEISTERP\26-May-2
017\0\\# opt freq b3lyp/6-311g(df,p)\\Methyl Selenide\\-1,1\C,-0.39512
82681,1.9365403654,0.0075972994\H,-0.0431390717,0.8995385481,-0.016301
1049\H,-0.0431257643,2.4343378715,-0.9024203803\H,-1.4901529756,1.9227
526485,-0.0163001048\Se,0.2704644824,2.8778099721,1.6379295276\\Versio
n=EM64L-G16RevA.03\State=1-A1\HF=-2441.5081854\RMSD=1.385e-09\RMSF=1.3
94e-04\Dipole=-0.2282521,-0.3227901,-0.5590907\Quadrupole=-0.013277,-0
.0066387,0.0199158,0.0093886,0.0162615,0.0229967\PG=C03V [C3(C1Se1),3S
GV(H1)]\\@

1\1\GINC-ORC72\FOpt\RB3LYP\6-311+G(2df,p)\C1H3Se1(1-)\MEISTERP\26-May-
2017\0\\# opt freq b3lyp/6-311+g(2df,p)\\Methyl Selenide\\-1,1\C,-0.39
73745007,1.9333637827,0.0020952783\H,-0.0416000069,0.90085417,-0.01302
82744\H,-0.0415866919,2.4365144016,-0.8996445968\H,-1.4894255779,1.924
6422174,-0.0130272737\Se,0.268886783,2.8755788172,1.6340650411\\Versio
n=EM64L-G16RevA.03\State=1-A1\HF=-2441.5161732\RMSD=9.286e-09\RMSF=2.6
25e-04\Dipole=-0.2985894,-0.42226,-0.7313781\Quadrupole=-0.2177685,-0.
1088882,0.3266567,0.1539902,0.2667197,0.3771904\PG=C03V [C3(C1Se1),3SG
V(H1)]\\@

1\1\GINC-ORC321\FOpt\RB3LYP\6-311++G(3df,3pd)\C1H3Se1(1-)\MEISTERP\26-
May-2017\0\\# opt freq b3lyp/6-311++g(3df,3pd)\\Methyl Selenide\\-1,1\
C,-0.397583406,1.9330683524,0.0015835763\H,-0.0418367653,0.9021160476,
-0.0126863772\H,-0.0418234642,2.4361795606,-0.8983808311\H,-1.48815694
47,1.9248396008,-0.0126853775\Se,0.2682970218,2.8747447873,1.632620454
\\Version=EM64L-G16RevA.03\State=1-A1\HF=-2441.5217345\RMSD=3.723e-09\
RMSF=2.236e-04\Dipole=-0.2821035,-0.3989458,-0.6909968\Quadrupole=-0.1
328508,-0.0664278,0.1992785,0.0939425,0.1627137,0.2301069\PG=C03V [C3(
C1Se1),3SGV(H1)]\\@

CH_3Se^- B3PW91

1\1\GINC-ORC136\FOpt\RB3PW91\6-31G(d)\C1H3Se1(1-)\MEISTERP\26-May-2017
\0\\# opt freq b3pw91/6-31g(d)\\Methyl Selenide\\-1,1\C,-0.3914741306,
1.9417079827,0.0165479049\H,-0.0432249242,0.8993329219,-0.0165600158\H
,-0.043211616,2.4342164615,-0.9027279136\H,-1.4903182274,1.9226031673,
-0.0165590156\Se,0.2671442528,2.8731145616,1.6297968109\\Version=EM64L
-G16RevA.03\State=1-A1\HF=-2439.2655067\RMSD=4.976e-09\RMSF=4.228e-05\
Dipole=-0.2178039,-0.3080145,-0.5334986\Quadrupole=-0.0540623,-0.02703
21,0.0810945,0.038229,0.0662148,0.0936398\PG=C03V [C3(C1Se1),3SGV(H1)]
\\@

1\1\GINC-ORC351\FOpt\RB3PW91\6-311G(d)\C1H3Se1(1-)\MEISTERP\26-May-201
7\0\\# opt freq b3pw91/6-311g(d)\\Methyl Selenide\\-1,1\C,-0.393574149
3,1.9387381729,0.0114040264\H,-0.0431185297,0.900274143,-0.0158428777\
H,-0.0431052284,2.4343669122,-0.9015542228\H,-1.489466292,1.9230172006
,-0.0158418781\Se,0.2681789085,2.8745777535,1.632331142\\Version=EM64L
-G16RevA.03\State=1-A1\HF=-2441.4643156\RMSD=5.151e-09\RMSF=9.808e-05\
Dipole=-0.2404173,-0.339994,-0.5888888\Quadrupole=-0.0887363,-0.044369
8,0.1331061,0.062748,0.108683,0.1536976\PG=C03V [C3(C1Se1),3SGV(H1)]\\
@

1\1\GINC-ORC323\FOpt\RB3PW91\6-311G(d,p)\C1H3Se1(1-)\MEISTERP\26-May-2
017\0\\# opt freq b3pw91/6-311g(d,p)\\Methyl Selenide\\-1,1\C,-0.39416
43011,1.9379035906,0.0099584826\H,-0.042293258,0.9000771235,-0.0146089

614\H,-0.0422799449,2.4355340152,-0.9011078852\H,-1.4899271196,1.92372
96091,-0.0146079609\Se,0.2675766476,2.8737260467,1.6308559376\\Version
=EM64L-G16RevA.03\State=1-A1\HF=-2441.469113\RMSD=2.497e-09\RMSF=1.075
e-04\Dipole=-0.2370357,-0.3352118,-0.5806057\Quadrupole=-0.0715286,-0.
0357656,0.1072942,0.0505799,0.0876072,0.1238926\PG=C03V [C3(C1Se1),3SG
V(H1)]\\@

1\1\GINC-ORC241\FOpt\RB3PW91\6-311+G(d,p)\C1H3Se1(1-)\MEISTERP\26-May-
2017\0\\# opt freq b3pw91/6-311+g(d,p)\\Methyl Selenide\\-1,1\C,-0.395
684722,1.935753438,0.0062342967\H,-0.0410474252,0.9003324532,-0.012427
1167\H,-0.041034099,2.4372958703,-0.8997958357\H,-1.4901016439,1.92498
93033,-0.0124261152\Se,0.2667717227,2.872587736,1.6288843191\\Version=
EM64L-G16RevA.03\State=1-A1\HF=-2441.4768049\RMSD=5.168e-09\RMSF=1.149
e-04\Dipole=-0.3128436,-0.4424179,-0.7662929\Quadrupole=-0.2500668,-0.
1250379,0.3751047,0.1768292,0.3062782,0.4331333\PG=C03V [C3(C1Se1),3SG
V(H1)]\\@

1\1\GINC-ORC147\FOpt\RB3PW91\6-311G(2d,p)\C1H3Se1(1-)\MEISTERP\26-May-
2017\0\\# opt freq b3pw91/6-311g(2d,p)\\Methyl Selenide\\-1,1\C,-0.394
363027,1.937622556,0.0094717148\H,-0.0422500597,0.9011460059,-0.013921
3193\H,-0.0422367553,2.435595092,-0.8998383847\H,-1.4889337588,1.92412
66115,-0.0139203195\Se,0.266691284,2.872473981,1.628687289\\Version=EM
64L-G16RevA.03\State=1-A1\HF=-2441.4679081\RMSD=9.656e-09\RMSF=1.094e-
04\Dipole=-0.2252265,-0.3185114,-0.5516798\Quadrupole=-0.0353331,-0.01
76672,0.0530003,0.024985,0.0432755,0.0611994\PG=C03V [C3(C1Se1),3SGV(H
1)]\\@

1\1\GINC-ORC145\FOpt\RB3PW91\6-311G(df,p)\C1H3Se1(1-)\MEISTERP\26-May-
2017\0\\# opt freq b3pw91/6-311g(df,p)\\Methyl Selenide\\-1,1\C,-0.393
4784455,1.9388735156,0.0116384476\H,-0.0423739615,0.9006976084,-0.0143
82524\H,-0.0423606547,2.4354198759,-0.9004573111\H,-1.4893152169,1.923
8603375,-0.014381524\Se,0.2664393012,2.8721176313,1.6280700712\\Versio
n=EM64L-G16RevA.03\State=1-A1\HF=-2441.4718728\RMSD=2.000e-09\RMSF=1.0
14e-04\Dipole=-0.2397622,-0.3390675,-0.5872841\Quadrupole=-0.0364164,-
0.0182088,0.0546252,0.025751,0.0446023,0.0630757\PG=C03V [C3(C1Se1),3S
GV(H1)]\\@

1\1\GINC-ORC145\FOpt\RB3PW91\6-311+G(2df,p)\C1H3Se1(1-)\MEISTERP\26-Ma
y-2017\0\\# opt freq b3pw91/6-311+g(2df,p)\\Methyl Selenide\\-1,1\C,-0
.3951433627,1.9365190189,0.0075603261\H,-0.0410852377,0.9019193822,-0.
0115726799\H,-0.0410719258,2.4372423745,-0.8979942966\H,-1.4885928584,
1.9254826003,-0.0115716795\Se,0.2647927192,2.869789064,1.6240368608\\V
ersion=EM64L-G16RevA.03\State=1-A1\HF=-2441.4778057\RMSD=9.449e-09\RMS
F=1.061e-04\Dipole=-0.2982001,-0.4217094,-0.7304246\Quadrupole=-0.2052
479,-0.1026276,0.3078755,0.1451365,0.2513847,0.3555038\PG=C03V [C3(C1S
e1),3SGV(H1)]\\@

1\1\GINC-ORC144\FOpt\RB3PW91\6-311++G(3df,3pd)\C1H3Se1(1-)\MEISTERP\26
-May-2017\0\\# opt freq b3pw91/6-311++g(3df,3pd)\\Methyl Selenide\\-1,
1\C,-0.3954162107,1.936133162,0.0068920001\H,-0.0412091096,0.902945598
7,-0.0111824945\H,-0.0411958081,2.4370671809,-0.8969104749\H,-1.487584
037,1.9257078653,-0.0111814949\Se,0.2643014401,2.8690943057,1.62283350
01\\Version=EM64L-G16RevA.03\State=1-A1\HF=-2441.4832365\RMSD=8.541e-0
9\RMSF=1.068e-04\Dipole=-0.2815384,-0.3981467,-0.6896125\Quadrupole=-0
.1213067,-0.0606555,0.1819622,0.0857794,0.1485747,0.2101117\PG=C03V [C
3(C1Se1),3SGV(H1)]\\@

CH_3Se^- wB97XD

```
1\1\GINC-ORC147\FOpt\RwB97XD\6-31G(d)\C1H3Se1(1-)\MEISTERP\26-May-2017
 \0\\# opt freq wb97xd/6-31g(d)\\Methyl Selenide\\-1,1\C,-0.3914803284,
 1.9416992178,0.0165327237\H,-0.0431131512,0.9004529316,-0.0157308745\H
 ,-0.0430998514,2.434374516,-0.9013433857\H,-1.48929952,1.9230818644,-0
 .015729875\Se,0.2659054572,2.8713626786,1.6267624503\\Version=EM64L-G1
 6RevA.03\State=1-A1\HF=-2439.3012419\RMSD=3.087e-09\RMSF=3.879e-05\Dip
 ole=-0.2204654,-0.3117783,-0.5400177\Quadrupole=-0.0594673,-0.0297347,
 0.089202,0.042051,0.0728347,0.1030015\PG=C03V [C3(C1Se1),3SGV(H1)]\\@
```

```
1\1\GINC-ORC145\FOpt\RwB97XD\6-311G(d)\C1H3Se1(1-)\MEISTERP\26-May-201
 7\0\\# opt freq wb97xd/6-311g(d)\\Methyl Selenide\\-1,1\C,-0.393183294
 6,1.9392909129,0.0123614034\H,-0.0432906966,0.9012257128,-0.0155746536
 \H,-0.0432774057,2.4341234207,-0.9005960278\H,-1.4885117512,1.92317205
 16,-0.0155736548\Se,0.2671770062,2.8731608808,1.6298770384\\Version=EM
 64L-G16RevA.03\State=1-A1\HF=-2441.5062635\RMSD=4.243e-09\RMSF=8.892e-
 05\Dipole=-0.2406665,-0.3403463,-0.589499\Quadrupole=-0.0953902,-0.047
 6968,0.143087,0.067453,0.1168325,0.1652225\PG=C03V [C3(C1Se1),3SGV(H1)
 ]\\@
```

```
1\1\GINC-ORC145\FOpt\RwB97XD\6-311G(d,p)\C1H3Se1(1-)\MEISTERP\26-May-2
 017\0\\# opt freq wb97xd/6-311g(d,p)\\Methyl Selenide\\-1,1\C,-0.39367
 06428,1.9386017136,0.0111676712\H,-0.0426270586,0.9010861105,-0.014571
 535\H,-0.0426137583,2.4350619404,-0.900215365\H,-1.4888645702,1.923751
 2075,-0.0145705355\Se,0.2666880869,2.8724694598,1.628679458\\Version=E
 M64L-G16RevA.03\State=1-A1\HF=-2441.5108722\RMSD=2.500e-09\RMSF=9.937e
 -05\Dipole=-0.2370261,-0.3351982,-0.5805822\Quadrupole=-0.0784905,-0.0
 392467,0.1177372,0.0555028,0.096134,0.1359511\PG=C03V [C3(C1Se1),3SGV(
 H1)]\\@
```

```
1\1\GINC-ORC144\FOpt\RwB97XD\6-311+G(d,p)\C1H3Se1(1-)\MEISTERP\26-May-
 2017\0\\# opt freq wb97xd/6-311+g(d,p)\\Methyl Selenide\\-1,1\C,-0.395
 1225638,1.9365484323,0.0076112718\H,-0.041370165,0.901416838,-0.012328
 0985\H,-0.0413568522,2.4368394367,-0.8988072231\H,-1.488971695,1.92504
 64612,-0.012327098\Se,0.2657257962,2.8711086051,1.6263223807\\Version=
 EM64L-G16RevA.03\State=1-A1\HF=-2441.5189008\RMSD=4.357e-09\RMSF=1.113
 e-04\Dipole=-0.3178311,-0.4494712,-0.7785095\Quadrupole=-0.275899,-0.1
 379545,0.4138534,0.1950959,0.3379171,0.4778765\PG=C03V [C3(C1Se1),3SGV
 (H1)]\\@
```

```
1\1\GINC-ORC143\FOpt\RwB97XD\6-311G(2d,p)\C1H3Se1(1-)\MEISTERP\26-May-
 2017\0\\# opt freq wb97xd/6-311g(2d,p)\\Methyl Selenide\\-1,1\C,-0.393
 7838406,1.9384416313,0.0108903995\H,-0.0426851658,0.9021541181,-0.0140
 498291\H,-0.0426718755,2.4349797508,-0.8990295905\H,-1.4878382677,1.92
 40524061,-0.0140488302\Se,0.2658871718,2.8713368196,1.6267176611\\Vers
 ion=EM64L-G16RevA.03\State=1-A1\HF=-2441.5097467\RMSD=1.775e-09\RMSF=9
 .971e-05\Dipole=-0.2255291,-0.3189393,-0.5524209\Quadrupole=-0.0411038
 ,-0.0205527,0.0616565,0.0290657,0.0503434,0.0711947\PG=C03V [C3(C1Se1)
 ,3SGV(H1)]\\@
```

```
1\1\GINC-ORC143\FOpt\RwB97XD\6-311G(df,p)\C1H3Se1(1-)\MEISTERP\26-May-
 2017\0\\# opt freq wb97xd/6-311g(df,p)\\Methyl Selenide\\-1,1\C,-0.393
 0718567,1.9394485063,0.0126343641\H,-0.0427302989,0.9016038844,-0.0144
 411984\H,-0.0427170044,2.4349159309,-0.8997017917\H,-1.4883419933,1.92
 38264528,-0.0144401992\Se,0.2657721564,2.8711741668,1.6264359372\\Vers
 ion=EM64L-G16RevA.03\State=1-A1\HF=-2441.513651\RMSD=2.462e-09\RMSF=9.
```

299e-05\Dipole=-0.239347,-0.3384803,-0.5862671\Quadrupole=-0.0433134,-0.0216575,0.0649709,0.0306281,0.0530497,0.0750219\PG=C03V [C3(C1Se1),3SGV(H1)]\\@

1\1\GINC-ORC142\FOpt\RwB97XD\6-311+G(2df,p)\C1H3Se1(1-)\MEISTERP\26-May-2017\0\\# opt freq wb97xd/6-311+g(2df,p)\\Methyl Selenide\\-1,1\C,-0.3945282648,1.9373888796,0.009066974\H,-0.0415815497,0.9029599706,-0.0117823889\H,-0.0415682529,2.4365404754,-0.8971979772\H,-1.4874463475,1.9253615138,-0.0117813896\Se,0.2640249298,2.8687032696,1.6221562036\\Version=EM64L-G16RevA.03\State=1-A1\HF=-2441.5199166\RMSD=4.408e-09\RMSF=9.893e-05\Dipole=-0.3027207,-0.4281023,-0.7414975\Quadrupole=-0.2288287,-0.1144185,0.3432472,0.1618112,0.2802661,0.3963475\PG=C03V [C3(C1Se1),3SGV(H1)]\\@

1\1\GINC-ORC141\FOpt\RwB97XD\6-311++G(3df,3pd)\C1H3Se1(1-)\MEISTERP\26-May-2017\0\\# opt freq wb97xd/6-311++g(3df,3pd)\\Methyl Selenide\\-1,1\C,-0.3948839576,1.936885865,0.0081957243\H,-0.0418184724,0.9042321687,-0.0114348015\H,-0.0418051894,2.4362054021,-0.8959224286\H,-1.4861679293,1.9255621824,-0.0114338032\Se,0.2635727836,2.8680638524,1.6210486967\\Version=EM64L-G16RevA.03\State=1-A1\HF=-2441.5256054\RMSD=3.832e-09\RMSF=1.008e-04\Dipole=-0.2900284,-0.4101531,-0.7104083\Quadrupole=-0.1485794,-0.0742924,0.2228718,0.1050647,0.181978,0.2573501\PG=C03V [C3(C1Se1),3SGV(H1)]\\@

CH_3Se^- M062X

1\1\GINC-ORC264\FOpt\RM062X\6-31G(d)\C1H3Se1(1-)\MEISTERP\01-Aug-2017\0\\# opt freq m062x/6-31g(d)\\Methyl Selenide\\-1,1\C,-0.3916465213,1.9414641907,0.0161256434\H,-0.0435762444,0.9020061556,-0.0155903765\H,-0.0435629637,2.4337195882,-0.8999280072\H,-1.4876807603,1.9231629663,-0.0155893785\Se,0.2653742128,2.8706114019,1.6254611966\\Version=EM64L-G09RevE.01\State=1-A1\HF=-2439.3159123\RMSD=3.954e-09\RMSF=3.950e-05\Dipole=-0.2216403,-0.3134398,-0.5428954\Quadrupole=-0.0424286,-0.021215,0.0636436,0.0300024,0.0519659,0.0734892\PG=C03V [C3(C1Se1),3SGV(H1)]\\@

1\1\GINC-ORC351\FOpt\RM062X\6-311G(d)\C1H3Se1(1-)\MEISTERP\26-May-2017\0\\# opt freq m062x/6-311g(d)\\Methyl Selenide\\-1,1\C,-0.3942274477,1.9378142898,0.0098038086\H,-0.0431484232,0.9026784501,-0.0145036111\H,-0.0431351431,2.4343246046,-0.8988023986\H,-1.487189509,1.9237904081,-0.0145026131\Se,0.2666141107,2.8723648439,1.6284982574\\Version=EM64L-G16RevA.03\State=1-A1\HF=-2441.4746907\RMSD=1.188e-09\RMSF=1.355e-04\Dipole=-0.2474369,-0.3499209,-0.6060829\Quadrupole=-0.0914192,-0.0457113,0.1371305,0.0646451,0.111969,0.1583445\PG=C03V [C3(C1Se1),3SGV(H1)]\\@

1\1\GINC-ORC341\FOpt\RM062X\6-311G(d,p)\C1H3Se1(1-)\MEISTERP\26-May-2017\0\\# opt freq m062x/6-311g(d,p)\\Methyl Selenide\\-1,1\C,-0.3948555787,1.9369259981,0.008265237\H,-0.0423550738,0.9024587912,-0.0133348941\H,-0.0423417821,2.4354465633,-0.898408267\H,-1.4876610412,1.9244651736,-0.0133338952\Se,0.2661238946,2.8716715888,1.6272975004\\Version=EM64L-G16RevA.03\State=1-A1\HF=-2441.4781594\RMSD=3.980e-09\RMSF=1.439e-04\Dipole=-0.2451742,-0.346721,-0.6005405\Quadrupole=-0.0758109,-0.0379068,0.1137177,0.053608,0.0928521,0.1313098\PG=C03V [C3(C1Se1),3SGV(H1)]\\@

1\1\GINC-ORC241\FOpt\RM062X\6-311+G(d,p)\C1H3Se1(1-)\MEISTERP\26-May-2
017\0\\# opt freq m062x/6-311+g(d,p)\\Methyl Selenide\\-1,1\C,-0.39602
4253,1.9352732792,0.0054026345\H,-0.0414052761,0.9026191698,-0.0116912
904\H,-0.0413919741,2.4367897665,-0.8974475694\H,-1.4878264144,1.92541
41132,-0.0116902908\Se,0.2655514551,2.8708620549,1.6258953418\\Version
=EM64L-G16RevA.03\State=1-A1\HF=-2441.4851465\RMSD=3.500e-09\RMSF=1.33
8e-04\Dipole=-0.3101769,-0.4386467,-0.7597609\Quadrupole=-0.2311438,-0
.1155761,0.3467199,0.1634483,0.2831016,0.4003574\PG=C03V [C3(C1Se1),3S
GV(H1)]\\@

1\1\GINC-ORC136\FOpt\RM062X\6-311G(2d,p)\C1H3Se1(1-)\MEISTERP\26-May-2
017\0\\# opt freq m062x/6-311g(2d,p)\\Methyl Selenide\\-1,1\C,-0.39500
74266,1.9367112573,0.0078932939\H,-0.0423052,0.9036718953,-0.012553087
2\H,-0.0422919181,2.4355170788,-0.8969667844\H,-1.486533931,1.92491654
11,-0.012552089\Se,0.2650440432,2.870144482,1.6246524649\\Version=EM64
L-G16RevA.03\State=1-A1\HF=-2441.4777095\RMSD=6.034e-09\RMSF=1.416e-04
\Dipole=-0.2304763,-0.3259355,-0.5645388\Quadrupole=-0.0349812,-0.0174
912,0.0524724,0.0247362,0.0428445,0.0605899\PG=C03V [C3(C1Se1),3SGV(H1
)]\\@

1\1\GINC-ORC321\FOpt\RM062X\6-311G(df,p)\C1H3Se1(1-)\MEISTERP\26-May-2
017\0\\# opt freq m062x/6-311g(df,p)\\Methyl Selenide\\-1,1\C,-0.39415
4502,1.9379174483,0.009982485\H,-0.0425308315,0.9028211357,-0.01341271
2\H,-0.042517545,2.4351980017,-0.8981333771\H,-1.4872608345,1.92442024
16,-0.0134117135\Se,0.2653743299,2.8706115676,1.6254614836\\Version=EM
64L-G16RevA.03\State=1-A1\HF=-2441.4810834\RMSD=7.891e-09\RMSF=1.389e-
04\Dipole=-0.2459992,-0.3478878,-0.6025614\Quadrupole=-0.03226,-0.0161
306,0.0483906,0.0228119,0.0395116,0.0558766\PG=C03V [C3(C1Se1),3SGV(H1
)]\\@

1\1\GINC-ORC134\FOpt\RM062X\6-311+G(2df,p)\C1H3Se1(1-)\MEISTERP\26-May
-2017\0\\# opt freq m062x/6-311+g(2df,p)\\Methyl Selenide\\-1,1\C,-0.3
955204415,1.9359857606,0.0066366925\H,-0.0415050446,0.9040511644,-0.01
1027476\H,-0.0414917563,2.4366486545,-0.8958755188\H,-1.4864430528,1.9
257973551,-0.0110264773\Se,0.2638599001,2.8684698874,1.6217519724\\Ver
sion=EM64L-G16RevA.03\State=1-A1\HF=-2441.4869165\RMSD=8.734e-09\RMSF=
1.322e-04\Dipole=-0.2909483,-0.4114539,-0.7126615\Quadrupole=-0.175403
8,-0.0877051,0.2631089,0.124033,0.2148321,0.3038118\PG=C03V [C3(C1Se1)
,3SGV(H1)]\\@

1\1\GINC-ORC278\FOpt\RM062X\6-311++G(3df,3pd)\C1H3Se1(1-)\MEISTERP\01-
Aug-2017\0\\# opt freq m062x/6-311++g(3df,3pd)\\Methyl Selenide\\-1,1\
C,-0.3958165265,1.9355670424,0.0059114489\H,-0.0419636526,0.9052626981
,-0.0110769224\H,-0.0419503804,2.4360000742,-0.8948510249\H,-1.4851479
391,1.9257687952,-0.0110759249\Se,0.2637715951,2.8683450081,1.62153567
43\\Version=EM64L-G09RevE.01\State=1-A1\HF=-2441.4923797\RMSD=7.546e-0
9\RMSF=1.147e-04\Dipole=-0.2759646,-0.3902642,-0.6759597\Quadrupole=-0
.099589,-0.0497963,0.1493853,0.0704222,0.1219752,0.1724952\PG=C03V [C3
(C1Se1),3SGV(H1)]\\@

CH_3Se^- M08HX

1\1\GINC-ORC241\FOpt\RM08HX\6-31G(d)\C1H3Se1(1-)\MEISTERP\26-May-2017\
0\\# opt freq m08hx/6-31g(d)\\Methyl Selenide\\-1,1\C,-0.3899728556,1.
9438310594,0.0202251941\H,-0.0422682771,0.8984544187,-0.0155050064\H,-
0.0422549495,2.435569365,-0.9029612111\H,-1.491465358,1.9232122897,-0.
0155040048\Se,0.2648828398,2.8699165109,1.6242576061\\Version=EM64L-G1

```
6RevA.03\State=1-A1\HF=-2439.3559102\RMSD=5.272e-09\RMSF=4.915e-05\Dip
ole=-0.2262167,-0.3199118,-0.5541053\Quadrupole=-0.0417249,-0.0208632,
0.0625881,0.0295048,0.0511041,0.0722705\PG=C03V [C3(C1Se1),3SGV(H1)]\\
@

1\1\GINC-ORC136\FOpt\RM08HX\6-311G(d)\C1H3Se1(1-)\MEISTERP\26-May-2017
\0\\# opt freq m08hx/6-311g(d)\\Methyl Selenide\\-1,1\C,-0.3930800241,
1.9394369562,0.0126143585\H,-0.0414079005,0.8990221299,-0.0137722609\H
,-0.0413945674,2.4367861032,-0.9016031825\H,-1.4912168862,1.9242126917
,-0.0137712589\Se,0.2660191933,2.871523522,1.6270410403\\Version=EM64L
-G16RevA.03\State=1-A1\HF=-2441.4952524\RMSD=1.906e-09\RMSF=1.242e-04\
Dipole=-0.2564419,-0.3626556,-0.6281401\Quadrupole=-0.1047241,-0.05236
39,0.157088,0.0740533,0.1282646,0.1813895\PG=C03V [C3(C1Se1),3SGV(H1)]
\\@

1\1\GINC-ORC341\FOpt\RM08HX\6-311G(d,p)\C1H3Se1(1-)\MEISTERP\26-May-20
17\0\\# opt freq m08hx/6-311g(d,p)\\Methyl Selenide\\-1,1\C,-0.3936701
124,1.9386024636,0.0111689702\H,-0.0407858716,0.8990469268,-0.01274217
67\H,-0.040772531,2.437665777,-0.9010666631\H,-1.4914008381,1.92480741
47,-0.0127411741\Se,0.2655457201,2.8708539446,1.6258812943\\Version=EM
64L-G16RevA.03\State=1-A1\HF=-2441.4991529\RMSD=5.522e-09\RMSF=1.377e-
04\Dipole=-0.2537958,-0.3589136,-0.6216587\Quadrupole=-0.0907756,-0.04
53894,0.136165,0.06419,0.1111806,0.1572297\PG=C03V [C3(C1Se1),3SGV(H1)
]\\@

1\1\GINC-ORC241\FOpt\RM08HX\6-311+G(d,p)\C1H3Se1(1-)\MEISTERP\26-May-2
017\0\\# opt freq m08hx/6-311+g(d,p)\\Methyl Selenide\\-1,1\C,-0.39501
51406,1.9367003484,0.007874399\H,-0.0397229257,0.8991560661,-0.0109433
851\H,-0.0397095731,2.4391689951,-0.9000727456\H,-1.4916522343,1.92584
59531,-0.0109423816\Se,0.2650084469,2.8700941423,1.6245652737\\Version
=EM64L-G16RevA.03\State=1-A1\HF=-2441.5064397\RMSD=2.117e-09\RMSF=1.43
0e-04\Dipole=-0.3160952,-0.4470163,-0.7742575\Quadrupole=-0.2300379,-0
.1150231,0.3450611,0.1626663,0.2817472,0.3984419\PG=C03V [C3(C1Se1),3S
GV(H1)]\\@

1\1\GINC-ORC164\FOpt\RM08HX\6-311G(2d,p)\C1H3Se1(1-)\MEISTERP\26-May-2
017\0\\# opt freq m08hx/6-311g(2d,p)\\Methyl Selenide\\-1,1\C,-0.39375
39885,1.9384838476,0.0109635206\H,-0.0407969026,0.9003963448,-0.011981
1286\H,-0.0407835739,2.4376501589,-0.8995175089\H,-1.4901249085,1.9252
467957,-0.0119801269\Se,0.2643705919,2.8691920988,1.6230028834\\Versio
n=EM64L-G16RevA.03\State=1-A1\HF=-2441.4990944\RMSD=4.294e-09\RMSF=1.3
79e-04\Dipole=-0.2357835,-0.3334409,-0.5775384\Quadrupole=-0.0483972,-
0.0241995,0.0725966,0.034223,0.0592762,0.0838273\PG=C03V [C3(C1Se1),3S
GV(H1)]\\@

1\1\GINC-ORC351\FOpt\RM08HX\6-311G(df,p)\C1H3Se1(1-)\MEISTERP\26-May-2
017\0\\# opt freq m08hx/6-311g(df,p)\\Methyl Selenide\\-1,1\C,-0.39293
20018,1.9396462868,0.012976931\H,-0.0410183676,0.8993524669,-0.0129454
429\H,-0.0410050326,2.4373369767,-0.9009036917\H,-1.4910352759,1.92469
00552,-0.0129444407\Se,0.2649075015,2.8699513871,1.6243180136\\Version
=EM64L-G16RevA.03\State=1-A1\HF=-2441.5024528\RMSD=1.538e-09\RMSF=1.27
2e-04\Dipole=-0.2521789,-0.356627,-0.6176982\Quadrupole=-0.041593,-0.0
207973,0.0623903,0.0294116,0.0509425,0.072042\PG=C03V [C3(C1Se1),3SGV(
H1)]\\@

1\1\GINC-ORC247\FOpt\RM08HX\6-311+G(2df,p)\C1H3Se1(1-)\MEISTERP\26-May
-2017\0\\# opt freq m08hx/6-311+g(2df,p)\\Methyl Selenide\\-1,1\C,-0.3
```

94427472,1.9375314189,0.0093138602\H,-0.0399455116,0.9006347053,-0.010
453201\H,-0.0399321745,2.438854194,-0.8985471153\H,-1.4901839588,1.926
1289484,-0.0104521987\Se,0.2633941495,2.8678112312,1.6206111425\\Versi
on=EM64L-G16RevA.03\State=1-A1\HF=-2441.5088882\RMSD=8.895e-09\RMSF=1.
330e-04\Dipole=-0.2914634,-0.4121824,-0.7139232\Quadrupole=-0.1697084,
-0.0848573,0.2545657,0.1200056,0.2078564,0.2939469\PG=C03V [C3(C1Se1),
3SGV(H1)]\\@

1\1\GINC-ORC134\FOpt\RM08HX\6-311++G(3df,3pd)\C1H3Se1(1-)\MEISTERP\26-
May-2017\0\\# opt freq m08hx/6-311++g(3df,3pd)\\Methyl Selenide\\-1,1\
C,-0.3951869767,1.9364573407,0.007453496\H,-0.0402040053,0.9025898874,
-0.0097465307\H,-0.0401906883,2.4384886057,-0.8965005439\H,-1.48825442
24,1.9265369282,-0.0097455299\Se,0.2627341068,2.8668778103,1.618994404
8\\Version=EM64L-G16RevA.03\State=1-A1\HF=-2441.514471\RMSD=7.893e-09\
RMSF=1.327e-04\Dipole=-0.2789693,-0.3945136,-0.6833198\Quadrupole=-0.0
963896,-0.0481965,0.1445861,0.0681598,0.1180566,0.1669535\PG=C03V [C3(
C1Se1),3SGV(H1)]\\@

CH_3Se^- QCISD

1\1\GINC-ORC113\FOpt\RQCISD-FC\6-311+G(2df,p)\C1H3Se1(1-)\MEISTERP\21-
Aug-2017\0\\# opt freq=noraman qcisd/6-311+g(2df,p) guess=read\\Methyl
Selenide\\-1,1\C,0.,0.,-1.543056\H,0.0000002965,1.023824,-1.93075\H,-
0.8866577413,-0.5119117432,-1.93075\H,0.8866574448,-0.5119122568,-1.93
075\Se,0.,0.,0.442664\\Version=EM64L-G09RevE.01\State=1-A1\HF=-2439.44
07893\MP2=-2439.7766691\MP3=-2439.8034515\MP4D=-2439.8121836\MP4DQ=-24
39.8031088\MP4SDQ=-2439.805298\QCISD=-2439.8051314\RMSD=3.010e-09\RMSF
=1.248e-04\Dipole=0.,0.,-0.8817766\PG=C03V [C3(C1Se1),3SGV(H1)]\\@

CH_3SeSeH B3LYP

1\1\GINC-ORC391\FOpt\RB3LYP\6-31G(d)\C1H4Se2\MEISTERP\20-Jul-2017\0\\#
opt=readfc freq b3lyp/6-31g(d)\\Methyl Se Se optimization and frequen
cy\\0,1\C,1.5635251228,1.2735134326,0.0488772484\H,1.2062354158,1.9858
653585,-0.6942806995\H,2.6566463845,1.2828569429,0.0827347902\H,1.1576
802614,1.4952896019,1.0357497636\Se,-1.2648248264,-0.2667755828,-0.703
9944743\Se,1.044426395,-0.5565849536,-0.4888748585\H,-1.6276157531,-0.
5411497995,0.7201922301\\Version=EM64L-G09RevE.01\State=1-A\HF=-4839.2
868317\RMSD=2.344e-09\RMSF=2.278e-05\Dipole=0.1993198,0.4624328,0.3902
03\Quadrupole=1.3972342,-0.8102845,-0.5869497,1.4519049,-0.4103711,0.1
956224\PG=C01 [X(C1H4Se2)]\\@

1\1\GINC-ORC391\FOpt\RB3LYP\6-311G(d)\C1H4Se2\MEISTERP\20-Jul-2017\0\\
opt=readfc freq b3lyp/6-311g(d)\\Methyl Se Se optimization and frequ
ency\\0,1\C,1.5719760483,1.2806620316,0.0468548921\H,1.2318872421,1.99
8383373,-0.6958487569\H,2.6630241783,1.2766654863,0.0840989122\H,1.170
8156673,1.5204696249,1.0289886673\Se,-1.296619142,-0.2815701617,-0.698
0066351\Se,1.0404253638,-0.5500736941,-0.4851391143\H,-1.645436358,-0.
5715216599,0.7194560348\\Version=EM64L-G09RevE.01\State=1-A\HF=-4843.6
366298\RMSD=4.046e-09\RMSF=4.956e-06\Dipole=0.1934502,0.4581399,0.3865
409\Quadrupole=1.5776542,-0.9308375,-0.6468167,1.4850957,-0.4041151,0.
1340367\PG=C01 [X(C1H4Se2)]\\@

1\1\GINC-ORC390\FOpt\RB3LYP\6-311G(d,p)\C1H4Se2\MEISTERP\20-Jul-2017\0
\\# opt=readfc freq b3lyp/6-311g(d,p)\\Methyl Se Se optimization and f
requency\\0,1\C,1.571911714,1.2828988063,0.0470630405\H,1.2325922311,1
.9965965705,-0.6998483927\H,2.6629251221,1.2727780166,0.0846697744\H,1

.1682474856,1.5218306842,1.0283322718\Se,-1.2987616829,-0.2823632679,-
0.6934006577\Se,1.0404869942,-0.5479429257,-0.4816739296\H,-1.64132886
41,-0.570782884,0.7152618934\\Version=EM64L-G09RevE.01\State=1-A\HF=-4
843.6445692\RMSD=9.230e-09\RMSF=4.104e-06\Dipole=0.1916823,0.4572718,0
.3909749\Quadrupole=1.5919129,-0.8950427,-0.6968703,1.5061827,-0.41012
32,0.1461292\PG=C01 [X(C1H4Se2)]\\@

1\1\GINC-ORC390\FOpt\RB3LYP\6-311+G(d,p)\C1H4Se2\MEISTERP\20-Jul-2017\
0\\# opt=readfc freq b3lyp/6-311+g(d,p)\\Methyl Se Se optimization and
frequency\\0,1\C,1.5720364545,1.2833288507,0.0472650455\H,1.231936843
4,1.9965952507,-0.6999107891\H,2.6632300521,1.272996194,0.0831967245\H
,1.1700505293,1.5232925329,1.0290831799\Se,-1.2984727885,-0.2831053458
,-0.6940313587\Se,1.0406082539,-0.5480417451,-0.4792565608\H,-1.643316
3448,-0.5720507375,0.7140577586\\Version=EM64L-G09RevE.01\State=1-A\HF
=-4843.6453675\RMSD=8.924e-09\RMSF=4.300e-06\Dipole=0.1939355,0.467318
8,0.3962318\Quadrupole=1.5885075,-0.9168558,-0.6716516,1.5627761,-0.42
83819,0.1353011\PG=C01 [X(C1H4Se2)]\\@

1\1\GINC-ORC389\FOpt\RB3LYP\6-311G(2d,p)\C1H4Se2\MEISTERP\20-Jul-2017\
0\\# opt=readfc freq b3lyp/6-311g(2d,p)\\Methyl Se Se optimization and
frequency\\0,1\C,1.5714095859,1.2828473469,0.0466712329\H,1.234739869
1,1.9950251109,-0.7011845028\H,2.6612423511,1.2715467222,0.0875197372\
H,1.164502521,1.5204529706,1.0255426179\Se,-1.2997409522,-0.2798851741
,-0.6917345937\Se,1.0415719464,-0.5449168314,-0.4819479962\H,-1.637652
3213,-0.5720551452,0.7155375047\\Version=EM64L-G09RevE.01\State=1-A\HF
=-4843.6404933\RMSD=7.496e-09\RMSF=4.764e-06\Dipole=0.2056558,0.453774
,0.3541399\Quadrupole=1.6855724,-0.9515326,-0.7340398,1.4284752,-0.300
9339,0.1533143\PG=C01 [X(C1H4Se2)]\\@

1\1\GINC-ORC389\FOpt\RB3LYP\6-311G(df,p)\C1H4Se2\MEISTERP\20-Jul-2017\
0\\# opt=readfc freq b3lyp/6-311g(df,p)\\Methyl Se Se optimization and
frequency\\0,1\C,1.5694097506,1.280591211,0.046544916\H,1.2320089907,
1.9969807819,-0.6986717718\H,2.6601693603,1.2690062853,0.0849878171\H,
1.1658937729,1.5201079143,1.0277473294\Se,-1.290354172,-0.2819825008,-
0.6929319122\Se,1.0365332103,-0.5431462189,-0.4834351957\H,-1.63758791
27,-0.5685424728,0.7161628171\\Version=EM64L-G09RevE.01\State=1-A\HF=-
4843.6509529\RMSD=8.377e-09\RMSF=4.134e-06\Dipole=0.1934994,0.4567654,
0.3895066\Quadrupole=1.4241085,-0.8198253,-0.6042831,1.5347584,-0.4181
689,0.144107\PG=C01 [X(C1H4Se2)]\\@

1\1\GINC-ORC141\FOpt\RB3LYP\6-311+G(2df,p)\C1H4Se2\MEISTERP\20-Jul-201
7\0\\# opt=readfc freq b3lyp/6-311+g(2df,p)\\Methyl Se Se optimization
and frequency\\0,1\C,1.5688323009,1.2808145689,0.0462148073\H,1.23352
40999,1.9954792656,-0.7003271092\H,2.6586898349,1.2689660373,0.0869816
243\H,1.1633654411,1.5207362155,1.025432598\Se,-1.2892580974,-0.280950
5418,-0.6915006424\Se,1.0368354568,-0.541861497,-0.481575396\H,-1.6359
160362,-0.5701690485,0.7151781179\\Version=EM64L-G09RevE.01\State=1-A\
HF=-4843.6476357\RMSD=5.691e-09\RMSF=3.923e-06\Dipole=0.2089532,0.4559
471,0.3582034\Quadrupole=1.4960961,-0.8801666,-0.6159295,1.5177864,-0.
3303471,0.1474021\PG=C01 [X(C1H4Se2)]\\@

1\1\GINC-ORC122\FOpt\RB3LYP\6-311++G(3df,3pd)\C1H4Se2\MEISTERP\20-Jul-
2017\0\\# opt=readfc freq b3lyp/6-311++g(3df,3pd)\\Methyl Se Se optimi
zation and frequency\\0,1\C,1.569085445,1.2814325867,0.0463942247\H,1.
2335748129,1.9943631971,-0.699195177\H,2.6572034769,1.268915683,0.0866
57552\H,1.1638194905,1.5192962943,1.0242683376\Se,-1.2897468994,-0.280
4911714,-0.690878152\Se,1.0370022351,-0.5408557385,-0.4821113401\H,-1.

6348655609,-0.5696458511,0.7152685547\\Version=EM64L-G09RevE.01\State=
1-A\HF=-4843.6570228\RMSD=5.305e-09\RMSF=3.651e-06\Dipole=0.2111538,0.
4478834,0.3478259\Quadrupole=1.4533633,-0.8472896,-0.6060737,1.4886332
,-0.3001216,0.1472123\PG=C01 [X(C1H4Se2)]\\@

CH_3SeSeH B3PW91

1\1\GINC-ORC121\FOpt\RB3PW91\6-31G(d)\C1H4Se2\MEISTERP\20-Jul-2017\0\\
opt=readfc freq b3pw91/6-31g(d)\\Methyl Se Se optimization and frequ
ency\\0,1\C,1.5556046608,1.2662197986,0.0483524355\H,1.1998408701,1.98
06483605,-0.6941693903\H,2.6490979224,1.2763211787,0.0814921391\H,1.15
25279486,1.4905074574,1.0362109768\Se,-1.247940601,-0.260189741,-0.703
8207754\Se,1.0387885473,-0.5484671688,-0.484329801\H,-1.6118463481,-0.
5320248854,0.7166684153\\Version=EM64L-G09RevE.01\State=1-A\HF=-4839.2
341679\RMSD=3.082e-09\RMSF=3.264e-06\Dipole=0.1987982,0.4790318,0.4130
815\Quadrupole=1.4037716,-0.8254524,-0.5783192,1.510279,-0.4518748,0.1
964931\PG=C01 [X(C1H4Se2)]\\@

1\1\GINC-ORC116\FOpt\RB3PW91\6-311G(d)\C1H4Se2\MEISTERP\20-Jul-2017\0\
\# opt=readfc freq b3pw91/6-311g(d)\\Methyl Se Se optimization and fre
quency\\0,1\C,1.5634127479,1.2730594461,0.0462745997\H,1.2254971517,1.
9937015804,-0.6958612726\H,2.655275186,1.2694013094,0.0832533952\H,1.1
647999344,1.515533529,1.0297271254\Se,-1.2778304079,-0.2730257984,-0.6
975086515\Se,1.034522605,-0.5423996171,-0.4819737823\H,-1.6296042171,-
0.5632554494,0.7164925861\\Version=EM64L-G09RevE.01\State=1-A\HF=-4843
.5828595\RMSD=7.891e-09\RMSF=4.585e-06\Dipole=0.1918546,0.4713092,0.40
85269\Quadrupole=1.5640064,-0.9247675,-0.639239,1.5378699,-0.4464579,0
.1347518\PG=C01 [X(C1H4Se2)]\\@

1\1\GINC-ORC114\FOpt\RB3PW91\6-311G(d,p)\C1H4Se2\MEISTERP\20-Jul-2017\
0\\# opt=readfc freq b3pw91/6-311g(d,p)\\Methyl Se Se optimization and
 frequency\\0,1\C,1.5633654842,1.2753003307,0.046502307\H,1.2261630561
,1.9922130838,-0.6996196234\H,2.655240911,1.2653130983,0.0834584217\H,
1.1627146703,1.5168247025,1.0293541549\Se,-1.28013949,-0.2740591125,-0
.6931189453\Se,1.0343239672,-0.5399569914,-0.4786438718\H,-1.625595598
7,-0.5626201114,0.7124715569\\Version=EM64L-G09RevE.01\State=1-A\HF=-4
843.5907792\RMSD=6.030e-09\RMSF=6.099e-06\Dipole=0.1906134,0.4703813,0
.4114721\Quadrupole=1.5766775,-0.8883961,-0.6882814,1.557683,-0.447075
5,0.147543\PG=C01 [X(C1H4Se2)]\\@

1\1\GINC-ORC109\FOpt\RB3PW91\6-311+G(d,p)\C1H4Se2\MEISTERP\20-Jul-2017
\0\\# opt=readfc freq b3pw91/6-311+g(d,p)\\Methyl Se Se optimization a
nd frequency\\0,1\C,1.5634506987,1.2755374276,0.0465649411\H,1.2260057
184,1.9923685329,-0.6997445528\H,2.6554925114,1.2653819019,0.082527155
4\H,1.1639825017,1.5181780766,1.029751175\Se,-1.2800148515,-0.27448754
94,-0.6934408705\Se,1.0342594508,-0.5399737102,-0.4768798904\H,-1.6271
030295,-0.5639896793,0.7116260423\\Version=EM64L-G09RevE.01\State=1-A\
HF=-4843.5914345\RMSD=5.667e-09\RMSF=5.296e-06\Dipole=0.1920228,0.4771
932,0.4135189\Quadrupole=1.5714314,-0.9053132,-0.6661182,1.6017833,-0.
4596503,0.1376763\PG=C01 [X(C1H4Se2)]\\@

1\1\GINC-ORC108\FOpt\RB3PW91\6-311G(2d,p)\C1H4Se2\MEISTERP\20-Jul-2017
\0\\# opt=readfc freq b3pw91/6-311g(2d,p)\\Methyl Se Se optimization a
nd frequency\\0,1\C,1.5628670705,1.2752129567,0.0460871646\H,1.2283809
28,1.9905410613,-0.7012768517\H,2.6537327906,1.2641035839,0.0868627781
\H,1.1581718143,1.5148627527,1.0265230236\Se,-1.2815243685,-0.27113046
56,-0.6914120017\Se,1.0356158333,-0.5371159987,-0.4796028888\H,-1.6211

710682,-0.5634588903,0.7132227759\\Version=EM64L-G09RevE.01\State=1-A\
HF=-4843.586501\RMSD=5.500e-09\RMSF=6.419e-06\Dipole=0.2054369,0.46729
26,0.3713183\Quadrupole=1.693369,-0.9580972,-0.7352718,1.4730363,-0.32
48818,0.1574984\PG=C01 [X(C1H4Se2)]\\@

1\1\GINC-ORC96\FOpt\RB3PW91\6-311G(df,p)\C1H4Se2\MEISTERP\20-Jul-2017\
0\\# opt=readfc freq b3pw91/6-311g(df,p)\\Methyl Se Se optimization an
d frequency\\0,1\C,1.5604190845,1.2737294827,0.0443833243\H,1.22533591
67,1.9900082345,-0.7033410176\H,2.6520296742,1.2632405172,0.0833747951
\H,1.1585710513,1.5194723397,1.025725748\Se,-1.2704451997,-0.277690355
2,-0.6905349805\Se,1.0319533738,-0.536967296,-0.4752638755\H,-1.621790
9008,-0.5587779229,0.7160600063\\Version=EM64L-G09RevE.01\State=1-A\HF
=-4843.5974043\RMSD=5.408e-09\RMSF=1.454e-05\Dipole=0.192329,0.4718676
,0.4086605\Quadrupole=1.407898,-0.8086235,-0.5992745,1.5870676,-0.4607
393,0.1489196\PG=C01 [X(C1H4Se2)]\\@

1\1\GINC-ORC94\FOpt\RB3PW91\6-311+G(2df,p)\C1H4Se2\MEISTERP\20-Jul-201
7\0\\# opt=readfc freq b3pw91/6-311+g(2df,p)\\Methyl Se Se optimizatio
n and frequency\\0,1\C,1.5602676576,1.272979492,0.045608432\H,1.226456
3724,1.9907041914,-0.7002592423\H,2.6511905929,1.2619586445,0.08603882
81\H,1.1570782244,1.5147296244,1.0265127317\Se,-1.270667575,-0.2718999
486,-0.691131776\Se,1.0313335223,-0.5345954856,-0.4791745247\H,-1.6195
857946,-0.5608615182,0.7128095512\\Version=EM64L-G09RevE.01\State=1-A\
HF=-4843.5937059\RMSD=4.428e-09\RMSF=1.315e-05\Dipole=0.2082105,0.4664
865,0.3723778\Quadrupole=1.4999616,-0.877825,-0.6221366,1.5511068,-0.3
50363,0.1543713\PG=C01 [X(C1H4Se2)]\\@

1\1\GINC-ORC87\FOpt\RB3PW91\6-311++G(3df,3pd)\C1H4Se2\MEISTERP\20-Jul-
2017\0\\# opt=readfc freq b3pw91/6-311++g(3df,3pd)\\Methyl Se Se optim
ization and frequency\\0,1\C,1.5605766749,1.2734770398,0.0457835816\H,
1.2266543501,1.9896461091,-0.6993543743\H,2.6500057463,1.2619389826,0.
0859269836\H,1.1573264065,1.5135031231,1.0254175008\Se,-1.2712663853,-
0.2712796181,-0.6907009142\Se,1.0311419631,-0.5335220754,-0.4796438819
\H,-1.6183657556,-0.5607485611,0.7129751044\\Version=EM64L-G09RevE.01\
State=1-A\HF=-4843.6031552\RMSD=3.209e-09\RMSF=4.902e-06\Dipole=0.2101
219,0.4575069,0.3625761\Quadrupole=1.4644718,-0.8511441,-0.6133277,1.5
20242,-0.3225998,0.1523998\PG=C01 [X(C1H4Se2)]\\@

CH_3SeSeH wB97XD

1\1\GINC-ORC48\FOpt\RwB97XD\6-31G(d)\C1H4Se2\MEISTERP\20-Jul-2017\0\\#
opt=readfc freq wb97xd/6-31g(d)\\Methyl Se Se optimization and freque
ncy\\0,1\C,1.5492248188,1.2585073234,0.0485748013\H,1.1868247621,1.971
6305747,-0.6913176649\H,2.6415862423,1.2805037499,0.0813239307\H,1.145
3900922,1.4804178382,1.0361156788\Se,-1.2360677925,-0.253720733,-0.705
0986535\Se,1.0426762659,-0.5528303157,-0.4827884069\H,-1.5935613888,-0
.5114934375,0.7135943145\\Version=EM64L-G09RevE.01\State=1-A\HF=-4839.
3065392\RMSD=6.159e-09\RMSF=2.922e-05\Dipole=0.1940736,0.4808899,0.418
8875\Quadrupole=1.3877074,-0.8427727,-0.5449347,1.5003377,-0.4856233,0
.2194525\PG=C01 [X(C1H4Se2)]\\@

1\1\GINC-ORC42\FOpt\RwB97XD\6-311G(d)\C1H4Se2\MEISTERP\20-Jul-2017\0\\
opt=readfc freq wb97xd/6-311g(d)\\Methyl Se Se optimization and freq
uency\\0,1\C,1.5565075133,1.2647472128,0.046245965\H,1.2128264728,1.98
40439266,-0.6941807217\H,2.6475322145,1.2745927354,0.0854404253\H,1.15
40588466,1.5041940024,1.0285531984\Se,-1.2646455398,-0.2657611526,-0.6
980469979\Se,1.0394068827,-0.5475308731,-0.4821527147\H,-1.6096133902,

-0.5412708515,0.7145448456\\Version=EM64L-G09RevE.01\State=1-A\HF=-484
3.668014\RMSD=1.897e-09\RMSF=8.585e-06\Dipole=0.1865696,0.4712375,0.41
26333\Quadrupole=1.5600613,-0.950343,-0.6097183,1.5177217,-0.4813491,0
.1615075\PG=C01 [X(C1H4Se2)]\\@

1\1\GINC-ORC25\FOpt\RwB97XD\6-311G(d,p)\C1H4Se2\MEISTERP\20-Jul-2017\0
\\# opt=readfc freq wb97xd/6-311g(d,p)\\Methyl Se Se optimization and
frequency\\0,1\C,1.5566545839,1.2668387564,0.0466076167\H,1.2122428488
,1.9822458034,-0.6972528476\H,2.647762265,1.2718448195,0.0843676507\H,
1.1534704601,1.5056863692,1.0287225762\Se,-1.2660001734,-0.2671705788,
-0.6940084426\Se,1.0398017377,-0.5457448104,-0.4777819941\H,-1.6078587
221,-0.5406853593,0.7097494407\\Version=EM64L-G09RevE.01\State=1-A\HF=
-4843.6758565\RMSD=6.300e-09\RMSF=1.032e-05\Dipole=0.1864026,0.4699018
,0.4134792\Quadrupole=1.5744892,-0.9091512,-0.665338,1.53328,-0.476280
4,0.1752445\PG=C01 [X(C1H4Se2)]\\@

1\1\GINC-ORC17\FOpt\RwB97XD\6-311+G(d,p)\C1H4Se2\MEISTERP\20-Jul-2017\
0\\# opt=readfc freq wb97xd/6-311+g(d,p)\\Methyl Se Se optimization an
d frequency\\0,1\C,1.5566865298,1.2670854088,0.0465510267\H,1.21281851
64,1.9826364345,-0.6976169678\H,2.6479127769,1.2715734632,0.0841480567
\H,1.1540179987,1.5069717485,1.0287302528\Se,-1.266124687,-0.267641006
2,-0.694022687\Se,1.0395940314,-0.545492177,-0.476766544\H,-1.60883216
61,-0.5421188718,0.7093808624\\Version=EM64L-G09RevE.01\State=1-A\HF=-
4843.6765121\RMSD=5.262e-09\RMSF=9.737e-06\Dipole=0.1888973,0.4772115,
0.4177726\Quadrupole=1.5806328,-0.9276119,-0.653021,1.5893622,-0.48227
63,0.1711735\PG=C01 [X(C1H4Se2)]\\@

1\1\GINC-ORC16\FOpt\RwB97XD\6-311G(2d,p)\C1H4Se2\MEISTERP\20-Jul-2017\
0\\# opt=readfc freq wb97xd/6-311g(2d,p)\\Methyl Se Se optimization an
d frequency\\0,1\C,1.5561530313,1.2666946048,0.0462421963\H,1.21420831
65,1.981121517,-0.698273321\H,2.6463281333,1.2706899628,0.0866554837\H
,1.1505197829,1.5043070737,1.0264953828\Se,-1.2669271899,-0.2656694015
,-0.6926722639\Se,1.0406428506,-0.5427104166,-0.4783086828\H,-1.604851
9247,-0.5414183402,0.7102652049\\Version=EM64L-G09RevE.01\State=1-A\HF
=-4843.6714624\RMSD=6.365e-09\RMSF=1.367e-05\Dipole=0.1976711,0.467198
6,0.3752916\Quadrupole=1.7089407,-0.9874729,-0.7214678,1.4517057,-0.35
4832,0.1858196\PG=C01 [X(C1H4Se2)]\\@

1\1\GINC-ORC12\FOpt\RwB97XD\6-311G(df,p)\C1H4Se2\MEISTERP\20-Jul-2017\
0\\# opt=readfc freq wb97xd/6-311g(df,p)\\Methyl Se Se optimization an
d frequency\\0,1\C,1.5545122286,1.2651000107,0.0461510288\H,1.21157557
8,1.9826692987,-0.6963416855\H,2.6454141273,1.2686390674,0.0844579333\
H,1.1514546887,1.5045335967,1.0282026124\Se,-1.2577441876,-0.268229149
4,-0.6937138297\Se,1.0359844591,-0.5413271035,-0.4789879022\H,-1.60512
3894,-0.5383707205,0.7106358429\\Version=EM64L-G09RevE.01\State=1-A\HF
=-4843.6827578\RMSD=6.228e-09\RMSF=1.016e-05\Dipole=0.1879008,0.469971
8,0.4126418\Quadrupole=1.4037993,-0.8313009,-0.5724984,1.5689574,-0.48
83131,0.1747063\PG=C01 [X(C1H4Se2)]\\@

1\1\GINC-ORC5\FOpt\RwB97XD\6-311+G(2df,p)\C1H4Se2\MEISTERP\20-Jul-2017
\0\\# opt=readfc freq wb97xd/6-311+g(2df,p)\\Methyl Se Se optimization
and frequency\\0,1\C,1.55405138,1.2649724896,0.0455983045\H,1.2141990
205,1.982271202,-0.6975557836\H,2.6442141626,1.2679879686,0.0868594685
\H,1.1489984004,1.5044920308,1.0259377966\Se,-1.2577748911,-0.26738820
35,-0.6919384113\Se,1.0356696593,-0.5395790484,-0.4792896775\H,-1.6032
847317,-0.5397414392,0.7107923027\\Version=EM64L-G09RevE.01\State=1-A\
HF=-4843.6790073\RMSD=5.085e-09\RMSF=1.276e-05\Dipole=0.2016783,0.4672

332,0.3784122\Quadrupole=1.5184255,-0.9060203,-0.6124052,1.5489659,-0.
377361,0.1874907\PG=C01 [X(C1H4Se2)]\\@

1\1\GINC-ORC195\FOpt\RwB97XD\6-311++G(3df,3pd)\C1H4Se2\MEISTERP\20-Jul
-2017\0\\# opt=readfc freq wb97xd/6-311++g(3df,3pd)\\Methyl Se Se opti
mization and frequency\\0,1\C,1.5543952724,1.2652264396,0.046677109\H,
1.2092047767,1.9795111297,-0.6943494752\H,2.6429900579,1.2701494624,0.
0821037586\H,1.1534831871,1.5021225661,1.0273770329\Se,-1.2566730602,-
0.2673868481,-0.693631435\Se,1.036851796,-0.540069327,-0.4763116043\H,
-1.6041790299,-0.5365384227,0.708538614\\Version=EM64L-G09RevE.01\Stat
e=1-A\HF=-4843.6888505\RMSD=6.351e-09\RMSF=1.542e-05\Dipole=0.2028739,
0.4621688,0.3707084\Quadrupole=1.4879886,-0.880546,-0.6074426,1.531748
,-0.3566187,0.1913101\PG=C01 [X(C1H4Se2)]\\@

CH_3SeSeH M062X

1\1\GINC-ORC84\FOpt\RM062X\6-31G(d)\C1H4Se2\MEISTERP\20-Jul-2017\0\\#
opt=readfc freq m062x/6-31g(d)\\Methyl Se Se optimization and frequenc
y\\0,1\C,1.54511471,1.2578605896,-0.0459758564\H,1.1130058028,1.476585
8147,-1.0208553984\H,2.6350759397,1.2781165043,-0.1067861761\H,1.19901
1014,1.9654100184,0.7052511769\Se,-1.2292953215,-0.236886777,0.6962744
379\Se,1.04605679,-0.5526307442,0.4950802167\H,-1.5728959351,-0.515440
4059,-0.7225844006\\Version=EM64L-G09RevE.01\State=1-A\HF=-4839.338804
8\RMSD=4.212e-09\RMSF=1.802e-05\Dipole=0.1941431,0.4823522,-0.4339893\
Quadrupole=1.3326391,-0.8311417,-0.5014974,1.5348332,0.5120918,-0.1858
507\PG=C01 [X(C1H4Se2)]\\@

1\1\GINC-ORC73\FOpt\RM062X\6-311G(d)\C1H4Se2\MEISTERP\20-Jul-2017\0\\#
opt=readfc freq m062x/6-311g(d)\\Methyl Se Se optimization and freque
ncy\\0,1\C,1.5526557822,1.2647925564,-0.0441314021\H,1.1239444694,1.50
11915219,-1.0143359903\H,2.6408731478,1.2732961726,-0.1093805013\H,1.2
229638585,1.9774614455,0.7069418623\Se,-1.2586025837,-0.2504282758,0.6
91185939\Se,1.0435359744,-0.5500343044,0.4919662675\H,-1.5892976485,-0
.5432641162,-0.7218421751\\Version=EM64L-G09RevE.01\State=1-A\HF=-4843
.6051701\RMSD=6.203e-09\RMSF=2.329e-05\Dipole=0.1890796,0.4787321,-0.4
360975\Quadrupole=1.5062863,-0.9379456,-0.5683407,1.5601212,0.5173839,
-0.1314741\PG=C01 [X(C1H4Se2)]\\@

1\1\GINC-ORC67\FOpt\RM062X\6-311G(d,p)\C1H4Se2\MEISTERP\20-Jul-2017\0\
\# opt=readfc freq m062x/6-311g(d,p)\\Methyl Se Se optimization and fr
equency\\0,1\C,1.5531756908,1.266994813,-0.044684643\H,1.1247130085,1.
50231796,-1.0152255168\H,2.6416095811,1.2711206467,-0.1070743503\H,1.2
212880643,1.9755164163,0.7093571396\Se,-1.2596610673,-0.2512747082,0.6
876310565\Se,1.04398059,-0.548521895,0.4870793573\H,-1.5890328674,-0.5
431382327,-0.7166790432\\Version=EM64L-G09RevE.01\State=1-A\HF=-4843.6
116164\RMSD=5.219e-09\RMSF=2.343e-05\Dipole=0.188767,0.4794685,-0.4403
82\Quadrupole=1.5225017,-0.902434,-0.6200677,1.5858219,0.5229961,-0.14
20155\PG=C01 [X(C1H4Se2)]\\@

1\1\GINC-ORC65\FOpt\RM062X\6-311+G(d,p)\C1H4Se2\MEISTERP\20-Jul-2017\0
\\# opt=readfc freq m062x/6-311+g(d,p)\\Methyl Se Se optimization and
frequency\\0,1\C,1.5533315954,1.2671938793,-0.0448800011\H,1.126516445
5,1.5034580936,-1.0160231269\H,2.6419836167,1.2712639192,-0.1056285487
\H,1.2205747415,1.9756155035,0.7090680211\Se,-1.2595543815,-0.25205386
55,0.6883036865\Se,1.0438048429,-0.5485194031,0.4852093768\H,-1.590583
8604,-0.5439431271,-0.7156454076\\Version=EM64L-G09RevE.01\State=1-A\H
F=-4843.6122188\RMSD=4.772e-09\RMSF=2.314e-05\Dipole=0.1906975,0.48275

86,-0.4416143\Quadrupole=1.5180401,-0.9205384,-0.5975017,1.646901,0.53
0699,-0.1372103\PG=C01 [X(C1H4Se2)]\\@

1\1\GINC-ORC60\FOpt\RM062X\6-311G(2d,p)\C1H4Se2\MEISTERP\20-Jul-2017\0
\\# opt=readfc freq m062x/6-311g(2d,p)\\Methyl Se Se optimization and
frequency\\0,1\C,1.5529095123,1.2670196123,-0.044646498\H,1.1226192783
,1.4996001563,-1.0137449907\H,2.6402939171,1.2705489581,-0.1079650214\
H,1.2212734327,1.9736294823,0.7096800436\Se,-1.2610649396,-0.249602094
5,0.6871952403\Se,1.0457608026,-0.5458322264,0.4867191602\H,-1.5857190
034,-0.5423488881,-0.7168339341\\Version=EM64L-G09RevE.01\State=1-A\HF
=-4843.6084711\RMSD=4.426e-09\RMSF=2.396e-05\Dipole=0.1993411,0.473285
4,-0.3981206\Quadrupole=1.6587884,-0.9877358,-0.6710526,1.4951281,0.40
12563,-0.1542546\PG=C01 [X(C1H4Se2)]\\@

1\1\GINC-ORC59\FOpt\RM062X\6-311G(df,p)\C1H4Se2\MEISTERP\20-Jul-2017\0
\\# opt=readfc freq m062x/6-311g(df,p)\\Methyl Se Se optimization and
frequency\\0,1\C,1.5513940083,1.2652956803,-0.0438594734\H,1.122168895
4,1.5021152353,-1.0139800591\H,2.6396615212,1.2673610155,-0.1082864274
\H,1.2226184483,1.9770090032,0.7088753\Se,-1.2531355702,-0.2526201792,
0.6867667644\Se,1.0402038824,-0.5435495518,0.4892143021\H,-1.586838185
4,-0.5425962034,-0.7183264067\\Version=EM64L-G09RevE.01\State=1-A\HF=-
4843.6185033\RMSD=5.476e-09\RMSF=2.073e-05\Dipole=0.1926834,0.4784002,
-0.4362519\Quadrupole=1.3359973,-0.8142668,-0.5217305,1.6231921,0.5235
215,-0.140995\PG=C01 [X(C1H4Se2)]\\@

1\1\GINC-ORC56\FOpt\RM062X\6-311+G(2df,p)\C1H4Se2\MEISTERP\20-Jul-2017
\0\\# opt=readfc freq m062x/6-311+g(2df,p)\\Methyl Se Se optimization
and frequency\\0,1\C,1.5511169775,1.2654666195,-0.0438392716\H,1.12101
91096,1.5007227091,-1.0129095884\H,2.6386045878,1.2673676148,-0.108717
2497\H,1.2225526544,1.9756323751,0.7092031735\Se,-1.2525329105,-0.2516
624529,0.6860656633\Se,1.0400851017,-0.5424785046,0.4883277712\H,-1.58
47725204,-0.542033361,-0.7177264984\\Version=EM64L-G09RevE.01\State=1-
A\HF=-4843.6159115\RMSD=3.794e-09\RMSF=2.022e-05\Dipole=0.2042628,0.46
84879,-0.3961731\Quadrupole=1.4509743,-0.8934989,-0.5574754,1.5936195,
0.4153959,-0.1553935\PG=C01 [X(C1H4Se2)]\\@

1\1\GINC-ORC8\FOpt\RM062X\6-311++G(3df,3pd)\C1H4Se2\MEISTERP\20-Aug-20
17\0\\# opt=readfc freq m062x/6-311++g(3df,3pd)\\Methyl Se Se optimiza
tion and frequency\\0,1\C,1.6200131267,1.3201853554,-0.0000271223\H,1.
291618989,1.8383192694,-0.8950221648\H,2.7032123811,1.2374941278,0.000
5750327\H,1.2905852098,1.8379509294,0.89481522\Se,-1.3191187265,0.1650
584689,-0.0000865206\Se,0.9472765247,-0.5062012662,-0.0008512962\H,-1.
7975145048,-1.2197918847,0.0010008513\\Version=EM64L-G09RevE.01\State=
1-A\HF=-4843.6173077\RMSD=7.073e-09\RMSF=2.562e-05\Dipole=0.2587184,0.
2716817,0.0004179\Quadrupole=2.1114335,1.2912935,-3.4027271,2.8178151,
-0.0015126,-0.0010777\PG=C01 [X(C1H4Se2)]\\@

CH_3SeSeH M08HX

1\1\GINC-ORC366\FOpt\RM08HX\6-31G(d)\C1H4Se2\MEISTERP\17-Aug-2017\0\\#
opt=readfc freq m08hx/6-31g(d)\\Methyl Se Se optimization and frequen
cy\\0,1\C,1.546820076,1.2547698693,0.0450678713\H,1.2020494214,1.96786
93555,-0.7083649533\H,2.6410418184,1.2741095007,0.107614977\H,1.112162
9025,1.477101566,1.0232550124\Se,-1.2313627,-0.2180223908,-0.690628326
6\Se,1.0409502612,-0.5537967003,-0.499358669\H,-1.5755887795,-0.529016
2004,0.7228180881\\Version=EM64L-G16RevA.03\State=1-A\HF=-4839.3946967
\RMSD=5.616e-09\RMSF=2.152e-05\Dipole=0.2041782,0.480156,0.4396574\Qua

```
drupole=1.3545197,-0.8244597,-0.53006,1.5599982,-0.5113395,0.1382242\P
G=C01 [X(C1H4Se2)]\\@

1\1\GINC-ORC389\FOpt\RM08HX\6-311G(d)\C1H4Se2\MEISTERP\17-Aug-2017\0\\
 # opt=readfc freq m08hx/6-311g(d)\\Methyl Se Se optimization and frequ
 ency\\0,1\C,1.5540991081,1.2623241954,0.0445848933\H,1.2165477636,1.97
 61698622,-0.7083017562\H,2.6468723713,1.2733224135,0.1024150673\H,1.12
 92228746,1.5017647681,1.0207054083\Se,-1.2553332728,-0.234178187,-0.68
 93743955\Se,1.0404372364,-0.5534901762,-0.4873494796\H,-1.5957730813,-
 0.552897876,0.7177242623\\Version=EM64L-G16RevA.03\State=1-A\HF=-4843.
 6232538\RMSD=2.811e-09\RMSF=1.195e-05\Dipole=0.1964472,0.4767451,0.435
 0303\Quadrupole=1.5257152,-0.9132066,-0.6125086,1.5689707,-0.5037127,0
 .0880772\PG=C01 [X(C1H4Se2)]\\@

1\1\GINC-ORC390\FOpt\RM08HX\6-311G(d,p)\C1H4Se2\MEISTERP\17-Aug-2017\0
 \\# opt=readfc freq m08hx/6-311g(d,p)\\Methyl Se Se optimization and f
 requency\\0,1\C,1.5548872116,1.2638078464,0.0453765423\H,1.2141943137,
 1.9749435088,-0.7084490253\H,2.6477983505,1.2694792219,0.0986422311\H,
 1.1316207172,1.499941954,1.022802374\Se,-1.2576794984,-0.2326487308,-0
 .6856740052\Se,1.0395792493,-0.5512486893,-0.485430577\H,-1.5943273439
 ,-0.551260111,0.7131364601\\Version=EM64L-G16RevA.03\State=1-A\HF=-484
 3.6297066\RMSD=9.372e-09\RMSF=9.259e-06\Dipole=0.1958301,0.4750643,0.4
 354788\Quadrupole=1.5388234,-0.8795458,-0.6592776,1.5846852,-0.4960367
 ,0.1017146\PG=C01 [X(C1H4Se2)]\\@

1\1\GINC-ORC187\FOpt\RM08HX\6-311+G(d,p)\C1H4Se2\MEISTERP\17-Aug-2017\
 0\\# opt=readfc freq m08hx/6-311+g(d,p)\\Methyl Se Se optimization and
  frequency\\0,1\C,1.5551442026,1.2638894752,0.0459120384\H,1.211188074
 1,1.9747551511,-0.7069646083\H,2.6484582154,1.2704449697,0.0946973292\
 H,1.1360543537,1.5010844054,1.025062881\Se,-1.2569568474,-0.2342099259
 ,-0.6875339705\Se,1.0397822842,-0.5518353916,-0.4816834446\H,-1.597597
 2827,-0.551113684,0.7109137747\\Version=EM64L-G16RevA.03\State=1-A\HF=
 -4843.63056\RMSD=2.138e-09\RMSF=1.268e-05\Dipole=0.1958614,0.474113,0.
 4333898\Quadrupole=1.5344342,-0.9072521,-0.6271821,1.6444754,-0.507342
 6,0.0905478\PG=C01 [X(C1H4Se2)]\\@

1\1\GINC-ORC183\FOpt\RM08HX\6-311G(2d,p)\C1H4Se2\MEISTERP\17-Aug-2017\
 0\\# opt=readfc freq m08hx/6-311g(2d,p)\\Methyl Se Se optimization and
  frequency\\0,1\C,1.5542446705,1.2633730459,0.0445094893\H,1.218311505
 5,1.9738680636,-0.7103665736\H,2.645763117,1.2676638676,0.1041391264\H
 ,1.1249435544,1.4970063521,1.0185711157\Se,-1.2602874861,-0.2297543346
 ,-0.6834391504\Se,1.0405983143,-0.5475831501,-0.4888803191\H,-1.587500
 6757,-0.5515588445,0.7158703117\\Version=EM64L-G16RevA.03\State=1-A\HF
 =-4843.6272171\RMSD=9.447e-09\RMSF=9.521e-06\Dipole=0.2049025,0.465599
 4,0.3911265\Quadrupole=1.6728957,-0.9734461,-0.6994497,1.478959,-0.374
 4519,0.1079613\PG=C01 [X(C1H4Se2)]\\@

1\1\GINC-ORC177\FOpt\RM08HX\6-311G(df,p)\C1H4Se2\MEISTERP\17-Aug-2017\
 0\\# opt=readfc freq m08hx/6-311g(df,p)\\Methyl Se Se optimization and
  frequency\\0,1\C,1.5534183946,1.2621618944,0.0446849969\H,1.214900727
 6,1.9762508083,-0.7076574778\H,2.6462468061,1.2664062427,0.0987602569\
 H,1.1306550809,1.5004448342,1.0220686842\Se,-1.2518108905,-0.235094945
 2,-0.6853874472\Se,1.0364677214,-0.5462434725,-0.4862357319\H,-1.59380
 484,-0.5509103619,0.7141707189\\Version=EM64L-G16RevA.03\State=1-A\HF=
 -4843.6370679\RMSD=4.208e-09\RMSF=1.124e-05\Dipole=0.1978144,0.470465,
 0.4309423\Quadrupole=1.3518362,-0.7975002,-0.554336,1.6156601,-0.50505
 03,0.1005717\PG=C01 [X(C1H4Se2)]\\@
```

```
1\1\GINC-ORC164\FOpt\RM08HX\6-311+G(2df,p)\C1H4Se2\MEISTERP\17-Aug-201
 7\0\\# opt=readfc freq m08hx/6-311+g(2df,p)\\Methyl Se Se optimization
  and frequency\\0,1\C,1.5528366984,1.2620228612,0.0441084261\H,1.21752
 28797,1.9755843043,-0.7089195828\H,2.6447438559,1.265555854,0.10204636
 18\H,1.1266577321,1.4989261297,1.0193357851\Se,-1.2516224338,-0.233340
 3053,-0.683585863\Se,1.0357053414,-0.5445776097,-0.4879378816\H,-1.589
 7710737,-0.5511562343,0.7153567543\\Version=EM64L-G16RevA.03\State=1-A
 \HF=-4843.6353753\RMSD=5.639e-09\RMSF=1.047e-05\Dipole=0.2063092,0.453
 4789,0.3855618\Quadrupole=1.4716986,-0.8939079,-0.5777907,1.5676256,-0
 .3946859,0.1024651\PG=C01 [X(C1H4Se2)]\\@
```

```
1\1\GINC-ORC162\FOpt\RM08HX\6-311++G(3df,3pd)\C1H4Se2\MEISTERP\17-Aug-
 2017\0\\# opt=readfc freq m08hx/6-311++g(3df,3pd)\\Methyl Se Se optimi
 zation and frequency\\0,1\C,1.5530734178,1.2620420784,0.0446102488\H,1
 .2160767294,1.9727382062,-0.7066621622\H,2.6423994611,1.2659556899,0.1
 020104566\H,1.1261659361,1.4941023733,1.0177837573\Se,-1.2520201205,-0
 .2291798984,-0.6822656394\Se,1.0366701934,-0.5443765864,-0.4903445287\
 H,-1.5862926174,-0.548266863,0.7152718676\\Version=EM64L-G16RevA.03\St
 ate=1-A\HF=-4843.6449548\RMSD=3.163e-09\RMSF=1.160e-05\Dipole=0.208258
 2,0.4473601,0.3819135\Quadrupole=1.4560332,-0.8713213,-0.5847119,1.553
 6306,-0.3786914,0.1124608\PG=C01 [X(C1H4Se2)]\\@
```

CH_3SeSeH MP2

```
1\1\GINC-ORC187\FOpt\RMP2-FC\6-31G(d)\C1H4Se2\MEISTERP\20-Jul-2017\0\\
 # opt=readfc freq mp2/6-31g(d)\\Methyl Se Se optimization and frequenc
 y\\0,1\C,1.5512566874,1.2607671316,0.0472146657\H,1.197560065,1.969234
 2993,-0.699869018\H,2.6421135688,1.2822840971,0.0996585104\H,1.1258767
 917,1.4777453903,1.0257321213\Se,-1.2445828852,-0.2400552437,-0.698796
 803\Se,1.0526876677,-0.5568911794,-0.4962378254\H,-1.5888388954,-0.520
 0694952,0.722702349\\Version=EM64L-G09RevE.01\State=1-A\HF=-4835.36345
 62\MP2=-4835.7162372\RMSD=7.440e-09\RMSF=8.376e-06\Dipole=0.1845892,0.
 4907802,0.4629396\PG=C01 [X(C1H4Se2)]\\@
```

```
1\1\GINC-ORC303\FOpt\RMP2-FC\6-311G(d)\C1H4Se2\MEISTERP\17-Aug-2017\0\
 \# opt freq=noraman mp2/6-311g(d)\\Methyl Se Se optimization and frequ
 ency\\0,1\C,1.5626475082,1.2596617945,0.0386477112\H,1.2038353577,2.00
 50191926,-0.6682720465\H,2.6545689347,1.2619961276,0.0573124105\H,1.17
 65197238,1.4594257023,1.0364650847\Se,-1.2976128148,-0.2137762797,-0.6
 622740086\Se,1.0255585447,-0.5317293534,-0.549978661\H,-1.5894442543,-
 0.567582184,0.7485035097\\Version=EM64L-G09RevE.01\State=1-A\HF=-4839.
 8067346\MP2=-4840.1861893\RMSD=3.568e-09\RMSF=9.000e-06\Dipole=0.19478
 61,0.4587812,0.468031\PG=C01 [X(C1H4Se2)]\\@
```

```
1\1\GINC-ORC165\FOpt\RMP2-FC\6-311G(d,p)\C1H4Se2\MEISTERP\20-Jul-2017\
 0\\# opt=readfc freq mp2/6-311g(d,p)\\Methyl Se Se optimization and fr
 equency\\0,1\C,1.5550116235,1.2651938462,0.0441174133\H,1.2192090114,1
 .9771589495,-0.7087491476\H,2.6466559588,1.2716053453,0.1022503849\H,1
 .1306098056,1.5051835691,1.0184013635\Se,-1.2694829979,-0.2513468894,-
 0.684016656\Se,1.0506171728,-0.5500638325,-0.4841407076\H,-1.596547574
 3,-0.5447159881,0.7125413495\\Version=EM64L-G09RevE.01\State=1-A\HF=-4
 839.816234\MP2=-4840.2211526\RMSD=8.642e-09\RMSF=1.131e-05\Dipole=0.17
 14613,0.4509908,0.4196402\PG=C01 [X(C1H4Se2)]\\@
```

```
1\1\GINC-ORC163\FOpt\RMP2-FC\6-311+G(d,p)\C1H4Se2\MEISTERP\20-Jul-2017
 \0\\# opt=readfc freq mp2/6-311+g(d,p)\\Methyl Se Se optimization and
```

frequency\\0,1\C,1.554599748,1.2648932932,0.0454781818\H,1.2116369274,
1.9748803508,-0.7063252517\H,2.6467620488,1.274827032,0.095798016\H,1.
1367665231,1.5059993696,1.0225220671\Se,-1.2669941095,-0.2550583502,-0
.6884059505\Se,1.0527027594,-0.5525306794,-0.4774357173\H,-1.599400897
3,-0.5399960159,0.7087726547\\Version=EM64L-G09RevE.01\State=1-A\HF=-4
839.8168963\MP2=-4840.2229909\RMSD=9.541e-09\RMSF=9.627e-06\Dipole=0.1
716045,0.4612438,0.4229574\PG=C01 [X(C1H4Se2)]\\@

1\1\GINC-ORC159\FOpt\RMP2-FC\6-311G(2d,p)\C1H4Se2\MEISTERP\20-Jul-2017
\0\\# opt=readfc freq mp2/6-311g(2d,p)\\Methyl Se Se optimization and
frequency\\0,1\C,1.5541313215,1.2636271797,0.0437602652\H,1.2203619623
,1.9747291947,-0.7079189877\H,2.6437259428,1.2733141598,0.1038561213\H
,1.1296429292,1.5034574325,1.015651277\Se,-1.2708350044,-0.2489883899,
-0.6843871019\Se,1.0517855029,-0.5480493095,-0.4830816626\H,-1.5927396
543,-0.5450752673,0.7125240887\\Version=EM64L-G09RevE.01\State=1-A\HF=
-4839.812627\MP2=-4840.2327948\RMSD=6.120e-09\RMSF=9.248e-06\Dipole=0.
1809195,0.4475799,0.3774787\PG=C01 [X(C1H4Se2)]\\@

1\1\GINC-ORC158\FOpt\RMP2-FC\6-311G(df,p)\C1H4Se2\MEISTERP\20-Jul-2017
\0\\# opt=readfc freq mp2/6-311g(df,p)\\Methyl Se Se optimization and
frequency\\0,1\C,1.5498672745,1.2616822196,0.0441586715\H,1.2138520434
,1.9761397207,-0.706601682\H,2.6416790399,1.2651428466,0.1005668138\H,
1.1269348773,1.499871316,1.019806683\Se,-1.2510535363,-0.2499807771,-0
.6868732479\Se,1.0427220971,-0.5419391961,-0.4854525313\H,-1.587928796
,-0.5379011297,0.714799293\\Version=EM64L-G09RevE.01\State=1-A\HF=-483
9.8243101\MP2=-4840.2879054\RMSD=5.619e-09\RMSF=4.752e-06\Dipole=0.171
2424,0.4590685,0.4271984\PG=C01 [X(C1H4Se2)]\\@

1\1\GINC-ORC157\FOpt\RMP2-FC\6-311+G(2df,p)\C1H4Se2\MEISTERP\20-Jul-20
17\0\\# opt=readfc freq mp2/6-311+g(2df,p)\\Methyl Se Se optimization
and frequency\\0,1\C,1.5493415859,1.2608889586,0.043943295\H,1.2146077
14,1.9745375669,-0.7062070764\H,2.6396766727,1.2691779264,0.1015885169
\H,1.1272850162,1.5008864003,1.0178446215\Se,-1.2491932439,-0.24948175
22,-0.6866268138\Se,1.0422720122,-0.5447787491,-0.4834553569\H,-1.5879
167572,-0.538215351,0.7133168138\\Version=EM64L-G09RevE.01\State=1-A\H
F=-4839.8215727\MP2=-4840.2977\RMSD=9.513e-09\RMSF=3.044e-06\Dipole=0.
1852381,0.4563867,0.390358\PG=C01 [X(C1H4Se2)]\\@

1\1\GINC-ORC280\FOpt\RMP2-FC\6-311++G(3df,3pd)\C1H4Se2\MEISTERP\22-Aug
-2017\0\\# opt freq=noraman mp2/6-311++g(3df,3pd) guess=read\\Methyl S
e Se optimization and frequency\\0,1\C,-1.716422,1.272513,-0.005592\H,
-1.465528,1.801857,0.907666\H,-2.792389,1.115316,-0.05969\H,-1.378945,
1.824924,-0.876218\Se,1.330695,0.125536,-0.045038\Se,-0.906107,-0.4970
82,0.005543\H,1.499409,0.255385,1.404642\\Version=EM64L-G09RevE.01\Sta
te=1-A\HF=-4839.8374223\MP2=-4840.3325823\RMSD=8.219e-09\RMSF=1.181e-0
5\Dipole=-0.2920056,0.4777782,0.2165053\PG=C01 [X(C1H4Se2)]\\@

CH_3SeSeH QCISD

1\1\GINC-ORC153\FOpt\RQCISD-FC\6-311+G(2df,p)\C1H4Se2\MEISTERP\22-Aug-
2017\0\\# opt freq=noraman qcisd/6-311+g(2df,p) guess=read\\Methyl Se
Se optimization and frequency\\0,1\C,-1.742682,1.268584,-0.00432\H,-1.
494117,1.807859,0.908912\H,-2.821277,1.099316,-0.051289\H,-1.419106,1.
828614,-0.880633\Se,1.338714,0.129125,-0.045446\Se,-0.907362,-0.49951,
0.005363\H,1.524637,0.245802,1.411744\\Version=EM64L-G09RevE.01\State=
1-A\HF=-4839.8214565\MP2=-4840.297615\MP3=-4840.3391604\MP4D=-4840.352

2105\MP4DQ=-4840.3384103\MP4SDQ=-4840.3417224\QCISD=-4840.3417019\RMSD
=5.302e-09\RMSF=5.822e-06\Dipole=-0.3063472,0.4948923,0.2299809\PG=C01
 [X(C1H4Se2)]\\@

CH_3SeSe^- B3LYP

1\1\GINC-ORC362\FOpt\RB3LYP\6-31G(d)\C1H3Se2(1-)\MEISTERP\25-Jun-2017\
0\\# opt freq b3lyp/6-31g(d)\\Methyl Se Se optimization and frequency\
\-1,1\C,-0.5623657311,2.0368957138,-0.0944900175\H,-0.7469456225,0.982
7228976,-0.3165685132\H,-0.2326204736,2.5604774073,-1.0015608975\H,-1.
4782439761,2.4933477513,0.2895509944\Se,-0.1615256664,0.9664596131,3.0
569694446\Se,0.8542656581,2.1674476771,1.2893380091\\Version=EM64L-G16
RevA.03\State=1-A'\HF=-4838.7253233\RMSD=5.832e-09\RMSF=5.049e-05\Dipo
le=0.0304735,0.4770028,-1.1520615\Quadrupole=1.1895479,-0.0581711,-1.1
313767,-0.4140413,1.5638065,1.5114006\PG=CS [SG(C1H1Se2),X(H2)]\\@

1\1\GINC-ORC361\FOpt\RB3LYP\6-311G(d)\C1H3Se2(1-)\MEISTERP\25-Jun-2017
\0\\# opt freq b3lyp/6-311g(d)\\Methyl Se Se optimization and frequenc
y\\-1,1\C,-0.5653370247,2.0370656185,-0.0984984091\H,-0.7527058013,0.9
869711184,-0.3260442866\H,-0.2283440485,2.5617061273,-0.9994636088\H,-
1.4817596514,2.492959557,0.2782149169\Se,-0.1539880959,0.959237559,3.0
840631564\Se,0.8546988111,2.1694110794,1.2849672536\\Version=EM64L-G16
RevA.03\State=1-A'\HF=-4843.0927045\RMSD=8.254e-09\RMSF=5.215e-05\Dipo
le=0.0515718,0.5083573,-1.2047504\Quadrupole=1.1448649,-0.0854537,-1.0
594112,-0.3250247,1.4403802,1.3721126\PG=CS [SG(C1H1Se2),X(H2)]\\@

1\1\GINC-ORC392\FOpt\RB3LYP\6-311G(d,p)\C1H3Se2(1-)\MEISTERP\25-Jun-20
17\0\\# opt freq b3lyp/6-311g(d,p)\\Methyl Se Se optimization and freq
uency\\-1,1\C,-0.5670392224,2.0367766853,-0.0998320482\H,-0.7518986304
,0.9858774261,-0.3253831523\H,-0.2260799471,2.5623704693,-0.9983876458
\H,-1.4817984436,2.4936133496,0.2795772079\Se,-0.1535176243,0.95949178
26,3.0839971936\Se,0.852898056,2.1692213474,1.2832674644\\Version=EM64
L-G16RevA.03\State=1-A'\HF=-4843.0974018\RMSD=4.351e-09\RMSF=5.650e-05
\Dipole=0.0543062,0.5080182,-1.2006063\Quadrupole=1.1513858,-0.0707021
,-1.0806837,-0.3222191,1.4221597,1.3781778\PG=CS [SG(C1H1Se2),X(H2)]\\
@

1\1\GINC-ORC391\FOpt\RB3LYP\6-311+G(d,p)\C1H3Se2(1-)\MEISTERP\25-Jun-2
017\0\\# opt freq b3lyp/6-311+g(d,p)\\Methyl Se Se optimization and fr
equency\\-1,1\C,-0.5689626918,2.0353958034,-0.0987112058\H,-0.75150603
86,0.9845344431,-0.3248070194\H,-0.2234757892,2.5620829337,-0.99452904
23\H,-1.4823091971,2.4941363846,0.2809020574\Se,-0.1542175038,0.961367
6918,3.0784774638\Se,0.8530354094,2.1698338035,1.2819067672\\Version=E
M64L-G16RevA.03\State=1-A'\HF=-4843.1052148\RMSD=6.084e-09\RMSF=5.554e
-05\Dipole=0.0668536,0.5740513,-1.3500411\Quadrupole=1.1715145,-0.1692
085,-1.0023059,-0.3730076,1.637359,1.4295136\PG=CS [SG(C1H1Se2),X(H2)]
\\@

1\1\GINC-ORC390\FOpt\RB3LYP\6-311G(2d,p)\C1H3Se2(1-)\MEISTERP\25-Jun-2
017\0\\# opt freq b3lyp/6-311g(2d,p)\\Methyl Se Se optimization and fr
equency\\-1,1\C,-0.5672179211,2.0363849292,-0.0990712834\H,-0.75090178
78,0.9864239259,-0.3240693524\H,-0.226274208,2.5614396553,-0.996302169
3\H,-1.4803914675,2.4933126468,0.2805510782\Se,-0.155417987,0.95905341
88,3.0827968874\Se,0.8527675556,2.1707364848,1.2793338525\\Version=EM6
4L-G16RevA.03\State=1-A'\HF=-4843.0927245\RMSD=1.995e-09\RMSF=6.628e-0
5\Dipole=0.0376133,0.4840703,-1.1610615\Quadrupole=1.1673336,-0.048229
7,-1.1191039,-0.3186507,1.3999772,1.393673\PG=CS [SG(C1H1Se2),X(H2)]\\

@

1\1\GINC-ORC389\FOpt\RB3LYP\6-311G(df,p)\C1H3Se2(1-)\MEISTERP\25-Jun-2
017\0\\# opt freq b3lyp/6-311g(df,p)\\Methyl Se Se optimization and fr
equency\\-1,1\C,-0.5655969049,2.036265626,-0.0968181488\H,-0.752201212
6,0.9859036763,-0.324081309\H,-0.2241669203,2.5619261734,-0.9949722175
\H,-1.4816187226,2.492643318,0.2804793054\Se,-0.1521150655,0.964229807
1,3.0738808905\Se,0.8482630148,2.1663824596,1.2847504994\\Version=EM64
L-G16RevA.03\State=1-A'\HF=-4843.1033404\RMSD=4.759e-09\RMSF=5.529e-05
\Dipole=0.0552937,0.5059114,-1.1941642\Quadrupole=1.1490302,0.0080127,
-1.157043,-0.4241166,1.4607006,1.498167\PG=CS [SG(C1H1Se2),X(H2)]\\@

1\1\GINC-ORC362\FOpt\RB3LYP\6-311+G(2df,p)\C1H3Se2(1-)\MEISTERP\25-Jun
-2017\0\\# opt freq b3lyp/6-311+g(2df,p)\\Methyl Se Se optimization an
d frequency\\-1,1\C,-0.5676443954,2.0345557193,-0.0950269159\H,-0.7513
337328,0.9852384434,-0.3230018813\H,-0.2215514741,2.5608934591,-0.9892
427939\H,-1.4812037063,2.4929127278,0.2819337469\Se,-0.153210355,0.966
5205976,3.0668500849\Se,0.8475078496,2.167230114,1.281726774\\Version=
EM64L-G16RevA.03\State=1-A'\HF=-4843.1065311\RMSD=3.825e-09\RMSF=5.390
e-05\Dipole=0.0595543,0.5467586,-1.2908267\Quadrupole=1.1687331,-0.085
3261,-1.0834069,-0.4599427,1.6478248,1.5345786\PG=CS [SG(C1H1Se2),X(H2
)]\\@

1\1\GINC-ORC392\FOpt\RB3LYP\6-311++G(3df,3pd)\C1H3Se2(1-)\MEISTERP\25-
Jun-2017\0\\# opt freq b3lyp/6-311++g(3df,3pd)\\Methyl Se Se optimizat
ion and frequency\\-1,1\C,-0.5681439955,2.0345056725,-0.095504965\H,-0
.7508692685,0.9863911409,-0.322157563\H,-0.2218859437,2.5601608992,-0.
9878205891\H,-1.4798603565,2.4922499334,0.2820496216\Se,-0.1535756781,
0.9666723643,3.0660310669\Se,0.8468994253,2.1673710528,1.2806414347\\V
ersion=EM64L-G16RevA.03\State=1-A'\HF=-4843.11579\RMSD=3.184e-09\RMSF=
5.328e-05\Dipole=0.0813317,0.5416378,-1.2517892\Quadrupole=1.1646453,0
.0257946,-1.1904399,-0.5169831,1.5797772,1.6128484\PG=CS [SG(C1H1Se2),
X(H2)]\\@

CH_3SeSe^- B3PW91

1\1\GINC-ORC229\FOpt\RB3PW91\6-31G(d)\C1H3Se2(1-)\MEISTERP\25-Jun-2017
\0\\# opt freq b3pw91/6-31g(d)\\Methyl Se Se optimization and frequenc
y\\-1,1\C,-0.5594276611,2.0355494653,-0.0875899225\H,-0.7442868506,0.9
811451672,-0.3101319348\H,-0.2293418739,2.5592686559,-0.9945926273\H,-
1.4757810545,2.492174584,0.2961498988\Se,-0.1633554074,0.9749672242,3.
0335583924\Se,0.8447570344,2.1642459634,1.2858452119\\Version=EM64L-G1
6RevA.03\State=1-A'\HF=-4838.6735409\RMSD=5.046e-09\RMSF=6.053e-05\Dip
ole=0.0134608,0.4807172,-1.1818453\Quadrupole=1.1375124,-0.1079933,-1.
0295191,-0.424459,1.6037937,1.4643473\PG=CS [SG(C1H1Se2),X(H2)]\\@

1\1\GINC-ORC229\FOpt\RB3PW91\6-31G(d)\C1H3Se2(1-)\MEISTERP\25-Jun-2017
\0\\# opt freq b3pw91/6-31g(d)\\Methyl Se Se optimization and frequenc
y\\-1,1\C,-0.5594276611,2.0355494653,-0.0875899225\H,-0.7442868506,0.9
811451672,-0.3101319348\H,-0.2293418739,2.5592686559,-0.9945926273\H,-
1.4757810545,2.492174584,0.2961498988\Se,-0.1633554074,0.9749672242,3.
0335583924\Se,0.8447570344,2.1642459634,1.2858452119\\Version=EM64L-G1
6RevA.03\State=1-A'\HF=-4838.6735409\RMSD=5.046e-09\RMSF=6.053e-05\Dip
ole=0.0134608,0.4807172,-1.1818453\Quadrupole=1.1375124,-0.1079933,-1.
0295191,-0.424459,1.6037937,1.4643473\PG=CS [SG(C1H1Se2),X(H2)]\\@

1\1\GINC-ORC157\FOpt\RB3PW91\6-311G(d,p)\C1H3Se2(1-)\MEISTERP\25-Jun-2
017\0\\# opt freq b3pw91/6-311g(d,p)\\Methyl Se Se optimization and fr
equency\\-1,1\C,-0.5633490221,2.0353575029,-0.0918427167\H,-0.74926899
96,0.983924003,-0.3183921022\H,-0.2234893188,2.5609181778,-0.991642458
2\H,-1.4794611894,2.4922638817,0.2868105875\Se,-0.1563126156,0.9686354
164,3.0578363906\Se,0.8444453304,2.1662520802,1.2804693107\\Version=EM
64L-G16RevA.03\State=1-A'\HF=-4843.0422675\RMSD=4.153e-09\RMSF=6.357e-
05\Dipole=0.0387412,0.5141916,-1.2347717\Quadrupole=1.1091828,-0.12883
4,-0.9803488,-0.3353348,1.4842731,1.3412699\PG=CS [SG(C1H1Se2),X(H2)]\
\@

1\1\GINC-ORC154\FOpt\RB3PW91\6-311+G(d,p)\C1H3Se2(1-)\MEISTERP\25-Jun-
2017\0\\# opt freq b3pw91/6-311+g(d,p)\\Methyl Se Se optimization and
frequency\\-1,1\C,-0.5648829783,2.0341455315,-0.0906728907\H,-0.749194
2032,0.9828899663,-0.318336207\H,-0.2211103955,2.5606879828,-0.9881985
151\H,-1.4801134554,2.4927317196,0.2874690913\Se,-0.1564701678,0.97028
64842,3.0535313622\Se,0.8443353885,2.166609376,1.2794461788\\Version=E
M64L-G16RevA.03\State=1-A'\HF=-4843.0483525\RMSD=6.651e-09\RMSF=5.977e
-05\Dipole=0.051749,0.5705522,-1.3595444\Quadrupole=1.1283109,-0.21012
64,-0.9181845,-0.3846139,1.674126,1.3936176\PG=CS [SG(C1H1Se2),X(H2)]\
\@

1\1\GINC-ORC144\FOpt\RB3PW91\6-311G(2d,p)\C1H3Se2(1-)\MEISTERP\25-Jun-
2017\0\\# opt freq b3pw91/6-311g(2d,p)\\Methyl Se Se optimization and
frequency\\-1,1\C,-0.5635673093,2.0349699997,-0.091140316\H,-0.7481425
475,0.9842336985,-0.3169570341\H,-0.2236636228,2.5601019509,-0.9898184
87\H,-1.4780986928,2.4920859857,0.2880500156\Se,-0.1582881636,0.968206
2769,3.0565223822\Se,0.8443245238,2.1677531502,1.2765824545\\Version=E
M64L-G16RevA.03\State=1-A'\HF=-4843.037304\RMSD=2.120e-09\RMSF=7.225e-
05\Dipole=0.0207096,0.4865764,-1.1877022\Quadrupole=1.1142524,-0.11383
51,-1.0004172,-0.3169965,1.4428165,1.3298744\PG=CS [SG(C1H1Se2),X(H2)]
\\@

1\1\GINC-ORC143\FOpt\RB3PW91\6-311G(df,p)\C1H3Se2(1-)\MEISTERP\25-Jun-
2017\0\\# opt freq b3pw91/6-311g(df,p)\\Methyl Se Se optimization and
frequency\\-1,1\C,-0.5618939357,2.0348986153,-0.0889434384\H,-0.749561
0422,0.9839430032,-0.3170214171\H,-0.2216532976,2.5605072181,-0.988403
0223\H,-1.4792603333,2.4912647134,0.2877727452\Se,-0.1550071469,0.9732
368447,3.047943383\Se,0.8399399424,2.1635006656,1.281890767\\Version=E
M64L-G16RevA.03\State=1-A'\HF=-4843.0484954\RMSD=4.388e-09\RMSF=6.485e
-05\Dipole=0.0394523,0.5115528,-1.227337\Quadrupole=1.1057917,-0.04899
07,-1.056801,-0.4397441,1.5240786,1.4631544\PG=CS [SG(C1H1Se2),X(H2)]\
\@

1\1\GINC-ORC142\FOpt\RB3PW91\6-311+G(2df,p)\C1H3Se2(1-)\MEISTERP\25-Ju
n-2017\0\\# opt freq b3pw91/6-311+g(2df,p)\\Methyl Se Se optimization
and frequency\\-1,1\C,-0.5636419023,2.0333387698,-0.0871648182\H,-0.74
88000004,0.9832800571,-0.3162138625\H,-0.2193137076,2.5596081433,-0.98
33394946\H,-1.4789901073,2.4916156332,0.2889871008\Se,-0.1559017954,0.
9752634853,3.0418129935\Se,0.8392116991,2.1642449725,1.2791570956\\Ver
sion=EM64L-G16RevA.03\State=1-A'\HF=-4843.0496969\RMSD=4.569e-09\RMSF=
6.139e-05\Dipole=0.0422492,0.5394807,-1.2935671\Quadrupole=1.1182052,-
0.1333237,-0.9848815,-0.4621406,1.6750396,1.4807188\PG=CS [SG(C1H1Se2)
,X(H2)]\\@

1\1\GINC-ORC141\FOpt\RB3PW91\6-311++G(3df,3pd)\C1H3Se2(1-)\MEISTERP\25
-Jun-2017\0\\# opt freq b3pw91/6-311++g(3df,3pd)\\Methyl Se Se optimiz

ation and frequency\\-1,1\C,-0.5640166959,2.0333525077,-0.087651255\H,
-0.748387463,0.9842969201,-0.3154285065\H,-0.2196462333,2.5590101635,-
0.9822503567\H,-1.4777919105,2.491009579,0.2891212815\Se,-0.1561818199
,0.9753973124,3.0411416013\Se,0.8385883069,2.1642845794,1.2783062455\\
Version=EM64L-G16RevA.03\State=1-A'\HF=-4843.0589946\RMSD=4.131e-09\RM
SF=5.979e-05\Dipole=0.0652328,0.5361311,-1.2574886\Quadrupole=1.114792
5,-0.0231366,-1.0916558,-0.5121686,1.5988798,1.5521142\PG=CS [SG(C1H1S
e2),X(H2)]\\@

CH_3SeSe^- wB97XD

1\1\GINC-ORC362\FOpt\RwB97XD\6-31G(d)\C1H3Se2(1-)\MEISTERP\25-Jun-2017
\0\\# opt freq wb97xd/6-31g(d)\\Methyl Se Se optimization and frequenc
y\\-1,1\C,-0.5562624102,2.0351155668,-0.0826895676\H,-0.7415872248,0.9
811005206,-0.3033267716\H,-0.2320196989,2.5567825189,-0.991627319\H,-1
.4721246049,2.4901534502,0.302162021\Se,-0.1704634381,0.9779663493,3.0
175076896\Se,0.8450215601,2.1662326562,1.2812129565\\Version=EM64L-G16
RevA.03\State=1-A'\HF=-4838.7450633\RMSD=2.336e-09\RMSF=5.497e-05\Dipo
le=0.0110279,0.4883675,-1.2038475\Quadrupole=1.0958236,-0.134977,-0.96
08466,-0.4626178,1.6608096,1.4603338\PG=CS [SG(C1H1Se2),X(H2)]\\@

1\1\GINC-ORC392\FOpt\RwB97XD\6-311G(d)\C1H3Se2(1-)\MEISTERP\25-Jun-201
7\0\\# opt freq wb97xd/6-311g(d)\\Methyl Se Se optimization and freque
ncy\\-1,1\C,-0.5577502935,2.0354422035,-0.0852988091\H,-0.7466299532,0
.9846968637,-0.3106819641\H,-0.2301438973,2.5574597722,-0.9910520281\H
,-1.4750257609,2.4893260005,0.2930318364\Se,-0.166201508,0.9710320588,
3.0399320694\Se,0.8483155971,2.1693941636,1.2773079051\\Version=EM64L-
G16RevA.03\State=1-A'\HF=-4843.1217476\RMSD=4.288e-09\RMSF=5.294e-05\D
ipole=0.0339278,0.5230945,-1.2627678\Quadrupole=1.0562042,-0.1787796,-
0.8774246,-0.3759775,1.5661378,1.324808\PG=CS [SG(C1H1Se2),X(H2)]\\@

1\1\GINC-ORC391\FOpt\RwB97XD\6-311G(d,p)\C1H3Se2(1-)\MEISTERP\25-Jun-2
017\0\\# opt freq wb97xd/6-311g(d,p)\\Methyl Se Se optimization and fr
equency\\-1,1\C,-0.5591103213,2.035240933,-0.0864380925\H,-0.745950166
4,0.9837801085,-0.3100686508\H,-0.2282372082,2.5580437645,-0.990207037
6\H,-1.475039688,2.4898422328,0.2942201182\Se,-0.1657712209,0.97126218
42,3.0398776826\Se,0.8466727891,2.1691818389,1.2758549905\\Version=EM6
4L-G16RevA.03\State=1-A'\HF=-4843.1262956\RMSD=7.150e-09\RMSF=5.280e-0
5\Dipole=0.0376934,0.5225113,-1.256771\Quadrupole=1.0635895,-0.1622346
,-0.9013549,-0.3721227,1.5447626,1.3309387\PG=CS [SG(C1H1Se2),X(H2)]\\
@

1\1\GINC-ORC390\FOpt\RwB97XD\6-311+G(d,p)\C1H3Se2(1-)\MEISTERP\25-Jun-
2017\0\\# opt freq wb97xd/6-311+g(d,p)\\Methyl Se Se optimization and
frequency\\-1,1\C,-0.5606708667,2.0340656021,-0.0853916654\H,-0.746138
9483,0.9829227238,-0.3107533829\H,-0.2256167126,2.5579164616,-0.986728
0683\H,-1.4759506171,2.4904765697,0.2941339208\Se,-0.1650771459,0.9727
285644,3.0370604587\Se,0.8460184736,2.1692411401,1.2749177469\\Version
=EM64L-G16RevA.03\State=1-A'\HF=-4843.1326206\RMSD=8.522e-09\RMSF=4.66
4e-05\Dipole=0.050511,0.5877264,-1.4038412\Quadrupole=1.0587972,-0.281
9626,-0.7768346,-0.4142241,1.756467,1.3460314\PG=CS [SG(C1H1Se2),X(H2)
]\\@

1\1\GINC-ORC389\FOpt\RwB97XD\6-311G(2d,p)\C1H3Se2(1-)\MEISTERP\25-Jun-
2017\0\\# opt freq wb97xd/6-311g(2d,p)\\Methyl Se Se optimization and
frequency\\-1,1\C,-0.5592847089,2.0348704464,-0.0857251358\H,-0.745256
8274,0.9842831983,-0.3094602251\H,-0.2281592535,2.5573065116,-0.988275

5362\H,-1.4740607712,2.4897554116,0.2945918494\Se,-0.1666914419,0.9710
400968,3.0393209189\Se,0.8460171884,2.1700953962,1.2727871433\\Version
=EM64L-G16RevA.03\State=1-A'\HF=-4843.121145\RMSD=9.783e-09\RMSF=5.613
e-05\Dipole=0.0189941,0.4946631,-1.2099264\Quadrupole=1.0643592,-0.154
3831,-0.9099761,-0.3446436,1.496523,1.3046207\PG=CS [SG(C1H1Se2),X(H2)
]\\@

1\1\GINC-ORC362\FOpt\RwB97XD\6-311G(df,p)\C1H3Se2(1-)\MEISTERP\25-Jun-
2017\0\\# opt freq wb97xd/6-311g(df,p)\\Methyl Se Se optimization and
frequency\\-1,1\C,-0.5581604949,2.0347829777,-0.0841507463\H,-0.746654
1468,0.9838591108,-0.3097057943\H,-0.2258410358,2.5579270947,-0.987025
2208\H,-1.4753513522,2.4891108375,0.2942578127\Se,-0.1630988753,0.9759
277658,3.0314739578\Se,0.8416700926,2.1657432745,1.2783890076\\Version
=EM64L-G16RevA.03\State=1-A'\HF=-4843.1328298\RMSD=6.675e-09\RMSF=5.36
0e-05\Dipole=0.0377106,0.5188442,-1.2476107\Quadrupole=1.0617036,-0.07
92636,-0.98244,-0.4770805,1.5824035,1.4554125\PG=CS [SG(C1H1Se2),X(H2)
]\\@

1\1\GINC-ORC390\FOpt\RwB97XD\6-311+G(2df,p)\C1H3Se2(1-)\MEISTERP\25-Ju
n-2017\0\\# opt freq wb97xd/6-311+g(2df,p)\\Methyl Se Se optimization
and frequency\\-1,1\C,-0.5598214074,2.0332801931,-0.0824093056\H,-0.74
65801569,0.9836182533,-0.3103998319\H,-0.223057517,2.5571773965,-0.981
7983711\H,-1.4756625711,2.489665696,0.2938830463\Se,-0.1622887446,0.97
7776385,3.0278441079\Se,0.8399745828,2.1658331377,1.2761193676\\Versio
n=EM64L-G16RevA.03\State=1-A'\HF=-4843.13412\RMSD=4.793e-09\RMSF=4.761
e-05\Dipole=0.0390476,0.5552462,-1.336722\Quadrupole=1.0476466,-0.2045
685,-0.8430781,-0.4886019,1.7513918,1.4290139\PG=CS [SG(C1H1Se2),X(H2)
]\\@

1\1\GINC-ORC245\FOpt\RwB97XD\6-311++G(3df,3pd)\C1H3Se2(1-)\MEISTERP\25
-Jun-2017\0\\# opt freq wb97xd/6-311++g(3df,3pd)\\Methyl Se Se optimiz
ation and frequency\\-1,1\C,-0.5602927016,2.0333073329,-0.0830455739\H
,-0.7460163134,0.9848629994,-0.3093198928\H,-0.2236886195,2.5564249372
,-0.9806844664\H,-1.4741237542,2.4888964639,0.2941549016\Se,-0.1628838
399,0.9778444096,3.0269565734\Se,0.8395694181,2.1660149168,1.275177480
2\\Version=EM64L-G16RevA.03\State=1-A'\HF=-4843.1438472\RMSD=8.652e-09
\RMSF=4.528e-05\Dipole=0.0551131,0.5516882,-1.3084711\Quadrupole=1.044
5462,-0.1040416,-0.9405046,-0.5508943,1.7052955,1.511505\PG=CS [SG(C1H
1Se2),X(H2)]\\@

CH_3SeSe^- M062X

1\1\GINC-ORC251\FOpt\RB3LYP\6-31G(d)\C1H3Se2(1-)\MEISTERP\25-Jun-2017\
0\\# opt freq b3lyp/6-31g(d)\\Methyl Se Se optimization and frequency\
\-1,1\C,-0.5623657314,2.0368957129,-0.0944900155\H,-0.7469456235,0.982
7228979,-0.3165685111\H,-0.2326204723,2.5604774069,-1.0015608949\H,-1.
478243976,2.4933477493,0.2895509956\Se,-0.1615256657,0.9664596173,3.05
6969435\Se,0.8542656573,2.1674476756,1.2893380119\\Version=EM64L-G16Re
vA.03\State=1-A'\HF=-4838.7253233\RMSD=5.832e-09\RMSF=5.049e-05\Dipole
=0.0304735,0.4770027,-1.1520615\Quadrupole=1.1895479,-0.0581711,-1.131
3767,-0.4140413,1.5638065,1.5114006\PG=CS [SG(C1H1Se2),X(H2)]\\@

1\1\GINC-ORC245\FOpt\RM062X\6-311G(d)\C1H3Se2(1-)\MEISTERP\25-Jun-2017
\0\\# opt freq m062x/6-311g(d)\\Methyl Se Se optimization and frequenc
y\\-1,1\C,-0.5565683987,2.0354791518,-0.0839649091\H,-0.7400978904,0.9
829737934,-0.2968120348\H,-0.2372068961,2.5544412158,-0.9920505986\H,-
1.4680219456,2.4866284427,0.3065107647\Se,-0.18193891,0.9729749988,3.0

161021326\Se,0.8563982268,2.1748534587,1.2734536602\\Version=EM64L-G16
RevA.03\State=1-A'\HF=-4843.0692442\RMSD=7.019e-09\RMSF=6.394e-05\Dipo
le=0.0406368,0.5241821,-1.2573838\Quadrupole=1.0117099,-0.1848536,-0.8
268563,-0.4174415,1.5983681,1.3259161\PG=CS [SG(C1H1Se2),X(H2)]\\@

1\1\GINC-ORC240\FOpt\RM062X\6-311G(d,p)\C1H3Se2(1-)\MEISTERP\25-Jun-20
17\0\\# opt freq m062x/6-311g(d,p)\\Methyl Se Se optimization and freq
uency\\-1,1\C,-0.5580295294,2.0353095689,-0.0853051505\H,-0.7391446951
,0.981930167,-0.2957050831\H,-0.2354498629,2.5550729338,-0.9915051177\
H,-1.4678049039,2.4871054706,0.3082278601\Se,-0.1819584894,0.973146490
9,3.0156511016\Se,0.8549516669,2.1747864299,1.2718754046\\Version=EM64
L-G16RevA.03\State=1-A'\HF=-4843.072719\RMSD=6.114e-09\RMSF=6.431e-05\
Dipole=0.0424266,0.5241598,-1.2551688\Quadrupole=1.0146901,-0.1715401,
-0.8431499,-0.4169325,1.5825502,1.3308304\PG=CS [SG(C1H1Se2),X(H2)]\\@

1\1\GINC-ORC362\FOpt\RM062X\6-311+G(d,p)\C1H3Se2(1-)\MEISTERP\25-Jun-2
017\0\\# opt freq m062x/6-311+g(d,p)\\Methyl Se Se optimization and fr
equency\\-1,1\C,-0.5598930837,2.0341252153,-0.0846018242\H,-0.73989507
34,0.9812142724,-0.2975995615\H,-0.232185464,2.5552596413,-0.988031867
9\H,-1.4693274132,2.4879845479,0.3069733444\Se,-0.1797208989,0.9746390
285,3.0146309757\Se,0.8535861203,2.1741283554,1.27186795\\Version=EM64
L-G16RevA.03\State=1-A'\HF=-4843.0783933\RMSD=9.491e-09\RMSF=5.607e-05
\Dipole=0.0545205,0.5745318,-1.3661188\Quadrupole=1.0131176,-0.253499,
-0.7596186,-0.4815312,1.7790046,1.3799968\PG=CS [SG(C1H1Se2),X(H2)]\\@

1\1\GINC-ORC392\FOpt\RM062X\6-311G(2d,p)\C1H3Se2(1-)\MEISTERP\25-Jun-2
017\0\\# opt freq m062x/6-311g(2d,p)\\Methyl Se Se optimization and fr
equency\\-1,1\C,-0.5584802218,2.0349629634,-0.0849850811\H,-0.73814887
91,0.982406148,-0.294946074\H,-0.2353068739,2.5543875352,-0.9896243867
\H,-1.4666020015,2.4871536782,0.3088152304\Se,-0.1829619757,0.97254005
96,3.0159517723\Se,0.8540641473,2.1759006731,1.2680275742\\Version=EM6
4L-G16RevA.03\State=1-A'\HF=-4843.0688219\RMSD=5.524e-09\RMSF=6.728e-0
5\Dipole=0.0248419,0.4935361,-1.200062\Quadrupole=1.0080598,-0.1581788
,-0.8498809,-0.3871758,1.5182829,1.2964682\PG=CS [SG(C1H1Se2),X(H2)]\\
@

1\1\GINC-ORC391\FOpt\RM062X\6-311G(df,p)\C1H3Se2(1-)\MEISTERP\25-Jun-2
017\0\\# opt freq m062x/6-311g(df,p)\\Methyl Se Se optimization and fr
equency\\-1,1\C,-0.5567551412,2.0350915108,-0.0832241054\H,-0.74017526
2,0.9822145804,-0.2960220961\H,-0.233253049,2.5551116413,-0.9889510772
\H,-1.4683802034,2.4864494494,0.3075335093\Se,-0.178920344,0.976989716
5,3.0097382722\Se,0.8500481848,2.171494163,1.2741645105\\Version=EM64L
-G16RevA.03\State=1-A'\HF=-4843.0794755\RMSD=6.165e-09\RMSF=6.582e-05\
Dipole=0.0409709,0.5178811,-1.2412769\Quadrupole=1.0088052,-0.0729676,
-0.9358376,-0.5380846,1.6195193,1.4716756\PG=CS [SG(C1H1Se2),X(H2)]\\@

1\1\GINC-ORC233\FOpt\RM062X\6-311+G(2df,p)\C1H3Se2(1-)\MEISTERP\25-Jun
-2017\0\\# opt freq m062x/6-311+g(2df,p)\\Methyl Se Se optimization an
d frequency\\-1,1\C,-0.5591902432,2.0335127324,-0.0822273452\H,-0.7403
713105,0.9819551188,-0.2976916752\H,-0.2295483052,2.5547625457,-0.9836
111639\H,-1.4691552629,2.4873860365,0.3063438299\Se,-0.1764627839,0.97
90491242,3.007570741\Se,0.8472920907,2.1706855039,1.2728546262\\Versio
n=EM64L-G16RevA.03\State=1-A'\HF=-4843.081339\RMSD=5.624e-09\RMSF=5.59
0e-05\Dipole=0.0445208,0.5383601,-1.2880334\Quadrupole=0.9982767,-0.16
48618,-0.8334149,-0.5547968,1.7548828,1.4607208\PG=CS [SG(C1H1Se2),X(H
2)]\\@

```
1\1\GINC-ORC215\FOpt\RM062X\6-311++G(3df,3pd)\C1H3Se2(1-)\MEISTERP\25-
 Jun-2017\0\\# opt freq m062x/6-311++g(3df,3pd)\\Methyl Se Se optimizat
 ion and frequency\\-1,1\C,-0.559385252,2.033535613,-0.0825196533\H,-0.
 7399232387,0.9834491482,-0.2966950373\H,-0.2306628883,2.5537882542,-0.
 9825277206\H,-1.4675435476,2.4864763564,0.3063760092\Se,-0.1773976095,
 0.9789048448,3.0068024365\Se,0.8474767209,2.1711968449,1.2718029776\\V
 ersion=EM64L-G16RevA.03\State=1-A'\HF=-4843.0905442\RMSD=7.378e-09\RMS
 F=5.372e-05\Dipole=0.0645209,0.5335824,-1.2519954\Quadrupole=0.9985314
 ,-0.0724942,-0.9260372,-0.6020531,1.7008812,1.5289701\PG=CS [SG(C1H1Se
 2),X(H2)]\\@
```

CH_3SeSe^- M08HX

```
1\1\GINC-ORC390\FOpt\RM08HX\6-31G(d)\C1H3Se2(1-)\MEISTERP\25-Jun-2017\
 0\\# opt freq m08hx/6-31g(d)\\Methyl Se Se optimization and frequency\
 \-1,1\C,-0.5507656489,2.0355596113,-0.0771642747\H,-0.7333836119,0.974
 3449297,-0.2837695498\H,-0.2406844702,2.5545757562,-0.9965816925\H,-1.
 4659192979,2.4875257103,0.3233754919\Se,-0.1937157218,0.9816000661,2.9
 80396956\Se,0.8570329331,2.1737449882,1.2769820778\\Version=EM64L-G16R
 evA.03\State=1-A'\HF=-4838.8441918\RMSD=6.703e-09\RMSF=9.378e-05\Dipol
 e=0.0360393,0.5051784,-1.2155682\Quadrupole=1.0521166,-0.1320979,-0.92
 00187,-0.5212923,1.7061189,1.4819127\PG=CS [SG(C1H1Se2),X(H2)]\\@
```

```
1\1\GINC-ORC255\FOpt\RM08HX\6-311G(d)\C1H3Se2(1-)\MEISTERP\25-Jun-2017
 \0\\# opt freq m08hx/6-311g(d)\\Methyl Se Se optimization and frequenc
 y\\-1,1\C,-0.5532048095,2.0356564618,-0.0803485624\H,-0.7376493913,0.9
 773913575,-0.2889639004\H,-0.2390370633,2.5550247183,-0.9957129979\H,-
 1.4680844633,2.4862329518,0.3164400965\Se,-0.1915624441,0.9753262057,2
 .9986312113\Se,0.8621023554,2.1777193667,1.2731931636\\Version=EM64L-G
 16RevA.03\State=1-A'\HF=-4843.0883723\RMSD=8.243e-09\RMSF=8.785e-05\Di
 pole=0.0631969,0.5456244,-1.2836049\Quadrupole=1.0055411,-0.2132364,-0
 .7923047,-0.4157597,1.6283457,1.3127267\PG=CS [SG(C1H1Se2),X(H2)]\\@
```

```
1\1\GINC-ORC236\FOpt\RM08HX\6-311G(d,p)\C1H3Se2(1-)\MEISTERP\25-Jun-20
 17\0\\# opt freq m08hx/6-311g(d,p)\\Methyl Se Se optimization and freq
 uency\\-1,1\C,-0.5547149346,2.0354702935,-0.081706581\H,-0.7366900491,
 0.9765512105,-0.2878535\H,-0.2373497646,2.5555445366,-0.9949727671\H,-
 1.4677211887,2.4866240869,0.3180445335\Se,-0.191551138,0.9755293222,2.
 9981386274\Se,0.8605912612,2.1776316114,1.2715887022\\Version=EM64L-G1
 6RevA.03\State=1-A'\HF=-4843.0922469\RMSD=6.866e-09\RMSF=8.668e-05\Dip
 ole=0.0661964,0.5452393,-1.2790263\Quadrupole=1.0114365,-0.2002602,-0.
 8111763,-0.4134364,1.6126424,1.3183587\PG=CS [SG(C1H1Se2),X(H2)]\\@
```

```
1\1\GINC-ORC234\FOpt\RM08HX\6-311+G(d,p)\C1H3Se2(1-)\MEISTERP\25-Jun-2
 017\0\\# opt freq m08hx/6-311+g(d,p)\\Methyl Se Se optimization and fr
 equency\\-1,1\C,-0.5572762888,2.0339929678,-0.0811149965\H,-0.73826871
 27,0.9758372794,-0.2910598043\H,-0.2326244067,2.5560788741,-0.99060322
 8\H,-1.4701576186,2.4876820224,0.3155491687\Se,-0.1876183407,0.9773960
 661,2.9982311809\Se,0.8585095542,2.1763638515,1.2722366946\\Version=EM
 64L-G16RevA.03\State=1-A'\HF=-4843.0984107\RMSD=8.874e-09\RMSF=7.474e-
 05\Dipole=0.0846143,0.5892398,-1.3664664\Quadrupole=1.0199801,-0.24737
 39,-0.7726061,-0.4802336,1.7744196,1.3861911\PG=CS [SG(C1H1Se2),X(H2)]
 \\@
```

```
1\1\GINC-ORC212\FOpt\RM08HX\6-311G(2d,p)\C1H3Se2(1-)\MEISTERP\25-Jun-2
 017\0\\# opt freq m08hx/6-311g(2d,p)\\Methyl Se Se optimization and fr
 equency\\-1,1\C,-0.5546889807,2.0352619781,-0.0811560847\H,-0.73546035
```

```
 37,0.9771968492,-0.286685653\H,-0.2379352018,2.5546288071,-0.993396849
 4\H,-1.4661314217,2.4865259348,0.3189139437\Se,-0.1931069625,0.9748170
 465,2.9980366827\Se,0.8598871084,2.1789204458,1.2675269768\\Version=EM
 64L-G16RevA.03\State=1-A'\HF=-4843.0889276\RMSD=9.875e-09\RMSF=8.670e-
 05\Dipole=0.0478369,0.511108,-1.2161122\Quadrupole=1.0016845,-0.184292
 7,-0.8173918,-0.3764359,1.5324249,1.274116\PG=CS [SG(C1H1Se2),X(H2)]\\
 @

1\1\GINC-ORC186\FOpt\RM08HX\6-311G(df,p)\C1H3Se2(1-)\MEISTERP\25-Jun-2
 017\0\\# opt freq m08hx/6-311g(df,p)\\Methyl Se Se optimization and fr
 equency\\-1,1\C,-0.5533056843,2.0353423173,-0.0796873316\H,-0.73796478
 4,0.9770094637,-0.2886659153\H,-0.2351931523,2.5556324395,-0.992589839
 9\H,-1.468475939,2.4860082208,0.3168011412\Se,-0.1882317535,0.97887220
 79,2.9938121114\Se,0.8557354982,2.1744864118,1.2735688485\\Version=EM6
 4L-G16RevA.03\State=1-A'\HF=-4843.0996191\RMSD=6.709e-09\RMSF=9.373e-0
 5\Dipole=0.0640631,0.5354682,-1.2572475\Quadrupole=1.005109,-0.0914928
 ,-0.9136161,-0.5375328,1.6401035,1.4639386\PG=CS [SG(C1H1Se2),X(H2)]\\
 @

1\1\GINC-ORC172\FOpt\RM08HX\6-311+G(2df,p)\C1H3Se2(1-)\MEISTERP\25-Jun
 -2017\0\\# opt freq m08hx/6-311+g(2df,p)\\Methyl Se Se optimization an
 d frequency\\-1,1\C,-0.5560845503,2.0335533516,-0.0785814837\H,-0.7386
 59293,0.9768034179,-0.2910946558\H,-0.2306891552,2.5554105513,-0.98660
 26452\H,-1.4697563159,2.4870123878,0.3148579837\Se,-0.1847325506,0.981
 3722153,2.9918032467\Se,0.8524860477,2.1731991384,1.2728565621\\Versio
 n=EM64L-G16RevA.03\State=1-A'\HF=-4843.1024707\RMSD=6.460e-09\RMSF=7.7
 31e-05\Dipole=0.0738436,0.5458833,-1.2714046\Quadrupole=1.0008199,-0.1
 518841,-0.8489358,-0.5417378,1.7215504,1.4520937\PG=CS [SG(C1H1Se2),X(
 H2)]\\@

1\1\GINC-ORC363\FOpt\RM08HX\6-311++G(3df,3pd)\C1H3Se2(1-)\MEISTERP\25-
 Jun-2017\0\\# opt freq m08hx/6-311++g(3df,3pd)\\Methyl Se Se optimizat
 ion and frequency\\-1,1\C,-0.5569561051,2.0335503838,-0.0796256399\H,-
 0.7371435062,0.9784023657,-0.2884566937\H,-0.2322347367,2.5540911187,-
 0.9851790209\H,-1.4669057777,2.4858541729,0.3163896684\Se,-0.187008432
 2,0.9813110775,2.9892097115\Se,0.8528127446,2.1741419424,1.2709009902\
 \Version=EM64L-G16RevA.03\State=1-A'\HF=-4843.1114529\RMSD=6.556e-09\R
 MSF=7.436e-05\Dipole=0.0986074,0.5420511,-1.2319757\Quadrupole=0.97566
 34,-0.0743336,-0.9013297,-0.5793267,1.6539463,1.4839319\PG=CS [SG(C1H1
 Se2),X(H2)]\\@
```

CH_3SeSe^- MP2

```
1\1\GINC-ORC392\FOpt\RMP2-FC\6-31G(d)\C1H3Se2(1-)\MEISTERP\25-Jun-2017
 \0\\# opt freq mp2/6-31g(d)\\Methyl Se Se optimization and frequency\\
 -1,1\C,-0.5555172941,2.0356800595,-0.0831974452\H,-0.735280864,0.97970
 57915,-0.2908496356\H,-0.2414315228,2.5540477191,-0.9961670092\H,-1.46
 54306155,2.4879580066,0.3143178802\Se,-0.1886280629,0.9741456636,3.005
 1138676\Se,0.8588525437,2.1758138214,1.2740213536\\Version=EM64L-G16Re
 vA.03\State=1-A'\HF=-4834.808778\MP2=-4835.1648116\RMSD=2.477e-09\RMSF
 =6.137e-05\Dipole=0.0097801,0.5049319,-1.2466363\PG=CS [SG(C1H1Se2),X(
 H2)]\\@

1\1\GINC-ORC391\FOpt\RMP2-FC\6-311G(d)\C1H3Se2(1-)\MEISTERP\25-Jun-201
 7\0\\# opt freq mp2/6-311g(d)\\Methyl Se Se optimization and frequency
 \\-1,1\C,-0.5566484691,2.0359609398,-0.0852622709\H,-0.7404988943,0.98
 19672317,-0.2974754287\H,-0.2373773675,2.5556761284,-0.9953340356\H,-1
 .4691713795,2.4871678944,0.3064676894\Se,-0.1837593361,0.9683288384,3.
```

0254853164\Se,0.8600196328,2.1782500272,1.2693577474\\Version=EM64L-G1
6RevA.03\State=1-A'\HF=-4839.2658404\MP2=-4839.6504843\RMSD=5.010e-09\
RMSF=5.951e-05\Dipole=0.0373408,0.5342213,-1.2863811\PG=CS [SG(C1H1Se2
),X(H2)]\\@

1\1\GINC-ORC390\FOpt\RMP2-FC\6-311G(d,p)\C1H3Se2(1-)\MEISTERP\25-Jun-2
017\0\\# opt freq mp2/6-311g(d,p)\\Methyl Se Se optimization and frequ
ency\\-1,1\C,-0.5558691478,2.0356421264,-0.0835274252\H,-0.7405442859,
0.9806753895,-0.2969529557\H,-0.2357927034,2.5560756159,-0.9944177368\
H,-1.4699524471,2.4873957197,0.3075999103\Se,-0.1842540407,0.968982372
9,3.0232596462\Se,0.8589768122,2.1785798363,1.2672775787\\Version=EM64
L-G16RevA.03\State=1-A'\HF=-4839.2709209\MP2=-4839.674001\RMSD=4.631e-
09\RMSF=6.978e-05\Dipole=0.0644889,0.531155,-1.2459841\PG=CS [SG(C1H1S
e2),X(H2)]\\@

1\1\GINC-ORC389\FOpt\RMP2-FC\6-311+G(d,p)\C1H3Se2(1-)\MEISTERP\25-Jun-
2017\0\\# opt freq mp2/6-311+g(d,p)\\Methyl Se Se optimization and fre
quency\\-1,1\C,-0.5580043767,2.0343187821,-0.0828054762\H,-0.740775433
9,0.9794241679,-0.2979838439\H,-0.2327541013,2.5561461002,-0.990927254
4\H,-1.4712611612,2.4883703998,0.3074621375\Se,-0.1833536208,0.9704527
482,3.0206814253\Se,0.8587128822,2.1786388621,1.2668120312\\Version=EM
64L-G16RevA.03\State=1-A'\HF=-4839.2764502\MP2=-4839.6827269\RMSD=7.15
7e-09\RMSF=6.445e-05\Dipole=0.0780693,0.5966955,-1.3929451\PG=CS [SG(C
1H1Se2),X(H2)]\\@

1\1\GINC-ORC138\FOpt\RMP2-FC\6-311G(2d,p)\C1H3Se2(1-)\MEISTERP\25-Jun-
2017\0\\# opt freq mp2/6-311g(2d,p)\\Methyl Se Se optimization and fre
quency\\-1,1\C,-0.5564185774,2.0353312419,-0.0834155118\H,-0.740479820
6,0.9827480017,-0.2986456582\H,-0.236006287,2.5548843901,-0.991706552\
H,-1.4689427561,2.4875158027,0.3051237796\Se,-0.1832163186,0.967584815
3,3.0279948011\Se,0.8576279464,2.1792868093,1.2638881577\\Version=EM64
L-G16RevA.03\State=1-A'\HF=-4839.2667615\MP2=-4839.688517\RMSD=4.294e-
09\RMSF=7.698e-05\Dipole=0.0419226,0.5061235,-1.2108251\PG=CS [SG(C1H1
Se2),X(H2)]\\@

1\1\GINC-ORC137\FOpt\RMP2-FC\6-311G(df,p)\C1H3Se2(1-)\MEISTERP\25-Jun-
2017\0\\# opt freq mp2/6-311g(df,p)\\Methyl Se Se optimization and fre
quency\\-1,1\C,-0.5546549429,2.0345945378,-0.0794515633\H,-0.740170420
1,0.9797234447,-0.2943686097\H,-0.2308776186,2.5560336692,-0.988383020
7\H,-1.4696451923,2.4865813716,0.3102394652\Se,-0.1804456396,0.9784225
587,3.0043269082\Se,0.8483579984,2.1719954791,1.2708758339\\Version=EM
64L-G16RevA.03\State=1-A'\HF=-4839.2783487\MP2=-4839.7429438\RMSD=3.56
9e-09\RMSF=6.685e-05\Dipole=0.0528965,0.5176316,-1.2262664\PG=CS [SG(C
1H1Se2),X(H2)]\\@

1\1\GINC-ORC125\FOpt\RMP2-FC\6-311+G(2df,p)\C1H3Se2(1-)\MEISTERP\25-Ju
n-2017\0\\# opt freq mp2/6-311+g(2df,p)\\Methyl Se Se optimization and
 frequency\\-1,1\C,-0.5569491548,2.0331851397,-0.0787069604\H,-0.74083
73906,0.9803001847,-0.2966087561\H,-0.2294145842,2.5549055117,-0.98380
61388\H,-1.4703116135,2.487156977,0.3079988636\Se,-0.1789858754,0.9798
255513,3.0025914874\Se,0.8490628073,2.1719776952,1.2717705262\\Version
=EM64L-G16RevA.03\State=1-A'\HF=-4839.2799049\MP2=-4839.7620424\RMSD=4
.321e-09\RMSF=6.770e-05\Dipole=0.0559466,0.5562867,-1.3189262\PG=CS [S
G(C1H1Se2),X(H2)]\\@

1\1\GINC-ORC256\FOpt\RMP2-FC\6-311++G(3df,3pd)\C1H3Se2(1-)\MEISTERP\25
-Jun-2017\0\\# opt freq mp2/6-311++g(3df,3pd)\\Methyl Se Se optimizati

on and frequency\\-1,1\C,-0.5563090156,2.0330831017,-0.0776803082\H,-0
.7370683589,0.9813846144,-0.2886148196\H,-0.2333196694,2.5523786576,-0
.9822200718\H,-1.4648305688,2.484704944,0.3145738382\Se,-0.1874961493,
0.9803854743,2.9909281393\Se,0.8515879481,2.1754142692,1.2662522367\\V
ersion=EM64L-G16RevA.03\State=1-A'\HF=-4839.295609\MP2=-4839.7938248\R
MSD=4.880e-09\RMSF=7.061e-05\Dipole=0.0899692,0.5500963,-1.2624487\PG=
CS [SG(C1H1Se2),X(H2)]\\@

CH_3SeSe^- QCISD

1\1\GINC-ORC297\FOpt\RQCISD-FC\6-311+G(2df,p)\C1H3Se2(1-)\MEISTERP\21-
Aug-2017\0\\# opt freq=noraman qcisd/6-311+g(2df,p) guess=read\\Methyl
Se Se optimization and frequency\\-1,1\C,1.73594,1.271997,0.000005\H,
1.422003,1.821077,-0.89006\H,2.824735,1.148195,-0.000855\H,1.423492,1.
820858,0.890721\Se,-1.372291,0.136008,0.000001\Se,0.899177,-0.501364,0
.000004\\Version=EM64L-G09RevE.01\State=1-A\HF=-4839.2799069\MP2=-4839
.7619194\MP3=-4839.7986568\MP4D=-4839.8113508\MP4DQ=-4839.7967987\MP4S
DQ=-4839.8003283\QCISD=-4839.8002032\RMSD=2.371e-09\RMSF=3.884e-05\Dip
ole=1.3984241,0.2659854,-0.0000436\PG=C01 [X(C1H3Se2)]\\@

CH_3SeSH B3LYP

1\1\GINC-ORC150\FOpt\RB3LYP\6-31G(d)\C1H4S1Se1\MEISTERP\20-Jul-2017\0\
\# opt=readfc freq=noraman b3lyp/6-31g(d) guess=read\\Methyl Selenium
Sulfide\\0,1\C,1.3183156068,1.1666713663,0.0395750211\H,1.0804912121,1
.8913589996,-0.738787809\H,2.3993628355,1.0030970396,0.0793255581\H,0.
9565601847,1.4956167634,1.0137791394\H,-2.0029065729,-0.1808516313,0.6
567318224\Se,0.5279697721,-0.582152864,-0.4198561608\S,-1.6135700383,-
0.0134826736,-0.6306755711\\Version=EM64L-G09RevE.01\State=1-A\HF=-283
8.0899861\RMSD=8.604e-09\RMSF=7.764e-06\Dipole=0.3557614,0.4080857,0.5
272625\Quadrupole=1.2979302,-0.6146733,-0.6832569,0.9982945,-1.3492989
,0.3173624\PG=C01 [X(C1H4S1Se1)]\\@

1\1\GINC-ORC260\FOpt\RB3LYP\6-311G(d)\C1H4S1Se1\MEISTERP\01-Aug-2017\0
\\# opt=readfc freq=noraman b3lyp/6-311g(d) guess=read\\Methyl Seleniu
m Sulfide\\0,1\C,1.3190535364,1.1640100404,0.0402982845\H,1.0772185999
,1.8887210873,-0.7336605708\H,2.3992530443,1.0099965542,0.0772700871\H
,0.9618520227,1.4998400678,1.0114887437\H,-2.0059994217,-0.1822440552,
0.6540557254\Se,0.5337100166,-0.5908745372,-0.4155979041\S,-1.61886479
82,-0.0091921573,-0.6337623657\\Version=EM64L-G09RevE.01\State=1-A\HF=
-2840.2938342\RMSD=6.714e-09\RMSF=1.210e-05\Dipole=0.3630453,0.4015627
,0.5352213\Quadrupole=1.3576764,-0.6957592,-0.6619173,0.9220767,-1.405
9131,0.2762775\PG=C01 [X(C1H4S1Se1)]\\@

1\1\GINC-ORC105\FOpt\RB3LYP\6-311G(d,p)\C1H4S1Se1\MEISTERP\20-Jul-2017
\0\\# opt=readfc freq=noraman b3lyp/6-311g(d,p) guess=read\\Methyl Sel
enium Sulfide\\0,1\C,1.3190654803,1.1660241804,0.0399587975\H,1.081858
1036,1.888835975,-0.7372299369\H,2.3978491695,1.0029721273,0.080388431
5\H,0.9573510255,1.500331993,1.0099074043\H,-1.9979590461,-0.185928719
5,0.6556800135\Se,0.5313159712,-0.5864056171,-0.4175183768\S,-1.623257
7042,-0.005572939,-0.6310943332\\Version=EM64L-G09RevE.01\State=1-A\HF
=-2840.3031471\RMSD=7.884e-09\RMSF=1.345e-05\Dipole=0.3669266,0.401483
9,0.5193182\Quadrupole=1.3308217,-0.6732989,-0.6575229,0.9445228,-1.32
73686,0.2696204\PG=C01 [X(C1H4S1Se1)]\\@

1\1\GINC-ORC102\FOpt\RB3LYP\6-311+G(d,p)\C1H4S1Se1\MEISTERP\20-Jul-201
7\0\\# opt=readfc freq=noraman b3lyp/6-311+g(d,p) guess=read\\Methyl S

elenium Sulfide\\0,1\C,1.3197474778,1.1665686124,0.0403685735\H,1.0808
12095,1.8895709661,-0.7363258941\H,2.3986581832,1.0024360368,0.0772730
764\H,0.9617632742,1.5017443458,1.0115249508\H,-2.0006680106,-0.187091
5944,0.6542369823\Se,0.5301918821,-0.5856637278,-0.4147189082\S,-1.624
2819017,-0.007307639,-0.6322667807\\Version=EM64L-G09RevE.01\State=1-A
\HF=-2840.3044788\RMSD=7.354e-09\RMSF=1.077e-05\Dipole=0.3327369,0.403
0372,0.5121192\Quadrupole=1.3991307,-0.6894737,-0.709657,1.014101,-1.3
065518,0.2829388\PG=C01 [X(C1H4S1Se1)]\\@

1\1\GINC-ORC101\FOpt\RB3LYP\6-311G(2d,p)\C1H4S1Se1\MEISTERP\20-Jul-201
7\0\\# opt=readfc freq=noraman b3lyp/6-311g(2d,p) guess=read\\Methyl S
elenium Sulfide\\0,1\C,1.3190202935,1.1670578158,0.0396946112\H,1.0837
982225,1.8885510457,-0.7376551539\H,2.3960222732,0.9995159207,0.080161
4606\H,0.9576683327,1.5015409415,1.0083042496\H,-1.9943962247,-0.18776
78786,0.6535018985\Se,0.5246209395,-0.5808605172,-0.415226296\S,-1.620
5108366,-0.0077803279,-0.6286887699\\Version=EM64L-G09RevE.01\State=1-
A\HF=-2840.3064206\RMSD=4.286e-09\RMSF=8.372e-06\Dipole=0.3536559,0.40
2581,0.4604623\Quadrupole=1.4156964,-0.7639887,-0.6517077,0.810093,-1.
1329844,0.2348856\PG=C01 [X(C1H4S1Se1)]\\@

1\1\GINC-ORC97\FOpt\RB3LYP\6-311G(df,p)\C1H4S1Se1\MEISTERP\20-Jul-2017
\0\\# opt=readfc freq=noraman b3lyp/6-311g(df,p) guess=read\\Methyl Se
lenium Sulfide\\0,1\C,1.3156597906,1.1637359405,0.039404292\H,1.079802
0178,1.8884418245,-0.7364445321\H,2.3941021902,1.0002509983,0.08100487
3\H,0.9535480443,1.4987993456,1.0089891855\H,-1.991792029,-0.181901697
2,0.6561603569\Se,0.5274526927,-0.5818468807,-0.4187886721\S,-1.612549
7067,-0.0072225309,-0.6302335033\\Version=EM64L-G09RevE.01\State=1-A\H
F=-2840.3097154\RMSD=4.546e-09\RMSF=7.344e-06\Dipole=0.3560057,0.40286
18,0.5168838\Quadrupole=1.2211143,-0.6518738,-0.5692405,0.9857452,-1.3
287697,0.2661262\PG=C01 [X(C1H4S1Se1)]\\@

1\1\GINC-ORC90\FOpt\RB3LYP\6-311+G(2df,p)\C1H4S1Se1\MEISTERP\20-Jul-20
17\0\\# opt=readfc freq=noraman b3lyp/6-311+g(2df,p) guess=read\\Methy
l Selenium Sulfide\\0,1\C,1.3164801264,1.1649598014,0.0396694162\H,1.0
804526942,1.8892714107,-0.7352721512\H,2.3936628863,0.9973421803,0.076
9509623\H,0.9595946618,1.501102967,1.0097070196\H,-1.9928764617,-0.185
2691617,0.652536317\Se,0.5203059431,-0.5772409441,-0.4137892971\S,-1.6
1139685,-0.0099092536,-0.6297102668\\Version=EM64L-G09RevE.01\State=1-
A\HF=-2840.3133702\RMSD=8.212e-09\RMSF=7.076e-06\Dipole=0.3156059,0.39
71687,0.4556187\Quadrupole=1.3716953,-0.7460637,-0.6256316,0.9426202,-
1.1280664,0.2491616\PG=C01 [X(C1H4S1Se1)]\\@

1\1\GINC-ORC85\FOpt\RB3LYP\6-311++G(3df,3pd)\C1H4S1Se1\MEISTERP\20-Jul
-2017\0\\# opt=readfc freq=noraman b3lyp/6-311++g(3df,3pd) guess=read\
\Methyl Selenium Sulfide\\0,1\C,1.3160743872,1.1656108134,0.0390395871
\H,1.083261659,1.8875631075,-0.7366673986\H,2.3911936594,0.997352039,0
.0811662138\H,0.9540280907,1.5006050552,1.0055654178\H,-1.989912953,-0
.1855800252,0.6532234207\Se,0.5210701955,-0.5764585761,-0.4158919527\S
,-1.6094920387,-0.0088354138,-0.626343288\\Version=EM64L-G09RevE.01\St
ate=1-A\HF=-2840.3205441\RMSD=5.553e-09\RMSF=6.694e-06\Dipole=0.333864
4,0.3886968,0.420478\Quadrupole=1.2743521,-0.7117184,-0.5626337,0.9138
871,-0.9987805,0.2318238\PG=C01 [X(C1H4S1Se1)]\\@

CH_3SeSH B3PW91

1\1\GINC-ORC80\FOpt\RB3PW91\6-31G(d)\C1H4S1Se1\MEISTERP\20-Jul-2017\0\
\# opt=readfc freq=noraman b3pw91/6-31g(d) guess=read\\Methyl Selenium

```
 Sulfide\\0,1\C,1.3097258099,1.1606749151,0.0390098354\H,1.0744643875,
1.887548316,-0.7386930206\H,2.39121441,0.9975422133,0.078070687\H,0.95
14957279,1.4916292762,1.0142740502\H,-1.9882635372,-0.1755204729,0.654
1973327\Se,0.5242786982,-0.572730571,-0.4168482875\S,-1.5966924963,-0.
0088866766,-0.6299185971\\Version=EM64L-G09RevE.01\State=1-A\HF=-2838.
006124\RMSD=7.861e-09\RMSF=7.044e-06\Dipole=0.3471627,0.4272435,0.5453
935\Quadrupole=1.3414808,-0.6432228,-0.698258,1.0392563,-1.3866713,0.3
2152\PG=C01 [X(C1H4S1Se1)]\\@

1\1\GINC-ORC313\FOpt\RB3PW91\6-311G(d)\C1H4S1Se1\MEISTERP\01-Aug-2017\
0\\# opt=readfc freq=noraman b3pw91/6-311g(d) guess=read\\Methyl Selen
ium Sulfide\\0,1\C,1.3102017886,1.1579079441,0.0397278717\H,1.07128418
99,1.8856846188,-0.7334974054\H,2.3911905845,1.0037356683,0.076226025\
H,0.9563430103,1.4961137587,1.0122882894\H,-1.9914231365,-0.1771555866
,0.6523882741\Se,0.5291068297,-0.5815417963,-0.4136977509\S,-1.6004802
664,-0.0044876071,-0.6333433039\\Version=EM64L-G09RevE.01\State=1-A\HF
=-2840.2077432\RMSD=3.680e-09\RMSF=6.514e-06\Dipole=0.3484141,0.415932
1,0.5516987\Quadrupole=1.3829932,-0.7031135,-0.6798798,0.9682894,-1.43
63709,0.2861504\PG=C01 [X(C1H4S1Se1)]\\@

1\1\GINC-ORC367\FOpt\RB3PW91\6-311G(d,p)\C1H4S1Se1\MEISTERP\20-Jul-201
7\0\\# opt=readfc freq=noraman b3pw91/6-311g(d,p) guess=read\\Methyl S
elenium Sulfide\\0,1\C,1.3123213707,1.1558900809,-0.0399251582\H,0.959
7861406,1.4841091642,-1.0163220428\H,2.3913139009,0.986812172,-0.07376
92917\H,1.078441715,1.89107255,0.7278236631\H,-1.9829187828,-0.1786965
611,-0.6563501282\Se,0.5188585133,-0.5725784518,0.4300147837\S,-1.6115
798577,0.0136480458,0.6286201741\\Version=EM64L-G09RevE.01\State=1-A\H
F=-2840.2171086\RMSD=5.319e-09\RMSF=2.860e-05\Dipole=0.3571072,0.40854
16,-0.5371896\Quadrupole=1.3479382,-0.6938395,-0.6540987,0.99802,1.347
6681,-0.2795448\PG=C01 [X(C1H4S1Se1)]\\@

1\1\GINC-ORC366\FOpt\RB3PW91\6-311+G(d,p)\C1H4S1Se1\MEISTERP\20-Jul-20
17\0\\# opt=readfc freq=noraman b3pw91/6-311+g(d,p) guess=read\\Methyl
 Selenium Sulfide\\0,1\C,1.3108460192,1.1603393687,0.0397027053\H,1.07
53716734,1.8867639541,-0.7361594918\H,2.3905645027,0.9959446063,0.0764
527091\H,0.9561823834,1.4980265344,1.0122339107\H,-1.9858225148,-0.182
4429217,0.6529055066\Se,0.5254428186,-0.5760422627,-0.4131891164\S,-1.
6063618825,-0.002332279,-0.6318542235\\Version=EM64L-G09RevE.01\State=
1-A\HF=-2840.2184732\RMSD=9.051e-09\RMSF=6.375e-06\Dipole=0.3250602,0.
4140106,0.5273193\Quadrupole=1.4130966,-0.6939517,-0.7191449,1.0528456
,-1.3341034,0.2895109\PG=C01 [X(C1H4S1Se1)]\\@

1\1\GINC-ORC365\FOpt\RB3PW91\6-311G(2d,p)\C1H4S1Se1\MEISTERP\20-Jul-20
17\0\\# opt=readfc freq=noraman b3pw91/6-311g(2d,p) guess=read\\Methyl
 Selenium Sulfide\\0,1\C,1.3105059707,1.1612011953,0.0392837782\H,1.07
76838812,1.8857602097,-0.7374064716\H,2.3884731367,0.9930459702,0.0782
950323\H,0.9533435944,1.4979983682,1.0098019891\H,-1.9802303901,-0.183
548708,0.6517460151\Se,0.5196242132,-0.5713063747,-0.412578403\S,-1.60
31774061,-0.0028936608,-0.6290499402\\Version=EM64L-G09RevE.01\State=1
-A\HF=-2840.2207451\RMSD=6.076e-09\RMSF=6.394e-06\Dipole=0.3416273,0.4
180277,0.4720695\Quadrupole=1.4486317,-0.7758484,-0.6727833,0.8456136,
-1.1448411,0.2399398\PG=C01 [X(C1H4S1Se1)]\\@

1\1\GINC-ORC365\FOpt\RB3PW91\6-311G(df,p)\C1H4S1Se1\MEISTERP\20-Jul-20
17\0\\# opt=readfc freq=noraman b3pw91/6-311g(df,p) guess=read\\Methyl
 Selenium Sulfide\\0,1\C,1.3069081709,1.1576741014,0.0388604156\H,1.07
39780284,1.8856288716,-0.7361619109\H,2.3861581492,0.9937070424,0.0797
```

126774\H,0.9484387203,1.4951178911,1.0099940339\H,-1.9764646458,-0.176
913364,0.6546990372\Se,0.5225637985,-0.5722874678,-0.4167183309\S,-1.5
953592215,-0.0026700747,-0.6302939224\\Version=EM64L-G09RevE.01\State=
1-A\HF=-2840.2239648\RMSD=9.712e-09\RMSF=3.101e-05\Dipole=0.3419855,0.
4178476,0.5316781\Quadrupole=1.2434078,-0.6569924,-0.5864153,1.0322377
,-1.3488517,0.2759087\PG=C01 [X(C1H4S1Se1)]\\@

1\1\GINC-ORC364\FOpt\RB3PW91\6-311+G(2df,p)\C1H4S1Se1\MEISTERP\20-Jul-
2017\0\\# opt=readfc freq=noraman b3pw91/6-311+g(2df,p) guess=read\\Me
thyl Selenium Sulfide\\0,1\C,1.3079830285,1.1589791252,0.0392890635\H,
1.0737023646,1.8862551174,-0.7349129003\H,2.3862446142,0.9914415903,0.
0750399435\H,0.9549849811,1.4970827878,1.0112572969\H,-1.9787406734,-0
.1802028423,0.650795217\Se,0.5160202603,-0.5683143187,-0.4112337835\S,
-1.5939715753,-0.0049844596,-0.6301428371\\Version=EM64L-G09RevE.01\St
ate=1-A\HF=-2840.2279179\RMSD=8.041e-09\RMSF=7.576e-06\Dipole=0.310075
7,0.4089595,0.4668905\Quadrupole=1.3931855,-0.7528377,-0.6403478,0.974
0769,-1.1412587,0.2554056\PG=C01 [X(C1H4S1Se1)]\\@

1\1\GINC-ORC364\FOpt\RB3PW91\6-311++G(3df,3pd)\C1H4S1Se1\MEISTERP\20-J
ul-2017\0\\# opt=readfc freq=noraman b3pw91/6-311++g(3df,3pd) guess=re
ad\\Methyl Selenium Sulfide\\0,1\C,1.3074827709,1.1593221693,0.0386471
978\H,1.0759094722,1.8845322021,-0.7362418182\H,2.3840679044,0.9918799
736,0.079228987\H,0.949039124,1.496112092,1.0073607434\H,-1.9756757584
,-0.1797513499,0.6517244505\Se,0.517202419,-0.5679310273,-0.4135922557
\S,-1.5918029321,-0.0039070598,-0.6270353048\\Version=EM64L-G09RevE.01
\State=1-A\HF=-2840.2348998\RMSD=5.772e-09\RMSF=7.272e-06\Dipole=0.329
9862,0.3997928,0.431445\Quadrupole=1.2895088,-0.7212207,-0.5682881,0.9
403143,-1.0138983,0.237655\PG=C01 [X(C1H4S1Se1)]\\@

CH_3SeSH wB97XD

1\1\GINC-ORC389\FOpt\RwB97XD\6-31G(d)\C1H4S1Se1\MEISTERP\20-Jul-2017\0
\\# opt=readfc freq=noraman wb97xd/6-31g(d) guess=read\\Methyl Seleniu
m Sulfide\\0,1\C,1.3032582681,1.1540055924,0.0390259852\H,1.0619220261
,1.8803178278,-0.7366904013\H,2.3857101177,1.003991092,0.0796921261\H,
0.9409001695,1.4816654439,1.0132004847\H,-1.9691663155,-0.1571252273,0
.6522455837\Se,0.5288200723,-0.5778528954,-0.4170167715\S,-1.585221338
2,-0.0047448334,-0.630365007\\Version=EM64L-G09RevE.01\State=1-A\HF=-2
838.0730357\RMSD=4.647e-09\RMSF=7.346e-06\Dipole=0.3586174,0.427717,0.
5540023\Quadrupole=1.295951,-0.6324597,-0.6634914,1.0351508,-1.4204012
,0.3572555\PG=C01 [X(C1H4S1Se1)]\\@

1\1\GINC-ORC305\FOpt\RwB97XD\6-311G(d)\C1H4S1Se1\MEISTERP\01-Aug-2017\
0\\# opt=readfc freq=noraman wb97xd/6-311g(d) guess=read\\Methyl Selen
ium Sulfide\\0,1\C,1.3034440279,1.1509048617,0.0397143157\H,1.05796765
94,1.8780581555,-0.7316509302\H,2.3856149934,1.010885764,0.078067921\H
,0.9448738058,1.4857125102,1.0112864274\H,-1.9717114035,-0.1573076761,
0.6502895201\Se,0.5339616584,-0.5873180794,-0.414090307\S,-1.587927741
4,-0.0006785358,-0.633524947\\Version=EM64L-G09RevE.01\State=1-A\HF=-2
840.2810864\RMSD=7.421e-09\RMSF=8.846e-06\Dipole=0.363121,0.4130624,0.
5607588\Quadrupole=1.3386684,-0.6899257,-0.6487427,0.95713,-1.4770591,
0.330383\PG=C01 [X(C1H4S1Se1)]\\@

1\1\GINC-ORC286\FOpt\RwB97XD\6-311G(d,p)\C1H4S1Se1\MEISTERP\20-Jul-201
7\0\\# opt=readfc freq=noraman wb97xd/6-311g(d,p) guess=read\\Methyl S
elenium Sulfide\\0,1\C,1.3032330746,1.1526339113,0.0393350937\H,1.0624
049093,1.8785053221,-0.734731784\H,2.3841832676,1.0042117728,0.0809456

807\H,0.9406700398,1.4859585884,1.0098540981\H,-1.9638740101,-0.160623
3881,0.652423457\Se,0.5316335706,-0.583028446,-0.4164024201\S,-1.59202
78518,0.0025992396,-0.6313321254\\Version=EM64L-G09RevE.01\State=1-A\H
F=-2840.2902127\RMSD=6.246e-09\RMSF=8.932e-06\Dipole=0.3678605,0.41188
31,0.5417719\Quadrupole=1.3069726,-0.6651246,-0.6418481,0.9754878,-1.3
853677,0.3234322\PG=C01 [X(C1H4S1Se1)]\\@

1\1\GINC-ORC285\FOpt\RwB97XD\6-311+G(d,p)\C1H4S1Se1\MEISTERP\20-Jul-20
17\0\\# opt=readfc freq=noraman wb97xd/6-311+g(d,p) guess=read\\Methyl
Selenium Sulfide\\0,1\C,1.3037869674,1.1527310448,0.0399952911\H,1.05
99391967,1.8789681026,-0.7329829804\H,2.3850312736,1.0042672292,0.0771
324448\H,0.9453720968,1.4863100148,1.0120767465\H,-1.9662221429,-0.160
085313,0.6506013185\Se,0.5309785889,-0.5828003182,-0.4135829445\S,-1.5
926629805,0.0008662398,-0.633147876\\Version=EM64L-G09RevE.01\State=1-
A\HF=-2840.2915926\RMSD=8.381e-09\RMSF=1.007e-05\Dipole=0.340583,0.411
4643,0.5358855\Quadrupole=1.3799184,-0.6837292,-0.6961891,1.0464233,-1
.3605062,0.3409128\PG=C01 [X(C1H4S1Se1)]\\@

1\1\GINC-ORC284\FOpt\RwB97XD\6-311G(2d,p)\C1H4S1Se1\MEISTERP\20-Jul-20
17\0\\# opt=readfc freq=noraman wb97xd/6-311g(2d,p) guess=read\\Methyl
Selenium Sulfide\\0,1\C,1.3037637899,1.1537325017,0.0394239217\H,1.06
31158201,1.8786417058,-0.7341931625\H,2.3833926949,1.0013447846,0.0783
912693\H,0.9441485503,1.4872641588,1.0097691774\H,-1.9631760019,-0.162
6092454,0.6492740215\Se,0.5253386218,-0.5776938542,-0.4122781238\S,-1.
5903604752,-0.0004230512,-0.6302951035\\Version=EM64L-G09RevE.01\State
=1-A\HF=-2840.2940055\RMSD=9.594e-09\RMSF=9.833e-06\Dipole=0.3506503,0
.4178474,0.48087\Quadrupole=1.4216452,-0.7681508,-0.6534944,0.8260453,
-1.1797841,0.2835586\PG=C01 [X(C1H4S1Se1)]\\@

1\1\GINC-ORC279\FOpt\RwB97XD\6-311G(df,p)\C1H4S1Se1\MEISTERP\20-Jul-20
17\0\\# opt=readfc freq=noraman wb97xd/6-311g(df,p) guess=read\\Methyl
Selenium Sulfide\\0,1\C,1.3005898361,1.1508023899,0.0389578189\H,1.06
01523061,1.8780369744,-0.7339874005\H,2.3812699917,1.0020239449,0.0809
01977\H,0.93789835,1.4847245288,1.0092779593\H,-1.9594962585,-0.157194
5426,0.6524220715\Se,0.5284032194,-0.5790726493,-0.4168752216\S,-1.582
5944449,0.000936354,-0.6306052047\\Version=EM64L-G09RevE.01\State=1-A\
HF=-2840.2973053\RMSD=2.652e-09\RMSF=1.380e-05\Dipole=0.356275,0.41424
25,0.5388439\Quadrupole=1.2022752,-0.6430754,-0.5591998,1.0201895,-1.3
85479,0.317502\PG=C01 [X(C1H4S1Se1)]\\@

1\1\GINC-ORC273\FOpt\RwB97XD\6-311+G(2df,p)\C1H4S1Se1\MEISTERP\20-Jul-
2017\0\\# opt=readfc freq=noraman wb97xd/6-311+g(2df,p) guess=read\\Me
thyl Selenium Sulfide\\0,1\C,1.301607291,1.1517402548,0.0395628247\H,1
.0589771381,1.8790846756,-0.7315915058\H,2.3815082443,1.0000600436,0.0
74954911\H,0.9461574776,1.4863670685,1.0113880576\H,-1.961987101,-0.15
90131736,0.6481353408\Se,0.5218819054,-0.5749628489,-0.4107839315\S,-1
.5819219554,-0.00301902,-0.6315736967\\Version=EM64L-G09RevE.01\State=
1-A\HF=-2840.3013748\RMSD=4.258e-09\RMSF=1.280e-05\Dipole=0.3193117,0.
4092888,0.4769551\Quadrupole=1.3787908,-0.7497589,-0.629032,0.9716813,
-1.1720611,0.3036193\PG=C01 [X(C1H4S1Se1)]\\@

1\1\GINC-ORC268\FOpt\RwB97XD\6-311++G(3df,3pd)\C1H4S1Se1\MEISTERP\20-J
ul-2017\0\\# opt=readfc freq=noraman wb97xd/6-311++g(3df,3pd) guess=re
ad\\Methyl Selenium Sulfide\\0,1\C,1.3009595903,1.1520237455,0.0390731
909\H,1.0602313378,1.8769788195,-0.732452122\H,2.3790605091,1.00109443
6,0.0786961427\H,0.9402993501,1.4844533578,1.007712985\H,-1.9584210407
,-0.1571730273,0.6490620103\Se,0.5236236595,-0.5750371718,-0.413072755

6\S,-1.5795304061,-0.0020831596,-0.6289274512\\Version=EM64L-G09RevE.0
1\State=1-A\HF=-2840.3086819\RMSD=6.785e-09\RMSF=1.344e-05\Dipole=0.33
53839,0.4043042,0.4424088\Quadrupole=1.2942713,-0.7274718,-0.5667995,0
.9426659,-1.0453508,0.2881407\PG=C01 [X(C1H4S1Se1)]\\@

CH_3SeSH M062X

1\1\GINC-ORC363\FOpt\RM062X\6-31G(d)\C1H4S1Se1\MEISTERP\20-Jul-2017\0\
\# opt=readfc freq=noraman m062x/6-31g(d) guess=read\\Methyl Selenium
Sulfide\\0,1\C,1.3031592803,1.1557221138,-0.0345923489\H,0.9136491852,
1.4910862547,-0.9943204123\H,2.3814801556,0.9969749361,-0.1061154368\H
,1.0854400143,1.8740714014,0.7537910489\H,-1.9578601006,-0.1885960297,
-0.662081081\Se,0.5246511503,-0.571143565,0.4293673543\S,-1.5842966851
,0.0221418888,0.6140428759\\Version=EM64L-G09RevE.01\State=1-A\HF=-283
8.0628691\RMSD=8.267e-09\RMSF=4.855e-05\Dipole=0.3229689,0.4243785,-0.
5411582\Quadrupole=1.3341764,-0.6923242,-0.6418522,1.1131138,1.3663922
,-0.2704768\PG=C01 [X(C1H4S1Se1)]\\@

1\1\GINC-ORC313\FOpt\RM062X\6-311G(d)\C1H4S1Se1\MEISTERP\01-Aug-2017\0
\\# opt=readfc freq=noraman m062x/6-311g(d) guess=read\\Methyl Seleniu
m Sulfide\\0,1\C,1.3698062812,1.1915156989,-0.0000738726\H,1.111623569
6,1.7502053574,-0.8966750484\H,2.4366868766,0.9743698348,0.0011067827\
H,1.1094613641,1.7497590505,0.8962121117\H,-2.2348870531,-0.765433887,
0.0019459261\Se,0.4603856018,-0.5324691705,-0.0018518872\S,-1.58685364
02,0.4123101159,-0.0005720122\\Version=EM64L-G09RevE.01\State=1-A\HF=-
2840.2202243\RMSD=6.046e-09\RMSF=2.300e-05\Dipole=0.3338527,0.0640236,
0.0013531\Quadrupole=2.6161098,0.6386777,-3.2547875,3.1456306,-0.00535
83,-0.0015992\PG=C01 [X(C1H4S1Se1)]\\@

1\1\GINC-ORC362\FOpt\RM062X\6-311G(d,p)\C1H4S1Se1\MEISTERP\20-Jul-2017
\0\\# opt=readfc freq=noraman m062x/6-311g(d,p) guess=read\\Methyl Sel
enium Sulfide\\0,1\C,1.3024511017,1.1551123099,-0.0363229233\H,0.91731
57853,1.4946185779,-0.9944518302\H,2.3805663236,1.0047285097,-0.100330
4736\H,1.0731876712,1.8695444672,0.7500888738\H,-1.9524558164,-0.18301
283,-0.6571027877\Se,0.5312743078,-0.5838759511,0.4184135136\S,-1.5861
163731,0.0231419164,0.6197976275\\Version=EM64L-G09RevE.01\State=1-A\H
F=-2840.2365506\RMSD=8.599e-09\RMSF=3.728e-05\Dipole=0.3393602,0.42130
49,-0.5400091\Quadrupole=1.3511196,-0.7086163,-0.6425032,1.0473562,1.3
649285,-0.2530288\PG=C01 [X(C1H4S1Se1)]\\@

1\1\GINC-ORC391\FOpt\RM062X\6-311+G(d,p)\C1H4S1Se1\MEISTERP\20-Jul-201
7\0\\# opt=readfc freq=noraman m062x/6-311+g(d,p) guess=read\\Methyl S
elenium Sulfide\\0,1\C,1.3032395094,1.1555287067,-0.0367650765\H,0.921
4355064,1.495583763,-0.9961543179\H,2.3815911924,1.0043551848,-0.09742
903\H,1.0720408912,1.8702758496,0.7490140446\H,-1.9553621237,-0.183982
0619,-0.6556498539\Se,0.5304495816,-0.5830823172,0.4160954995\S,-1.587
1715572,0.021577875,0.6209807342\\Version=EM64L-G09RevE.01\State=1-A\H
F=-2840.2380405\RMSD=7.452e-09\RMSF=3.566e-05\Dipole=0.3108195,0.41651
96,-0.5318549\Quadrupole=1.4236298,-0.7328565,-0.6907733,1.1306458,1.3
394059,-0.2686634\PG=C01 [X(C1H4S1Se1)]\\@

1\1\GINC-ORC391\FOpt\RM062X\6-311G(2d,p)\C1H4S1Se1\MEISTERP\20-Jul-201
7\0\\# opt=readfc freq=noraman m062x/6-311g(2d,p) guess=read\\Methyl S
elenium Sulfide\\0,1\C,1.303518005,1.1559038821,-0.0369234024\H,0.9218
650128,1.4933303289,-0.9958273448\H,2.3801464822,1.0004167022,-0.09684
91591\H,1.0735830577,1.870113829,0.7479162049\H,-1.9516368562,-0.18264
27871,-0.6536518753\Se,0.5243879381,-0.5778509753,0.4158150996\S,-1.58

56406397,0.0209860203,0.6196124772\\Version=EM64L-G09RevE.01\State=1-A
\HF=-2840.240705\RMSD=9.318e-09\RMSF=3.359e-05\Dipole=0.3258941,0.4273
914,-0.4752386\Quadrupole=1.4635914,-0.80839,-0.6552015,0.8731193,1.15
0031,-0.2134928\PG=C01 [X(C1H4S1Se1)]\\@

1\1\GINC-ORC390\FOpt\RM062X\6-311G(df,p)\C1H4S1Se1\MEISTERP\20-Jul-201
7\0\\# opt=readfc freq=noraman m062x/6-311g(df,p) guess=read\\Methyl S
elenium Sulfide\\0,1\C,1.3005019122,1.1534862656,-0.0358858999\H,0.916
6553647,1.4946362037,-0.9942209604\H,2.3784569304,1.0020165067,-0.0991
556163\H,1.0716435401,1.8699750526,0.7490719713\H,-1.9505238648,-0.182
5438838,-0.6568782507\Se,0.5271849561,-0.5788520352,0.4183183364\S,-1.
5776958387,0.0215388904,0.6188424196\\Version=EM64L-G09RevE.01\State=1
-A\HF=-2840.2436632\RMSD=8.955e-09\RMSF=3.657e-05\Dipole=0.3332132,0.4
204395,-0.5366507\Quadrupole=1.2262796,-0.6827781,-0.5435014,1.1032027
,1.3683992,-0.2421216\PG=C01 [X(C1H4S1Se1)]\\@

1\1\GINC-ORC390\FOpt\RM062X\6-311+G(2df,p)\C1H4S1Se1\MEISTERP\20-Jul-2
017\0\\# opt=readfc freq=noraman m062x/6-311+g(2df,p) guess=read\\Meth
yl Selenium Sulfide\\0,1\C,1.3018423268,1.1539924538,-0.0371500645\H,0
.9257300706,1.4928147773,-0.9982938394\H,2.3790535419,0.999034524,-0.0
920994126\H,1.0691063012,1.8711341704,0.7448791705\H,-1.9527193322,-0.
1802030851,-0.6522816171\Se,0.5206127539,-0.575006573,0.4140931821\S,-
1.5774026621,0.0184907325,0.6209445809\\Version=EM64L-G09RevE.01\State
=1-A\HF=-2840.2482222\RMSD=4.670e-09\RMSF=3.789e-05\Dipole=0.2965018,0
.4115244,-0.4676756\Quadrupole=1.4043957,-0.7886167,-0.615779,1.033223
6,1.1367039,-0.2319758\PG=C01 [X(C1H4S1Se1)]\\@

1\1\GINC-ORC389\FOpt\RM062X\6-311++G(3df,3pd)\C1H4S1Se1\MEISTERP\20-Ju
l-2017\0\\# opt=readfc freq=noraman m062x/6-311++g(3df,3pd) guess=read
\\Methyl Selenium Sulfide\\0,1\C,1.3011986898,1.1542839841,-0.03623174
58\H,0.9181793012,1.4916858621,-0.9929335077\H,2.3762654441,1.00016506
69,-0.0975461781\H,1.0720295101,1.8689378067,0.7464531514\H,-1.9486276
435,-0.1798718895,-0.6540010641\Se,0.5225223692,-0.5750898659,0.417173
6845\S,-1.575344671,0.0201460357,0.6171776598\\Version=EM64L-G09RevE.0
1\State=1-A\HF=-2840.2557476\RMSD=3.680e-09\RMSF=3.374e-05\Dipole=0.31
31633,0.4029196,-0.4310454\Quadrupole=1.3187581,-0.7607383,-0.5580198,
0.9955042,1.0054517,-0.2148338\PG=C01 [X(C1H4S1Se1)]\\@

CH_3SeSH M08HX

1\1\GINC-ORC22\FOpt\RM08HX\6-31G(d)\C1H4S1Se1\MEISTERP\21-Aug-2017\0\\
opt freq=noraman m08hx/6-31g(d) guess=read\\Methyl Selenium Sulfide\
\0,1\C,1.2914037597,1.1586918019,0.0316837241\H,1.0350437084,1.8633248
425,-0.7638934775\H,2.3793766323,1.0326159719,0.0802160991\H,0.9153632
155,1.5011240652,0.9995523128\H,-1.9414481763,-0.1561931276,0.66486494
21\Se,0.5478920216,-0.5950394862,-0.3896568184\S,-1.5614081613,-0.0242
670678,-0.6226747822\\Version=EM64L-G16RevA.03\State=1-A\HF=-2838.0944
274\RMSD=8.024e-09\RMSF=6.970e-06\Dipole=0.2912186,0.467725,0.533972\Q
uadrupole=1.3645196,-0.6760027,-0.6885169,1.024797,-1.3722058,0.33874\
PG=C01 [X(C1H4S1Se1)]\\@

1\1\GINC-ORC334\FOpt\RM08HX\6-311G(d)\C1H4S1Se1\MEISTERP\21-Aug-2017\0
\\# opt freq=noraman m08hx/6-311g(d) guess=read\\Methyl Selenium Sulfi
de\\0,1\C,1.3052250332,1.1308061414,0.0288609936\H,1.0255797737,1.8966
230561,-0.6964289181\H,2.3898557245,0.9872353973,0.0152533187\H,0.9837
999707,1.4144692965,1.0326401898\H,-1.9369438436,-0.1446976765,0.69731
36378\Se,0.5031191056,-0.5789131691,-0.4838526926\S,-1.604412764,0.074

7339544,-0.5936945292\\Version=EM64L-G16RevA.03\State=1-A\HF=-2840.244
981\RMSD=9.590e-09\RMSF=1.152e-05\Dipole=0.3331388,0.405106,0.5663281\
Quadrupole=1.3261853,-0.7971354,-0.5290499,1.0209564,-1.4391039,0.3111
046\PG=C01 [X(C1H4S1Se1)]\\@

1\1\GINC-ORC330\FOpt\RM08HX\6-311G(d,p)\C1H4S1Se1\MEISTERP\21-Aug-2017
\0\\# opt freq=noraman m08hx/6-311g(d,p) guess=read\\Methyl Selenium S
ulfide\\0,1\C,1.2988107978,1.1448494125,0.0350786315\H,1.0264677644,1.
8900277685,-0.7138896489\H,2.3840293977,1.0064045069,0.0463218893\H,0.
951912275,1.4435034586,1.0257035741\H,-1.9299032245,-0.1609360147,0.67
61483256\Se,0.5249257452,-0.5832061086,-0.4567510592\S,-1.5900197556,0
.0396139767,-0.6125197124\\Version=EM64L-G16RevA.03\State=1-A\HF=-2840
.2529351\RMSD=7.900e-09\RMSF=1.154e-05\Dipole=0.3217829,0.4209345,0.54
67199\Quadrupole=1.3256683,-0.7372361,-0.5884322,1.0256831,-1.3487656,
0.3123553\PG=C01 [X(C1H4S1Se1)]\\@

1\1\GINC-ORC327\FOpt\RM08HX\6-311+G(d,p)\C1H4S1Se1\MEISTERP\21-Aug-201
7\0\\# opt freq=noraman m08hx/6-311+g(d,p) guess=read\\Methyl Selenium
 Sulfide\\0,1\C,1.2993202716,1.1450269165,0.0346244397\H,1.0256163252,
1.8834059522,-0.7208638962\H,2.3848690674,1.0071709313,0.0453578466\H,
0.9544630794,1.4534458166,1.0231712551\H,-1.9353636179,-0.1530434175,0
.673966715\Se,0.5258508021,-0.5876599935,-0.4406928826\S,-1.5885329278
,0.0319107944,-0.6154714776\\Version=EM64L-G16RevA.03\State=1-A\HF=-28
40.2544463\RMSD=2.897e-09\RMSF=1.982e-05\Dipole=0.2919047,0.4195271,0.
5340381\Quadrupole=1.4075551,-0.7686964,-0.6388587,1.083312,-1.3318946
,0.3267522\PG=C01 [X(C1H4S1Se1)]\\@

1\1\GINC-ORC205\FOpt\RM08HX\6-311G(2d,p)\C1H4S1Se1\ROOT\21-Aug-2017\0\
\# opt freq=noraman m08hx/6-311g(2d,p) guess=read\\Methyl Selenium Sul
fide\\0,1\C,1.2922709597,1.1591164414,0.0502344974\H,1.0517516303,1.83
78911701,-0.7678376357\H,2.3753757222,1.0335626678,0.125883928\H,0.893
1838903,1.5290836882,0.9946673539\H,-1.9576707108,-0.1337109278,0.6058
89669\Se,0.5554990849,-0.6082324621,-0.3399385477\S,-1.5441875767,-0.0
374535776,-0.6688072647\\Version=EM64L-G16RevA.03\State=1-A\HF=-2840.2
571985\RMSD=7.533e-09\RMSF=1.371e-05\Dipole=0.2799793,0.4634436,0.4662
699\Quadrupole=1.5419191,-0.7666367,-0.7752824,0.7642236,-1.1093649,0.
3180793\PG=C01 [X(C1H4S1Se1)]\\@

1\1\GINC-ORC191\FOpt\RM08HX\6-311G(df,p)\C1H4S1Se1\MEISTERP\21-Aug-201
7\0\\# opt freq=noraman m08hx/6-311g(df,p) guess=read\\Methyl Selenium
 Sulfide\\0,1\C,1.2927851249,1.1510872123,-0.0387373167\H,0.9143125299
,1.5002153111,-1.001104069\H,2.3776592009,1.0197797233,-0.0907227163\H
,1.0412153367,1.8567880728,0.7547303925\H,-1.9424180334,-0.1401438301,
-0.6478386807\Se,0.5418804166,-0.5986603663,0.3860702178\S,-1.55921157
57,-0.0088091231,0.6376941724\\Version=EM64L-G16RevA.03\State=1-A\HF=-
2840.2604086\RMSD=8.315e-09\RMSF=1.606e-05\Dipole=0.3029272,0.4463415,
-0.5311399\Quadrupole=1.2529725,-0.6674434,-0.5855291,1.005797,1.35870
15,-0.3250041\PG=C01 [X(C1H4S1Se1)]\\@

1\1\GINC-ORC284\FOpt\RM08HX\6-311++G(3df,3pd)\C1H4S1Se1\MEISTERP\21-Au
g-2017\0\\# opt freq=noraman m08hx/6-311++g(3df,3pd) guess=read\\Methy
l Selenium Sulfide\\0,1\C,1.2983487696,1.1421251484,-0.0365426054\H,0.
9373902694,1.4620741287,-1.0117244768\H,2.3789914983,0.9972720203,-0.0
667910258\H,1.0404332476,1.8693375734,0.7303287631\H,-1.9347341683,-0.
134135697,-0.6621143042\Se,0.5187983716,-0.5851084254,0.426010149\S,-1
.5730049882,0.0286922516,0.6209255002\\Version=EM64L-G16RevA.03\State=
1-A\HF=-2840.2731772\RMSD=5.778e-09\RMSF=1.261e-05\Dipole=0.3023414,0.

4043279,-0.4405778\Quadrupole=1.3147756,-0.7941052,-0.5206703,0.929715
4,1.0368512,-0.2887766\PG=C01 [X(C1H4S1Se1)]\\@

CH_3SeSH QCISD

1\1\GINC-ORC238\FOpt\RQCISD-FC\6-311+G(2df,p)\C1H4S1Se1\MEISTERP\21-Au
g-2017\0\\# opt freq=noraman qcisd/6-311+g(2df,p) guess=read\\Methyl S
elenium Sulfide\\0,1\C,-1.4440382198,1.1593669805,-0.002709116\H,-1.25
0568118,1.7285876665,0.9058502235\H,-2.4976820944,0.871638169,-0.04007
15432\H,-1.1910336437,1.7465936274,-0.8845304966\Se,-0.4165539497,-0.5
009093487,0.0046092156\S,1.6213387312,0.3287991565,-0.0859433568\H,1.8
251612943,0.4629457488,1.2377610734\\Version=EM64L-G09RevE.01\State=1-
A\HF=-2837.5589554\MP2=-2838.054129\MP3=-2838.0974298\MP4D=-2838.11104
87\MP4DQ=-2838.0965417\MP4SDQ=-2838.0999582\QCISD=-2838.0998596\RMSD=6
.745e-09\RMSF=6.355e-06\Dipole=-0.3924847,0.4418167,0.3349577\PG=C01 [
X(C1H4S1Se1)]\\@

CH_3SeS^- B3LYP

1\1\GINC-ORC387\FOpt\RB3LYP\6-31G(d)\C1H3S1Se1(1-)\MEISTERP\03-Jul-201
7\0\\# opt freq b3lyp/6-31g(d)\\Methyl Selenium Sulfide\\-1,1\C,-0.507
1069356,2.0029127984,-0.0463553286\H,-0.4782189056,0.9239937325,-0.220
8685653\H,-0.1542357555,2.5358156409,-0.9391747072\H,-1.5300303679,2.3
083098606,0.1896870931\Se,0.6780888476,2.4501671647,1.4819662627\S,-0.
2512990886,1.2564445369,3.125954327\\Version=EM64L-G16RevA.03\State=1-
A'\HF=-2837.5277064\RMSD=4.215e-09\RMSF=3.847e-05\Dipole=0.3146556,0.7
869784,-1.8474197\Quadrupole=1.7623758,0.4668321,-2.2292079,-0.532549,
2.0281963,2.6979256\PG=CS [SG(C1H1S1Se1),X(H2)]\\@

1\1\GINC-ORC385\FOpt\RB3LYP\6-311G(d)\C1H3S1Se1(1-)\MEISTERP\03-Jul-20
17\0\\# opt freq b3lyp/6-311g(d)\\Methyl Selenium Sulfide\\-1,1\C,-0.5
066729209,2.0033592179,-0.0467486593\H,-0.4797067247,0.926312384,-0.21
82442754\H,-0.1562916242,2.5341923125,-0.9389681253\H,-1.527660526,2.3
055513453,0.1908056145\Se,0.685013391,2.4572901056,1.4756891897\S,-0.2
574838012,1.2509383685,3.1286753369\\Version=EM64L-G16RevA.03\State=1-
A'\HF=-2839.7447049\RMSD=7.889e-09\RMSF=6.020e-05\Dipole=0.3720139,0.8
697612,-1.9796001\Quadrupole=1.8129134,0.4814925,-2.2944058,-0.4782152
,2.0312243,2.712992\PG=CS [SG(C1H1S1Se1),X(H2)]\\@

1\1\GINC-ORC382\FOpt\RB3LYP\6-311G(d,p)\C1H3S1Se1(1-)\MEISTERP\03-Jul-
2017\0\\# opt freq b3lyp/6-311g(d,p)\\Methyl Selenium Sulfide\\-1,1\C,
-0.5081539918,2.002633972,-0.0480976548\H,-0.4788477528,0.9253945537,-
0.2174612972\H,-0.154255039,2.5352946825,-0.9374675366\H,-1.5280227726
,2.3062407921,0.1920652733\Se,0.6835479654,2.4567551028,1.4737388123\S
,-0.2570706127,1.2513246311,3.1284314891\\Version=EM64L-G16RevA.03\Sta
te=1-A'\HF=-2839.7494297\RMSD=5.066e-09\RMSF=6.379e-05\Dipole=0.373693
2,0.8698383,-1.9755576\Quadrupole=1.8202618,0.4949051,-2.3151669,-0.47
90741,2.0171909,2.7126258\PG=CS [SG(C1H1S1Se1),X(H2)]\\@
1\1\GINC-ORC381\FOpt\RB3LYP\6-311+G(d,p)\C1H3S1Se1(1-)\MEISTERP\03-Jul
-2017\0\\# opt freq b3lyp/6-311+g(d,p)\\Methyl Selenium Sulfide\\-1,1\
C,-0.5100847414,2.0012834524,-0.0484903872\H,-0.4790901555,0.924332451
3,-0.2182581922\H,-0.1516975615,2.5363796958,-0.9345739392\H,-1.529281
9809,2.3065169337,0.1916652701\Se,0.6822309558,2.4549978315,1.47628992
38\S,-0.2548787173,1.2541333697,3.1245764175\\Version=EM64L-G16RevA.03
\State=1-A'\HF=-2839.7534019\RMSD=5.915e-09\RMSF=5.737e-05\Dipole=0.31
74763,0.803483,-1.8958436\Quadrupole=1.7255022,0.4081364,-2.1336386,-0
.539513,2.0910503,2.701845\PG=CS [SG(C1H1S1Se1),X(H2)]\\@

```
1\1\GINC-ORC378\FOpt\RB3LYP\6-311G(2d,p)\C1H3S1Se1(1-)\MEISTERP\03-Jul
 -2017\0\\# opt freq b3lyp/6-311g(2d,p)\\Methyl Selenium Sulfide\\-1,1\
 C,-0.5090788986,2.0017521959,-0.0474940111\H,-0.4790736744,0.925916589
 9,-0.2179457963\H,-0.1523859446,2.5355279718,-0.9334656705\H,-1.527746
 798,2.3061022698,0.1913848681\Se,0.6800816308,2.4540001604,1.474147479
 8\S,-0.2545985199,1.2543445466,3.1245822128\\Version=EM64L-G16RevA.03\
 State=1-A'\HF=-2839.7502501\RMSD=3.485e-09\RMSF=6.470e-05\Dipole=0.335
 1844,0.8122406,-1.8800056\Quadrupole=1.7455811,0.5000867,-2.2456678,-0
 .4273749,1.86245,2.5500258\PG=CS [SG(C1H1S1Se1),X(H2)]\\@
```

```
1\1\GINC-ORC376\FOpt\RB3LYP\6-311G(df,p)\C1H3S1Se1(1-)\MEISTERP\03-Jul
 -2017\0\\# opt freq b3lyp/6-311g(df,p)\\Methyl Selenium Sulfide\\-1,1\
 C,-0.5067840172,2.0026915609,-0.044782066\H,-0.4790128247,0.9254202344
 ,-0.2151499562\H,-0.1531421748,2.5351640496,-0.934175996\H,-1.52742442
 37,2.305261715,0.1940786268\Se,0.6796401739,2.4532880526,1.4754175947\
 S,-0.256078938,1.2558181217,3.1158208804\\Version=EM64L-G16RevA.03\Sta
 te=1-A'\HF=-2839.7558347\RMSD=4.842e-09\RMSF=6.065e-05\Dipole=0.370818
 6,0.861478,-1.9547329\Quadrupole=1.8194505,0.5335515,-2.3530019,-0.583
 6704,2.0229815,2.7681627\PG=CS [SG(C1H1S1Se1),X(H2)]\\@
```

```
1\1\GINC-ORC370\FOpt\RB3LYP\6-311+G(2df,p)\C1H3S1Se1(1-)\MEISTERP\03-J
 ul-2017\0\\# opt freq b3lyp/6-311+g(2df,p)\\Methyl Selenium Sulfide\\-
 1,1\C,-0.5094024439,2.0006025818,-0.0444466275\H,-0.4796727632,0.92497
 0942,-0.2171474575\H,-0.149150374,2.536317184,-0.9278374665\H,-1.52857
 73864,2.3054613044,0.1922735685\Se,0.6753419783,2.4490898526,1.4785614
 917\S,-0.2513412193,1.2612018694,3.1098055651\\Version=EM64L-G16RevA.0
 3\State=1-A'\HF=-2839.7598474\RMSD=4.415e-09\RMSF=5.223e-05\Dipole=0.2
 822297,0.7463548,-1.793517\Quadrupole=1.6617894,0.4426816,-2.1044709,-
 0.5589363,1.950803,2.5915552\PG=CS [SG(C1H1S1Se1),X(H2)]\\@
```

```
1\1\GINC-ORC368\FOpt\RB3LYP\6-311++G(3df,3pd)\C1H3S1Se1(1-)\MEISTERP\0
 3-Jul-2017\0\\# opt freq b3lyp/6-311++g(3df,3pd)\\Methyl Selenium Sulf
 ide\\-1,1\C,-0.5095594694,2.0004625383,-0.044376714\H,-0.4792714226,0.
 9260996368,-0.2152930361\H,-0.1496706176,2.5354059521,-0.926097788\H,-
 1.5269224664,2.3049401311,0.1936386778\Se,0.6750208463,2.4491826928,1.
 4774257369\S,-0.2523990782,1.2615527831,3.1059121986\\Version=EM64L-G1
 6RevA.03\State=1-A'\HF=-2839.7658015\RMSD=9.406e-09\RMSF=5.229e-05\Dip
 ole=0.3129255,0.7550963,-1.7443514\Quadrupole=1.6253207,0.4980294,-2.1
 233501,-0.6509691,1.86527,2.567175\PG=CS [SG(C1H1S1Se1),X(H2)]\\@
```

CH_3SeS^- B3PW91

```
1\1\GINC-ORC267\FOpt\RB3PW91\6-31G(d)\C1H3S1Se1(1-)\MEISTERP\03-Jul-20
 17\0\\# opt freq b3pw91/6-31g(d)\\Methyl Selenium Sulfide\\-1,1\C,-0.5
 049568789,2.0024831836,-0.0393984719\H,-0.4761741398,0.9233577669,-0.2
 148317548\H,-0.1514700016,2.5357438579,-0.9318470234\H,-1.5283498995,2
 .3081533562,0.1958661005\Se,0.6697742315,2.4452068543,1.4773900969\S,-
 0.2516255161,1.2626987155,3.1040301366\\Version=EM64L-G16RevA.03\State
 =1-A'\HF=-2837.4446054\RMSD=3.786e-09\RMSF=4.407e-05\Dipole=0.2941391,
 0.776452,-1.8644882\Quadrupole=1.7126385,0.4179104,-2.1305489,-0.53004
 1,2.0473454,2.6652686\PG=CS [SG(C1H1S1Se1),X(H2)]\\@
```

```
1\1\GINC-ORC257\FOpt\RB3PW91\6-311G(d)\C1H3S1Se1(1-)\MEISTERP\03-Jul-2
 017\0\\# opt freq b3pw91/6-311g(d)\\Methyl Selenium Sulfide\\-1,1\C,-0
 .5037968611,2.0032066818,-0.0388660965\H,-0.4778455145,0.9254305361,-0
 .2117316429\H,-0.1540996721,2.5338603006,-0.9322330339\H,-1.5261319464
```

,2.305107281,0.1974480833\Se,0.6773029835,2.4523186827,1.4726984281\S,
-0.2582311929,1.257720253,3.1038933447\\Version=EM64L-G16RevA.03\State
=1-A'\HF=-2839.6564458\RMSD=9.950e-09\RMSF=6.473e-05\Dipole=0.3510617,
0.8551379,-1.983971\Quadrupole=1.7590264,0.4236849,-2.1827113,-0.47820
59,2.063014,2.6843546\PG=CS [SG(C1H1S1Se1),X(H2)]\\@

1\1\GINC-ORC247\FOpt\RB3PW91\6-311G(d,p)\C1H3S1Se1(1-)\MEISTERP\03-Jul
-2017\0\\# opt freq b3pw91/6-311g(d,p)\\Methyl Selenium Sulfide\\-1,1\
C,-0.5052705005,2.0024854281,-0.0402095136\H,-0.4770545815,0.924511083
7,-0.2111850482\H,-0.1519709652,2.5350180103,-0.9306830317\H,-1.526580
5114,2.3058191639,0.1984784937\Se,0.6756457249,2.4516339654,1.47076139
88\S,-0.2575713695,1.2581760832,3.1040467874\\Version=EM64L-G16RevA.03
\State=1-A'\HF=-2839.6612217\RMSD=3.317e-09\RMSF=6.605e-05\Dipole=0.35
27791,0.8551904,-1.9797482\Quadrupole=1.767346,0.4384801,-2.2058261,-0
.478781,2.0475548,2.6839455\PG=CS [SG(C1H1S1Se1),X(H2)]\\@

1\1\GINC-ORC245\FOpt\RB3PW91\6-311+G(d,p)\C1H3S1Se1(1-)\MEISTERP\03-Ju
l-2017\0\\# opt freq b3pw91/6-311+g(d,p)\\Methyl Selenium Sulfide\\-1,
1\C,-0.5068843117,2.0013252002,-0.0404319025\H,-0.4775010924,0.9236618
358,-0.2125144275\H,-0.149578913,2.5360624204,-0.9280763351\H,-1.52785
21819,2.3060559298,0.1974712006\Se,0.6741491255,2.4498408028,1.4729734
339\S,-0.2551348331,1.2606975454,3.1017871085\\Version=EM64L-G16RevA.0
3\State=1-A'\HF=-2839.6645811\RMSD=5.009e-09\RMSF=5.972e-05\Dipole=0.3
033635,0.7945107,-1.9017466\Quadrupole=1.6739844,0.358117,-2.0321014,-
0.5432246,2.114443,2.6729672\PG=CS [SG(C1H1S1Se1),X(H2)]\\@

1\1\GINC-ORC226\FOpt\RB3PW91\6-311G(2d,p)\C1H3S1Se1(1-)\MEISTERP\03-Ju
l-2017\0\\# opt freq b3pw91/6-311g(2d,p)\\Methyl Selenium Sulfide\\-1,
1\C,-0.5062948851,2.0015849132,-0.0397975408\H,-0.4771724458,0.9248613
005,-0.2118186391\H,-0.1500209992,2.5353988682,-0.9269715525\H,-1.5264
685421,2.3058668907,0.1977551914\Se,0.6721968719,2.4489282419,1.471048
8959\S,-0.2550422031,1.2610035202,3.10099273\\Version=EM64L-G16RevA.03
\State=1-A'\HF=-2839.66219\RMSD=9.740e-09\RMSF=6.883e-05\Dipole=0.3095
372,0.7921092,-1.8778325\Quadrupole=1.6862905,0.4423748,-2.1286653,-0.
4123478,1.8750198,2.4999714\PG=CS [SG(C1H1S1Se1),X(H2)]\\@

1\1\GINC-ORC221\FOpt\RB3PW91\6-311G(df,p)\C1H3S1Se1(1-)\MEISTERP\03-Ju
l-2017\0\\# opt freq b3pw91/6-311g(df,p)\\Methyl Selenium Sulfide\\-1,
1\C,-0.5038070034,2.0026514838,-0.0370200573\H,-0.4771352202,0.9245615
821,-0.2085817813\H,-0.1510839399,2.534824274,-0.9277573005\H,-1.52585
46181,2.3048081648,0.2007669454\Se,0.6720618214,2.4484796694,1.4722154
09\S,-0.2569832421,1.2623185601,3.0915858739\\Version=EM64L-G16RevA.03
\State=1-A'\HF=-2839.6679778\RMSD=3.592e-09\RMSF=6.636e-05\Dipole=0.34
95316,0.8461127,-1.9574595\Quadrupole=1.7646966,0.4766898,-2.2413864,-
0.5859917,2.0535383,2.7394074\PG=CS [SG(C1H1S1Se1),X(H2)]\\@

1\1\GINC-ORC217\FOpt\RB3PW91\6-311+G(2df,p)\C1H3S1Se1(1-)\MEISTERP\03-
Jul-2017\0\\# opt freq b3pw91/6-311+g(2df,p)\\Methyl Selenium Sulfide\
\-1,1\C,-0.5063014539,2.0006565743,-0.0366841862\H,-0.4779060006,0.924
090099,-0.2112875738\H,-0.1470743173,2.5361079939,-0.921813422\H,-1.52
72535098,2.3051633552,0.1983063249\Se,0.6676267638,2.4442819079,1.4750
071971\S,-0.2518936887,1.2673438036,3.0876807395\\Version=EM64L-G16Rev
A.03\State=1-A'\HF=-2839.6714324\RMSD=7.299e-09\RMSF=5.893e-05\Dipole=
0.2643422,0.7326099,-1.7929981\Quadrupole=1.6034349,0.3887593,-1.99219
42,-0.5547329,1.9658101,2.5489745\PG=CS [SG(C1H1S1Se1),X(H2)]\\@

1\1\GINC-ORC215\FOpt\RB3PW91\6-311++G(3df,3pd)\C1H3S1Se1(1-)\MEISTERP\
03-Jul-2017\0\\# opt freq b3pw91/6-311++g(3df,3pd)\\Methyl Selenium Su
lfide\\-1,1\C,-0.5063036579,2.00063801,-0.0366272373\H,-0.4774723501,0
.925072556,-0.2092841646\H,-0.1476918706,2.5352662439,-0.9205573234\H,
-1.5256818078,2.3046479932,0.1998655161\Se,0.6674602463,2.4444463931,1
.4740259795\S,-0.2531127637,1.2675725382,3.0837863151\\Version=EM64L-G
16RevA.03\State=1-A'\HF=-2839.6773214\RMSD=8.632e-09\RMSF=5.844e-05\Di
pole=0.2956077,0.7423401,-1.7457066\Quadrupole=1.5736881,0.4489596,-2.
0226478,-0.6410978,1.8764293,2.52533\PG=CS [SG(C1H1S1Se1),X(H2)]\\@

CH_3SeS^- wB97XD

1\1\GINC-ORC361\FOpt\RwB97XD\6-31G(d)\C1H3S1Se1(1-)\MEISTERP\03-Jul-20
17\0\\# opt freq wb97xd/6-31g(d)\\Methyl Selenium Sulfide\\-1,1\C,-0.5
020495347,2.003141761,-0.034170682\H,-0.4736909651,0.9242901853,-0.206
6587638\H,-0.1546107057,2.5327970978,-0.9299573429\H,-1.5245256576,2.3
073207616,0.2035156301\Se,0.6710006802,2.4478435429,1.4716417463\S,-0.
2589260242,1.262250386,3.0868384885\\Version=EM64L-G16RevA.03\State=1-
A'\HF=-2837.511086\RMSD=1.703e-09\RMSF=4.122e-05\Dipole=0.306019,0.796
0533,-1.9001447\Quadrupole=1.6987827,0.3834739,-2.0822566,-0.58899,2.1
368239,2.7274746\PG=CS [SG(C1H1S1Se1),X(H2)]\\@

1\1\GINC-ORC392\FOpt\RwB97XD\6-311G(d)\C1H3S1Se1(1-)\MEISTERP\03-Jul-2
017\0\\# opt freq wb97xd/6-311g(d)\\Methyl Selenium Sulfide\\-1,1\C,-0
.5005898785,2.004021017,-0.0333958423\H,-0.4753229404,0.9262390888,-0.
203322823\H,-0.1576814097,2.5307292899,-0.9308519888\H,-1.5223448306,2
.3042515379,0.2053633122\Se,0.6790390498,2.4551993925,1.4674328849\S,-
0.2659021957,1.2572034075,3.0859835412\\Version=EM64L-G16RevA.03\State
=1-A'\HF=-2839.7294032\RMSD=5.503e-09\RMSF=5.519e-05\Dipole=0.3684402,
0.8828535,-2.0329004\Quadrupole=1.7500592,0.3868062,-2.1368654,-0.5344
704,2.1619586,2.7537847\PG=CS [SG(C1H1S1Se1),X(H2)]\\@

1\1\GINC-ORC391\FOpt\RwB97XD\6-311G(d,p)\C1H3S1Se1(1-)\MEISTERP\03-Jul
-2017\0\\# opt freq wb97xd/6-311g(d,p)\\Methyl Selenium Sulfide\\-1,1\
C,-0.5017585321,2.0034548946,-0.0344809835\H,-0.4745958526,0.925472926
6,-0.2025439549\H,-0.1559929863,2.5316461148,-0.9296177404\H,-1.522610
2293,2.3047916127,0.2065295794\Se,0.6777717693,2.4547455221,1.46571657
66\S,-0.2656163706,1.2575326636,3.0856056132\\Version=EM64L-G16RevA.03
\State=1-A'\HF=-2839.7339873\RMSD=7.052e-09\RMSF=5.400e-05\Dipole=0.37
11766,0.8830618,-2.0265922\Quadrupole=1.7568314,0.4008574,-2.1576888,-
0.5352576,2.145859,2.7519074\PG=CS [SG(C1H1S1Se1),X(H2)]\\@

1\1\GINC-ORC390\FOpt\RwB97XD\6-311+G(d,p)\C1H3S1Se1(1-)\MEISTERP\03-Ju
l-2017\0\\# opt freq wb97xd/6-311+g(d,p)\\Methyl Selenium Sulfide\\-1,
1\C,-0.5030696612,2.0024694977,-0.0345174215\H,-0.4750122282,0.9247735
442,-0.203815554\H,-0.1538073019,2.532580328,-0.9271681786\H,-1.523720
2637,2.3050051725,0.2055287376\Se,0.676136706,2.4530028373,1.467403676
5\S,-0.2633294582,1.2598123539,3.0837778188\\Version=EM64L-G16RevA.03\
State=1-A'\HF=-2839.7373118\RMSD=5.936e-09\RMSF=4.904e-05\Dipole=0.321
9767,0.8297303,-1.9728147\Quadrupole=1.6353205,0.2768563,-1.9121767,-0
.5932253,2.2496032,2.7324993\PG=CS [SG(C1H1S1Se1),X(H2)]\\@

1\1\GINC-ORC389\FOpt\RwB97XD\6-311G(2d,p)\C1H3S1Se1(1-)\MEISTERP\03-Ju
l-2017\0\\# opt freq wb97xd/6-311g(2d,p)\\Methyl Selenium Sulfide\\-1,
1\C,-0.5026404541,2.0026356004,-0.0339778935\H,-0.4750392299,0.9258959
293,-0.2037801739\H,-0.1539371907,2.5321142173,-0.9259293065\H,-1.5226
947521,2.3047423177,0.2051532881\Se,0.6739881312,2.4516194221,1.466563

```
8107\S,-0.2624787098,1.2606362475,3.0831793562\\Version=EM64L-G16RevA.
03\State=1-A'\HF=-2839.7351108\RMSD=5.386e-09\RMSF=5.542e-05\Dipole=0.
3246968,0.8173253,-1.9240187\Quadrupole=1.6729717,0.4034327,-2.0764045
,-0.4567329,1.9626547,2.5542875\PG=CS [SG(C1H1S1Se1),X(H2)]\\@
```

```
1\1\GINC-ORC330\FOpt\RwB97XD\6-311G(df,p)\C1H3S1Se1(1-)\MEISTERP\03-Ju
l-2017\0\\# opt freq wb97xd/6-311g(df,p)\\Methyl Selenium Sulfide\\-1,
1\C,-0.5006925504,2.003532937,-0.0320131661\H,-0.4749413722,0.92549120
04,-0.2009974184\H,-0.1547859889,2.5317561624,-0.9268965671\H,-1.52228
09579,2.3039217766,0.2078127234\Se,0.6740924704,2.4513721197,1.4676649
767\S,-0.2641938067,1.2615695383,3.075638532\\Version=EM64L-G16RevA.03
\State=1-A'\HF=-2839.7410536\RMSD=8.868e-09\RMSF=5.396e-05\Dipole=0.36
58612,0.8719837,-2.0028565\Quadrupole=1.7558729,0.4420324,-2.1979053,-
0.6409493,2.1475389,2.8058872\PG=CS [SG(C1H1S1Se1),X(H2)]\\@
```

```
1\1\GINC-ORC385\FOpt\RwB97XD\6-311+G(2df,p)\C1H3S1Se1(1-)\MEISTERP\03-
Jul-2017\0\\# opt freq wb97xd/6-311+g(2df,p)\\Methyl Selenium Sulfide\
\-1,1\C,-0.5027000778,2.0017979046,-0.0313060925\H,-0.4759636969,0.925
2828325,-0.2041755171\H,-0.1509610729,2.5329460575,-0.9211095334\H,-1.
523644707,2.3041627663,0.2047678936\Se,0.6692108448,2.4468719302,1.470
3324229\S,-0.2587434928,1.2665822432,3.0726999155\\Version=EM64L-G16Re
vA.03\State=1-A'\HF=-2839.744579\RMSD=8.832e-09\RMSF=4.788e-05\Dipole=
0.2778634,0.7631738,-1.8614135\Quadrupole=1.5641805,0.3109869,-1.87516
74,-0.5990317,2.0892838,2.5994387\PG=CS [SG(C1H1S1Se1),X(H2)]\\@
```

```
1\1\GINC-ORC382\FOpt\RwB97XD\6-311++G(3df,3pd)\C1H3S1Se1(1-)\MEISTERP\
03-Jul-2017\0\\# opt freq wb97xd/6-311++g(3df,3pd)\\Methyl Selenium Su
lfide\\-1,1\C,-0.5028519118,2.0017237193,-0.0314449407\H,-0.4754031336
,0.9265376421,-0.2020220879\H,-0.1518685439,2.5318856478,-0.919858902\
H,-1.5217449546,2.3036550346,0.2063985945\Se,0.6692561309,2.4472296634
,1.4692422333\S,-0.2601897897,1.2666120271,3.0688941902\\Version=EM64L
-G16RevA.03\State=1-A'\HF=-2839.7506678\RMSD=8.503e-09\RMSF=4.640e-05\
Dipole=0.2988964,0.7663292,-1.8181679\Quadrupole=1.5328234,0.3597661,-
1.8925896,-0.6717096,2.0086221,2.5717249\PG=CS [SG(C1H1S1Se1),X(H2)]\\
@
```

CH_3SeS^- M062X

```
1\1\GINC-ORC366\FOpt\RM062X\6-31G(d)\C1H3S1Se1(1-)\MEISTERP\03-Jul-201
7\0\\# opt freq m062x/6-31g(d)\\Methyl Selenium Sulfide\\-1,1\C,-0.499
7798575,2.0041992091,-0.0319214619\H,-0.468787032,0.9251322849,-0.1936
157372\H,-0.1610884771,2.5285380818,-0.9321922891\H,-1.518723499,2.306
9806835,0.2162080505\Se,0.677176425,2.4539568512,1.4668506006\S,-0.271
599764,1.2588366235,3.0658799212\\Version=EM64L-G16RevA.03\State=1-A'\
HF=-2837.5079458\RMSD=5.120e-09\RMSF=5.343e-05\Dipole=0.2934827,0.7656
152,-1.8296301\Quadrupole=1.6009407,0.3470709,-1.9480116,-0.632087,2.0
98914,2.6510496\PG=CS [SG(C1H1S1Se1),X(H2)]\\@
```

```
1\1\GINC-ORC364\FOpt\RM062X\6-311G(d)\C1H3S1Se1(1-)\MEISTERP\03-Jul-20
17\0\\# opt freq m062x/6-311g(d)\\Methyl Selenium Sulfide\\-1,1\C,-0.4
988701626,2.0050207797,-0.0323610758\H,-0.4699647556,0.9270356623,-0.1
889513732\H,-0.1649796748,2.5260948475,-0.9339231083\H,-1.5160853999,2
.3038619585,0.2193829768\Se,0.6873119131,2.4627577569,1.463141936\S,-0
.280214126,1.2528727305,3.0639197209\\Version=EM64L-G16RevA.03\State=1
-A'\HF=-2839.6846935\RMSD=8.532e-09\RMSF=6.714e-05\Dipole=0.3585068,0.
8579729,-1.9744564\Quadrupole=1.6461291,0.3321546,-1.9782837,-0.575547
2,2.1480391,2.6829987\PG=CS [SG(C1H1S1Se1),X(H2)]\\@
```

1\1\GINC-ORC363\FOpt\RM062X\6-311G(d,p)\C1H3S1Se1(1-)\MEISTERP\03-Jul-
2017\0\\# opt freq m062x/6-311g(d,p)\\Methyl Selenium Sulfide\\-1,1\C,
-0.5001051071,2.0044633743,-0.0336454412\H,-0.469001248,0.9262317811,-
0.1876294813\H,-0.1634195292,2.527010846,-0.9330147114\H,-1.5161657582
,2.304431936,0.221112323\Se,0.6862295281,2.4624752228,1.4613216019\S,-
0.2803400884,1.253030574,3.0630647967\\Version=EM64L-G16RevA.03\State=
1-A'\HF=-2839.6881992\RMSD=6.421e-09\RMSF=6.635e-05\Dipole=0.3598812,0
.8582318,-1.9718081\Quadrupole=1.6505218,0.3433755,-1.9938974,-0.57881
95,2.1357464,2.6822925\PG=CS [SG(C1H1S1Se1),X(H2)]\\@

1\1\GINC-ORC362\FOpt\RM062X\6-311+G(d,p)\C1H3S1Se1(1-)\MEISTERP\03-Jul
-2017\0\\# opt freq m062x/6-311+g(d,p)\\Methyl Selenium Sulfide\\-1,1\
C,-0.5016458186,2.0033543446,-0.0338531846\H,-0.4697166369,0.925459576
4,-0.1896398891\H,-0.1607193449,2.5282691468,-0.9303398031\H,-1.517633
8867,2.3046504314,0.2193957335\Se,0.6840507067,2.4601157876,1.46369520
2\S,-0.2771372206,1.2557944488,3.0619510296\\Version=EM64L-G16RevA.03\
State=1-A'\HF=-2839.69144\RMSD=5.225e-09\RMSF=6.007e-05\Dipole=0.31308
63,0.80038,-1.8966276\Quadrupole=1.5240414,0.2367956,-1.760837,-0.6584
559,2.2162641,2.6586025\PG=CS [SG(C1H1S1Se1),X(H2)]\\@

1\1\GINC-ORC361\FOpt\RM062X\6-311G(2d,p)\C1H3S1Se1(1-)\MEISTERP\03-Jul
-2017\0\\# opt freq m062x/6-311g(2d,p)\\Methyl Selenium Sulfide\\-1,1\
C,-0.5012957017,2.0035922097,-0.0337582489\H,-0.4692513962,0.926701342
3,-0.1891533609\H,-0.1611447204,2.5276770493,-0.9294331409\H,-1.516226
3925,2.3046520731,0.21951447\Se,0.6819875038,2.4592200754,1.4614296099
\S,-0.2768714962,1.2558009845,3.0626097574\\Version=EM64L-G16RevA.03\S
tate=1-A'\HF=-2839.6896452\RMSD=4.502e-09\RMSF=6.580e-05\Dipole=0.3134
611,0.7933642,-1.8720115\Quadrupole=1.5777697,0.3600799,-1.9378497,-0.
4921963,1.9357085,2.4814487\PG=CS [SG(C1H1S1Se1),X(H2)]\\@

1\1\GINC-ORC392\FOpt\RM062X\6-311G(df,p)\C1H3S1Se1(1-)\MEISTERP\03-Jul
-2017\0\\# opt freq m062x/6-311g(df,p)\\Methyl Selenium Sulfide\\-1,1\
C,-0.4989202891,2.0046870745,-0.0313643049\H,-0.4697760261,0.926263329
2,-0.1870308814\H,-0.1618822511,2.5274322328,-0.930497162\H,-1.5162125
521,2.3035053654,0.2214267673\Se,0.6818417425,2.4583516334,1.463984403
2\S,-0.2778528248,1.2574040981,3.0546902741\\Version=EM64L-G16RevA.03\
State=1-A'\HF=-2839.6952284\RMSD=6.164e-09\RMSF=6.481e-05\Dipole=0.355
7908,0.8485727,-1.9497187\Quadrupole=1.6568144,0.3993807,-2.0561951,-0
.7019212,2.1331448,2.7490097\PG=CS [SG(C1H1S1Se1),X(H2)]\\@

1\1\GINC-ORC391\FOpt\RM062X\6-311+G(2df,p)\C1H3S1Se1(1-)\MEISTERP\03-J
ul-2017\0\\# opt freq m062x/6-311+g(2df,p)\\Methyl Selenium Sulfide\\-
1,1\C,-0.5015458969,2.0027252744,-0.0314760875\H,-0.4708088375,0.92594
69693,-0.1908898475\H,-0.1574403736,2.5290760349,-0.9246600094\H,-1.51
78623312,2.3040010126,0.2178086235\Se,0.6763147468,2.4532553374,1.4670
084205\S,-0.2714595105,1.2626391058,3.0534179878\\Version=EM64L-G16Rev
A.03\State=1-A'\HF=-2839.6990634\RMSD=3.652e-09\RMSF=5.559e-05\Dipole=
0.2736884,0.7380661,-1.7874512\Quadrupole=1.4703949,0.2930587,-1.76345
36,-0.6646533,2.0396192,2.5321205\PG=CS [SG(C1H1S1Se1),X(H2)]\\@

1\1\GINC-ORC390\FOpt\RM062X\6-311++G(3df,3pd)\C1H3S1Se1(1-)\MEISTERP\0
3-Jul-2017\0\\# opt freq m062x/6-311++g(3df,3pd)\\Methyl Selenium Sulf
ide\\-1,1\C,-0.5014338548,2.0026998405,-0.0311032861\H,-0.4703135503,0
.9273862706,-0.1885109765\H,-0.1587604811,2.5276857275,-0.9233541677\H
,-1.5157532277,2.3033163287,0.2195575703\Se,0.6767594745,2.4538532992,
1.4661315638\S,-0.2733005625,1.2627022679,3.0484883861\\Version=EM64L-

G16RevA.03\State=1-A'\HF=-2839.7052486\RMSD=4.884e-09\RMSF=5.372e-05\D
ipole=0.3019332,0.7452723,-1.7393882\Quadrupole=1.4368601,0.3369725,-1
.7738326,-0.7291866,1.9588035,2.4983936\PG=CS [SG(C1H1S1Se1),X(H2)]\\@

CH_3SeS^- M08HX

1\1\GINC-ORC387\FOpt\RM08HX\6-31G(d)\C1H3S1Se1(1-)\MEISTERP\03-Jul-201
7\0\\# opt freq m08hx/6-31g(d)\\Methyl Selenium Sulfide\\-1,1\C,-0.496
6570894,2.0054968193,-0.0282964712\H,-0.4669804095,0.9210836587,-0.188
3970813\H,-0.1632230892,2.5283211069,-0.9369293996\H,-1.5204516548,2.3
0758427,0.2228064434\Se,0.6840202654,2.4599126903,1.4643020195\S,-0.27
95102282,1.2552451903,3.0577235655\\Version=EM64L-G16RevA.03\State=1-A
'\HF=-2837.538204\RMSD=2.512e-09\RMSF=7.282e-05\Dipole=0.2887032,0.759
4197,-1.8209849\Quadrupole=1.5557768,0.3077472,-1.863524,-0.641308,2.1
154723,2.6268615\PG=CS [SG(C1H1S1Se1),X(H2)]\\@

1\1\GINC-ORC377\FOpt\RM08HX\6-311G(d)\C1H3S1Se1(1-)\MEISTERP\03-Jul-20
17\0\\# opt freq m08hx/6-311g(d)\\Methyl Selenium Sulfide\\-1,1\C,-0.4
959057093,2.0063159556,-0.0291334668\H,-0.4678638564,0.9229075061,-0.1
826397481\H,-0.166925918,2.5260535437,-0.9387699624\H,-1.5175000921,2.
3043607627,0.2270668498\Se,0.6935856604,2.4680775014,1.4612776122\S,-0
.2881922885,1.2499284649,3.0534078005\\Version=EM64L-G16RevA.03\State=
1-A'\HF=-2839.6983371\RMSD=7.022e-09\RMSF=9.634e-05\Dipole=0.3543655,0
.8460575,-1.9448896\Quadrupole=1.5896042,0.2855499,-1.875154,-0.582915
2,2.1607145,2.6466884\PG=CS [SG(C1H1S1Se1),X(H2)]\\@

1\1\GINC-ORC370\FOpt\RM08HX\6-311G(d,p)\C1H3S1Se1(1-)\MEISTERP\03-Jul-
2017\0\\# opt freq m08hx/6-311g(d,p)\\Methyl Selenium Sulfide\\-1,1\C,
-0.49721909,2.0056988525,-0.0304174905\H,-0.4670100729,0.9222945868,-0
.1815222021\H,-0.1653452579,2.5268956725,-0.9375599343\H,-1.5174951413
,2.3048650138,0.2285157223\Se,0.6923920416,2.4677051686,1.45947509\S,-
0.2881246877,1.2501844396,3.05271789\\Version=EM64L-G16RevA.03\State=1
-A'\HF=-2839.7022458\RMSD=6.164e-09\RMSF=9.503e-05\Dipole=0.3566711,0.
846291,-1.9397698\Quadrupole=1.5947733,0.2965571,-1.8913304,-0.5843193
,2.1485065,2.6457045\PG=CS [SG(C1H1S1Se1),X(H2)]\\@

1\1\GINC-ORC369\FOpt\RM08HX\6-311+G(d,p)\C1H3S1Se1(1-)\MEISTERP\03-Jul
-2017\0\\# opt freq m08hx/6-311+g(d,p)\\Methyl Selenium Sulfide\\-1,1\
C,-0.4992410832,2.0043299239,-0.03098191\H,-0.468219484,0.9214019553,-
0.1845915583\H,-0.1617327979,2.5287119571,-0.9344292628\H,-1.519511245
8,2.3050340923,0.2257612443\Se,0.6896501828,2.4647785548,1.4623186511\
S,-0.2837477763,1.253387251,3.0531319194\\Version=EM64L-G16RevA.03\Sta
te=1-A'\HF=-2839.7058719\RMSD=7.584e-09\RMSF=8.537e-05\Dipole=0.308846
4,0.7784839,-1.8336605\Quadrupole=1.4599374,0.2132394,-1.6731768,-0.67
68358,2.1834063,2.5996053\PG=CS [SG(C1H1S1Se1),X(H2)]\\@

1\1\GINC-ORC367\FOpt\RM08HX\6-311G(2d,p)\C1H3S1Se1(1-)\MEISTERP\03-Jul
-2017\0\\# opt freq m08hx/6-311g(2d,p)\\Methyl Selenium Sulfide\\-1,1\
C,-0.498027634,2.0050279819,-0.030226868\H,-0.4672436787,0.9230027191,
-0.1830173342\H,-0.16351558,2.5272942793,-0.9342164694\H,-1.517325218,
2.3050420512,0.2268630798\Se,0.6881374935,2.4644848036,1.4594337331\S,
-0.2848275882,1.2527918973,3.0523729459\\Version=EM64L-G16RevA.03\Stat
e=1-A'\HF=-2839.7038892\RMSD=6.183e-09\RMSF=9.563e-05\Dipole=0.3145417
,0.7852912,-1.8420223\Quadrupole=1.5329794,0.3236745,-1.8566539,-0.497
5756,1.9444236,2.4517968\PG=CS [SG(C1H1S1Se1),X(H2)]\\@

1\1\GINC-ORC365\FOpt\RM08HX\6-311G(df,p)\C1H3S1Se1(1-)\MEISTERP\03-Jul
-2017\0\\# opt freq m08hx/6-311g(df,p)\\Methyl Selenium Sulfide\\-1,1\
C,-0.4961845712,2.0059007103,-0.0284477608\H,-0.4682212807,0.922377906
9,-0.182131626\H,-0.1632573074,2.5276923944,-0.9348971642\H,-1.5179554
581,2.3039600669,0.2276132016\Se,0.6872861398,2.4629868069,1.462303593
5\S,-0.2844697263,1.2547258467,3.0467688482\\Version=EM64L-G16RevA.03\
State=1-A'\HF=-2839.7094276\RMSD=5.099e-09\RMSF=9.418e-05\Dipole=0.355
1319,0.8393949,-1.9204609\Quadrupole=1.6118097,0.3668761,-1.9786858,-0
.7096922,2.1360683,2.717591\PG=CS [SG(C1H1S1Se1),X(H2)]\\@

1\1\GINC-ORC363\FOpt\RM08HX\6-311+G(2df,p)\C1H3S1Se1(1-)\MEISTERP\03-J
ul-2017\0\\# opt freq m08hx/6-311+g(2df,p)\\Methyl Selenium Sulfide\\-
1,1\C,-0.4987481437,2.003991981,-0.0285795579\H,-0.4692687348,0.922183
3514,-0.1859172577\H,-0.1589454101,2.5293098824,-0.929304285\H,-1.5194
787295,2.3043917469,0.2240132967\Se,0.6817150229,2.4577565885,1.465666
1368\S,-0.2780762064,1.2600101843,3.0453307574\\Version=EM64L-G16RevA.
03\State=1-A'\HF=-2839.7138776\RMSD=7.859e-09\RMSF=7.689e-05\Dipole=0.
2785093,0.7233467,-1.7254693\Quadrupole=1.4217517,0.2880397,-1.7097914
,-0.6810982,1.9925199,2.4778641\PG=CS [SG(C1H1S1Se1),X(H2)]\\@

1\1\GINC-ORC362\FOpt\RM08HX\6-311++G(3df,3pd)\C1H3S1Se1(1-)\MEISTERP\0
3-Jul-2017\0\\# opt freq m08hx/6-311++g(3df,3pd)\\Methyl Selenium Sulf
ide\\-1,1\C,-0.4992036088,2.0038104121,-0.0291342081\H,-0.4679061112,0
.9239640328,-0.1815713749\H,-0.1605732639,2.5275996102,-0.927708004\H,
-1.5162597902,2.3037292834,0.2276346\Se,0.6822740976,2.4586984471,1.46
39226727\S,-0.2811335304,1.2598419486,3.0380653918\\Version=EM64L-G16R
evA.03\State=1-A'\HF=-2839.720221\RMSD=7.595e-09\RMSF=7.243e-05\Dipole
=0.3121047,0.7324359,-1.6700477\Quadrupole=1.3627953,0.3241719,-1.6869
672,-0.7540103,1.8965802,2.4202806\PG=CS [SG(C1H1S1Se1),X(H2)]\\@

CH$_3$SeS$^-$ QCISD

1\1\GINC-ORC194\FOpt\RQCISD-FC\6-311+G(2df,p)\C1H3S1Se1(1-)\MEISTERP\2
1-Aug-2017\0\\# opt freq=noraman qcisd/6-311+g(2df,p) guess=read\\Meth
yl Selenium Sulfide\\-1,1\C,1.455162,1.133864,0.000003\H,1.220214,1.72
2012,-0.890083\H,2.516932,0.861343,-0.000502\H,1.221082,1.721793,0.890
457\Se,0.374506,-0.502544,0.000003\S,-1.6514,0.373636,0.000001\\Versio
n=EM64L-G09RevE.01\State=1-A\HF=-2837.0020275\MP2=-2837.5048685\MP3=-2
837.5427437\MP4D=-2837.5560926\MP4DQ=-2837.5407661\MP4SDQ=-2837.544539
\QCISD=-2837.54446\RMSD=6.389e-09\RMSF=6.746e-05\Dipole=1.956398,-0.04
46316,-0.0000327\PG=C01 [X(C1H3S1Se1)]\\@

CH$_3$SSeH B3LYP

1\1\GINC-ORC238\FOpt\RB3LYP\6-31G(d)\C1H4S1Se1\MEISTERP\20-Jul-2017\0\
\# opt=readfc freq=noraman b3lyp/6-31g(d) guess=read\\Methyl Sulfur Se
lenide\\0,1\C,-1.9050220934,-0.7676981271,0.0405266104\H,-1.6854801407
,-1.5626735917,-0.674899285\H,-2.9858446516,-0.5947841387,0.0733783786
\H,-1.5530188954,-1.0502570295,1.0352027118\S,-1.1529110896,0.81926687
97,-0.498289318\Se,0.9881513096,0.2724994876,-0.6994495874\H,1.3751465
611,0.5182785197,0.7241624895\\Version=EM64L-G09RevE.01\State=1-A\HF=-
2838.0873199\RMSD=4.187e-09\RMSF=2.150e-05\Dipole=-0.1492896,-0.527219
2,0.4160757\Quadrupole=1.897119,-1.6524297,-0.2446893,1.5811471,0.0953
02,-0.0299285\PG=C01 [X(C1H4S1Se1)]\\@

1\1\GINC-ORC227\FOpt\RB3LYP\6-311G(d)\C1H4S1Se1\ROOT\20-Jul-2017\0\\#
opt=readfc freq=noraman b3lyp/6-311g(d) guess=read\\Methyl Sulfur Sele
nide\\0,1\C,-1.9111320047,-0.7744248952,0.0389469586\H,-1.705655731,-1

.5677369497,-0.6777399533\H,-2.9875821132,-0.590113765,0.0713525382\H,
-1.5681288972,-1.0639101002,1.031349422\S,-1.1532936107,0.8082609984,-
0.4916277403\Se,1.0098570806,0.2821584871,-0.6931317736\H,1.3969562763
,0.5403982246,0.7214825484\\Version=EM64L-G09RevE.01\State=1-A\HF=-284
0.2930701\RMSD=4.242e-09\RMSF=9.741e-06\Dipole=-0.134968,-0.5339749,0.
415854\Quadrupole=2.0726955,-1.7893045,-0.283391,1.6220624,0.0820175,0
.0446321\PG=C01 [X(C1H4S1Se1)]\\@

1\1\GINC-ORC222\FOpt\RB3LYP\6-311G(d,p)\C1H4S1Se1\ROOT\20-Jul-2017\0\\
opt=readfc freq=noraman b3lyp/6-311g(d,p) guess=read\\Methyl Sulfur
Selenide\\0,1\C,-1.9124444212,-0.7768620368,0.0391125015\H,-1.70736197
29,-1.566217491,-0.6822362326\H,-2.9881350554,-0.5875337899,0.07213105
64\H,-1.5672789952,-1.0678442801,1.0304030269\S,-1.1532502397,0.807188
9623,-0.4873968826\Se,1.0123237632,0.2829647058,-0.6879187676\H,1.3971
679212,0.5429359296,0.716537298\\Version=EM64L-G09RevE.01\State=1-A\HF
=-2840.3005834\RMSD=3.254e-09\RMSF=1.081e-05\Dipole=-0.1310319,-0.5295
602,0.4190088\Quadrupole=2.0914485,-1.7602347,-0.3312138,1.6359751,0.0
935347,0.0418511\PG=C01 [X(C1H4S1Se1)]\\@

1\1\GINC-ORC220\FOpt\RB3LYP\6-311+G(d,p)\C1H4S1Se1\ROOT\20-Jul-2017\0\
\# opt=readfc freq=noraman b3lyp/6-311+g(d,p) guess=read\\Methyl Sulfu
r Selenide\\0,1\C,-1.9130112677,-0.7773665019,0.0385058552\H,-1.710692
6013,-1.5667950298,-0.6839682399\H,-2.988479599,-0.5865052034,0.074036
1563\H,-1.5663589402,-1.0704794589,1.0289013229\S,-1.1523571416,0.8061
102231,-0.4871091465\Se,1.0127575228,0.2820828395,-0.6864832563\H,1.39
91630268,0.5475851315,0.7167493083\\Version=EM64L-G09RevE.01\State=1-A
\HF=-2840.3019875\RMSD=3.553e-09\RMSF=1.067e-05\Dipole=-0.1584793,-0.5
119658,0.4122061\Quadrupole=2.0599071,-1.7895292,-0.270378,1.6122268,0
.1548481,0.0639582\PG=C01 [X(C1H4S1Se1)]\\@

1\1\GINC-ORC219\FOpt\RB3LYP\6-311G(2d,p)\C1H4S1Se1\MEISTERP\20-Jul-201
7\0\\# opt=readfc freq=noraman b3lyp/6-311g(2d,p) guess=read\\Methyl S
ulfur Selenide\\0,1\C,-1.911726192,-0.7781233676,0.0385844285\H,-1.712
3478981,-1.5648062779,-0.6856041676\H,-2.985579745,-0.5834891256,0.077
1751878\H,-1.5608559412,-1.0687485546,1.0266678254\S,-1.1482485626,0.8
011128902,-0.488849174\Se,1.0079948105,0.2808351908,-0.6847746672\H,1.
3917845284,0.5478512447,0.7174325671\\Version=EM64L-G09RevE.01\State=1
-A\HF=-2840.3037007\RMSD=2.421e-09\RMSF=9.140e-06\Dipole=-0.1699856,-0
.5011017,0.3781382\Quadrupole=2.1433896,-1.7203702,-0.4230194,1.518660
9,0.0077543,0.0108261\PG=C01 [X(C1H4S1Se1)]\\@

1\1\GINC-ORC207\FOpt\RB3LYP\6-311G(df,p)\C1H4S1Se1\ROOT\20-Jul-2017\0\
\# opt=readfc freq=noraman b3lyp/6-311g(df,p) guess=read\\Methyl Sulfu
r Selenide\\0,1\C,-1.9104126443,-0.7757026712,0.0390519363\H,-1.704997
6081,-1.5669203011,-0.6801939856\H,-2.9858217823,-0.5860106019,0.07041
40759\H,-1.5671621327,-1.0672452559,1.0308869173\S,-1.1476753349,0.804
6735286,-0.4876717364\Se,1.0030394569,0.2862903924,-0.6887094826\H,1.3
940510454,0.539546909,0.716854275\\Version=EM64L-G09RevE.01\State=1-A\
HF=-2840.3066803\RMSD=9.753e-09\RMSF=1.031e-05\Dipole=-0.1374331,-0.52
56984,0.4181007\Quadrupole=1.9365506,-1.6656675,-0.2708831,1.6694697,0
.097466,0.0255002\PG=C01 [X(C1H4S1Se1)]\\@

1\1\GINC-ORC193\FOpt\RB3LYP\6-311+G(2df,p)\C1H4S1Se1\MEISTERP\20-Jul-2
017\0\\# opt=readfc freq=noraman b3lyp/6-311+g(2df,p) guess=read\\Meth
yl Sulfur Selenide\\0,1\C,-1.909200994,-0.7761504586,0.0378520267\H,-1
.7108797073,-1.5653870938,-0.6844617388\H,-2.9831807607,-0.5818765932,
0.0759759301\H,-1.5605354437,-1.0691938677,1.0264228628\S,-1.145125526

9,0.7970476219,-0.4876138068\Se,0.9981524115,0.2828624512,-0.684322820
1\H,1.391791021,0.54732994,0.716779546\\Version=EM64L-G09RevE.01\State
=1-A\HF=-2840.3110309\RMSD=3.500e-09\RMSF=1.396e-05\Dipole=-0.1953693,
-0.480288,0.3710429\Quadrupole=1.9823594,-1.6880172,-0.2943421,1.51300
29,0.0808627,0.0329059\PG=C01 [X(C1H4S1Se1)]\\@

1\1\GINC-ORC187\FOpt\RB3LYP\6-311++G(3df,3pd)\C1H4S1Se1\MEISTERP\20-Ju
l-2017\0\\# opt=readfc freq=noraman b3lyp/6-311++g(3df,3pd) guess=read
\\Methyl Sulfur Selenide\\0,1\C,-1.9082189035,-0.7757973293,0.03746902
09\H,-1.7127849017,-1.5653923332,-0.6827718723\H,-2.9802185391,-0.5799
605928,0.0761490828\H,-1.5605666846,-1.0677640733,1.0248925554\S,-1.14
48042661,0.7925265723,-0.4895420205\Se,0.9987583472,0.2843760813,-0.68
36348936\H,1.3888559478,0.546643675,0.7180701273\\Version=EM64L-G09Rev
E.01\State=1-A\HF=-2840.3174359\RMSD=6.883e-09\RMSF=1.001e-05\Dipole=-
0.190118,-0.4754349,0.3624519\Quadrupole=1.9518102,-1.6208114,-0.33099
88,1.5200063,0.032464,0.0036607\PG=C01 [X(C1H4S1Se1)]\\@

CH_3SSeH B3PW91

1\1\GINC-ORC176\FOpt\RB3PW91\6-31G(d)\C1H4S1Se1\MEISTERP\20-Jul-2017\0
\\# opt=readfc freq=noraman b3pw91/6-31g(d) guess=read\\Methyl Sulfur
Selenide\\0,1\C,-1.8971832594,-0.7624937744,0.0398505245\H,-1.68109093
19,-1.5589073892,-0.6756667006\H,-2.9782674603,-0.5898955563,0.0741788
822\H,-1.5465469736,-1.0479257964,1.0345972788\S,-1.1482345455,0.81263
66561,-0.4952297271\Se,0.9718806846,0.2668656406,-0.6980323122\H,1.360
463486,0.5143522194,0.7209340544\\Version=EM64L-G09RevE.01\State=1-A\H
F=-2838.0037355\RMSD=3.405e-09\RMSF=1.224e-05\Dipole=-0.159632,-0.5359
497,0.4385871\Quadrupole=1.9131635,-1.6818264,-0.2313371,1.6177009,0.1
340215,-0.0208019\PG=C01 [X(C1H4S1Se1)]\\@

1\1\GINC-ORC173\FOpt\RB3PW91\6-311G(d)\C1H4S1Se1\MEISTERP\20-Jul-2017\
0\\# opt=readfc freq=noraman b3pw91/6-311g(d) guess=read\\Methyl Sulfu
r Selenide\\0,1\C,-1.9025904093,-0.7692755291,0.0381010786\H,-1.701380
6033,-1.5643788674,-0.6791162678\H,-2.9796369465,-0.584491327,0.072224
9394\H,-1.5609232581,-1.0619414876,1.0309225788\S,-1.1482309192,0.8013
165847,-0.4888377121\Se,0.9911731345,0.2759367393,-0.6914089534\H,1.38
26100018,0.5374658871,0.7187463365\\Version=EM64L-G09RevE.01\State=1-A
\HF=-2840.2072815\RMSD=3.393e-09\RMSF=1.351e-05\Dipole=-0.1451794,-0.5
362511,0.4352306\Quadrupole=2.0621413,-1.7973869,-0.2647544,1.6460645,
0.1285397,0.0566653\PG=C01 [X(C1H4S1Se1)]\\@

1\1\GINC-ORC155\FOpt\RB3PW91\6-311G(d,p)\C1H4S1Se1\MEISTERP\20-Jul-201
7\0\\# opt=readfc freq=noraman b3pw91/6-311g(d,p) guess=read\\Methyl S
ulfur Selenide\\0,1\C,-1.9039419264,-0.7716204746,0.0383598783\H,-1.70
23973397,-1.5631017657,-0.6830009086\H,-2.980335003,-0.5821266506,0.07
21383029\H,-1.5609873959,-1.065424755,1.0305225602\S,-1.1481944699,0.8
002092707,-0.4844901305\Se,0.9936262506,0.2772977172,-0.6866493192\H,1
.3832508843,0.5393986581,0.7137516168\\Version=EM64L-G09RevE.01\State=
1-A\HF=-2840.2147513\RMSD=9.209e-09\RMSF=1.379e-05\Dipole=-0.1414618,-
0.5317622,0.4367432\Quadrupole=2.0795954,-1.7678787,-0.3117167,1.65755
52,0.1361643,0.0516988\PG=C01 [X(C1H4S1Se1)]\\@

1\1\GINC-ORC155\FOpt\RB3PW91\6-311+G(d,p)\C1H4S1Se1\MEISTERP\20-Jul-20
17\0\\# opt=readfc freq=noraman b3pw91/6-311+g(d,p) guess=read\\Methyl
Sulfur Selenide\\0,1\C,-1.9045795758,-0.7720303044,0.0377204966\H,-1.
7059336801,-1.5637786454,-0.6845726395\H,-2.9807305667,-0.5809157691,0
.0742305613\H,-1.5598468678,-1.0677678971,1.028948696\S,-1.1471423763,

0.7990291847,-0.4848140021\Se,0.9946190625,0.2761611814,-0.6851444499\
H,1.3846350043,0.5439342498,0.7142633377\\Version=EM64L-G09RevE.01\Sta
te=1-A\HF=-2840.2162259\RMSD=7.935e-09\RMSF=1.274e-05\Dipole=-0.162345
5,-0.5165137,0.4284511\Quadrupole=2.0527749,-1.7900604,-0.2627146,1.63
80265,0.1867143,0.0681745\PG=C01 [X(C1H4S1Se1)]\\@

1\1\GINC-ORC154\FOpt\RB3PW91\6-311G(2d,p)\C1H4S1Se1\MEISTERP\20-Jul-20
17\0\\# opt=readfc freq=noraman b3pw91/6-311g(2d,p) guess=read\\Methyl
Sulfur Selenide\\0,1\C,-1.903298358,-0.7731517423,0.0377737875\H,-1.7
080814219,-1.5618417841,-0.6868506511\H,-2.9778410614,-0.5773321381,0.
0776600485\H,-1.5538928171,-1.0661025707,1.0267507764\S,-1.1433605265,
0.7935626271,-0.4866926137\Se,0.9900921688,0.2749433435,-0.6833020694\
H,1.3774030162,0.5445542647,0.7152927218\\Version=EM64L-G09RevE.01\Sta
te=1-A\HF=-2840.2183912\RMSD=5.473e-09\RMSF=1.226e-05\Dipole=-0.181448
7,-0.5030016,0.3932246\Quadrupole=2.14048,-1.7312797,-0.4092003,1.5337
58,0.0394817,0.0198439\PG=C01 [X(C1H4S1Se1)]\\@

1\1\GINC-ORC154\FOpt\RB3PW91\6-311G(df,p)\C1H4S1Se1\MEISTERP\20-Jul-20
17\0\\# opt=readfc freq=noraman b3pw91/6-311g(df,p) guess=read\\Methyl
Sulfur Selenide\\0,1\C,-1.9020394843,-0.7704632197,0.0383438919\H,-1.
7001326596,-1.5637685791,-0.6809683958\H,-2.9781945778,-0.5806156387,0
.070528958\H,-1.5608932531,-1.0649190646,1.0310028308\S,-1.142841369,0
.7977782384,-0.4847647742\Se,0.9847518749,0.2804899056,-0.6874457935\H
,1.380370469,0.5361303582,0.7139352828\\Version=EM64L-G09RevE.01\State
=1-A\HF=-2840.2210466\RMSD=7.063e-09\RMSF=1.252e-05\Dipole=-0.1489807,
-0.5278421,0.4363255\Quadrupole=1.9245021,-1.6720244,-0.2524777,1.6926
281,0.1401716,0.0343509\PG=C01 [X(C1H4S1Se1)]\\@

1\1\GINC-ORC152\FOpt\RB3PW91\6-311+G(2df,p)\C1H4S1Se1\MEISTERP\20-Jul-
2017\0\\# opt=readfc freq=noraman b3pw91/6-311+g(2df,p) guess=read\\Me
thyl Sulfur Selenide\\0,1\C,-1.9008838609,-0.7710960517,0.0370177332\H
,-1.706789671,-1.5623863083,-0.6857080337\H,-2.9755501844,-0.575687313
8,0.0771884046\H,-1.5526857199,-1.0660304626,1.0262736674\S,-1.1403018
444,0.7898605835,-0.4865895728\Se,0.9811148617,0.2766648319,-0.6827598
116\H,1.3761174188,0.543306721,0.715209613\\Version=EM64L-G09RevE.01\S
tate=1-A\HF=-2840.2258965\RMSD=4.773e-09\RMSF=1.195e-05\Dipole=-0.2013
447,-0.4849261,0.3849705\Quadrupole=1.9810316,-1.6914707,-0.2895609,1.
5311213,0.102775,0.0351378\PG=C01 [X(C1H4S1Se1)]\\@

1\1\GINC-ORC179\FOpt\RB3PW91\6-311++G(3df,3pd)\C1H4S1Se1\MEISTERP\20-J
ul-2017\0\\# opt=readfc freq=noraman b3pw91/6-311++g(3df,3pd) guess=re
ad\\Methyl Sulfur Selenide\\0,1\C,-1.9001922797,-0.7708663778,0.036768
3107\H,-1.7083503747,-1.5624879883,-0.6840652104\H,-2.9731415337,-0.57
39351661,0.0766576423\H,-1.5535903636,-1.0648322789,1.0252920097\S,-1.
1397213188,0.7856209279,-0.4876536583\Se,0.9817351961,0.2779040276,-0.
6824228342\H,1.3742816743,0.5432288556,0.7160557401\\Version=EM64L-G09
RevE.01\State=1-A\HF=-2840.2320493\RMSD=4.208e-09\RMSF=1.323e-05\Dipol
e=-0.1948439,-0.4809573,0.3769073\Quadrupole=1.9584766,-1.6273541,-0.3
311225,1.5446034,0.0549997,0.0056228\PG=C01 [X(C1H4S1Se1)]\\@

CH_3SSeH wB97XD

1\1\GINC-ORC128\FOpt\RwB97XD\6-31G(d)\C1H4S1Se1\MEISTERP\20-Jul-2017\0
\\# opt=readfc freq=noraman wb97xd/6-31g(d) guess=read\\Methyl Sulfur
Selenide\\0,1\C,-1.8928606381,-0.7584322704,0.0410095259\H,-1.66683485
5,-1.552828063,-0.6727162446\H,-2.9741914857,-0.5957448648,0.073026181
4\H,-1.5401217602,-1.0387942884,1.0356018399\S,-1.1508334493,0.8185460

929,-0.4935321638\Se,0.9630265682,0.2667982195,-0.7006826391\H,1.34283
66201,0.4950871741,0.7179255002\\Version=EM64L-G09RevE.01\State=1-A\HF
=-2838.0705058\RMSD=4.303e-09\RMSF=1.789e-05\Dipole=-0.1434754,-0.5477
603,0.4436306\Quadrupole=1.8954102,-1.7116262,-0.183784,1.6283557,0.15
88576,-0.0549434\PG=C01 [X(C1H4S1Se1)]\\@

1\1\GINC-ORC122\FOpt\RwB97XD\6-311G(d)\C1H4S1Se1\MEISTERP\20-Jul-2017\
0\\# opt=readfc freq=noraman wb97xd/6-311g(d) guess=read\\Methyl Sulfu
r Selenide\\0,1\C,-1.8978679165,-0.7649970767,0.0390823646\H,-1.687958
6343,-1.5578580158,-0.6774524655\H,-2.9754395908,-0.5902399472,0.07340
09465\H,-1.5512699791,-1.0528013834,1.0310217981\S,-1.150490559,0.8079
478706,-0.4887913885\Se,0.9813751011,0.2739429102,-0.6932992057\H,1.36
26725786,0.5186376423,0.7166699505\\Version=EM64L-G09RevE.01\State=1-A
\HF=-2840.2803356\RMSD=7.092e-09\RMSF=2.607e-05\Dipole=-0.124352,-0.54
83765,0.4391147\Quadrupole=2.0473085,-1.8301682,-0.2171403,1.6537148,0
.1531343,0.0237404\PG=C01 [X(C1H4S1Se1)]\\@

1\1\GINC-ORC119\FOpt\RwB97XD\6-311G(d,p)\C1H4S1Se1\MEISTERP\20-Jul-201
7\0\\# opt=readfc freq=noraman wb97xd/6-311g(d,p) guess=read\\Methyl S
ulfur Selenide\\0,1\C,-1.8991340873,-0.7671270068,0.0393314159\H,-1.68
81989447,-1.5563969162,-0.6810537812\H,-2.9762379175,-0.5888712214,0.0
729494886\H,-1.5516257852,-1.0561162343,1.0306900944\S,-1.1511433482,0
.8073519541,-0.4840646994\Se,0.9828159668,0.2759094964,-0.6886900735\H
,1.3645451161,0.5198819283,0.7114695552\\Version=EM64L-G09RevE.01\Stat
e=1-A\HF=-2840.287763\RMSD=4.318e-09\RMSF=2.971e-05\Dipole=-0.1218713,
-0.5434959,0.4386207\Quadrupole=2.0642501,-1.7944846,-0.2697655,1.6609
098,0.1564399,0.0156598\PG=C01 [X(C1H4S1Se1)]\\@

1\1\GINC-ORC116\FOpt\RwB97XD\6-311+G(d,p)\C1H4S1Se1\MEISTERP\20-Jul-20
17\0\\# opt=readfc freq=noraman wb97xd/6-311+g(d,p) guess=read\\Methyl
 Sulfur Selenide\\0,1\C,-1.8996763061,-0.7676654091,0.0385162121\H,-1.
6927292181,-1.5572478543,-0.6830599878\H,-2.9764167055,-0.5872567033,0
.0758185653\H,-1.5498381365,-1.0587850647,1.0286650951\S,-1.1500081842
,0.8057226444,-0.4850073353\Se,0.9840828233,0.2749661187,-0.6867916484
\H,1.3656067272,0.5248982683,0.7124910989\\Version=EM64L-G09RevE.01\St
ate=1-A\HF=-2840.2892453\RMSD=4.481e-09\RMSF=2.562e-05\Dipole=-0.14349
06,-0.5272733,0.4331143\Quadrupole=2.0495194,-1.8194185,-0.2301009,1.6
459896,0.1997251,0.0303706\PG=C01 [X(C1H4S1Se1)]\\@

1\1\GINC-ORC114\FOpt\RwB97XD\6-311G(2d,p)\C1H4S1Se1\MEISTERP\20-Jul-20
17\0\\# opt=readfc freq=noraman wb97xd/6-311g(2d,p) guess=read\\Methyl
 Sulfur Selenide\\0,1\C,-1.8985360642,-0.7688990536,0.0387605104\H,-1.
6934031871,-1.5551091415,-0.6850123232\H,-2.9739393453,-0.5848941166,0
.0780075995\H,-1.5452786805,-1.057156187,1.0270784123\S,-1.1469797346,
0.8012821994,-0.4854012685\Se,0.9789302524,0.2744381755,-0.6855395907\
H,1.3602277593,0.5249701237,0.7127386602\\Version=EM64L-G09RevE.01\Sta
te=1-A\HF=-2840.2915308\RMSD=5.203e-09\RMSF=2.834e-05\Dipole=-0.161711
8,-0.5124028,0.3968027\Quadrupole=2.1399729,-1.7665344,-0.3734386,1.53
33106,0.0630179,-0.0127434\PG=C01 [X(C1H4S1Se1)]\\@

1\1\GINC-ORC112\FOpt\RwB97XD\6-311G(df,p)\C1H4S1Se1\MEISTERP\20-Jul-20
17\0\\# opt=readfc freq=noraman wb97xd/6-311g(df,p) guess=read\\Methyl
 Sulfur Selenide\\0,1\C,-1.8974300866,-0.7660267893,0.0396142331\H,-1.
6843971831,-1.5569862678,-0.6783421232\H,-2.9744978118,-0.5881700918,0
.0697757448\H,-1.5532210063,-1.0552072231,1.0321332829\S,-1.1467732689
,0.8052398483,-0.4830621971\Se,0.9742355935,0.2801496588,-0.6903788279
\H,1.3631047632,0.5156328649,0.7108918873\\Version=EM64L-G09RevE.01\St

ate=1-A\HF=-2840.2942278\RMSD=4.364e-09\RMSF=2.891e-05\Dipole=-0.12999
86,-0.5400928,0.4385466\Quadrupole=1.9112203,-1.7002167,-0.2110036,1.6
981273,0.1635237,-0.003114\PG=C01 [X(C1H4S1Se1)]\\@

1\1\GINC-ORC110\FOpt\RwB97XD\6-311+G(2df,p)\C1H4S1Se1\MEISTERP\20-Jul-
2017\0\\# opt=readfc freq=noraman wb97xd/6-311+g(2df,p) guess=read\\Me
thyl Sulfur Selenide\\0,1\C,-1.8962916364,-0.7670244599,0.0380707866\H
,-1.6922216516,-1.5558649904,-0.6837171267\H,-2.9718047427,-0.58333046
5,0.0772048127\H,-1.5447682935,-1.057279934,1.0268289931\S,-1.14423189
49,0.7973897226,-0.4851206168\Se,0.9707152046,0.2771857593,-0.68529387
65\H,1.3596240145,0.5235563674,0.7126590276\\Version=EM64L-G09RevE.01\
State=1-A\HF=-2840.2991903\RMSD=4.867e-09\RMSF=2.869e-05\Dipole=-0.183
146,-0.4941508,0.3906808\Quadrupole=1.9893216,-1.7290608,-0.2602608,1.
5402342,0.1219319,-0.0014509\PG=C01 [X(C1H4S1Se1)]\\@

1\1\GINC-ORC109\FOpt\RwB97XD\6-311++G(3df,3pd)\C1H4S1Se1\MEISTERP\20-J
ul-2017\0\\# opt=readfc freq=noraman wb97xd/6-311++g(3df,3pd) guess=re
ad\\Methyl Sulfur Selenide\\0,1\C,-1.8956870338,-0.7667435787,0.038230
9103\H,-1.6918915293,-1.5556108059,-0.6811586022\H,-2.9695563263,-0.58
24706086,0.0747472562\H,-1.5475977201,-1.0557110485,1.0267752392\S,-1.
1442696297,0.7936844393,-0.4844453072\Se,0.9710847777,0.2790622578,-0.
6858798195\H,1.3589384615,0.5224213446,0.7123623231\\Version=EM64L-G09
RevE.01\State=1-A\HF=-2840.3056166\RMSD=5.421e-09\RMSF=3.469e-05\Dipol
e=-0.1780437,-0.4904879,0.3842748\Quadrupole=1.9773208,-1.6734461,-0.3
038747,1.5599196,0.0810658,-0.0316594\PG=C01 [X(C1H4S1Se1)]\\@

CH_3SSeH M062X

1\1\GINC-ORC151\FOpt\RM062X\6-31G(d)\C1H4S1Se1\MEISTERP\20-Jul-2017\0\
\# opt=readfc freq=noraman m062x/6-31g(d) guess=read\\Methyl Sulfur Se
lenide\\0,1\C,-1.8904348121,-0.7580126074,0.0361310766\H,-1.691461977,
-1.5492648436,-0.6875892804\H,-2.9678635355,-0.5851789444,0.103094281\
H,-1.5061055729,-1.0432623183,1.0164613208\S,-1.1501556565,0.812545800
5,-0.5148942943\Se,0.96081022,0.2420555812,-0.6834852044\H,1.326232334
1,0.5157493321,0.7309141006\\Version=EM64L-G09RevE.01\State=1-A\HF=-28
38.0599882\RMSD=9.846e-09\RMSF=4.603e-05\Dipole=-0.1788162,-0.509813,0
.4590253\Quadrupole=1.8416712,-1.6648236,-0.1768476,1.5723702,0.207356
9,0.0296332\PG=C01 [X(C1H4S1Se1)]\\@

1\1\GINC-ORC150\FOpt\RM062X\6-311G(d)\C1H4S1Se1\MEISTERP\20-Jul-2017\0
\\# opt=readfc freq=noraman m062x/6-311g(d) guess=read\\Methyl Sulfur
Selenide\\0,1\C,-1.8952201416,-0.7635486934,0.0366406451\H,-1.69631013
95,-1.5526227067,-0.6853987419\H,-2.9705746094,-0.5858473757,0.0905991
872\H,-1.5292078334,-1.0517088612,1.0201841244\S,-1.1521535105,0.80707
60589,-0.5006852783\Se,0.9766870873,0.2548979169,-0.6842576622\H,1.347
8001471,0.5263856611,0.7235497257\\Version=EM64L-G09RevE.01\State=1-A\
HF=-2840.2278487\RMSD=4.597e-09\RMSF=3.528e-05\Dipole=-0.1546933,-0.52
35425,0.4604569\Quadrupole=2.0071494,-1.810425,-0.1967244,1.6114822,0.
204132,0.0831397\PG=C01 [X(C1H4S1Se1)]\\@

1\1\GINC-ORC150\FOpt\RM062X\6-311G(d,p)\C1H4S1Se1\MEISTERP\20-Jul-2017
\0\\# opt=readfc freq=noraman m062x/6-311g(d,p) guess=read\\Methyl Sul
fur Selenide\\0,1\C,-1.8963331823,-0.7652102133,0.0373550352\H,-1.6932
905687,-1.5513395006,-0.6869442931\H,-2.9716440789,-0.5855646828,0.087
586955\H,-1.5314834724,-1.0526832457,1.0216001066\S,-1.1533185727,0.80
73453312,-0.4955287565\Se,0.9773467659,0.2581943075,-0.6814139406\H,1.
3497441092,0.5238900038,0.7179768934\\Version=EM64L-G09RevE.01\State=1

-A\HF=-2840.2339744\RMSD=7.257e-09\RMSF=3.128e-05\Dipole=-0.1521299,-0
.5219023,0.4632982\Quadrupole=2.0257695,-1.7857163,-0.2400532,1.625143
9,0.2147154,0.0658334\PG=C01 [X(C1H4S1Se1)]\\@

1\1\GINC-ORC147\FOpt\RM062X\6-311+G(d,p)\C1H4S1Se1\MEISTERP\20-Jul-201
7\0\\# opt=readfc freq=noraman m062x/6-311+g(d,p) guess=read\\Methyl S
ulfur Selenide\\0,1\C,-1.8968780221,-0.7655186367,0.0369655513\H,-1.69
47069771,-1.5518970679,-0.6877242553\H,-2.9721247834,-0.5850608188,0.0
879376146\H,-1.5317775903,-1.0540191497,1.0210848102\S,-1.1526651832,0
.806605449,-0.4947732042\Se,0.9776381538,0.2581166881,-0.6808032654\H,
1.3515354024,0.526405536,0.7179447488\\Version=EM64L-G09RevE.01\State=
1-A\HF=-2840.2354859\RMSD=7.397e-09\RMSF=3.518e-05\Dipole=-0.1735229,-
0.5052374,0.4542743\Quadrupole=2.0119909,-1.8111832,-0.2008076,1.60608
57,0.2625834,0.0735696\PG=C01 [X(C1H4S1Se1)]\\@

1\1\GINC-ORC141\FOpt\RM062X\6-311G(2d,p)\C1H4S1Se1\MEISTERP\20-Jul-201
7\0\\# opt=readfc freq=noraman m062x/6-311g(2d,p) guess=read\\Methyl S
ulfur Selenide\\0,1\C,-1.8960268137,-0.7670055626,0.0371935439\H,-1.69
63027343,-1.5494847513,-0.690249851\H,-2.9696262865,-0.5826044965,0.09
13033877\H,-1.525579919,-1.0525641338,1.0186093983\S,-1.1501324319,0.8
02007734,-0.4956747075\Se,0.9735397898,0.2571842585,-0.6793020145\H,1.
3451493955,0.5270989517,0.7187522431\\Version=EM64L-G09RevE.01\State=1
-A\HF=-2840.2378589\RMSD=4.120e-09\RMSF=3.136e-05\Dipole=-0.1883828,-0
.4901682,0.4176904\Quadrupole=2.1094921,-1.7661362,-0.3433559,1.481505
2,0.1228156,0.0342911\PG=C01 [X(C1H4S1Se1)]\\@

1\1\GINC-ORC134\FOpt\RM062X\6-311G(df,p)\C1H4S1Se1\MEISTERP\20-Jul-201
7\0\\# opt=readfc freq=noraman m062x/6-311g(df,p) guess=read\\Methyl S
ulfur Selenide\\0,1\C,-1.894741527,-0.7639408216,0.0375475924\H,-1.689
1969872,-1.5522232315,-0.6840803442\H,-2.970310309,-0.5854416792,0.083
9889099\H,-1.5337997529,-1.0521357936,1.0233460683\S,-1.1493863166,0.8
053969924,-0.4941110252\Se,0.9684706025,0.2636129166,-0.6833160056\H,1
.3499852901,0.5193636169,0.7172568045\\Version=EM64L-G09RevE.01\State=
1-A\HF=-2840.2406409\RMSD=6.469e-09\RMSF=3.688e-05\Dipole=-0.157335,-0
.5203737,0.4598428\Quadrupole=1.8624677,-1.6791975,-0.1832702,1.673208
2,0.2120552,0.0395842\PG=C01 [X(C1H4S1Se1)]\\@

1\1\GINC-ORC134\FOpt\RM062X\6-311+G(2df,p)\C1H4S1Se1\MEISTERP\20-Jul-2
017\0\\# opt=readfc freq=noraman m062x/6-311+g(2df,p) guess=read\\Meth
yl Sulfur Selenide\\0,1\C,-1.893925713,-0.7650432308,0.0366624987\H,-1
.6938926113,-1.5507939994,-0.6880541199\H,-2.9680023432,-0.5816560446,
0.0888659731\H,-1.5268184128,-1.0523994984,1.0194435319\S,-1.147390735
1,0.7984954147,-0.4944975192\Se,0.9651248458,0.2617325323,-0.679987272
2\H,1.3459259696,0.5242968262,0.7181989075\\Version=EM64L-G09RevE.01\S
tate=1-A\HF=-2840.2456786\RMSD=4.412e-09\RMSF=3.106e-05\Dipole=-0.2076
676,-0.4728591,0.4060885\Quadrupole=1.9483786,-1.7132861,-0.2350926,1.
4991515,0.1747309,0.0323354\PG=C01 [X(C1H4S1Se1)]\\@

1\1\GINC-ORC128\FOpt\RM062X\6-311++G(3df,3pd)\C1H4S1Se1\MEISTERP\20-Ju
l-2017\0\\# opt=readfc freq=noraman m062x/6-311++g(3df,3pd) guess=read
\\Methyl Sulfur Selenide\\0,1\C,-1.8932304505,-0.7647994283,0.03668872
11\H,-1.693876771,-1.5503982222,-0.6855961329\H,-2.9654342856,-0.58091
40568,0.087497298\H,-1.5284280356,-1.0505752645,1.0187182339\S,-1.1474
414511,0.7953617355,-0.4949171718\Se,0.965646481,0.2635639957,-0.68011
92696\H,1.3437855128,0.5223932406,0.7183603213\\Version=EM64L-G09RevE.
01\State=1-A\HF=-2840.2524529\RMSD=6.263e-09\RMSF=2.734e-05\Dipole=-0.
2000209,-0.4695592,0.3953965\Quadrupole=1.9325754,-1.6448021,-0.287773

3,1.5081354,0.1231558,-0.0055846\PG=C01 [X(C1H4S1Se1)]\\@

CH_3SSeH M08HX

1\1\GINC-ORC296\FOpt\RM08HX\6-31G(d)\C1H4S1Se1\MEISTERP\18-Aug-2017\0\
\# opt=readfc freq=noraman m08hx/6-31g(d) guess=read\\Methyl Sulfur Se
lenide\\0,1\C,-1.8837966513,-0.7498007715,0.0402567709\H,-1.6540860021
,-1.5443399215,-0.6779272042\H,-2.9706618113,-0.6024141968,0.086320881
1\H,-1.5146601702,-1.0295807512,1.0333323445\S,-1.1589379025,0.8303952
069,-0.499761021\Se,0.948119449,0.2536742668,-0.7028968958\H,1.3150440
884,0.4766981674,0.7213071246\\Version=EM64L-G16RevA.03\State=1-A\HF=-
2838.0923634\RMSD=1.831e-09\RMSF=2.080e-05\Dipole=-0.1952075,-0.509315
8,0.4565591\Quadrupole=1.8200867,-1.6715037,-0.148583,1.4966917,0.2105
866,-0.0612141\PG=C01 [X(C1H4S1Se1)]\\@

1\1\GINC-ORC294\FOpt\RM08HX\6-311G(d)\C1H4S1Se1\MEISTERP\18-Aug-2017\0
\\# opt=readfc freq=noraman m08hx/6-311g(d) guess=read\\Methyl Sulfur
Selenide\\0,1\C,-1.8905456544,-0.7567043057,0.0384012102\H,-1.67733118
48,-1.5505197883,-0.6811529396\H,-2.972879684,-0.5936366141,0.08501141
24\H,-1.5279040811,-1.041808907,1.0290582425\S,-1.1563610654,0.8177717
705,-0.4981492285\Se,0.9686801307,0.2548438082,-0.6934315724\H,1.33736
25391,0.5046860363,0.7208948754\\Version=EM64L-G16RevA.03\State=1-A\HF
=-2840.2446217\RMSD=4.120e-09\RMSF=1.100e-05\Dipole=-0.1757848,-0.5162
078,0.4564559\Quadrupole=1.9848453,-1.8142404,-0.170605,1.5413869,0.19
87257,0.0446123\PG=C01 [X(C1H4S1Se1)]\\@

1\1\GINC-ORC293\FOpt\RM08HX\6-311G(d,p)\C1H4S1Se1\MEISTERP\18-Aug-2017
\0\\# opt=readfc freq=noraman m08hx/6-311g(d,p) guess=read\\Methyl Sul
fur Selenide\\0,1\C,-1.8927649029,-0.7599463698,0.0381855567\H,-1.6817
774596,-1.5485695339,-0.6876678134\H,-2.9740531286,-0.5911725884,0.087
1751161\H,-1.5267805069,-1.0491409227,1.0263022083\S,-1.1560272696,0.8
157905467,-0.4923651256\Se,0.9705781558,0.2534584699,-0.6867915143\H,1
.3418461119,0.5142123981,0.7157935722\\Version=EM64L-G16RevA.03\State=
1-A\HF=-2840.2507214\RMSD=4.808e-09\RMSF=8.710e-06\Dipole=-0.1721851,-
0.5099295,0.4546441\Quadrupole=2.0037007,-1.7757602,-0.2279405,1.56018
79,0.2041721,0.0588108\PG=C01 [X(C1H4S1Se1)]\\@

1\1\GINC-ORC288\FOpt\RM08HX\6-311+G(d,p)\C1H4S1Se1\MEISTERP\18-Aug-201
7\0\\# opt=readfc freq=noraman m08hx/6-311+g(d,p) guess=read\\Methyl S
ulfur Selenide\\0,1\C,-1.8951196355,-0.7617052761,0.0368423821\H,-1.69
11952704,-1.5491652841,-0.6927845042\H,-2.9755780332,-0.5879076,0.0905
848462\H,-1.5271099578,-1.0572566357,1.0226349246\S,-1.1527192757,0.81
23645435,-0.4900520669\Se,0.9727754704,0.2464825329,-0.6814448564\H,1.
3499677024,0.5318197195,0.7148512746\\Version=EM64L-G16RevA.03\State=1
-A\HF=-2840.2523674\RMSD=3.611e-09\RMSF=1.653e-05\Dipole=-0.1912329,-0
.4868639,0.444037\Quadrupole=2.002129,-1.7970923,-0.2050366,1.5571106,
0.263797,0.1136893\PG=C01 [X(C1H4S1Se1)]\\@

1\1\GINC-ORC176\FOpt\RM08HX\6-311G(2d,p)\C1H4S1Se1\MEISTERP\18-Aug-201
7\0\\# opt=readfc freq=noraman m08hx/6-311g(2d,p) guess=read\\Methyl S
ulfur Selenide\\0,1\C,-1.8926726296,-0.761756567,0.0374782738\H,-1.688
0512073,-1.5476528996,-0.6912355196\H,-2.9718638381,-0.5867135987,0.09
2163652\H,-1.5207991189,-1.0500060099,1.022232935\S,-1.1515030019,0.80
84896135,-0.4944042644\Se,0.9683237489,0.2522917494,-0.6834519136\H,1.
3375870469,0.5199797123,0.7178488367\\Version=EM64L-G16RevA.03\State=1
-A\HF=-2840.2547105\RMSD=4.162e-09\RMSF=8.505e-06\Dipole=-0.2005038,-0
.4796663,0.4083353\Quadrupole=2.0833386,-1.7565726,-0.326766,1.4309041

```
,0.1053302,0.0304849\PG=C01 [X(C1H4S1Se1)]\\@
```

```
1\1\GINC-ORC176\FOpt\RM08HX\6-311G(df,p)\C1H4S1Se1\MEISTERP\18-Aug-201
 7\0\\# opt=readfc freq=noraman m08hx/6-311g(df,p) guess=read\\Methyl S
 ulfur Selenide\\0,1\C,-1.8898926896,-0.757902278,0.0391167732\H,-1.671
 8804531,-1.5493181798,-0.6820032881\H,-2.9721557748,-0.5940866931,0.08
 12247527\H,-1.5298575749,-1.0450582689,1.0303538097\S,-1.1544466698,0.
 816424867,-0.4904201379\Se,0.9604509379,0.2654964752,-0.6925003507\H,1
 .3388032244,0.4990760776,0.714860441\\Version=EM64L-G16RevA.03\State=1
 -A\HF=-2840.2576594\RMSD=4.244e-09\RMSF=9.421e-06\Dipole=-0.1668171,-0
 .5118118,0.4509312\Quadrupole=1.8279224,-1.6659742,-0.1619482,1.603479
 3,0.2020475,-0.0016383\PG=C01 [X(C1H4S1Se1)]\\@
```

```
1\1\GINC-ORC105\FOpt\RM08HX\6-311+G(2df,p)\C1H4S1Se1\MEISTERP\18-Aug-2
 017\0\\# opt=readfc freq=noraman m08hx/6-311+g(2df,p) guess=read\\Meth
 yl Sulfur Selenide\\0,1\C,-1.8897452379,-0.7593906597,0.0373933166\H,-
 1.6814916649,-1.5489921979,-0.6873257289\H,-2.9700286103,-0.587829625,
 0.0877639573\H,-1.5230223643,-1.047855441,1.0248020725\S,-1.1506578395
 ,0.8068988185,-0.4921652939\Se,0.9587759639,0.2611104195,-0.6868341558
 \H,1.3371907531,0.5106906857,0.7169978322\\Version=EM64L-G16RevA.03\St
 ate=1-A\HF=-2840.2631119\RMSD=8.835e-09\RMSF=6.828e-06\Dipole=-0.20833
 03,-0.4616204,0.3940628\Quadrupole=1.9196697,-1.711511,-0.2081587,1.43
 86695,0.161687,0.0132668\PG=C01 [X(C1H4S1Se1)]\\@
```

```
1\1\GINC-ORC27\FOpt\RM08HX\6-311++G(3df,3pd)\C1H4S1Se1\MEISTERP\18-Aug
 -2017\0\\# opt=readfc freq=noraman m08hx/6-311++g(3df,3pd) guess=read\
 \Methyl Sulfur Selenide\\0,1\C,-1.8880691549,-0.7591254911,0.037919750
 1\H,-1.6782706447,-1.5467255474,-0.6847995744\H,-2.9661054047,-0.58942
 98477,0.0878267768\H,-1.521193741,-1.0438514922,1.0236020704\S,-1.1530
 295134,0.8060490251,-0.4927530652\Se,0.9573801724,0.2654028222,-0.6883
 643757\H,1.3303092863,0.5023125311,0.717200418\\Version=EM64L-G16RevA.
 03\State=1-A\HF=-2840.2702544\RMSD=8.105e-09\RMSF=1.231e-05\Dipole=-0.
 2007557,-0.4645738,0.3933263\Quadrupole=1.9134578,-1.6606539,-0.252803
 9,1.4726562,0.1153857,-0.0411819\PG=C01 [X(C1H4S1Se1)]\\@
```

CH_3SSeH QCISD

```
1\1\GINC-ORC269\FOpt\RQCISD-FC\6-311+G(2df,p)\C1H4S1Se1\MEISTERP\20-Au
 g-2017\0\\# opt freq=noraman qcisd/6-311+g(2df,p)\\Methyl Sulfur Selen
 ide\\0,1\C,2.0578691961,0.755709673,-0.008409687\H,1.845722322,1.36542
 31068,-0.8868000486\H,3.0931879169,0.4063011287,-0.0518410448\H,1.9138
 176853,1.3419716012,0.8996710738\S,1.0181215219,-0.7361811511,0.009187
 5537\Se,-1.0061708487,0.1178174732,-0.045981628\H,-1.1766987936,0.2901
 011683,1.4086527809\\Version=EM64L-G09RevE.01\State=1-A\HF=-2837.55663
 61\MP2=-2838.0519878\MP3=-2838.095173\MP4D=-2838.1089261\MP4DQ=-2838.0
 943827\MP4SDQ=-2838.0977645\QCISD=-2838.0977371\RMSD=4.839e-09\RMSF=1.
 111e-05\Dipole=0.3152747,0.5100955,0.2217639\PG=C01 [X(C1H4S1Se1)]\\@
```

CH_3SSe^- B3LYP

```
1\1\GINC-ORC389\FOpt\RB3LYP\6-31G(d)\C1H3S1Se1(1-)\MEISTERP\27-May-201
 7\0\\# opt freq b3lyp/6-31g(d)\\Methyl Sulfur Selenide\\-1,1\C,-0.4827
 416621,2.0115061186,-0.012907642\H,-0.4683160648,0.9309452052,-0.19569
 95325\H,-0.1394321101,2.5398656837,-0.9148921472\H,-1.5138404094,2.306
 9779989,0.2124023716\S,0.6216125855,2.4342576769,1.3909310499\Se,-0.29
 94992622,1.2342237298,3.0773755308\\Version=EM64L-G16RevA.03\State=1-A
 '\HF=-2837.5253918\RMSD=2.396e-09\RMSF=5.915e-05\Dipole=-0.312248,0.02
```

11123,-0.8711406\Quadrupole=0.3885141,-0.1389603,-0.2495538,-0.4067585
,1.119039,1.0687213\PG=CS [SG(C1H1S1Se1),X(H2)]\\@

1\1\GINC-ORC226\FOpt\RB3LYP\6-311G(d)\C1H3S1Se1(1-)\MEISTERP\27-May-20
17\0\\# opt freq b3lyp/6-311g(d)\\Methyl Sulfur Selenide\\-1,1\C,-0.48
64255938,2.0096562052,-0.0161080649\H,-0.4690705803,0.9330916185,-0.20
31171963\H,-0.1360837104,2.540914906,-0.9098515699\H,-1.5141206048,2.3
085001513,0.2047995654\S,0.6183496901,2.4341566984,1.3829122514\Se,-0.
2948661285,1.2314568333,3.098574634\\Version=EM64L-G16RevA.03\State=1-
A'\HF=-2839.7488875\RMSD=8.721e-09\RMSF=6.609e-05\Dipole=-0.3200962,0.
0259036,-0.9074023\Quadrupole=0.2570594,-0.1927783,-0.0642811,-0.38991
15,1.0206469,0.9048418\PG=CS [SG(C1H1S1Se1),X(H2)]\\@

1\1\GINC-ORC198\FOpt\RB3LYP\6-311G(d,p)\C1H3S1Se1(1-)\MEISTERP\27-May-
2017\0\\# opt freq b3lyp/6-311g(d,p)\\Methyl Sulfur Selenide\\-1,1\C,-
0.4875966033,2.0092590718,-0.0177690481\H,-0.4686322088,0.9323561313,-
0.203044367\H,-0.1345682727,2.541933758,-0.9094044924\H,-1.5146376293,
2.3090220774,0.2052453166\S,0.6172809834,2.4335022166,1.382381084\Se,-
0.2940631959,1.2317031577,3.0998011294\\Version=EM64L-G16RevA.03\State
=1-A'\HF=-2839.7532834\RMSD=4.251e-09\RMSF=7.078e-05\Dipole=-0.315732,
0.0265719,-0.8984748\Quadrupole=0.2728206,-0.1683108,-0.1045098,-0.387
7532,0.9955636,0.9044499\PG=CS [SG(C1H1S1Se1),X(H2)]\\@

1\1\GINC-ORC369\FOpt\RB3LYP\6-311+G(d,p)\C1H3S1Se1(1-)\MEISTERP\27-May
-2017\0\\# opt freq b3lyp/6-311+g(d,p)\\Methyl Sulfur Selenide\\-1,1\C
,-0.4891105559,2.007739558,-0.0165241958\H,-0.469593649,0.931426775,-0
.2052813627\H,-0.1315370559,2.5428532531,-0.9047391\H,-1.5163859399,2.
3091283348,0.2033154618\S,0.6159511927,2.4324057955,1.3826711657\Se,-0
.2915409202,1.2342226963,3.0977676492\\Version=EM64L-G16RevA.03\State=
1-A'\HF=-2839.7604379\RMSD=5.502e-09\RMSF=7.002e-05\Dipole=-0.2146237,
0.1618845,-1.0956891\Quadrupole=0.3378881,-0.1423859,-0.1955022,-0.264
3683,0.9457815,0.8705109\PG=CS [SG(C1H1S1Se1),X(H2)]\\@

1\1\GINC-ORC389\FOpt\RB3LYP\6-311G(2d,p)\C1H3S1Se1(1-)\MEISTERP\27-May
-2017\0\\# opt freq b3lyp/6-311g(2d,p)\\Methyl Sulfur Selenide\\-1,1\C
,-0.4882316385,2.0085015177,-0.0168416459\H,-0.4683674275,0.9326676858
,-0.2028268371\H,-0.1332139422,2.5419843347,-0.9061053399\H,-1.5142132
584,2.3091235932,0.2054005535\S,0.6127876035,2.4291348399,1.3855952701
\Se,-0.2909782655,1.2363644419,3.0919876151\\Version=EM64L-G16RevA.03\
State=1-A'\HF=-2839.7555112\RMSD=6.871e-09\RMSF=7.293e-05\Dipole=-0.30
46173,0.0229472,-0.8577783\Quadrupole=0.3312071,-0.1439626,-0.1872445,
-0.3341944,0.9921758,0.9232158\PG=CS [SG(C1H1S1Se1),X(H2)]\\@

1\1\GINC-ORC389\FOpt\RB3LYP\6-311G(df,p)\C1H3S1Se1(1-)\MEISTERP\27-May
-2017\0\\# opt freq b3lyp/6-311g(df,p)\\Methyl Sulfur Selenide\\-1,1\C
,-0.487124933,2.009034038,-0.0158019007\H,-0.4698158918,0.9323321244,-
0.2034349847\H,-0.1328581416,2.5423354201,-0.9063775926\H,-1.515128223
,2.3080858835,0.2045841638\S,0.6130471674,2.4287411011,1.3875878554\Se
,-0.2903369046,1.2372478456,3.0906520807\\Version=EM64L-G16RevA.03\Sta
te=1-A'\HF=-2839.7589206\RMSD=3.929e-09\RMSF=6.517e-05\Dipole=-0.30867
46,0.0308885,-0.894949\Quadrupole=0.3261557,-0.1102508,-0.2159048,-0.4
765312,1.0297881,1.0100004\PG=CS [SG(C1H1S1Se1),X(H2)]\\@

1\1\GINC-ORC199\FOpt\RB3LYP\6-311+G(2df,p)\C1H3S1Se1(1-)\MEISTERP\27-M
ay-2017\0\\# opt freq b3lyp/6-311+g(2df,p)\\Methyl Sulfur Selenide\\-1
,1\C,-0.4889994876,2.0066910432,-0.0127042791\H,-0.470200518,0.9316459
829,-0.2049052538\H,-0.127738266,2.5435419082,-0.8973289032\H,-1.51627

02081,2.3083965153,0.2034095163\S,0.6064979719,2.4239538323,1.38695099
39\Se,-0.2855064212,1.2435471309,3.0817875423\\Version=EM64L-G16RevA.0
3\State=1-A'\HF=-2839.7683528\RMSD=7.848e-09\RMSF=5.950e-05\Dipole=-0.
2006892,0.1544848,-1.0350394\Quadrupole=0.4090282,-0.0818378,-0.327190
3,-0.2819079,0.9466042,0.9380415\PG=CS [SG(C1H1S1Se1),X(H2)]\\@

1\1\GINC-ORC40\FOpt\RB3LYP\6-311++G(3df,3pd)\C1H3S1Se1(1-)\MEISTERP\27
-May-2017\0\\# opt freq b3lyp/6-311++g(3df,3pd)\\Methyl Sulfur Selenid
e\\-1,1\C,-0.4889829167,2.0064024917,-0.0116888915\H,-0.4704470352,0.9
330581543,-0.2048866557\H,-0.1272124791,2.5429571951,-0.8940103513\H,-
1.5150521094,2.3078810803,0.2028564275\S,0.6041516838,2.4228325311,1.3
847207809\Se,-0.2846740732,1.2446449602,3.080218304\\Version=EM64L-G16
RevA.03\State=1-A'\HF=-2839.7746868\RMSD=7.241e-09\RMSF=5.486e-05\Dipo
le=-0.1925649,0.1426896,-0.9744548\Quadrupole=0.4737753,-0.0143939,-0.
4593814,-0.3543219,0.9670435,1.0378785\PG=CS [SG(C1H1S1Se1),X(H2)]\\@

CH_3SSe^- B3PW91

1\1\GINC-ORC40\FOpt\RB3PW91\6-31G(d)\C1H3S1Se1(1-)\MEISTERP\27-May-201
7\0\\# opt freq b3pw91/6-31g(d)\\Methyl Sulfur Selenide\\-1,1\C,-0.480
7574805,2.0110524422,-0.0062946273\H,-0.4670442246,0.9298604783,-0.189
6585331\H,-0.1371616305,2.5397147961,-0.9085665998\H,-1.5128053299,2.3
062048769,0.2185357864\S,0.6157065351,2.430394918,1.3888246236\Se,-0.3
001547994,1.2405489012,3.0543689646\\Version=EM64L-G16RevA.03\State=1-
A'\HF=-2837.4423149\RMSD=7.512e-09\RMSF=1.743e-04\Dipole=-0.3139804,0.
0312825,-0.9098706\Quadrupole=0.3413181,-0.1797706,-0.1615475,-0.40419
79,1.1265705,1.0324744\PG=CS [SG(C1H1S1Se1),X(H2)]\\@

1\1\GINC-ORC259\FOpt\RB3PW91\6-311G(d)\C1H3S1Se1(1-)\MEISTERP\27-May-2
017\0\\# opt freq b3pw91/6-311g(d)\\Methyl Sulfur Selenide\\-1,1\C,-0.
4840270163,2.0092478751,-0.0085862998\H,-0.467411673,0.9319175357,-0.1
963618857\H,-0.1344086114,2.5403049127,-0.9035033329\H,-1.5128550534,2
.3078437711,0.2117084155\S,0.6124386544,2.4301449531,1.3812954036\Se,-
0.295953229,1.2383173649,3.0726573167\\Version=EM64L-G16RevA.03\State=
1-A'\HF=-2839.6610937\RMSD=9.574e-09\RMSF=4.641e-05\Dipole=-0.3125478,
0.0451554,-0.9529767\Quadrupole=0.226294,-0.2242731,-0.0020208,-0.3794
857,1.0280759,0.8779674\PG=CS [SG(C1H1S1Se1),X(H2)]\\@

1\1\GINC-ORC188\FOpt\RB3PW91\6-311G(d,p)\C1H3S1Se1(1-)\MEISTERP\27-May
-2017\0\\# opt freq b3pw91/6-311g(d,p)\\Methyl Sulfur Selenide\\-1,1\C
,-0.4851172611,2.0089110492,-0.010243714\H,-0.4670233928,0.9312059046,
-0.1963614214\H,-0.1328964449,2.5413423541,-0.9031273156\H,-1.51338525
33,2.3083409675,0.2120673922\S,0.6112553749,2.4293920308,1.3808026306\
Se,-0.29504995,1.2385841062,3.0740720501\\Version=EM64L-G16RevA.03\Sta
te=1-A'\HF=-2839.6655197\RMSD=5.250e-09\RMSF=5.055e-05\Dipole=-0.30779
61,0.0458724,-0.9432209\Quadrupole=0.2433283,-0.1981548,-0.0451734,-0.
3770353,1.0014866,0.8777363\PG=CS [SG(C1H1S1Se1),X(H2)]\\@

1\1\GINC-ORC99\FOpt\RB3PW91\6-311+G(d,p)\C1H3S1Se1(1-)\MEISTERP\27-May
-2017\0\\# opt freq b3pw91/6-311+g(d,p)\\Methyl Sulfur Selenide\\-1,1\
C,-0.4863894309,2.0076279369,-0.0091765293\H,-0.468078068,0.9304554914
,-0.1987796251\H,-0.1301471338,2.5422362806,-0.8990979308\H,-1.5150479
037,2.3083907212,0.2098865009\S,0.609926762,2.4282680392,1.3811886912\
Se,-0.2924811556,1.2407979434,3.0731885074\\Version=EM64L-G16RevA.03\S
tate=1-A'\HF=-2839.6714608\RMSD=6.698e-09\RMSF=4.907e-05\Dipole=-0.218
941,0.1627241,-1.1095804\Quadrupole=0.3056707,-0.175658,-0.1300127,-0.
2701501,0.9667604,0.8573013\PG=CS [SG(C1H1S1Se1),X(H2)]\\@

1\1\GINC-ORC329\FOpt\RB3PW91\6-311G(2d,p)\C1H3S1Se1(1-)\MEISTERP\27-Ma
y-2017\0\\# opt freq b3pw91/6-311g(2d,p)\\Methyl Sulfur Selenide\\-1,1
\C,-0.4860350224,2.0079968152,-0.0095123423\H,-0.4667162483,0.93125907
18,-0.196565569\H,-0.1309766097,2.5417562184,-0.8996043142\H,-1.513297
8023,2.3086832772,0.2119489981\S,0.6062686521,2.4247227942,1.383770718
8\Se,-0.2914598997,1.2433582358,3.0671721219\\Version=EM64L-G16RevA.03
\State=1-A'\HF=-2839.6683231\RMSD=5.845e-09\RMSF=5.262e-05\Dipole=-0.2
988062,0.0380604,-0.893849\Quadrupole=0.295705,-0.1764899,-0.1192151,-
0.3174396,0.9900265,0.8845864\PG=CS [SG(C1H1S1Se1),X(H2)]\\@

1\1\GINC-ORC128\FOpt\RB3PW91\6-311G(df,p)\C1H3S1Se1(1-)\MEISTERP\27-Ma
y-2017\0\\# opt freq b3pw91/6-311g(df,p)\\Methyl Sulfur Selenide\\-1,1
\C,-0.4847328937,2.0085646156,-0.008090895\H,-0.4688128671,0.930898499
2,-0.1975930631\H,-0.1304546712,2.5422025512,-0.8997720888\H,-1.514547
8352,2.3072084982,0.2105910542\S,0.6071652753,2.4247929008,1.385831411
1\Se,-0.290833939,1.2441093477,3.0662431934\\Version=EM64L-G16RevA.03\
State=1-A'\HF=-2839.6714258\RMSD=2.495e-09\RMSF=1.643e-04\Dipole=-0.30
10359,0.0497907,-0.9391135\Quadrupole=0.3000372,-0.1385461,-0.1614911,
-0.4672491,1.0386832,0.988222\PG=CS [SG(C1H1S1Se1),X(H2)]\\@

1\1\GINC-ORC329\FOpt\RB3PW91\6-311+G(2df,p)\C1H3S1Se1(1-)\MEISTERP\27-
May-2017\0\\# opt freq b3pw91/6-311+g(2df,p)\\Methyl Sulfur Selenide\\
-1,1\C,-0.4866588509,2.0063110574,-0.0054265243\H,-0.4690293128,0.9301
253236,-0.1991966834\H,-0.1253806937,2.5435576696,-0.8913421363\H,-1.5
157537604,2.3077375938,0.2093736597\S,0.600507628,2.4199366906,1.38514
91605\Se,-0.2859019375,1.2501080778,3.0586521439\\Version=EM64L-G16Rev
A.03\State=1-A'\HF=-2839.6801174\RMSD=4.818e-09\RMSF=1.422e-04\Dipole=
-0.2078843,0.1510724,-1.0419668\Quadrupole=0.3767862,-0.1137238,-0.263
0623,-0.2838635,0.9620549,0.9201745\PG=CS [SG(C1H1S1Se1),X(H2)]\\@

1\1\GINC-ORC199\FOpt\RB3PW91\6-311++G(3df,3pd)\C1H3S1Se1(1-)\MEISTERP\
27-May-2017\0\\# opt freq b3pw91/6-311++g(3df,3pd)\\Methyl Sulfur Sele
nide\\-1,1\C,-0.4867195686,2.0060120312,-0.0045738252\H,-0.4692595526,
0.9313201167,-0.1993264596\H,-0.1247390573,2.5431450964,-0.8883072062\
H,-1.5147691921,2.3073335566,0.2087697047\S,0.5982552123,2.4188356572,
1.3830911021\Se,-0.2849847691,1.2511299545,3.0575563051\\Version=EM64L
-G16RevA.03\State=1-A'\HF=-2839.6861917\RMSD=3.499e-09\RMSF=1.337e-04\
Dipole=-0.2004217,0.1390865,-0.9824342\Quadrupole=0.4362841,-0.050441,
-0.3858431,-0.3616893,0.9869416,1.0206405\PG=CS [SG(C1H1S1Se1),X(H2)]\
\@

CH_3SSe^- wB97XD

1\1\GINC-ORC226\FOpt\RwB97XD\6-31G(d)\C1H3S1Se1(1-)\MEISTERP\27-May-20
17\0\\# opt freq wb97xd/6-31g(d)\\Methyl Sulfur Selenide\\-1,1\C,-0.48
02837071,2.0109913494,-0.0048748661\H,-0.4658250073,0.9305722722,-0.18
58523264\H,-0.1384051553,2.5381558943,-0.9064961254\H,-1.5107518174,2.
3058186403,0.2220163407\S,0.6157612558,2.4309703848,1.3870244619\Se,-0
.3027124939,1.2412678718,3.0453921408\\Version=EM64L-G16RevA.03\State=
1-A'\HF=-2837.50864\RMSD=5.231e-09\RMSF=1.493e-04\Dipole=-0.3257194,0.
0293883,-0.9335581\Quadrupole=0.2921939,-0.2182001,-0.0739938,-0.44210
95,1.1575833,1.0267105\PG=CS [SG(C1H1S1Se1),X(H2)]\\@

1\1\GINC-ORC117\FOpt\RwB97XD\6-311G(d)\C1H3S1Se1(1-)\MEISTERP\27-May-2
017\0\\# opt freq wb97xd/6-311g(d)\\Methyl Sulfur Selenide\\-1,1\C,-0.
4831621916,2.009425788,-0.0069705723\H,-0.4661275995,0.9322257414,-0.1
921737168\H,-0.1360999018,2.5387544137,-0.9026083308\H,-1.5110465684,2

.3074617896,0.2156918896\S,0.6134890616,2.4313959759,1.3797682801\Se,-
0.2992697254,1.2385127042,3.0635020766\\Version=EM64L-G16RevA.03\State
=1-A'\HF=-2839.7336944\RMSD=8.322e-09\RMSF=1.413e-04\Dipole=-0.3293725
,0.039141,-0.9758009\Quadrupole=0.1688693,-0.2735486,0.1046793,-0.4179
39,1.0680305,0.8700337\PG=CS [SG(C1H1S1Se1),X(H2)]\\@

1\1\GINC-ORC164\FOpt\RwB97XD\6-311G(d,p)\C1H3S1Se1(1-)\MEISTERP\27-May
-2017\0\\# opt freq wb97xd/6-311g(d,p)\\Methyl Sulfur Selenide\\-1,1\C
,-0.4840066732,2.0091828613,-0.0083149695\H,-0.4658193689,0.9316374511
,-0.1920414657\H,-0.1348418119,2.5396250625,-0.9023208477\H,-1.5114530
953,2.3078142042,0.2161031337\S,0.6125078186,2.4308000453,1.3792637656
\Se,-0.2986037955,1.2387167884,3.0645200072\\Version=EM64L-G16RevA.03\
State=1-A'\HF=-2839.7379519\RMSD=7.394e-09\RMSF=1.526e-04\Dipole=-0.32
40844,0.0398323,-0.9645841\Quadrupole=0.1876013,-0.2467898,0.0591885,-
0.4139852,1.0415099,0.870457\PG=CS [SG(C1H1S1Se1),X(H2)]\\@

1\1\GINC-ORC259\FOpt\RwB97XD\6-311+G(d,p)\C1H3S1Se1(1-)\MEISTERP\27-Ma
y-2017\0\\# opt freq wb97xd/6-311+g(d,p)\\Methyl Sulfur Selenide\\-1,1
\C,-0.485303139,2.0078743717,-0.0072244624\H,-0.4669336429,0.930923271
7,-0.1949096385\H,-0.1319455893,2.5405816613,-0.8981264053\H,-1.513222
736,2.3079625642,0.2134907717\S,0.6108096128,2.4294111311,1.3795962216
\Se,-0.2956214321,1.2410234129,3.0643831358\\Version=EM64L-G16RevA.03\
State=1-A'\HF=-2839.7441207\RMSD=6.671e-09\RMSF=1.488e-04\Dipole=-0.23
46589,0.1618121,-1.1467732\Quadrupole=0.2214961,-0.2611028,0.0396067,-
0.2884572,1.0212184,0.8242507\PG=CS [SG(C1H1S1Se1),X(H2)]\\@

1\1\GINC-ORC199\FOpt\RwB97XD\6-311G(2d,p)\C1H3S1Se1(1-)\MEISTERP\27-Ma
y-2017\0\\# opt freq wb97xd/6-311g(2d,p)\\Methyl Sulfur Selenide\\-1,1
\C,-0.4848346526,2.0083389013,-0.0075905329\H,-0.4654984557,0.93180218
17,-0.1922323677\H,-0.1332249478,2.5398900423,-0.8990720272\H,-1.51125
60318,2.3081419354,0.2159605742\S,0.607917645,2.4264740999,1.382090276
2\Se,-0.2953204822,1.2431292522,3.0580537032\\Version=EM64L-G16RevA.03
\State=1-A'\HF=-2839.7407696\RMSD=8.905e-09\RMSF=1.579e-04\Dipole=-0.3
119309,0.0354087,-0.9185326\Quadrupole=0.236463,-0.2294634,-0.0069996,
-0.3477264,1.0281372,0.8701027\PG=CS [SG(C1H1S1Se1),X(H2)]\\@

1\1\GINC-ORC137\FOpt\RwB97XD\6-311G(df,p)\C1H3S1Se1(1-)\MEISTERP\27-Ma
y-2017\0\\# opt freq wb97xd/6-311g(df,p)\\Methyl Sulfur Selenide\\-1,1
\C,-0.4835693694,2.0090283552,-0.0066736708\H,-0.4670375439,0.93166150
99,-0.1927183623\H,-0.1331013011,2.5401485311,-0.8996268236\H,-1.51198
78824,2.3069388442,0.2151594887\S,0.6082988531,2.4261738022,1.38407944
07\Se,-0.2948196842,1.24382537,3.0569895463\\Version=EM64L-G16RevA.03\
State=1-A'\HF=-2839.7440242\RMSD=5.194e-09\RMSF=1.416e-04\Dipole=-0.31
78386,0.0436335,-0.9614\Quadrupole=0.2421295,-0.1879268,-0.0542027,-0.
507091,1.0792024,0.9808762\PG=CS [SG(C1H1S1Se1),X(H2)]\\@

1\1\GINC-ORC164\FOpt\RwB97XD\6-311+G(2df,p)\C1H3S1Se1(1-)\MEISTERP\27-
May-2017\0\\# opt freq wb97xd/6-311+g(2df,p)\\Methyl Sulfur Selenide\\
-1,1\C,-0.4854808613,2.0068170762,-0.004114797\H,-0.4674455063,0.93105
86202,-0.195029771\H,-0.1281750876,2.5414434653,-0.8913725023\H,-1.513
2886982,2.3075110544,0.2131965896\S,0.601695446,2.4212551703,1.3837466
217\Se,-0.2895222224,1.2496910263,3.0507834735\\Version=EM64L-G16RevA.
03\State=1-A'\HF=-2839.75286\RMSD=9.731e-09\RMSF=1.220e-04\Dipole=-0.2
210273,0.1534324,-1.0835958\Quadrupole=0.2880901,-0.1999349,-0.0881552
,-0.3039433,1.0128807,0.8825674\PG=CS [SG(C1H1S1Se1),X(H2)]\\@

1\1\GINC-ORC117\FOpt\RwB97XD\6-311++G(3df,3pd)\C1H3S1Se1(1-)\MEISTERP\
27-May-2017\0\\# opt freq wb97xd/6-311++g(3df,3pd)\\Methyl Sulfur Sele
nide\\-1,1\C,-0.4855780428,2.0065403097,-0.0034305707\H,-0.4678219326,
0.9325791645,-0.195488013\H,-0.1275968698,2.540960817,-0.8882637671\H,
-1.5121405928,2.3070251359,0.2121432736\S,0.5992601444,2.4200254582,1.
3816539019\Se,-0.2883396357,1.2506455274,3.0505947911\\Version=EM64L-G
16RevA.03\State=1-A'\HF=-2839.7591462\RMSD=9.972e-09\RMSF=1.106e-04\Di
pole=-0.2138836,0.1451044,-1.0372138\Quadrupole=0.3461561,-0.1421271,-
0.204029,-0.3807561,1.0452162,0.9849623\PG=CS [SG(C1H1S1Se1),X(H2)]\\@

CH_3SSe^- M062X

1\1\GINC-ORC353\FOpt\RM062X\6-31G(d)\C1H3S1Se1(1-)\MEISTERP\27-May-201
7\0\\# opt freq m062x/6-31g(d)\\Methyl Sulfur Selenide\\-1,1\C,-0.4778
686513,2.0119872009,-0.0020454863\H,-0.4613684908,0.9310024518,-0.1715
744121\H,-0.1448418429,2.5339575619,-0.9088305189\H,-1.5051285358,2.30
471322,0.2358388289\S,0.6228028873,2.4375282023,1.3829529918\Se,-0.315
8122922,1.2385877766,3.020868219\\Version=EM64L-G16RevA.03\State=1-A'\
HF=-2837.5066584\RMSD=5.097e-09\RMSF=1.951e-04\Dipole=-0.2895184,0.056
7672,-0.9331297\Quadrupole=0.340222,-0.1720074,-0.1682146,-0.4393243,1
.137107,1.0546236\PG=CS [SG(C1H1S1Se1),X(H2)]\\@

1\1\GINC-ORC389\FOpt\RM062X\6-311G(d)\C1H3S1Se1(1-)\MEISTERP\27-May-20
17\0\\# opt freq m062x/6-311g(d)\\Methyl Sulfur Selenide\\-1,1\C,-0.48
14805451,2.0101345179,-0.0050520154\H,-0.4618853881,0.9327655256,-0.17
86803099\H,-0.1418782787,2.5348812154,-0.9043524682\H,-1.5056013023,2.
3064182126,0.2287157054\S,0.6199640636,2.4374461925,1.3759566728\Se,-0
.3113354757,1.2361307489,3.0406220385\\Version=EM64L-G16RevA.03\State=
1-A'\HF=-2839.6905557\RMSD=3.372e-09\RMSF=1.722e-04\Dipole=-0.303535,0
.0602123,-0.9806553\Quadrupole=0.1882468,-0.2518925,0.0636457,-0.42067
11,1.0570409,0.881939\PG=CS [SG(C1H1S1Se1),X(H2)]\\@

1\1\GINC-ORC353\FOpt\RM062X\6-311G(d,p)\C1H3S1Se1(1-)\MEISTERP\27-May-
2017\0\\# opt freq m062x/6-311g(d,p)\\Methyl Sulfur Selenide\\-1,1\C,-
0.4823328268,2.0099081263,-0.0064721485\H,-0.4613575574,0.9321438796,-
0.1779896777\H,-0.140751674,2.5357153952,-0.9042788741\H,-1.5058198087
,2.3067788338,0.2296976571\S,0.6191521585,2.4370470995,1.3752222937\Se
,-0.3111072177,1.2361830783,3.0410303737\\Version=EM64L-G16RevA.03\Sta
te=1-A'\HF=-2839.6938072\RMSD=7.672e-09\RMSF=1.872e-04\Dipole=-0.30017
38,0.0606548,-0.9735362\Quadrupole=0.2011193,-0.2308202,0.0297008,-0.4
200216,1.0353603,0.8816071\PG=CS [SG(C1H1S1Se1),X(H2)]\\@

1\1\GINC-ORC389\FOpt\RM062X\6-311+G(d,p)\C1H3S1Se1(1-)\MEISTERP\27-May
-2017\0\\# opt freq m062x/6-311+g(d,p)\\Methyl Sulfur Selenide\\-1,1\C
,-0.4837458415,2.0085699059,-0.0055799843\H,-0.4626071019,0.9313766916
,-0.1813918654\H,-0.137607577,2.5368910178,-0.900187899\H,-1.507823826
8,2.3070046216,0.2265899648\S,0.6171451676,2.4353199758,1.3759040378\S
e,-0.3075777511,1.2386142001,3.0418753583\\Version=EM64L-G16RevA.03\St
ate=1-A'\HF=-2839.699409\RMSD=7.074e-09\RMSF=1.837e-04\Dipole=-0.21408
91,0.1709255,-1.1248035\Quadrupole=0.258437,-0.2120268,-0.0464102,-0.3
175998,1.0038244,0.8608902\PG=CS [SG(C1H1S1Se1),X(H2)]\\@

1\1\GINC-ORC389\FOpt\RM062X\6-311G(2d,p)\C1H3S1Se1(1-)\MEISTERP\27-May
-2017\0\\# opt freq m062x/6-311g(2d,p)\\Methyl Sulfur Selenide\\-1,1\C
,-0.4834627652,2.0089306744,-0.0060712001\H,-0.4609939492,0.9322515326
,-0.1783760024\H,-0.1386596358,2.5362383505,-0.9006825342\H,-1.5057146
273,2.3072266068,0.2294122048\S,0.6138440319,2.4321463019,1.3781477459

\Se,-0.3072299807,1.2409829464,3.0347794096\\Version=EM64L-G16RevA.03\State=1-A'\HF=-2839.6968567\RMSD=6.069e-09\RMSF=1.935e-04\Dipole=-0.2900028,0.0510447,-0.9150758\Quadrupole=0.2427405,-0.2111578,-0.0315827,-0.3502619,1.0046991,0.8651498\PG=CS [SG(C1H1S1Se1),X(H2)]\\@

1\1\GINC-ORC199\FOpt\RM062X\6-311G(df,p)\C1H3S1Se1(1-)\MEISTERP\27-May-2017\0\\# opt freq m062x/6-311g(df,p)\\Methyl Sulfur Selenide\\-1,1\C,-0.4817178754,2.0098737544,-0.0047807975\H,-0.4629652004,0.9321598974,-0.1795409464\H,-0.1388303461,2.536404937,-0.9016815745\H,-1.5067180009,2.3058611311,0.2278694669\S,0.6146133949,2.432015035,1.3805613961\Se,-0.3065989008,1.2414616574,3.0347820743\\Version=EM64L-G16RevA.03\State=1-A'\HF=-2839.7004529\RMSD=7.219e-09\RMSF=1.733e-04\Dipole=-0.2974908,0.0594722,-0.9626749\Quadrupole=0.2569461,-0.1669443,-0.0900018,-0.5337327,1.081127,1.0075264\PG=CS [SG(C1H1S1Se1),X(H2)]\\@

1\1\GINC-ORC226\FOpt\RM062X\6-311+G(2df,p)\C1H3S1Se1(1-)\MEISTERP\27-May-2017\0\\# opt freq m062x/6-311+g(2df,p)\\Methyl Sulfur Selenide\\-1,1\C,-0.4839992466,2.0075462667,-0.0027776942\H,-0.4634409814,0.9314491376,-0.1822899762\H,-0.1334054103,2.5380910531,-0.8934685488\H,-1.5082564857,2.3065490142,0.2255352447\S,0.6074559276,2.4265439081,1.380672015\Se,-0.3005707262,1.2475970336,3.0295385905\\Version=EM64L-G16RevA.03\State=1-A'\HF=-2839.7088189\RMSD=9.376e-09\RMSF=1.589e-04\Dipole=-0.2055122,0.1534338,-1.043852\Quadrupole=0.3215016,-0.1489252,-0.1725764,-0.3431646,0.9945028,0.9209872\PG=CS [SG(C1H1S1Se1),X(H2)]\\@

1\1\GINC-ORC389\FOpt\RM062X\6-311++G(3df,3pd)\C1H3S1Se1(1-)\MEISTERP\27-May-2017\0\\# opt freq m062x/6-311++g(3df,3pd)\\Methyl Sulfur Selenide\\-1,1\C,-0.4841658271,2.0072334137,-0.0021495864\H,-0.463870009,0.9330833649,-0.1829250085\H,-0.1330702528,2.5374539689,-0.8904617898\H,-1.5070686165,2.3060552155,0.2242690854\S,0.605512212,2.4256634247,1.3786611743\Se,-0.2995544318,1.2482870256,3.0298157495\\Version=EM64L-G16RevA.03\State=1-A'\HF=-2839.7152073\RMSD=9.825e-09\RMSF=1.508e-04\Dipole=-0.2010074,0.1400055,-0.9870337\Quadrupole=0.3759174,-0.0982256,-0.2776918,-0.4098425,1.0272861,1.0154412\PG=CS [SG(C1H1S1Se1),X(H2)]\\@

CH_3SSe^- M08HX

1\1\GINC-ORC199\FOpt\RM08HX\6-31G(d)\C1H3S1Se1(1-)\MEISTERP\27-May-2017\0\\# opt freq m08hx/6-31g(d)\\Methyl Sulfur Selenide\\-1,1\C,-0.474941871,2.0132821257,0.0010864795\H,-0.4597509725,0.9270767196,-0.1669538815\H,-0.1455784131,2.5346683138,-0.9131140568\H,-1.5069644121,2.3053325607,0.2418073309\S,0.6242519648,2.4378401033,1.3856137482\Se,-0.3192332248,1.2395765896,3.0087699969\\Version=EM64L-G16RevA.03\State=1-A'\HF=-2837.5401671\RMSD=4.405e-09\RMSF=6.885e-05\Dipole=-0.258742,0.0890705,-0.9632031\Quadrupole=0.3671787,-0.1525308,-0.2146479,-0.414965,1.1204105,1.056128\PG=CS [SG(C1H1S1Se1),X(H2)]\\@

1\1\GINC-ORC99\FOpt\RM08HX\6-311G(d)\C1H3S1Se1(1-)\MEISTERP\27-May-2017\0\\# opt freq m08hx/6-311g(d)\\Methyl Sulfur Selenide\\-1,1\C,-0.4785103599,2.0110874146,-0.000655604\H,-0.46071815,0.9281878451,-0.1730565819\H,-0.1419945235,2.5357540081,-0.9075931492\H,-1.5078986774,2.3064003698,0.2356917838\S,0.6228215434,2.4390836406,1.3777561803\Se,-0.31591676,1.2372631354,3.0250669892\\Version=EM64L-G16RevA.03\State=1-A'\HF=-2839.7080436\RMSD=3.918e-09\RMSF=1.959e-04\Dipole=-0.2587086,0.1049631,-1.0167037\Quadrupole=0.2309695,-0.2167931,-0.0141763,-0.3758745,1.0179685,0.8750399\PG=CS [SG(C1H1S1Se1),X(H2)]\\@

1\1\GINC-ORC353\FOpt\RM08HX\6-311G(d,p)\C1H3S1Se1(1-)\MEISTERP\27-May-2017\0\\# opt freq m08hx/6-311g(d,p)\\Methyl Sulfur Selenide\\-1,1\C,-0.4794241663,2.0108307326,-0.0021312259\H,-0.4603436836,0.9278007873,-0.1725865141\H,-0.1408423798,2.5365267492,-0.9072469682\H,-1.5080098351,2.3066524503,0.2363514064\S,0.621856503,2.4385542805,1.3770687136\Se,-0.3154533642,1.2374114132,3.0257542107\\Version=EM64L-G16RevA.03\State=1-A'\HF=-2839.7116709\RMSD=6.828e-09\RMSF=2.092e-04\Dipole=-0.2542707,0.1055625,-1.0073557\Quadrupole=0.2470909,-0.1950486,-0.0520423,-0.3723348,0.9974451,0.8764517\PG=CS [SG(C1H1S1Se1),X(H2)]\\@

1\1\GINC-ORC199\FOpt\RM08HX\6-311+G(d,p)\C1H3S1Se1(1-)\MEISTERP\27-May-2017\0\\# opt freq m08hx/6-311+g(d,p)\\Methyl Sulfur Selenide\\-1,1\C,-0.4809800228,2.00935789,-0.0011510935\H,-0.4617895951,0.9269759123,-0.1761862944\H,-0.1375088585,2.5377791943,-0.9029297325\H,-1.5101801858,2.3067810224,0.2330343981\S,0.6200703758,2.4369380857,1.3779422641\Se,-0.3118286405,1.2399443078,3.0265000806\\Version=EM64L-G16RevA.03\State=1-A'\HF=-2839.7177451\RMSD=4.218e-09\RMSF=2.067e-04\Dipole=-0.1607523,0.2163278,-1.1412459\Quadrupole=0.32946,-0.1353818,-0.1940782,-0.2702587,0.9251901,0.8566263\PG=CS [SG(C1H1S1Se1),X(H2)]\\@

1\1\GINC-ORC389\FOpt\RM08HX\6-311G(2d,p)\C1H3S1Se1(1-)\MEISTERP\27-May-2017\0\\# opt freq m08hx/6-311g(2d,p)\\Methyl Sulfur Selenide\\-1,1\C,-0.4806592388,2.0097767123,-0.0017414503\H,-0.4604153758,0.9281448453,-0.1743694019\H,-0.1383766016,2.5371919083,-0.9031726173\H,-1.5082003723,2.3071529223,0.2346149077\S,0.6158248698,2.4330506549,1.3801732078\Se,-0.3103902092,1.2424593687,3.0217049758\\Version=EM64L-G16RevA.03\State=1-A'\HF=-2839.7149687\RMSD=5.353e-09\RMSF=2.108e-04\Dipole=-0.253271,0.0848624,-0.934998\Quadrupole=0.2815802,-0.1786415,-0.1029387,-0.306964,0.9660922,0.8560662\PG=CS [SG(C1H1S1Se1),X(H2)]\\@

1\1\GINC-ORC164\FOpt\RM08HX\6-311G(df,p)\C1H3S1Se1(1-)\MEISTERP\27-May-2017\0\\# opt freq m08hx/6-311g(df,p)\\Methyl Sulfur Selenide\\-1,1\C,-0.4787280786,2.010842918,-0.0003889916\H,-0.4619307684,0.9278455585,-0.1742853283\H,-0.1391797101,2.5370800335,-0.9048528987\H,-1.5089154163,2.3058002827,0.2343865793\S,0.6176712292,2.433815979,1.3823229225\Se,-0.3111341839,1.2423916414,3.0200273345\\Version=EM64L-G16RevA.03\State=1-A'\HF=-2839.7189437\RMSD=4.421e-09\RMSF=1.995e-04\Dipole=-0.2564428,0.0972378,-0.9848511\Quadrupole=0.3032134,-0.1326203,-0.1705931,-0.4908158,1.0499546,1.0083216\PG=CS [SG(C1H1S1Se1),X(H2)]\\@

1\1\GINC-ORC353\FOpt\RM08HX\6-311+G(2df,p)\C1H3S1Se1(1-)\MEISTERP\27-May-2017\0\\# opt freq m08hx/6-311+g(2df,p)\\Methyl Sulfur Selenide\\-1,1\C,-0.4812948159,2.008327832,0.0015155685\H,-0.4628134537,0.9272377241,-0.1780540578\H,-0.1331669278,2.5390451765,-0.8960746768\H,-1.5107607232,2.3064593715,0.2309935922\S,0.6100349395,2.4280154175,1.3823176199\Se,-0.3042159476,1.2486908911,3.0165115711\\Version=EM64L-G16RevA.03\State=1-A'\HF=-2839.7280349\RMSD=2.659e-09\RMSF=1.778e-04\Dipole=-0.1641216,0.1819763,-1.0340517\Quadrupole=0.3828479,-0.0769557,-0.3058922,-0.300067,0.9141167,0.910125\PG=CS [SG(C1H1S1Se1),X(H2)]\\@

1\1\GINC-ORC389\FOpt\RM08HX\6-311++G(3df,3pd)\C1H3S1Se1(1-)\MEISTERP\27-May-2017\0\\# opt freq m08hx/6-311++g(3df,3pd)\\Methyl Sulfur Selenide\\-1,1\C,-0.4818458801,2.0078766359,0.0016251232\H,-0.4625732397,0.9292237243,-0.1769534237\H,-0.1333616709,2.5379125032,-0.8927544599\H,-1.5085768901,2.3058873407,0.2313355689\S,0.6086018701,2.4276856044,1.3797582709\Se,-0.3044611148,1.2491906044,3.0141985454\\Version=EM64L-G16RevA.03\State=1-A'\HF=-2839.7347469\RMSD=3.614e-09\RMSF=1.819e-04\Dip

ole=-0.1600644,0.1660064,-0.9698104\Quadrupole=0.4114973,-0.0411335,-0
.3703638,-0.3790835,0.9475679,0.9901295\PG=CS [SG(C1H1S1Se1),X(H2)]\\@

CH_3SSe^- QCISD

1\1\GINC-ORC269\FOpt\RQCISD-FC\6-311+G(2df,p)\C1H3S1Se1(1-)\MEISTERP\2
0-Aug-2017\0\\# opt freq=noraman qcisd/6-311+g(2df,p)\\Methyl Sulfur S
elenide\\-1,1\C,-0.483707876,2.0078236313,-0.0029664408\H,-0.465705364
4,0.930095022,-0.1899548389\H,-0.1339802787,2.5389918999,-0.8979787793
\H,-1.5122609375,2.3074850335,0.218549587\S,0.61266763,2.4300221994,1.
3822959204\Se,-0.2992300963,1.2433586272,3.0472641815\\Version=EM64L-G
09RevE.01\State=1-A'\HF=-2837.012746\MP2=-2837.5138327\MP3=-2837.55206
03\MP4D=-2837.5654648\MP4DQ=-2837.5502498\MP4SDQ=-2837.5538362\QCISD=-
2837.5536578\RMSD=6.817e-09\RMSF=1.485e-04\Dipole=-0.2028917,0.1628075
,-1.0687445\PG=CS [SG(C1H1S1Se1),X(H2)]\\@

CH_2CHSeH B3LYP

1\1\GINC-ORC335\FOpt\RB3PW91\6-31G(d)\C2H4Se1\MEISTERP\28-Jul-2017\0\\
opt=readfc freq b3pw91/6-31g(d)\\CH2CHSeH\\0,1\C,2.1242531157,-0.289
1582958,-0.0489168221\H,2.0069205082,-1.3558484145,-0.2218743139\H,3.1
395962221,0.0845150153,0.0597324237\C,1.0825313197,0.5362065644,0.0388
714458\H,1.214521603,1.6019206406,0.2066890875\Se,-0.7158206246,-0.084
8720627,0.0226321528\H,-1.2563021441,1.0804885527,-0.7242399738\\Versi
on=EM64L-G09RevE.01\State=1-A\HF=-2477.9101916\RMSD=9.781e-09\RMSF=9.0
53e-06\Dipole=0.2696231,0.3123007,-0.0973968\Quadrupole=1.5785083,1.01
29886,-2.5914969,-0.5197299,0.8812772,-0.7053118\PG=C01 [X(C2H4Se1)]\\
@

1\1\GINC-ORC324\FOpt\RB3PW91\6-311G(d)\C2H4Se1\MEISTERP\28-Jul-2017\0\
\# opt=readfc freq b3pw91/6-311g(d)\\CH2CHSeH\\0,1\C,2.1297476453,-0.2
939377878,-0.0412316539\H,2.0327044569,-1.3641760425,-0.1969431425\H,3
.1365142791,0.0973557331,0.0730600369\C,1.0820539956,0.5202513795,0.01
64144828\H,1.1983788066,1.5888055422,0.1688360857\Se,-0.7245456497,-0.
1004575913,-0.0272842114\H,-1.2591535338,1.1254107668,-0.6599575977\\V
ersion=EM64L-G09RevE.01\State=1-A\HF=-2480.0999628\RMSD=5.175e-09\RMSF
=9.505e-06\Dipole=0.2305503,0.3222228,-0.0744307\Quadrupole=1.6514084,
1.3281549,-2.9795633,-0.5735755,0.8433848,-0.6508474\PG=C01 [X(C2H4Se1
)]\\@

1\1\GINC-ORC384\FOpt\RB3PW91\6-311G(d,p)\C2H4Se1\MEISTERP\28-Jul-2017\
0\\# opt=readfc freq b3pw91/6-311g(d,p)\\CH2CHSeH\\0,1\C,2.1369992997,
-0.2992735059,-0.0284741542\H,2.0529085559,-1.3736771974,-0.1556700098
\H,3.1351662536,0.1069922198,0.0978520147\C,1.0815078945,0.5066912796,
-0.0179960288\H,1.1823991958,1.5791797781,0.1074052144\Se,-0.718968583
6,-0.1205276865,-0.1269803391\H,-1.274312616,1.1738671125,-0.543242697
2\\Version=EM64L-G09RevE.01\State=1-A\HF=-2480.1087589\RMSD=2.763e-09\
RMSF=7.719e-06\Dipole=0.1839285,0.3459236,-0.0375692\Quadrupole=1.5798
471,1.5603423,-3.1401894,-0.6794511,0.7524674,-0.3963565\PG=C01 [X(C2H
4Se1)]\\@

1\1\GINC-ORC383\FOpt\RB3PW91\6-311+G(d,p)\C2H4Se1\MEISTERP\28-Jul-2017
\0\\# opt=readfc freq b3pw91/6-311+g(d,p)\\CH2CHSeH\\0,1\C,2.138959899
3,-0.3004180391,-0.0214815886\H,2.0613178149,-1.3767708799,-0.13681106
06\H,3.1346409856,0.111939849,0.105178727\C,1.0797170786,0.5025273494,
-0.0281105207\H,1.1779938484,1.5768216667,0.0841673447\Se,-0.718196906
8,-0.1254870674,-0.1571045571\H,-1.2787327201,1.1846391214,-0.51294434

49\\Version=EM64L-G09RevE.01\State=1-A\HF=-2480.1100444\RMSD=5.180e-09\RMSF=7.207e-05\Dipole=0.1692517,0.3540707,-0.0290113\Quadrupole=1.5289424,1.7390871,-3.2680295,-0.7144118,0.7356635,-0.3329417\PG=C01 [X(C2H4Se1)]\\@

1\1\GINC-ORC383\FOpt\RB3PW91\6-311G(2d,p)\C2H4Se1\MEISTERP\28-Jul-2017\0\\# opt=readfc freq b3pw91/6-311g(2d,p)\\CH2CHSeH\\0,1\C,2.1346818422,-0.29949921,-0.0277728168\H,2.0507246571,-1.3735564314,-0.1529096891\H,3.1320962337,0.1064209277,0.100390021\C,1.082239385,0.5042831667,-0.0214847846\H,1.1849104081,1.57580422,0.1028582862\Se,-0.7154868348,-0.1196762221,-0.1380632571\H,-1.2734656915,1.179475549,-0.5301237595\\Version=EM64L-G09RevE.01\State=1-A\HF=-2480.1095165\RMSD=5.271e-09\RMSF=1.607e-05\Dipole=0.1981415,0.3178518,-0.0269638\Quadrupole=1.4915842,1.604694,-3.0962781,-0.6281824,0.7068348,-0.3651168\PG=C01 [X(C2H4Se1)]\\@

1\1\GINC-ORC382\FOpt\RB3PW91\6-311G(df,p)\C2H4Se1\MEISTERP\28-Jul-2017\0\\# opt=readfc freq b3pw91/6-311g(df,p)\\CH2CHSeH\\0,1\C,2.1348603556,-0.3017276167,-0.0258513121\H,2.0528803513,-1.3768654245,-0.1471471327\H,3.132620334,0.1043849987,0.101854401\C,1.080523655,0.5024397785,-0.0247047991\H,1.1822628927,1.5753204545,0.0948883377\Se,-0.7143881395,-0.1188850256,-0.1478557405\H,-1.2730594491,1.1885848352,-0.5182897543\\Version=EM64L-G09RevE.01\State=1-A\HF=-2480.1141025\RMSD=9.042e-09\RMSF=3.670e-05\Dipole=0.1807172,0.3502638,-0.0289001\Quadrupole=1.475981,1.5808784,-3.0568594,-0.6961539,0.7071033,-0.3201105\PG=C01 [X(C2H4Se1)]\\@

1\1\GINC-ORC382\FOpt\RB3PW91\6-311+G(2df,p)\C2H4Se1\MEISTERP\28-Jul-2017\0\\# opt=readfc freq b3pw91/6-311+g(2df,p)\\CH2CHSeH\\0,1\C,2.1379260762,-0.3029318662,-0.001744687\H,2.0656840865,-1.3827673323,-0.0748068173\H,3.1281314948,0.1148718497,0.1409646399\C,1.0821419462,0.4946152954,-0.0797343881\H,1.1779351555,1.5713041826,-0.0040411941\Se,-0.6958441476,-0.1291696828,-0.3288172634\H,-1.3002746115,1.2073295536,-0.31892629\\Version=EM64L-G09RevE.01\State=1-A\HF=-2480.1148271\RMSD=8.100e-09\RMSF=8.720e-06\Dipole=0.1571686,0.3335509,0.0389382\Quadrupole=1.408481,1.869726,-3.2782069,-0.7531079,0.5196841,0.225849\PG=C01 [X(C2H4Se1)]\\@

1\1\GINC-ORC381\FOpt\RB3PW91\6-311++G(3df,3pd)\C2H4Se1\MEISTERP\28-Jul-2017\0\\# opt=readfc freq b3pw91/6-311++g(3df,3pd)\\CH2CHSeH\\0,1\C,2.1381058874,-0.3037555841,-0.002142973\H,2.065825929,-1.3820467625,-0.076657903\H,3.1265541238,0.1144112262,0.1411123735\C,1.0824290269,0.4942176042,-0.079054016\H,1.1778121852,1.5695284408,-0.0019764692\Se,-0.6961290898,-0.1280636976,-0.3286324498\H,-1.2988980625,1.208960773,-0.3197545625\\Version=EM64L-G09RevE.01\State=1-A\HF=-2480.1206978\RMSD=8.879e-09\RMSF=9.107e-06\Dipole=0.1548893,0.317287,0.0376648\Quadrupole=1.3618071,1.7790534,-3.1408605,-0.7505632,0.4997546,0.2139682\PG=C01 [X(C2H4Se1)]\\@

CH_2CHSeH B3PW91

1\1\GINC-ORC56\FOpt\RB3PW91\6-31G(d)\C2H4Se1\MEISTERP\28-Jul-2017\0\\# opt=readfc freq b3pw91/6-31g(d)\\CH2CHSeH\\0,1\C,2.124198175,-0.2891143697,-0.0489778514\H,2.0068191539,-1.3557909906,-0.2219964338\H,3.1395749567,0.0844599255,0.059751358\C,1.0825192199,0.5362799592,0.0389081925\H,1.2146240661,1.6019651119,0.2068103006\Se,-0.7158263439,-0.0848074793,0.0228421512\H,-1.2562092276,1.0802598431,-0.7244437171\\Version

=EM64L-G09RevE.01\State=1-A\HF=-2477.9101916\RMSD=4.029e-09\RMSF=4.655
e-06\Dipole=0.2696949,0.3122059,-0.097446\Quadrupole=1.5786372,1.01200
78,-2.5906449,-0.5194259,0.8815812,-0.7055551\PG=C01 [X(C2H4Se1)]\\@

1\1\GINC-ORC46\FOpt\RB3PW91\6-311G(d)\C2H4Se1\MEISTERP\28-Jul-2017\0\\
opt freq b3pw91/6-311g(d)\\CH2CHSeH\\0,1\C,2.1326385488,-0.283649070
3,-0.0422929631\H,2.0286102211,-1.3537213429,-0.1945712594\H,3.1430113
725,0.1045822588,0.0482729317\C,1.0891242143,0.5338839041,0.0387919421
\H,1.2122750397,1.6022150901,0.1874307871\Se,-0.7199442704,-0.08135140
53,0.0382899156\H,-1.264830126,1.1434945655,-0.587697354\\Version=EM64
L-G09RevE.01\State=1-A\HF=-2480.0999629\RMSD=7.007e-09\RMSF=1.037e-05\
Dipole=0.2305604,0.3209027,-0.0808288\Quadrupole=1.6848806,1.3227211,-
3.0076017,-0.5862961,0.7374171,-0.6499715\PG=C01 [X(C2H4Se1)]\\@

1\1\GINC-ORC36\FOpt\RB3PW91\6-311G(d,p)\C2H4Se1\MEISTERP\28-Jul-2017\0
\\# opt=readfc freq b3pw91/6-311g(d,p)\\CH2CHSeH\\0,1\C,2.1369733638,-
0.2993204718,-0.0279598146\H,2.0528674231,-1.3738556124,-0.1540606791\
H,3.1351537322,0.1070372901,0.0979597237\C,1.081510351,0.5067007252,-0
.0183207094\H,1.1824669412,1.5793094835,0.1059912847\Se,-0.7190118491,
-0.1204414009,-0.1268126415\H,-1.2742599622,1.1738219862,-0.5439031639
\\Version=EM64L-G09RevE.01\State=1-A\HF=-2480.1087589\RMSD=3.324e-09\R
MSF=1.266e-05\Dipole=0.1838893,0.3458879,-0.0378266\Quadrupole=1.57959
14,1.5603084,-3.1398999,-0.678915,0.7527504,-0.4004333\PG=C01 [X(C2H4S
e1)]\\@

1\1\GINC-ORC19\FOpt\RB3PW91\6-311+G(d,p)\C2H4Se1\MEISTERP\20-Aug-2017\
0\\# opt freq=noraman b3pw91/6-311+g(d,p) guess=read\\CH2CHSeH\\0,1\C,
-2.1449260532,-0.3136379979,0.0211897881\H,-2.070507595,-1.38977566,0.
1407791878\H,-3.1374836114,0.09986677,-0.1248055967\C,-1.0842106684,0.
4872887363,0.043824593\H,-1.1774233626,1.5614943857,-0.0738720143\Se,0
.7091289353,-0.1479238912,0.197980343\H,1.2641393552,1.151549657,0.598
8306992\\Version=EM64L-G09RevE.01\State=1-A\HF=-2480.1100461\RMSD=4.40
2e-09\RMSF=6.287e-06\Dipole=-0.1730917,0.3519457,0.0310189\Quadrupole=
1.5051882,1.6985327,-3.2037209,0.6933436,0.8520779,0.3925362\PG=C01 [X
(C2H4Se1)]\\@

1\1\GINC-ORC20\FOpt\RB3PW91\6-311G(2d,p)\C2H4Se1\MEISTERP\20-Aug-2017\
0\\# opt freq=noraman b3pw91/6-311g(2d,p) guess=read\\CH2CHSeH\\0,1\C,
-2.1336755919,-0.2815593205,0.0276997396\H,-2.0385095464,-1.356976126,
0.1311921337\H,-3.1382292006,0.1210751249,-0.041624809\C,-1.0865549764
,0.5276628313,-0.0212641413\H,-1.2002992115,1.6004269647,-0.1232363915
\Se,0.7174018588,-0.0890611005,-0.0101426429\H,1.2896276681,1.20533862
6,0.3773051115\\Version=EM64L-G09RevE.01\State=1-A\HF=-2480.1095165\RM
SD=4.057e-09\RMSF=1.123e-05\Dipole=-0.1983182,0.316086,0.0429996\Quadr
upole=1.548297,1.5933007,-3.1415978,0.6373279,0.4747478,0.4161667\PG=C
01 [X(C2H4Se1)]\\@

1\1\GINC-ORC341\FOpt\RB3PW91\6-311G(df,p)\C2H4Se1\MEISTERP\28-Jul-2017
\0\\# opt=readfc freq b3pw91/6-311g(df,p)\\CH2CHSeH\\0,1\C,2.135007152
6,-0.3013058214,-0.0250981491\H,2.053231225,-1.37657333,-0.1454934238\
H,3.1327148935,0.1050874118,0.1025222628\C,1.080476669,0.5026694415,-0
.0249038224\H,1.1821086004,1.575725372,0.0936264497\Se,-0.7142458038,-
0.1195691965,-0.1474785342\H,-1.2735927366,1.1872181226,-0.520280783\\
Version=EM64L-G09RevE.01\State=1-A\HF=-2480.1141025\RMSD=8.055e-09\RMS
F=5.135e-06\Dipole=0.1807184,0.35017,-0.0293652\Quadrupole=1.4768411,1
.5787408,-3.0555819,-0.6947886,0.7095906,-0.3258679\PG=C01 [X(C2H4Se1)
]\\@

1\1\GINC-ORC340\FOpt\RB3PW91\6-311+G(2df,p)\C2H4Se1\MEISTERP\28-Jul-20
17\0\\# opt=readfc freq b3pw91/6-311+g(2df,p)\\CH2CHSeH\\0,1\C,2.13792
54294,-0.3029297859,-0.0017419436\H,2.065678932,-1.3827644005,-0.07482
59552\H,3.1281332205,0.1148694245,0.1409695269\C,1.0821335074,0.494613
8482,-0.0796779512\H,1.1779147146,1.571303013,-0.0039665528\Se,-0.6958
725749,-0.1291890882,-0.3286094665\H,-1.300213229,1.2073489889,-0.3192
536577\\Version=EM64L-G09RevE.01\State=1-A\HF=-2480.1148271\RMSD=8.862
e-09\RMSF=8.755e-06\Dipole=0.1571795,0.3335535,0.0388519\Quadrupole=1.
408362,1.8698652,-3.2782272,-0.7530027,0.5200785,0.224939\PG=C01 [X(C2
H4Se1)]\\@

1\1\GINC-ORC338\FOpt\RB3PW91\6-311++G(3df,3pd)\C2H4Se1\MEISTERP\28-Jul
-2017\0\\# opt=readfc freq b3pw91/6-311++g(3df,3pd)\\CH2CHSeH\\0,1\C,2
.1380246779,-0.3038840566,-0.0020033094\H,2.0655655548,-1.3821940789,-
0.0760636818\H,3.1265572247,0.1141864872,0.1409505423\C,1.0824653297,0
.4942199442,-0.0790945713\H,1.178027776,1.5695477651,-0.0024784838\Se,
-0.6962460682,-0.1278939475,-0.3280100787\H,-1.2986944948,1.2092698866
,-0.3204064173\\Version=EM64L-G09RevE.01\State=1-A\HF=-2480.1206978\RM
SD=3.402e-09\RMSF=1.014e-05\Dipole=0.1549632,0.3172886,0.0373863\Quadr
upole=1.3615345,1.7796263,-3.1411608,-0.7501267,0.5001452,0.2105978\PG
=C01 [X(C2H4Se1)]\\@

CH_2CHSeH wB97XD

1\1\GINC-ORC376\FOpt\RwB97XD\6-31G(d)\C2H4Se1\MEISTERP\28-Jul-2017\0\\
opt=readfc freq wb97xd/6-31g(d)\\CH2CHSeH\\0,1\C,2.1176614109,-0.284
8342594,-0.0533335472\H,1.9892923166,-1.3482185406,-0.2348510538\H,3.1
355163182,0.0821610705,0.0455355862\C,1.0842615018,0.5436424624,0.0552
918999\H,1.2265495041,1.6056638293,0.2316868158\Se,-0.7162806683,-0.07
342745,0.0509744923\H,-1.2413003834,1.0482648878,-0.7624101932\\Versio
n=EM64L-G09RevE.01\State=1-A\HF=-2477.9477543\RMSD=5.327e-09\RMSF=7.98
7e-06\Dipole=0.3064942,0.3042608,-0.1166233\Quadrupole=1.6335581,0.854
1999,-2.487758,-0.4870832,0.9092432,-0.7484248\PG=C01 [X(C2H4Se1)]\\@

1\1\GINC-ORC375\FOpt\RwB97XD\6-311G(d)\C2H4Se1\MEISTERP\28-Jul-2017\0\
\# opt=readfc freq wb97xd/6-311g(d)\\CH2CHSeH\\0,1\C,2.1232106149,-0.2
893651809,-0.0468902556\H,2.012778117,-1.3559683952,-0.2151757673\H,3.
1336554148,0.0930508707,0.058252408\C,1.0843481769,0.5297170041,0.0354
134033\H,1.2128537664,1.5943825366,0.1996356786\Se,-0.7244816561,-0.08
72430197,0.012162543\H,-1.2466644339,1.0886781844,-0.71050401\\Version
=EM64L-G09RevE.01\State=1-A\HF=-2480.1440905\RMSD=8.030e-09\RMSF=7.944
e-06\Dipole=0.2659102,0.3131691,-0.0970393\Quadrupole=1.7195148,1.1278
741,-2.8473889,-0.5285423,0.8852498,-0.7322443\PG=C01 [X(C2H4Se1)]\\@

1\1\GINC-ORC375\FOpt\RwB97XD\6-311G(d,p)\C2H4Se1\MEISTERP\28-Jul-2017\
0\\# opt=readfc freq wb97xd/6-311g(d,p)\\CH2CHSeH\\0,1\C,2.128988726,-
0.2934460751,-0.0379469807\H,2.0275845686,-1.3629230558,-0.1882920631\
H,3.1329029653,0.1003031591,0.0771763234\C,1.083627974,0.5197532507,0.
0120458348\H,1.1999676649,1.5874738558,0.1585014677\Se,-0.7210913168,-
0.1036791824,-0.0491797107\H,-1.2562805819,1.1257700477,-0.6394108715\
\Version=EM64L-G09RevE.01\State=1-A\HF=-2480.1527014\RMSD=9.384e-09\RM
SF=2.885e-05\Dipole=0.231105,0.3311168,-0.0732996\Quadrupole=1.6610649
,1.2887598,-2.9498247,-0.5994183,0.8350046,-0.5989585\PG=C01 [X(C2H4Se
1)]\\@

1\1\GINC-ORC374\FOpt\RwB97XD\6-311+G(d,p)\C2H4Se1\MEISTERP\28-Jul-2017
\0\\# opt=readfc freq wb97xd/6-311+g(d,p)\\CH2CHSeH\\0,1\C,2.130381835
7,-0.2945889967,-0.0363763355\H,2.0321591603,-1.364853471,-0.184224964
8\H,3.1330734449,0.1020010999,0.0800368031\C,1.0824023512,0.5174264826
,0.0078542796\H,1.1970946045,1.5858896797,0.1511498765\Se,-0.720862733
2,-0.107035245,-0.062088256\H,-1.2585486634,1.1344124505,-0.623457403\
\Version=EM64L-G09RevE.01\State=1-A\HF=-2480.1539309\RMSD=3.625e-09\RM
SF=2.191e-05\Dipole=0.2246111,0.3375742,-0.0687739\Quadrupole=1.633738
3,1.432035,-3.0657734,-0.6302934,0.8370802,-0.5773708\PG=C01 [X(C2H4Se
1)]\\@

1\1\GINC-ORC374\FOpt\RwB97XD\6-311G(2d,p)\C2H4Se1\MEISTERP\28-Jul-2017
\0\\# opt=readfc freq wb97xd/6-311g(2d,p)\\CH2CHSeH\\0,1\C,2.127571601
9,-0.2925796783,-0.0378456973\H,2.028458104,-1.3618938103,-0.186014487
1\H,3.1299394945,0.1028658771,0.0786522641\C,1.0837300925,0.5165952208
,0.0084127615\H,1.2006345251,1.5834853861,0.1535775733\Se,-0.717648434
1,-0.1055841734,-0.0574397658\H,-1.2569853839,1.1303631779,-0.62644864
85\\Version=EM64L-G09RevE.01\State=1-A\HF=-2480.1534766\RMSD=3.831e-09
\RMSF=2.155e-05\Dipole=0.2407087,0.3062064,-0.0605573\Quadrupole=1.589
8946,1.3345046,-2.9243991,-0.560844,0.7861236,-0.5865002\PG=C01 [X(C2H
4Se1)]\\@

1\1\GINC-ORC373\FOpt\RwB97XD\6-311G(df,p)\C2H4Se1\MEISTERP\28-Jul-2017
\0\\# opt=readfc freq wb97xd/6-311g(df,p)\\CH2CHSeH\\0,1\C,2.128951295
6,-0.2931549576,-0.0327494547\H,2.0340009446,-1.363734391,-0.178707411
1\H,3.1307248566,0.1036836285,0.0880315877\C,1.081690861,0.5148841732,
0.0048905947\H,1.1946288064,1.5834450684,0.1467418963\Se,-0.7158993417
,-0.1096105251,-0.0747986617\H,-1.2583974224,1.1377390035,-0.620514551
1\\Version=EM64L-G09RevE.01\State=1-A\HF=-2480.1580294\RMSD=9.106e-09\
RMSF=2.041e-05\Dipole=0.2254183,0.3385253,-0.0630259\Quadrupole=1.5590
97,1.332879,-2.891976,-0.6244159,0.80983,-0.520363\PG=C01 [X(C2H4Se1)]
\\@

1\1\GINC-ORC373\FOpt\RwB97XD\6-311+G(2df,p)\C2H4Se1\MEISTERP\28-Jul-20
17\0\\# opt=readfc freq wb97xd/6-311+g(2df,p)\\CH2CHSeH\\0,1\C,2.12277
05164,-0.2986566007,0.0250386607\H,2.0161116962,-1.3773764449,0.003386
5329\H,3.1170736263,0.0981091105,0.1933606802\C,1.0987547401,0.5203240
689,-0.1482899155\H,1.2244873931,1.5958205961,-0.1186879814\Se,-0.6568
240181,-0.082447327,-0.5628526035\H,-1.326673954,1.1174785971,-0.05906
13733\\Version=EM64L-G09RevE.01\State=1-A\HF=-2480.1587303\RMSD=4.647e
-09\RMSF=2.145e-05\Dipole=0.2009281,0.3057657,0.1395298\Quadrupole=1.6
387535,1.342133,-2.9808865,-0.752511,0.2633492,0.89468\PG=C01 [X(C2H4S
e1)]\\@

1\1\GINC-ORC372\FOpt\RwB97XD\6-311++G(3df,3pd)\C2H4Se1\MEISTERP\28-Jul
-2017\0\\# opt=readfc freq wb97xd/6-311++g(3df,3pd)\\CH2CHSeH\\0,1\C,2
.1311949329,-0.2950878159,-0.0333200236\H,2.0409747504,-1.3640978905,-
0.1777114826\H,3.1276224171,0.1067619381,0.0943068766\C,1.0813310886,0
.5090468349,-0.0052363137\H,1.19175526,1.5757036253,0.1359740268\Se,-0
.7120046849,-0.1159440254,-0.1085945633\H,-1.2651737641,1.1568693337,-
0.5725245203\\Version=EM64L-G09RevE.01\State=1-A\HF=-2480.1649376\RMSD
=5.840e-09\RMSF=2.523e-05\Dipole=0.2180436,0.3081217,-0.0423685\Quadru
pole=1.4873187,1.4807911,-2.9681098,-0.6429413,0.7331917,-0.3929038\PG
=C01 [X(C2H4Se1)]\\@

CH_2CHSeH M062X


```
1\1\GINC-ORC380\FOpt\RM062X\6-31G(d)\C2H4Se1\MEISTERP\28-Jul-2017\0\\#
 opt=readfc freq m062x/6-31g(d)\\CH2CHSeH\\0,1\C,2.1072938748,-0.27700
 53135,-0.0620953103\H,1.9543531936,-1.3317778807,-0.2700990118\H,3.132
 3160148,0.0721698508,0.0243045502\C,1.0880960908,0.5611103135,0.087880
 3373\H,1.2428619577,1.616447341,0.2899027101\Se,-0.7155470216,-0.04866
 74483,0.102684108\H,-1.2136741101,0.9809751371,-0.8396833836\\Version=
 EM64L-G09RevE.01\State=1-A\HF=-2477.9544144\RMSD=9.572e-09\RMSF=3.397e
 -05\Dipole=0.3472114,0.2849926,-0.1516311\Quadrupole=1.6253382,0.54561
 89,-2.1709571,-0.4076903,0.9311819,-0.7490158\PG=C01 [X(C2H4Se1)]\\@

1\1\GINC-ORC379\FOpt\RM062X\6-311G(d)\C2H4Se1\MEISTERP\28-Jul-2017\0\\
 # opt=readfc freq m062x/6-311g(d)\\CH2CHSeH\\0,1\C,2.1220891246,-0.288
 0443013,-0.0474900583\H,2.0013837187,-1.3522945583,-0.2181074775\H,3.1
 341927915,0.0882216434,0.0543807223\C,1.0876062184,0.5356599822,0.0394
 813623\H,1.2155745361,1.5990845957,0.2057585516\Se,-0.7204460901,-0.08
 62815649,0.0206629405\H,-1.2447002992,1.0769062033,-0.7217920408\\Vers
 ion=EM64L-G09RevE.01\State=1-A\HF=-2480.1047836\RMSD=3.584e-09\RMSF=1.
 232e-05\Dipole=0.2805077,0.3241667,-0.1086666\Quadrupole=1.7434313,1.0
 933481,-2.8367794,-0.55174,0.9069621,-0.7496111\PG=C01 [X(C2H4Se1)]\\@

1\1\GINC-ORC379\FOpt\RB3PW91\6-311G(d,p)\C2H4Se1\MEISTERP\28-Jul-2017\
 0\\# opt=readfc freq b3pw91/6-311g(d,p)\\CH2CHSeH\\0,1\C,2.137033175,-
 0.2993118018,-0.0276360299\H,2.0531270733,-1.3738460658,-0.153858055\H
 ,3.1350833465,0.1071503651,0.0989451834\C,1.0814797958,0.506592001,-0.
 0185950835\H,1.1822245233,1.5791986389,0.1058798859\Se,-0.7188947855,-
 0.1207088164,-0.1286099898\H,-1.2743531284,1.1741776789,-0.5432319111\
 \Version=EM64L-G09RevE.01\State=1-A\HF=-2480.1087589\RMSD=3.864e-09\RM
 SF=7.675e-06\Dipole=0.183545,0.346114,-0.0372107\Quadrupole=1.578916,1
 .5629581,-3.1418741,-0.6799212,0.7534726,-0.3961449\PG=C01 [X(C2H4Se1)
 ]\\@

1\1\GINC-ORC378\FOpt\RM062X\6-311+G(d,p)\C2H4Se1\MEISTERP\28-Jul-2017\
 0\\# opt=readfc freq m062x/6-311+g(d,p)\\CH2CHSeH\\0,1\C,2.1239679179,
 -0.2894527024,-0.0455844847\H,2.0032514411,-1.3534752476,-0.2144748559
 \H,3.133803898,0.090697543,0.0581713473\C,1.0873733611,0.5337528992,0.
 0358609083\H,1.2113537757,1.597693528,0.1983337757\Se,-0.7196875939,-0
 .089314577,0.0050627414\H,-1.2443627999,1.083350557,-0.7044754321\\Ver
 sion=EM64L-G09RevE.01\State=1-A\HF=-2480.1132602\RMSD=8.028e-09\RMSF=2
 .114e-05\Dipole=0.2675319,0.3317678,-0.1073599\Quadrupole=1.6827082,1.
 1744974,-2.8572056,-0.5794785,0.8986319,-0.7147473\PG=C01 [X(C2H4Se1)]
 \\@

1\1\GINC-ORC378\FOpt\RM062X\6-311G(2d,p)\C2H4Se1\MEISTERP\28-Jul-2017\
 0\\# opt=readfc freq m062x/6-311g(2d,p)\\CH2CHSeH\\0,1\C,2.1284780964,
 -0.292298899,-0.0359986532\H,2.0236965668,-1.3607010091,-0.180440172\H
 ,3.1306822794,0.1011893502,0.0790715333\C,1.0857071754,0.5181490684,0.
 0063824209\H,1.1973855112,1.5852002383,0.1473935671\Se,-0.7141672885,-
 0.1109316163,-0.0656895829\H,-1.2560823407,1.1326448675,-0.6178251133\
 \Version=EM64L-G09RevE.01\State=1-A\HF=-2480.1136838\RMSD=4.600e-09\RM
 SF=2.688e-05\Dipole=0.2480425,0.3255891,-0.0631274\Quadrupole=1.614244
 1,1.389319,-3.003563,-0.6051868,0.7971005,-0.5756072\PG=C01 [X(C2H4Se1
 )]\\@

1\1\GINC-ORC377\FOpt\RM062X\6-311G(df,p)\C2H4Se1\MEISTERP\28-Jul-2017\
 0\\# opt=readfc freq m062x/6-311g(df,p)\\CH2CHSeH\\0,1\C,2.1289652187,
```

-0.2928924211,-0.0354700617\H,2.0276361617,-1.3625765909,-0.1803660714
\H,3.1320416447,0.1007544181,0.0800549702\C,1.083721532,0.5171043664,0
.0059872693\H,1.1945523423,1.5855845315,0.1461586166\Se,-0.7134612531,
-0.1117214399,-0.0697568225\H,-1.2577556463,1.1369991359,-0.6137139005
\\Version=EM64L-G09RevE.01\State=1-A\HF=-2480.1173215\RMSD=6.751e-09\R
MSF=2.188e-05\Dipole=0.2393904,0.353544,-0.0679841\Quadrupole=1.580128
,1.350872,-2.931,-0.6630193,0.8037695,-0.5153974\PG=C01 [X(C2H4Se1)]\\
@

1\1\GINC-ORC377\FOpt\RM062X\6-311+G(2df,p)\C2H4Se1\MEISTERP\28-Jul-201
7\0\\# opt=readfc freq m062x/6-311+g(2df,p)\\CH2CHSeH\\0,1\C,2.1295697
869,-0.2932265785,-0.0334919342\H,2.0284736109,-1.3627549376,-0.174783
376\H,3.1310329782,0.1014905752,0.0849533454\C,1.0840798836,0.51584972
55,0.0003066461\H,1.1945147423,1.5840271485,0.1371421238\Se,-0.7097466
953,-0.114347946,-0.084688969\H,-1.2622243066,1.142214013,-0.596543836
2\\Version=EM64L-G09RevE.01\State=1-A\HF=-2480.1189684\RMSD=9.306e-09\
RMSF=1.309e-05\Dipole=0.2364892,0.3300166,-0.0566826\Quadrupole=1.5404
54,1.499612,-3.040066,-0.642401,0.7833481,-0.5005058\PG=C01 [X(C2H4Se1
)]\\@

1\1\GINC-ORC376\FOpt\RM062X\6-311++G(3df,3pd)\C2H4Se1\MEISTERP\28-Jul-
2017\0\\# opt=readfc freq m062x/6-311++g(3df,3pd)\\CH2CHSeH\\0,1\C,2.1
30332236,-0.2937206862,-0.0331241395\H,2.0299678219,-1.3616270823,-0.1
726852226\H,3.1293621973,0.1019758205,0.0860011333\C,1.0843091512,0.51
5076216,-0.0013827517\H,1.1940068279,1.5816365526,0.1340444371\Se,-0.7
09620942,-0.1146178132,-0.0888322757\H,-1.2626572922,1.1445289927,-0.5
911271809\\Version=EM64L-G09RevE.01\State=1-A\HF=-2480.1249887\RMSD=4.
164e-09\RMSF=1.385e-05\Dipole=0.2300922,0.3152776,-0.0534876\Quadrupol
e=1.5019627,1.4275003,-2.929463,-0.6507324,0.7415466,-0.4512317\PG=C01
 [X(C2H4Se1)]\\@

CH_2CHSeH M08HX

1\1\GINC-ORC14\FOpt\RM08HX\6-31G(d)\C2H4Se1\MEISTERP\18-Aug-2017\0\\#
opt=readfc freq m08hx/6-31g(d)\\CH2CHSeH\\0,1\C,2.1160697333,-0.283662
5224,-0.0536417754\H,1.9791860427,-1.3498760017,-0.2383985525\H,3.1397
773276,0.0798742477,0.0432309001\C,1.0852058523,0.5478710885,0.0581765
206\H,1.2258304382,1.6140902111,0.2369409765\Se,-0.713183156,-0.075646
5454,0.0555743755\H,-1.2371862379,1.0406015222,-0.7689884448\\Version=
EM64L-G16RevA.03\State=1-A\HF=-2477.995561\RMSD=8.782e-09\RMSF=7.653e-
06\Dipole=0.310214,0.3169416,-0.1252459\Quadrupole=1.6255573,0.8672343
,-2.4927916,-0.4972087,0.9193859,-0.7373295\PG=C01 [X(C2H4Se1)]\\@

1\1\GINC-ORC10\FOpt\RM08HX\6-311G(d)\C2H4Se1\MEISTERP\18-Aug-2017\0\\#
opt=readfc freq m08hx/6-311g(d)\\CH2CHSeH\\0,1\C,2.1274961662,-0.2917
408707,-0.0407245329\H,2.0192169943,-1.3642714297,-0.1968032462\H,3.13
88064426,0.0961315588,0.071123444\C,1.0845656732,0.5247116971,0.018511
6983\H,1.2004564163,1.5961616441,0.170785777\Se,-0.7193838519,-0.10661
96953,-0.0321736577\H,-1.2554578407,1.1188790957,-0.6578254825\\Versio
n=EM64L-G16RevA.03\State=1-A\HF=-2480.1259337\RMSD=8.250e-09\RMSF=2.62
2e-05\Dipole=0.2493701,0.3430695,-0.0817124\Quadrupole=1.7044105,1.345
3161,-3.0497265,-0.6082799,0.868566,-0.6508502\PG=C01 [X(C2H4Se1)]\\@

1\1\GINC-ORC294\FOpt\RM08HX\6-311G(d,p)\C2H4Se1\MEISTERP\18-Aug-2017\0
\\# opt=readfc freq m08hx/6-311g(d,p)\\CH2CHSeH\\0,1\C,2.1293254048,-0
.2930613727,-0.0381529776\H,2.0201435225,-1.3652972176,-0.1892695384\H
,3.1379816921,0.0982311279,0.0761413758\C,1.0856603644,0.5228007387,0.

```
0123605442\H,1.1968592506,1.5945530931,0.1588416793\Se,-0.7173420627,-
0.1094897873,-0.0525961927\H,-1.2569281718,1.1255154178,-0.6344308906\
\Version=EM64L-G16RevA.03\State=1-A\HF=-2480.1332168\RMSD=4.176e-09\RM
SF=8.547e-06\Dipole=0.2382861,0.3473175,-0.0755971\Quadrupole=1.668279
2,1.3370111,-3.0052903,-0.6282081,0.8386004,-0.589603\PG=C01 [X(C2H4Se
1)]\\@
```

```
1\1\GINC-ORC99\FOpt\RM08HX\6-311+G(d,p)\C2H4Se1\MEISTERP\18-Aug-2017\0
\\# opt=readfc freq m08hx/6-311+g(d,p)\\CH2CHSeH\\0,1\C,2.1302452907,-
0.2940144505,-0.0365994717\H,2.0235044917,-1.3669201814,-0.186063005\H
,3.1383098651,0.0990654332,0.0779726222\C,1.0843983628,0.5215556877,0.
0098505372\H,1.1950682724,1.5940012694,0.1534662807\Se,-0.7171620282,-
0.1111731461,-0.0600232186\H,-1.2586642545,1.1307373878,-0.6257097447\
\Version=EM64L-G16RevA.03\State=1-A\HF=-2480.1347962\RMSD=1.427e-09\RM
SF=3.275e-05\Dipole=0.2277552,0.3501672,-0.0738697\Quadrupole=1.623306
5,1.4598865,-3.083193,-0.6448351,0.8406379,-0.5815676\PG=C01 [X(C2H4Se
1)]\\@
```

```
1\1\GINC-ORC90\FOpt\RM08HX\6-311G(2d,p)\C2H4Se1\MEISTERP\18-Aug-2017\0
\\# opt=readfc freq m08hx/6-311g(2d,p)\\CH2CHSeH\\0,1\C,2.1277723115,-
0.2919765306,-0.0371602793\H,2.0201173494,-1.363756722,-0.1857261572\H
,3.1348388408,0.100157872,0.0788274103\C,1.0858823099,0.5200755018,0.0
088151404\H,1.1973745055,1.5908591075,0.1532503861\Se,-0.7132596619,-0
.1111510325,-0.0604285695\H,-1.2570256551,1.1290438039,-0.6246839308\\
Version=EM64L-G16RevA.03\State=1-A\HF=-2480.1353525\RMSD=3.297e-09\RMS
F=8.716e-06\Dipole=0.2416581,0.3189161,-0.0629543\Quadrupole=1.5888699
,1.3726294,-2.9614993,-0.587366,0.7906033,-0.5823055\PG=C01 [X(C2H4Se1
)]\\@
```

```
1\1\GINC-ORC84\FOpt\RM08HX\6-311G(df,p)\C2H4Se1\MEISTERP\18-Aug-2017\0
\\# opt=readfc freq m08hx/6-311g(df,p)\\CH2CHSeH\\0,1\C,2.1283870341,-
0.2927947758,-0.0361582394\H,2.0240303426,-1.3661435413,-0.1837470783\
H,3.136469736,0.0997547128,0.0792935377\C,1.0841748558,0.5191597348,0.
0081623512\H,1.1946695396,1.5916760098,0.1502709006\Se,-0.7128212784,-
0.1119232403,-0.0646888837\H,-1.2592102298,1.1335231,-0.620238588\\Ver
sion=EM64L-G16RevA.03\State=1-A\HF=-2480.1389522\RMSD=8.391e-09\RMSF=2
.601e-05\Dipole=0.2328527,0.3493952,-0.0688129\Quadrupole=1.5543343,1.
344059,-2.8983933,-0.6446453,0.7983195,-0.5199799\PG=C01 [X(C2H4Se1)]\
\@
```

```
1\1\GINC-ORC77\FOpt\RM08HX\6-311+G(2df,p)\C2H4Se1\MEISTERP\18-Aug-2017
\0\\# opt=readfc freq m08hx/6-311+g(2df,p)\\CH2CHSeH\\0,1\C,2.13366654
,-0.2968086796,-0.0239059519\H,2.0422943123,-1.3738513355,-0.145623275
9\H,3.1343141971,0.1075668729,0.1043048609\C,1.0829530936,0.5071202091
,-0.0252960586\H,1.1842099299,1.582409257,0.0931376113\Se,-0.704843829
8,-0.1289420766,-0.1596170261\H,-1.2768942431,1.1757577527,-0.51010615
98\\Version=EM64L-G16RevA.03\State=1-A\HF=-2480.1413704\RMSD=9.765e-09
\RMSF=5.698e-06\Dipole=0.1969543,0.3358428,-0.0250953\Quadrupole=1.476
0022,1.7106526,-3.1866547,-0.6948416,0.7070787,-0.2936749\PG=C01 [X(C2
H4Se1)]\\@
```

```
1\1\GINC-ORC43\FOpt\RM08HX\6-311++G(3df,3pd)\C2H4Se1\MEISTERP\18-Aug-2
017\0\\# opt=readfc freq m08hx/6-311++g(3df,3pd)\\CH2CHSeH\\0,1\C,2.13
46346386,-0.2975044773,-0.0227524405\H,2.043843663,-1.372374616,-0.141
3569765\H,3.1319652254,0.1081359381,0.1061826685\C,1.0835523799,0.5062
307089,-0.0283397874\H,1.1829454185,1.5796161247,0.0875857758\Se,-0.70
42605772,-0.1294664438,-0.1693061683\H,-1.2769807482,1.1786147655,-0.4
```

991190715\\Version=EM64L-G16RevA.03\State=1-A\HF=-2480.1478655\RMSD=9.
080e-09\RMSF=1.435e-05\Dipole=0.194381,0.3302788,-0.0216402\Quadrupole
=1.453735,1.6547365,-3.1084716,-0.7108297,0.6738234,-0.2414472\PG=C01
[X(C2H4Se1)]\\@

CH_2CHSeH QCISD

1\1\GINC-ORC171\FOpt\RQCISD-FC\6-311+G(2df,p)\C2H4Se1\MEISTERP\20-Aug-
2017\0\\# opt freq qcisd/6-311+g(2df,p) guess=read\\CH2CHSeH\\0,1\C,2.
1386643894,-0.2812535402,0.0002827161\H,2.0407491235,-1.3622471656,0.0
00775283\H,3.1446613857,0.1236185647,0.0000637994\C,1.0857075666,0.532
2606671,-0.000072504\H,1.2043860426,1.6101330511,-0.0005406575\Se,-0.7
173383704,-0.0917175671,-0.0002389209\H,-1.3131991375,1.2485289901,0.0
020112839\\Version=EM64L-G09RevE.01\State=1-A\HF=-2477.856112\MP2=-247
8.3288855\MP3=-2478.3612272\MP4D=-2478.3741259\MP4DQ=-2478.3604403\MP4
SDQ=-2478.3645357\QCISD=-2478.364918\RMSD=7.194e-09\RMSF=3.463e-05\Dip
ole=0.2060632,0.3366251,0.0003409\PG=C01 [X(C2H4Se1)]\\@

CH_2CHSe^- B3LYP

1\1\GINC-ORC251\FOpt\RB3LYP\6-31G(d)\C2H3Se1(1-)\MEISTERP\24-Jun-2017\
0\\# opt freq b3lyp/6-31g(d)\\CH2CHSe\\-1,1\C,-3.9296391916,0.56183953
6,-0.0000514064\H,-3.4717912958,-0.426478324,0.0004112672\H,-5.0221060
439,0.597936337,-0.0020537796\C,-3.1817420112,1.6869084034,0.001552781
8\H,-3.7459295274,2.6283849226,0.0005734745\Se,-1.30348917,1.876185575
1,0.0048755925\\Version=EM64L-G16RevA.03\State=1-A\HF=-2477.3952446\RM
SD=3.795e-09\RMSF=8.433e-05\Dipole=-0.3098969,0.0723476,-0.0006287\Qua
drupole=-1.3774668,0.7504366,0.6270303,-2.2223231,-0.0038009,-0.003760
1\PG=C01 [X(C2H3Se1)]\\@

1\1\GINC-ORC181\FOpt\RB3LYP\6-311G(d)\C2H3Se1(1-)\MEISTERP\24-Jun-2017
\0\\# opt freq b3lyp/6-311g(d)\\CH2CHSe\\-1,1\C,-3.9310974225,0.559673
203,-0.0000536973\H,-3.4722402153,-0.4265565299,0.0004155869\H,-5.0221
618882,0.5990189372,-0.0020670856\C,-3.1898642588,1.6835647442,0.00157
01132\H,-3.7410824917,2.6291179461,0.0005917639\Se,-1.2982509634,1.879
9581495,0.0048512489\\Version=EM64L-G16RevA.03\State=1-A\HF=-2479.6034
286\RMSD=9.402e-09\RMSF=5.743e-05\Dipole=-0.3988208,0.063245,-0.000825
2\Quadrupole=-1.039233,0.762131,0.277102,-2.1574001,-0.0024644,-0.0035
276\PG=C01 [X(C2H3Se1)]\\@

1\1\GINC-ORC180\FOpt\RB3LYP\6-311G(d,p)\C2H3Se1(1-)\MEISTERP\24-Jun-20
17\0\\# opt freq b3lyp/6-311g(d,p)\\CH2CHSe\\-1,1\C,-3.9315052239,0.55
8736988,-0.0000683434\H,-3.4707279328,-0.42537602,0.000431341\H,-5.021
1003749,0.6004104155,-0.0020512038\C,-3.1909025924,1.6833020187,0.0015
234533\H,-3.7404042706,2.6285584273,0.0006098786\Se,-1.3000568455,1.87
91446205,0.0048628043\\Version=EM64L-G16RevA.03\State=1-A\HF=-2479.609
0328\RMSD=6.646e-09\RMSF=6.089e-05\Dipole=-0.3885402,0.0656562,-0.0007
672\Quadrupole=-1.1235109,0.7245491,0.3989618,-2.1689489,-0.0029335,-0
.0036404\PG=C01 [X(C2H3Se1)]\\@

1\1\GINC-ORC88\FOpt\RB3LYP\6-311+G(d,p)\C2H3Se1(1-)\MEISTERP\24-Jun-20
17\0\\# opt freq b3lyp/6-311+g(d,p)\\CH2CHSe\\-1,1\C,-3.9318043553,0.5
570974379,-0.0000720804\H,-3.469124596,-0.4257222462,0.000453923\H,-5.
0205260796,0.5985421698,-0.0020642662\C,-3.1930591228,1.6857738363,0.0
015220204\H,-3.7381438735,2.6319899208,0.0006220975\Se,-1.3020392129,1
.8770953314,0.0048462357\\Version=EM64L-G16RevA.03\State=1-A\HF=-2479.
6187865\RMSD=6.816e-09\RMSF=5.122e-05\Dipole=-0.5340957,0.0193819,-0.0

```
010556\Quadrupole=-0.7854834,0.6956472,0.0898363,-2.2825544,-0.0017087
,-0.0037451\PG=C01 [X(C2H3Se1)]\\@
```

```
1\1\GINC-ORC384\FOpt\RB3LYP\6-311G(2d,p)\C2H3Se1(1-)\MEISTERP\24-Jun-2
017\0\\# opt freq b3lyp/6-311g(2d,p)\\CH2CHSe\\-1,1\C,-3.9296754828,0.
5603573183,-0.0000713004\H,-3.4718763667,-0.4244292145,0.0004409344\H,
-5.0183076148,0.6041170588,-0.0020350705\C,-3.1909668613,1.6811732787,
0.0014936424\H,-3.741989954,2.6240317624,0.00060331\Se,-1.3018809604,1
.8795262462,0.0048764141\\Version=EM64L-G16RevA.03\State=1-A\HF=-2479.
6092402\RMSD=8.525e-09\RMSF=5.796e-05\Dipole=-0.3972223,0.0447604,-0.0
007626\Quadrupole=-1.2477008,0.7492966,0.4984042,-2.2009606,-0.0033967
,-0.0037556\PG=C01 [X(C2H3Se1)]\\@
```

```
1\1\GINC-ORC379\FOpt\RB3LYP\6-311G(df,p)\C2H3Se1(1-)\MEISTERP\24-Jun-2
017\0\\# opt freq b3lyp/6-311g(df,p)\\CH2CHSe\\-1,1\C,-3.9299808628,0.
5602101189,-0.0000770229\H,-3.4712343997,-0.4247885005,0.0004535923\H,
-5.019455278,0.6013546326,-0.0020293418\C,-3.1898383074,1.6820384896,0
.0014649926\H,-3.7401483782,2.6269336962,0.0006104258\Se,-1.3040400139
,1.8790280132,0.004885284\\Version=EM64L-G16RevA.03\State=1-A\HF=-2479
.6140969\RMSD=8.748e-09\RMSF=6.059e-05\Dipole=-0.3923785,0.068954,-0.0
00735\Quadrupole=-1.2379993,0.7520105,0.4859888,-2.1995673,-0.0033925,
-0.0037729\PG=C01 [X(C2H3Se1)]\\@
```

```
1\1\GINC-ORC378\FOpt\RB3LYP\6-311+G(2df,p)\C2H3Se1(1-)\MEISTERP\24-Jun
-2017\0\\# opt freq b3lyp/6-311+g(2df,p)\\CH2CHSe\\-1,1\C,-3.929337258
7,0.5594779862,-0.0000827814\H,-3.4697937585,-0.4240563567,0.000483238
9\H,-5.0170872959,0.6016856888,-0.0020375533\C,-3.1920013838,1.6837409
427,0.0014489136\H,-3.7392962504,2.6275315692,0.0006236325\Se,-1.30718
12928,1.8763966198,0.0048724797\\Version=EM64L-G16RevA.03\State=1-A\HF
=-2479.6226722\RMSD=5.278e-09\RMSF=4.749e-05\Dipole=-0.5239772,0.01100
68,-0.000967\Quadrupole=-0.9386205,0.7304805,0.2081399,-2.3058808,-0.0
023776,-0.0039249\PG=C01 [X(C2H3Se1)]\\@
```

```
1\1\GINC-ORC374\FOpt\RB3LYP\6-311++G(3df,3pd)\C2H3Se1(1-)\MEISTERP\24-
Jun-2017\0\\# opt freq b3lyp/6-311++g(3df,3pd)\\CH2CHSe\\-1,1\C,-3.929
5247996,0.5590313639,-0.0000856834\H,-3.4708587822,-0.4231252346,0.000
4897519\H,-5.0154621658,0.6026819771,-0.0020348733\C,-3.1922721365,1.6
835515381,0.0014385114\H,-3.7391404717,2.6256472544,0.0006243464\Se,-1
.3074388841,1.8769895512,0.004875877\\Version=EM64L-G16RevA.03\State=1
-A\HF=-2479.6285022\RMSD=8.273e-09\RMSF=5.054e-05\Dipole=-0.4891847,0.
0091249,-0.000895\Quadrupole=-1.1509769,0.7697944,0.3811824,-2.3053249
,-0.0030985,-0.0039753\PG=C01 [X(C2H3Se1)]\\@
```

CH_2CHSe^- B3PW91

```
1\1\GINC-ORC378\FOpt\RB3PW91\6-31G(d)\C2H3Se1(1-)\MEISTERP\24-Jun-2017
\0\\# opt freq b3pw91/6-31g(d)\\CH2CHSe\\-1,1\C,-3.9280967633,0.562209
1322,-0.0000764508\H,-3.4675670671,-0.4252016564,0.00045711\H,-5.02053
04273,0.5990791555,-0.00199995\C,-3.1803597093,1.6870278657,0.00142569
96\H,-3.7441157882,2.6290031392,0.0005792987\Se,-1.3140274847,1.872658
8137,0.0049222226\\Version=EM64L-G16RevA.03\State=1-A\HF=-2477.3459247
\RMSD=5.048e-09\RMSF=9.031e-05\Dipole=-0.3308827,0.0665777,-0.0006085\
Quadrupole=-1.267975,0.7884664,0.4795087,-2.2027785,-0.0034874,-0.0038
057\PG=C01 [X(C2H3Se1)]\\@
```

```
1\1\GINC-ORC375\FOpt\RB3PW91\6-311G(d)\C2H3Se1(1-)\MEISTERP\24-Jun-201
7\0\\# opt freq b3pw91/6-311g(d)\\CH2CHSe\\-1,1\C,-3.9293022827,0.5600
```

690589,-0.0000891741\H,-3.4675521717,-0.4256018202,0.000466803\H,-5.02
06865803,0.6004604318,-0.0020070529\C,-3.1882366058,1.684004002,0.0014
177853\H,-3.7400862413,2.6300552122,0.000616868\Se,-1.3088333581,1.875
7895653,0.0049027009\\Version=EM64L-G16RevA.03\State=1-A\HF=-2479.5499
238\RMSD=4.346e-09\RMSF=6.186e-05\Dipole=-0.415081,0.0563026,-0.000743
7\Quadrupole=-0.9492979,0.7904114,0.1588864,-2.1446046,-0.0023743,-0.0
036469\PG=C01 [X(C2H3Se1)]\\@

1\1\GINC-ORC373\FOpt\RB3PW91\6-311G(d,p)\C2H3Se1(1-)\MEISTERP\24-Jun-2
017\0\\# opt freq b3pw91/6-311g(d,p)\\CH2CHSe\\-1,1\C,-3.9297418779,0.
5591489529,-0.0000989859\H,-3.4659215847,-0.4244828833,0.0004790367\H,
-5.0197558744,0.6018388819,-0.0019928679\C,-3.1892286801,1.683691501,0
.001379378\H,-3.7394414183,2.6296148093,0.0006299495\Se,-1.3106078046,
1.8749651882,0.0049114195\\Version=EM64L-G16RevA.03\State=1-A\HF=-2479
.5555355\RMSD=9.318e-09\RMSF=6.787e-05\Dipole=-0.4039192,0.0588293,-0.
0006949\Quadrupole=-1.0372452,0.7516878,0.2855575,-2.1565498,-0.002827
9,-0.0037379\PG=C01 [X(C2H3Se1)]\\@

1\1\GINC-ORC364\FOpt\RB3PW91\6-311+G(d,p)\C2H3Se1(1-)\MEISTERP\24-Jun-
2017\0\\# opt freq b3pw91/6-311+g(d,p)\\CH2CHSe\\-1,1\C,-3.9298630271,
0.5577904311,-0.0001009838\H,-3.4648153032,-0.4250301023,0.0004989168\
H,-5.019273034,0.6003217894,-0.0020097905\C,-3.1908039446,1.6857121141
,0.0013845178\H,-3.7374985304,2.632497785,0.0006416034\Se,-1.312443400
7,1.8734844325,0.0048936662\\Version=EM64L-G16RevA.03\State=1-A\HF=-24
79.56323\RMSD=6.858e-09\RMSF=5.760e-05\Dipole=-0.5261964,0.0213175,-0.
0009008\Quadrupole=-0.7918513,0.7337345,0.0581168,-2.2676338,-0.002020
9,-0.0039103\PG=C01 [X(C2H3Se1)]\\@

1\1\GINC-ORC363\FOpt\RB3PW91\6-311G(2d,p)\C2H3Se1(1-)\MEISTERP\24-Jun-
2017\0\\# opt freq b3pw91/6-311g(2d,p)\\CH2CHSe\\-1,1\C,-3.9278048831,
0.5607831151,-0.0000999538\H,-3.4668108848,-0.4235452981,0.000482248\H
,-5.0170386072,0.6054994126,-0.0019761422\C,-3.1893424383,1.6817632619
,0.0013571111\H,-3.7413244601,2.6253266205,0.0006221141\Se,-1.31237596
65,1.8749493381,0.0049225528\\Version=EM64L-G16RevA.03\State=1-A\HF=-2
479.5556852\RMSD=9.065e-09\RMSF=6.534e-05\Dipole=-0.4120305,0.0382383,
-0.0007038\Quadrupole=-1.1636084,0.7780825,0.385526,-2.1839398,-0.0032
908,-0.0038315\PG=C01 [X(C2H3Se1)]\\@

1\1\GINC-ORC391\FOpt\RB3PW91\6-311G(df,p)\C2H3Se1(1-)\MEISTERP\24-Jun-
2017\0\\# opt freq b3pw91/6-311g(df,p)\\CH2CHSe\\-1,1\C,-3.9282766898,
0.5605133693,-0.0001037804\H,-3.4664792965,-0.4240248905,0.0004893573\
H,-5.0181755774,0.6028218229,-0.0019717816\C,-3.1879961056,1.682581242
9,0.0013396061\H,-3.7393640647,2.6279781392,0.0006258596\Se,-1.3144055
06,1.8749067662,0.004928669\\Version=EM64L-G16RevA.03\State=1-A\HF=-24
79.5604721\RMSD=4.630e-09\RMSF=6.950e-05\Dipole=-0.4068115,0.0628284,-
0.0006754\Quadrupole=-1.1568688,0.7789049,0.3779639,-2.1896579,-0.0032
975,-0.0038584\PG=C01 [X(C2H3Se1)]\\@

1\1\GINC-ORC390\FOpt\RB3PW91\6-311+G(2df,p)\C2H3Se1(1-)\MEISTERP\24-Ju
n-2017\0\\# opt freq b3pw91/6-311+g(2df,p)\\CH2CHSe\\-1,1\C,-3.9273972
273,0.5600776908,-0.0001029162\H,-3.4652809096,-0.4235032721,0.0005088
92\H,-5.0160043285,0.603441253,-0.0019869037\C,-3.1897449323,1.6838667
722,0.0013459891\H,-3.7388965584,2.6283026605,0.0006337896\Se,-1.31737
32838,1.8725913457,0.0049090791\\Version=EM64L-G16RevA.03\State=1-A\HF
=-2479.5669583\RMSD=5.546e-09\RMSF=5.802e-05\Dipole=-0.5145883,0.01172
48,-0.0008495\Quadrupole=-0.9436692,0.765914,0.1777553,-2.2946677,-0.0
026216,-0.0040399\PG=C01 [X(C2H3Se1)]\\@

```
1\1\GINC-ORC318\FOpt\RB3PW91\6-311++G(3df,3pd)\C2H3Se1(1-)\MEISTERP\24
 -Jun-2017\0\\# opt freq b3pw91/6-311++g(3df,3pd)\\CH2CHSe\\-1,1\C,-3.9
 274879594,0.559607036,-0.0001044443\H,-3.4663665249,-0.4229232916,0.00
 05133417\H,-5.0145822974,0.6046465186,-0.0019825048\C,-3.189841684,1.6
 835852374,0.0013353408\H,-3.7389086442,2.6264804893,0.0006331813\Se,-1
 .3175101301,1.8733804603,0.0049130154\\Version=EM64L-G16RevA.03\State=
 1-A\HF=-2479.5725442\RMSD=5.617e-09\RMSF=6.056e-05\Dipole=-0.4814387,0
 .0097574,-0.0007829\Quadrupole=-1.1447563,0.8055939,0.3391624,-2.29197
 13,-0.0033019,-0.0040754\PG=C01 [X(C2H3Se1)]\\@
```

CH_2CHSe^- wB97XD

```
1\1\GINC-ORC22\FOpt\RwB97XD\6-31G(d)\C2H3Se1(1-)\MEISTERP\07-Jul-2017\
 0\\# opt freq wb97xd/6-31g(d)\\CH2CHSeH\\-1,1\C,-3.9269825117,0.564744
 1239,-0.0000894757\H,-3.4640450571,-0.4207666885,0.0004840123\H,-5.018
 2920636,0.6003205656,-0.0019970121\C,-3.1831544921,1.6867450954,0.0013
 942692\H,-3.7473636329,2.6269520711,0.0005933399\Se,-1.3148594827,1.86
 67812824,0.0049227963\\Version=EM64L-G16RevA.03\State=1-A\HF=-2477.381
 5799\RMSD=3.409e-09\RMSF=7.030e-05\Dipole=-0.4003519,0.0480964,-0.0007
 177\Quadrupole=-1.1612854,0.7761092,0.3851762,-2.1588348,-0.0031775,-0
 .003764\PG=C01 [X(C2H3Se1)]\\@
```

```
1\1\GINC-ORC22\FOpt\RwB97XD\6-311G(d)\C2H3Se1(1-)\MEISTERP\07-Jul-2017
 \0\\# opt freq wb97xd/6-311g(d)\\CH2CHSeH\\-1,1\C,-3.9283385679,0.5626
 871669,-0.0000980837\H,-3.4644214406,-0.4214694543,0.0004831909\H,-5.0
 188063418,0.601529083,-0.0020040913\C,-3.190530441,1.6838131507,0.0013
 983096\H,-3.7437118268,2.6279358979,0.0006273904\Se,-1.3088886219,1.87
 02806059,0.0049012141\\Version=EM64L-G16RevA.03\State=1-A\HF=-2479.592
 3378\RMSD=4.359e-09\RMSF=4.714e-05\Dipole=-0.4837597,0.0364751,-0.0008
 541\Quadrupole=-0.8315213,0.7681196,0.0634017,-2.107151,-0.0020357,-0.
 0036004\PG=C01 [X(C2H3Se1)]\\@
```

```
1\1\GINC-ORC370\FOpt\RwB97XD\6-311G(d,p)\C2H3Se1(1-)\MEISTERP\07-Jul-2
 017\0\\# opt freq wb97xd/6-311g(d,p)\\CH2CHSeH\\-1,1\C,-3.9287301336,0
 .5619015722,-0.000103992\H,-3.4625124911,-0.4201311964,0.000492296\H,-
 5.0178749413,0.6030441401,-0.0019950982\C,-3.191463849,1.6835477572,0.
 0013747966\H,-3.7435214071,2.6273637208,0.000635437\Se,-1.3105944179,1
 .8690504561,0.0049044906\\Version=EM64L-G16RevA.03\State=1-A\HF=-2479.
 5976996\RMSD=4.536e-09\RMSF=5.211e-05\Dipole=-0.4718799,0.0399397,-0.0
 00817\Quadrupole=-0.9163387,0.7286605,0.1876782,-2.1217611,-0.0024472,
 -0.0036807\PG=C01 [X(C2H3Se1)]\\@
```

```
1\1\GINC-ORC363\FOpt\RwB97XD\6-311+G(d,p)\C2H3Se1(1-)\MEISTERP\07-Jul-
 2017\0\\# opt freq wb97xd/6-311+g(d,p)\\CH2CHSeH\\-1,1\C,-3.9289161101
 ,0.5604556144,-0.0001045563\H,-3.4613068825,-0.4206958082,0.0005088714
 \H,-5.0174733025,0.6015985697,-0.0020111104\C,-3.1934800199,1.68550693
 79,0.001382136\H,-3.7415460312,2.6303146478,0.0006468046\Se,-1.3119748
 938,1.8675964883,0.0048857847\\Version=EM64L-G16RevA.03\State=1-A\HF=-
 2479.6056701\RMSD=5.897e-09\RMSF=4.333e-05\Dipole=-0.617444,-0.0046097
 ,-0.0010688\Quadrupole=-0.5988239,0.703577,-0.104753,-2.187883,-0.0013
 764,-0.0037541\PG=C01 [X(C2H3Se1)]\\@
```

```
1\1\GINC-ORC362\FOpt\RwB97XD\6-311G(2d,p)\C2H3Se1(1-)\MEISTERP\07-Jul-
 2017\0\\# opt freq wb97xd/6-311g(2d,p)\\CH2CHSeH\\-1,1\C,-3.9268422745
 ,0.5634580621,-0.0001027664\H,-3.4637062818,-0.4193728609,0.0004931343
 \H,-5.0151394333,0.6067885893,-0.0019803969\C,-3.1915086603,1.68151461
```

97,0.0013573735\H,-3.7452506781,2.6229430642,0.0006265951\Se,-1.312249
912,1.8694449756,0.0049139904\\Version=EM64L-G16RevA.03\State=1-A\HF=-
2479.5978968\RMSD=9.407e-09\RMSF=4.953e-05\Dipole=-0.4752127,0.0187334
,-0.000822\Quadrupole=-1.0349473,0.7568383,0.278109,-2.1332883,-0.0028
584,-0.0037358\PG=C01 [X(C2H3Se1)]\\@

1\1\GINC-ORC392\FOpt\RwB97XD\6-311+G(2df,p)\C2H3Se1(1-)\MEISTERP\07-Ju
l-2017\0\\# opt freq wb97xd/6-311+g(2df,p)\\CH2CHSeH\\-1,1\C,-3.926556
7048,0.5625998192,-0.0001030503\H,-3.4622848479,-0.4194952722,0.000515
8309\H,-5.0142585756,0.6048696105,-0.0019936494\C,-3.192301927,1.68350
42642,0.0013545048\H,-3.742836608,2.6259174454,0.0006375871\Se,-1.3164
585766,1.867380583,0.0048967068\\Version=EM64L-G16RevA.03\State=1-A\HF
=-2479.6094264\RMSD=6.543e-09\RMSF=4.230e-05\Dipole=-0.5998392,-0.0151
443,-0.0010196\Quadrupole=-0.7497896,0.7384971,0.0112925,-2.2013933,-0
.0019204,-0.003839\PG=C01 [X(C2H3Se1)]\\@

1\1\GINC-ORC391\FOpt\RwB97XD\6-311++G(3df,3pd)\C2H3Se1(1-)\MEISTERP\07
-Jul-2017\0\\# opt freq wb97xd/6-311++g(3df,3pd)\\CH2CHSeH\\-1,1\C,-3.
9267947454,0.5621449236,-0.0001028418\H,-3.4631579857,-0.4183917155,0.
0005186072\H,-5.0126477821,0.6061225819,-0.0019926753\C,-3.1925786848,
1.6832240636,0.0013528767\H,-3.742936435,2.6238177012,0.0006355032\Se,
-1.316581607,1.8678588952,0.00489646\\Version=EM64L-G16RevA.03\State=1
-A\HF=-2479.6153387\RMSD=2.781e-09\RMSF=4.371e-05\Dipole=-0.5758471,-0
.0215244,-0.0009786\Quadrupole=-0.935436,0.7932113,0.1422247,-2.192154
7,-0.0024951,-0.0038405\PG=C01 [X(C2H3Se1)]\\@

CH_2CHSe^- M062X

1\1\GINC-ORC303\FOpt\RM062X\6-31G(d)\C2H3Se1(1-)\MEISTERP\07-Jul-2017\
0\\# opt freq m062x/6-31g(d)\\CH2CHSe\\-1,1\C,-3.9272136826,0.56583647
88,-0.0001028058\H,-3.4594816466,-0.4159374587,0.0004938996\H,-5.01711
50785,0.5990573286,-0.0019742081\C,-3.185175725,1.6876001333,0.0013506
737\H,-3.7480934983,2.6275275648,0.0006109937\Se,-1.317617609,1.860692
4032,0.004929377\\Version=EM64L-G16RevA.03\State=1-A\HF=-2477.3974753\
RMSD=4.331e-09\RMSF=7.195e-05\Dipole=-0.4076413,0.0399726,-0.0007057\Q
uadrupole=-1.2083067,0.8024271,0.4058796,-2.1121997,-0.0033742,-0.0037
146\PG=C01 [X(C2H3Se1)]\\@

1\1\GINC-ORC363\FOpt\RM062X\6-311G(d)\C2H3Se1(1-)\MEISTERP\24-Jun-2017
\0\\# opt freq m062x/6-311g(d)\\CH2CHSe\\-1,1\C,-3.9291820691,0.563087
3116,-0.0001071097\H,-3.4597094642,-0.4165728083,0.000490622\H,-5.0178
953402,0.5996024032,-0.0019943976\C,-3.1936641768,1.6852974732,0.00137
73847\H,-3.7437602558,2.6291778884,0.0006446653\Se,-1.3104859339,1.864
184182,0.0048967653\\Version=EM64L-G16RevA.03\State=1-A\HF=-2479.56275
46\RMSD=9.109e-09\RMSF=4.554e-05\Dipole=-0.4986259,0.0308652,-0.000862
7\Quadrupole=-0.8266716,0.8113264,0.0153452,-2.0647533,-0.0019903,-0.0
035194\PG=C01 [X(C2H3Se1)]\\@

1\1\GINC-ORC362\FOpt\RM062X\6-311G(d,p)\C2H3Se1(1-)\MEISTERP\24-Jun-20
17\0\\# opt freq m062x/6-311g(d,p)\\CH2CHSe\\-1,1\C,-3.929623536,0.562
2845739,-0.0001101475\H,-3.4576095432,-0.4152265222,0.0004970755\H,-5.
0171899704,0.601101488,-0.0019887578\C,-3.1947169442,1.6850148384,0.00
13625263\H,-3.7435941524,2.6287112376,0.0006505027\Se,-1.3119630938,1.
8628908343,0.0048967308\\Version=EM64L-G16RevA.03\State=1-A\HF=-2479.5
669893\RMSD=7.804e-09\RMSF=4.937e-05\Dipole=-0.4932869,0.0333997,-0.00
08443\Quadrupole=-0.8932058,0.7799594,0.1132464,-2.0836462,-0.0023046,
-0.0035868\PG=C01 [X(C2H3Se1)]\\@

```
1\1\GINC-ORC390\FOpt\RM062X\6-311+G(d,p)\C2H3Se1(1-)\MEISTERP\24-Jun-2
 017\0\\# opt freq m062x/6-311+g(d,p)\\CH2CHSe\\-1,1\C,-3.9297325229,0.
 5608344316,-0.0001102998\H,-3.4568307631,-0.4160757672,0.0005148601\H,
 -5.016806948,0.59972333,-0.0020040832\C,-3.1959558616,1.6869829441,0.0
 013681483\H,-3.7417669764,2.6314931071,0.000658027\Se,-1.313604168,1.8
 618184045,0.0048812777\\Version=EM64L-G16RevA.03\State=1-A\HF=-2479.57
 43338\RMSD=2.199e-09\RMSF=4.239e-05\Dipole=-0.5986695,-0.0012753,-0.00
 10217\Quadrupole=-0.6603293,0.7770546,-0.1167253,-2.1861401,-0.0015146
 ,-0.0037405\PG=C01 [X(C2H3Se1)]\\@
```

```
1\1\GINC-ORC180\FOpt\RM062X\6-311G(2d,p)\C2H3Se1(1-)\MEISTERP\24-Jun-2
 017\0\\# opt freq m062x/6-311g(2d,p)\\CH2CHSe\\-1,1\C,-3.9278384156,0.
 5638356513,-0.0001085911\H,-3.4584834816,-0.4140689686,0.0004975669\H,
 -5.0143043481,0.604716285,-0.0019759238\C,-3.1948895517,1.6830311672,0
 .0013494804\H,-3.7452572299,2.6243827287,0.0006429235\Se,-1.3139242132
 ,1.8628795864,0.004902474\\Version=EM64L-G16RevA.03\State=1-A\HF=-2479
 .568124\RMSD=8.444e-09\RMSF=4.708e-05\Dipole=-0.4948076,0.0120109,-0.0
 008489\Quadrupole=-1.0107859,0.8100788,0.200707,-2.0880113,-0.002705,-
 0.0036249\PG=C01 [X(C2H3Se1)]\\@
```

```
1\1\GINC-ORC373\FOpt\RM062X\6-311G(df,p)\C2H3Se1(1-)\MEISTERP\24-Jun-2
 017\0\\# opt freq m062x/6-311g(df,p)\\CH2CHSe\\-1,1\C,-3.9282815056,0.
 5636545368,-0.0001099696\H,-3.4590787747,-0.4155476232,0.0005003843\H,
 -5.0160279202,0.6022408679,-0.001974435\C,-3.192978882,1.6836403022,0.
 0013399007\H,-3.7437471486,2.6267886769,0.0006423374\Se,-1.3145830089,
 1.8639996893,0.0049097123\\Version=EM64L-G16RevA.03\State=1-A\HF=-2479
 .5720316\RMSD=5.352e-09\RMSF=5.221e-05\Dipole=-0.4926501,0.0386673,-0.
 0008326\Quadrupole=-1.0337514,0.8106597,0.2230917,-2.1175192,-0.002805
 5,-0.003693\PG=C01 [X(C2H3Se1)]\\@
```

```
1\1\GINC-ORC382\FOpt\RM062X\6-311+G(2df,p)\C2H3Se1(1-)\MEISTERP\05-Jul
 -2017\0\\# opt freq m062x/6-311+g(2df,p)\\CH2CHSe\\-1,1\C,-3.927545919
 6,0.5631985838,-0.0001019348\H,-3.457687057,-0.4146841828,0.0005118126
 \H,-5.0137512615,0.6024571662,-0.0019931269\C,-3.1950986423,1.68521027
 09,0.0013553286\H,-3.7436764861,2.6274879643,0.0006499625\Se,-1.316937
 8735,1.8611066477,0.004885888\\Version=EM64L-G09RevE.01\State=1-A\HF=-
 2479.5789278\RMSD=7.688e-09\RMSF=5.144e-05\Dipole=-0.5787247,-0.011716
 6,-0.0009863\Quadrupole=-0.8164644,0.8233316,-0.0068673,-2.1910911,-0.
 0020042,-0.0037655\PG=C01 [X(C2H3Se1)]\\@
```

```
1\1\GINC-ORC389\FOpt\RM062X\6-311++G(3df,3pd)\C2H3Se1(1-)\MEISTERP\24-
 Jun-2017\0\\# opt freq m062x/6-311++g(3df,3pd)\\CH2CHSe\\-1,1\C,-3.927
 6856432,0.5625569567,-0.0001055704\H,-3.4595774032,-0.4141736276,0.000
 5180252\H,-5.0121280128,0.6042074484,-0.0019885334\C,-3.1945683427,1.6
 846266975,0.0013509175\H,-3.7432279129,2.6245940318,0.0006435598\Se,-1
 .3175099253,1.8629649433,0.0048895313\\Version=EM64L-G16RevA.03\State=
 1-A\HF=-2479.5846038\RMSD=8.589e-09\RMSF=4.415e-05\Dipole=-0.5418167,-
 0.0109844,-0.000916\Quadrupole=-1.0123446,0.8610549,0.1512898,-2.17711
 89,-0.0026692,-0.0037953\PG=C01 [X(C2H3Se1)]\\@
```

CH_2CHSe^- M08HX

```
1\1\GINC-ORC318\FOpt\RM08HX\6-31G(d)\C2H3Se1(1-)\MEISTERP\24-Jun-2017\
 0\\# opt freq m08hx/6-31g(d)\\CH2CHSe\\-1,1\C,-3.9261256855,0.56576253
 87,-0.0001175104\H,-3.4549286745,-0.4192799752,0.000493374\H,-5.020409
 3804,0.5983094374,-0.0019440264\C,-3.1838949698,1.6890831219,0.0012940
```

784\H,-3.7502708417,2.6336815308,0.0006297423\Se,-1.319067688,1.857219
7963,0.004952272\\Version=EM64L-G16RevA.03\State=1-A\HF=-2477.4409721\
RMSD=8.547e-09\RMSF=8.707e-05\Dipole=-0.4063032,0.0367339,-0.0006685\Q
uadrupole=-1.2135273,0.8478748,0.3656525,-2.1024087,-0.0034316,-0.0037
139\PG=C01 [X(C2H3Se1)]\\@

1\1\GINC-ORC277\FOpt\RM08HX\6-311G(d)\C2H3Se1(1-)\MEISTERP\24-Jun-2017
\0\\# opt freq m08hx/6-311g(d)\\CH2CHSe\\-1,1\C,-3.9279387849,0.562944
1051,-0.0001199515\H,-3.4540026148,-0.4198785916,0.0004941793\H,-5.021
4052552,0.598937609,-0.0019674375\C,-3.1930901182,1.6867704774,0.00132
33306\H,-3.7453158112,2.6359518949,0.0006587039\Se,-1.3129446557,1.860
0509552,0.0049191052\\Version=EM64L-G16RevA.03\State=1-A\HF=-2479.5859
956\RMSD=8.720e-09\RMSF=6.102e-05\Dipole=-0.4990107,0.0308697,-0.00082
14\Quadrupole=-0.8393854,0.8214019,0.0179834,-2.0552133,-0.0021422,-0.
0035457\PG=C01 [X(C2H3Se1)]\\@

1\1\GINC-ORC251\FOpt\RM08HX\6-311G(d,p)\C2H3Se1(1-)\MEISTERP\24-Jun-20
17\0\\# opt freq m08hx/6-311g(d,p)\\CH2CHSe\\-1,1\C,-3.9284049669,0.56
20989469,-0.0001222031\H,-3.4521418981,-0.4182679091,0.0004984342\H,-5
.0202834015,0.6006042624,-0.0019613074\C,-3.1941988424,1.6864423194,0.
0013113325\H,-3.7451564687,2.6350802688,0.0006643696\Se,-1.3145116623,
1.8588185615,0.0049173042\\Version=EM64L-G16RevA.03\State=1-A\HF=-2479
.5906076\RMSD=4.731e-09\RMSF=6.626e-05\Dipole=-0.4919467,0.0331735,-0.
0008037\Quadrupole=-0.9143391,0.7841547,0.1301844,-2.0674108,-0.002484
3,-0.0035991\PG=C01 [X(C2H3Se1)]\\@

1\1\GINC-ORC194\FOpt\RM08HX\6-311+G(d,p)\C2H3Se1(1-)\MEISTERP\24-Jun-2
017\0\\# opt freq m08hx/6-311+g(d,p)\\CH2CHSe\\-1,1\C,-3.9285024201,0.
5606521704,-0.0001209338\H,-3.4516965965,-0.4193672031,0.0005135041\H,
-5.0199375967,0.5988778245,-0.0019806009\C,-3.1950006112,1.6884743827,
0.0013247646\H,-3.7430382107,2.6381112215,0.0006720995\Se,-1.316521804
8,1.8580280541,0.0048990965\\Version=EM64L-G16RevA.03\State=1-A\HF=-24
79.5984254\RMSD=9.400e-09\RMSF=6.058e-05\Dipole=-0.5823811,0.0130605,-
0.0009429\Quadrupole=-0.7463494,0.7994929,-0.0531435,-2.2016141,-0.001
9472,-0.0038243\PG=C01 [X(C2H3Se1)]\\@

1\1\GINC-ORC181\FOpt\RB3LYP\6-311G(2d,p)\C2H3Se1(1-)\MEISTERP\24-Jun-2
017\0\\# opt freq b3lyp/6-311g(2d,p)\\CH2CHSe\\-1,1\C,-3.9296754828,0.
5603573183,-0.0000713004\H,-3.4718763667,-0.4244292145,0.0004409344\H,
-5.0183076148,0.6041170588,-0.0020350705\C,-3.1909668613,1.6811732787,
0.0014936425\H,-3.741989954,2.6240317624,0.00060331\Se,-1.3018809604,1
.8795262462,0.004876414\\Version=EM64L-G16RevA.03\State=1-A\HF=-2479.6
092402\RMSD=8.525e-09\RMSF=5.796e-05\Dipole=-0.3972223,0.0447604,-0.00
07626\Quadrupole=-1.2477008,0.7492966,0.4984042,-2.2009606,-0.0033967,
-0.0037556\PG=C01 [X(C2H3Se1)]\\@

1\1\GINC-ORC177\FOpt\RM08HX\6-311G(df,p)\C2H3Se1(1-)\MEISTERP\24-Jun-2
017\0\\# opt freq m08hx/6-311g(df,p)\\CH2CHSe\\-1,1\C,-3.9269676806,0.
5636517963,-0.0001125431\H,-3.4535636827,-0.4183696373,0.0004915755\H,
-5.0189781089,0.6016457297,-0.0019553424\C,-3.1925778798,1.6851062313,
0.0013196243\H,-3.7455921141,2.6331476979,0.0006453105\Se,-1.317017774
,1.8595946322,0.0049193052\\Version=EM64L-G16RevA.03\State=1-A\HF=-247
9.5960934\RMSD=7.933e-09\RMSF=7.498e-05\Dipole=-0.4893063,0.0389498,-0
.0008129\Quadrupole=-1.0556187,0.8192336,0.2363851,-2.1015993,-0.00292
19,-0.0036686\PG=C01 [X(C2H3Se1)]\\@

1\1\GINC-ORC88\FOpt\RM08HX\6-311+G(2df,p)\C2H3Se1(1-)\MEISTERP\24-Jun-
2017\0\\# opt freq m08hx/6-311+g(2df,p)\\CH2CHSe\\-1,1\C,-3.9260770848
,0.5631059075,-0.0001017965\H,-3.4529541236,-0.4178077698,0.000500916\
H,-5.0164021546,0.6022762325,-0.0019802867\C,-3.1937670867,1.686378630
2,0.0013554749\H,-3.7451121908,2.6332053046,0.0006449649\Se,-1.3203845
995,1.8576181451,0.0048886573\\Version=EM64L-G16RevA.03\State=1-A\HF=-
2479.6040512\RMSD=8.053e-09\RMSF=7.214e-05\Dipole=-0.5527479,0.0040227
,-0.0009316\Quadrupole=-0.9022438,0.8409668,0.061277,-2.1947249,-0.002
3327,-0.0037786\PG=C01 [X(C2H3Se1)]\\@

1\1\GINC-ORC380\FOpt\RM08HX\6-311++G(3df,3pd)\C2H3Se1(1-)\MEISTERP\24-
Jun-2017\0\\# opt freq m08hx/6-311++g(3df,3pd)\\CH2CHSe\\-1,1\C,-3.926
6044342,0.5623134271,-0.0001061645\H,-3.4543628898,-0.4161287357,0.000
5036862\H,-5.0142342889,0.6035952086,-0.0019714753\C,-3.1943444447,1.6
859477856,0.0013374286\H,-3.744340241,2.630358518,0.0006519805\Se,-1.3
208109414,1.8586902464,0.0048924745\\Version=EM64L-G16RevA.03\State=1-
A\HF=-2479.6097102\RMSD=4.336e-09\RMSF=6.993e-05\Dipole=-0.5227214,0.0
069568,-0.0008628\Quadrupole=-1.0574479,0.869749,0.1876989,-2.1931246,
-0.0028882,-0.0038254\PG=C01 [X(C2H3Se1)]\\@

CH_2CHSe^- QCISD

1\1\GINC-ORC161\FOpt\RQCISD-FC\6-311+G(2df,p)\C2H3Se1(1-)\MEISTERP\20-
Aug-2017\0\\# opt freq qcisd/6-311+g(2df,p) guess=read\\CH2CHSe benchm
ark\\-1,1\C,-2.160669,-0.29055,0.\H,-2.088741,-1.375105,-0.000001\H,-3
.158929,0.14603,-0.000023\C,-1.061881,0.486841,0.000013\H,-1.228964,1.
566692,-0.000024\Se,0.759174,-0.044569,-0.000001\\Version=EM64L-G09Rev
E.01\State=1-A\HF=-2477.3075072\MP2=-2477.7878607\MP3=-2477.8143795\MP
4D=-2477.8269818\MP4DQ=-2477.8124357\MP4SDQ=-2477.8171123\QCISD=-2477.
8172326\RMSD=9.149e-09\RMSF=6.191e-05\Dipole=-0.6150788,0.1949297,-0.0
00024\PG=C01 [X(C2H3Se1)]\\@

$CH_2CHSeSeH$ B3LYP

1\1\GINC-ORC380\FOpt\RB3LYP\6-31G(d)\C2H4Se2\MEISTERP\24-Jun-2017\0\\#
opt freq b3lyp/6-31g(d)\\cisCH2CHSeSeH\\0,1\C,-3.7319420055,0.5517207
414,0.2252773444\H,-3.1076850792,-0.3073902248,-0.0054691796\H,-4.6892
626516,0.3508816318,0.7015074598\C,-3.3602036361,1.7967254229,-0.07143
92066\H,-3.9661091997,2.6635998278,0.1776345751\Se,-1.7474311092,2.156
6621352,-1.0506131425\Se,-0.7682093087,3.7606775236,0.3559848\H,-1.298
53758,4.9649952521,-0.3504449005\\Version=EM64L-G16RevA.03\State=1-A\H
F=-4877.3631902\RMSD=6.900e-09\RMSF=3.516e-06\Dipole=-0.5178473,0.0079
935,0.0804358\Quadrupole=0.0297484,2.4043337,-2.4340821,1.2667269,-0.7
466061,-0.2962424\PG=C01 [X(C2H4Se2)]\\@

1\1\GINC-ORC364\FOpt\RB3LYP\6-311G(d)\C2H4Se2\MEISTERP\24-Jun-2017\0\\
opt freq b3lyp/6-311g(d)\\CH2CHSeSeH\\0,1\C,-3.850305856,0.731112730
7,-0.0731159875\H,-3.5372301317,-0.1991662518,-0.5358272872\H,-4.91303
24361,0.8520870241,0.1166627256\C,-2.9889707392,1.6862073044,0.2622980
014\H,-3.3010185925,2.6240333898,0.70970453\Se,-1.0844256428,1.4407407
364,0.1063974353\Se,-0.4622691704,3.4840483,-0.9255181155\H,-0.1064649
513,4.2024000765,0.3267975379\\Version=EM64L-G16RevA.03\State=1-A\HF=-
4881.721677\RMSD=4.772e-09\RMSF=4.384e-06\Dipole=-0.4275607,0.0883701,
0.2627669\Quadrupole=1.0685888,1.1291319,-2.1977207,2.0281338,-0.25487
52,1.1138253\PG=C01 [X(C2H4Se2)]\\@

```
1\1\GINC-ORC363\FOpt\RB3LYP\6-311G(d,p)\C2H4Se2\MEISTERP\24-Jun-2017\0
 \\# opt freq b3lyp/6-311g(d,p)\\CH2CHSeSeH\\0,1\C,-3.8508968716,0.7271
 700092,-0.0699969972\H,-3.5327378751,-0.2114777742,-0.5099049626\H,-4.
 9135191915,0.8542450625,0.1108945942\C,-2.9918184364,1.6894465581,0.24
 96473027\H,-3.302667095,2.6376442956,0.6730721985\Se,-1.0869075444,1.4
 408221938,0.1071660799\Se,-0.4546157594,3.4883843609,-0.9121024715\H,-
 0.1105547467,4.1952286041,0.338623096\\Version=EM64L-G16RevA.03\State=
 1-A\HF=-4881.7303791\RMSD=4.655e-09\RMSF=2.808e-05\Dipole=-0.4162694,0
 .0957657,0.262601\Quadrupole=1.0375093,1.1750283,-2.2125376,1.9962682,
 -0.2152037,1.041523\PG=C01 [X(C2H4Se2)]\\@

1\1\GINC-ORC391\FOpt\RB3LYP\6-311+G(d,p)\C2H4Se2\MEISTERP\24-Jun-2017\
 0\\# opt freq b3lyp/6-311+g(d,p)\\CH2CHSeSeH\\0,1\C,-3.8521923465,0.72
 4960225,-0.0645753298\H,-3.540067393,-0.2148847472,-0.5065903481\H,-4.
 9124572294,0.8534365969,0.1286916989\C,-2.9881546847,1.6878580584,0.24
 53261937\H,-3.2950728141,2.6362493823,0.6716491052\Se,-1.0850013106,1.
 4407507191,0.0836477118\Se,-0.4620803817,3.5013536851,-0.9139910754\H,
 -0.10869136,4.1917393903,0.3432408839\\Version=EM64L-G16RevA.03\State=
 1-A\HF=-4881.7321342\RMSD=4.738e-09\RMSF=2.798e-05\Dipole=-0.405679,0.
 1009782,0.256643\Quadrupole=0.9997609,1.2152994,-2.2150603,1.9930627,-
 0.2110043,1.1241735\PG=C01 [X(C2H4Se2)]\\@

1\1\GINC-ORC384\FOpt\RB3LYP\6-311G(2d,p)\C2H4Se2\MEISTERP\24-Jun-2017\
 0\\# opt freq b3lyp/6-311g(2d,p)\\CH2CHSeSeH\\0,1\C,-3.8498215442,0.72
 64053469,-0.0652957312\H,-3.5396606839,-0.2110004887,-0.5116973124\H,-
 4.9082194347,0.8519032,0.1359677387\C,-2.9882674761,1.6856899224,0.239
 974552\H,-3.2938103408,2.6309512335,0.6713565922\Se,-1.0894253202,1.44
 00619614,0.0687315069\Se,-0.4643512837,3.5124642898,-0.9081779339\H,-0
 .1101614364,4.1849878448,0.3565394277\\Version=EM64L-G16RevA.03\State=
 1-A\HF=-4881.7279469\RMSD=4.690e-09\RMSF=8.679e-06\Dipole=-0.4223878,0
 .0755715,0.2368449\Quadrupole=0.9081958,1.1741241,-2.0823199,1.9571435
 ,-0.285854,0.905663\PG=C01 [X(C2H4Se2)]\\@

1\1\GINC-ORC374\FOpt\RB3LYP\6-311G(df,p)\C2H4Se2\MEISTERP\24-Jun-2017\
 0\\# opt freq b3lyp/6-311g(df,p)\\cisCH2CHSeSeH\\0,1\C,-3.7550991333,0
 .5350993387,0.2009988149\H,-3.1740434,-0.3385043817,-0.0724865228\H,-4
 .7227629211,0.3590403354,0.6593401784\C,-3.325008581,1.7710328046,-0.0
 159956891\H,-3.8937519647,2.6481371595,0.2708300031\Se,-1.6999978306,2
 .1307483018,-0.9745383874\Se,-0.779566326,3.8520786346,0.3512500566\H,
 -1.3191504131,4.980240117,-0.4369607038\\Version=EM64L-G16RevA.03\Stat
 e=1-A\HF=-4881.7388751\RMSD=3.047e-09\RMSF=1.266e-05\Dipole=-0.4882903
 ,0.0379595,0.0522994\Quadrupole=-0.0434521,2.4527394,-2.4092873,1.2567
 934,-0.8055801,-0.2849676\PG=C01 [X(C2H4Se2)]\\@

1\1\GINC-ORC373\FOpt\RB3LYP\6-311+G(2df,p)\C2H4Se2\MEISTERP\24-Jun-201
 7\0\\# opt freq b3lyp/6-311+g(2df,p)\\cisCH2CHSeSeH\\0,1\C,-3.75982592
 87,0.5340140706,0.1948987062\H,-3.1906446644,-0.3429181871,-0.09042907
 73\H,-4.7296412526,0.3653344098,0.6498271609\C,-3.3135657726,1.7665470
 072,-0.0047159634\H,-3.8720134155,2.6453357209,0.2944388474\Se,-1.6848
 366628,2.1217731612,-0.9543733726\Se,-0.7889033558,3.8690580903,0.3512
 231262\H,-1.3299495177,4.9787280371,-0.4584316775\\Version=EM64L-G16Re
 vA.03\State=1-A\HF=-4881.7373359\RMSD=3.051e-09\RMSF=1.359e-05\Dipole=
 -0.4597309,0.0200849,0.0696484\Quadrupole=-0.0710836,2.432837,-2.36175
 35,1.2101002,-0.7454753,-0.205285\PG=C01 [X(C2H4Se2)]\\@

1\1\GINC-ORC363\FOpt\RB3LYP\6-311++G(3df,3pd)\C2H4Se2\MEISTERP\24-Jun-
 2017\0\\# opt freq b3lyp/6-311++g(3df,3pd)\\cisCH2CHSeSeH\\0,1\C,-3.75
```

```
87702406,0.534094313,0.1964958585\H,-3.1885169459,-0.3409068629,-0.086
1757944\H,-4.7266874403,0.3658644945,0.6516104637\C,-3.3155260737,1.76
71106407,-0.0075338935\H,-3.875767622,2.6437895968,0.2888789014\Se,-1.
6881926412,2.1237229988,-0.9587488124\Se,-0.7880487533,3.8659514201,0.
3516146267\H,-1.3278708528,4.9782457091,-0.4537036\\Version=EM64L-G16R
evA.03\State=1-A\HF=-4881.747012\RMSD=3.286e-09\RMSF=1.249e-05\Dipole=
-0.4477493,0.0102804,0.070861\Quadrupole=-0.0650265,2.356703,-2.291676
5,1.2045479,-0.7275766,-0.2099272\PG=C01 [X(C2H4Se2)]\\@
```

$CH_2CHSeSeH$ B3PW91

```
1\1\GINC-ORC277\FOpt\RB3PW91\6-31G(d)\C2H4Se2\MEISTERP\24-Jun-2017\0\\
 # opt freq b3pw91/6-31g(d)\\CH2CHSeSeH\\0,1\C,-3.8346523086,0.74756078
 07,-0.075542279\H,-3.5091798049,-0.1839986952,-0.5317875846\H,-4.90270
 32397,0.8685960886,0.092596722\C,-2.9728459102,1.698761533,0.281537573
 2\H,-3.2899200968,2.6441553111,0.7144104239\Se,-1.0879042902,1.4413363
 91,0.1377364715\Se,-0.4971681213,3.4190607655,-0.9303061974\H,-0.14934
 37483,4.1859911352,0.2987537104\\Version=EM64L-G16RevA.03\State=1-A\HF
 =-4877.2937648\RMSD=2.622e-09\RMSF=6.228e-06\Dipole=-0.4503216,0.09976
 09,0.2728372\Quadrupole=1.0913745,1.1151373,-2.2065118,1.9749662,-0.24
 92738,1.1717711\PG=C01 [X(C2H4Se2)]\\@
```

```
1\1\GINC-ORC248\FOpt\RB3PW91\6-311G(d)\C2H4Se2\MEISTERP\24-Jun-2017\0\
 \# opt freq b3pw91/6-311g(d)\\CH2CHSeSeH\\0,1\C,-3.8433048399,0.728878
 7104,-0.0651403107\H,-3.5304486195,-0.2098794818,-0.5124884234\H,-4.90
 49499501,0.8518829792,0.132130664\C,-2.9822290357,1.6926435349,0.24405
 22802\H,-3.2937774914,2.6386806907,0.6765088938\Se,-1.0912178236,1.449
 0873994,0.0761600953\Se,-0.482387196,3.4884461971,-0.9120528814\H,-0.1
 154025638,4.1817232801,0.3482285222\\Version=EM64L-G16RevA.03\State=1-
 A\HF=-4881.6502608\RMSD=7.785e-09\RMSF=2.714e-06\Dipole=-0.418526,0.10
 14039,0.2805019\Quadrupole=1.0883628,1.206672,-2.2950348,2.0611028,-0.
 2339496,1.187443\PG=C01 [X(C2H4Se2)]\\@
```

```
1\1\GINC-ORC234\FOpt\RB3PW91\6-311G(d,p)\C2H4Se2\MEISTERP\24-Jun-2017\
 0\\# opt freq b3pw91/6-311g(d,p)\\CH2CHSeSeH\\0,1\C,-3.8440199292,0.72
 4701391,-0.0603332252\H,-3.5275891846,-0.220745407,-0.4891112014\H,-4.
 9046633536,0.8520462366,0.1350749705\C,-2.9843931785,1.6950648782,0.23
 11384929\H,-3.2934670452,2.6495676015,0.6444782629\Se,-1.0938252337,1.
 4500011309,0.0666467239\Se,-0.4786031118,3.4983873076,-0.9008602811\H,
 -0.1171564833,4.1724401711,0.3603650976\\Version=EM64L-G16RevA.03\Stat
 e=1-A\HF=-4881.658895\RMSD=3.052e-09\RMSF=8.277e-06\Dipole=-0.4051914,
 0.106209,0.2825379\Quadrupole=1.0521182,1.2381971,-2.2903153,2.0316312
 ,-0.2036087,1.1131645\PG=C01 [X(C2H4Se2)]\\@
```

```
1\1\GINC-ORC215\FOpt\RB3PW91\6-311+G(d,p)\C2H4Se2\MEISTERP\24-Jun-2017
 \0\\# opt freq b3pw91/6-311+g(d,p)\\CH2CHSeSeH\\0,1\C,-3.8440993499,0.
 7215291216,-0.0632027938\H,-3.5208761743,-0.238555348,-0.4530156745\H,
 -4.9085101095,0.8575093559,0.1042738791\C,-2.9882852312,1.7005787706,0
 .2148402735\H,-3.3049853145,2.66866669,0.5895225105\Se,-1.0950403412,1
 .450363904,0.0986305836\Se,-0.455840051,3.4847895664,-0.8820166271\H,-
 0.1260809484,4.1765812495,0.3783666887\\Version=EM64L-G16RevA.03\State
 =1-A\HF=-4881.6602984\RMSD=5.577e-09\RMSF=3.198e-05\Dipole=-0.4024894,
 0.1185183,0.2610206\Quadrupole=1.0241807,1.3306804,-2.3548611,1.970046
 3,-0.1529594,1.1293288\PG=C01 [X(C2H4Se2)]\\@
```

```
1\1\GINC-ORC212\FOpt\RB3PW91\6-311G(2d,p)\C2H4Se2\MEISTERP\24-Jun-2017
 \0\\# opt freq b3pw91/6-311g(2d,p)\\CH2CHSeSeH\\0,1\C,-3.8437505658,0.
```

7240461511,-0.0546694587\H,-3.5353891825,-0.2219110818,-0.4865851641\H
,-4.9001496774,0.8513941713,0.1596271453\C,-2.981499536,1.6920400573,0
.2188313399\H,-3.2838259583,2.6459839838,0.6361839049\Se,-1.0972732756
,1.4481851679,0.0243673132\Se,-0.4858085125,3.523008623,-0.893635735\H
,-0.1160208119,4.1587162374,0.3832794944\\Version=EM64L-G16RevA.03\Sta
te=1-A\HF=-4881.6563202\RMSD=7.691e-09\RMSF=2.898e-06\Dipole=-0.410647
,0.0865828,0.2534816\Quadrupole=0.9085942,1.2537531,-2.1623472,1.98929
16,-0.2770648,0.961035\PG=C01 [X(C2H4Se2)]\\@

1\1\GINC-ORC186\FOpt\RB3PW91\6-311G(df,p)\C2H4Se2\MEISTERP\24-Jun-2017
\0\\# opt freq b3pw91/6-311g(df,p)\\CH2CHSeSeH\\0,1\C,-3.8377877878,0.
7176366446,-0.0580262211\H,-3.5086616704,-0.2529771937,-0.4147782439\H
,-4.9046076397,0.8542802057,0.0906953296\C,-2.9892333352,1.7038720656,
0.2006333797\H,-3.3128111761,2.6821572425,0.5409885388\Se,-1.099103024
9,1.4588542774,0.1127344095\Se,-0.4487412106,3.4734471235,-0.870890444
8\H,-0.1427716752,4.1841929444,0.3860420922\\Version=EM64L-G16RevA.03\
State=1-A\HF=-4881.667425\RMSD=3.689e-09\RMSF=2.420e-05\Dipole=-0.4027
779,0.1188933,0.2642141\Quadrupole=1.0508487,1.257182,-2.3080308,1.864
3956,-0.1314145,1.1008931\PG=C01 [X(C2H4Se2)]\\@

1\1\GINC-ORC181\FOpt\RB3PW91\6-311+G(2df,p)\C2H4Se2\MEISTERP\24-Jun-20
17\0\\# opt freq b3pw91/6-311+g(2df,p)\\CH2CHSeSeH\\0,1\C,-3.843833626
9,0.7200346074,-0.0417634404\H,-3.5433469222,-0.2366234676,-0.45563017
74\H,-4.897261626,0.8533656539,0.1824684522\C,-2.9762428611,1.69310020
2,0.1983871224\H,-3.273364411,2.6570779422,0.5970590925\Se,-1.09845481
74,1.4526430665,-0.0233043214\Se,-0.4966371008,3.5382738901,-0.8793415
708\H,-0.1145761546,4.1435914155,0.4095236829\\Version=EM64L-G16RevA.0
3\State=1-A\HF=-4881.66542\RMSD=3.971e-09\RMSF=5.695e-06\Dipole=-0.379
711,0.0916329,0.2550118\Quadrupole=0.9153396,1.1761882,-2.0915278,1.88
28931,-0.2414926,1.0991726\PG=C01 [X(C2H4Se2)]\\@

1\1\GINC-ORC180\FOpt\RB3PW91\6-311++G(3df,3pd)\C2H4Se2\MEISTERP\24-Jun
-2017\0\\# opt freq b3pw91/6-311++g(3df,3pd)\\CH2CHSeSeH\\0,1\C,-3.726
3239241,0.7614822463,0.2136732447\H,-3.2513260161,-0.1892731816,0.4237
920429\H,-4.7801140449,0.8512905235,0.4508310051\C,-3.0694676873,1.780
3307184,-0.3228244014\H,-3.5354074199,2.7382387189,-0.5211533571\Se,-1
.260995855,1.6308380377,-0.9055918907\Se,-0.4695788108,3.760480702,-0.
3822951488\H,-0.1505037619,3.4880755449,1.0309673454\\Version=EM64L-G1
6RevA.03\State=1-A\HF=-4881.6752767\RMSD=2.951e-09\RMSF=8.234e-06\Dipo
le=-0.3576947,-0.0680862,0.3382745\Quadrupole=0.845525,0.3729773,-1.21
85023,1.2503038,0.081662,-0.1209905\PG=C01 [X(C2H4Se2)]\\@

$CH_2CHSeSeH$ wB97XD

1\1\GINC-ORC21\FOpt\RwB97XD\6-31G(d)\C2H4Se2\MEISTERP\10-Aug-2017\0\\#
opt freq wb97xd/6-31g(d)\\CH2CHSeSeH\\0,1\C,-3.8249729232,0.761147337
7,-0.0821015206\H,-3.4992900458,-0.156844422,-0.5638899827\H,-4.892796
2411,0.8836908531,0.0788800988\C,-2.9633074047,1.6942905079,0.30897460
7\H,-3.282748186,2.6256226947,0.7679373903\Se,-1.0791092752,1.43640427
9,0.1591710307\Se,-0.5184507395,3.3927084801,-0.9462278378\H,-0.183042
7045,4.1844435796,0.2646550544\\Version=EM64L-G09RevE.01\State=1-A\HF=
-4877.3672851\RMSD=8.445e-09\RMSF=1.750e-05\Dipole=-0.473418,0.1030797
,0.2693472\Quadrupole=1.109503,1.1013547,-2.2108577,2.0052614,-0.27441
95,1.2293722\PG=C01 [X(C2H4Se2)]\\@

1\1\GINC-ORC14\FOpt\RwB97XD\6-311G(d)\C2H4Se2\MEISTERP\10-Aug-2017\0\\
opt freq wb97xd/6-311g(d)\\CH2CHSeSeH\\0,1\C,-3.8384031835,0.7358462

6,-0.0651376644\H,-3.5261721675,-0.2086664518,-0.4999099135\H,-4.90032
58695,0.8685722534,0.1182765048\C,-2.9755161671,1.6937977891,0.2441892
965\H,-3.2882792301,2.6442358156,0.6642748202\Se,-1.0848590302,1.44263
06355,0.0855828248\Se,-0.4895949312,3.4725037989,-0.907967333\H,-0.140
5669409,4.1725432093,0.3480903045\\Version=EM64L-G09RevE.01\State=1-A\
HF=-4881.7367067\RMSD=4.916e-09\RMSF=1.532e-05\Dipole=-0.425781,0.1106
466,0.2767586\Quadrupole=1.0870569,1.2641098,-2.3511667,2.0507688,-0.2
278361,1.2031503\PG=C01 [X(C2H4Se2)]\\@

1\1\GINC-ORC12\FOpt\RwB97XD\6-311G(d,p)\C2H4Se2\MEISTERP\10-Aug-2017\0
\\# opt freq wb97xd/6-311g(d,p)\\CH2CHSeSeH\\0,1\C,-3.8607943997,0.756
49861,-0.0432607412\H,-3.539748976,-0.239190688,-0.3308665836\H,-4.928
4462219,0.9314332452,0.0387770007\C,-2.998600906,1.7307852901,0.211213
5545\H,-3.3134359763,2.732325587,0.4831785843\Se,-1.1095664818,1.42760
98297,0.2323783813\Se,-0.3798950777,3.3374123969,-0.9017895138\H,-0.11
32294806,4.1445890391,0.2977681577\\Version=EM64L-G09RevE.01\State=1-A
\HF=-4881.7452076\RMSD=5.277e-09\RMSF=8.851e-06\Dipole=-0.4301371,0.15
05018,0.2311278\Quadrupole=1.1738947,1.410208,-2.5841027,1.9311901,-0.
1133049,0.8683213\PG=C01 [X(C2H4Se2)]\\@

1\1\GINC-ORC12\FOpt\RwB97XD\6-311+G(d,p)\C2H4Se2\MEISTERP\10-Aug-2017\
0\\# opt freq wb97xd/6-311+g(d,p)\\CH2CHSeSeH\\0,1\C,-3.8382515612,0.7
289611763,-0.0575715275\H,-3.5261792551,-0.2195530626,-0.4823528912\H,
-4.8977055521,0.8646131777,0.1337377274\C,-2.9742488503,1.6934322927,0
.23131796\H,-3.2835011245,2.649309201,0.6403921512\Se,-1.0853444085,1.
444951807,0.057366125\Se,-0.4947448236,3.4950308287,-0.8982248708\H,-0
.1437419446,4.1647178892,0.362734166\\Version=EM64L-G09RevE.01\State=1
-A\HF=-4881.7466193\RMSD=4.187e-09\RMSF=2.120e-05\Dipole=-0.4068935,0.
1163216,0.27328\Quadrupole=1.0303015,1.3093375,-2.339639,2.0333459,-0.
2109496,1.1906721\PG=C01 [X(C2H4Se2)]\\@

1\1\GINC-ORC7\FOpt\RwB97XD\6-311G(2d,p)\C2H4Se2\MEISTERP\10-Aug-2017\0
\\# opt freq wb97xd/6-311g(2d,p)\\CH2CHSeSeH\\0,1\C,-3.8372695546,0.73
08786173,-0.0567517764\H,-3.5312521028,-0.2132769954,-0.4933004371\H,-
4.8928726366,0.8634725502,0.1528662545\C,-2.9721420648,1.6895568541,0.
225623868\H,-3.2756570767,2.6402068426,0.6480934232\Se,-1.0891525599,1
.4431762355,0.0255299416\Se,-0.5004809847,3.5124765021,-0.8934694328\H
,-0.1448905399,4.1549727035,0.3788069991\\Version=EM64L-G09RevE.01\Sta
te=1-A\HF=-4881.7424752\RMSD=6.340e-09\RMSF=1.147e-05\Dipole=-0.417526
6,0.0931001,0.2533879\Quadrupole=0.9032698,1.2929001,-2.1961699,2.0088
659,-0.2972268,0.9882356\PG=C01 [X(C2H4Se2)]\\@

1\1\GINC-ORC3\FOpt\RwB97XD\6-311G(df,p)\C2H4Se2\MEISTERP\10-Aug-2017\0
\\# opt freq wb97xd/6-311g(df,p)\\CH2CHSeSeH\\0,1\C,-3.8380213303,0.73
18023359,-0.0517694663\H,-3.526401638,-0.2270271098,-0.4523570391\H,-4
.8982233403,0.8720977749,0.1300109786\C,-2.9752216157,1.6982069157,0.2
189138675\H,-3.2849380282,2.6634770686,0.6043992661\Se,-1.0902806064,1
.4452363467,0.0599268546\Se,-0.4815670674,3.477587574,-0.8911578526\H,
-0.1490638936,4.1600824038,0.3694322311\\Version=EM64L-G09RevE.01\Stat
e=1-A\HF=-4881.7539959\RMSD=3.484e-09\RMSF=1.849e-05\Dipole=-0.4071625
,0.1167608,0.2792723\Quadrupole=1.0671162,1.1957502,-2.2628665,1.91828
54,-0.2047423,1.1834451\PG=C01 [X(C2H4Se2)]\\@

1\1\GINC-ORC3\FOpt\RwB97XD\6-311+G(2df,p)\C2H4Se2\MEISTERP\10-Aug-2017
\0\\# opt freq wb97xd/6-311+g(2df,p)\\CH2CHSeSeH\\0,1\C,-3.8372208353,
0.7305111983,-0.0521630431\H,-3.5331775565,-0.2220969241,-0.4716550141
\H,-4.8929991024,0.8684177378,0.1527109402\C,-2.9697638543,1.692743248

7,0.2144138362\H,-3.2735704906,2.6514188279,0.6192461258\Se,-1.0899159352,1.4455229946,0.0174738403\Se,-0.496356302,3.5042404344,-0.8833670897\H,-0.1507134437,4.1507057923,0.3907392445\\Version=EM64L-G09RevE.01\State=1-A\HF=-4881.7519349\RMSD=6.063e-09\RMSF=1.616e-05\Dipole=-0.4018546,0.0990626,0.2482511\Quadrupole=0.954388,1.2090066,-2.1633946,1.9300818,-0.2722309,1.1393358\PG=C01 [X(C2H4Se2)]\\@

1\1\GINC-ORC181\FOpt\RwB97XD\6-311++G(3df,3pd)\C2H4Se2\MEISTERP\24-Jun-2017\0\\# opt freq wb97xd/6-311++g(3df,3pd)\\cisCH2CHSeSeH\\0,1\C,-3.7519809261,0.5472893157,0.1930509652\H,-3.185855857,-0.3297207876,-0.0940416921\H,-4.7189319277,0.3862444486,0.652339032\C,-3.3022043424,1.7746403701,-0.0088367577\H,-3.8604810345,2.6522016707,0.2911702412\Se,-1.6790991545,2.1187126672,-0.9478194936\Se,-0.8125050012,3.8425535154,0.3490281597\H,-1.3583223267,4.94595111,-0.4524527047\\Version=EM64L-G16RevA.03\State=1-A\HF=-4881.7618992\RMSD=4.679e-09\RMSF=3.548e-05\Dipole=-0.4655402,0.0277359,0.073039\Quadrupole=-0.0583367,2.5109793,-2.4526425,1.2526946,-0.7909444,-0.2130691\PG=C01 [X(C2H4Se2)]\\@

CH_2CHSeSeH M062X

1\1\GINC-ORC372\FOpt\RM062X\6-31G(d)\C2H4Se2\MEISTERP\24-Jun-2017\0\\# opt freq m062x/6-31g(d)\\CH2CHSeSeH\\0,1\C,-3.8250384715,0.7560222767,-0.086115372\H,-3.4870687889,-0.1770451646,-0.5272827737\H,-4.8950492739,0.8844926337,0.0499686432\C,-2.9705554564,1.7026845861,0.2858371478\H,-3.289456916,2.6530530945,0.70303628\Se,-1.0865755387,1.4361264275,0.1723278398\Se,-0.5144399576,3.3956448488,-0.9152202537\H,-0.1755331171,4.1704846073,0.3048473285\\Version=EM64L-G16RevA.03\State=1-A\HF=-4877.4004334\RMSD=2.364e-09\RMSF=7.612e-06\Dipole=-0.471553,0.1143436,0.2712409\Quadrupole=1.1551407,1.1233967,-2.2785373,2.0022131,-0.1611679,1.2413406\PG=C01 [X(C2H4Se2)]\\@

1\1\GINC-ORC363\FOpt\RM062X\6-311G(d)\C2H4Se2\MEISTERP\24-Jun-2017\0\\# opt freq m062x/6-311g(d)\\CH2CHSeSeH\\0,1\C,-3.8356625681,0.739009492,-0.0796903446\H,-3.5358434039,-0.1656874839,-0.5973268463\H,-4.8885017097,0.8519457096,0.1567024588\C,-2.9632781121,1.6768735694,0.2623636511\H,-3.2519408768,2.5934400688,0.7643939626\Se,-1.0818225025,1.442089151,-0.0025515777\Se,-0.5562846256,3.5356292736,-0.8968797648\H,-0.1303837214,4.1481635295,0.3803873009\\Version=EM64L-G16RevA.03\State=1-A\HF=-4881.6759059\RMSD=8.314e-09\RMSF=5.686e-06\Dipole=-0.4258882,0.0935702,0.3171111\Quadrupole=1.1303424,1.0620348,-2.1923772,2.1998394,-0.1916447,1.4006562\PG=C01 [X(C2H4Se2)]\\@

1\1\GINC-ORC362\FOpt\RM062X\6-311G(d,p)\C2H4Se2\MEISTERP\24-Jun-2017\0\\# opt freq m062x/6-311g(d,p)\\CH2CHSeSeH\\0,1\C,-3.8285612973,0.7267705316,-0.0725919858\H,-3.5153003266,-0.1870698507,-0.5643856798\H,-4.8831306616,0.8392806885,0.1531591561\C,-2.966410394,1.6796824306,0.2527837282\H,-3.2632605212,2.6070277234,0.7282964347\Se,-1.081763801,1.4516100067,0.0075481959\Se,-0.5557865669,3.5428344769,-0.8940639184\H,-0.1495039515,4.161327303,0.3766529092\\Version=EM64L-G16RevA.03\State=1-A\HF=-4881.6830887\RMSD=7.015e-09\RMSF=6.580e-06\Dipole=-0.4226134,0.0985778,0.3166473\Quadrupole=1.0942226,1.1499405,-2.244163,2.1654296,-0.1890955,1.3264767\PG=C01 [X(C2H4Se2)]\\@

1\1\GINC-ORC390\FOpt\RM062X\6-311+G(d,p)\C2H4Se2\MEISTERP\24-Jun-2017\0\\# opt freq m062x/6-311+g(d,p)\\CH2CHSeSeH\\0,1\C,-3.8273666814,0.7229673488,-0.0585388053\H,-3.5278791035,-0.1822105101,-0.5746128756\H,-4.8748534052,0.8301427666,0.2006700082\C,-2.9566635358,1.6730468921,0.

2568416263\H,-3.2419656385,2.5917406833,0.7561269443\Se,-1.0799479153,
1.455712704,-0.0480049833\Se,-0.5848451715,3.5704391538,-0.9102012065\
H,-0.1501960687,4.1596242715,0.3651181319\\Version=EM64L-G16RevA.03\St
ate=1-A\HF=-4881.6844752\RMSD=3.131e-09\RMSF=2.196e-05\Dipole=-0.40573
4,0.0951931,0.3199566\Quadrupole=1.0407601,1.1232754,-2.1640355,2.1994
149,-0.2432909,1.391963\PG=C01 [X(C2H4Se2)]\\@

1\1\GINC-ORC389\FOpt\RM062X\6-311G(2d,p)\C2H4Se2\MEISTERP\24-Jun-2017\
0\\# opt freq m062x/6-311g(2d,p)\\CH2CHSeSeH\\0,1\C,-3.8304931948,0.72
86945689,-0.0734890946\H,-3.5151125919,-0.2004742528,-0.5321821539\H,-
4.8861309203,0.8510796555,0.1375431168\C,-2.970778264,1.6855387348,0.2
309855998\H,-3.2700063482,2.6265543395,0.6748656408\Se,-1.0870120457,1
.4450047857,0.0205356462\Se,-0.5246691803,3.5323683312,-0.8759545161\H
,-0.1595149748,4.1526971473,0.4050946009\\Version=EM64L-G16RevA.03\Sta
te=1-A\HF=-4881.6818996\RMSD=6.445e-09\RMSF=1.693e-05\Dipole=-0.435131
9,0.0916856,0.2722134\Quadrupole=0.9598782,1.241436,-2.2013142,2.08380
74,-0.2456079,1.13494\PG=C01 [X(C2H4Se2)]\\@

1\1\GINC-ORC256\FOpt\RM062X\6-311G(df,p)\C2H4Se2\MEISTERP\24-Jun-2017\
0\\# opt freq m062x/6-311g(df,p)\\CH2CHSeSeH\\0,1\C,-3.8225283398,0.72
07242029,-0.0806020325\H,-3.4930554768,-0.2222081572,-0.5023048502\H,-
4.8855132649,0.8449910703,0.0938616301\C,-2.9735354826,1.6901434629,0.
2206338312\H,-3.29003665,2.6441393319,0.6263936863\Se,-1.0849947992,1.
4561975893,0.0664110096\Se,-0.509780985,3.5125298129,-0.8552691041\H,-
0.1842725216,4.174945997,0.4182746695\\Version=EM64L-G16RevA.03\State=
1-A\HF=-4881.6918301\RMSD=8.330e-09\RMSF=6.791e-06\Dipole=-0.4296752,0
.1102877,0.2934198\Quadrupole=1.1015145,1.1595339,-2.2610485,1.9742013
,-0.132961,1.3474682\PG=C01 [X(C2H4Se2)]\\@

1\1\GINC-ORC251\FOpt\RM062X\6-311+G(2df,p)\C2H4Se2\MEISTERP\24-Jun-201
7\0\\# opt freq m062x/6-311+g(2df,p)\\CH2CHSeSeH\\0,1\C,-3.8333591036,
0.7276632972,-0.0704871577\H,-3.5168200799,-0.223103022,-0.4825743973\
H,-4.8917974871,0.8633391774,0.117884501\C,-2.9730679698,1.6940247847,
0.2041443645\H,-3.2760642665,2.6556180819,0.6002496022\Se,-1.09039457,
1.4448917107,0.0257753165\Se,-0.5001012685,3.5168963762,-0.8474671206\
H,-0.1621127745,4.142132904,0.4398737315\\Version=EM64L-G16RevA.03\Sta
te=1-A\HF=-4881.6913217\RMSD=4.960e-09\RMSF=2.547e-05\Dipole=-0.413304
9,0.0992209,0.2581026\Quadrupole=1.0209078,1.1769696,-2.1978774,1.9558
456,-0.1888264,1.2514509\PG=C01 [X(C2H4Se2)]\\@

1\1\GINC-ORC234\FOpt\RM062X\6-311++G(3df,3pd)\C2H4Se2\MEISTERP\24-Jun-
2017\0\\# opt freq m062x/6-311++g(3df,3pd)\\CH2CHSeSeH\\0,1\C,-3.83303
52699,0.7294857458,-0.0754512344\H,-3.5127990275,-0.2220108352,-0.4779
678605\H,-4.8918045716,0.8682613461,0.0977369804\C,-2.9745998537,1.695
2921742,0.2073258808\H,-3.2818839047,2.6570862792,0.5947635645\Se,-1.0
899464986,1.4436293807,0.0573587888\Se,-0.4919218019,3.5010021339,-0.8
473719048\H,-0.1677265921,4.1487170852,0.4310046252\\Version=EM64L-G16
RevA.03\State=1-A\HF=-4881.7008678\RMSD=5.377e-09\RMSF=1.373e-05\Dipol
e=-0.4069639,0.0942811,0.2393469\Quadrupole=1.0263215,1.1519857,-2.178
3072,1.909661,-0.1670218,1.186845\PG=C01 [X(C2H4Se2)]\\@

CH_2CHSeSeH M08HX

1\1\GINC-ORC215\FOpt\RM08HX\6-31G(d)\C2H4Se2\MEISTERP\24-Jun-2017\0\\#
opt freq m08hx/6-31g(d)\\CH2CHSeSeH\\0,1\C,-3.8288580097,0.6972772681
,0.0122358245\H,-3.5380478284,-0.3191255596,-0.2571757681\H,-4.8767384
613,0.8635196457,0.2618632274\C,-2.9591142898,1.7008059579,0.048714848

1\H,-3.236828212,2.7266122213,0.2961103048\Se,-1.0974511407,1.47160231
71,-0.310754213\Se,-0.559817892,3.6752778595,-0.726552683\H,-0.1468616
862,4.0054936,0.6629572992\\Version=EM64L-G16RevA.03\State=1-A\HF=-487
7.4591318\RMSD=6.894e-09\RMSF=1.338e-05\Dipole=-0.3329768,0.0896277,0.
3567563\Quadrupole=0.871497,1.2083406,-2.0798376,1.6853907,-0.0678756,
1.1322728\PG=C01 [X(C2H4Se2)]\\@

1\1\GINC-ORC212\FOpt\RM08HX\6-311G(d)\C2H4Se2\MEISTERP\24-Jun-2017\0\\
opt freq m08hx/6-311g(d)\\CH2CHSeSeH\\0,1\C,-3.8324051054,0.72482421
68,-0.0691126746\H,-3.5088577391,-0.2289826865,-0.4841821914\H,-4.8991
662906,0.8566050474,0.1080434988\C,-2.9753264343,1.6948002003,0.219688
3615\H,-3.2834926607,2.6605511337,0.6176722922\Se,-1.0833210776,1.4466
459262,0.0573122946\Se,-0.5234552587,3.5122480156,-0.8698581472\H,-0.1
376929536,4.1547714565,0.4078354061\\Version=EM64L-G16RevA.03\State=1-
A\HF=-4881.6959085\RMSD=6.323e-09\RMSF=1.236e-05\Dipole=-0.4128528,0.1
094191,0.296473\Quadrupole=1.1088842,1.2801174,-2.3890016,2.0560023,-0
.0722648,1.2481329\PG=C01 [X(C2H4Se2)]\\@

1\1\GINC-ORC186\FOpt\RM08HX\6-311G(d,p)\C2H4Se2\MEISTERP\24-Jun-2017\0
\\# opt freq m08hx/6-311g(d,p)\\CH2CHSeSeH\\0,1\C,-3.8411914127,0.7275
04356,-0.0507156144\H,-3.5218265755,-0.2395999385,-0.4349424156\H,-4.9
052020264,0.873076866,0.126243108\C,-2.9781604062,1.7016163629,0.20426
54104\H,-3.2763299254,2.6820142861,0.5705115441\Se,-1.088575603,1.4387
403009,0.0412865817\Se,-0.5102464947,3.5063236132,-0.8723411814\H,-0.1
22185076,4.1317874636,0.4030914072\\Version=EM64L-G16RevA.03\State=1-A
\HF=-4881.703068\RMSD=8.340e-09\RMSF=6.430e-05\Dipole=-0.3992101,0.117
1035,0.2981715\Quadrupole=1.1058127,1.2961623,-2.401975,2.0234123,-0.0
637602,1.1358754\PG=C01 [X(C2H4Se2)]\\@

1\1\GINC-ORC181\FOpt\RM08HX\6-311+G(d,p)\C2H4Se2\MEISTERP\24-Jun-2017\
0\\# opt freq m08hx/6-311+g(d,p)\\CH2CHSeSeH\\0,1\C,-3.8331923521,0.71
4410619,-0.0460249313\H,-3.4998167126,-0.2698152554,-0.370867415\H,-4.
9026408967,0.8635285822,0.0915989718\C,-2.9814577819,1.7059916727,0.18
39947917\H,-3.2955431394,2.7023352394,0.4899768016\Se,-1.0868419475,1.
4501531057,0.0888522558\Se,-0.4909283103,3.4957049611,-0.8604901219\H,
-0.1532963795,4.1591543853,0.4103584874\\Version=EM64L-G16RevA.03\Stat
e=1-A\HF=-4881.7048243\RMSD=3.837e-09\RMSF=1.523e-05\Dipole=-0.3911388
,0.1294726,0.2706405\Quadrupole=1.0513911,1.4370047,-2.4883958,1.95868
78,-0.0266055,1.1030234\PG=C01 [X(C2H4Se2)]\\@

1\1\GINC-ORC389\FOpt\RM08HX\6-311G(2d,p)\C2H4Se2\MEISTERP\24-Jun-2017\
0\\# opt freq m08hx/6-311g(2d,p)\\cisCH2CHSeSeH\\0,1\C,-3.7637099611,0
.5459764288,0.1775177863\H,-3.2172000217,-0.3365789418,-0.1468699996\H
,-4.7388628763,0.3938736231,0.6339299793\C,-3.279274145,1.7662587412,0
.0225362828\H,-3.8010850969,2.6605004054,0.3542279603\Se,-1.6399136275
,2.0904428631,-0.9042648754\Se,-0.8427252642,3.8837973991,0.3612371508
\H,-1.3866095774,4.9336017911,-0.5158765344\\Version=EM64L-G16RevA.03\
State=1-A\HF=-4881.7027552\RMSD=2.455e-09\RMSF=2.594e-05\Dipole=-0.483
2119,0.0464488,0.0483539\Quadrupole=-0.2107242,2.6070423,-2.3963182,1.
328891,-0.7710985,-0.0686662\PG=C01 [X(C2H4Se2)]\\@

1\1\GINC-ORC300\FOpt\RM08HX\6-311G(df,p)\C2H4Se2\MEISTERP\24-Jun-2017\
0\\# opt freq m08hx/6-311g(df,p)\\cisCH2CHSeSeH\\0,1\C,-3.763635856,0.
5459092822,0.1762625522\H,-3.2192978712,-0.3391316832,-0.1487511831\H,
-4.738748451,0.392898693,0.6348634734\C,-3.2777872874,1.7673281965,0.0
213154366\H,-3.7998816073,2.6619210933,0.3558877861\Se,-1.6360347783,2
.0929144012,-0.8986714949\Se,-0.8483898621,3.8793204534,0.3587077363\H

```
,-1.3856048566,4.9367118736,-0.5171765566\\Version=EM64L-G16RevA.03\St
ate=1-A\HF=-4881.7124743\RMSD=3.142e-09\RMSF=2.195e-05\Dipole=-0.49932
02,0.0742891,0.0182028\Quadrupole=-0.140601,2.5916147,-2.4510137,1.205
1337,-0.9236183,-0.3128252\PG=C01 [X(C2H4Se2)]\\@
```

```
1\1\GINC-ORC105\FOpt\RM08HX\6-311+G(2df,p)\C2H4Se2\MEISTERP\24-Jun-201
7\0\\# opt freq m08hx/6-311+g(2df,p)\\CH2CHSeSeH\\0,1\C,-3.8419699971,
0.7215799394,-0.0244179481\H,-3.5391637453,-0.2674396243,-0.3616883557
\H,-4.8989526833,0.8801509638,0.1763319648\C,-2.9714350205,1.702806002
9,0.1470092003\H,-3.2582485073,2.7023112692,0.4665633273\Se,-1.0939899
093,1.439242172,-0.0649730725\Se,-0.4995980931,3.5460776434,-0.8349885
511\H,-0.1403595641,4.0967349436,0.483562275\\Version=EM64L-G16RevA.03
\State=1-A\HF=-4881.713106\RMSD=4.727e-09\RMSF=7.963e-06\Dipole=-0.356
5705,0.1047163,0.2643602\Quadrupole=0.9119134,1.278682,-2.1905954,1.82
06283,-0.1559055,1.086989\PG=C01 [X(C2H4Se2)]\\@
```

```
1\1\GINC-ORC88\FOpt\RM08HX\6-311++G(3df,3pd)\C2H4Se2\MEISTERP\24-Jun-2
017\0\\# opt freq m08hx/6-311++g(3df,3pd)\\CH2CHSeSeH\\0,1\C,-3.713096
0639,0.7453718839,0.2067734218\H,-3.2416716262,-0.2231194023,0.3400933
39\H,-4.7560063879,0.8385137235,0.4915872242\C,-3.0594326924,1.7821925
087,-0.2916125286\H,-3.5083468906,2.7627930755,-0.4171527959\Se,-1.266
4653323,1.6413615555,-0.926733216\Se,-0.5239235409,3.7782068486,-0.408
6082937\H,-0.1747749859,3.4961431167,0.9930516891\\Version=EM64L-G16Re
vA.03\State=1-A\HF=-4881.723003\RMSD=4.111e-09\RMSF=2.003e-05\Dipole=-
0.3331224,-0.0654203,0.3632662\Quadrupole=0.8057749,0.4496483,-1.25542
31,1.2695752,-0.0053311,-0.0022328\PG=C01 [X(C2H4Se2)]\\@
```

$CH_2CHSeSeH$ QCISD

```
1\1\GINC-GRA131\FOpt\RQCISD-FC\6-311+G(2df,p)\C2H4Se2\MEISTERP\15-Nov-
2018\0\\# opt freq=noraman qcisd/6-311+g(2df,p)\\Optimization and freq
uency on CH2=CH-Se-Se-H\\0,1\C,-3.5420504544,1.7268856898,-0.002065068
1\H,-3.2436892328,0.6832558767,-0.0040975661\H,-4.6059356147,1.9366894
55,-0.0016551853\C,-2.6620207009,2.725064026,-0.0001223299\H,-2.983224
0757,3.7622134813,0.0018973991\Se,-0.7748606611,2.4476967636,-0.000660
327\Se,-0.2216609485,4.761326533,0.0037836637\H,1.2181383781,4.4689654
545,0.0032711536\\Version=EM64L-G09RevE.01\State=1-A\HF=-4877.6620216\
MP2=-4878.2815516\MP3=-4878.3243288\MP4D=-4878.3415145\MP4DQ=-4878.322
2644\MP4SDQ=-4878.3275045\QCISD=-4878.3277316\RMSD=8.515e-09\RMSF=6.32
4e-05\Dipole=-0.1326051,-0.0249023,-0.0000376\PG=C01 [X(C2H4Se2)]\\@
```

$CH_2CHSeSe^-$ B3LYP

```
1\1\GINC-ORC362\FOpt\RB3LYP\6-31G(d)\C2H3Se2(1-)\MEISTERP\11-Aug-2017\
0\\# opt freq b3lyp/6-31g(d) guess=read\\CH2CHSeSeH\\-1,1\C,-3.0526034
375,0.770601223,-0.0000161632\H,-3.6158538785,-0.1613459627,-0.0000647
515\H,-3.6371174262,1.6896419804,0.000002771\C,-1.710009884,0.79941750
99,0.0000049852\H,-1.16904447,1.7452667358,0.0000387096\Se,-0.52255238
92,-0.693944769,-0.0000302929\Se,1.6102854853,0.3202382826,0.000103741
8\\Version=EM64L-G09RevE.01\State=1-A\HF=-4876.8107731\RMSD=6.727e-09\
RMSF=2.593e-05\Dipole=-1.0159155,-0.1202413,-0.0000529\Quadrupole=-5.5
626913,4.0301672,1.5325241,-0.1192532,-0.0002223,0.0000688\PG=C01 [X(C
2H3Se2)]\\@
```

```
1\1\GINC-ORC361\FOpt\RB3LYP\6-311G(d)\C2H3Se2(1-)\MEISTERP\11-Aug-2017
\0\\# opt freq b3lyp/6-311g(d) guess=read\\CH2CHSeSeH\\-1,1\C,-3.05573
92442,0.770558953,-0.0000233011\H,-3.6172271833,-0.1609009918,-0.00007
```

13737\H,-3.6385680813,1.688914693,-0.000004735\C,-1.7171224053,0.80364
73339,0.0000103927\H,-1.1775479967,1.7478888974,0.0000577484\Se,-0.526
5152097,-0.6964936255,-0.0000121045\Se,1.6358241204,0.31625974,0.00008
23732\\Version=EM64L-G09RevE.01\State=1-A\HF=-4881.1851062\RMSD=8.443e
-09\RMSF=8.785e-05\Dipole=-1.121301,-0.1256981,-0.0000345\Quadrupole=-
5.3269219,4.1531589,1.1737629,-0.1272774,-0.0001757,0.000102\PG=C01 [X
(C2H3Se2)]\\@

1\1\GINC-ORC264\FOpt\RB3LYP\6-311G(d,p)\C2H3Se2(1-)\MEISTERP\11-Aug-20
17\0\\# opt freq b3lyp/6-311g(d,p) guess=read\\CH2CHSeSeH\\-1,1\C,-3.0
562611193,0.7702953295,-0.0000242192\H,-3.6142344152,-0.1618615294,-0.
0000727649\H,-3.6373977588,1.6883998501,-0.0000066738\C,-1.7175651243,
0.805068436,0.0000129843\H,-1.1753461361,1.7467961654,0.0000604086\Se,
-0.5292811569,-0.695739141,-0.0000094421\Se,1.6331897107,0.3169158893,
0.000078707\\Version=EM64L-G09RevE.01\State=1-A\HF=-4881.1907294\RMSD=
4.119e-09\RMSF=1.672e-05\Dipole=-1.1146769,-0.1307228,-0.0000321\Quadr
upole=-5.398166,4.10367,1.2944959,-0.0937383,-0.000169,0.0000983\PG=C0
1 [X(C2H3Se2)]\\@

1\1\GINC-ORC83\FOpt\RB3LYP\6-311+G(d,p)\C2H3Se2(1-)\MEISTERP\11-Aug-20
17\0\\# opt freq b3lyp/6-311+g(d,p) guess=read\\CH2CHSeSeH\\-1,1\C,-3.
0556171611,0.7696312751,-0.0000240226\H,-3.6151217005,-0.1613941668,-0
.0000725037\H,-3.6337689963,1.6891718886,-0.0000063411\C,-1.714616219,
0.8033115559,0.0000127195\H,-1.1730977805,1.745462011,0.000059623\Se,-
0.5309283231,-0.6984510951,-0.0000100022\Se,1.6262541805,0.3221435314,
0.0000795271\\Version=EM64L-G09RevE.01\State=1-A\HF=-4881.1989578\RMSD
=8.654e-09\RMSF=1.554e-05\Dipole=-1.2707889,-0.1486862,-0.0000374\Quad
rupole=-5.5068406,4.3532646,1.153576,-0.1580769,-0.0001708,0.0001101\P
G=C01 [X(C2H3Se2)]\\@

1\1\GINC-ORC61\FOpt\RB3LYP\6-311G(2d,p)\C2H3Se2(1-)\MEISTERP\11-Aug-20
17\0\\# opt freq b3lyp/6-311g(2d,p) guess=read\\CH2CHSeSeH\\-1,1\C,-3.
0532800746,0.7700526101,-0.000022963\H,-3.6128356197,-0.1603023227,-0.
0000700737\H,-3.6321487498,1.6885296156,-0.0000030548\C,-1.7189705679,
0.8028115549,0.0000091843\H,-1.1796251736,1.74518366,0.0000551278\Se,-
0.532499414,-0.6957621231,-0.0000141041\Se,1.6324635997,0.3193620052,0
.0000848836\\Version=EM64L-G09RevE.01\State=1-A\HF=-4881.187542\RMSD=7
.604e-09\RMSF=9.952e-05\Dipole=-1.0900293,-0.1068006,-0.0000344\Quadru
pole=-5.3310839,4.0054859,1.325598,-0.0577255,-0.0001858,0.0000924\PG=
C01 [X(C2H3Se2)]\\@

1\1\GINC-ORC29\FOpt\RB3LYP\6-311G(df,p)\C2H3Se2(1-)\MEISTERP\11-Aug-20
17\0\\# opt freq b3lyp/6-311g(df,p) guess=read\\CH2CHSeSeH\\-1,1\C,-3.
0525842358,0.7697363859,-0.0000235308\H,-3.6122608723,-0.1613325563,-0
.0000704845\H,-3.6340535573,1.6873996062,-0.0000046424\C,-1.7161911931
,0.8028267975,0.0000098686\H,-1.1756210595,1.7457152443,0.0000574306\S
e,-0.5277514429,-0.6903332715,-0.0000127387\Se,1.6215663609,0.31586279
39,0.0000830972\\Version=EM64L-G09RevE.01\State=1-A\HF=-4881.1990272\R
MSD=7.268e-09\RMSF=5.702e-05\Dipole=-1.1004057,-0.131827,-0.0000346\Qu
adrupole=-5.5992307,4.0794538,1.5197769,-0.1536302,-0.0001991,0.000086
3\PG=C01 [X(C2H3Se2)]\\@

1\1\GINC-ORC28\FOpt\RB3LYP\6-311+G(2df,p)\C2H3Se2(1-)\MEISTERP\11-Aug-
2017\0\\# opt freq b3lyp/6-311+g(2df,p) guess=read\\CH2CHSeSeH\\-1,1\C
,-3.0503446006,0.7690150895,-0.0000234528\H,-3.6102060283,-0.160885184
1,-0.0000707239\H,-3.628810993,1.6872490033,-0.0000039649\C,-1.7138199
077,0.801645718,0.0000096435\H,-1.1758233544,1.7449716055,0.0000567653

\Se,-0.5310780456,-0.6920236868,-0.0000125005\Se,1.6131869297,0.319902
4546,0.0000832333\\Version=EM64L-G09RevE.01\State=1-A\HF=-4881.203315\
RMSD=2.798e-09\RMSF=6.326e-05\Dipole=-1.219536,-0.1356766,-0.0000376\Q
uadrupole=-5.6188324,4.2416876,1.3771448,-0.1677301,-0.0001994,0.00009
65\PG=C01 [X(C2H3Se2)]\\@

1\1\GINC-ORC22\FOpt\RB3LYP\6-311++G(3df,3pd)\C2H3Se2(1-)\MEISTERP\11-A
ug-2017\0\\# opt freq b3lyp/6-311++g(3df,3pd) guess=read\\CH2CHSeSeH\\
-1,1\C,-3.0505592683,0.7690538386,-0.0000237188\H,-3.6098767347,-0.159
2161867,-0.0000708448\H,-3.627086406,1.6864790737,-0.0000041662\C,-1.7
139132339,0.8013563642,0.0000097998\H,-1.1773631157,1.7437424845,0.000
0573009\Se,-0.5310251058,-0.692166026,-0.0000118355\Se,1.6129278644,0.
3206254519,0.0000824646\\Version=EM64L-G09RevE.01\State=1-A\HF=-4881.2
127613\RMSD=3.454e-09\RMSF=7.081e-05\Dipole=-1.2029613,-0.1570604,-0.0
000371\Quadrupole=-5.729138,4.1613105,1.5678275,-0.1391064,-0.0002048,
0.0000882\PG=C01 [X(C2H3Se2)]\\@

$CH_2CHSeSe^-$ B3PW91

1\1\GINC-ORC28\FOpt\RB3PW91\6-31G(d)\C2H3Se2(1-)\MEISTERP\11-Aug-2017\
0\\# opt freq b3pw91/6-31g(d) guess=read\\CH2CHSeSeH\\-1,1\C,-3.039875
,0.758858,-0.000056\H,-3.601633,-0.174107,-0.000099\H,-3.623716,1.6782
48,-0.000188\C,-1.697381,0.789807,0.000095\H,-1.157941,1.737098,0.0002
41\Se,-0.512664,-0.688504,0.000007\Se,1.595218,0.31988,-0.000013\\Vers
ion=EM64L-G09RevE.01\State=1-A\HF=-4876.7425536\RMSD=3.355e-09\RMSF=5.
720e-05\Dipole=-1.0302817,-0.1127601,0.0000049\Quadrupole=-5.4087799,4
.0469442,1.3618357,-0.1571605,0.000186,0.0001756\PG=C01 [X(C2H3Se2)]\\
@

1\1\GINC-ORC22\FOpt\RB3PW91\6-311G(d)\C2H3Se2(1-)\MEISTERP\11-Aug-2017
\0\\# opt freq b3pw91/6-311g(d) guess=read\\CH2CHSeSeH\\-1,1\C,-3.0424
128774,0.758682873,-0.0000486346\H,-3.6020707704,-0.1744936363,-0.0001
292959\H,-3.6248526129,1.6777504107,-0.000026284\C,-1.7036611379,0.794
2164363,0.000015419\H,-1.1655834799,1.740517363,0.000088442\Se,-0.5169
183283,-0.6916227465,-0.0000100244\Se,1.6175072068,0.3162292998,0.0000
973779\\Version=EM64L-G09RevE.01\State=1-A\HF=-4881.1127448\RMSD=6.779
e-09\RMSF=9.245e-05\Dipole=-1.1316624,-0.1233673,-0.0000406\Quadrupole
=-5.2223313,4.1763563,1.045975,-0.1493616,-0.0001925,0.0001621\PG=C01
[X(C2H3Se2)]\\@

1\1\GINC-ORC22\FOpt\RB3PW91\6-311G(d,p)\C2H3Se2(1-)\MEISTERP\11-Aug-20
17\0\\# opt freq b3pw91/6-311g(d,p) guess=read\\CH2CHSeSeH\\-1,1\C,-3.
0430142462,0.7584152418,-0.0000507469\H,-3.5994068047,-0.1754399687,-0
.0001271793\H,-3.6236368726,1.677443835,-0.0000235406\C,-1.7041701144,
0.7954116559,0.0000115524\H,-1.1627281855,1.7390408556,0.0000879935\Se
,-0.5195734626,-0.6909882047,-0.0000106799\Se,1.6145376859,0.317396585
,0.0000996007\\Version=EM64L-G09RevE.01\State=1-A\HF=-4881.1183883\RMS
D=3.749e-09\RMSF=1.854e-05\Dipole=-1.1239327,-0.1294814,-0.0000381\Qua
drupole=-5.2963567,4.1244737,1.171883,-0.1138192,-0.0002297,0.0001573\
PG=C01 [X(C2H3Se2)]\\@

1\1\GINC-ORC17\FOpt\RB3PW91\6-311+G(d,p)\C2H3Se2(1-)\MEISTERP\11-Aug-2
017\0\\# opt freq b3pw91/6-311+g(d,p) guess=read\\CH2CHSeSeH\\-1,1\C,-
3.0423166083,0.7578301508,-0.000050729\H,-3.5999102094,-0.1752414815,-
0.0001276488\H,-3.6208814559,1.6779145453,-0.0000237086\C,-1.701610663
1,0.7941611359,0.0000119668\H,-1.1610427571,1.7384008295,0.000088097\S
e,-0.5210306397,-0.6929967036,-0.0000100785\Se,1.6088003335,0.32121152

35,0.0000991012\\Version=EM64L-G09RevE.01\State=1-A\HF=-4881.1247683\R
MSD=2.054e-09\RMSF=1.730e-05\Dipole=-1.2491242,-0.1461862,-0.000043\Qu
adrupole=-5.4227722,4.3385161,1.0842561,-0.1649324,-0.0002279,0.000172
6\PG=C01 [X(C2H3Se2)]\\@

1\1\GINC-ORC378\FOpt\RB3PW91\6-311G(2d,p)\C2H3Se2(1-)\MEISTERP\11-Aug-
2017\0\\# opt freq b3pw91/6-311g(2d,p) guess=read\\CH2CHSeSeH\\-1,1\C,
-3.0400454859,0.758168554,-0.0000504634\H,-3.5970894444,-0.1744991357,
-0.0001257039\H,-3.6192914961,1.6773298366,-0.0000227133\C,-1.70545002
05,0.7941857397,0.0000101903\H,-1.1658767334,1.7378964443,0.0000867159
\Se,-0.523651456,-0.6914836584,-0.000012228\Se,1.6134126362,0.31968221
95,0.0001012023\\Version=EM64L-G09RevE.01\State=1-A\HF=-4881.1149667\R
MSD=2.953e-09\RMSF=1.948e-05\Dipole=-1.0948255,-0.1038229,-0.0000376\Q
uadrupole=-5.1847796,4.0109559,1.1738237,-0.0528624,-0.0002296,0.00015
28\PG=C01 [X(C2H3Se2)]\\@

1\1\GINC-ORC375\FOpt\RB3PW91\6-311G(df,p)\C2H3Se2(1-)\MEISTERP\11-Aug-
2017\0\\# opt freq b3pw91/6-311g(df,p) guess=read\\CH2CHSeSeH\\-1,1\C,
-3.0395834281,0.7580970819,-0.0000492519\H,-3.5981905139,-0.1744172743
,-0.000122408\H,-3.6204712481,1.6766343272,-0.0000206524\C,-1.70275909
31,0.7923496562,0.000001912\H,-1.16388718,1.7377148136,0.000086023\Se,
-0.5174550771,-0.6852881814,-0.0000151832\Se,1.6043545403,0.3161895767
,0.0001065605\\Version=EM64L-G09RevE.01\State=1-A\HF=-4881.12679\RMSD=
3.211e-09\RMSF=1.853e-04\Dipole=-1.1057085,-0.1305804,-0.0000399\Quadr
upole=-5.5091033,4.100352,1.4087513,-0.1742164,-0.0002799,0.0001394\PG
=C01 [X(C2H3Se2)]\\@

1\1\GINC-ORC368\FOpt\RB3PW91\6-311+G(2df,p)\C2H3Se2(1-)\MEISTERP\11-Au
g-2017\0\\# opt freq b3pw91/6-311+g(2df,p) guess=read\\CH2CHSeSeH\\-1,
1\C,-3.0373463718,0.7572618176,-0.0000485437\H,-3.5952093007,-0.174885
8754,-0.0001286915\H,-3.6162738402,1.6762160044,-0.0000241829\C,-1.700
8650848,0.7925447263,0.0000143445\H,-1.16341708,1.7379237813,0.0000856
785\Se,-0.5212023481,-0.6868457247,-0.0000111188\Se,1.5963220256,0.319
0652705,0.0000995139\\Version=EM64L-G09RevE.01\State=1-A\HF=-4881.1290
572\RMSD=3.691e-09\RMSF=6.350e-05\Dipole=-1.1912991,-0.1304756,-0.0000
45\Quadrupole=-5.50974,4.2186185,1.2911215,-0.1562734,-0.0002178,0.000
1538\PG=C01 [X(C2H3Se2)]\\@

1\1\GINC-ORC366\FOpt\RB3PW91\6-311++G(3df,3pd)\C2H3Se2(1-)\MEISTERP\11
-Aug-2017\0\\# opt freq b3pw91/6-311++g(3df,3pd) guess=read\\CH2CHSeSe
H\\-1,1\C,-3.0375275005,0.7572249282,-0.000048693\H,-3.5950564362,-0.1
734682305,-0.0001289291\H,-3.6146410936,1.6756337567,-0.0000247102\C,-
1.7009241634,0.7922165789,0.0000150445\H,-1.1648734308,1.7369482314,0.
0000862266\Se,-0.5210713109,-0.6869551126,-0.0000104358\Se,1.596101935
3,0.3196798479,0.000098497\\Version=EM64L-G09RevE.01\State=1-A\HF=-488
1.1384468\RMSD=4.148e-09\RMSF=6.844e-05\Dipole=-1.177105,-0.1530208,-0
.0000452\Quadrupole=-5.6075332,4.1441999,1.4633332,-0.1221546,-0.00022
12,0.0001431\PG=C01 [X(C2H3Se2)]\\@

$CH_2CHSeSe^-$ wB97XD

1\1\GINC-ORC14\FOpt\RwB97XD\6-31G(d)\C2H3Se2(1-)\MEISTERP\10-Aug-2017\
0\\# opt freq wb97xd/6-31g(d)\\CH2CHSeSeH\\-1,1\C,-3.920100899,0.58315
71216,0.0008166309\H,-3.5104171343,-0.4253815846,0.0026680841\H,-5.004
7393367,0.6707757985,0.0001504605\C,-3.1391081495,1.6698591175,-0.0005
749742\H,-3.5725997625,2.669225749,-0.0023730365\Se,-1.2470281381,1.70
84607513,0.0000220886\Se,-0.7902139099,3.9967510867,-0.0043546634\\Ver

sion=EM64L-G09RevE.01\State=1-A\HF=-4876.8145544\RMSD=6.871e-09\RMSF=2
.321e-05\Dipole=-0.6074466,-0.9306809,0.0016393\Quadrupole=0.9678382,-
2.1864406,1.2186024,-4.2870078,0.0078272,0.005396\PG=C01 [X(C2H3Se2)]\
\@

1\1\GINC-ORC12\FOpt\RwB97XD\6-311G(d)\C2H3Se2(1-)\MEISTERP\10-Aug-2017
\0\\# opt freq wb97xd/6-311g(d)\\CH2CHSeSeH\\-1,1\C,-3.9221873789,0.58
15384788,0.0009672776\H,-3.5122729264,-0.4262399429,0.0021855586\H,-5.
0061088163,0.6693184258,0.0003627966\C,-3.1463117931,1.6677588516,-0.0
004218048\H,-3.5785964548,2.6660678675,-0.0024037266\Se,-1.2456850969,
1.7019951963,0.000219738\Se,-0.7730448634,4.0124091629,-0.0045552494\\
Version=EM64L-G09RevE.01\State=1-A\HF=-4881.1979291\RMSD=5.155e-09\RMS
F=1.117e-04\Dipole=-0.655184,-1.0140117,0.0017151\Quadrupole=1.0953006
,-1.9995342,0.9042336,-4.2643656,0.0092741,0.0061832\PG=C01 [X(C2H3Se2
)]\\@

1\1\GINC-ORC7\FOpt\RwB97XD\6-311G(d,p)\C2H3Se2(1-)\MEISTERP\10-Aug-201
7\0\\# opt freq wb97xd/6-311g(d,p)\\CH2CHSeSeH\\-1,1\C,-3.922466702,0.
5808790721,0.0009742625\H,-3.5104621855,-0.4249721928,0.0021342744\H,-
5.0051166169,0.6708815646,0.0002819897\C,-3.1470004141,1.6673721142,-0
.0003405302\H,-3.576213768,2.6664882293,-0.0023839974\Se,-1.2475013315
,1.7009093248,0.0003366992\Se,-0.7754463119,4.0112899278,-0.0046481083
\\Version=EM64L-G09RevE.01\State=1-A\HF=-4881.203314\RMSD=5.152e-09\RM
SF=1.149e-04\Dipole=-0.6442017,-1.0102129,0.001755\Quadrupole=1.003725
4,-2.0348561,1.0311306,-4.2884137,0.0096448,0.0068055\PG=C01 [X(C2H3Se
2)]\\@

1\1\GINC-ORC98\FOpt\RwB97XD\6-311+G(d,p)\C2H3Se2(1-)\MEISTERP\10-Aug-2
017\0\\# opt freq wb97xd/6-311+g(d,p)\\CH2CHSeSeH\\-1,1\C,-3.921548763
2,0.5809244966,0.0009777986\H,-3.5100516717,-0.4250612679,0.0021153924
\H,-5.0038531052,0.6726479264,0.0002582182\C,-3.1451956344,1.668964750
5,-0.0003029145\H,-3.5758605499,2.6675455855,-0.0023521081\Se,-1.24695
77125,1.6989181132,0.0003514353\Se,-0.7807398931,4.0089084358,-0.00469
32318\\Version=EM64L-G09RevE.01\State=1-A\HF=-4881.2099682\RMSD=2.995e
-09\RMSF=1.065e-04\Dipole=-0.7182295,-1.1314282,0.0019498\Quadrupole=1
.1706817,-2.0558268,0.8851451,-4.3677932,0.0102872,0.0071063\PG=C01 [X
(C2H3Se2)]\\@

1\1\GINC-ORC95\FOpt\RwB97XD\6-311G(2d,p)\C2H3Se2(1-)\MEISTERP\10-Aug-2
017\0\\# opt freq wb97xd/6-311g(2d,p)\\CH2CHSeSeH\\-1,1\C,-3.920664091
1,0.582648302,0.0007391598\H,-3.5092229799,-0.4224609761,0.0022957045\
H,-5.0024113845,0.6740402687,0.0001564203\C,-3.147563222,1.6659304357,
-0.000403567\H,-3.5788977312,2.6630650766,-0.0020337385\Se,-1.25041975
94,1.6985881847,0.000382489\Se,-0.7750281618,4.0110367483,-0.004781878
2\\Version=EM64L-G09RevE.01\State=1-A\HF=-4881.1996532\RMSD=9.488e-09\
RMSF=2.178e-05\Dipole=-0.6429543,-0.9710181,0.0019891\Quadrupole=0.905
967,-1.9230074,1.0170404,-4.1981933,0.0082165,0.0057027\PG=C01 [X(C2H3
Se2)]\\@

1\1\GINC-ORC30\FOpt\RwB97XD\6-311G(df,p)\C2H3Se2(1-)\MEISTERP\10-Aug-2
017\0\\# opt freq wb97xd/6-311g(df,p)\\CH2CHSeSeH\\-1,1\C,-3.920028839
,0.5826201214,0.0007970635\H,-3.5075431191,-0.4227591223,0.0024351782\
H,-5.0026124297,0.6710945214,0.0002711871\C,-3.1462536981,1.6679810723
,-0.0005068498\H,-3.5779746661,2.6660742345,-0.0021839717\Se,-1.251400
0051,1.7066726916,0.0000526638\Se,-0.7783945728,4.001164521,-0.0045106
811\\Version=EM64L-G09RevE.01\State=1-A\HF=-4881.2119678\RMSD=4.177e-0
9\RMSF=2.331e-05\Dipole=-0.6357215,-0.995508,0.0018524\Quadrupole=0.96

48322,-2.2343771,1.269545,-4.3638047,0.0079683,0.0061572\PG=C01 [X(C2H
3Se2)]\\@

1\1\GINC-ORC25\FOpt\RwB97XD\6-311+G(2df,p)\C2H3Se2(1-)\MEISTERP\10-Aug
-2017\0\\# opt freq wb97xd/6-311+g(2df,p)\\CH2CHSeSeH\\-1,1\C,-3.91805
95046,0.5837119042,0.0007909697\H,-3.5053369766,-0.4208206819,0.002370
2751\H,-4.999714121,0.6735699238,0.0002948471\C,-3.1453753806,1.669546
3809,-0.0004850037\H,-3.5802982835,2.6654955833,-0.0021243932\Se,-1.25
31333743,1.7042888884,0.0000666822\Se,-0.7822896894,3.9970560413,-0.00
45587873\\Version=EM64L-G09RevE.01\State=1-A\HF=-4881.2143297\RMSD=2.7
23e-09\RMSF=2.372e-05\Dipole=-0.6946635,-1.0767847,0.0019902\Quadrupol
e=1.048417,-2.1330082,1.0845912,-4.3609082,0.0081217,0.0059947\PG=C01
[X(C2H3Se2)]\\@

1\1\GINC-ORC174\FOpt\RwB97XD\6-311++G(3df,3pd)\C2H3Se2(1-)\MEISTERP\10
-Aug-2017\0\\# opt freq wb97xd/6-311++g(3df,3pd)\\CH2CHSeSeH\\-1,1\C,-
3.9183074758,0.5835441645,0.0007672924\H,-3.5062196886,-0.4193947236,0
.0023230843\H,-4.9980609551,0.6751512478,0.0002539182\C,-3.1451545197,
1.6691579413,-0.0004487489\H,-3.5806887244,2.6631842344,-0.0020659207\
Se,-1.2528993478,1.7040145585,0.000181587\Se,-0.7828766186,3.997190617
2,-0.0046566222\\Version=EM64L-G09RevE.01\State=1-A\HF=-4881.2242005\R
MSD=3.400e-09\RMSF=2.392e-05\Dipole=-0.6748855,-1.0821265,0.0020903\Qu
adrupole=0.9649072,-2.2061235,1.2412163,-4.373272,0.0082783,0.0067253\
PG=C01 [X(C2H3Se2)]\\@

$CH_2CHSeSe^-$ M062X

1\1\GINC-ORC17\FOpt\RM062X\6-31G(d)\C2H3Se2(1-)\MEISTERP\11-Aug-2017\0
\\# opt freq m062x/6-31g(d) guess=read\\CH2CHSeSeH\\-1,1\C,-3.01139690
24,0.7730611485,-0.00003481\H,-3.5684280498,-0.1608773773,-0.000057697
6\H,-3.5930232945,1.691307336,-0.000012284\C,-1.6743348988,0.799582629
9,-0.0000160585\H,-1.1257548096,1.7405323708,0.0000278903\Se,-0.513855
1434,-0.6955743742,0.0000038313\Se,1.5841460985,0.3210102665,0.0000771
284\\Version=EM64L-G09RevE.01\State=1-A\HF=-4876.8572792\RMSD=6.171e-0
9\RMSF=4.571e-05\Dipole=-1.119524,-0.0722636,-0.0000211\Quadrupole=-5.
1020968,3.8680194,1.2340773,-0.3660526,-0.0002344,0.000043\PG=C01 [X(C
2H3Se2)]\\@

1\1\GINC-ORC378\FOpt\RM062X\6-311G(d)\C2H3Se2(1-)\MEISTERP\11-Aug-2017
\0\\# opt freq m062x/6-311g(d) guess=read\\CH2CHSeSeH\\-1,1\C,-3.01431
8454,0.7731402688,-0.000033204\H,-3.5679084748,-0.1614444609,-0.000066
9956\H,-3.595626986,1.6903246805,-0.0000284888\C,-1.6800101082,0.80582
51082,0.0000053786\H,-1.1331561486,1.7452806193,0.0000407461\Se,-0.517
9907121,-0.7010435604,0.0000140534\Se,1.6063638837,0.3169593444,0.0000
565103\\Version=EM64L-G09RevE.01\State=1-A\HF=-4881.1463983\RMSD=2.144
e-09\RMSF=3.679e-05\Dipole=-1.2177629,-0.0787696,-0.0000172\Quadrupole
=-4.8525379,4.0180362,0.8345017,-0.3494744,-0.0001093,0.0000574\PG=C01
[X(C2H3Se2)]\\@

1\1\GINC-ORC375\FOpt\RM062X\6-311G(d,p)\C2H3Se2(1-)\MEISTERP\11-Aug-20
17\0\\# opt freq m062x/6-311g(d,p) guess=read\\CH2CHSeSeH\\-1,1\C,-3.0
150584572,0.7729448243,-0.0000331204\H,-3.5656099312,-0.1623903258,-0.
0000665453\H,-3.594092326,1.6904628562,-0.0000281915\C,-1.680792424,0.
8065291595,0.0000050444\H,-1.1316309971,1.7440672791,0.0000401791\Se,-
0.5199169634,-0.7002555721,0.0000133613\Se,1.6044540989,0.3176837788,0
.0000572724\\Version=EM64L-G09RevE.01\State=1-A\HF=-4881.1507375\RMSD=
8.616e-09\RMSF=3.644e-05\Dipole=-1.2141253,-0.0809829,-0.0000179\Quadr

upole=-4.9164841,3.9832952,0.9331889,-0.3271439,-0.0001152,0.0000549\P
G=C01 [X(C2H3Se2)]\\@

1\1\GINC-ORC368\FOpt\RM062X\6-311+G(d,p)\C2H3Se2(1-)\MEISTERP\11-Aug-2
017\0\\# opt freq m062x/6-311+g(d,p) guess=read\\CH2CHSeSeH\\-1,1\C,-3
.0146449011,0.7723395184,-0.0000333947\H,-3.566100863,-0.1624317879,-0
.0000679008\H,-3.5919205287,1.6907854634,-0.000029222\C,-1.6784348502,
0.8056926366,0.0000064357\H,-1.1305605787,1.7441130234,0.0000418083\Se
,-0.5206938134,-0.7016331287,0.0000154728\Se,1.599708535,0.3201762748,
0.0000548008\\Version=EM64L-G09RevE.01\State=1-A\HF=-4881.1568744\RMSD
=4.231e-09\RMSF=3.219e-05\Dipole=-1.323818,-0.0963424,-0.0000186\Quadr
upole=-4.9884334,4.1445162,0.8439173,-0.4028017,-0.0001045,0.0000608\P
G=C01 [X(C2H3Se2)]\\@

1\1\GINC-ORC366\FOpt\RM062X\6-311G(2d,p)\C2H3Se2(1-)\MEISTERP\11-Aug-2
017\0\\# opt freq m062x/6-311g(2d,p) guess=read\\CH2CHSeSeH\\-1,1\C,-3
.0125450025,0.7727332581,-0.0000332079\H,-3.5627961635,-0.1617482059,-
0.0000665617\H,-3.5904269191,1.6899178962,-0.0000282622\C,-1.682387338
4,0.8058861235,0.0000051102\H,-1.1354794972,1.7435723006,0.0000404997\
Se,-0.523800467,-0.6995868551,0.0000137872\Se,1.6047883876,0.318267482
5,0.0000566347\\Version=EM64L-G09RevE.01\State=1-A\HF=-4881.1487103\RM
SD=9.616e-09\RMSF=3.494e-05\Dipole=-1.1748052,-0.0590539,-0.0000174\Qu
adrupole=-4.7749916,3.8579868,0.9170048,-0.2563666,-0.0001104,0.000054
3\PG=C01 [X(C2H3Se2)]\\@

1\1\GINC-ORC362\FOpt\RM062X\6-311G(df,p)\C2H3Se2(1-)\MEISTERP\11-Aug-2
017\0\\# opt freq m062x/6-311g(df,p) guess=read\\CH2CHSeSeH\\-1,1\C,-3
.0122359997,0.7724853888,-0.0000301674\H,-3.5652959862,-0.1616632701,-
0.0000671349\H,-3.5916615331,1.6899049897,-0.0000338102\C,-1.680086510
6,0.8039471019,0.0000120997\H,-1.1333956405,1.7434804528,0.0000402455\
Se,-0.5170425476,-0.6945880816,0.0000111531\Se,1.5970712176,0.31547541
85,0.0000556142\\Version=EM64L-G09RevE.01\State=1-A\HF=-4881.159663\RM
SD=2.526e-09\RMSF=9.313e-05\Dipole=-1.1889915,-0.079016,-0.0000233\Qua
drupole=-5.1501866,3.9424068,1.2077798,-0.3910347,-0.0000878,0.000045\
PG=C01 [X(C2H3Se2)]\\@

1\1\GINC-ORC361\FOpt\RM062X\6-311+G(2df,p)\C2H3Se2(1-)\MEISTERP\11-Aug
-2017\0\\# opt freq m062x/6-311+g(2df,p) guess=read\\CH2CHSeSeH\\-1,1\
C,-3.0105263588,0.7717031618,-0.0000333745\H,-3.5609345,-0.1629164046,
-0.0000649631\H,-3.5889575405,1.6886272958,-0.0000268246\C,-1.67843132
69,0.8054975957,0.00000292\H,-1.1345293945,1.7455902728,0.0000390001\S
e,-0.5201703175,-0.6940533902,0.0000117935\Se,1.5909024383,0.314593468
7,0.0000594487\\Version=EM64L-G09RevE.01\State=1-A\HF=-4881.1631304\RM
SD=5.353e-09\RMSF=2.659e-05\Dipole=-1.2543529,-0.0817765,-0.0000199\Qu
adrupole=-5.0711838,4.0178782,1.0533055,-0.3778451,-0.0001372,0.000056
4\PG=C01 [X(C2H3Se2)]\\@

1\1\GINC-ORC389\FOpt\RM062X\6-311++G(3df,3pd)\C2H3Se2(1-)\MEISTERP\11-
Aug-2017\0\\# opt freq m062x/6-311++g(3df,3pd) guess=read\\CH2CHSeSeH\
\-1,1\C,-3.0108563906,0.771978751,-0.000029381\H,-3.5622960239,-0.1599
99096,-0.0000676048\H,-3.5862170226,1.6886067211,-0.0000305917\C,-1.67
86316978,0.8034738428,0.0000102124\H,-1.1365281595,1.7426061769,0.0000
368442\Se,-0.5193816898,-0.6950111878,0.0000104584\Se,1.5912639842,0.3
17386792,0.0000580626\\Version=EM64L-G09RevE.01\State=1-A\HF=-4881.172
1179\RMSD=3.931e-09\RMSF=9.545e-05\Dipole=-1.2344717,-0.106419,-0.0000
27\Quadrupole=-5.1716942,3.9420842,1.22961,-0.3580733,-0.00009,0.00004
41\PG=C01 [X(C2H3Se2)]\\@

$CH_2CHSeSe^-$ M08HX

```
1\1\GINC-ORC342\FOpt\RM08HX\6-31G(d)\C2H3Se2(1-)\MEISTERP\21-Aug-2017\
 0\\# opt freq=noraman m08hx/6-31g(d) guess=read\\CH2CHSeSeH\\-1,1\C,-3
 .006818,0.777836,0.000413\H,-3.572411,-0.156162,0.001791\H,-3.585332,1
 .703161,-0.000236\C,-1.668514,0.79766,-0.00042\H,-1.111064,1.739295,-0
 .001545\Se,-0.510283,-0.698518,-0.000099\Se,1.578542,0.323834,0.0001\\
 Version=EM64L-G16RevA.03\State=1-A\HF=-4876.9174143\RMSD=7.986e-09\RMS
 F=3.030e-05\Dipole=-1.1402267,-0.0943156,-0.0002953\Quadrupole=-5.1068
 616,3.9179315,1.1889302,-0.3419013,-0.0004403,-0.0026547\PG=C01 [X(C2H
 3Se2)]\\@
```

```
1\1\GINC-ORC255\FOpt\RM08HX\6-311G(d)\C2H3Se2(1-)\MEISTERP\21-Aug-2017
 \0\\# opt freq=noraman m08hx/6-311g(d) guess=read\\CH2CHSeSeH\\-1,1\C,
 -3.011988,0.786064,0.000104\H,-3.57612,-0.147652,0.000497\H,-3.587873,
 1.712021,-0.000087\C,-1.676689,0.808698,-0.000099\H,-1.117223,1.746608
 ,-0.000408\Se,-0.52143,-0.701493,-0.000027\Se,1.592409,0.322682,0.0000
 26\\Version=EM64L-G16RevA.03\State=1-A\HF=-4881.1683393\RMSD=4.818e-09
 \RMSF=2.171e-05\Dipole=-1.2460672,-0.1089409,-0.0000774\Quadrupole=-4.
 8864273,4.0574436,0.8289837,-0.3126366,-0.0001921,-0.0007625\PG=C01 [X
 (C2H3Se2)]\\@
```

```
1\1\GINC-ORC6\FOpt\RM08HX\6-311G(d,p)\C2H3Se2(1-)\MEISTERP\21-Aug-2017
 \0\\# opt freq=noraman m08hx/6-311g(d,p) guess=read\\CH2CHSeSeH\\-1,1\
 C,-3.010499,0.786756,0.000048\H,-3.571986,-0.147144,0.000293\H,-3.5831
 23,1.713208,-0.000088\C,-1.675245,0.809233,-0.000037\H,-1.112448,1.744
 284,-0.000209\Se,-0.521958,-0.701408,-0.000015\Se,1.592017,0.3224,0.00
 0013\\Version=EM64L-G16RevA.03\State=1-A\HF=-4881.1730378\RMSD=3.763e-
 09\RMSF=2.204e-05\Dipole=-1.2406439,-0.1126896,-0.0000431\Quadrupole=-
 4.9517808,4.0096319,0.9421489,-0.2841997,-0.0000991,-0.0004028\PG=C01
 [X(C2H3Se2)]\\@
```

```
1\1\GINC-ORC32\FOpt\RHF\6-311+G(d,p)\C2H3Se2(1-)\MEISTERP\21-Aug-2017\
 0\\# opt freq=noraman 6-311+g(d,p) guess=read\\CH2CHSeSeH\\-1,1\C,-3.0
 030632271,0.7885881295,0.0000840442\H,-3.5615554339,-0.1332513414,0.00
 03296505\H,-3.5704913304,1.7050318363,-0.0000170788\C,-1.6781710107,0.
 8137525603,-0.0000934833\H,-1.1437240273,1.7481639666,-0.0003289158\Se
 ,-0.5266937623,-0.7031817935,-0.0000049997\Se,1.6162947916,0.313261642
 2,0.0000427829\\Version=EM64L-G16RevA.03\State=1-A\HF=-4877.1309565\RM
 SD=6.162e-09\RMSF=8.527e-05\Dipole=-1.4631505,0.0073497,-0.0000648\Qua
 drupole=-4.6512214,4.0057056,0.6455158,-0.7066051,-0.0002,-0.0006318\P
 G=C01 [X(C2H3Se2)]\\@
```

```
1\1\GINC-ORC255\FOpt\RM08HX\6-311G(2d,p)\C2H3Se2(1-)\MEISTERP\21-Aug-2
 017\0\\# opt freq=noraman m08hx/6-311g(2d,p) guess=read\\CH2CHSeSeH\\-
 1,1\C,-3.005538,0.788392,0.000363\H,-3.567313,-0.144046,0.001539\H,-3.
 576824,1.714342,-0.00016\C,-1.674614,0.809553,-0.000367\H,-1.113659,1.
 744419,-0.00139\Se,-0.525509,-0.70043,-0.000086\Se,1.594295,0.320948,0
 .000087\\Version=EM64L-G16RevA.03\State=1-A\HF=-4881.1718128\RMSD=3.52
 5e-09\RMSF=2.361e-05\Dipole=-1.191781,-0.0894023,-0.0002162\Quadrupole
 =-4.7917376,3.8631014,0.9286362,-0.1899523,-0.0005518,-0.0022365\PG=C0
 1 [X(C2H3Se2)]\\@
```

```
1\1\GINC-ORC6\FOpt\RM08HX\6-311G(df,p)\C2H3Se2(1-)\MEISTERP\21-Aug-201
 7\0\\# opt freq=noraman m08hx/6-311g(df,p) guess=read\\CH2CHSeSeH\\-1,
 1\C,-3.007308,0.77938,0.000336\H,-3.568276,-0.155034,0.001446\H,-3.583
```

286,1.703959,-0.000158\C,-1.674354,0.803626,-0.000338\H,-1.115819,1.74
1711,-0.001292\Se,-0.51717,-0.697962,-0.000081\Se,1.586504,0.321824,0.
000081\\Version=EM64L-G16RevA.03\State=1-A\HF=-4881.1827555\RMSD=3.675
e-09\RMSF=2.620e-05\Dipole=-1.2079622,-0.1103181,-0.0002317\Quadrupole
=-5.1826308,3.9647139,1.2179169,-0.3616161,-0.0005148,-0.0021213\PG=C0
1 [X(C2H3Se2)]\\@

1\1\GINC-ORC336\FOpt\RM08HX\6-311+G(2df,p)\C2H3Se2(1-)\MEISTERP\21-Aug
-2017\0\\# opt freq=noraman m08hx/6-311+g(2df,p) guess=read\\CH2CHSeSe
H\\-1,1\C,-3.002293,0.777515,0.000571\H,-3.561128,-0.156996,0.00235\H,
-3.577662,1.701064,-0.000179\C,-1.669373,0.803842,-0.000594\H,-1.11523
2,1.74343,-0.002156\Se,-0.516362,-0.696941,-0.000133\Se,1.583539,0.321
187,0.000136\\Version=EM64L-G16RevA.03\State=1-A\HF=-4881.1873529\RMSD
=3.174e-09\RMSF=2.498e-05\Dipole=-1.250404,-0.1213658,-0.0003299\Quadr
upole=-5.1227164,4.0383373,1.0843791,-0.3120102,-0.001197,-0.0037719\P
G=C01 [X(C2H3Se2)]\\@

1\1\GINC-ORC336\FOpt\RM08HX\6-311++G(3df,3pd)\C2H3Se2(1-)\MEISTERP\21-
Aug-2017\0\\# opt freq=noraman m08hx/6-311++g(3df,3pd) guess=read\\CH2
CHSeSeH\\-1,1\C,-3.001545,0.780385,0.00036\H,-3.560634,-0.150895,0.001
524\H,-3.572123,1.703834,-0.000153\C,-1.668348,0.804136,-0.000363\H,-1
.114332,1.741022,-0.001389\Se,-0.517111,-0.698121,-0.000086\Se,1.58377
1,0.321618,0.000087\\Version=EM64L-G16RevA.03\State=1-A\HF=-4881.19627
01\RMSD=9.957e-09\RMSF=2.521e-05\Dipole=-1.240842,-0.1430621,-0.000203
4\Quadrupole=-5.154176,3.9634941,1.190682,-0.3051102,-0.0007527,-0.002
346\PG=C01 [X(C2H3Se2)]\\@

$CH_2CHSeSe^-$ QCISD

1\1\GINC-ORC24\FOpt\RQCISD-FC\6-311+G(2df,p)\C2H3Se2(1-)\MEISTERP\23-A
ug-2017\0\\# opt freq=noraman qcisd/6-311+g(2df,p) guess=read\\CH2CHSe
Se benchmark\\-1,1\C,3.021994,0.783902,0.000082\H,3.583313,-0.146809,0
.000158\H,3.595834,1.706526,0.000057\C,1.683219,0.809842,0.000019\H,1.
141761,1.753716,-0.000055\Se,0.525799,-0.700044,0.000046\Se,-1.600863,
0.321341,-0.000069\\Version=EM64L-G09RevE.01\State=1-A\HF=-4877.137680
6\MP2=-4877.7628779\MP3=-4877.8002596\MP4D=-4877.8168874\MP4DQ=-4877.7
969085\MP4SDQ=-4877.8027201\QCISD=-4877.802826\RMSD=3.943e-09\RMSF=6.0
79e-05\Dipole=1.3322645,-0.0696333,0.0000474\PG=C01 [X(C2H3Se2)]\\@

$H^•$ B3LYP

1\1\GINC-GRA349\FOpt\UB3LYP\6-311+G(d,p)\H1(2)\MEISTERP\20-Nov-2018\0\
\# opt freq=noraman b3lyp/6-311+g(d,p)\\H atom\\0,2\H,-1.8346233,1.403
11149,-0.01836652\\Version=ES64L-G16RevB.01\State=2-A1G\HF=-0.5021559\
S2=0.75\S2-1=0.\S2A=0.75\RMSD=0.000e+00\RMSF=0.000e+00\Dipole=0.,0.,0.
\Quadrupole=0.,0.,0.,0.,0.,0.\PG=OH [O(H1)]\\@

1\1\GINC-GRA351\FOpt\UB3LYP\6-311++G(3df,3pd)\H1(2)\MEISTERP\20-Nov-20
18\0\\# opt freq=noraman b3lyp/6-311++g(3df,3pd)\\H atom\\0,2\H,-1.834
6233,1.40311149,-0.01836652\\Version=ES64L-G16RevB.01\State=2-A1G\HF=-
0.502257\S2=0.75\S2-1=0.\S2A=0.75\RMSD=0.000e+00\RMSF=0.000e+00\Dipole
=0.,0.,0.\Quadrupole=0.,0.,0.,0.,0.,0.\PG=OH [O(H1)]\\@

1\1\GINC-ORC19\FOpt\UB3LYP\6-31G(d)\H1(2)\SNIKOO\19-Aug-2017\0\\# opt
freq ub3lyp/6-31g(d)\\H RADICAL\\0,2\H,-0.29572617,0.14351703,0.385678
64\\Version=EM64L-G16RevA.03\State=2-A1G\HF=-0.5002728\S2=0.75\S2-1=0.
\S2A=0.75\RMSD=0.000e+00\RMSF=0.000e+00\Dipole=0.,0.,0.\Quadrupole=0.,

0.,0.,0.,0.,0.\PG=OH [O(H1)]\\@

1\1\GINC-ORC278\Freq\UB3LYP\6-311G(2d,p)\H1(2)\SNIKOO\20-Aug-2017\0\\#
N Geom=AllCheck Guess=TCheck SCRF=Check GenChk UB3LYP/6-311G(2d,p) Fre
q\\H RADICAL\\0,2\H,0.,0.,0.\\Version=EM64L-G16RevA.03\State=2-A1G\HF=
-0.5021559\S2=0.75\S2-1=0.\S2A=0.75\RMSD=0.000e+00\RMSF=0.000e+00\Ther
mal=0.0014163\Dipole=0.,0.,0.\DipoleDeriv=0.,0.,0.,0.,0.,0.,0.,0.,0.\P
olar=0.5906855,0.,0.5906855,0.,0.,0.5906855\Quadrupole=0.,0.,0.,0.,0.,
0.\PG=OH [O(H1)]\NImag=0\\0.,0.,0.,0.,0.,0.\\0.,0.,0.\\\@

1\1\GINC-ORC90\FOpt\UB3LYP\6-311++G(3df,3pd)\H1(2)\SNIKOO\20-Aug-2017\
0\\# opt freq ub3lyp/6-311++g(3df,3pd)\\H RADICAL\\0,2\H,0.,0.,0.\\Ver
sion=EM64L-G09RevD.01\State=2-A1G\HF=-0.502257\S2=0.75\S2-1=0.\S2A=0.7
5\RMSD=1.352e-09\RMSF=0.000e+00\Dipole=0.,0.,0.\Quadrupole=0.,0.,0.,0.
,0.,0.\PG=OH [O(H1)]\\@

H• B3PW91

1\1\GINC-GRA349\FOpt\UB3PW91\6-311+G(d,p)\H1(2)\MEISTERP\20-Nov-2018\0
\\# opt freq=noraman b3pw91/6-311+g(d,p)\\H atom\\0,2\H,-1.8346233,1.4
0311149,-0.01836652\\Version=ES64L-G16RevB.01\State=2-A1G\HF=-0.503978
7\S2=0.75\S2-1=0.\S2A=0.75\RMSD=0.000e+00\RMSF=0.000e+00\Dipole=0.,0.,
0.\Quadrupole=0.,0.,0.,0.,0.,0.\PG=OH [O(H1)]\\@

1\1\GINC-GRA349\FOpt\UB3PW91\6-311++G(3df,3pd)\H1(2)\MEISTERP\20-Nov-2
018\0\\# opt freq=noraman b3pw91/6-311++g(3df,3pd)\\H atom\\0,2\H,-1.8
346233,1.40311149,-0.01836652\\Version=ES64L-G16RevB.01\State=2-A1G\HF
=-0.5040652\S2=0.75\S2-1=0.\S2A=0.75\RMSD=0.000e+00\RMSF=0.000e+00\Dip
ole=0.,0.,0.\Quadrupole=0.,0.,0.,0.,0.,0.\PG=OH [O(H1)]\\@

1\1\GINC-ORC288\FOpt\UB3PW91\6-31G(d)\H1(2)\SNIKOO\20-Aug-2017\0\\# op
t freq ub3pw91/6-31g(d)\\H RADICAL\\0,2\H,0.,0.,0.\\Version=EM64L-G09R
evD.01\State=2-A1G\HF=-0.5021769\S2=0.75\S2-1=0.\S2A=0.75\RMSD=3.958e-
11\RMSF=0.000e+00\Dipole=0.,0.,0.\Quadrupole=0.,0.,0.,0.,0.,0.\PG=OH [
O(H1)]\\@

1\1\GINC-ORC285\FOpt\UB3PW91\6-311G(2d,p)\H1(2)\SNIKOO\20-Aug-2017\0\\
opt freq ub3pw91/6-311g(2d,p)\\H RADICAL\\0,2\H,0.,0.,0.\\Version=EM
64L-G09RevD.01\State=2-A1G\HF=-0.5039787\S2=0.75\S2-1=0.\S2A=0.75\RMSD
=2.862e-12\RMSF=0.000e+00\Dipole=0.,0.,0.\Quadrupole=0.,0.,0.,0.,0.,0.
\PG=OH [O(H1)]\\@

1\1\GINC-ORC213\FOpt\UB3PW91\6-311++G(3df,3pd)\H1(2)\SNIKOO\20-Aug-201
7\0\\# opt freq ub3pw91/6-311++g(3df,3pd)\\H RADICAL\\0,2\H,0.,0.,0.\\
Version=EM64L-G09RevD.01\State=2-A1G\HF=-0.5040652\S2=0.75\S2-1=0.\S2A
=0.75\RMSD=3.457e-09\RMSF=0.000e+00\Dipole=0.,0.,0.\Quadrupole=0.,0.,0
.,0.,0.,0.\PG=OH [O(H1)]\\@

H• wB97XD

1\1\GINC-GRA701\FOpt\UwB97XD\6-311+G(d,p)\H1(2)\MEISTERP\20-Nov-2018\0
\\# opt freq=noraman wb97xd/6-311+g(d,p)\\H atom\\0,2\H,-1.8346233,1.4
0311149,-0.01836652\\Version=ES64L-G16RevB.01\State=2-A1G\HF=-0.502668
3\S2=0.75\S2-1=0.\S2A=0.75\RMSD=0.000e+00\RMSF=0.000e+00\Dipole=0.,0.,
0.\Quadrupole=0.,0.,0.,0.,0.,0.\PG=OH [O(H1)]\\@

1\1\GINC-GRA701\FOpt\UwB97XD\6-311++G(3df,3pd)\H1(2)\MEISTERP\20-Nov-2
018\0\\# opt freq=noraman wb97xd/6-311++g(3df,3pd)\\H atom\\0,2\H,-1.8
346233,1.40311149,-0.01836652\\Version=ES64L-G16RevB.01\State=2-A1G\HF
=-0.5028028\S2=0.75\S2-1=0.\S2A=0.75\RMSD=0.000e+00\RMSF=0.000e+00\Dip
ole=0.,0.,0.\Quadrupole=0.,0.,0.,0.,0.,0.\PG=OH [O(H1)]\\@

1\1\GINC-ORC300\FOpt\UwB97XD\6-31G(d)\H1(2)\SNIKOO\20-Aug-2017\0\\# op
t freq uwb97xd/6-31g(d)\\H RADICAL\\0,2\H,0.,0.,0.\\Version=EM64L-G09R
evD.01\State=2-A1G\HF=-0.5006076\S2=0.75\S2-1=0.\S2A=0.75\RMSD=1.037e-
14\RMSF=0.000e+00\Dipole=0.,0.,0.\Quadrupole=0.,0.,0.,0.,0.,0.\PG=OH [
O(H1)]\\@

1\1\GINC-ORC278\FOpt\UwB97XD\6-311G(2d,p)\H1(2)\SNIKOO\20-Aug-2017\0\\
opt freq uwb97xd/6-311g(2d,p)\\H RADICAL\\0,2\H,0.,0.,0.\\Version=EM
64L-G09RevD.01\State=2-A1G\HF=-0.5026683\S2=0.75\S2-1=0.\S2A=0.75\RMSD
=1.199e-09\RMSF=0.000e+00\Dipole=0.,0.,0.\Quadrupole=0.,0.,0.,0.,0.,0.
\PG=OH [O(H1)]\\@

1\1\GINC-ORC213\FOpt\UwB97XD\6-311++G(3df,3pd)\H1(2)\SNIKOO\20-Aug-201
7\0\\# opt freq uwb97xd/6-311++g(3df,3pd)\\H RADICAL\\0,2\H,0.,0.,0.\\
Version=EM64L-G09RevD.01\State=2-A1G\HF=-0.5028028\S2=0.75\S2-1=0.\S2A
=0.75\RMSD=3.591e-09\RMSF=0.000e+00\Dipole=0.,0.,0.\Quadrupole=0.,0.,0
.,0.,0.,0.\PG=OH [O(H1)]\\@

H• M062X

1\1\GINC-GRA737\FOpt\UM062X\6-311+G(d,p)\H1(2)\MEISTERP\20-Nov-2018\0\
\# opt freq=noraman m062x/6-311+g(d,p)\\H atom\\0,2\H,-1.8346233,1.403
11149,-0.01836652\\Version=ES64L-G16RevB.01\State=2-A1G\HF=-0.4981341\
S2=0.75\S2-1=0.\S2A=0.75\RMSD=0.000e+00\RMSF=0.000e+00\Dipole=0.,0.,0.
\Quadrupole=0.,0.,0.,0.,0.,0.\PG=OH [O(H1)]\\@

1\1\GINC-GRA320\FOpt\UM062X\6-311++G(3df,3pd)\H1(2)\MEISTERP\20-Nov-20
18\0\\# opt freq=noraman m062x/6-311++g(3df,3pd)\\H atom\\0,2\H,-1.834
6233,1.40311149,-0.01836652\\Version=ES64L-G16RevB.01\State=2-A1G\HF=-
0.4981948\S2=0.75\S2-1=0.\S2A=0.75\RMSD=0.000e+00\RMSF=0.000e+00\Dipol
e=0.,0.,0.\Quadrupole=0.,0.,0.,0.,0.,0.\PG=OH [O(H1)]\\@

1\1\GINC-ORC330\FOpt\UM062X\6-31G(d)\H1(2)\SNIKOO\20-Aug-2017\0\\# opt
 freq m062x/6-31g(d)\\H RADICAL\\0,2\H,0.,0.,0.\\Version=EM64L-G09RevD
.01\State=2-A1G\HF=-0.4966657\S2=0.75\S2-1=0.\S2A=0.75\RMSD=1.267e-14\
RMSF=0.000e+00\Dipole=0.,0.,0.\Quadrupole=0.,0.,0.,0.,0.,0.\PG=OH [O(H
1)]\\@

1\1\GINC-ORC330\FOpt\UM062X\6-311G(2d,p)\H1(2)\SNIKOO\20-Aug-2017\0\\#
 opt freq m062x/6-311g(2d,p)\\H RADICAL\\0,2\H,0.,0.,0.\\Version=EM64L
-G09RevD.01\State=2-A1G\HF=-0.4981341\S2=0.75\S2-1=0.\S2A=0.75\RMSD=1.
228e-15\RMSF=0.000e+00\Dipole=0.,0.,0.\Quadrupole=0.,0.,0.,0.,0.,0.\PG
=OH [O(H1)]\\@

1\1\GINC-ORC213\FOpt\UM062X\6-311++G(3df,3pd)\H1(2)\SNIKOO\20-Aug-2017
\0\\# opt freq m062x/6-311++g(3df,3pd)\\H RADICAL\\0,2\H,0.,0.,0.\\Ver
sion=EM64L-G09RevD.01\State=2-A1G\HF=-0.4981948\S2=0.75\S2-1=0.\S2A=0.
75\RMSD=5.125e-09\RMSF=0.000e+00\Dipole=0.,0.,0.\Quadrupole=0.,0.,0.,0
.,0.,0.\PG=OH [O(H1)]\\@

H• M08HX

```
1\1\GINC-GRA320\FOpt\UM08HX\6-311+G(d,p)\H1(2)\MEISTERP\20-Nov-2018\0\
 \# opt freq=noraman m08hx/6-311+g(d,p)\\H atom\\0,2\H,-1.8346233,1.403
 11149,-0.01836652\\Version=ES64L-G16RevB.01\State=2-A1G\HF=-0.5018648\
 S2=0.75\S2-1=0.\S2A=0.75\RMSD=0.000e+00\RMSF=0.000e+00\Dipole=0.,0.,0.
 \Quadrupole=0.,0.,0.,0.,0.,0.\PG=OH [O(H1)]\\@
```

```
1\1\GINC-GRA320\FOpt\UM08HX\6-311++G(3df,3pd)\H1(2)\MEISTERP\20-Nov-20
 18\0\\# opt freq=noraman m08hx/6-311++g(3df,3pd)\\H atom\\0,2\H,-1.834
 6233,1.40311149,-0.01836652\\Version=ES64L-G16RevB.01\State=2-A1G\HF=-
 0.5020349\S2=0.75\S2-1=0.\S2A=0.75\RMSD=0.000e+00\RMSF=0.000e+00\Dipol
 e=0.,0.,0.\Quadrupole=0.,0.,0.,0.,0.,0.\PG=OH [O(H1)]\\@
```

```
1\1\GINC-ORC213\FOpt\UM08HX\6-31G(d)\H1(2)\SNIKOO\20-Aug-2017\0\\# opt
  freq 6-31g(d) m08hx\\H RADICAL\\0,2\H,0.,0.,0.\\Version=EM64L-G16RevA
 .03\State=2-A1G\HF=-0.4992935\S2=0.75\S2-1=0.\S2A=0.75\RMSD=7.222e-16\
 RMSF=0.000e+00\Dipole=0.,0.,0.\Quadrupole=0.,0.,0.,0.,0.,0.\PG=OH [O(H
 1)]\\@
```

```
1\1\GINC-ORC330\FOpt\UM08HX\6-311G(2d,p)\H1(2)\SNIKOO\20-Aug-2017\0\\#
  opt freq 6-311g(2d,p) m08hx\\H RADICAL\\0,2\H,0.,0.,0.\\Version=EM64L
 -G16RevA.03\State=2-A1G\HF=-0.5018648\S2=0.75\S2-1=0.\S2A=0.75\RMSD=4.
 028e-10\RMSF=0.000e+00\Dipole=0.,0.,0.\Quadrupole=0.,0.,0.,0.,0.,0.\PG
 =OH [O(H1)]\\@
```

```
1\1\GINC-ORC330\FOpt\UM08HX\6-311++G(3df,3pd)\H1(2)\SNIKOO\20-Aug-2017
 \0\\# opt freq m08hx/6-311++g(3df,3pd)\\H RADICAL\\0,2\H,0.,0.,0.\\Ver
 sion=EM64L-G16RevA.03\State=2-A1G\HF=-0.5020349\S2=0.75\S2-1=0.\S2A=0.
 75\RMSD=2.835e-09\RMSF=0.000e+00\Dipole=0.,0.,0.\Quadrupole=0.,0.,0.,0
 .,0.,0.\PG=OH [O(H1)]\\@
```

H• QCISD

```
1\1\GINC-ORC330\FOpt\UQCISD-FC\6-311+G(2df,p)\H1(2)\SNIKOO\20-Aug-2017
 \0\\# opt freq 6-311+g(2df,p) qcisd\\H RADICAL\\0,2\H,0.,0.,0.\\Versio
 n=EM64L-G09RevD.01\State=2-A1G\HF=-0.4998098\MP2=-0.4998098\MP3=-0.499
 8098\MP4D=-0.4998098\MP4DQ=-0.4998098\MP4SDQ=-0.4998098\QCISD=-0.49980
 98\S2=0.75\S2-1=0.\S2A=0.75\RMSD=1.663e-09\RMSF=0.000e+00\Dipole=0.,0.
 ,0.\PG=OH [O(H1)]\\@
```

H-Se• B3LYP

```
1\1\GINC-ORC112\FOpt\UB3LYP\6-311+G(d,p)\H1Se1(2)\MEISTERP\18-Aug-2017
 \0\\# opt freq=noraman b3lyp/6-311+g(d,p)\\SeH radical\\0,2\Se,-0.3211
 199346,1.53392328,0.\H,-1.7978475854,1.53392328,0.\\Version=EM64L-G09R
 evE.01\HF=-2402.117583\S2=0.752253\S2-1=0.\S2A=0.750002\RMSD=1.578e-09
 \RMSF=1.082e-04\Dipole=-0.2570475,0.,0.\Quadrupole=1.3354995,-2.020743
 ,0.6852435,0.,0.,0.\PG=C*V [C*(H1Se1)]\\@
```

```
1\1\GINC-ORC19\FOpt\UB3LYP\6-311G(df,p)\H1Se1(2)\MEISTERP\18-Aug-2017\
 0\\# opt freq=noraman b3lyp/6-311g(df,p)\\SeH radical\\0,2\Se,-0.32094
 78576,1.53392328,0.\H,-1.7980196624,1.53392328,0.\\Version=EM64L-G09Re
 vE.01\HF=-2402.1187682\S2=0.752633\S2-1=0.\S2A=0.750004\RMSD=1.337e-09
 \RMSF=1.207e-04\Dipole=-0.2515439,0.,0.\Quadrupole=1.2524579,0.608052,
 -1.8605099,0.,0.,0.\PG=C*V [C*(H1Se1)]\\@
```

```
1\1\GINC-ORC17\FOpt\UB3LYP\6-311+G(2df,p)\H1Se1(2)\MEISTERP\18-Aug-201
 7\0\\# opt freq=noraman b3lyp/6-311+g(2df,p)\\SeH radical\\0,2\Se,-0.3
 219735733,1.53392328,0.\H,-1.7969939467,1.53392328,0.\\Version=EM64L-G
 09RevE.01\HF=-2402.11691\S2=0.752714\S2-1=0.\S2A=0.750004\RMSD=1.129e-
 09\RMSF=9.161e-05\Dipole=-0.2086828,0.,0.\Quadrupole=1.3294349,-1.9495
 476,0.6201127,0.,0.,0.\PG=C*V [C*(H1Se1)]\\@
```

```
1\1\GINC-ORC19\FOpt\UB3LYP\6-311++G(3df,3pd)\H1Se1(2)\MEISTERP\18-Aug-
 2017\0\\# opt freq=noraman b3lyp/6-311++g(3df,3pd)\\SeH radical\\0,2\S
 e,-0.3224746543,1.53392328,0.\H,-1.7964928657,1.53392328,0.\\Version=E
 M64L-G09RevE.01\HF=-2402.1207423\S2=0.752781\S2-1=0.\S2A=0.750004\RMSD
 =6.528e-09\RMSF=6.797e-05\Dipole=-0.1985183,0.,0.\Quadrupole=1.2467592
 ,0.6494316,-1.8961908,0.,0.,0.\PG=C*V [C*(H1Se1)]\\@
```

H-Se˙ B3PW91

```
1\1\GINC-ORC4\FOpt\UB3PW91\6-311+G(d,p)\H1Se1(2)\MEISTERP\18-Aug-2017\
 0\\# opt freq=noraman b3pw91/6-311+g(d,p)\\SeH radical\\0,2\Se,-0.3225
 794724,1.53392328,0.\H,-1.7963880476,1.53392328,0.\\Version=EM64L-G09R
 evE.01\HF=-2402.0958434\S2=0.752778\S2-1=0.\S2A=0.750003\RMSD=7.802e-0
 9\RMSF=7.555e-05\Dipole=-0.2715574,0.,0.\Quadrupole=1.3511156,-2.00507
 17,0.6539561,0.,0.,0.\PG=C*V [C*(H1Se1)]\\@
```

```
1\1\GINC-ORC316\FOpt\UB3PW91\6-311G(df,p)\H1Se1(2)\MEISTERP\18-Aug-201
 7\0\\# opt freq=noraman b3pw91/6-311g(df,p)\\SeH radical\\0,2\Se,-0.32
 24632672,1.53392328,0.\H,-1.7965042528,1.53392328,0.\\Version=EM64L-G0
 9RevE.01\HF=-2402.0973011\S2=0.753334\S2-1=0.\S2A=0.750006\RMSD=8.470e
 -10\RMSF=8.513e-05\Dipole=-0.2680132,0.,0.\Quadrupole=1.2799192,0.5823
 544,-1.8622735,0.,0.,0.\PG=C*V [C*(H1Se1)]\\@
```

```
1\1\GINC-ORC294\FOpt\UB3PW91\6-311+G(2df,p)\H1Se1(2)\MEISTERP\18-Aug-2
 017\0\\# opt freq=noraman b3pw91/6-311+g(2df,p)\\SeH radical\\0,2\Se,-
 0.3234265345,1.53392328,0.\H,-1.7955409855,1.53392328,0.\\Version=EM64
 L-G09RevE.01\HF=-2402.0952488\S2=0.753453\S2-1=0.\S2A=0.750006\RMSD=5.
 092e-09\RMSF=4.649e-05\Dipole=-0.2195591,0.,0.\Quadrupole=1.3448797,-1
 .9360076,0.5911278,0.,0.,0.\PG=C*V [C*(H1Se1)]\\@
```

```
1\1\GINC-ORC176\FOpt\UB3PW91\6-311++G(3df,3pd)\H1Se1(2)\MEISTERP\18-Au
 g-2017\0\\# opt freq=noraman b3pw91/6-311++g(3df,3pd)\\SeH radical\\0,
 2\Se,-0.3237023871,1.53392328,0.\H,-1.7952651329,1.53392328,0.\\Versio
 n=EM64L-G09RevE.01\HF=-2402.0991365\S2=0.753545\S2-1=0.\S2A=0.750007\R
 MSD=4.713e-09\RMSF=3.200e-05\Dipole=-0.2105259,0.,0.\Quadrupole=1.2629
 941,-1.8823148,0.6193207,0.,0.,0.\PG=C*V [C*(H1Se1)]\\@
```

H-Se˙ wB97XD

```
1\1\GINC-ORC19\FOpt\UwB97XD\6-311+G(d,p)\H1Se1(2)\MEISTERP\18-Aug-2017
 \0\\# opt freq=noraman wb97xd/6-311+g(d,p)\\SeH radical\\0,2\Se,-0.324
 9121851,1.53392328,0.\H,-1.7940553349,1.53392328,0.\\Version=EM64L-G09
 RevE.01\HF=-2402.1384837\S2=0.752448\S2-1=0.\S2A=0.750003\RMSD=5.231e-
 09\RMSF=2.128e-05\Dipole=-0.271799,0.,0.\Quadrupole=1.3494599,-2.01549
 51,0.6660352,0.,0.,0.\PG=C*V [C*(H1Se1)]\\@
```

```
1\1\GINC-ORC19\FOpt\UwB97XD\6-311G(df,p)\H1Se1(2)\MEISTERP\18-Aug-2017
 \0\\# opt freq=noraman wb97xd/6-311g(df,p)\\SeH radical\\0,2\Se,-0.324
 48376,1.53392328,0.\H,-1.79448376,1.53392328,0.\\Version=EM64L-G09RevE
 .01\HF=-2402.1398503\S2=0.752803\S2-1=0.\S2A=0.750004\RMSD=1.943e-09\R
```

MSF=8.782e-05\Dipole=-0.2681488,0.,0.\Quadrupole=1.2777593,-1.873308,0
.5955486,0.,0.,0.\PG=C*V [C*(H1Se1)]\\@

1\1\GINC-ORC17\FOpt\UwB97XD\6-311+G(2df,p)\H1Se1(2)\MEISTERP\18-Aug-20
17\0\\# opt freq=noraman wb97xd/6-311+g(2df,p)\\SeH radical\\0,2\Se,-0
.3255708611,1.53392328,0.\H,-1.7933966589,1.53392328,0.\\Version=EM64L
-G09RevE.01\HF=-2402.1378523\S2=0.752869\S2-1=0.\S2A=0.750005\RMSD=6.5
79e-09\RMSF=5.764e-05\Dipole=-0.221024,0.,0.\Quadrupole=1.3538721,-1.9
496775,0.5958054,0.,0.,0.\PG=C*V [C*(H1Se1)]\\@

1\1\GINC-ORC4\FOpt\UwB97XD\6-311++G(3df,3pd)\H1Se1(2)\MEISTERP\18-Aug-
2017\0\\# opt freq=noraman wb97xd/6-311++g(3df,3pd)\\SeH radical\\0,2\
Se,-0.3259310858,1.53392328,0.\H,-1.7930364342,1.53392328,0.\\Version=
EM64L-G09RevE.01\HF=-2402.1417312\S2=0.752921\S2-1=0.\S2A=0.750005\RMS
D=6.960e-09\RMSF=7.270e-05\Dipole=-0.2142293,0.,0.\Quadrupole=1.279611
,0.6233857,-1.9029967,0.,0.,0.\PG=C*V [C*(H1Se1)]\\@

H-Se• M062X

1\1\GINC-ORC176\FOpt\UM062X\6-311+G(d,p)\H1Se1(2)\MEISTERP\18-Aug-2017
\0\\# opt freq=noraman m062x/6-311+g(d,p)\\SeH radical\\0,2\Se,-0.3255
690823,1.53392328,0.\H,-1.7933984377,1.53392328,0.\\Version=EM64L-G09R
evE.01\HF=-2402.1133057\S2=0.754206\S2-1=0.\S2A=0.75001\RMSD=5.344e-09
\RMSF=8.819e-05\Dipole=-0.3014202,0.,0.\Quadrupole=1.3340588,0.7099843
,-2.0440431,0.,0.,0.\PG=C*V [C*(H1Se1)]\\@

1\1\GINC-ORC112\FOpt\UM062X\6-311G(df,p)\H1Se1(2)\MEISTERP\18-Aug-2017
\0\\# opt freq=noraman m062x/6-311g(df,p)\\SeH radical\\0,2\Se,-0.3250
462069,1.53392328,0.\H,-1.7939213131,1.53392328,0.\\Version=EM64L-G09R
evE.01\HF=-2402.1147752\S2=0.754234\S2-1=0.\S2A=0.75001\RMSD=3.210e-09
\RMSF=4.721e-05\Dipole=-0.2927214,0.,0.\Quadrupole=1.2473852,-1.881214
4,0.6338292,0.,0.,0.\PG=C*V [C*(H1Se1)]\\@

1\1\GINC-ORC289\FOpt\UM062X\6-311+G(2df,p)\H1Se1(2)\MEISTERP\18-Aug-20
17\0\\# opt freq=noraman m062x/6-311+g(2df,p)\\SeH radical\\0,2\Se,-0.
3260300152,1.53392328,0.\H,-1.7929375048,1.53392328,0.\\Version=EM64L-
G09RevE.01\HF=-2402.1133256\S2=0.754543\S2-1=0.\S2A=0.750012\RMSD=8.26
8e-09\RMSF=1.343e-04\Dipole=-0.2415069,0.,0.\Quadrupole=1.3280456,-1.9
576106,0.629565,0.,0.,0.\PG=C*V [C*(H1Se1)]\\@

1\1\GINC-ORC170\FOpt\UM062X\6-311++G(3df,3pd)\H1Se1(2)\MEISTERP\18-Aug
-2017\0\\# opt freq=noraman m062x/6-311++g(3df,3pd)\\SeH radical\\0,2\
Se,-0.3262936627,1.53392328,0.\H,-1.7926738573,1.53392328,0.\\Version=
EM64L-G09RevE.01\HF=-2402.117066\S2=0.754543\S2-1=0.\S2A=0.750012\RMSD
=8.631e-09\RMSF=1.323e-08\Dipole=-0.2303904,0.,0.\Quadrupole=1.2402199
,-1.9029093,0.6626894,0.,0.,0.\PG=C*V [C*(H1Se1)]\\@

H-Se• M08HX

1\1\GINC-ORC105\FOpt\UM08HX\6-311+G(d,p)\H1Se1(2)\MEISTERP\18-Aug-2017
\0\\# opt freq=noraman m08hx/6-311+g(d,p)\\SeH radical\\0,2\Se,-0.3244
8376,1.53392328,0.\H,-1.79448376,1.53392328,0.\\Version=EM64L-G16RevA.
03\HF=-2402.1112094\S2=0.752796\S2-1=0.\S2A=0.750005\RMSD=8.578e-09\RM
SF=1.670e-04\Dipole=-0.3018128,0.,0.\Quadrupole=1.3471476,-2.0222257,0
.6750781,0.,0.,0.\PG=C*V [C*(H1Se1)]\\@

```
1\1\GINC-ORC25\FOpt\UM08HX\6-311G(df,p)\H1Se1(2)\MEISTERP\18-Aug-2017\
 0\\# opt freq=noraman m08hx/6-311g(df,p)\\SeH radical\\0,2\Se,-0.32448
 376,1.53392328,0.\H,-1.79448376,1.53392328,0.\\Version=EM64L-G16RevA.0
 3\HF=-2402.1129799\S2=0.752839\S2-1=0.\S2A=0.750005\RMSD=3.024e-09\RMS
 F=5.119e-06\Dipole=-0.294663,0.,0.\Quadrupole=1.2634719,-1.8487431,0.5
 852712,0.,0.,0.\PG=C*V [C*(H1Se1)]\\@
```

```
1\1\GINC-ORC23\FOpt\UB3LYP\6-311+G(2df,p)\H1Se1(2)\MEISTERP\18-Aug-201
 7\0\\# opt freq=noraman b3lyp/6-311+g(2df,p)\\SeH radical\\0,2\Se,-0.3
 221430178,1.53392328,0.\H,-1.7968245022,1.53392328,0.\\Version=EM64L-G
 16RevA.03\HF=-2402.1169647\S2=0.752713\S2-1=0.\S2A=0.750004\RMSD=1.052
 e-09\RMSF=1.223e-04\Dipole=-0.2087455,0.,0.\Quadrupole=1.3291422,-1.94
 93879,0.6202457,0.,0.,0.\PG=C*V [C*(H1Se1)]\\@
```

```
1\1\GINC-ORC23\FOpt\UB3LYP\6-311++G(3df,3pd)\H1Se1(2)\MEISTERP\18-Aug-
 2017\0\\# opt freq=noraman b3lyp/6-311++g(3df,3pd)\\SeH radical\\0,2\S
 e,-0.3226445284,1.53392328,0.\H,-1.7963229916,1.53392328,0.\\Version=E
 M64L-G16RevA.03\HF=-2402.1207973\S2=0.75278\S2-1=0.\S2A=0.750004\RMSD=
 5.962e-09\RMSF=9.153e-05\Dipole=-0.198587,0.,0.\Quadrupole=1.2464719,0
 .649561,-1.8960329,0.,0.,0.\PG=C*V [C*(H1Se1)]\\@
```

H-Se• QCISD

```
1\1\GINC-ORC79\SP\UQCISD-FC\6-311+G(2df,p)\H1Se1(2)\MEISTERP\01-Sep-20
 17\0\\# qcisd/6-311+g(2df,p)\\SeH radical qcisd single point\\0,2\Se,0
 ,0.,0.,0.041868\H,0,0.,0.,-1.423501\\Version=EM64L-G09RevE.01\HF=-2400
 .3670514\MP2=-2400.4957541\MP3=-2400.5152345\MP4D=-2400.5199823\MP4DQ=
 -2400.5167367\PUHF=-2400.3699969\PMP2-0=-2400.4974555\PMP3-0=-2400.516
 0926\MP4SDQ=-2400.5173276\QCISD=-2400.5176096\S2=0.761297\S2-1=0.75173
 2\S2A=0.750044\RMSD=3.371e-09\PG=C*V [C*(H1Se1)]\\@
```

CH_3-Se• B3LYP

```
1\1\GINC-GRA608\FOpt\UB3LYP\6-311+G(d,p)\C1H3Se1(2)\MEISTERP\01-Nov-20
 18\0\\# opt freq=noraman ub3lyp/6-311+g(d,p) guess=read\\Methyl seleny
 l radical test file for 2A' state from thiyl radical\\0,2\C,0.00646378
 86,0.0110729478,0.0126782626\H,-0.0006959801,-0.0011922662,1.1058115\H
 ,1.0378403596,-0.0057023125,-0.3344271287\H,-0.5152631969,0.9009156268
 ,-0.3344271281\Se,-0.9381777761,-1.6071678898,-0.5625804233\\Version=E
 M64L-G09RevE.01\State=2-A'\HF=-2441.4447453\S2=0.752308\S2-1=0.\S2A=0.
 750003\RMSD=4.769e-09\RMSF=8.448e-05\Dipole=0.2971761,0.5090847,0.2028
 627\Quadrupole=-1.1030572,0.3564094,0.7466479,1.2923319,0.1222498,0.20
 94229\PG=CS [SG(C1H1Se1),X(H2)]\\@
```

```
1\1\GINC-GRA608\FOpt\UB3LYP\6-311G(df,p)\C1H3Se1(2)\MEISTERP\01-Nov-20
 18\0\\# opt freq=noraman ub3lyp/6-311g(df, p) guess=read\\Methyl selen
 yl radical test file for 2A' state from thiyl radical\\0,2\C,0.0055835
 756,0.0095650777,0.0113111997\H,-0.0013020036,-0.0022304291,1.10427851
 37\H,1.0372484589,-0.005320797,-0.3349422817\H,-0.5146399522,0.9005878
 061,-0.334942281\Se,-0.9363223799,-1.6039894589,-0.5612870187\\Version
 =EM64L-G09RevE.01\State=2-A'\HF=-2441.4473332\S2=0.752688\S2-1=0.\S2A=
 0.750004\RMSD=3.408e-09\RMSF=7.554e-05\Dipole=0.2888372,0.4947994,0.19
 91098\Quadrupole=-1.0088707,0.3331362,0.6757345,1.1883235,0.1193546,0.
 2044633\PG=CS [SG(C1H1Se1),X(H2)]\\@
```

```
1\1\GINC-GRA608\FOpt\UB3LYP\6-311+G(2df,p)\C1H3Se1(2)\MEISTERP\01-Nov-
 2018\0\\# opt freq=noraman ub3lyp/6-311+g(2df,p) guess=read\\Methyl se
```

lenyl radical test file for 2A' state from thiyl radical\\0,2\C,0.0054
032024,0.0092560851,0.0114142668\H,-0.0011380847,-0.0019496239,1.10354
8934\H,1.0361728142,-0.0057917142,-0.3343192176\H,-0.5145211265,0.8994
196216,-0.334319217\Se,-0.9355003259,-1.6025812196,-0.5608923054\\Vers
ion=EM64L-G09RevE.01\State=2-A'\HF=-2441.4462049\S2=0.752802\S2-1=0.\S
2A=0.750005\RMSD=3.774e-09\RMSF=1.016e-04\Dipole=0.2915663,0.4994746,0
.1969147\Quadrupole=-1.0499705,0.3607459,0.6892245,1.2491645,0.1250825
,0.2142756\PG=CS [SG(C1H1Se1),X(H2)]\\@

1\1\GINC-GRA608\FOpt\UB3LYP\6-311++G(3df,3pd)\C1H3Se1(2)\MEISTERP\01-N
ov-2018\0\\# opt freq=noraman ub3lyp/6-311++g(3df,3pd) guess=read\\Met
hyl selenyl radical test file for 2A' state from thiyl radical\\0,2\C,
0.005770094,0.0098845976,0.0110396236\H,-0.0012705419,-0.0021765329,1.
1013995392\H,1.0348593702,-0.0057447911,-0.3339335709\H,-0.5138344573,
0.8982989867,-0.3339335703\Se,-0.9348941421,-1.601542782,-0.5605152057
\\Version=EM64L-G09RevE.01\State=2-A'\HF=-2441.4514955\S2=0.75288\S2-1
=0.\S2A=0.750005\RMSD=3.433e-09\RMSF=1.614e-04\Dipole=0.2872942,0.4921
562,0.1921713\Quadrupole=-1.0306023,0.3326921,0.6979101,1.2071732,0.11
31921,0.1939065\PG=CS [SG(C1H1Se1),X(H2)]\\@

CH_3-Se$^{\bullet}$ B3PW91

1\1\GINC-GRA1101\FOpt\UB3PW91\6-311+G(d,p)\C1H3Se1(2)\MEISTERP\31-Oct-
2018\0\\# opt freq=noraman ub3pw91/6-311+g(d,p) guess=read\\Methyl sel
enyl radical test file for 2A' state from thiyl radical\\0,2\C,0.00424
45883,0.0072712936,0.0089031159\H,-0.0018322357,-0.003138756,1.1028586
498\H,1.0376337701,-0.0066665064,-0.3352915704\H,-0.5160012089,0.90026
16486,-0.3352915697\Se,-0.9330605641,-1.5984017273,-0.5595143414\\Vers
ion=EM64L-G09RevE.01\State=2-A'\HF=-2441.4082685\S2=0.752771\S2-1=0.\S
2A=0.750004\RMSD=8.834e-09\RMSF=9.189e-05\Dipole=0.3015456,0.5165699,0
.2076605\Quadrupole=-1.0876909,0.3669191,0.7207718,1.2880315,0.132088,
0.2262764\PG=CS [SG(C1H1Se1),X(H2)]\\@

1\1\GINC-GRA1101\FOpt\UB3PW91\6-311G(df,p)\C1H3Se1(2)\MEISTERP\31-Oct-
2018\0\\# opt freq=noraman ub3pw91/6-311g(df, p) guess=read\\Methyl se
lenyl radical test file for 2A' state from thiyl radical\\0,2\C,0.0032
874421,0.0056316313,0.0071908033\H,-0.0025016321,-0.0042854815,1.10107
56623\H,1.0370533406,-0.0062158468,-0.3358914036\H,-0.5153233959,0.899
9778143,-0.3358914029\Se,-0.9310269159,-1.5949179376,-0.5581207763\\Ve
rsion=EM64L-G09RevE.01\State=2-A'\HF=-2441.4111159\S2=0.753326\S2-1=0.
\S2A=0.750006\RMSD=8.680e-09\RMSF=1.754e-04\Dipole=0.2957842,0.5067003
,0.2053272\Quadrupole=-1.0033386,0.3466762,0.6566623,1.1954143,0.12990
03,0.2225288\PG=CS [SG(C1H1Se1),X(H2)]\\@

1\1\GINC-GRA1101\FOpt\UB3PW91\6-311+G(2df,p)\C1H3Se1(2)\MEISTERP\31-Oc
t-2018\0\\# opt freq=noraman ub3pw91/6-311+g(2df,p) guess=read\\Methyl
 selenyl radical test file for 2A' state from thiyl radical\\0,2\C,0.0
031683302,0.0054275838,0.0074788962\H,-0.0023774736,-0.0040727888,1.10
07314709\H,1.0361847526,-0.0067880205,-0.3353947632\H,-0.5153945494,0.
8989401413,-0.3353947626\Se,-0.930286783,-1.5936500352,-0.5577646637\\
Version=EM64L-G09RevE.01\State=2-A'\HF=-2441.4097434\S2=0.753489\S2-1=
0.\S2A=0.750007\RMSD=2.777e-09\RMSF=1.982e-04\Dipole=0.2962778,0.50754
58,0.2015566\Quadrupole=-1.0366247,0.3716193,0.6650054,1.2469752,0.134
7071,0.2307632\PG=CS [SG(C1H1Se1),X(H2)]\\@

1\1\GINC-GRA1101\FOpt\UB3PW91\6-311++G(3df,3pd)\C1H3Se1(2)\MEISTERP\31
-Oct-2018\0\\# opt freq=noraman ub3pw91/6-311++g(3df,3pd) guess=read\\
Methyl selenyl radical test file for 2A' state from thiyl radical\\0,2

\C,0.0041367527,0.0070865632,0.0084178818\H,-0.0027151143,-0.004651192
3,1.1002479402\H,1.0353434126,-0.0076676524,-0.3353953112\H,-0.5157468
255,0.8977750205,-0.3353953106\Se,-0.9298347722,-1.5928757073,-0.55746
244\\Version=EM64L-G09RevE.01\State=2-A'\HF=-2441.4149987\S2=0.753602\
S2-1=0.\S2A=0.750007\RMSD=4.749e-09\RMSF=8.076e-06\Dipole=0.2920875,0.
5003675,0.1968931\Quadrupole=-1.0168219,0.34004,0.6767819,1.2014773,0.
1202993,0.2060815\PG=CS [SG(C1H1Se1),X(H2)]\\@

CH_3-Se$^{\bullet}$ wB97XD

1\1\GINC-GRA1101\FOpt\UwB97XD\6-311+G(d,p)\C1H3Se1(2)\MEISTERP\31-Oct-
2018\0\\# opt freq=noraman uwb97xd/6-311+g(d,p) guess=read\\Methyl sel
enyl radical test file for 2A' state from thiyl radical\\0,2\C,0.00314
95505,0.0053954129,0.0079592873\H,-0.0016690347,-0.0028591806,1.101672
5634\H,1.0364683868,-0.0063290353,-0.3347807298\H,-0.5151343398,0.8994
128006,-0.3347807292\Se,-0.9317723352,-1.5961948959,-0.558788382\\Vers
ion=EM64L-G09RevE.01\State=2-A'\HF=-2441.451227\S2=0.752648\S2-1=0.\S2
A=0.750004\RMSD=5.531e-09\RMSF=1.277e-04\Dipole=0.2998985,0.5137483,0.
2059797\Quadrupole=-1.0994666,0.369652,0.7298146,1.3008786,0.1317841,0
.2257558\PG=CS [SG(C1H1Se1),X(H2)]\\@

1\1\GINC-GRA1101\FOpt\UwB97XD\6-311G(df,p)\C1H3Se1(2)\MEISTERP\31-Oct-
2018\0\\# opt freq=noraman uwb97xd/6-311g(df, p) guess=read\\Methyl se
lenyl radical test file for 2A' state from thiyl radical\\0,2\C,0.0024
803811,0.0042490763,0.0062581288\H,-0.0023915076,-0.0040968301,1.09994
11935\H,1.036147388,-0.0057993346,-0.3355354568\H,-0.5145152602,0.8993
937343,-0.3355354561\Se,-0.9301058922,-1.5933401558,-0.5576061573\\Ver
sion=EM64L-G09RevE.01\State=2-A'\HF=-2441.4539853\S2=0.753007\S2-1=0.\
S2A=0.750006\RMSD=3.586e-09\RMSF=1.659e-04\Dipole=0.2926286,0.5012944,
0.2027809\Quadrupole=-1.0135185,0.3458145,0.667704,1.2036654,0.1278171
,0.2189602\PG=CS [SG(C1H1Se1),X(H2)]\\@

1\1\GINC-GRA1101\FOpt\UwB97XD\6-311+G(2df,p)\C1H3Se1(2)\MEISTERP\31-Oc
t-2018\0\\# opt freq=noraman uwb97xd/6-311+g(2df,p) guess=read\\Methyl
selenyl radical test file for 2A' state from thiyl radical\\0,2\C,0.0
023016567,0.0039429081,0.006255484\H,-0.0022989159,-0.0039382136,1.099
3284502\H,1.0352932449,-0.0061459686,-0.3351024036\H,-0.5143971231,0.8
984795357,-0.335102403\Se,-0.9293767991,-1.5920911655,-0.5572416448\\V
ersion=EM64L-G09RevE.01\State=2-A'\HF=-2441.4526428\S2=0.753105\S2-1=0
.\S2A=0.750006\RMSD=5.515e-09\RMSF=1.684e-04\Dipole=0.2944815,0.504468
6,0.1988147\Quadrupole=-1.0471567,0.3794888,0.6676678,1.2632694,0.1364
252,0.2337065\PG=CS [SG(C1H1Se1),X(H2)]\\@

1\1\GINC-GRA1101\FOpt\UwB97XD\6-311++G(3df,3pd)\C1H3Se1(2)\MEISTERP\31
-Oct-2018\0\\# opt freq=noraman uwb97xd/6-311++g(3df,3pd) guess=read\\
Methyl selenyl radical test file for 2A' state from thiyl radical\\0,2
\C,0.0027937096,0.004785831,0.0060672318\H,-0.0024459359,-0.0041900698
,1.0973666275\H,1.034020616,-0.0061883425,-0.3347618864\H,-0.513808279
5,0.8973505347,-0.3347618858\Se,-0.9288615071,-1.5912084322,-0.5569201
466\\Version=EM64L-G09RevE.01\State=2-A'\HF=-2441.4581739\S2=0.75317\S
2-1=0.\S2A=0.750006\RMSD=9.566e-09\RMSF=1.799e-04\Dipole=0.2918838,0.5
000186,0.195097\Quadrupole=-1.0297609,0.3543862,0.6753747,1.2256379,0.
1248061,0.2138021\PG=CS [SG(C1H1Se1),X(H2)]\\@

CH_3-Se$^{\bullet}$ M062X

1\1\GINC-GRA1101\FOpt\UM062X\6-311+G(d,p)\C1H3Se1(2)\MEISTERP\31-Oct-2

018\0\\# opt freq=noraman um062x/6-311+g(d,p) guess=read\\Methyl selen
yl radical test file for 2A' state from thiyl radical\\0,2\C,0.0044069
034,0.0075493513,0.0126571526\H,-0.0013135987,-0.0022502925,1.10418984
13\H,1.0344733545,-0.007102589,-0.333952349\H,-0.5148269852,0.89729523
78,-0.3339523484\Se,-0.9329585565,-1.5982269807,-0.5593014164\\Version
=EM64L-G09RevE.01\State=2-A'\HF=-2441.4182217\S2=0.754541\S2-1=0.\S2A=
0.750013\RMSD=5.358e-09\RMSF=1.337e-04\Dipole=0.3053,0.5230015,0.20634
54\Quadrupole=-1.1111829,0.3216829,0.7895001,1.2687774,0.1040837,0.178
3031\PG=CS [SG(C1H1Se1),X(H2)]\\@

1\1\GINC-GRA1101\FOpt\UM062X\6-311G(df,p)\C1H3Se1(2)\MEISTERP\31-Oct-2
018\0\\# opt freq=noraman um062x/6-311g(df, p) guess=read\\Methyl sele
nyl radical test file for 2A' state from thiyl radical\\0,2\C,0.003430
1892,0.0058761676,0.0107816807\H,-0.0019194832,-0.0032882173,1.1023930
03\H,1.0341021212,-0.0064123098,-0.3345680427\H,-0.5140433789,0.897311
3841,-0.334568042\Se,-0.9312280652,-1.5952625211,-0.5580904082\\Versio
n=EM64L-G09RevE.01\State=2-A'\HF=-2441.4209051\S2=0.754546\S2-1=0.\S2A
=0.750012\RMSD=4.856e-09\RMSF=1.768e-04\Dipole=0.2977621,0.5100885,0.2
033885\Quadrupole=-1.0165676,0.2970483,0.7195193,1.1631837,0.1003439,0
.1718966\PG=CS [SG(C1H1Se1),X(H2)]\\@

1\1\GINC-GRA1101\FOpt\UM062X\6-311+G(2df,p)\C1H3Se1(2)\MEISTERP\31-Oct
-2018\0\\# opt freq=noraman um062x/6-311+g(2df,p) guess=read\\Methyl s
elenyl radical test file for 2A' state from thiyl radical\\0,2\C,0.003
5064279,0.0060067703,0.0110623689\H,-0.0019238317,-0.0032956667,1.1018
744217\H,1.0332225296,-0.0070476583,-0.3341965431\H,-0.5141641326,0.89
6233067,-0.3341965425\Se,-0.930435719,-1.5939051735,-0.5576813223\\Ver
sion=EM64L-G09RevE.01\State=2-A'\HF=-2441.4202586\S2=0.754924\S2-1=0.\
S2A=0.750015\RMSD=4.149e-09\RMSF=2.032e-04\Dipole=0.2961779,0.5073747,
0.1976707\Quadrupole=-1.0485005,0.3339907,0.7145097,1.2241716,0.109311
7,0.187259\PG=CS [SG(C1H1Se1),X(H2)]\\@

1\1\GINC-GRA1101\FOpt\UM062X\6-311++G(3df,3pd)\C1H3Se1(2)\MEISTERP\31-
Oct-2018\0\\# opt freq=noraman um062x/6-311++g(3df,3pd) guess=read\\Me
thyl selenyl radical test file for 2A' state from thiyl radical\\0,2\C
,0.0044489809,0.0076214332,0.0123172421\H,-0.0022638859,-0.0038782047,
1.1013994911\H,1.0319641164,-0.0078552727,-0.3341196434\H,-0.514248626
6,0.894740182,-0.3341196427\Se,-0.9298929815,-1.592975424,-0.557278533
1\\Version=EM64L-G09RevE.01\State=2-A'\HF=-2441.4256554\S2=0.754945\S2
-1=0.\S2A=0.750016\RMSD=3.429e-09\RMSF=1.622e-05\Dipole=0.2912939,0.49
90079,0.1926021\Quadrupole=-1.0280682,0.3056262,0.7224421,1.1809629,0.
0964513,0.1652281\PG=CS [SG(C1H1Se1),X(H2)]\\@

CH_3-Se$^\bullet$ M08HX

1\1\GINC-ORC362\FOpt\UM08HX\6-311+G(d,p)\C1H3Se1(2)\MEISTERP\18-Aug-20
17\0\\# opt freq=noraman m08hx/6-311+g(d,p)\\Methyl Selenide\\0,2\C,-0
.4078875865,1.9513072563,-0.0047156199\H,-0.0266440009,0.9240008325,0.
0247806255\H,-0.0275032444,2.4426570689,-0.902304169\H,-1.4991853335,1
.9223348021,-0.0010972643\Se,0.2600749415,2.8305894665,1.5936858153\\V
ersion=EM64L-G16RevA.03\State=2-A\HF=-2441.4268421\S2=0.752906\S2-1=0.
\S2A=0.750006\RMSD=4.808e-09\RMSF=3.236e-05\Dipole=-0.2141067,-0.31479
07,-0.5313648\Quadrupole=-0.9767039,0.6362606,0.3404433,-0.4991595,1.1
169039,0.6129399\PG=C01 [X(C1H3Se1)]\\@

1\1\GINC-ORC294\FOpt\UM08HX\6-311G(df,p)\C1H3Se1(2)\MEISTERP\18-Aug-20
17\0\\# opt freq=noraman m08hx/6-311g(df,p)\\Methyl Selenide\\0,2\C,-0

.3880499462,1.9181658438,0.0087077646\H,-0.0347487968,0.8863059705,-0.
0102773512\H,-0.0370022087,2.4578119755,-0.8761355256\H,-1.4819114144,
1.9469375281,0.0084521325\Se,0.2405671424,2.8616681083,1.5796023675\\V
ersion=EM64L-G16RevA.03\State=2-A\HF=-2441.4296655\S2=0.752992\S2-1=0.
\S2A=0.750006\RMSD=8.083e-09\RMSF=2.857e-05\Dipole=-0.2198291,-0.28794
58,-0.5252717\Quadrupole=0.1397401,-0.8922969,0.7525568,0.9668124,-0.1
502161,0.7218317\PG=C01 [X(C1H3Se1)]\\@

1\1\GINC-ORC176\FOpt\UM08HX\6-311+G(2df,p)\C1H3Se1(2)\MEISTERP\18-Aug-
2017\0\\# opt freq=noraman m08hx/6-311+g(2df,p)\\Methyl Selenide\\0,2\
C,-0.4072953561,1.9522794353,-0.0031968635\H,-0.0266153724,0.925698157
2,0.0258126626\H,-0.0279883917,2.442394595,-0.9008654826\H,-1.49771951
69,1.9227605924,-0.0008431963\Se,0.2584734135,2.8277566464,1.589442267
5\\Version=EM64L-G16RevA.03\State=2-A\HF=-2441.4296\S2=0.753371\S2-1=0
.\S2A=0.750008\RMSD=5.793e-10\RMSF=3.181e-05\Dipole=-0.2064259,-0.2994
713,-0.5099716\Quadrupole=-0.925262,0.5808013,0.3444607,-0.4515205,1.0
614182,0.6000856\PG=C01 [X(C1H3Se1)]\\@

1\1\GINC-ORC112\FOpt\UM08HX\6-311++G(3df,3pd)\C1H3Se1(2)\MEISTERP\18-A
ug-2017\0\\# opt freq=noraman m08hx/6-311++g(3df,3pd)\\Methyl Selenide
\\0,2\C,-0.4080016244,1.9515998539,-0.0047161367\H,-0.0270317842,0.927
7863482,0.0263206478\H,-0.0282696841,2.441724241,-0.8990422994\H,-1.49
58398307,1.9228608342,-0.0003730981\Se,0.2579976995,2.826918149,1.5881
602741\\Version=EM64L-G16RevA.03\State=2-A\HF=-2441.4353311\S2=0.75336
6\S2-1=0.\S2A=0.750008\RMSD=8.566e-09\RMSF=3.478e-05\Dipole=-0.2054635
,-0.2958801,-0.506356\Quadrupole=-0.9028885,0.5758345,0.327054,-0.4517
055,1.0365217,0.5727346\PG=C01 [X(C1H3Se1)]\\@

CH3-Se• QCISD

1\1\GINC-GRA1058\FOpt\UQCISD-FC\6-311+G(2df,p)\C1H3Se1(2)\MEISTERP\02-
Nov-2018\0\\# opt freq=noraman qcisd/6-311+g(2df,p) guess=read\\Methyl
 selenyl radical test file for 2A' state from thiyl radical\\0,2\C,0.0
036604035,0.0062705418,0.0137899228\H,-0.0011817061,-0.0020243506,1.10
72235624\H,1.0356243126,-0.0060683131,-0.3345685336\H,-0.5144922864,0.
8988060019,-0.334568533\Se,-0.9342051624,-1.6003625087,-0.5597885628\\
Version=EM64L-G09RevE.01\State=2-A'\HF=-2439.412499\MP2=-2439.7105079\
MP3=-2439.7396147\MP4D=-2439.7479907\MP4DQ=-2439.7406234\PUHF=-2439.41
55427\PMP2-0=-2439.7122859\PMP3-0=-2439.7405221\MP4SDQ=-2439.7428106\Q
CISD=-2439.7430819\S2=0.762051\S2-1=0.751998\S2A=0.750062\RMSD=4.528e-
09\RMSF=6.580e-05\Dipole=0.2855273,0.4891293,0.1905045\PG=CS [SG(C1H1S
e1),X(H2)]\\@

CH_3-Se-Se• B3LYP

1\1\GINC-ORC315\FOpt\UB3LYP\6-311+G(d,p)\C1H3Se2(2)\MEISTERP\24-Aug-20
17\0\\# opt freq=noraman b3lyp/6-311+g(d,p)\\Methyl Se Se optimization
 and frequency\\0,2\C,1.992434319,0.9300790898,0.\H,2.3330815606,0.415
3465982,0.895647165\H,2.3311773066,1.9671427248,0.\H,2.3330815606,0.41
53465982,-0.895647165\Se,-0.5631136677,-1.2191882923,0.\Se,0.011016920
8,0.9759862814,0.\\Version=EM64L-G09RevE.01\State=2-A"\HF=-4843.035561
\S2=0.752799\S2-1=0.\S2A=0.750006\RMSD=6.146e-09\RMSF=1.391e-05\Dipole
=0.7823907,0.5767946,0.\Quadrupole=0.6164178,0.307144,-0.9235619,1.577
1617,0.,0.\PG=CS [SG(C1H1Se2),X(H2)]\\@

1\1\GINC-ORC315\FOpt\UB3LYP\6-311G(df,p)\C1H3Se2(2)\MEISTERP\24-Aug-20
17\0\\# opt freq=noraman b3lyp/6-311g(df,p)\\Methyl Se Se optimization

```
 and frequency\\0,2\C,1.989779653,0.9293491919,0.\H,2.3318407583,0.414
 5288064,0.8949032332\H,2.3283791297,1.9660694599,0.\H,2.3318407583,0.4
 145288064,-0.8949032332\Se,-0.5582357827,-1.2109098034,0.\Se,0.0140734
 832,0.9711465389,0.\\Version=EM64L-G09RevE.01\State=2-A"\HF=-4843.0412
 465\S2=0.753131\S2-1=0.\S2A=0.750007\RMSD=4.675e-09\RMSF=1.937e-05\Dip
 ole=0.768184,0.5630598,0.\Quadrupole=0.673421,0.1655763,-0.8389973,1.4
 790561,0.,0.\PG=CS [SG(C1H1Se2),X(H2)]\\@
```

```
1\1\GINC-ORC315\FOpt\UB3LYP\6-311+G(2df,p)\C1H3Se2(2)\MEISTERP\24-Aug-
 2017\0\\# opt freq=noraman b3lyp/6-311+g(2df,p)\\Methyl Se Se optimiza
 tion and frequency\\0,2\C,1.9894323213,0.9286530949,0.\H,2.3310111318,
 0.4148311998,0.8946173449\H,2.3270711037,1.9648609389,0.\H,2.331011131
 8,0.4148311998,-0.8946173449\Se,-0.5567047878,-1.2093537869,0.\Se,0.01
 58570991,0.9708903534,0.\\Version=EM64L-G09RevE.01\State=2-A"\HF=-4843
 .0376333\S2=0.753609\S2-1=0.\S2A=0.750009\RMSD=5.806e-09\RMSF=2.110e-0
 5\Dipole=0.7645063,0.5617328,0.\Quadrupole=0.6608314,0.2751701,-0.9360
 015,1.5540683,0.,0.\PG=CS [SG(C1H1Se2),X(H2)]\\@
```

```
1\1\GINC-ORC314\FOpt\UB3LYP\6-311++G(3df,3pd)\C1H3Se2(2)\MEISTERP\24-A
 ug-2017\0\\# opt freq=noraman b3lyp/6-311++g(3df,3pd)\\Methyl Se Se op
 timization and frequency\\0,2\C,1.9901467949,0.9288730989,0.\H,2.32978
 80725,0.4153417791,0.8934389239\H,2.327011345,1.9634802386,0.\H,2.3297
 880725,0.4153417791,-0.8934389239\Se,-0.5559207912,-1.2096374495,0.\Se
 ,0.0168645064,0.9713135537,0.\\Version=EM64L-G09RevE.01\State=2-A"\HF=
 -4843.0466186\S2=0.753676\S2-1=0.\S2A=0.75001\RMSD=8.468e-09\RMSF=1.98
 5e-05\Dipole=0.7561612,0.565144,0.\Quadrupole=0.6226249,0.2590841,-0.8
 81709,1.5246288,0.,0.\PG=CS [SG(C1H1Se2),X(H2)]\\@
```

CH_3-Se-Se• B3PW91

```
1\1\GINC-ORC314\FOpt\UB3PW91\6-311+G(d,p)\C1H3Se2(2)\MEISTERP\24-Aug-2
 017\0\\# opt freq=noraman b3pw91/6-311+g(d,p)\\Methyl Se Se optimizati
 on and frequency\\0,2\C,1.9763437582,0.9156880773,0.\H,2.3187503558,0.
 4005104223,0.8958213866\H,2.3198499293,1.9520627818,0.\H,2.3187503558,
 0.4005104223,-0.8958213866\Se,-0.563660904,-1.2078624119,0.\Se,0.01180
 9505,0.9659197081,0.\\Version=EM64L-G09RevE.01\State=2-A"\HF=-4842.980
 2732\S2=0.752962\S2-1=0.\S2A=0.750007\RMSD=8.229e-09\RMSF=1.447e-05\Di
 pole=0.8001224,0.5711778,0.\Quadrupole=0.6816522,0.2624145,-0.9440667,
 1.5840729,0.,0.\PG=CS [SG(C1H1Se2),X(H2)]\\@
```

```
1\1\GINC-ORC314\FOpt\UB3PW91\6-311G(df,p)\C1H3Se2(2)\MEISTERP\24-Aug-2
 017\0\\# opt freq=noraman b3pw91/6-311g(df,p)\\Methyl Se Se optimizati
 on and frequency\\0,2\C,1.9738305029,0.9149819298,0.\H,2.3173450661,0.
 3996277687,0.8951522591\H,2.3170758466,1.9510562169,0.\H,2.3173450661,
 0.3996277687,-0.8951522591\Se,-0.5586595318,-1.1998165304,0.\Se,0.0149
 060502,0.9613518464,0.\\Version=EM64L-G09RevE.01\State=2-A"\HF=-4842.9
 864313\S2=0.753452\S2-1=0.\S2A=0.750009\RMSD=6.823e-09\RMSF=1.833e-05\
 Dipole=0.7906066,0.5621829,0.\Quadrupole=0.739669,0.117104,-0.8567729,
 1.4941499,0.,0.\PG=CS [SG(C1H1Se2),X(H2)]\\@
```

```
1\1\GINC-ORC311\FOpt\UB3PW91\6-311+G(2df,p)\C1H3Se2(2)\MEISTERP\24-Aug
 -2017\0\\# opt freq=noraman b3pw91/6-311+g(2df,p)\\Methyl Se Se optimi
 zation and frequency\\0,2\C,1.9735763869,0.9144307284,0.\H,2.316096716
 5,0.3997296654,0.8950538244\H,2.3163599448,1.9500336492,0.\H,2.3160967
 165,0.3997296654,-0.8950538244\Se,-0.5566214797,-1.1985529931,0.\Se,0.
 016334715,0.9614582847,0.\\Version=EM64L-G09RevE.01\State=2-A"\HF=-484
 2.9824396\S2=0.754055\S2-1=0.\S2A=0.750012\RMSD=8.567e-09\RMSF=1.978e-
```

05\Dipole=0.7816665,0.5546892,0.\Quadrupole=0.7206986,0.2551238,-0.975
8224,1.5746216,0.,0.\PG=CS [SG(C1H1Se2),X(H2)]\\@

1\1\GINC-ORC311\FOpt\UB3PW91\6-311++G(3df,3pd)\C1H3Se2(2)\MEISTERP\24-
Aug-2017\0\\# opt freq=noraman b3pw91/6-311++g(3df,3pd)\\Methyl Se Se
optimization and frequency\\0,2\C,1.974129956,0.9147217829,0.\H,2.3148
513546,0.4001043113,0.8940058541\H,2.3165118477,1.9488589856,0.\H,2.31
48513546,0.4001043113,-0.8940058541\Se,-0.5557136488,-1.1987943036,0.\
Se,0.0172121359,0.9618339126,0.\\Version=EM64L-G09RevE.01\State=2-A"\H
F=-4842.9915478\S2=0.754131\S2-1=0.\S2A=0.750012\RMSD=9.212e-09\RMSF=1
.917e-05\Dipole=0.7726372,0.5592728,0.\Quadrupole=0.6739489,0.2474426,
-0.9213915,1.5454089,0.,0.\PG=CS [SG(C1H1Se2),X(H2)]\\@

CH_3-Se-Se$^•$ wB97XD

1\1\GINC-ORC306\FOpt\UwB97XD\6-311+G(d,p)\C1H3Se2(2)\MEISTERP\24-Aug-2
017\0\\# opt freq=noraman wb97xd/6-311+g(d,p)\\Methyl Se Se optimizati
on and frequency\\0,2\C,1.9664725066,0.8975111017,0.\H,2.3066819635,0.
3796119573,0.8945744779\H,2.324350209,1.9283136253,0.\H,2.3066819635,0
.3796119573,-0.8945744779\Se,-0.5605821434,-1.2010576252,0.\Se,0.00975
65007,0.9656509836,0.\\Version=EM64L-G09RevE.01\State=2-A"\HF=-4843.06
44886\S2=0.754653\S2-1=0.\S2A=0.750014\RMSD=4.736e-09\RMSF=1.727e-05\D
ipole=0.7924052,0.5485472,0.\Quadrupole=0.6754968,0.2657117,-0.9412085
,1.6046458,0.,0.\PG=CS [SG(C1H1Se2),X(H2)]\\@

1\1\GINC-ORC305\FOpt\UwB97XD\6-311G(df,p)\C1H3Se2(2)\MEISTERP\24-Aug-2
017\0\\# opt freq=noraman wb97xd/6-311g(df,p)\\Methyl Se Se optimizati
on and frequency\\0,2\C,1.9646243261,0.8969897481,0.\H,2.3058561293,0.
3789685679,0.8939984096\H,2.3215561389,1.927753401,0.\H,2.3058561293,0
.3789685679,-0.8939984096\Se,-0.5570771528,-1.1932805273,0.\Se,0.01254
54293,0.9602422425,0.\\Version=EM64L-G09RevE.01\State=2-A"\HF=-4843.07
10242\S2=0.755136\S2-1=0.\S2A=0.750018\RMSD=9.901e-09\RMSF=2.290e-05\D
ipole=0.7830846,0.5404773,0.\Quadrupole=0.7237767,0.1101366,-0.8339133
,1.4984221,0.,0.\PG=CS [SG(C1H1Se2),X(H2)]\\@

1\1\GINC-ORC305\FOpt\UwB97XD\6-311+G(2df,p)\C1H3Se2(2)\MEISTERP\24-Aug
-2017\0\\# opt freq=noraman wb97xd/6-311+g(2df,p)\\Methyl Se Se optimi
zation and frequency\\0,2\C,1.9642248751,0.8964181145,0.\H,2.305518543
8,0.379500276,0.8937660973\H,2.3205163809,1.926766315,0.\H,2.305518543
8,0.379500276,-0.8937660973\Se,-0.5565782002,-1.1921759258,0.\Se,0.014
1608567,0.9596329443,0.\\Version=EM64L-G09RevE.01\State=2-A"\HF=-4843.
0670898\S2=0.755815\S2-1=0.\S2A=0.750021\RMSD=3.094e-09\RMSF=2.409e-05
\Dipole=0.7762939,0.5336518,0.\Quadrupole=0.7162838,0.2701669,-0.98645
07,1.605867,0.,0.\PG=CS [SG(C1H1Se2),X(H2)]\\@

1\1\GINC-ORC305\FOpt\UwB97XD\6-311++G(3df,3pd)\C1H3Se2(2)\MEISTERP\24-
Aug-2017\0\\# opt freq=noraman wb97xd/6-311++g(3df,3pd)\\Methyl Se Se
optimization and frequency\\0,2\C,1.9649217179,0.8967136694,0.\H,2.304
0635222,0.379962356,0.8925648259\H,2.3207414256,1.9253710474,0.\H,2.30
40635222,0.379962356,-0.8925648259\Se,-0.5554582368,-1.1924876072,0.\S
e,0.0150290489,0.9601201783,0.\\Version=EM64L-G09RevE.01\State=2-A"\HF
=-4843.0765424\S2=0.755966\S2-1=0.\S2A=0.750022\RMSD=6.154e-09\RMSF=2.
341e-05\Dipole=0.7706384,0.5391878,0.\Quadrupole=0.6829737,0.2621374,-
0.9451111,1.5862362,0.,0.\PG=CS [SG(C1H1Se2),X(H2)]\\@

CH_3-Se-Se• M062X

1\1\GINC-ORC311\FOpt\UM062X\6-311+G(d,p)\C1H3Se2(2)\MEISTERP\24-Aug-20
17\0\\# opt freq=noraman m062x/6-311+g(d,p)\\Methyl Se Se optimization
and frequency\\0,2\C,1.9669930972,0.8656190449,0.\H,2.290798125,0.340
5141256,0.8946605042\H,2.3432158553,1.8884273257,0.\H,2.290798125,0.34
05141256,-0.8946605042\Se,-0.5653090628,-1.2023937531,0.\Se,0.00884886
03,0.9749111313,0.\\Version=EM64L-G09RevE.01\State=2-A"\HF=-4843.00441
65\S2=0.754978\S2-1=0.\S2A=0.750017\RMSD=3.110e-09\RMSF=1.449e-05\Dipo
le=0.7663604,0.4309169,0.\Quadrupole=0.7160809,0.1796088,-0.8956897,1.
6273678,0.,0.\PG=CS [SG(C1H1Se2),X(H2)]\\@

1\1\GINC-ORC310\FOpt\UM062X\6-311G(df,p)\C1H3Se2(2)\MEISTERP\24-Aug-20
17\0\\# opt freq=noraman m062x/6-311g(df,p)\\Methyl Se Se optimization
and frequency\\0,2\C,1.9648500117,0.8651236135,0.\H,2.2906904091,0.34
00659409,0.8940636817\H,2.3404286761,1.8879682099,0.\H,2.2906904091,0.
3400659409,-0.8940636817\Se,-0.562873521,-1.1957570631,0.\Se,0.0115590
152,0.9701253579,0.\\Version=EM64L-G09RevE.01\State=2-A"\HF=-4843.0106
406\S2=0.754872\S2-1=0.\S2A=0.750016\RMSD=6.646e-09\RMSF=1.907e-05\Dip
ole=0.7563121,0.4165847,0.\Quadrupole=0.7593209,0.0301875,-0.7895084,1
.5243552,0.,0.\PG=CS [SG(C1H1Se2),X(H2)]\\@

1\1\GINC-ORC310\FOpt\UM062X\6-311+G(2df,p)\C1H3Se2(2)\MEISTERP\24-Aug-
2017\0\\# opt freq=noraman m062x/6-311+g(2df,p)\\Methyl Se Se optimiza
tion and frequency\\0,2\C,1.9652455175,0.8646066037,0.\H,2.2899395139,
0.3404784367,0.8939736165\H,2.3392789895,1.8872234773,0.\H,2.289939513
9,0.3404784367,-0.8939736165\Se,-0.5622214945,-1.1946528093,0.\Se,0.01
31629596,0.9694578548,0.\\Version=EM64L-G09RevE.01\State=2-A"\HF=-4843
.0078189\S2=0.756057\S2-1=0.\S2A=0.750025\RMSD=9.699e-09\RMSF=2.148e-0
5\Dipole=0.7409389,0.4106745,0.\Quadrupole=0.7481155,0.1815099,-0.9296
255,1.6219046,0.,0.\PG=CS [SG(C1H1Se2),X(H2)]\\@

1\1\GINC-ORC310\FOpt\UM062X\6-311++G(3df,3pd)\C1H3Se2(2)\MEISTERP\24-A
ug-2017\0\\# opt freq=noraman m062x/6-311++g(3df,3pd)\\Methyl Se Se op
timization and frequency\\0,2\C,1.9657075159,0.8649751272,0.\H,2.28828
359,0.3409717486,0.8925255181\H,2.3398709067,1.8855275378,0.\H,2.28828
359,0.3409717486,-0.8925255181\Se,-0.5605290302,-1.1947619469,0.\Se,0.
0137284277,0.9699077847,0.\\Version=EM64L-G09RevE.01\State=2-A"\HF=-48
43.0170382\S2=0.756097\S2-1=0.\S2A=0.750025\RMSD=7.664e-09\RMSF=1.971e
-05\Dipole=0.7329107,0.4214792,0.\Quadrupole=0.712671,0.1775204,-0.890
1914,1.5918889,0.,0.\PG=CS [SG(C1H1Se2),X(H2)]\\@

CH_3-Se-Se• M08HX

1\1\GINC-ORC308\FOpt\UM08HX\6-311+G(d,p)\C1H3Se2(2)\MEISTERP\24-Aug-20
17\0\\# opt freq=noraman m08hx/6-311+g(d,p)\\Methyl Se Se optimization
and frequency\\0,2\C,1.9676002032,0.8447398261,0.\H,2.2844637984,0.31
15715917,0.8976864783\H,2.3577480528,1.8664346818,0.\H,2.2844637984,0.
3115715917,-0.8976864783\Se,-0.5724559604,-1.1949628384,0.\Se,0.008304
1076,0.9675351472,0.\\Version=EM64L-G16RevA.03\State=2-A"\HF=-4843.015
2108\S2=0.752798\S2-1=0.\S2A=0.750005\RMSD=3.429e-09\RMSF=1.747e-05\Di
pole=0.7935955,0.5199087,0.\Quadrupole=0.7565063,0.1227584,-0.8792646,
1.5380651,0.,0.\PG=CS [SG(C1H1Se2),X(H2)]\\@

1\1\GINC-ORC308\FOpt\UM08HX\6-311G(df,p)\C1H3Se2(2)\MEISTERP\24-Aug-20
17\0\\# opt freq=noraman m08hx/6-311g(df,p)\\Methyl Se Se optimization
and frequency\\0,2\C,1.9652124257,0.8442649459,0.\H,2.2841905763,0.31

```
12369661,0.8970473178\H,2.3551913887,1.8657130319,0.\H,2.2841905763,0.
3112369661,-0.8970473178\Se,-0.5699170489,-1.1892704007,0.\Se,0.011256
082,0.9637084907,0.\\Version=EM64L-G16RevA.03\State=2-A"\HF=-4843.0213
931\S2=0.752825\S2-1=0.\S2A=0.750006\RMSD=3.743e-09\RMSF=2.148e-05\Dip
ole=0.7823891,0.4927111,0.\Quadrupole=0.8065811,-0.0041144,-0.8024667,
1.4389299,0.,0.\PG=CS [SG(C1H1Se2),X(H2)]\\@
```

```
1\1\GINC-ORC308\FOpt\UM08HX\6-311+G(2df,p)\C1H3Se2(2)\MEISTERP\24-Aug-
2017\0\\# opt freq=noraman m08hx/6-311+g(2df,p)\\Methyl Se Se optimiza
tion and frequency\\0,2\C,1.9651863279,0.8436772231,0.\H,2.2832530679,
0.3115770823,0.8969018096\H,2.3542519681,1.864718858,0.\H,2.2832530679
,0.3115770823,-0.8969018096\Se,-0.5684947247,-1.1880374115,0.\Se,0.012
6742928,0.9633771659,0.\\Version=EM64L-G16RevA.03\State=2-A"\HF=-4843.
019413\S2=0.753599\S2-1=0.\S2A=0.750009\RMSD=9.997e-09\RMSF=2.331e-05\
Dipole=0.7561847,0.4773629,0.\Quadrupole=0.7743806,0.1549808,-0.929361
4,1.5421843,0.,0.\PG=CS [SG(C1H1Se2),X(H2)]\\@
```

```
1\1\GINC-ORC307\FOpt\UM08HX\6-311++G(3df,3pd)\C1H3Se2(2)\MEISTERP\24-A
ug-2017\0\\# opt freq=noraman m08hx/6-311++g(3df,3pd)\\Methyl Se Se op
timization and frequency\\0,2\C,1.966286994,0.844259818,0.\H,2.2801599
678,0.311897458,0.8951848859\H,2.3551953935,1.862573458,0.\H,2.2801599
678,0.311897458,-0.8951848859\Se,-0.5651025278,-1.1881492991,0.\Se,0.0
134242047,0.9644111071,0.\\Version=EM64L-G16RevA.03\State=2-A"\HF=-484
3.0287287\S2=0.753627\S2-1=0.\S2A=0.750009\RMSD=8.354e-09\RMSF=2.211e-
05\Dipole=0.7538183,0.4945949,0.\Quadrupole=0.7468272,0.1582735,-0.905
1007,1.525665,0.,0.\PG=CS [SG(C1H1Se2),X(H2)]\\@
```

CH_3-Se-Se• QCISD

```
1\1\GINC-ORC121\SP\UQCISD-FC\6-311+G(2df,p)\C1H3Se2(2)\MEISTERP\01-Sep
-2017\0\\# qcisd/6-311+g(2df,p)\\CH3-Se-Se qcisd single point\\0,2\C,0
,1.949323,0.895705,0.\H,0,2.286622,0.375854,0.894535\H,0,2.308298,1.92
5872,0.\H,0,2.286622,0.375854,-0.894535\Se,0,-0.546396,-1.193195,0.\Se
,0,0.,0.956377,0.\\Version=EM64L-G09RevE.01\State=2-A"\HF=-4839.239883
4\MP2=-4839.6916576\MP3=-4839.7294798\MP4D=-4839.7415801\MP4DQ=-4839.7
281752\PUHF=-4839.2435904\PMP2-0=-4839.6939341\PMP3-0=-4839.7306729\MP
4SDQ=-4839.7324152\QCISD=-4839.7341041\S2=0.768775\S2-1=0.754172\S2A=0
.750244\RMSD=5.673e-09\PG=CS [SG(C1H1Se2),X(H2)]\\@
```

CH_3-Se-S• B3LYP

```
1\1\GINC-ORC288\FOpt\UB3LYP\6-311+G(d,p)\C1H3S1Se1(2)\MEISTERP\24-Aug-
2017\0\\# opt freq=noraman b3lyp/6-311+g(d,p)\\Methyl Selenium Sulfide
\\0,2\C,1.8745620025,-0.0316636051,0.\H,2.0507686743,-0.6235818668,0.8
957252334\H,2.4982536041,0.8634199579,0.\H,2.0507686743,-0.6235818668,
-0.8957252334\Se,-0.0111114535,0.5748065397,0.\S,-1.1115055017,-1.2444
37159,0.\\Version=EM64L-G09RevE.01\State=2-A"\HF=-2839.6873852\S2=0.75
3178\S2-1=0.\S2A=0.750007\RMSD=5.962e-09\RMSF=6.084e-06\Dipole=1.01025
83,0.4743877,0.\Quadrupole=0.9662519,-0.7560568,-0.2101951,-0.1381907,
0.,0.\PG=CS [SG(C1H1S1Se1),X(H2)]\\@
```

```
1\1\GINC-ORC288\FOpt\UB3LYP\6-311G(df,p)\C1H3S1Se1(2)\MEISTERP\24-Aug-
2017\0\\# opt freq=noraman b3lyp/6-311g(df,p)\\Methyl Selenium Sulfide
\\0,2\C,1.87118175,-0.0311603165,0.\H,2.0471042422,-0.6241549011,0.894
9135378\H,2.4964161847,0.8623361547,0.\H,2.0471042422,-0.6241549011,-0
.8949135378\Se,-0.0093797437,0.5712094556,0.\S,-1.1006906753,-1.239113
```

4916,0.\\Version=EM64L-G09RevE.01\State=2-A"\HF=-2839.6929032\S2=0.753
641\S2-1=0.\S2A=0.75001\RMSD=2.833e-09\RMSF=1.028e-05\Dipole=1.0341953
,0.4964576,0.\Quadrupole=0.8805927,-0.7827767,-0.097816,-0.252321,0.,0
.\PG=CS [SG(C1H1S1Se1),X(H2)]\\@

1\1\GINC-ORC288\FOpt\UB3LYP\6-311+G(2df,p)\C1H3S1Se1(2)\MEISTERP\24-Au
g-2017\0\\# opt freq=noraman b3lyp/6-311+g(2df,p)\\Methyl Selenium Sul
fide\\0,2\C,1.8717039851,-0.0322939801,0.\H,2.0495042326,-0.6235331982
,0.8946519848\H,2.4930439808,0.8630266019,0.\H,2.0495042326,-0.6235331
982,-0.8946519848\Se,-0.0090414557,0.5657931235,0.\S,-1.1029789754,-1.
2344973489,0.\\Version=EM64L-G09RevE.01\State=2-A"\HF=-2839.6958661\S2
=0.754487\S2-1=0.\S2A=0.750014\RMSD=4.981e-09\RMSF=1.254e-05\Dipole=0.
9643293,0.4257916,0.\Quadrupole=0.9604552,-0.6584875,-0.3019677,-0.013
121,0.,0.\PG=CS [SG(C1H1S1Se1),X(H2)]\\@

1\1\GINC-ORC287\FOpt\UB3LYP\6-311++G(3df,3pd)\C1H3S1Se1(2)\MEISTERP\24
-Aug-2017\0\\# opt freq=noraman b3lyp/6-311++g(3df,3pd)\\Methyl Seleni
um Sulfide\\0,2\C,1.8719718248,-0.0323568316,0.\H,2.047571433,-0.62293
45407,0.8934485174\H,2.4924464358,0.8614203804,0.\H,2.047571433,-0.622
9345407,-0.8934485174\Se,-0.0083596261,0.5658809553,0.\S,-1.0994655005
,-1.2341134227,0.\\Version=EM64L-G09RevE.01\State=2-A"\HF=-2839.701616
\S2=0.754628\S2-1=0.\S2A=0.750015\RMSD=4.437e-09\RMSF=1.208e-05\Dipole
=0.9550366,0.4374971,0.\Quadrupole=0.9238535,-0.6547814,-0.2690721,-0.
0104159,0.,0.\PG=CS [SG(C1H1S1Se1),X(H2)]\\@

CH3-Se-S• B3PW91

1\1\GINC-ORC287\FOpt\UB3PW91\6-311+G(d,p)\C1H3S1Se1(2)\MEISTERP\24-Aug
-2017\0\\# opt freq=noraman b3pw91/6-311+g(d,p)\\Methyl Selenium Sulfi
de\\0,2\C,1.8667462889,-0.031914357,0.\H,2.0461375368,-0.6243916655,0.
895838501\H,2.4920952931,0.8629689277,0.\H,2.0461375368,-0.6243916655,
-0.895838501\Se,-0.0035510941,0.5676076512,0.\S,-1.0958295616,-1.23491
68909,0.\\Version=EM64L-G09RevE.01\State=2-A"\HF=-2839.6002026\S2=0.75
3177\S2-1=0.\S2A=0.750007\RMSD=7.987e-09\RMSF=1.414e-05\Dipole=1.02572
4,0.4682213,0.\Quadrupole=1.012044,-0.786096,-0.2259481,-0.1569693,0.,
0.\PG=CS [SG(C1H1S1Se1),X(H2)]\\@

1\1\GINC-ORC287\FOpt\UB3PW91\6-311G(df,p)\C1H3S1Se1(2)\MEISTERP\24-Aug
-2017\0\\# opt freq=noraman b3pw91/6-311g(df,p)\\Methyl Selenium Sulfi
de\\0,2\C,1.863502859,-0.0314173211,0.\H,2.0424214028,-0.625025227,0.8
950571779\H,2.4902863963,0.8619399096,0.\H,2.0424214028,-0.625025227,-
0.8950571779\Se,-0.0018474075,0.5642393983,0.\S,-1.0850486534,-1.22974
95328,0.\\Version=EM64L-G09RevE.01\State=2-A"\HF=-2839.6059537\S2=0.75
3737\S2-1=0.\S2A=0.75001\RMSD=8.461e-09\RMSF=1.805e-05\Dipole=1.046399
5,0.484436,0.\Quadrupole=0.9333421,-0.8161994,-0.1171427,-0.2637535,0.
,0.\PG=CS [SG(C1H1S1Se1),X(H2)]\\@

1\1\GINC-ORC286\FOpt\UB3PW91\6-311+G(2df,p)\C1H3S1Se1(2)\MEISTERP\24-A
ug-2017\0\\# opt freq=noraman b3pw91/6-311+g(2df,p)\\Methyl Selenium S
ulfide\\0,2\C,1.8642887928,-0.0324940398,0.\H,2.0445760631,-0.62438967
63,0.8950267381\H,2.4874500522,0.8627293409,0.\H,2.0445760631,-0.62438
96763,-0.8950267381\Se,-0.0014939696,0.559337032,0.\S,-1.0876610017,-1
.2258309806,0.\\Version=EM64L-G09RevE.01\State=2-A"\HF=-2839.6091673\S
2=0.754838\S2-1=0.\S2A=0.750017\RMSD=7.176e-09\RMSF=2.073e-05\Dipole=0
.9787454,0.4160544,0.\Quadrupole=1.0109799,-0.6755766,-0.3354033,-0.01
48327,0.,0.\PG=CS [SG(C1H1S1Se1),X(H2)]\\@

```
1\1\GINC-ORC286\FOpt\UB3PW91\6-311++G(3df,3pd)\C1H3S1Se1(2)\MEISTERP\2
 4-Aug-2017\0\\# opt freq=noraman b3pw91/6-311++g(3df,3pd)\\Methyl Sele
 nium Sulfide\\0,2\C,1.8644121051,-0.0323554746,0.\H,2.0423779539,-0.62
 39454471,0.8939343801\H,2.4872961395,0.8612181216,0.\H,2.0423779539,-0
 .6239454471,-0.8939343801\Se,-0.0009246048,0.5596283407,0.\S,-1.083803
 5477,-1.2256380934,0.\\Version=EM64L-G09RevE.01\State=2-A"\HF=-2839.61
 49213\S2=0.755021\S2-1=0.\S2A=0.750018\RMSD=2.525e-09\RMSF=2.156e-05\D
 ipole=0.9695173,0.4308312,0.\Quadrupole=0.9677575,-0.6717968,-0.295960
 7,-0.0132543,0.,0.\PG=CS [SG(C1H1S1Se1),X(H2)]\\@
```

CH_3-Se-S$^•$ wB97XD

```
1\1\GINC-ORC283\FOpt\UwB97XD\6-311+G(d,p)\C1H3S1Se1(2)\MEISTERP\24-Aug
 -2017\0\\# opt freq=noraman wb97xd/6-311+g(d,p)\\Methyl Selenium Sulfi
 de\\0,2\C,1.8598615401,-0.031201333,0.\H,2.0391923731,-0.6246650011,0.
 894679396\H,2.492240866,0.8580059147,0.\H,2.0391923731,-0.6246650011,-
 0.894679396\Se,-0.0014043484,0.571058639,0.\S,-1.0773468041,-1.2335712
 184,0.\\Version=EM64L-G09RevE.01\State=2-A"\HF=-2839.671441\S2=0.75440
 3\S2-1=0.\S2A=0.750012\RMSD=8.818e-09\RMSF=1.255e-05\Dipole=1.0341416,
 0.491349,0.\Quadrupole=1.0360691,-0.8243936,-0.2116755,-0.1800758,0.,0
 .\PG=CS [SG(C1H1S1Se1),X(H2)]\\@
```

```
1\1\GINC-ORC283\FOpt\UwB97XD\6-311G(df,p)\C1H3S1Se1(2)\MEISTERP\24-Aug
 -2017\0\\# opt freq=noraman wb97xd/6-311g(df,p)\\Methyl Selenium Sulfi
 de\\0,2\C,1.8572330822,-0.0307199515,0.\H,2.0361363324,-0.6251745499,0
 .8940001002\H,2.4906778102,0.8572348104,0.\H,2.0361363324,-0.625174549
 9,-0.8940001002\Se,-0.0002039807,0.5672746793,0.\S,-1.0682435766,-1.22
 84784385,0.\\Version=EM64L-G09RevE.01\State=2-A"\HF=-2839.6774762\S2=0
 .754943\S2-1=0.\S2A=0.750016\RMSD=5.657e-09\RMSF=1.723e-05\Dipole=1.05
 42426,0.5057909,0.\Quadrupole=0.9327808,-0.846205,-0.0865758,-0.291853
 ,0.,0.\PG=CS [SG(C1H1S1Se1),X(H2)]\\@
```

```
1\1\GINC-ORC282\FOpt\UwB97XD\6-311+G(2df,p)\C1H3S1Se1(2)\MEISTERP\24-A
 ug-2017\0\\# opt freq=noraman wb97xd/6-311+g(2df,p)\\Methyl Selenium S
 ulfide\\0,2\C,1.8577735059,-0.0316037519,0.\H,2.0386941177,-0.62441882
 09,0.8938420236\H,2.4879413865,0.8580130191,0.\H,2.0386941177,-0.62441
 88209,-0.8938420236\Se,0.0003080561,0.5618370761,0.\S,-1.0716751839,-1
 .2244467015,0.\\Version=EM64L-G09RevE.01\State=2-A"\HF=-2839.6809964\S
 2=0.756139\S2-1=0.\S2A=0.750023\RMSD=6.924e-09\RMSF=2.041e-05\Dipole=0
 .9917219,0.439434,0.\Quadrupole=1.0345295,-0.7074636,-0.3270659,-0.033
 3889,0.,0.\PG=CS [SG(C1H1S1Se1),X(H2)]\\@
```

```
1\1\GINC-ORC282\FOpt\UwB97XD\6-311++G(3df,3pd)\C1H3S1Se1(2)\MEISTERP\2
 4-Aug-2017\0\\# opt freq=noraman wb97xd/6-311++g(3df,3pd)\\Methyl Sele
 nium Sulfide\\0,2\C,1.8579912367,-0.0315522387,0.\H,2.0363017848,-0.62
 3770038,0.8926737894\H,2.4877932513,0.8561679397,0.\H,2.0363017848,-0.
 623770038,-0.8926737894\Se,0.0009237546,0.5622984718,0.\S,-1.067575812
 3,-1.2244120968,0.\\Version=EM64L-G09RevE.01\State=2-A"\HF=-2839.68703
 83\S2=0.756364\S2-1=0.\S2A=0.750025\RMSD=6.052e-09\RMSF=2.133e-05\Dipo
 le=0.9824933,0.4497112,0.\Quadrupole=1.0026238,-0.6928473,-0.3097765,-
 0.0170043,0.,0.\PG=CS [SG(C1H1S1Se1),X(H2)]\\@
```

CH_3-Se-S$^•$ M062X

```
1\1\GINC-ORC286\FOpt\UM062X\6-311+G(d,p)\C1H3S1Se1(2)\MEISTERP\24-Aug-
 2017\0\\# opt freq=noraman m062x/6-311+g(d,p)\\Methyl Selenium Sulfide
 \\0,2\C,1.8590962539,-0.0297311033,0.\H,2.0266955929,-0.6250403102,0.8
```

943937283\H,2.4980781088,0.8528028044,0.\H,2.0266955929,-0.6250403102,
-0.8943937283\Se,-0.0022715239,0.5804409798,0.\S,-1.0565580248,-1.2384
700605,0.\\Version=EM64L-G09RevE.01\State=2-A"\HF=-2839.6207675\S2=0.7
53887\S2-1=0.\S2A=0.750009\RMSD=4.341e-09\RMSF=6.369e-05\Dipole=1.0026
643,0.4423589,0.\Quadrupole=1.0736415,-0.8121015,-0.26154,-0.0842396,0
.,0.\PG=CS [SG(C1H1S1Se1),X(H2)]\\@

1\1\GINC-ORC285\FOpt\UM062X\6-311G(df,p)\C1H3S1Se1(2)\MEISTERP\24-Aug-
2017\0\\# opt freq=noraman m062x/6-311g(df,p)\\Methyl Selenium Sulfide
\\0,2\C,1.8564157345,-0.0291513233,0.\H,2.0245069604,-0.625544177,0.89
36519994\H,2.4964379616,0.8523153341,0.\H,2.0245069604,-0.625544177,-0
.8936519994\Se,-0.0009835661,0.576350985,0.\S,-1.0491480509,-1.2334646
417,0.\\Version=EM64L-G09RevE.01\State=2-A"\HF=-2839.6260456\S2=0.7539
69\S2-1=0.\S2A=0.75001\RMSD=6.766e-09\RMSF=6.248e-05\Dipole=1.0278641,
0.4584336,0.\Quadrupole=0.9650926,-0.8393497,-0.125743,-0.2107808,0.,0
.\PG=CS [SG(C1H1S1Se1),X(H2)]\\@

1\1\GINC-ORC285\FOpt\UM062X\6-311+G(2df,p)\C1H3S1Se1(2)\MEISTERP\24-Au
g-2017\0\\# opt freq=noraman m062x/6-311+g(2df,p)\\Methyl Selenium Sul
fide\\0,2\C,1.8580959534,-0.0304998106,0.\H,2.0272426767,-0.6248908086
,0.8937694977\H,2.4928151325,0.8539341503,0.\H,2.0272426767,-0.6248908
086,-0.8937694977\Se,0.0000311312,0.5705745563,0.\S,-1.0536915706,-1.2
292652788,0.\\Version=EM64L-G09RevE.01\State=2-A"\HF=-2839.6301305\S2=
0.755116\S2-1=0.\S2A=0.750015\RMSD=7.046e-09\RMSF=4.180e-05\Dipole=0.9
565713,0.3867859,0.\Quadrupole=1.062784,-0.6906461,-0.372138,0.0619133
,0.,0.\PG=CS [SG(C1H1S1Se1),X(H2)]\\@

1\1\GINC-ORC285\FOpt\UM062X\6-311++G(3df,3pd)\C1H3S1Se1(2)\MEISTERP\24
-Aug-2017\0\\# opt freq=noraman m062x/6-311++g(3df,3pd)\\Methyl Seleni
um Sulfide\\0,2\C,1.8578198794,-0.0299939232,0.\H,2.0242234362,-0.6240
778464,0.8922054635\H,2.4937799413,0.8511945608,0.\H,2.0242234362,-0.6
240778464,-0.8922054635\Se,0.0000261224,0.5716064549,0.\S,-1.048336815
5,-1.2296893998,0.\\Version=EM64L-G09RevE.01\State=2-A"\HF=-2839.63629
9\S2=0.755298\S2-1=0.\S2A=0.750016\RMSD=5.052e-09\RMSF=6.816e-05\Dipol
e=0.9469881,0.4063391,0.\Quadrupole=1.0277775,-0.6810793,-0.3466982,0.
0632578,0.,0.\PG=CS [SG(C1H1S1Se1),X(H2)]\\@

CH_3-Se-S$^{\bullet}$ M08HX

1\1\GINC-ORC284\FOpt\UM08HX\6-311+G(d,p)\C1H3S1Se1(2)\MEISTERP\24-Aug-
2017\0\\# opt freq=noraman m08hx/6-311+g(d,p)\\Methyl Selenium Sulfide
\\0,2\C,1.856416674,-0.0295998168,0.\H,2.0237412523,-0.6277127107,0.89
73871687\H,2.5006825853,0.8537740568,0.\H,2.0237412523,-0.6277127107,-
0.8973871687\Se,-0.0035432852,0.5884671444,0.\S,-1.0493024793,-1.24225
3963,0.\\Version=EM64L-G16RevA.03\State=2-A"\HF=-2839.6313316\S2=0.753
393\S2-1=0.\S2A=0.750008\RMSD=7.524e-09\RMSF=4.422e-05\Dipole=0.958585
2,0.395564,0.\Quadrupole=1.0776792,-0.7773184,-0.3003608,-0.0228108,0.
,0.\PG=CS [SG(C1H1S1Se1),X(H2)]\\@

1\1\GINC-ORC284\FOpt\UM08HX\6-311G(df,p)\C1H3S1Se1(2)\MEISTERP\24-Aug-
2017\0\\# opt freq=noraman m08hx/6-311g(df,p)\\Methyl Selenium Sulfide
\\0,2\C,1.8539108915,-0.0292305403,0.\H,2.0222578614,-0.6280983965,0.8
966564285\H,2.4985131959,0.8534813716,0.\H,2.0222578614,-0.6280983965,
-0.8966564285\Se,-0.0020006099,0.5840789023,0.\S,-1.0432031996,-1.2371
709406,0.\\Version=EM64L-G16RevA.03\State=2-A"\HF=-2839.6368281\S2=0.7
53789\S2-1=0.\S2A=0.75001\RMSD=6.992e-09\RMSF=3.518e-05\Dipole=0.97720
39,0.4027572,0.\Quadrupole=0.9859119,-0.79964,-0.1862719,-0.1328364,0.

,0.\PG=CS [SG(C1H1S1Se1),X(H2)]\\@

1\1\GINC-ORC284\FOpt\UM08HX\6-311+G(2df,p)\C1H3S1Se1(2)\MEISTERP\24-Au
g-2017\0\\# opt freq=noraman m08hx/6-311+g(2df,p)\\Methyl Selenium Sul
fide\\0,2\C,1.8546274096,-0.0299485657,0.\H,2.0236264995,-0.6272716023
,0.8965625317\H,2.496699851,0.8537849629,0.\H,2.0236264995,-0.62727160
23,-0.8965625317\Se,-0.0016051733,0.579727444,0.\S,-1.0452390865,-1.23
40586367,0.\\Version=EM64L-G16RevA.03\State=2-A"\HF=-2839.6409442\S2=0
.754567\S2-1=0.\S2A=0.750014\RMSD=7.636e-09\RMSF=3.867e-05\Dipole=0.90
79747,0.3449121,0.\Quadrupole=1.0523014,-0.6444607,-0.4078407,0.120067
2,0.,0.\PG=CS [SG(C1H1S1Se1),X(H2)]\\@

1\1\GINC-ORC283\FOpt\UM08HX\6-311+G(3df,3pd)\C1H3S1Se1(2)\MEISTERP\24-
Aug-2017\0\\# opt freq=noraman m08hx/6-311+g(3df,3pd)\\Methyl Selenium
 Sulfide\\0,2\C,1.8552814701,-0.0298870024,0.\H,2.0202551619,-0.626414
5755,0.894812759\H,2.4964866157,0.8512615361,0.\H,2.0202551619,-0.6264
145755,-0.894812759\Se,-0.0009219706,0.5804569292,0.\S,-1.0396204392,-
1.2340403119,0.\\Version=EM64L-G16RevA.03\State=2-A"\HF=-2839.6475203\
S2=0.754745\S2-1=0.\S2A=0.750015\RMSD=7.025e-09\RMSF=4.917e-05\Dipole=
0.9063794,0.3653145,0.\Quadrupole=1.0200027,-0.6440744,-0.3759283,0.11
50274,0.,0.\PG=CS [SG(C1H1S1Se1),X(H2)]\\@

CH_3-Se-S• QCISD

1\1\GINC-ORC8\SP\UQCISD-FC\6-311+G(2df,p)\C1H3S1Se1(2)\MEISTERP\01-Sep
-2017\0\\# qcisd/6-311+g(2df,p)\\CH3-Se-S radical qcisd single point\\
0,2\C,0,1.842119,-0.056033,0.\H,0,2.001523,-0.656092,0.894104\H,0,2.49
8681,0.815022,0.\H,0,2.001523,-0.656092,-0.894104\Se,0,0.,0.577142,0.\
S,0,-1.097152,-1.174343,0.\\Version=EM64L-G09RevE.01\State=2-A"\HF=-28
36.9678291\MP2=-2837.4396977\MP3=-2837.479386\MP4D=-2837.4922997\MP4DQ
=-2837.4779497\PUHF=-2836.97248\PMP2-0=-2837.4426063\PMP3-0=-2837.4808
962\MP4SDQ=-2837.4825948\QCISD=-2837.4843247\S2=0.773708\S2-1=0.755781
\S2A=0.750366\RMSD=8.117e-09\PG=CS [SG(C1H1S1Se1),X(H2)]\\@

CH_3-S-Se• B3LYP

1\1\GINC-ORC295\FOpt\UB3LYP\6-311+G(d,p)\C1H3S1Se1(2)\MEISTERP\24-Aug-
2017\0\\# opt freq=noraman b3lyp/6-311+g(d,p)\\Methyl Sulfur Selenide\
\0,2\C,-1.0126129242,-1.9659620761,0.\H,-1.5838302455,-1.7175149014,0.
8934075245\H,-0.7642950263,-3.0292650506,0.\H,-1.5838302455,-1.7175149
014,-0.8934075245\S,0.5809089915,-1.0564006991,0.\Se,0.05633245,1.0310
616287,0.\\Version=EM64L-G09RevE.01\State=2-A"\HF=-2839.6910246\S2=0.7
52626\S2-1=0.\S2A=0.750005\RMSD=9.152e-09\RMSF=1.538e-04\Dipole=-0.516
273,-0.7446817,0.\Quadrupole=-1.5448893,2.3620305,-0.8171412,1.2382295
,0.,0.\PG=CS [SG(C1H1S1Se1),X(H2)]\\@

1\1\GINC-ORC295\FOpt\UB3LYP\6-311G(df,p)\C1H3S1Se1(2)\MEISTERP\24-Aug-
2017\0\\# opt freq=noraman b3lyp/6-311g(df,p)\\Methyl Sulfur Selenide\
\0,2\C,-1.0122430698,-1.9650242304,0.\H,-1.5839750773,-1.7167979455,0.
8927587314\H,-0.7625593272,-3.0276289338,0.\H,-1.5839750773,-1.7167979
455,-0.8927587314\S,0.5781376454,-1.051629727,0.\Se,0.0572879063,1.022
2827825,0.\\Version=EM64L-G09RevE.01\State=2-A"\HF=-2839.6958905\S2=0.
753061\S2-1=0.\S2A=0.750007\RMSD=6.528e-09\RMSF=3.087e-05\Dipole=-0.52
80923,-0.7249561,0.\Quadrupole=-1.4409297,2.217892,-0.7769623,1.288092
6,0.,0.\PG=CS [SG(C1H1S1Se1),X(H2)]\\@

```
1\1\GINC-ORC294\FOpt\UB3LYP\6-311+G(2df,p)\C1H3S1Se1(2)\MEISTERP\24-Au
 g-2017\0\\# opt freq=noraman b3lyp/6-311+g(2df,p)\\Methyl Sulfur Selen
 ide\\0,2\C,-1.0118041677,-1.9635300729,0.\H,-1.5821384268,-1.715451775
 8,0.8928635219\H,-0.7600457033,-3.0252702618,0.\H,-1.5821384268,-1.715
 4517758,-0.8928635219\S,0.5711682453,-1.0502581051,0.\Se,0.0576314795,
 1.0143659917,0.\\Version=EM64L-G09RevE.01\State=2-A"\HF=-2839.7012679\
 S2=0.753837\S2-1=0.\S2A=0.750011\RMSD=6.498e-09\RMSF=2.980e-05\Dipole=
 -0.4832783,-0.7548714,0.\Quadrupole=-1.4260918,2.3059804,-0.8798887,1.
 1945757,0.,0.\PG=CS [SG(C1H1S1Se1),X(H2)]\\@
```

```
1\1\GINC-ORC294\FOpt\UB3LYP\6-311++G(3df,3pd)\C1H3S1Se1(2)\MEISTERP\24
 -Aug-2017\0\\# opt freq=noraman b3lyp/6-311++g(3df,3pd)\\Methyl Sulfur
  Selenide\\0,2\C,-1.0116176226,-1.9627614044,0.\H,-1.5817671504,-1.716
 5999842,0.891545326\H,-0.7585469209,-3.0224708488,0.\H,-1.5817671504,-
 1.7165999842,-0.891545326\S,0.5668962814,-1.0504637416,0.\Se,0.0594755
 631,1.0132999632,0.\\Version=EM64L-G09RevE.01\State=2-A"\HF=-2839.7073
 469\S2=0.754017\S2-1=0.\S2A=0.750012\RMSD=6.581e-09\RMSF=2.376e-05\Dip
 ole=-0.4809296,-0.7423183,0.\Quadrupole=-1.406269,2.272415,-0.8661461,
 1.2373081,0.,0.\PG=CS [SG(C1H1S1Se1),X(H2)]\\@
```

CH_3-S-Se$^{\bullet}$ B3PW91

```
1\1\GINC-ORC294\FOpt\UB3PW91\6-311+G(d,p)\C1H3S1Se1(2)\MEISTERP\24-Aug
 -2017\0\\# opt freq=noraman b3pw91/6-311+g(d,p)\\Methyl Sulfur Selenid
 e\\0,2\C,-1.0088707726,-1.9604217745,0.\H,-1.5816928857,-1.7133433312,
 0.8936605245\H,-0.7607211596,-3.0245097187,0.\H,-1.5816928857,-1.71334
 33312,-0.8936605245\S,0.5736057057,-1.0564737923,0.\Se,0.0520449983,1.
 0124959482,0.\\Version=EM64L-G09RevE.01\State=2-A"\HF=-2839.6037854\S2
 =0.752688\S2-1=0.\S2A=0.750006\RMSD=4.981e-09\RMSF=1.715e-05\Dipole=-0
 .5265378,-0.7488073,0.\Quadrupole=-1.5229183,2.3475869,-0.8246687,1.26
 38824,0.,0.\PG=CS [SG(C1H1S1Se1),X(H2)]\\@
```

```
1\1\GINC-ORC293\FOpt\UB3PW91\6-311G(df,p)\C1H3S1Se1(2)\MEISTERP\24-Aug
 -2017\0\\# opt freq=noraman b3pw91/6-311g(df,p)\\Methyl Sulfur Selenid
 e\\0,2\C,-1.0079714895,-1.9592573121,0.\H,-1.5817577897,-1.7128681746,
 0.8930077274\H,-0.75949322,-3.0229318607,0.\H,-1.5817577897,-1.7128681
 746,-0.8930077274\S,0.570468766,-1.051644028,0.\Se,0.0531845234,1.0039
 735505,0.\\Version=EM64L-G09RevE.01\State=2-A"\HF=-2839.6089271\S2=0.7
 53247\S2-1=0.\S2A=0.750008\RMSD=7.059e-09\RMSF=1.896e-05\Dipole=-0.535
 8417,-0.7396991,0.\Quadrupole=-1.4241758,2.2006194,-0.7764436,1.312929
 6,0.,0.\PG=CS [SG(C1H1S1Se1),X(H2)]\\@
```

```
1\1\GINC-ORC293\FOpt\UB3PW91\6-311+G(2df,p)\C1H3S1Se1(2)\MEISTERP\24-A
 ug-2017\0\\# opt freq=noraman b3pw91/6-311+g(2df,p)\\Methyl Sulfur Sel
 enide\\0,2\C,-1.0078794239,-1.958164601,0.\H,-1.5795928509,-1.71108570
 33,0.8934554101\H,-0.7571032062,-3.021067695,0.\H,-1.5795928509,-1.711
 0857033,-0.8934554101\S,0.5637131541,-1.0506281932,0.\Se,0.0531281783,
 0.9964358965,0.\\Version=EM64L-G09RevE.01\State=2-A"\HF=-2839.6148668\
 S2=0.754225\S2-1=0.\S2A=0.750013\RMSD=5.913e-09\RMSF=2.254e-05\Dipole=
 -0.4937418,-0.7592115,0.\Quadrupole=-1.4093201,2.3084756,-0.8991556,1.
 2190591,0.,0.\PG=CS [SG(C1H1S1Se1),X(H2)]\\@
```

```
1\1\GINC-ORC293\FOpt\UB3PW91\6-311++G(3df,3pd)\C1H3S1Se1(2)\MEISTERP\2
 4-Aug-2017\0\\# opt freq=noraman b3pw91/6-311++g(3df,3pd)\\Methyl Sulf
 ur Selenide\\0,2\C,-1.0077990189,-1.9575335912,0.\H,-1.5793745027,-1.7
 122313837,0.8923412307\H,-0.7555621353,-3.0186120958,0.\H,-1.579374502
 7,-1.7122313837,-0.8923412307\S,0.5596570752,-1.0507051895,0.\Se,0.055
```

1260844,0.9957176442,0.\\Version=EM64L-G09RevE.01\State=2-A"\HF=-2839.
6207164\S2=0.754457\S2-1=0.\S2A=0.750015\RMSD=5.894e-09\RMSF=2.157e-05
\Dipole=-0.4926005,-0.745248,0.\Quadrupole=-1.3933888,2.281983,-0.8885
941,1.2687384,0.,0.\PG=CS [SG(C1H1S1Se1),X(H2)]\\@

CH_3-S-Se• wB97XD

1\1\GINC-ORC290\FOpt\UwB97XD\6-311+G(d,p)\C1H3S1Se1(2)\MEISTERP\24-Aug
-2017\0\\# opt freq=noraman wb97xd/6-311+g(d,p)\\Methyl Sulfur Selenid
e\\0,2\C,-1.0060175767,-1.9580288225,0.\H,-1.5777578666,-1.7090099104,
0.8932030327\H,-0.7652909933,-3.0227035757,0.\H,-1.5777578666,-1.70900
99104,-0.8932030327\S,0.5770752066,-1.0592725106,0.\Se,0.0424220964,1.
0024287297,0.\\Version=EM64L-G09RevE.01\State=2-A"\HF=-2839.6759541\S2
=0.753683\S2-1=0.\S2A=0.75001\RMSD=5.188e-09\RMSF=9.670e-05\Dipole=-0.
5351767,-0.704463,0.\Quadrupole=-1.5655348,2.3561849,-0.7906501,1.2782
082,0.,0.\PG=CS [SG(C1H1S1Se1),X(H2)]\\@

1\1\GINC-ORC289\FOpt\UwB97XD\6-311G(df,p)\C1H3S1Se1(2)\MEISTERP\24-Aug
-2017\0\\# opt freq=noraman wb97xd/6-311g(df,p)\\Methyl Sulfur Selenid
e\\0,2\C,-1.005623647,-1.9572388678,0.\H,-1.5781057009,-1.7087565673,0
.8925801409\H,-0.7633402329,-3.0212612498,0.\H,-1.5781057009,-1.708756
5673,-0.8925801409\S,0.5734343598,-1.0544423488,0.\Se,0.0444139222,0.9
948596014,0.\\Version=EM64L-G09RevE.01\State=2-A"\HF=-2839.6811808\S2=
0.754168\S2-1=0.\S2A=0.750013\RMSD=4.986e-09\RMSF=1.949e-05\Dipole=-0.
5449286,-0.6979958,0.\Quadrupole=-1.4663218,2.2015229,-0.7352011,1.321
3341,0.,0.\PG=CS [SG(C1H1S1Se1),X(H2)]\\@

1\1\GINC-ORC289\FOpt\UwB97XD\6-311+G(2df,p)\C1H3S1Se1(2)\MEISTERP\24-A
ug-2017\0\\# opt freq=noraman wb97xd/6-311+g(2df,p)\\Methyl Sulfur Sel
enide\\0,2\C,-1.005637058,-1.95612754,0.\H,-1.5761653499,-1.7074115192
,0.8929554213\H,-0.7610553908,-3.0193255542,0.\H,-1.5761653499,-1.7074
115192,-0.8929554213\S,0.5668572997,-1.0530606174,0.\Se,0.0448388488,0
.98774075,0.\\Version=EM64L-G09RevE.01\State=2-A"\HF=-2839.6872694\S2=
0.755322\S2-1=0.\S2A=0.750019\RMSD=4.047e-09\RMSF=2.104e-05\Dipole=-0.
5003312,-0.7208603,0.\Quadrupole=-1.4601844,2.340945,-0.8807606,1.2242
138,0.,0.\PG=CS [SG(C1H1S1Se1),X(H2)]\\@

1\1\GINC-ORC289\FOpt\UwB97XD\6-311++G(3df,3pd)\C1H3S1Se1(2)\MEISTERP\2
4-Aug-2017\0\\# opt freq=noraman wb97xd/6-311++g(3df,3pd)\\Methyl Sulf
ur Selenide\\0,2\C,-1.0055519522,-1.9555399543,0.\H,-1.5758550888,-1.7
087516423,0.8916603709\H,-0.7596328919,-3.0167327457,0.\H,-1.575855088
8,-1.7087516423,-0.8916603709\S,0.5625690192,-1.0532069311,0.\Se,0.046
9990022,0.9873869155,0.\\Version=EM64L-G09RevE.01\State=2-A"\HF=-2839.
6933213\S2=0.755528\S2-1=0.\S2A=0.75002\RMSD=3.016e-09\RMSF=2.195e-05\
Dipole=-0.4992009,-0.7107538,0.\Quadrupole=-1.4466568,2.3295242,-0.882
8674,1.2759228,0.,0.\PG=CS [SG(C1H1S1Se1),X(H2)]\\@

CH_3-S-Se• M062X

1\1\GINC-ORC292\FOpt\UM062X\6-311+G(d,p)\C1H3S1Se1(2)\MEISTERP\24-Aug-
2017\0\\# opt freq=noraman m062x/6-311+g(d,p)\\Methyl Sulfur Selenide\
\0,2\C,-1.0042303549,-1.9557569796,0.\H,-1.5717136391,-1.700560064,0.8
926307105\H,-0.7706977366,-3.0211535513,0.\H,-1.5717136391,-1.70056006
4,-0.8926307105\S,0.5862336753,-1.0691792357,0.\Se,0.0247946943,0.9916
138946,0.\\Version=EM64L-G09RevE.01\State=2-A"\HF=-2839.6270133\S2=0.7
54913\S2-1=0.\S2A=0.750017\RMSD=3.613e-09\RMSF=1.144e-04\Dipole=-0.527
0393,-0.6463049,0.\Quadrupole=-1.5541354,2.2402072,-0.6860718,1.242856

,0.,0.\PG=CS [SG(C1H1S1Se1),X(H2)]\\@

1\1\GINC-ORC292\FOpt\UM062X\6-311+G(df,p)\C1H3S1Se1(2)\MEISTERP\24-Aug-2017\0\\# opt freq=noraman m062x/6-311+g(df,p)\\Methyl Sulfur Selenide\\0,2\C,-1.0038898417,-1.9548391776,0.\H,-1.5734958868,-1.7016357104,0.8920953513\H,-0.7673834952,-3.0195271086,0.\H,-1.5734958868,-1.7016357104,-0.8920953513\S,0.5814574746,-1.0630178803,0.\Se,0.0294806359,0.9850595872,0.\\Version=EM64L-G09RevE.01\State=2-A"\HF=-2839.63434\S2=0.755133\S2-1=0.\S2A=0.750019\RMSD=5.897e-09\RMSF=2.372e-05\Dipole=-0.5206943,-0.6515671,0.\Quadrupole=-1.4563734,2.0623336,-0.6059602,1.3066342,0.,0.\PG=CS [SG(C1H1S1Se1),X(H2)]\\@

1\1\GINC-ORC292\FOpt\UM062X\6-311+G(2df,p)\C1H3S1Se1(2)\MEISTERP\24-Aug-2017\0\\# opt freq=noraman m062x/6-311+g(2df,p)\\Methyl Sulfur Selenide\\0,2\C,-1.0040743104,-1.9541200517,0.\H,-1.5703188574,-1.6990020166,0.8924477792\H,-0.7662372254,-3.0179731552,0.\H,-1.5703188574,-1.6990020166,-0.8924477792\S,0.5759772078,-1.0626659189,0.\Se,0.0276450429,0.9771671589,0.\\Version=EM64L-G09RevE.01\State=2-A"\HF=-2839.6385045\S2=0.756178\S2-1=0.\S2A=0.750025\RMSD=8.011e-09\RMSF=2.403e-05\Dipole=-0.4875838,-0.6621293,0.\Quadrupole=-1.442525,2.2199745,-0.7774495,1.1815507,0.,0.\PG=CS [SG(C1H1S1Se1),X(H2)]\\@

1\1\GINC-ORC291\FOpt\UM062X\6-311++G(3df,3pd)\C1H3S1Se1(2)\MEISTERP\24-Aug-2017\0\\# opt freq=noraman m062x/6-311++g(3df,3pd)\\Methyl Sulfur Selenide\\0,2\C,-1.0043811023,-1.9539539363,0.\H,-1.5699604079,-1.700354038,0.8910761667\H,-0.7650712384,-3.0155823553,0.\H,-1.5699604079,-1.700354038,-0.8910761667\S,0.5722141292,-1.0624880314,0.\Se,0.0298320272,0.9771363989,0.\\Version=EM64L-G09RevE.01\State=2-A"\HF=-2839.6446713\S2=0.756355\S2-1=0.\S2A=0.750026\RMSD=6.253e-09\RMSF=2.082e-05\Dipole=-0.4850293,-0.6517704,0.\Quadrupole=-1.421449,2.2095016,-0.7880526,1.223145,0.,0.\PG=CS [SG(C1H1S1Se1),X(H2)]\\@

CH_3-S-Se• M08HX

1\1\GINC-ORC291\FOpt\UM08HX\6-311+G(d,p)\C1H3S1Se1(2)\MEISTERP\24-Aug-2017\0\\# opt freq=noraman m08hx/6-311+g(d,p)\\Methyl Sulfur Selenide\\0,2\C,-1.001840758,-1.9562298208,0.\H,-1.5725530312,-1.6972257426,0.8948720223\H,-0.7693152034,-3.0259309048,0.\H,-1.5725530312,-1.6972257426,-0.8948720223\S,0.5828010541,-1.0622137778,0.\Se,0.0261339697,0.9832299886,0.\\Version=EM64L-G16RevA.03\State=2-A"\HF=-2839.6362198\S2=0.753437\S2-1=0.\S2A=0.750008\RMSD=3.465e-09\RMSF=2.855e-05\Dipole=-0.496078,-0.7840526,0.\Quadrupole=-1.4988113,2.2466915,-0.7478802,1.1774966,0.,0.\PG=CS [SG(C1H1S1Se1),X(H2)]\\@

1\1\GINC-ORC291\FOpt\UM08HX\6-311G(df,p)\C1H3S1Se1(2)\MEISTERP\24-Aug-2017\0\\# opt freq=noraman m08hx/6-311g(df,p)\\Methyl Sulfur Selenide\\0,2\C,-1.000620347,-1.9552813708,0.\H,-1.5726641722,-1.6969741432,0.8941506045\H,-0.7685447252,-3.0248057887,0.\H,-1.5726641722,-1.6969741432,-0.8941506045\S,0.5796623978,-1.0577301016,0.\Se,0.0275040188,0.9761695476,0.\\Version=EM64L-G16RevA.03\State=2-A"\HF=-2839.6420315\S2=0.753767\S2-1=0.\S2A=0.75001\RMSD=4.298e-09\RMSF=2.601e-05\Dipole=-0.5125206,-0.7673959,0.\Quadrupole=-1.3793144,2.0890804,-0.7097659,1.2420065,0.,0.\PG=CS [SG(C1H1S1Se1),X(H2)]\\@

1\1\GINC-ORC290\FOpt\UM08HX\6-311+G(2df,p)\C1H3S1Se1(2)\MEISTERP\24-Aug-2017\0\\# opt freq=noraman m08hx/6-311+g(2df,p)\\Methyl Sulfur Selenide\\0,2\C,-1.0009332842,-1.9545271456,0.\H,-1.5708688163,-1.695970575

7,0.8945121837\H,-0.765642495,-3.0229078317,0.\H,-1.5708688163,-1.6959
705757,-0.8945121837\S,0.5720536827,-1.0563107811,0.\Se,0.0289327293,0
.97009091,0.\\Version=EM64L-G16RevA.03\State=2-A"\HF=-2839.6486597\S2=
0.754792\S2-1=0.\S2A=0.750015\RMSD=2.232e-09\RMSF=2.429e-05\Dipole=-0.
4616884,-0.774599,0.\Quadrupole=-1.3895101,2.2409164,-0.8514063,1.1343
001,0.,0.\PG=CS [SG(C1H1S1Se1),X(H2)]\\@

1\1\GINC-ORC290\FOpt\UM08HX\6-311++G(3df,3pd)\C1H3S1Se1(2)\MEISTERP\24
-Aug-2017\0\\# opt freq=noraman m08hx/6-311++g(3df,3pd)\\Methyl Sulfur
 Selenide\\0,2\C,-1.0015872186,-1.9546944716,0.\H,-1.5695120336,-1.696
4282555,0.8929547623\H,-0.7650252792,-3.0202172609,0.\H,-1.5695120336,
-1.6964282555,-0.8929547623\S,0.5687084104,-1.056708252,0.\Se,0.029601
1546,0.9688804956,0.\\Version=EM64L-G16RevA.03\State=2-A"\HF=-2839.655
5539\S2=0.75505\S2-1=0.\S2A=0.750016\RMSD=2.176e-09\RMSF=2.507e-05\Dip
ole=-0.4644406,-0.7700724,0.\Quadrupole=-1.3762765,2.2444743,-0.868197
8,1.2050079,0.,0.\PG=CS [SG(C1H1S1Se1),X(H2)]\\@

CH_3-S-Se$^{\bullet}$ QCISD

1\1\GINC-ORC27\SP\UQCISD-FC\6-311+G(2df,p)\C1H3S1Se1(2)\MEISTERP\01-Se
p-2017\0\\# qcisd/6-311+g(2df,p)\\Methyl Sulfur Selenide\\0,2\C,0,-0.9
58151,-1.945731,0.\H,0,-1.532909,-1.706457,0.893212\H,0,-0.692444,-3.0
04451,0.\H,0,-1.532909,-1.706457,-0.893212\S,0,0.594198,-1.00758,0.\Se
,0,0.,1.006266,0.\\Version=EM64L-G09RevE.01\State=2-A"\HF=-2836.976086
6\MP2=-2837.4465997\MP3=-2837.485819\MP4D=-2837.49862\MP4DQ=-2837.4845
1\PUHF=-2836.9799181\PMP2-0=-2837.4489489\PMP3-0=-2837.4870292\MP4SDQ=
-2837.4887558\QCISD=-2837.4900477\S2=0.768687\S2-1=0.754116\S2A=0.7502
42\RMSD=5.903e-09\PG=CS [SG(C1H1S1Se1),X(H2)]\\@

CH_2=CH-Se$^{\bullet}$ B3LYP

1\1\GINC-GRA581\FOpt\UB3LYP\6-311+G(d,p)\C2H3Se1(2)\MEISTERP\05-Nov-20
18\0\\# opt freq=noraman ub3lyp/6-311+g(d,p)\\Methyl Selenyl Radical f
rom methyl thiyl radical for 2A' symmetry\\0,2\C,0.0076960008,0.,-0.00
13949168\H,0.0019652713,0.,1.0866085017\C,1.1503846631,0.,-0.686129578
9\H,2.0978635378,0.,-0.1590848855\H,1.1880524645,0.,-1.7691986675\Se,-
1.7566525644,0.,-0.7128095378\\Version=EM64L-G09RevE.01\State=2-A'\HF=
-2479.5311749\S2=0.752465\S2-1=0.\S2A=0.750003\RMSD=4.196e-09\RMSF=1.2
09e-05\Dipole=0.2560867,0.,0.1574044\Quadrupole=1.1893021,-3.1913594,2
.0020573,0.,0.0770091,0.\PG=CS [SG(C2H3Se1)]\\@

1\1\GINC-GRA581\FOpt\UB3LYP\6-311G(df,p)\C2H3Se1(2)\MEISTERP\05-Nov-20
18\0\\# opt freq=noraman ub3lyp/6-311g(df,p)\\Methyl Selenyl Radical f
rom methyl thiyl radical for 2A' symmetry\\0,2\C,-0.0255442246,0.,0.05
5051816\H,0.0057081251,0.,1.1403255365\C,1.1210227438,0.,-0.6506381204
\H,2.0859684627,0.,-0.1518511601\H,1.1176338616,0.,-1.7344039856\Se,-1
.713018066,0.,-0.744850661\\Version=EM64L-G09RevE.01\State=2-A"\HF=-24
79.5435598\S2=0.766763\S2-1=0.\S2A=0.750076\RMSD=7.978e-09\RMSF=1.240e
-04\Dipole=0.7720794,0.,0.1684817\Quadrupole=1.8527919,-1.306566,-0.54
62258,0.,0.8289612,0.\PG=CS [SG(C2H3Se1)]\\@

1\1\GINC-GRA581\FOpt\UB3LYP\6-311+G(2df,p)\C2H3Se1(2)\MEISTERP\05-Nov-
2018\0\\# opt freq=noraman ub3lyp/6-311+g(2df,p)\\Methyl Selenyl Radic
al from methyl thiyl radical for 2A' symmetry\\0,2\C,0.0076109468,0.,-
0.0041363867\H,0.003862618,0.,1.0830851197\C,1.1476494792,0.,-0.684856
7339\H,2.0945385003,0.,-0.1585280274\H,1.1863404346,0.,-1.767093924\Se
,-1.7501326393,0.,-0.7114040684\\Version=EM64L-G09RevE.01\State=2-A'\H

F=-2479.5357863\S2=0.752933\S2-1=0.\S2A=0.750005\RMSD=4.608e-09\RMSF=2
.046e-05\Dipole=0.257353,0.,0.1532326\Quadrupole=1.0952867,-3.0315793,
1.9362926,0.,0.0818103,0.\PG=CS [SG(C2H3Se1)]\\@

1\1\GINC-GRA581\FOpt\UB3LYP\6-311++G(3df,3pd)\C2H3Se1(2)\MEISTERP\05-N
ov-2018\0\\# opt freq=noraman ub3lyp/6-311++g(3df,3pd)\\Methyl Selenyl
Radical from methyl thiyl radical for 2A' symmetry\\0,2\C,0.007988304
8,0.,-0.0051693169\H,0.0049694264,0.,1.080479009\C,1.1483586079,0.,-0.
6856660622\H,2.0930071799,0.,-0.1587826258\H,1.1874587001,0.,-1.766185
5773\Se,-1.7501222953,0.,-0.7106522291\\Version=EM64L-G09RevE.01\State
=2-A'\HF=-2479.5414373\S2=0.75301\S2-1=0.\S2A=0.750005\RMSD=6.731e-09\
RMSF=2.461e-05\Dipole=0.2513109,0.,0.1436522\Quadrupole=1.0594884,-2.9
483498,1.8888615,0.,0.0434211,0.\PG=CS [SG(C2H3Se1)]\\@

CH_2=CH-Se$^{\bullet}$ B3PW91

1\1\GINC-GRA581\FOpt\UB3PW91\6-311+G(d,p)\C2H3Se1(2)\MEISTERP\05-Nov-2
018\0\\# opt freq=noraman ub3pw91/6-311+g(d,p)\\Methyl Selenyl Radical
from methyl thiyl radical for 2A' symmetry\\0,2\C,0.0053918193,0.,-0.
0036350892\H,0.0019790214,0.,1.0854544972\C,1.148361999,0.,-0.68706074
84\H,2.0950784236,0.,-0.1574506585\H,1.1865349592,0.,-1.7708895182\Se,
-1.7466181183,0.,-0.7107885544\\Version=EM64L-G09RevE.01\State=2-A'\HF
=-2479.4770354\S2=0.753033\S2-1=0.\S2A=0.750005\RMSD=2.476e-09\RMSF=4.
802e-05\Dipole=0.2585086,0.,0.1597497\Quadrupole=1.2117942,-3.2124747,
2.0006805,0.,0.0881758,0.\PG=CS [SG(C2H3Se1)]\\@

1\1\GINC-GRA581\FOpt\UB3PW91\6-311G(df,p)\C2H3Se1(2)\MEISTERP\05-Nov-2
018\0\\# opt freq=noraman ub3pw91/6-311g(df,p)\\Methyl Selenyl Radical
from methyl thiyl radical for 2A' symmetry\\0,2\C,-0.0279878864,0.,0.
0557987034\H,0.0034345214,0.,1.1421351143\C,1.1191209651,0.,-0.6502139
81\H,2.0849122175,0.,-0.1510618124\H,1.1134086187,0.,-1.7349191304\Se,
-1.7039090276,0.,-0.7445464845\\Version=EM64L-G09RevE.01\State=2-A"\HF
=-2479.4891386\S2=0.770946\S2-1=0.\S2A=0.750117\RMSD=3.218e-09\RMSF=1.
406e-04\Dipole=0.7774519,0.,0.1656208\Quadrupole=1.886458,-1.3791632,-
0.5072948,0.,0.8247171,0.\PG=CS [SG(C2H3Se1)]\\@

1\1\GINC-GRA581\FOpt\UB3PW91\6-311+G(2df,p)\C2H3Se1(2)\MEISTERP\05-Nov
-2018\0\\# opt freq=noraman ub3pw91/6-311+g(2df,p)\\Methyl Selenyl Rad
ical from methyl thiyl radical for 2A' symmetry\\0,2\C,0.005669125,0.,
-0.0062787453\H,0.0029899007,0.,1.0821978312\C,1.1460181257,0.,-0.6860
98416\H,2.0923660434,0.,-0.1569938597\H,1.1847172107,0.,-1.7692591312\
Se,-1.7401853015,0.,-0.7093371222\\Version=EM64L-G09RevE.01\State=2-A'
\HF=-2479.481568\S2=0.753721\S2-1=0.\S2A=0.750008\RMSD=8.199e-09\RMSF=
3.155e-05\Dipole=0.2599779,0.,0.1553334\Quadrupole=1.1150917,-3.054184
1,1.9390924,0.,0.0909858,0.\PG=CS [SG(C2H3Se1)]\\@

1\1\GINC-GRA581\FOpt\UB3PW91\6-311++G(3df,3pd)\C2H3Se1(2)\MEISTERP\05-
Nov-2018\0\\# opt freq=noraman ub3pw91/6-311++g(3df,3pd)\\Methyl Selen
yl Radical from methyl thiyl radical for 2A' symmetry\\0,2\C,0.0060513
76,0.,-0.0073833848\H,0.0040026761,0.,1.079802236\C,1.1467047069,0.,-0
.68701786\H,2.0909944085,0.,-0.1571227702\H,1.1859409043,0.,-1.7687138
278\Se,-1.740209802,0.,-0.7085673085\\Version=EM64L-G09RevE.01\State=2
-A'\HF=-2479.4870437\S2=0.753829\S2-1=0.\S2A=0.750009\RMSD=3.512e-09\R
MSF=3.711e-05\Dipole=0.2541582,0.,0.1459573\Quadrupole=1.0845206,-2.98
13116,1.896791,0.,0.0520965,0.\PG=CS [SG(C2H3Se1)]\\@

CH_2=CH-Se• wB97XD

```
1\1\GINC-GRA581\FOpt\UwB97XD\6-311+G(d,p)\C2H3Se1(2)\MEISTERP\05-Nov-2
 018\0\\# opt freq=noraman uwb97xd/6-311+g(d,p)\\Methyl Selenyl Radical
  from methyl thiyl radical for 2A' symmetry\\0,2\C,0.0052131949,0.,0.0
 007083618\H,0.0054656292,0.,1.0890543508\C,1.1446307105,0.,-0.68276540
 87\H,2.0914910498,0.,-0.1549470106\H,1.1790648014,0.,-1.7664599496\Se,
 -1.7442753597,0.,-0.7145379982\\Version=EM64L-G09RevE.01\State=2-A'\HF
 =-2479.5209079\S2=0.752744\S2-1=0.\S2A=0.750005\RMSD=5.105e-09\RMSF=8.
 811e-05\Dipole=0.282972,0.,0.1649718\Quadrupole=1.2778205,-3.2780296,2
 .0002091,0.,0.0839307,0.\PG=CS [SG(C2H3Se1)]\\@
```

```
1\1\GINC-GRA581\FOpt\UwB97XD\6-311G(df,p)\C2H3Se1(2)\MEISTERP\05-Nov-2
 018\0\\# opt freq=noraman uwb97xd/6-311g(df,p)\\Methyl Selenyl Radical
  from methyl thiyl radical for 2A' symmetry\\0,2\C,0.00548301,0.,-0.00
 0954411\H,0.0058147242,0.,1.0871534123\C,1.1426634583,0.,-0.6818069184
 \H,2.0898284422,0.,-0.1550381667\H,1.1779116025,0.,-1.7652074616\Se,-1
 .7398156257,0.,-0.7135973021\\Version=EM64L-G09RevE.01\State=2-A'\HF=-
 2479.5250536\S2=0.753082\S2-1=0.\S2A=0.750006\RMSD=6.339e-09\RMSF=7.43
 9e-05\Dipole=0.2823778,0.,0.162891\Quadrupole=1.2050257,-3.0594583,1.8
 544325,0.,0.1014264,0.\PG=CS [SG(C2H3Se1)]\\@
```

```
1\1\GINC-GRA581\FOpt\UwB97XD\6-311+G(2df,p)\C2H3Se1(2)\MEISTERP\05-Nov
 -2018\0\\# opt freq=noraman uwb97xd/6-311+g(2df,p)\\Methyl Selenyl Rad
 ical from methyl thiyl radical for 2A' symmetry\\0,2\C,0.005177967,0.,
 -0.0012297267\H,0.0071211337,0.,1.0863966397\C,1.1419059466,0.,-0.6814
 056296\H,2.0883166866,0.,-0.154217019\H,1.1767120374,0.,-1.764359948\S
 e,-1.7379602396,0.,-0.7136146185\\Version=EM64L-G09RevE.01\State=2-A'\
 HF=-2479.5253493\S2=0.753153\S2-1=0.\S2A=0.750006\RMSD=6.773e-09\RMSF=
 6.414e-05\Dipole=0.281278,0.,0.1577217\Quadrupole=1.1935496,-3.1229267
 ,1.9293771,0.,0.0885503,0.\PG=CS [SG(C2H3Se1)]\\@
```

```
1\1\GINC-GRA581\FOpt\UwB97XD\6-311++G(3df,3pd)\C2H3Se1(2)\MEISTERP\05-
 Nov-2018\0\\# opt freq=noraman uwb97xd/6-311++g(3df,3pd)\\Methyl Selen
 yl Radical from methyl thiyl radical for 2A' symmetry\\0,2\C,0.0055643
 538,0.,-0.0019854389\H,0.008251946,0.,1.0840659048\C,1.142512526,0.,-0
 .6819967651\H,2.0867402076,0.,-0.1543840336\H,1.1773515544,0.,-1.76323
 31563\Se,-1.7378446132,0.,-0.7130770732\\Version=EM64L-G09RevE.01\Stat
 e=2-A'\HF=-2479.5311291\S2=0.753219\S2-1=0.\S2A=0.750007\RMSD=3.499e-0
 9\RMSF=5.779e-05\Dipole=0.2776242,0.,0.1488457\Quadrupole=1.175304,-3.
 068304,1.893,0.,0.0494069,0.\PG=CS [SG(C2H3Se1)]\\@
```

CH_2=CH-Se• M062X

```
1\1\GINC-GRA581\FOpt\UM062X\6-311+G(d,p)\C2H3Se1(2)\MEISTERP\05-Nov-20
 18\0\\# opt freq=noraman um062x/6-311+g(d,p)\\Methyl Selenyl Radical f
 rom methyl thiyl radical for 2A' symmetry\\0,2\C,0.0052917398,0.,0.014
 412658\H,0.00175537,0.,1.1009081048\C,1.140394097,0.,-0.6754029316\H,2
 .0905509535,0.,-0.1551490441\H,1.1631278713,0.,-1.7587338924\Se,-1.739
 2420672,0.,-0.7223375631\\Version=EM64L-G09RevE.01\State=2-A'\HF=-2479
 .4900993\S2=0.754549\S2-1=0.\S2A=0.750012\RMSD=8.852e-09\RMSF=5.160e-0
 5\Dipole=0.3039766,0.,0.1750035\Quadrupole=1.2630898,-3.3064204,2.0433
 306,0.,0.0176193,0.\PG=CS [SG(C2H3Se1)]\\@
```

```
1\1\GINC-GRA581\FOpt\UM062X\6-311G(df,p)\C2H3Se1(2)\MEISTERP\05-Nov-20
 18\0\\# opt freq=noraman um062x/6-311g(df,p)\\Methyl Selenyl Radical f
 rom methyl thiyl radical for 2A' symmetry\\0,2\C,0.0054599326,0.,0.011
```

0413647\H,0.0024590861,0.,1.0976395361\C,1.1388062895,0.,-0.6751246458
\H,2.0893282334,0.,-0.1554494034\H,1.164254505,0.,-1.758490277\Se,-1.7
35725883,0.,-0.7204031422\\Version=EM64L-G09RevE.01\State=2-A'\HF=-247
9.4941461\S2=0.754535\S2-1=0.\S2A=0.750012\RMSD=4.896e-09\RMSF=3.766e-
05\Dipole=0.3020415,0.,0.1713555\Quadrupole=1.1958765,-3.0839633,1.888
0867,0.,0.0312706,0.\PG=CS [SG(C2H3Se1)]\\@

1\1\GINC-GRA581\FOpt\UM062X\6-311+G(2df,p)\C2H3Se1(2)\MEISTERP\05-Nov-
2018\0\\# opt freq=noraman um062x/6-311+g(2df,p)\\Methyl Selenyl Radic
al from methyl thiyl radical for 2A' symmetry\\0,2\C,0.0051718571,0.,0
.0123179087\H,0.0033817159,0.,1.098229814\C,1.1377140015,0.,-0.6738939
137\H,2.087609337,0.,-0.1545186181\H,1.1609452518,0.,-1.7566983925\Se,
-1.7332328322,0.,-0.7213664859\\Version=EM64L-G09RevE.01\State=2-A'\HF
=-2479.4955985\S2=0.7549\S2-1=0.\S2A=0.750015\RMSD=5.944e-09\RMSF=3.57
3e-05\Dipole=0.2971254,0.,0.1678735\Quadrupole=1.1915257,-3.1420333,1.
9505076,0.,0.0255822,0.\PG=CS [SG(C2H3Se1)]\\@

1\1\GINC-GRA581\FOpt\UM062X\6-311++G(3df,3pd)\C2H3Se1(2)\MEISTERP\05-N
ov-2018\0\\# opt freq=noraman um062x/6-311++g(3df,3pd)\\Methyl Selenyl
 Radical from methyl thiyl radical for 2A' symmetry\\0,2\C,0.005664204
,0.,0.0108583693\H,0.004540499,0.,1.094998291\C,1.1387450945,0.,-0.674
9313896\H,2.0861512079,0.,-0.1548117062\H,1.1623884016,0.,-1.755836939
9\Se,-1.7333752344,0.,-0.7203410058\\Version=EM64L-G09RevE.01\State=2-
A'\HF=-2479.5008728\S2=0.754918\S2-1=0.\S2A=0.750015\RMSD=8.246e-09\RM
SF=3.438e-05\Dipole=0.2875477,0.,0.1580532\Quadrupole=1.1648642,-3.065
5005,1.9006363,0.,-0.01433,0.\PG=CS [SG(C2H3Se1)]\\@

CH_2=CH-Se$^\bullet$ M08HX

1\1\GINC-ORC66\FOpt\UM08HX\6-311+G(d,p)\C2H3Se1(2)\MEISTERP\20-Aug-201
7\0\\# opt freq=noraman m08hx/6-311+g(d,p)\\CH2CHSeH\\0,2\C,-2.1469377
894,-0.2874632896,0.0221269345\H,-2.0702328755,-1.369622772,0.10154389
89\H,-3.1448871279,0.1434167048,-0.0100820359\C,-1.0799749466,0.503713
5643,-0.0353138296\H,-1.1816348641,1.5875264117,-0.114933295\Se,0.7261
956036,-0.0494076191,0.0062633271\\Version=EM64L-G16RevA.03\State=2-A\
HF=-2479.501094\S2=0.753012\S2-1=0.\S2A=0.750006\RMSD=4.368e-09\RMSF=2
.333e-05\Dipole=-0.3091125,0.1429018,-0.0106957\Quadrupole=1.2404587,1
.9947634,-3.2352221,-0.1221785,0.0115849,-0.3860173\PG=C01 [X(C2H3Se1)
]\\@

1\1\GINC-ORC25\FOpt\UM08HX\6-311G(df,p)\C2H3Se1(2)\MEISTERP\20-Aug-201
7\0\\# opt freq=noraman m08hx/6-311g(df,p)\\CH2CHSeH\\0,2\C,-2.1447812
957,-0.2860168716,0.0220225499\H,-2.0698489289,-1.3682696294,0.1014750
882\H,-3.1431633516,0.1436694736,-0.010155444\C,-1.0797916142,0.501596
5125,-0.0351326907\H,-1.1818938932,1.5852685417,-0.1147553436\Se,0.722
0070836,-0.0480850268,0.0061508402\\Version=EM64L-G16RevA.03\State=2-A
\HF=-2479.5053675\S2=0.753095\S2-1=0.\S2A=0.750007\RMSD=4.048e-09\RMSF
=3.411e-05\Dipole=-0.3084903,0.1413872,-0.0106018\Quadrupole=1.1795081
,1.8256788,-3.005187,-0.1221339,0.0114131,-0.3567646\PG=C01 [X(C2H3Se1
)]\\@

1\1\GINC-ORC20\FOpt\UM08HX\6-311+G(2df,p)\C2H3Se1(2)\MEISTERP\20-Aug-2
017\0\\# opt freq=noraman m08hx/6-311+g(2df,p)\\CH2CHSeH\\0,2\C,-2.144
1976097,-0.2855820668,0.0219838081\H,-2.0680090081,-1.3670056141,0.101
3908944\H,-3.1416974494,0.1441574761,-0.0101958039\C,-1.079701633,0.50
15797197,-0.0351179986\H,-1.1835384818,1.5843686051,-0.1146994421\Se,0
.719672182,-0.04935512,0.006243542\\Version=EM64L-G16RevA.03\State=2-A

\HF=-2479.5075342\S2=0.753446\S2-1=0.\S2A=0.750008\RMSD=6.149e-09\RMSF
=3.690e-05\Dipole=-0.2971701,0.135409,-0.0101515\Quadrupole=1.1568174,
1.9005766,-3.0573939,-0.1225228,0.0116167,-0.3654848\PG=C01 [X(C2H3Se1
)]\\@

1\1\GINC-ORC16\FOpt\UM08HX\6-311++G(3df,3pd)\C2H3Se1(2)\MEISTERP\20-Au
g-2017\0\\# opt freq=noraman m08hx/6-311++g(3df,3pd)\\CH2CHSeH\\0,2\C,
-2.1448414219,-0.2860127116,0.0220157902\H,-2.0686227439,-1.3648866833
,0.1012270344\H,-3.1394530934,0.1441832183,-0.0101818236\C,-1.08017221
05,0.5013366738,-0.0351102918\H,-1.183835173,1.5818443057,-0.114506158
6\Se,0.7194526427,-0.0483018029,0.0061604493\\Version=EM64L-G16RevA.03
\State=2-A\HF=-2479.5133542\S2=0.753428\S2-1=0.\S2A=0.750008\RMSD=4.33
3e-10\RMSF=3.342e-05\Dipole=-0.2938112,0.1302863,-0.0097596\Quadrupole
=1.1423936,1.874619,-3.0170127,-0.0868308,0.0089682,-0.3603825\PG=C01
[X(C2H3Se1)]\\@

CH_2=CH-Se$^{\bullet}$ QCISD

1\1\GINC-GRA355\FOpt\UQCISD-FC\6-311+G(2df,p)\C2H3Se1(2)\MEISTERP\05-N
ov-2018\0\\# opt freq=noraman qcisd/6-311+g(2df,p)\\Methyl Selenyl Rad
ical from methyl thiyl radical for 2A' symmetry\\0,2\C,-0.0070194231,0
.,0.0181874214\H,-0.0094089537,0.,1.1060012718\C,1.1337853725,0.,-0.66
42088133\H,2.0826703619,0.,-0.139090117\H,1.1669855357,0.,-1.748391156
3\Se,-1.7528925817,0.,-0.7210206694\\Version=EM64L-G09RevE.01\State=2-
A'\HF=-2477.2639359\MP2=-2477.7035868\MP3=-2477.7342258\MP4D=-2477.746
3507\MP4DQ=-2477.7337871\PUHF=-2477.2675182\PMP2-0=-2477.7061955\PMP3-
0=-2477.7358834\MP4SDQ=-2477.7380607\QCISD=-2477.7390127\S2=0.772213\S
2-1=0.760027\S2A=0.750343\RMSD=4.833e-09\RMSF=4.367e-05\Dipole=0.30230
17,0.,0.1589669\PG=CS [SG(C2H3Se1)]\\@

CH_2=CH-Se-Se$^{\bullet}$ B3LYP

1\1\GINC-ORC10\FOpt\UB3LYP\6-311+G(d,p)\C2H3Se2(2)\MEISTERP\23-Aug-201
7\0\\# opt freq=noraman b3lyp/6-311+g(d,p)\\CH2CHSeSeH\\0,2\C,-3.02817
24098,0.7694035542,-0.0000537021\H,-3.5394555076,-0.1875587309,-0.0001
058283\H,-3.6510265585,1.6575210366,-0.0000408839\C,-1.7017488302,0.87
74405387,-0.0000066817\H,-1.170683188,1.822660773,0.000044689\Se,-0.54
6854728,-0.6658629498,-0.0000229189\Se,1.5362902222,0.2354337781,0.000
0323259\\Version=EM64L-G09RevE.01\State=2-A\HF=-4881.1223272\S2=0.7541
97\S2-1=0.\S2A=0.750014\RMSD=7.578e-09\RMSF=6.514e-05\Dipole=-0.921242
2,0.1790362,-0.0000126\Quadrupole=2.5728639,0.0035781,-2.576442,-0.258
4059,0.0001246,0.0000868\PG=C01 [X(C2H3Se2)]\\@

1\1\GINC-ORC4\FOpt\UB3LYP\6-311G(df,p)\C2H3Se2(2)\MEISTERP\23-Aug-2017
\0\\# opt freq=noraman b3lyp/6-311g(df,p)\\CH2CHSeSeH\\0,2\C,-3.026361
5438,0.7574292281,-0.0000532996\H,-3.532979588,-0.2017533197,-0.000104
8116\H,-3.6551403508,1.6410614981,-0.0000403183\C,-1.7039344111,0.8706
344644,-0.0000074871\H,-1.1775380917,1.8182947459,0.0000433342\Se,-0.5
444834763,-0.6619253061,-0.0000247657\Se,1.5260524617,0.2362756893,0.0
000333481\\Version=EM64L-G09RevE.01\State=2-A\HF=-4881.1293915\S2=0.75
4588\S2-1=0.\S2A=0.750017\RMSD=6.032e-09\RMSF=6.897e-05\Dipole=-0.9208
983,0.1766491,-0.0000129\Quadrupole=2.3860351,0.0009806,-2.3870157,-0.
37873,0.0001112,0.0000776\PG=C01 [X(C2H3Se2)]\\@

1\1\GINC-ORC4\FOpt\UB3LYP\6-311+G(2df,p)\C2H3Se2(2)\MEISTERP\23-Aug-20
17\0\\# opt freq=noraman b3lyp/6-311+g(2df,p)\\CH2CHSeSeH\\0,2\C,-3.01
95917524,0.7619056301,-0.0000531273\H,-3.5263573572,-0.196478953,-0.00

01037467\H,-3.6465573091,1.6461023949,-0.0000400694\C,-1.6975923559,0.
8728193652,-0.0000084451\H,-1.1719856272,1.8202665009,0.0000415927\Se,
-0.5427383449,-0.6602985153,-0.0000261362\Se,1.5289907466,0.2314005772
,0.000034932\\Version=EM64L-G09RevE.01\State=2-A\HF=-4881.127491\S2=0.
754999\S2-1=0.\S2A=0.75002\RMSD=5.424e-09\RMSF=7.019e-05\Dipole=-0.908
6963,0.1869332,-0.0000133\Quadrupole=2.5303845,-0.020562,-2.5098225,-0
.30637,0.0001195,0.0000817\PG=C01 [X(C2H3Se2)]\\@

1\1\GINC-ORC10\FOpt\UB3LYP\6-311++G(3df,3pd)\C2H3Se2(2)\MEISTERP\23-Au
g-2017\0\\# opt freq ub3lyp/6-311++g(3df,3pd)\\CH2CHSeSeH\\0,2\C,-3.01
9763902,0.7624300887,-0.0000535027\H,-3.5263399961,-0.1941393476,-0.00
01049829\H,-3.6441253202,1.6463882315,-0.0000401671\C,-1.6975414139,0.
8722229806,-0.0000079498\H,-1.1726340645,1.8183311036,0.0000429336\Se,
-0.5428048219,-0.660762952,-0.0000257043\Se,1.5290895187,0.2324628952,
0.0000343731\\Version=EM64L-G09RevE.01\State=2-A\HF=-4881.1367399\S2=0
.755043\S2-1=0.\S2A=0.750021\RMSD=7.299e-09\RMSF=7.104e-05\Dipole=-0.9
063425,0.1749213,-0.0000135\Quadrupole=2.4885581,-0.0601761,-2.428382,
-0.2982173,0.0001179,0.0000792\PG=C01 [X(C2H3Se2)]\\@

CH_2=CH-Se-Se• B3PW91

1\1\GINC-ORC10\FOpt\UB3PW91\6-311+G(d,p)\C2H3Se2(2)\MEISTERP\23-Aug-20
17\0\\# opt freq=noraman ub3pw91/6-311+g(d,p)\\CH2CHSeSeH\\0,2\C,3.013
9491481,0.7580313058,0.00008377\H,3.5227636035,-0.2011935485,0.0001629
423\H,3.6381107537,1.6461481431,0.0000643316\C,1.6881321953,0.86911852
94,0.0000126052\H,1.1589372438,1.8168272783,-0.0000653784\Se,0.5395134
888,-0.6619135858,0.000037911\Se,-1.5236484331,0.2340018779,-0.0000511
817\\Version=EM64L-G09RevE.01\State=2-A\HF=-4881.0491964\S2=0.754861\S
2-1=0.\S2A=0.750019\RMSD=7.065e-09\RMSF=7.725e-05\Dipole=0.9256601,0.1
871501,0.0000204\Quadrupole=2.6130685,0.0269171,-2.6399857,0.2799586,0
.000197,-0.0001357\PG=C01 [X(C2H3Se2)]\\@

1\1\GINC-ORC10\FOpt\UB3PW91\6-311G(df,p)\C2H3Se2(2)\MEISTERP\23-Aug-20
17\0\\# opt freq=noraman ub3pw91/6-311g(df,p)\\CH2CHSeSeH\\0,2\C,3.012
4248709,0.7471947658,0.0000833006\H,3.517144065,-0.2138894939,0.000161
4584\H,3.6415631945,1.631462644,0.0000634332\C,1.689935699,0.862620343
1,0.0000141199\H,1.1651351208,1.8125949349,-0.0000629589\Se,0.53750039
96,-0.6583530749,0.000041001\Se,-1.5135603499,0.2342198811,-0.00005335
41\\Version=EM64L-G09RevE.01\State=2-A\HF=-4881.0567013\S2=0.755428\S2
-1=0.\S2A=0.750024\RMSD=6.477e-09\RMSF=8.343e-05\Dipole=0.9276945,0.18
36729,0.0000211\Quadrupole=2.4377214,0.0301635,-2.4678848,0.3902506,0.
0001781,-0.0001233\PG=C01 [X(C2H3Se2)]\\@

1\1\GINC-ORC10\FOpt\UB3PW91\6-311+G(2df,p)\C2H3Se2(2)\MEISTERP\23-Aug-
2017\0\\# opt freq=noraman ub3pw91/6-311+g(2df,p)\\CH2CHSeSeH\\0,2\C,3
.0057587227,0.7509757857,0.0000826112\H,3.5095449443,-0.210090652,0.00
01601583\H,3.6344538328,1.6350774556,0.0000625171\C,1.6841749343,0.865
2060906,0.000014243\H,1.1595671171,1.8147849389,-0.0000625959\Se,0.536
0550727,-0.6565797778,0.000041874\Se,-1.5165976239,0.229781159,-0.0000
558078\\Version=EM64L-G09RevE.01\State=2-A\HF=-4881.0543473\S2=0.75594
5\S2-1=0.\S2A=0.750029\RMSD=7.215e-09\RMSF=8.442e-05\Dipole=0.9108266,
0.196193,0.0000211\Quadrupole=2.596206,-0.0057082,-2.5904979,0.3170075
,0.0001911,-0.00013\PG=C01 [X(C2H3Se2)]\\@

1\1\GINC-ORC10\FOpt\UB3PW91\6-311++G(3df,3pd)\C2H3Se2(2)\MEISTERP\23-A
ug-2017\0\\# opt freq=noraman ub3pw91/6-311++g(3df,3pd)\\CH2CHSeSeH\\0
,2\C,3.0059355279,0.7513629737,0.0000830148\H,3.5096072178,-0.20815820

38,0.0001604617\H,3.6322949847,1.6353768824,0.0000633179\C,1.684144493
4,0.8646657067,0.000014095\H,1.1601029404,1.8131371229,-0.0000626304\S
e,0.5361948149,-0.6569833394,0.0000407316\Se,-1.516514979,0.2307938575
,-0.0000549905\\Version=EM64L-G09RevE.01\State=2-A\HF=-4881.0636188\S2
=0.755997\S2-1=0.\S2A=0.750029\RMSD=5.674e-09\RMSF=8.481e-05\Dipole=0.
908745,0.1838096,0.0000215\Quadrupole=2.5604194,-0.0423269,-2.5180925,
0.308003,0.0001883,-0.0001238\PG=C01 [X(C2H3Se2)]\\@

CH_2=CH-Se-Se$^{\bullet}$ wB97XD

1\1\GINC-ORC280\FOpt\UwB97XD\6-311+G(d,p)\C2H3Se2(2)\MEISTERP\23-Aug-2
017\0\\# opt freq=noraman uwb97xd/6-311+g(d,p)\\CH2CHSeSeH\\0,2\C,-2.9
980719596,0.7652139769,-0.0004352139\H,-3.5071143943,-0.1935319092,-0.
0012534246\H,-3.6202155978,1.6535506394,-0.0001112906\C,-1.6758362408,
0.8739712592,0.0001847607\H,-1.1490794811,1.8222759415,0.001009097\Se,
-0.5346777212,-0.6638376829,-0.0002194906\Se,1.5174793947,0.2343437753
,0.000127562\\Version=EM64L-G09RevE.01\State=2-A\HF=-4881.1344555\S2=0
.756424\S2-1=0.\S2A=0.750032\RMSD=9.508e-09\RMSF=5.327e-05\Dipole=-0.8
752182,0.1939588,0.0000059\Quadrupole=2.6259374,0.0028743,-2.6288116,-
0.2420563,0.0011674,0.0016048\PG=C01 [X(C2H3Se2)]\\@

1\1\GINC-ORC278\FOpt\UwB97XD\6-311G(df,p)\C2H3Se2(2)\MEISTERP\23-Aug-2
017\0\\# opt freq=noraman uwb97xd/6-311g(df,p)\\CH2CHSeSeH\\0,2\C,-2.9
94602385,0.7650332129,-0.0004323419\H,-3.5021788164,-0.194216849,-0.00
12445154\H,-3.6196597193,1.6510220337,-0.0001041028\C,-1.6754398818,0.
8746285404,0.0001773616\H,-1.1503351327,1.8236706631,0.0009992708\Se,-
0.5344605955,-0.6571099921,-0.0002365195\Se,1.5091605308,0.2289583911,
0.0001428473\\Version=EM64L-G09RevE.01\State=2-A\HF=-4881.142304\S2=0.
757032\S2-1=0.\S2A=0.750039\RMSD=7.362e-09\RMSF=5.327e-05\Dipole=-0.88
20168,0.1951488,0.000005\Quadrupole=2.4240799,0.0122279,-2.4363078,-0.
3702122,0.0010444,0.0014891\PG=C01 [X(C2H3Se2)]\\@

1\1\GINC-ORC276\FOpt\UwB97XD\6-311+G(2df,p)\C2H3Se2(2)\MEISTERP\23-Aug
-2017\0\\# opt freq=noraman uwb97xd/6-311+g(2df,p)\\CH2CHSeSeH\\0,2\C,
-2.9934810968,0.7649474126,-0.000429321\H,-3.4992965611,-0.1946315268,
-0.0012414344\H,-3.6188438375,1.6501841825,-0.0001027094\C,-1.67527463
21,0.8749631417,0.0001755784\H,-1.1521562364,1.8245779914,0.0009954214
\Se,-0.5361805072,-0.65521908,-0.000243147\Se,1.507716871,0.2271638786
,0.000147612\\Version=EM64L-G09RevE.01\State=2-A\HF=-4881.14003\S2=0.7
57669\S2-1=0.\S2A=0.750044\RMSD=7.065e-09\RMSF=5.240e-05\Dipole=-0.866
2404,0.2030181,-0.0000036\Quadrupole=2.6362785,-0.0346608,-2.6016177,-
0.2828209,0.0011402,0.0015523\PG=C01 [X(C2H3Se2)]\\@

1\1\GINC-ORC275\FOpt\UwB97XD\6-311++G(3df,3pd)\C2H3Se2(2)\MEISTERP\23-
Aug-2017\0\\# opt freq=noraman uwb97xd/6-311++g(3df,3pd)\\CH2CHSeSeH\\
0,2\C,-2.9938839629,0.7650250074,-0.0004342325\H,-3.4989001859,-0.1930
632874,-0.001253256\H,-3.6168996105,1.6498039533,-0.0001118718\C,-1.67
55242595,0.874386228,0.0001850172\H,-1.1535709197,1.8229023879,0.00101
21677\Se,-0.5364104809,-0.6555411535,-0.0002156431\Se,1.5076734194,0.2
284728643,0.0001198184\\Version=EM64L-G09RevE.01\State=2-A\HF=-4881.14
9677\S2=0.757793\S2-1=0.\S2A=0.750046\RMSD=8.364e-09\RMSF=5.205e-05\Di
pole=-0.8671039,0.1922389,-0.0000028\Quadrupole=2.623601,-0.0695829,-2
.5540181,-0.2817739,0.0011362,0.0015089\PG=C01 [X(C2H3Se2)]\\@

CH_2=CH-Se-Se$^{\bullet}$ M062X

1\1\GINC-ORC102\FOpt\UM062X\6-311+G(d,p)\C2H3Se2(2)\MEISTERP\23-Aug-20

17\0\\# opt freq=noraman m062x/6-311+g(d,p)\\CH2CHSeSeH\\0,2\C,2.98986
30046,0.7714797624,0.000060353\H,3.4998505479,-0.1857031763,0.00010059
38\H,3.6063505309,1.6629274993,0.0000619615\C,1.6676445812,0.872987452
1,0.0000113495\H,1.1295810171,1.8141225064,-0.0000274841\Se,0.53721462
59,-0.6750883574,0.0000003109\Se,-1.5156853076,0.2488723136,-0.0000120
845\\Version=EM64L-G09RevE.01\State=2-A\HF=-4881.0760388\S2=0.755446\S
2-1=0.\S2A=0.750023\RMSD=4.249e-09\RMSF=7.156e-05\Dipole=0.7436726,0.2
293965,0.000008\Quadrupole=2.5996578,-0.0095177,-2.5901401,0.2151755,0
.0001207,-0.0000519\PG=C01 [X(C2H3Se2)]\\@

1\1\GINC-ORC19\FOpt\UM062X\6-311G(df,p)\C2H3Se2(2)\MEISTERP\23-Aug-201
7\0\\# opt freq=noraman m062x/6-311g(df,p)\\CH2CHSeSeH\\0,2\C,2.986632
5372,0.7712713434,0.0000597902\H,3.4967633749,-0.1859059055,0.00009915
81\H,3.6054068757,1.6611522146,0.0000610304\C,1.6675712892,0.872564387
,0.0000125512\H,1.1314142447,1.8149138052,-0.0000256453\Se,0.536406062
7,-0.6688446762,0.0000028087\Se,-1.5093753845,0.2444468315,-0.00001469
34\\Version=EM64L-G09RevE.01\State=2-A\HF=-4881.0835177\S2=0.755371\S2
-1=0.\S2A=0.750022\RMSD=2.623e-09\RMSF=7.102e-05\Dipole=0.7403581,0.23
33358,0.0000081\Quadrupole=2.4137527,-0.0125961,-2.4011566,0.3379147,0
.0001134,-0.0000465\PG=C01 [X(C2H3Se2)]\\@

1\1\GINC-ORC10\FOpt\UM062X\6-311+G(2df,p)\C2H3Se2(2)\MEISTERP\23-Aug-2
017\0\\# opt freq=noraman m062x/6-311+g(2df,p)\\CH2CHSeSeH\\0,2\C,2.98
58590003,0.7713295013,0.0000593183\H,3.4931287152,-0.1865799602,0.0000
977105\H,3.6048947661,1.6601867192,0.0000600296\C,1.6676096323,0.87336
70187,0.000013729\H,1.1328895785,1.8158168611,-0.0000237213\Se,0.53832
82822,-0.6668791177,0.0000053753\Se,-1.5078909746,0.2423569774,-0.0000
174412\\Version=EM64L-G09RevE.01\State=2-A\HF=-4881.0827754\S2=0.75651
6\S2-1=0.\S2A=0.750031\RMSD=8.583e-09\RMSF=7.025e-05\Dipole=0.7210369,
0.2347277,0.0000089\Quadrupole=2.5909467,-0.0497996,-2.5411471,0.25325
63,0.0001179,-0.0000486\PG=C01 [X(C2H3Se2)]\\@

1\1\GINC-ORC102\FOpt\UM062X\6-311++G(3df,3pd)\C2H3Se2(2)\MEISTERP\23-A
ug-2017\0\\# opt freq=noraman m062x/6-311++g(3df,3pd)\\CH2CHSeSeH\\0,2
\C,2.986349576,0.7714237832,0.0000592732\H,3.493215568,-0.1845580013,0
.0000974278\H,3.6029564547,1.6597371447,0.0000598584\C,1.6677855064,0.
8725581719,0.0000138848\H,1.1343085383,1.8136858904,-0.0000233517\Se,0
.5380032502,-0.6670687011,0.0000058382\Se,-1.5077998937,0.2438197123,-
0.0000179307\\Version=EM64L-G09RevE.01\State=2-A\HF=-4881.0918\S2=0.75
6581\S2-1=0.\S2A=0.750032\RMSD=3.094e-09\RMSF=6.796e-05\Dipole=0.72606
18,0.2183963,0.0000093\Quadrupole=2.561635,-0.0883173,-2.4733177,0.249
0771,0.0001163,-0.0000465\PG=C01 [X(C2H3Se2)]\\@

CH_2=CH-Se-Se• M08HX

1\1\GINC-ORC55\FOpt\UM08HX\6-311+G(d,p)\C2H3Se2(2)\MEISTERP\25-Aug-201
7\0\\# opt freq=noraman m08hx/6-311+g(d,p)\\CH2CHSeSe radical\\0,2\C,2
.9887876484,0.7608391796,0.0000568659\H,3.4959746619,-0.2029184932,0.0
00089775\H,3.6124827391,1.652496207,0.0000559988\C,1.6659246135,0.8683
683708,0.0000195885\H,1.1255635063,1.8136595424,-0.0000133143\Se,0.528
8003481,-0.6749947624,0.0000190909\Se,-1.5098485174,0.2493059558,-0.00
00320048\\Version=EM64L-G16RevA.03\State=2-A\HF=-4881.0893953\S2=0.753
608\S2-1=0.\S2A=0.75001\RMSD=2.290e-09\RMSF=5.760e-05\Dipole=0.8647203
,0.190733,0.0000146\Quadrupole=2.5414715,0.0619695,-2.603441,0.2806975
,0.0001178,-0.0000459\PG=C01 [X(C2H3Se2)]\\@

1\1\GINC-ORC30\FOpt\UM08HX\6-311G(df,p)\C2H3Se2(2)\MEISTERP\25-Aug-201
7\0\\# opt freq=noraman m08hx/6-311g(df,p)\\CH2CHSeSe radical\\0,2\C,2
.985363471,0.7606877408,0.00005641\H,3.4924103826,-0.2030758029,0.0000
886166\H,3.6112647503,1.6507094095,0.0000551693\C,1.6658992049,0.86793
21116,0.0000205033\H,1.1272726081,1.8141585857,-0.0000117626\Se,0.5292
328924,-0.66928539,0.0000212259\Se,-1.5037583093,0.2456293453,-0.00003
41625\\Version=EM64L-G16RevA.03\State=2-A\HF=-4881.0969\S2=0.75363\S2-
1=0.\S2A=0.75001\RMSD=8.144e-09\RMSF=6.115e-05\Dipole=0.8458262,0.1994
214,0.0000147\Quadrupole=2.3688154,0.0597089,-2.4285243,0.3761356,0.00
01079,-0.0000402\PG=C01 [X(C2H3Se2)]\\@

1\1\GINC-ORC29\FOpt\UM08HX\6-311+G(2df,p)\C2H3Se2(2)\MEISTERP\25-Aug-2
017\0\\# opt freq=noraman m08hx/6-311+g(2df,p)\\CH2CHSeSe radical\\0,2
\C,2.9843733818,0.760856501,0.0000561739\H,3.4885521862,-0.2035440975,
0.0000878984\H,3.6108188484,1.6495974469,0.0000546863\C,1.6657363885,0
.8686205946,0.0000210526\H,1.1284590375,1.8148120613,-0.0000108634\Se,
0.5314700884,-0.6675057925,0.0000224784\Se,-1.5017249308,0.2439192863,
-0.0000354263\\Version=EM64L-G16RevA.03\State=2-A\HF=-4881.097209\S2=0
.754369\S2-1=0.\S2A=0.750015\RMSD=3.535e-09\RMSF=6.079e-05\Dipole=0.81
22187,0.2005179,0.0000143\Quadrupole=2.5459689,0.014029,-2.559998,0.28
61959,0.0001157,-0.0000437\PG=C01 [X(C2H3Se2)]\\@

1\1\GINC-ORC25\FOpt\UM08HX\6-311++G(3df,3pd)\C2H3Se2(2)\MEISTERP\25-Au
g-2017\0\\# opt freq=noraman m08hx/6-311++g(3df,3pd)\\CH2CHSeSe radica
l\\0,2\C,2.9849672181,0.7608224362,0.0000562155\H,3.4878902936,-0.2013
772334,0.000087944\H,3.6086489681,1.6484804975,0.0000547485\C,1.666020
2821,0.8684715033,0.0000209647\H,1.1296818155,1.812605449,-0.000010921
8\Se,0.5318628358,-0.6674563872,0.0000222529\Se,-1.5013864132,0.245209
7346,-0.0000352038\\Version=EM64L-G16RevA.03\State=2-A\HF=-4881.106586
3\S2=0.754439\S2-1=0.\S2A=0.750015\RMSD=5.692e-09\RMSF=6.095e-05\Dipol
e=0.8291568,0.1873257,0.0000149\Quadrupole=2.5500599,-0.0114516,-2.538
6084,0.3050672,0.0001151,-0.0000422\PG=C01 [X(C2H3Se2)]\\@

CH_2=CH-Se-Se$^{\bullet}$ QCISD

1\1\GINC-ORC340\SP\UQCISD-FC\6-311+G(2df,p)\C2H3Se2(2)\MEISTERP\09-Sep
-2017\0\\# qcisd/6-311+g(2df,p)\\CH2CHSeSeH\\0,2\C,0,2.995099,0.658265
,0.000079\H,0,3.462548,-0.32099,0.000157\H,0,3.656454,1.518208,0.00005
9\C,0,1.678718,0.821897,0.000011\H,0,1.190031,1.790452,-0.000066\Se,0,
0.474426,-0.655835,0.000039\Se,0,-1.543601,0.306758,-0.000059\\Version
=EM64L-G09RevE.01\State=2-A\HF=-4877.092408\MP2=-4877.6826199\MP3=-487
7.7226262\MP4D=-4877.7384616\MP4DQ=-4877.7199844\PUHF=-4877.0997867\PM
P2-0=-4877.6887438\PMP3-0=-4877.7269652\MP4SDQ=-4877.7267844\QCISD=-48
77.7305742\S2=0.839387\S2-1=0.808256\S2A=0.753617\RMSD=4.705e-09\PG=C0
1 [X(C2H3Se2)]\\@
